# Supplementary material for: Diversity of Cellular Slime Molds (Dictyostelids) in the Fanjing Mountain Nature Reserve and Geographical Distribution Comparisons with Other Representative Nature Reserves in Different Climate Zones of China
Source: Microorganisms. 2024 May 24;12(6):1061. doi: 10.3390/microorganisms12061061 (PMC11206006; doi:10.3390/microorganisms12061061)
Supplement: Supplementary file 1 [file microorganisms-12-01061-s001.zip › microorganisms-2994770-supplementary.pdf]

Supplementary Table S1 Species and sequences used in the phylogenetic analyses. Newly generated sequences are indicated in bold.

Species name	Strain number	GenBank accession numbers	
		SSU	ITS
Cavenderia amphispora	BM9A	HQ141521.1	
Cavenderia antarctica	NZ43B	AM168080.1	
Cavenderia aureostabilis	TH10B	MH745571.1	
Cavenderia aureostipes	YA6	AM168083.1	
Cavenderia aureostipes	B15A	KF662199.1	
Cavenderia aureostipes	OH396	KF662201.1	
Cavenderia aureostipes var. *helvetia*	HM592	KF662214.1	
Cavenderia basinodulosa	Mad5-1A	MN338955.1	
Cavenderia bhumiboliana	THC11X	HQ141523.1	
Cavenderia bifurcata	UK5	AM168084.1	
Cavenderia boomerangispora	K26B	HQ141520.1	
Cavenderia canoespora	Mad14-3C	MN338956.1	
Cavenderia delicata	TNS-C-226	AM168093.1	
Cavenderia deminutiva	MexM19A	AM168092.1	
Cavenderia exigua	TNS-C-199	AM168085.1	
Cavenderia fasciculata	SmokOW9A	AM168086.1	
Cavenderia fasciculata	SH3	AM168087.1	
Cavenderia fasciculoidea		GQ496157.1	
Cavenderia fulva	Krug6-5A	MH762953.1	
Cavenderia granulophora	CHII-4	AM168072.1	
Cavenderia helicoidea	Landolt TH19B	OM677255.1	
Cavenderia macrocarpa	MGE2	HQ141519.1	
Cavenderia medusoides	OH592	AM168088.1	
Cavenderia mexicana	MexTF4B1	AM168089.1	
Cavenderia microspora	TNS-C-38	AM168090.1	
Cavenderia minima	Eden1	MH762954.1	
Cavenderia multistipes	UK26b	AM168070.1	
Cavenderia myxobasis	NT2A	HQ141522.1	
Cavenderia parvibrachiata	Landolt TH20C	OM677256.1	
Cavenderia parvibrachiata	Landolt 2019TH20C	OM677257.1	
Cavenderia parvispora	OS126	AM168091.1	
Cavenderia protodigitata	TH18BA	MH745572.1	
Cavenderia protumula	Landolt TH20A	OM677258.1	
Cavenderia pseudoaureostipes	TH39A	HQ141518.1	
Cavenderia stellata	SAB7B	AM168081.1	
Cavenderia subdiscoidea	TH1A	HQ141515.1	
Cavenderia ungulata	Landolt TH18B	OM677259.1	
Acytostelium amazonicum	Landolt X	HQ141510.1	
Acytostelium amazonicum	HN1B1	HQ141511.1	

Acytostelium anastomosans	PP1	AM168115.1	
Acytostelium digitatum	OH517	AM168114.1	
Acytostelium leptosomum	212rjb	HQ141512.1	
Acytostelium leptosomum	FG12	AM168111.1	
Acytostelium longisorophorum	DB10A	AM168109.1	
Acytostelium magnisorum	08A	HQ141513.1	
Acytostelium serpentarium	SAB3A	AM168113.1	
Acytostelium singulare	FDIB	HQ141514.1	
Acytostelium subglobosum	LB1	AM168110.1	
Rostrostelium ellipticum	AE2	AM168112.1	
Heterostelium album	PN500	AM168104.1	
Heterostelium ampliverticillatum		KP167480.1	
Heterostelium anisocaule	NZ47B	AM168096.1	
Heterostelium arachnoideum	YA1	AM168102.1	
Heterostelium asymetricum	OH567	AM168097.1	
Heterostelium asymetricum	HN20C	HQ141503.1	
Heterostelium australicum	NB1AP	HQ141508.1	
Heterostelium boreale	BSB10A	HQ141499.1	
Heterostelium candidum	bsb6b	HQ141498.1	
Heterostelium candidum		AY040337.1	
Heterostelium colligatum	HN13C1	HQ141505.1	
Heterostelium colligatum	OH538	AM168098.1	
Heterostelium cumulocystum		KP167479.1	
Heterostelium equisetoides	B7JB	AM168099.1	
Heterostelium filamentosum	SU-1	AM168100.1	
Heterostelium flexuosum	AU4B	HQ141500.1	
Heterostelium gloeosporum	TCK52	AM168074.1	
Heterostelium granulosum	MF5A	HQ141502.1	
Heterostelium irregularibrachiatum	Krug6-5B	MH762955.1	
Heterostelium lapidosum		KP167477.1	
Heterostelium luridum	LR-2	AM168101.1	
Heterostelium migratissimum		KP167481.1	
Heterostelium multicystogenum	AS2	HQ141506.1	
Heterostelium oculare		HQ141497.1	
Heterostelium oculare	DB4B	AM168079.1	
Heterostelium pallidum	PPHU8	EU004605.1	
Heterostelium pallidum	TNS-C-98	AM168103.1	
Heterostelium pallidum	WS-28		LC159251.2
Heterostelium pallidum	**C345**	**OR294052**	**OR295631**
Heterostelium parvimigratum		KP167483.1	
Heterostelium plurimicrocystogenum		KP167475.1	
Heterostelium pseudocandidum	TNS-C-91	AM168107.1	
Heterostelium pseudocolligatum		KP167474.1	
Heterostelium pseudoplasmodiofascium		KP167482.1	

Heterostelium pseudoplasmodiomagnum		KP167472.1
Heterostelium racemiferum		KP167476.1
Heterostelium radiatum	M26B	MN338953.1
Heterostelium rotatum	QC2C	HQ141501.1
Heterostelium stolonicoideum	K12A	HQ141507.1
Heterostelium tenuissimum	TNS-C-97	AM168105.1
Heterostelium tenuissimum		AY040339.1
Heterostelium tikalense	OH595	AM168106.1
Heterostelium tikalense	HN1C1	HQ141509.1
Heterostelium unguliferum		KP167473.1
Heterostelium versatile	Mad52	MN338954.1
Heterostelium violaceotypum		KP167478.1
Heterostelium recretum	5756-1-17	MW857293.1
Heterostelium multibrachiatum	5916lun	MN752217.1
Heterostelium naviculare	JC SMA	
Tieghemostelium angelicum	38B0	JF892716.1
Tieghemostelium dumosum	OH602	JF892722.1
Tieghemostelium lacteum		AM168045.1
Tieghemostelium menorah	M1	AM168073.1
Tieghemostelium montium	57a	JF892717.1
Tieghemostelium simplex	OH598	JF892720.1
Tieghemostelium unicornutum	OH599	JF892725.1
Hagiwaraea coeruleostipes	CRLC53B	AM168036.1
Hagiwaraea lavandula	B15	AM168047.1
Hagiwaraea radiculata	ML5A	HQ141494.1
Hagiwaraea rhizopodium	AusKY-4	AM168063.1
Hagiwaraea tenebrica	Ong2	MH762956.1
Hagiwaraea vinaceofusca	CC4	AM168062.1
Raperostelium australe	NZ80B	AM168029.1
Raperostelium capillare	37A	JF892721.1
Raperostelium crispum	Eden2	MH762957.1
Raperostelium cymosum	Krug15A	MH762958.1
Raperostelium filiforme	OH603	JF892724.1
Raperostelium gracile	TNS-C-183	AM168078.1
Raperostelium ibericum	214rjb	HQ141495.1
Raperostelium maeandriforme	OH604	JF892719.1
Raperostelium minutum	71-2	AM168051.1
Raperostelium monochasioides	HAG653	AM168052.1
Raperostelium ohioense	Okla4C	HQ141493.1
Raperostelium potamoides	FP1A	AM168069.1
Raperostelium reciprocatum	38A	JF892718.1
Raperostelium reciprocatum var. *transitum*	OH601	JF892723.1
Raperostelium stabile	M12A	MN338957.1
Raperostelium tenue	Pan52	AM168076.1

Raperostelium tenue	PJ6	AM168094.1	
Raperostelium tenue	PR4	AM168075.1	
Speleostelium caveatum	WS695	AM168077.1	
Dictyostelium ammophilum	KBK4A	HQ141478.1	
Dictyostelium aureocephalum	TNS-C-180	AM167876.1	
Dictyostelium aureum	SL1	AM168028.1	
Dictyostelium austroandinum		GQ496158.1	
Dictyostelium barbarae	1-5	MK322959.1	
Dictyostelium barbibulus	Sweden-4R	JX173878.1	
Dictyostelium brefeldianum	TNS-C-115	AM168030.1	
Dictyostelium brunneum	WS700	AM168031.1	
Dictyostelium capitatum	91HO-50	AM168032.1	
Dictyostelium chordatum		GQ496159.1	
Dictyostelium citrinum	OH494	AM168033.1	
Dictyostelium clavatum	TNS-C-189	AM168034.1	
Dictyostelium clavatum	TNS-C-220	AM168035.1	
Dictyostelium crassicaule	93HO-33	AM168037.1	
Dictyostelium dimigraformum	AR5b	AM168038.1	
Dictyostelium discoideum	NC4	AM168071.1	
Dictyostelium discoideum	V34	AM168039.1	
Dictyostelium firmibasis	TNS-C-14	AM168041.1	
Dictyostelium gargantuum		GQ496161.1	
Dictyostelium giganteum	WS589	AM168042.1	
Dictyostelium implicatum	93HO-1	AM168043.1	
Dictyostelium insulinativitatis		MK322958.1	
Dictyostelium intermedium	PJ11	AM168044.1	
Dictyostelium leptosomopsis	Araucaria 1	HM159992.1	
Dictyostelium leptosomum	NZN49A	HQ141480.1	
Dictyostelium longosporum	TNS-C-109	AM168048.1	
Dictyostelium macrocephalum	B33	AM168049.1	
Dictyostelium macrocephalum			DQ463371.1
Dictyostelium medium	TNS-C-205	AM168050.1	
Dictyostelium minimum	2794	MG490369.1	MG490372.1
Dictyostelium brefeldianum	S28b	AM168054.1	
Dictyostelium mucoroides var. *stoloniferum*	FOII-1	AM168055.1	
Dictyostelium multiforme	4007	MG490370.1	MG490373.1
Dictyostelium pseudobrefeldianum	91HO-8	AM168059.1	
Dictyostelium purpureum	QSpu1	FJ424829.1	
Dictyostelium purpureum	QSpu2	FJ424839.1	
Dictyostelium purpureum	QSpu23	FJ424832.1	
Dictyostelium purpureum	QSpu28	FJ424836.1	
Dictyostelium purpureum		DQ340386.1	
Dictyostelium purpureum	QSpu4	FJ424826.1	
Dictyostelium purpureum		AY040335.1	

Dictyostelium purpureum	QSpu36	FJ424828.1	
Dictyostelium purpureum	C143	AM168060.1	
Dictyostelium purpureum	WS321	AM168061.1	
Dictyostelium purpureum	cavender	HQ141481.1	
Dictyostelium purpureum	WS-22		LC159248.1
Dictyostelium purpureum	WS321		HQ141472.1
Dictyostelium purpureum	**C211**	**OR294051**	**OR295628**
Dictyostelium purpureum var. *pseudosessile*	MR273 (4637)	MH280022.1	
Dictyostelium purpureum var. *pseudosessile*	MR273 (4446)	MH280023.1	
Dictyostelium quercibrachium	NZ201B	HQ141479.1	
Dictyostelium robusticaule	5729-bai-2021	MW931857.1	
Dictyostelium robusticaule	5729-huang-2021	MW931856.1	
Dictyostelium robusticaule	**B341**	**OR294050**	**OR295630**
Dictyostelium robustum	TNS-C-219	AM168064.1	
Dictyostelium rosarium	M45	AM168065.1	
Dictyostelium septentrionale	IY49	AM168066.1	
Dictyostelium septentrionale	AK2	AM168067.1	
Dictyostelium sphaerocephalum	GR11	AM168068.1	
Dictyostelium sphaerocephalum	14A		AM282600.1
Dictyostelium valdivianum		GQ496155.1	
Dictyostelium brevicaule	SMA		
Polysphondylium fuscans	Sweden-11D	JX173877.1	
Polysphondylium fuscans	**A241**	**OR294048**	**OR295629**
Polysphondylium laterosorum	AE4	AM168046.1	
Polysphondylium patagonicum		GQ496156.1	
Polysphondylium violaceum	209	HQ141486.1	
Polysphondylium violaceum	WS-17		LC159243.1
Polysphondylium violaceum	P6	AM168108.1	
Polysphondylium acuminatum	OH500 SML		
Coremiostelium polycephalum	Landolt #1130 SS3B	HQ141488.1	
Coremiostelium polycephalum	Landolt #2132 B-9c	HQ141489.1	
Coremiostelium polycephalum	Landolt #1675 GUAM	HQ141490.1	
Coremiostelium polycephalum	MY1-1	AM168056.1	
Synstelium polycarpum	VE1b	AM168057.1	
Synstelium polycarpum	OhioWILDS	AM168058.1	
Physarum polycephalum		X13160.1	

>HQ141521.1 *Cavenderia amphispora* BM9A
AACCTGGTTGATCCTGCCAGTAGTCATATGCTTGTTTCAAGGATTAAGCCATGCATGTCTAAGTATAAGCCCTAGTAC
GGCTAGACTGCAGACGGCTCATTACAACGGTTGCAGCTTACAGAGCATCCGGGTCGCAAGACCTTCTGGATAACCG
CAGTAAATCGGGGCTAATACATACAAACGGAGGGGCGGAGCGGGCAACCGCGAAGCTTCTGCGATGGACACTTAGC
TATTCGACTGACCCCTCACGGGAATGGTTGGAACCGGTTCATATTGCTAATCGACTTGTGGCTTGCCCAAGTCTGATA
AGCTCTATAGGCAACCGCCCTATCAACTTGATGGTAAGGTATTGGCTTACCATGGTTGTAACGGGTAACGGGGAATC
AGGGTTCGATTCCGGAGAGGACGCCTGAGAAACGGCGTCCACATCTACGGGTGGCAGCAGGCGCGTAAATTGCCC
AATCTCAATAGAGAGGAGGCGGTGACAATAAATCCCGATGGCCATGGGGGCAACCCCGGGCCAATCAGAATAAGTA
CACATTAAATACCCTAACCAATATAATTGGAGGGCAAGTCTGGTGCCAGCAGCCGCGGTAATTCCAGCTCCAATAGC
GTATACTAATGTTGTTGCAGTTAAAACGCTCGTAGCTGAACAACTTGATGGCAAATTCAGGTCAATGTGGCACGTCC
ATGGGGGGAAACCTTTATGCGGCAGTGTCCTTGACCGGCTAAGCAGGTAGGTAGTTTGGCGTGTGTAGCAATACATG
CGTCAGGCGATCGACTGTGCATAAACCTTGATGCTCAAGGTAAAGACGACTCTAGTCAAAGATACGCAGTGCATGG
CATTGTGGAACAAGGCGATCCGTAGCTTGGTTGGTGAGCTACGGGTGCAATGATTAATAGGGAGGAGCGGGGGCCT
TCATATTGCCGGGCGAGAGGTGAAATTCGTTGACCCTGGCAAGATGTCCTACAGCGAAAGCATTGGCCAAGTGCCT
CTCCATTAGTCAAGAACGAAAGTTTGGGGATCAAAGACGATCAGATACCGTCGTAGTCCAAACCATAAACTATGTCG
ACCAGCGATTAGGTTCGCCACCTTCTTCGAGAGCAACCTGGCAGCTTGTGGGAAACCATGAGTTCTTGGACTCTGG
GGGGAGTATGGTCGCAAGGCTGAAACTTAAAGGAATTGACGGAAGGGCACACCATGGAGTGGAGCCTGCGGCTTA
ATTTGACTCAACACGGGAAAGCTTACCAAGCTCAGATATGATAAGGATTGACAGACCAAAAGATCTTTCATGATCTC
ATAAGTGGTGGTGCATGGCCGTTCTTAGTTGGTGGAGTGATTTGTCAGGTCAATTCCGGTAACGGACGAGACCTCGA
CCTGCTAACTAGTGGCAGTTGTCTTTTCGCTCGGCGAGGCGTTTTGGGCGTGGAACTTTGATAGGGGCAACTCTATC
TTTGGGAAGCGAACGAGGCTAGTAGTCTGGAGAGGCAGCTGTCCCCATATTAAAATAATCTTCCTAGAGGTACTTCC
AGCTCTAAGTTGGAGGAAGTCCGAGGCAATAACAGGTCTGTGATGCCCTTAGATATCTTGGGCCGCACGCGTGCTAC
AATGTAGGCGCTAATGAGTCGTTCTTAATATCCCGGCACCGCCAAGGTGTCTGGTAATCCCCAATCCCCTGCGTGATT
GGGATTGGTCACTGTAACTGTGACCATCAACGAGGAATTCCTTGTATGCGCGGGTCACTATCCCGCGCAGAATCTGT
CCCTGCCCTTTGTACACACCGCCCGTCGCTCCTACCGATCGAACGGTAAGGTAAAGTGGACGGACAGCTGTCCTTCA
CGGATGGCTGGAAGTCCCATTGAACCTTCGTCGTTAGAGGAAGGAGAAGTCGTAACAAGGTATCGGTAGGTGAACC
TGCAGAAGGATCA

>AM168080.1 *Cavenderia antarctica* NZ43B
AACCTGGTTGATCCTGCCAGTAGTCATATGCTTGTTTCAAGGATTAAGCCATGCATGTCTAAGTATAAGCTCTTGTAC
GGCTAGACTGCAGACGGCTCATTACAACGGTTGTATCTTACAGGGCATCCGGGTCGCAAGACCTTCTGGATAACCGC
AGTAAATCGGGGCTAATACATACAAACGGAGGGATGGAGAGGGCAACCTTGAAGTTTCTGCGATGGACATTTAGCTA
TTCGACCAACCCCGCAAGGGAATGGTTGGAACCGGTTCATATTGCTAATCGACTCTAGCTTGCTAGTAGTCTGATAA
GCCCTATAGACAACCGCCCTATCAACTTGATGGTAAGGTTTTGGCTTACCATGGTTGTAACGGGTAACGGGGAATCA
GGGTTCGATTCCGGAGAGGACGCCTGAGAAACGGCGTCCACATCTACGGGTGGCAGCAGGCGCGTAAATTGCCCA
ATCTCAACAGAGAGGAGGCGGTGACAATAAATCCCGATGGCTTTGGGGGCAACCCCAGGCTAATCAGAATAAGTAC
ACATTAAATCCCTTAACCAATATAATTGGAGGGCAAGTCTGGTGCCAGCAGCCGCGGTAATTCCAGCTCCAATAGCAT
ATACTAATGTTGTTGCAGTTAAAACGCTCGTAGCTTAATATCTTTTGAGCTACTATCGACTGTCATCCTCTTATGTAAG
CCGCAAGGTCCAAGTAAGAGGACCAGTCGGCTCAAACACTGTAGGTCATGCGGCTAGCAATAGTTGCATAATCGAC
TGTGCATAAACCTTGATGCTCAAGGTAGGCCTTTATAGGGTAGATACACAGTGCATGGCATTGTGGAACAAGGCATC
TCGCGGCTTAGTTGGTGGGCCGCGGGGGCAATGATTAATAGGAAGGAGCGGGGGCCTTCATATTGCAGGGCGAGAG
GTGAAATTCGTTGACCCTTGCAAGATGTCCTACAGCGAAAGCATTGGCCAAGTGCCTCCCCATTAGTCAAGAACGA
AAGTTTGGGGATCAAAGACGATCAGATACCGTCGTAGTCCAAACCATAAACTATGTCGACCAGCGATTAGGCGGGCT
ACCTTCTTCGAGAGCTGCCTAGCAGCTTGTGGGAAACCATGAGTGCTTGGACTCTGGGGGGAGTATGGTCGCAAGG
CTGAAACTTAAAGGAATTGACGGAAGGGCACACCATGGAGTGGAGCCTGCGGCTTAATTTGACTCAACACGGGAA
AGCTTACCAAGCTCAGATATGATTAGGATTGACAGACTAAAAGATCTTTCATGATCTCATAAGTGGTGGTGCATGGTC
GTTCTTAGTTGGTGGAGTGATTTGTCAGGTCAATTCCGGTAACGGACGAGACCTCGACCTGCTAACTAGTGGGATTC
ATTCTTTCGATTGACGAGGCAGGTATTGCTTTGATTATAGGGGGCAACTTTTATAGTCGGGTAGTGCTTGTATTAGTCT
GGGAGAGTGGGTTTCAAAAATTTAATTAATCTTCCTAGAGGTACTTCTGGCTCTAAGCCAGAGGAAGTCCGAGGCA
ATAACAGGTCTGTGATGCCCTTAGATATCTTGGGCCGCACGCGTGCTACAATGTAGGCGCTAATGAGTTTATTTACAT
CCATCTCCGCAAGGAGTATGGTAATCTTGGAATCCCCTGCGTGATTGGGCTTGACCACTGTAACTGTGGTCATCAAC
GAGGAATTCCTTGTATGCGCGAGTCACTATCTCGCGCAGAATCTGTCCCTGCCCTTTGTACACGCCGCCCGTCGCTCC
TACCGATCGAACGATCAGGTAAAGTGGACAGACTGAAACCCGCAAGGGTTTTGGAAGTCCATTGAACCTCGCCGT
TTAGAGGAAGGAGAAGTCGTAACAAGGTATCGGTAGGTGAACCTGCAGAAGGATCAA

>MH745571.1 *Cavenderia aureostabilis* TH10B
CATGTCTAAGTATAAGCTCTTGTACGGCTAGACTGCAGACGGCTCATTACAACGGTTGTATCTTCCAGGACATCCGGG
TCGCAAGATCTTCTGGATAACCGCAGTAAATCGGGGCTAATACATACAAACGAAGGGGTGGAGAGGGCAACCTTGA
AGCTTCTGCGATGGACACTTAGCTATTCGACCAACCCCGCAAGGGAGTGGTTGGAACCGGTTCATATTGCTAATCGA
CTCTGGCTTGCCATGAGTCGATAAGTCCTATAGACAACCGCCCTATCAACTTGATGGTAAGGTTTTGGCTTACCATGG
TTGTAACGGGTAACGGGGAATCAGGGTTCGATTCCGGAGAGGACGCCTGAGAAACGGCGTCCACATCTACGGGTGG
CAGCAGGCGCGTAAATTGCCCAATCTCAACAGAGAGGAGGCGGTGACAATAAATCCCGATGGCTTTGGGGGCAACC
CCAGGCTAATCAGAATAAGTACACATTAAATCCCTTAACCAATATAATTGGAGGGCAAGTCTGGTGCCAGCAGCCGC
GGTAATTCCAGCTCCAATAGCATATACTAATGTTGTTGCAGTTAAAACGCTCGTAGCTTAATTTCTTTTGAGCTACTTA
CGGTTGTCAAACCTGCGAAGTCTTCGGACCTAGTAGGAACAACCGGCTCAAACACTGTAGGTCATGGGCTTAGCAA
TAAGTTCATAATCGACTGTGCATAAACCTTGATGCTCAAGGTAGGCCTTTATAGGGTAGATACACAGTGCATGGCATT
GTGGAACAAGGCATCTCGCGGCTTAGTTGGTGGGCCGCGGGGGCAATGATTAATAGGGAGGAGCGGGGGCCTTCAT
ATTGCAGGGCGAGAGGTGAAATTCGTTGACCCTTGCAAGATGTCCGACAGCGAAAGCATTGGCCAAGTGCCTCCCC
ATTAGTCAAGAACGAAAGTTTGGGGATCAAAGACGATCAGATACCGTCGTAGTCCAAACCATAAACTATGTCGACCA
GCGATTAGGCGGGCTACCTTCTTCGAGAGCTGCCTAGCAGCTTGTGGGAAACCATGAGTGCTTGGACTCTGGGGGG
AGTATGGTCGCAAGGCTGAAACTTAAAGGAATTGACGGAAGGGCACACCATGGAGTGGAGCCTGCGGCTTAATTTG
ACTCAACACGGGAAAGCTTACCAAGCTCAGATATGATAAGGATTGACAGACTAAAAGATCTTTCATGATCTCATAAG
TGGTGGTGCATGGTCGTTCTTAGTTGGTGGAGTGATTTGTCAGGTCAATTCCGGTAACGGACGAGACCTCGACCTGC
TAACTAGTGGGATTCATTCCTTCGCTCGACGAGGCGGGCTCTGCTTTTATTATGAGGGCAACTTCATAGTAGGGTAGG

GTTCGTATTAGTCTGGGGGAGTGGGTTTCAAAATTTAATTTAATCTTCCTAGAGGTACTTCTGGCTCTAAGCCAGAGG
AAGTTCGAGGCAATAACAGGTCTGTGATGCCCTTAGATATCTTGGGCCGCACGCGTGCTACAATGTAGGCGCTAATG
AGTCTTAATACATCCAGCTCCGTAAGGAGTCTGGTAATCTTGGAATCCCCTGCGTGATTGGGCTTGACTACTGTAACT
GTGGTCATCAACGAGGAATTCCTTGTATGCGCGAGTCACTATCTCGCGCAGAATCTGTCCCTGCCCTTTGTACACACC
GCCCGTCGCTCCTACCGATCGAACGATCAGGTAAAGTGGACAGACTAGAGGCCGCAAGGCATTTGGAAAGTCCATT
GAACCTCGCCGTTTAGAGGAAGGAGAAGTCGTAACAAGGTATCGGTAGGTGAACCTGCAGAAGGATCAA

>AM168083.1 *Cavenderia aureostipes* YA6
AACCTGGTTGATCCTGCCAGTAGTCATATGCTTGTTTCAAGGATTAAGCCATGCATGTCTAAGTATAAGCTCTTGTAC
GGCTAGACTGCAGACGGCTCATTACAACGGTTGTATCTTCCAGGACATCCGGGTCGCAAGACCTTCTGGATAACCGC
AGTAAATCGGGGCTAATACATACAAACGGAGGGGTAGAGAGGGCAACCTTGAAGCTTCTGCGATGGACAATTAGTT
ATTCGACCAACCCCGCAAGGGAATGGTTGGAACCGGTTCATATTGCTAATCGACTCTAGCTTGCTAGTAGTCTGATAA
GTCCTATAGACAACCGCCCTATCAACTTGATGGTAAGGTTTTGGCTTACCATGGTTGTAACGGGTAACGGGGAATCA
GGGTTCGATTCCGGAGAGGACGCCTGAGAAACGGCGTCCACATCTACGGGTGGCAGCAGGCGCGTAAATTGCCCA
ATCTCAACAGAGAGGAGGCGGCGACAATAAATCCCGATGGCTTTGGGGGCAACCCCAGGCCAATCAGAATAAGTAC
ACATTAAATCCCTTAACCAATATAATTGGAGGGCAAGTCTGGTGCCAGCAGCCGCGGTAATTCCAGCTCCAATAGCAT
ATACTAATGTTGTTGCAGTTAAAACGCTCGTAGCTCAATATCTTTTGAGCGCTTTCGGCCGTTCTCTTTAGCTACCGCA
AGGCTGTTAAAGAACCGGTCGGCTCAAACACTGTAGGTCATGGTAAAGTTAGCAATAGCTTTATCATAATCGACTGT
GCATAAACCTTGATGCTCAAGGTAGGCCTTTTATAGGGTAGATACACAGTGCATGGCATTGTGGAACAAGGCATCTC
GCGGCTTAGTTGGTGGGCCGCGGGGGCAATGATTAATAGGGAGGAGCGGGGGCCTTCATATTGCAGGGCGAGAGGT
GAAATTCGTTGACCCTTGCAAGATGTCCTACAGCGAAAGCATTGGCCAAGTGCCTCCCCATTAGTCAAGAACGAAA
GTTTGGGGATCAAAGACGATCAGATACCGTCGTAGTCCAAACCATAAACTATGTCGACCAGCGATTAGGCGGGCTAC
CTTCTTCGAGAGCTGCCTAGCAGCTTGTGGGAAACCATGAGTGCTTGGACTCTGGGGGGAGTATGGTCGCAAGGCT
GAAACTTAAAGGAATTGACGGAAGGGCACACCATGGAGTGGAGCCTGCGGCTTAATTTGACTCAACACGGGAAAG
CTTACCAAGCTCAGATATGATTAGGATTGACAGACTAAAAGATCTTTCATGATCTCATAAGTGGTGGTGCATGGTCGT
TCTTAGTTGGTGGAGTGATTTGTCAGGTCAATTCCGGTAACGGACGAGACCTCGACCTGCTAACTAGTGGGATTCAT
TCTTTCGATTGACGAGGCAGGTTTTGCTTTGATTATAGGGGGCAACTTCTATAGTCGGGTAGAGCTTGTATTAGTCTG
GGAGAGTGGGTTTCAAAAATTAAAATTAATCTTCCTAGAGGTACTTCTGGCTCTAAGCCAGAGGAAGTCCGAGGCA
ATAACAGGTCTGTGATGCCCTTAGATATCTTGGGCCGCACGCGTGCTACAATGTAGGCGCTAATGAGTTTATTTACAT
CCATCTCCGCAAGGAGTATGGTAATCTTGGAATCCCCTGCGTGATTGGGCTTGACCACTGTAACTGTGGTCATCAAC
GAGGAATTCCTTGTATGCGCGAGTCACTATCTCGCGCAGAATCTGTCCCTGCCCTTTGTACACACCGCCCGTCGCTCC
TACCGATCGAACGATCAGGTAAAGTGGACAGACTGGAACTCGCAAGAGTTCTTGGAAGTCCATTGAACCTCGCCGT
TTAGAGGAAGGAGAAGTCGTAACAAGGTATCGGTAGGTGAACCTGCAGAAGGATCAA

>KF662199.1 *Cavenderia aureostipes* B15A
ATGCATGTCTAAGTATAAGCTCTTGTACGGCTAGACTGCAGACGGCTCATTACAACGGTTGTATCTTCCAGGGCATCC
GGGTCGCAAGACCTTCTGGATAACCGCAGTAAATCGGGGCTAATACATACAAACGGAGGGATGGAGAGGGCAACCT
TGAAGTTTCTGCGATGGACAATTAGTTATTCGACCAACCCCGCAAGGGAACGGTTGGAACCGGTTCATATTGCTAAT
CGACTCTAGCTTGCTAGTAGTCTGATAAGCCCTATAGACAACCGCCCTATCAACTTGATGGTAAGGTTTTGGCTTACC
ATGGTTGTAACGGGTAACGGGGAATCAGGGTTCGATTCCGGAGAGGACGCCTGAGAAACGGCGTCCACATCTACGG
GTGGCAGCAGGCGCGTAAATTGCCCAATCTCAACAGAGAGGAGGCGGTGACAATAAATCCCGATGGCTTTGGGGGC
AACCCCAGGCTAATCAGAATAAGTACACATTAAATCCCTTAACCAATATAATTGGAGGGCAAGTCTGGTGCCAGCAG
CCGCGGTAATTCCAGCTCCAATAGCATATACTAATGTTGTTGCAGTTAAAACGCTCGTAGCTTAATATCTTTTGAGCTA
CTATCGATTGTTATTCCTTTGGATCCTCACGGTGAAGGAGGGAGGCAATCGGCTCAAACACTGTAGGTCATGCGGCT
AGCAATAGTTGCATAATCGACTGTGCATAAACCTTGATGCTCAAGGTAGGCCTTTATAGGGTAGATACACAGTGCATG
GCATTGTGGAACAAGGCATCTCGCGGCTTAGTTGGTGGGCCGCGGGGGCAATGATTAATAGGGAGGAGCGGGGGCC
TTCATATTGCAGGGCGAGAGGTGAAATTCGTTGACCCTTGCAAGATGTCCTACAGCGAAAGCATTGGCCAAGTGCCT
CCCCATTAGTCAAGAACGAAAGTTTGGGGATCAAAGACGATCAGATACCGTCGTAGTCCAAACCATAAACTATGTCG
ACCAGCGATTAGGCGGGCTACCTTCTTCGAGAGCTGCCTAGCAGCTTGTGGGAAACCATGAGTGCTTGGACTCTGG
GGGGAGTATGGTCGCAAGGCTGAAACTTAAAGGAATTGACGGAAGGGCACACCATGGAGTGGAGCCTGCGGCTTA
ATTTGACTCAACACGGGAAAGCTTACCAAGCTCAGATATGATTAGGATTGACAGACTAAAAGATCTTTCATGATCTC
ATAAGTGGTGGTGCATGGTCGTTCTTAGTTGGTGGAGTGATTTGTCAGGTCAATTCCGGTAACGGACGAGACCTCGA
CCTGCTAACTAGTGGGATTCATTCTTTCGATTGACGAGGCAGGTCTTGCTTTGATTATAGGGGGCAACTTTTATAGTC
GGGTACGGTTTGTAGTAGTCTGGAGGAGTGGATTTCAAAAATTAAATTAATCTTCCTAGAGGTACTTCTGGCTCTAAG
CCAGAGGAAGTCCGAGGCAATAACAGGTCTGTGATGCCCTTAGATATCTTGGGCCGCACGCGTGCTACAATGTAGGC
GCTAATGAGTTTATTTACATCCATCTCCGCAAGGAGTATGGTAATCTTGGAATCACCTGCGTGATTGGGCTTGACCAC
TGTAACTGTGGTCATCAACGAGGAATTCCTTGTATGCGCGAGTCACTATCTCGCGCAGAATCTGTCCCTGCCCTTTGT
ACACACCGCCCGTCGCTCCTACCGATCGAACGATCAGGTAAAGTGGACAGACTGGAGCCCGCAAGGGCACTTGGA
AGTCCATTGAACCTCGCCGTTAGAGGAAGA

>KF662201.1 *Cavenderia aureostipes* OH396
TGTATCTTCCAGGGCATCCGGGTCGCAAGACCTTCTGGATAACCGCAGTAAATCGGGGCTAATACATATAAACGGAG
GGATGGAGAGGGCAACCTTGAAGTTTCTGCGATGGACAATTAGTTATTCGACCAACCCCGCAAGGGAATGGTTGGA
ACCGGTTCATATTGCTAATCGACTCTAGCTTGCTAGTAGTCTGATAAGCCCTATAGACAACCGCCCTATCAACTTGATG
GTAAGGTTTTGGCTTACCATGGTTGTAACGGGTAACGGGGAATCAGGGTTCGATTCCGGAGAGGACGCCTGAGAAA
CGGCGTCCACATCTACGGGTGGCAGCAGGCGCGTAAATTGCCCAATCTCAACAGAGAGGAGGCGGTGACAATAAAT
CCCGATGGCTTTGGGGGCAACCCCAGGCTAATCAGAATAAGTACACATTAAATCCCTTAACCAATATAATTGGAGGG
CAAGTCTGGTGCCAGCAGCCGCGGTAATTCCAGCTCCAATAGCATATACTAATGTTGTTGCAGTTAAAACGCTCGTA
GCTGAATATCTTTTGAGCTACTATCGACTGTTTTCCTTCTGGATCTTCGGAAAAGGGAGGGGCAGTCGGCTCAAACA
CTGTAGGTCATGCGGCTAGCAATAGTTGCATAATCGACTGTGCATAAACCTTGATGCTCAAGGTAGGCCTTTATAGGG
TAGATACACAGTGCATGGCATTGTGGAACAAGGCATCTCGCGGCTTAGTTGGTGGGCCGCGGGGGCAATGATTAATA
GGGAGGAGCGGGGGCCTTCATATTGCAGGGCGAGAGGTGAAATTCGTTGACCCTTGCAAGATGTCCTACAGCGAAA
GCATTGGCCAAGTGCCTCCCCATTAGTCAAGAACGAAAGTTTGGGGATCAAAGACGATCAGATACCGTCGTAGTCC
AAACCATAAACTATGTCGACCAGCGATTAGGCGGGCTACCTTCTTCGAGAGCTGCCTAGCAGCTTGTGGGAAACCAT
GAGTGCTTGGACTCTGGGGGGAGTATGGTCGCAAGGCTGAAACTTAAAGGAATTGACGGAAGGGCACACCATGGA

GTGGAGCCTGCGGCTTAATTTGACTCAACACGGGAAAGCTTACCAAGCTCAGATATGATTAGGATTGACAGACTAAA
AGATCTTTCATGATCTCATAAGTGGTGGTGCATGGTCGTTCTTAGTTGGTGGAGTGATTTGTCAGGTCAATTCCGGTA
ACGGACGAGACCTCGACCTGCTAACTAGTGGGATTCATTCTTTCGATTGACGAGGCAGGTCTTGCTTTGATTATAGG
GGGCAACTTCTATAGTCGGGTAGGGTTTGTAGTAGTCTGGAGGAGTGGATTTCAAAAATTAAATTAATCTTCCTAGAG
GTACTTCTGGCTCTAAGCCAGAGGAAGTCCGAGGCAATAACAGGTCTGTGATGCCCTTAGATATCTTGGGCCGCACG
CGTGCTACAATGTAGGCGCTAATGAGTTTATTTACATCCATCTCCGCAAGGAGTATGGTAATCTTGGAATCACCTGCG
TGATTGGGCTTGACCACTGTAACTGTGGTCATCAACGAGGAATTCCTTGTATGCGCGAGTCACTATCTCGCGCAGAA
TCTGTCCCTGCCCTTTGTACACACCGCCCGTCGCTCCTACCGATCGAACGATCAGGTAAAGTGGACAGACTGGAACC
CGCAAGGGTACTTG

>KF662214.1 *Cavenderia aureostipes* var. *helvetia* HM592
TGTATCTTACAGGGCATCCGGGTCGCAAGACCTTCTGGATAACCGCAGTAAATCGGGGCTAATACATACAAACGGAG
GAATGGAGGGGGCAACCTTGAAGTTTCTGCGATGGACATTTAGCTATTCGACCAACCCCGCAAGGGAATGGTTGGA
ACCGGTTCATATTGCTAATCGACTCTAGCTTGCTAGTAGTCTGATAAGCCCTATAGACAACCGCCCTATCAACTTGATG
GTAAGGTTTTGGCTTACCATGGTTGTAACGGGTAACGGGGAATCAGGGTTCGATTCCGGAGAGGACGCCTGAGAAA
CGGCGTCCACATCTACGGGTGGCAGCAGGCGCGTAAATTGCCCAATCTCAACAGAGAGGAGGCGGTGACAATAAAT
CCCGATGGCTTTGGGGGCAACCCCAGGCTAATCAGAATAAGTACACATTAAATCCCCTAACCAATATAATTGGAGGG
CAAGTCTGGTGCCAGCAGCCGCGGTAATTCCAGCTCCAATAGCATATACTAATGTTGTTGCAGTTAAAACGCTCGTA
GCTGAATATCTTTTGAGCTACTATCGGTTGTGTTCTATTGACCCGCAAGGGGAGGTAGCGCAGCCGGCTCAAACACT
GTAGGTCATGGCGTTGGTAGTAATACTGATGTCATAATCGACTGTGCATAAACCTTGATGCTCAAGGTAGGCCTTTAT
AGGGTAGATACACAGTGCATGGCATTGTGGAACAAGGCATCTCGCGGCTTAGTTGGTGGGCCGCGGGGGCAATGAT
TAATAGGGAGGAGCGGGGGCCTTCATATTGCAGGGCGAGAGGTGAAATTCGTTGACCCTTGCAAGATGTCCTACAG
CGAAAGCATTGGCCAAGTGCCTCCCCATTAGTCAAGAACGAAAGTTTGGGGATCAAAGACGATCAGATACCGTCGT
AGTCCAAACCATAAACTATGTCGACCAGCGATTAGGCGGGCTACCTTCTTCGAGAGCTGCCTAGCAGCTTGTGGGAA
ACCATGAGTGCTTGGACTCTGGGGGGAGTATGGTCGCAAGGCTGAAACTTAAAGGAATTGACGGAAGGGCACACC
ATGGAGTGGAGCCTGCGGCTTAATTTGACTCAACACGGGAAAGCTTACCAAGCTCAGATATGATAAGGATTGACAG
ACTAAAAGATCTTTCATGATCTCATAAGTGGTGGTGCATGGTCGTTCTTAGTTGGTGGAGTGATTTGTCAGGTCAATT
CCGGTAACGGACGAGACCTCGACCTGCTAACTAGTGGGATTCATTCCTTCGCTCGACGAGGCAGGCTTTGCTTTGAT
TATAGGGGGCAACCTTTATAGTCGGGTAAGGTTTGTATTAGTCTGGGGGAGTGGGTTTCATATAATTTAATTAATCTTC
CTAGAGGTACTTCTGGCTCTAAGCCAGAGGAAGTCCGAGGCAATAACAGGTCTGTGATGCCCTTAGATATCTTGGGC
CGCACGCGTGCTACAATGTAGGCGCTAATGAGTCTTTATTTATCCAGCTCCGTAAGGAGTCTGGTAATCTTGGAATCC
CCTGCGTGATTGGGCTTGACCACTGTAACTGTGGTCATCAACGAGGAATTCCTTGTATGCGCGAGTCACTATCTCGC
GCAGAATCTGTCCCTGCCCTTTGTACACACCGCCCGTCGCTCCTACCGATCGAACGATCAGGTAAAGTGGACAGACT
TCGACTCGCAAGAGTTT

>MN338955.1 *Cavenderia basinodulosa* Mad5-1A
AGCCATGCATGTCTAAGTAAGCTCTTGCTACTGTTATGACTGCAGACGGCTCATTATCATATCTGTGTATGTATCTTAC
AGGGCATCCGGGTCGCAAGACCTTCTGGATAACCGCAGTAAATCGGGGCTAATACATATAAACGGAGGGATGGAGA
GGGCTACCTTGAAGTTTCTGCGATGGACATTTAGCTATTCGACCTCCCCCGCAAGGGAACGGTTGGAACCGGTTCAT
ATTGCTAATCGACTCTAGCTTGCTAGTTTTCTGATGAGCTCTATAGACAACCGCCCTATCAACTTGATGGTAAGGTTTT
GGCTTACCTTGGTTGTATCGGGTAACGGGGAATCAGGGTTCGATTCCGGAGAGGACGCCTGACAAACGGCGTCCAC
ATCTACGGGTGGCAGCAGGCGCGTAAATTGCCCAATCTCAACAGAGAGGAGGCGGTGACAATAAATCCCGATGGCT
TTGGGGGCAACCCCAGGCCAATCAGAATAAGTACACATTAAATCCCTTAACCAATATAATTGGAGGGCAAGTCTGGT
GCCAGCAGCCGCGGTAATTCCAGCTCCAATAGCATATACTAATGTTGTTGCAGTTAAAACGCTCGTAGCTGAATATCT
TTTGAGCTACTATCGACTACATTCCTTCAAAGGTCGCAAGACTGATGAAGATGTGGTCGGCTCAAACACTGTAGGTG
ATGATATTGGTAGCAATACTAATATCATAATCGACTGTGCATAAACCTTGATGCTCAAGGTAGGCCTTTATAGGGTAGA
TACACAGTGCATGGCATTGTGGAACAAGGCATCTCGCGGCTTAGTTGGTGGGCCGCGGGGGCAATGATTAATAGGG
AGGAGCGGGGGCCTTCATATTGCAGGGCGAGAGGTGAAATTCGTTGACCCTTGCAAGATGTCCGACAGCGAAAGC
ATTGGCCAAGTGCCTCTCCATTAGTCAAGAACGAAAGTTTGGGGATCAAAGACGATCAGATACCGTCGTAGTCCAA
ACCATAAACTATGTCGACCAGCGATTAGGCGGGCTACCTTCTTCGAGAGCTGCCTAGCAGCTTGTGGGAAACCATGA
GTGCTTGGACTCTGGGGGGAGTATGGTCGCAAGGCTGAAACTTAAAGGAATTGACGGAAGGGCACACCATGGAGT
GGAGCCTGCGGCTTAATTTGACTCAACACGGGAAAGCTTACCAAGCTCAGATATGATAAGGATTGACAGACTAAAA
GATCTTTCATGATCTCATAAGTGGTGGTGCATGGTCGTTCTTAGTTGGTGGAGTGATTTGTCAGGTCAATTCCGGTAA
CGGACGAGACCTCGACCTGCTAACTAGTGGGATTCATTCTTTCGATTGACGAGGCGGGTATTGCTTTGATTATGAGG
GGCAACCTTTATAGTCGGGTAATATTTGTAGTAGTCTGGAGGAGTGGGTTTCAGACAATTTAATTAATCTTCCTAGAG
GTACTTCTGGCTCTAAGCCAGAGGAAGTCCGAGGCAATAACAGGTCTGTGATGCCCTTAGATATCTTGGGCCGCACG
CGTGCTACAATGTAGGCGCTAATGAGTCGTAACAATTTCCAGCTCCGTAAGGAGTCTGGTAATCTTGGAATCACCTG
CGTGATTGGGCTTGACTACTGTAACTGTGGTCATCAACGAGGAATTCCTTGTATGCGCGAGTCACTATCTCGCGCAG
AATCTGTCCCTGCCCTTTGTACACACCGCCCGTCGCTCCTACCGATCGAACGATCAGGTAAAATGGACATTGATACCC
TCCGCAGAGTCCTCCCGATGTAATACGGTTATAATTAGACAGGCATGGGTAAGGTGAACCTGCAGAAGGAT

>HQ141523.1 *Cavenderia bhumiboliana* THC11X
AACCTGGTTGATCCTGCCAGTAGTCATATGCTTGTTTCAAGGATTAAGCCATGCATGTCTAAGTATAAGCTCTTGTAC
GGCTAGACTGCAGACGGCTCATTACAACGGTTGTATCTTCCAGGGCATCCGGGTCGCAAGACCTTCTGGATAACCGC
AGTAAATCGGGGCTAATACATACAAACGGAGGGATGGAGAGGGCAACCTTGAAGTTTCTGCGATGGACAATTAGTT
ATTCGACCAACCCCGCAAGGGAATGGTTGGAACCGGTTCATATTGCTAATCGACTCTAGCTTGCTAGTAGTCTGATAA
GCCCTATAGACAACCGCCCTATCAACTTGATGGTAAGGTTTTGGCTTACCATGGTTGTAACGGGTAACGGGGAATCA
GGGTTCGATTCCGGAGAGGACGCCTGAGAAACGGCGTCCACATCTACGGGTGGCAGCAGGCGCGTAAATTGCCCA
ATCTCAACAGAGAGGAGGCGGTGACAATAAATCCCGATGGCTTTGGGGGCAACCCCAGGCTAATCAGAATAAGTAC
ACATTAAATCCCTTAACCAATATAATTGGAGGGCAAGTCTGGTGCCAGCAGCCGCGGTAATTCCAGCTCCAATAGCAT
ATACTAATGTTGTTGCAGTTAAAACGCTCGTAGCTTAATATCTTTTGAGCTACTATCGATTGTTATCCTGCTGACCCGA
AAGGTGATGTAGGAGGCAGTCGGCTCAAACACTGTAGGTCATGCGGCTAGCAATAGTTGCATAATCGACTGTGCATA
AACCTTGATGCTCAAGGTAGGCCTTTATAGGGTAGATACACAGTGCATGGCATTGTGGAACAAGGCATCTCGCGGCT
TAGTTGGTGGGCCGCGGGGGCAATGATTAATAGGGAGGAGCGGGGGCCTTCATATTGCAGGGCGAGAGGTGAAATT
CGTTGACCCTTGCAAGATGTCCTACAGCGAAAGCATTGGCCAAGTGCCTCCCCATTAGTCAAGAACGAAAGTTTGG

GGATCAAAGACGATCAGATACCGTCGTAGTCCAAACCATAAACTATGTCGACCAGCGATTAGGCGGGCTACCTTCTT
CGAGAGCTGCCTAGCAGCTTGTGGGAAACCATGAGTGCTTGGACTCTGGGGGGAGTATGGTCGCAAGGCTGAAACT
TAAAGGAATTGACGGAAGGGCACACCATGGAGTGGAGCCTGCGGCTTAATTTGACTCAACACGGGAAAGCTTACCA
AGCTCAGATATGATTAGGATTGACAGACTAAAAGATCTTTCATGATCTCATAAGTGGTGGTGCATGGTCGTTCTTAGT
TGGTGGAGTGATTTGTCAGGTCAATTCCGGTAACGGACGAGACCTCGACCTGCTAACTAGTGGGATTCATTCTTTCG
ATTGACGAGGCAGGTCTTGCTTTGATTATAGGGGGCAACTTCTATAGTCGGGTACGGTTTGTAGTAGTCTGGAGGAG
TGGATTTCAAAAATTAAATTAATCTTCCTAGAGGTACTTCTGGCTCTAAGCCAGAGGAAGTCCGAGGCAATAACAGG
TCTGTGATGCCCTTAGATATCTTGGGCCGCACGCGTGCTACAATGTAGGCGCTAATGAGTTTATTTACATCCATCTCCG
CAAGGAGTATGGTAATCTTGGAATCACCTGCGTGATTGGGCTTGACCACTGTAACTGTGGTCATCAACGAGGAATTC
CTTGTATGCGCGAGTCACTATCTCGCGCAGAATCTGTCCCTGCCCTTTGTACACACCGCCCGTCGCTCCTACCGATCG
AACGATCAGGTAAAGTGGACAGACTGGAGCCCGCAAGGGTTCTTGGAAGTCCATTGAACCTCGCCGTTTAGAGGA
AGGAGAAGTCGTAACAAGGTATCGGTAGGTGAACCTGCAGAAGGATCA

>AM168084.1 *Cavenderia bifurcata* UK5
AACCTGGTTGATCCTGCCAGTAGTCATATGCTTGTTTCAAGGATTAAGCCATGCATGTCTAAGTATAAGCTCTTGTAC
GGCTAGACTGCAGACGGCTCATTACAACGGTTGTAGCTTCCAGGACATCCGGGTCGCAAGACCTTCTGGATAACCG
CAGTAAATCGGGGCTAATACATACAAACGGAGGGATGGATAGGGCAACCTTGAAGTTTCTGCGATGGACACTTAGCT
ATTCGACCAGCCCTGGCAACAGGAATGGTTGGAACCGGTTCATATTGCTAATCGACTCTAGCTTGCTAGCAGTCTGA
TAAGTCCTATAGACAACCGCCCTATCAACTTGATGGTAAGGTTTTGGCTTACCATGGTTGTAACGGGTAACGGGGAAT
CAGGGTTCGATTCCGGAGAGGACGCCTGAGAAACGGCGTCCACATCTACGGGTGGCAGCAGGCGCGTAAATTGCCC
AATCTCAACAGAGAGGAGGCGGTGACAATAAATCCCGATGGCTTTGGGGGCAACCCCAGGCCAATCAGAATAAGTA
CACATTAAATCCCTTAACCAATATAATTGGAGGGCAAGTCTGGTGCCAGCAGCCGCGGTAATTCCAGCTCCAATAGC
ATATACTAATGTTGTTGCAGTTAAAACGCTCGTAGCTCAATTACTTTTGAGCTTATTACGGTTGTTCATTAGTTTGCTTA
AAGGCCTCACGGCTAATAGGTAAATTGATACGACCGGCTCAAACACTGTAGGTCATGGCATTAGGGAGCAATTTCTA
GTGTCATAATCGACTGTGCATAAACCTTGATGCTCAAGGTAGGCCTTTTATAGGGCAGATACACAGTGCATGGCATTG
TGGAACAAGGCATCTCGCGGCTTAGTTGGTGGGCCGCGGGGGCAATGATTAATAGGGAGGAGCGGGGGCCTTCATA
TTGCAGGGCGAGAGGTGAAATTCGTTGACCCTTGCAAGATGTCCGACAGCGAAAGCATTGGCCAAGTGCCTCTCCA
TTAGTCAAGAACGAAAGTTTGGGGATCAAAGACGATCAGATACCGTCGTAGTCCAAACCATAAACTATGTCGACCA
GCGATTAGGCGCGCTACCTTCTTCGAGAGCTGCCTAGCAGCTTGTGGGAAACCATGAGTGCTTGGACTCTGGGGGG
AGTATGGTCGCAAGTCTGAAACTTAAAGGAATTGACGGAAGGGCACACCATGGAGTGGAGCCTGCGGCTTAATTTG
ACTCAACACGGGAAAGCTTACCAAGCTCAGATATGATAAGGATTGACAGACTAAAAGATCTTTCATGATCTCATAAG
TGGTGGTGCATGGTCGTTCTTAGTTGGTGGAGTGATTTGTCAGGTCAATTCCGGTAACGGACGAGACCTCGACCTGC
TAACTAGTGGGGTTCATTCTTTCGCTTGACGAGGCAGGTTTTGCCTTTGATTACAGAAGGGGGGCAACTTTCTTTTG
TAGTTGGGGTAGAGTTTGTAGTAGTCTGGAGGAGTGGACTTCAAAAATTAAAAATAATCTTCCTAGAGGTACTTCTG
GCTCTAAGTCAGAGGAAGTCCGAGGCAATAACAGGTCTGTGATGCCCTTAAATATCTTGGGCCGCACGCGTGCTACA
ATGTAGGCGCTAGAAAGTCATAATTAAAACCATCTCCGCAAGGAGTATGGTAATCTTATAATCACCTGCGTGATTGGG
CTTGACCACTGTAACTGTGGTCATCAACGAGGAATTCCTTGTATGCGCGAGTCACTATCTCGCGCAGAATCTGTCCCT
GCCCTTTGTACACACCGCCCGTCGCTCCTACCGATCGAACGATCAGGTAAAGTGGACAGACACGAAGCTCGCAAGA
GCTTTTGGAAGTCCATTGAACCTCGCCGTTTAGAGGAAGGAGAAGTCGTAACAAGGTATCGGTAGGTGAACCTGCA
GAAGGATCAA

>HQ141520.1 *Cavenderia boomerangispora* K26B
AACCTGGTTGATCCTGCCAGTAGTCATATGCTTGTCTCAAGGATTAAGCCATGCATGTCTAAGTATAAGCCCTTGTAC
GGCTAGACTGCAGACGGCTCATTACAACGGTTGTAGCTTACAGAGCATCCGGGCGCAAGCCTTCTGGATAACCGCA
GTAAATCGGGGCTAATACATACAAACGGAGGGGTGGAGCGGGCAACCGCAAAGCTTCTGCGATGGACACTTAGCTA
TTCGACTAACCCTACGGGTCAGGTTGGAACCGGTTCATATTGCCAATCGACTATGGCTTGCCATAGTCTGATAAGCTC
TATAGACAACCGCCCTATCAACTTGATGGTAAGGTATTGGCTTACCATGGTTGTAACGGGTAACGGGGAATCAGGGT
TCGATTCCGGAGAGGACGCCTGAGAAACGGCGTCCACATCTACGGGTGGCAGCAGGCGCGTAAATTGCCCAATCTC
AATAGAGAGGAGGCGGCGACAATAAATCCCGATGGCTATGGGGGCAACCCCGGGCCAATCAGAATAAGTACACAAT
AAATACCCTAACCAATATAATTGGAGGGCAAGTCTGGTGCCAGCAGCCGCGGTAATTCCAGCTCCAATAGCATATACT
AACGTTGTTGCAGTTAAAACGCTCGTAGCTGAACAACTTGATGGCAAATTCAGGGCAGTGTCAAGAGTACTGGGGT
CAAACCTTGGATACATCTTGCCTGTCCGGCCAAGCAAGTAGGTAGTTTGGAGTAGGGGCAACTCTGCTTCAGGCGAT
CGACTGTGCATAAACCTTGATGCTCAAGGTAAAGACGACTCTAGTCAAAGATACGCAGTGCATGGCATTGTGGAAC
AAGGCGATCCGTAGCTTAGTTAGTGGGCTGCGGGTGCAATGATTAATAGGGAGGAGCGGGGGCCTTCATATTGCCGG
GCGAGAGGTGAAATTCGTTGACCCTGGCAAGATGTCCTACAGCGAAAGCATTGGCCAAGTGCCTCTCCATTAGTCA
AGAACGAAAGTTTGGGGATCAAAGACGATCAGATACCGTCGTAGTCCAAACCATAAACGATGTCGACCAGCGATTA
GGTTCGCCACCTTCTTCGAGAGCAACCTGGCAGCTTGTGGGAAACCATGAGTTCTTGGACTCTGGGGGGAGTATGG
TCGCAAGGCTGAAACTTAAAGGAATTGACGGAAGGGCACACCATGGAGTGGAGCCTGCGGCTTAATTTGACTCAAC
ACGGGAAAGCTTACCAAGCTCAGATATGATAAGGATTGACAGACCAATAGATCTTTCATGATCT

>MN338956.1 *Cavenderia canoespora* Mad14-3C
TATAAGCTCTTGTACGGCTAGACTGCAGACGGCTCATTACAACGGTTGTATCTTACAGGGCATCCGGGTCGCAAGAC
CTTCTGGATAACCGCAGTAAATCGGGGCTAATACATATAAACGGAGGGATGGAGAGGGCAACCTTGAAGTTTCTGCG
ATGGACATTTAGCTATTCGACCAACCCCGCAAGGGAACGGTTGGAACCGGTTCATATTGCTAATCGACTCTAGCTTG
CTAGTAGTCTGATGAGCTCTATAGACAACCGCCCTATCAACTTGATGGTAAGGTTTTGGCTTACCATGGTTGTAACGG
GTAACGGGGAATCAGGGTTCGATTCCGGAGAGGACGCCTGAGAAACGGCGTCCACATCTACGGGTGGCAGCAGGC
GCGTAAATTGCCCAATCTCAACAGAGAGGAGGCGGTGACAATAAATCCCGATGGCTTTGGGGGCAACCCCAGGCCA
ATCAGAATAAGTACACATTAAATCCCTTAACCAATATAATTGGAGGGCAAGTCTGGTGCCAGCAGCCGCGGTAATTCC
AGCTCCAATAGCATATACTAATGTTGTTGCAGTTAAAACGCTCGTAGCTGAATATCTTTTGAGCTACTATCGACTACAT
TCCTTCAAAGGTCGCAAGACTGATGAAGATGTGGTCGGCTCAAACACTGTAGGTGATGATATTGGTAGCAATACTAA
TATCATAATCGACTGTGCATAAACCTTGATGCTCAAGGTAGGCCTTTATAGGGTAGATACACAGTGCATGGCATTGTG
GAACAAGGCATCTCGCGGCTTAGTTGGTGGGCCGCGGGGGCAATGATTAATAGGGAGGAGCGGGGGCCTTCATATT
GCAGGGCGAGAGGTGAAATTCGTTGACCCTTGCAAGATGTCCGACAGCGAAAGCATTGGCCAAGTGCCTCTCCATT
AGTCAAGAACGAAAGTTTGGGGATCAAAGACGATCAGATACCGTCGTAGTCCAAACCATAAACTATGTCGACCAGC
GATTAGGCGGGCTACCTTCTTCGAGAGCTGCCTAGCAGCTTGTGGGAAACCATGAGTGCTTGGACTCTGGGGGGAG

TATGGTCGCAAGGCTGAAACTTAAAGGAATTGACGGAAGGGCACACCATGGAGTGGAGCCTGCGGCTTAATTTGAC
TCAACACGGGAAAGCTTACCAAGCTCAGATATGATAAGGATTGACAGACTAAAAGATCTTTCATGATCTCATAAGTG
GTGGTGCATGGTCGTTCTTAGTTGGTGGAGTGATTTGTCAGGTCAATTCCGGTAACGGACGAGACCTCGACCTGCTA
ACTAGTGGGATTCATTCTTTCGATTGACGAGGCGGGTATTGCTTTGATTATGAGGGGCAACCTTTATAGTCGGGTAAT
ATTTGTAGTAGTCTGGAGGAGTGGGTTTCAGACAATTTAATTAATCTTCCTAGAGGTACTTCTGGCTCTAAGCCAGAG
GAAGTCCGAGGCAATAACAGGTCTGTGATGCCCTTAGATATCTTGGGCCGCACGCGTGCTACAATGTAGGCGCTAAT
GAGTCGTAACAATTTCCAGCTCCGTAAGGAGTCTGGTAATCTTGGAATCACCTGCGTGATTGGGCTTGACTACTGTA
ACTGTGGTCATCAACGAGGAATTCCTTGTATGCGCGAGTCACTATCTCGCGCAGAATCTGTCCCTGCCCTTTGTACAC
ACCGCCCGTCGCTCCTACCGATCGAACGATCAGGTAAAGTGGACAGATTAGGAACCGCAAGGATCTTTAGAAGTCC
A

>AM168093.1 *Cavenderia delicata* TNS-C-226
AACCTGGTTGATCCTGCCAGTAGTCATATGCTTGTTTCAAGGATTAAGCCATGCATGTCTAAGTATAAGCTCTTGTAC
GGCTAGACTGCAGACGGCTCATTACAACGGTTGTATCTTACAGGGCATCCGGGTCGCAAGACCTTCTGGATAACCGC
AGTAAATCGGGGCTAATACATACAAACGGAGGGATGGAGAGGGTAACCTTGAAGTTTCTGCGATGGACATTTAGCTA
TTCGACCAACCCCGCAAGGGAATGGTTGGAACCGGTTCATATTGCTAATCGACTCTAGCTTGCTAGTAGTCTGATAA
GCCCTATAGACAACCGCCCTATCAACTTGATGGTAAGGTTTTGGCTTACCATGGTTGTAACGGGTAACGGGGAATCA
GGGTTCGATTCCGGAGAGGACGCCTGAGAAACGGCGTCCACATCTACGGGTGGCAGCAGGCGCGTAAATTGCCCA
ATCTCAACAGAGAGGAGGCGGTGACAATAAATCCCGATGGCTTTGGGGGCAACCCCAGGCTAATCAGAATAAGTAC
ACATTAAATCCCTTAACCAATATAATTGGAGGGCAAGTCTGGTGCCAGCAGCCGCGGTAATTCCAGCTCCAATAGCAT
ATACTAATGTTGTTGCAGTTAAAACGCTCGTAGCTTAATATCTTTTGAGCTACTATCGACTGTCATCCTTTTGTGGCGC
CTTCGGGTTAAGCAGGAGGACCGGTCGGCTCAAACACTGTAGGTCATGCGGCTAGCAATAGTTGCATAATCGACTGT
GCATAAACCTTGATGCTCAAGGTAGGCCTTTATAGGGTAGATACACAGTGCATGGCATTGTGGAACAAGGCATCTCG
CGGCTTAGTTGGTGGGCCGCGGGGGCAATGATTAATAGGGAGGAGCGGGGGCCTTCATATTGCAGGGCGAGAGGTG
AAATTCGTTGACCCTTGCAAGATGTCCTACAGCGAAAGCATTGGCCAAGTGCCTCCCCATTAGTTAAGAACGAAAGT
TTGGGGATCAAAGACGATCAGATACCGTCGTAGTCCAAACCATAAACTATGTCGACCAGCGATTAGGCGGGCTACCT
TCTTCGAGAGCTGCCTAGCAGCTTGTGGGAAACCATGAGTGCTTGGACTCTGGGGGGAGTATGGTCGCAAGGCTGA
AACTTAAAGGAATTGACGGAAGGGCACACCATGGAGTGGAGCCTGCGGCTTAATTTGACTCAACACGGGAAAGCTT
ACCAAGCTCAGATATGATTAGGATTGACAGACTAAAAGATCTTTCATGATCTCATAAGTGGTGGTGCATGGTCGTTCT
TAGTTGGTGGAGTGATTTGTCAGGTCAATTCCGGTAACGGACGAGACCTCGACCTGCTAACTAGTGGGATTCATTCT
TTCGATTGACGAGGCAGGTATTGCTTTGATTATAGGGGGCAACTTTTATAGTCGGGTAGTGCTTGTATTAGTCTGGGA
GAGTGGGTTTCAAAAATTTAATTAATCTTCCTAGAGGTACTTCTGGCTCTAAGCCAGAGGAAGTCCGAGGCAATAAC
AGGTCTGTGATGCCCTTAGATATCTTGGGCCGCACGCGTGCTACAATGTAGGCGCTAATGAGTTTATTTACATCCATCT
CCGCAAGGAGTATGGTAATCTTGGAATCCCCTGCGTGATTGGGCTTGACCACTGTAACTGTGGTCATCAACGAGGAA
TTCCTTGTATGCGCGAGTCACTATCTCGCGCAGAATCTGTCCCTGCCCTTTGTACACACCGCCCGTCGCTCCTACCGA
TCGAACGATCAGGTAAAGTGGACAGACTGAGACTCGCAAGAGTTTTTGGAAGTCCATTGAACCTCGCCGTTTAGAG
GAAGGAGAAGTCGTAACAAGGTATCGGTAGGTGAACCTGCAGAAGGATCAA

>AM168092.1 *Cavenderia deminutiva* MexM19A
AACCTGGTTGATCCTGCCAGTAGTCATATGCTTGTCTCAAGGATTAAGCCATGCATGTCTAAGTATAAGTCCTTGTAC
GACTAGACTGGCAGACGGCTCATTACAACAGTTGTTGTCTACAGGACATCCGCGCTTTTGCGTTTTGGATAACCGCAG
TAAATCGGGGCTAATACATATAAACGAAGGAATGACTGGTAACGGAAGTTTCTGCGATGGATTTAGCTATTCAACCG
GCCTTGTGTTTGTTGGAACCGATCCATATTGCTAATCGGCTATTAGTTTACTAATAGCCTGATGAGTTCTATGAACAAC
TGCCCTATCAACTTGATGGTAAGGTATTGGCTTACCATGGTTGTAACGGGTAACGGGGAATCAGGGTTCGATTCCGG
AGAGGACGCCTGAGAAACGGCGTCCACATCTACGGGTGGCAGCAGGCGCGTAAATTACCCAATCTCAATAGAGAGG
AGGTGGCGACAATAAATCCTGATGCCTATAGGCTCACGCCTAGGGCAATTAGAATAAGTACAAAATAAATCCATTA
ACCAATGTAATTGGAGGGCAAGTCTGGTGCCAGCAGCCGCGGTAATTCCAGCTCCAATAGCGTATACTAATGTTGTT
GCAGTTAAAACGCTCGTAGCTTAAAAACACGGATCATTTTTTAGCTAAATCGATTGCCACCTGGAAACAGGTGAGGT
GATTTATTTGGCATGGCACGTGCTGGTTGGGTTTGTTGGTTCGCTCTAGCAAGCTTAGTCAACTGTGCATAAACCTTG
ATGCTCAAGGTAAAGACCGTAGGTCTGTGATACTAAGTGCATGGTATTGTAGAACATGGTAACAGGTGGCTTTGTTG
GTTGGGTCACTAGTGCAATGATTAATAGGGAGGAGCGGGGGCCTTCATATTGATGGGCGAGAGGTGAAATTCGTTGA
CCCTATCAAGATGTCCTACAGCGAAAGCATTGGCCAAGTGCCTCTCCATTAGTCAAGAACGATAGTTTGGGGATCAA
AGACGATCAGATACCGTCGTAGTCCAAACCATAAACGATGTCGACCAGTGATTAGGCACGTCACCTTTCTAGAGAAC
TGCCTAGGAACTTAGTGGGAAACCATTGAGTTTTTGGACTCTGGGGGGAGTATGGTCGCAAGGCTGAAACTTAAAG
GAATTGACGGAAGGGCACACCATGGAGTGGAGCCTGCGGCTTAATTTGACTCAACACGGGAAAACTTACCAAGCTC
TGATATGATAAGGATTGACAGACTAAAAGATCTTTCATGATCGCATAAGTGGTGGTGCATGGTCGTTCTTAGTTGGTG
GAGTGATTTGTCAGGTCAATTCCGGTAACGGACGAGACCTCGACCTGCTAACTAGTGACAATTAGATTGTCGTATGG
GCGAAGTTAGTTCGGCAGCTACTTAATGGGCAACTGTTAGGTAGTTGACGTATTGATTGTAGCCTGGCAATGTAGTTG
TTATATTTTCCTTCTTAGAGGGACTTCCAGCTTTAAGTTGGAGGAAGTCCGAGGCAATAACAGGTCTGTGATGCCCTT
AGATATCTTGGGCCGCACGCGTGCTACAATGTAGGCGCTAATAGGCATTTACCCTTTACCTAGAGGTAGAGGTAAC
CTACAATCCCCTACGTGATGGGGATTGACCACTGTAACTGTGGTCATCAACGAGGAATTCCTTGTAGGCGTGCGTCA
CTATCGCACGCCGAATCTGTCCCTGCCCTCTGTACACACCGCCCGTCGCTCCTACCGATCGAACGTCCAGGTAAAGT
CGACGGACTGTCTTCGAAAGTTGATAGAAAGTCCATTGAACCTCGTCGTTTAGAGGAAGGAGAAGTCGTAACAAGG
TATCGGTAGGTGAACCTGCAGAAGGATCAA

>AM168085.1 *Cavenderia exigua* TNS-C-199
AACCTGGTTGATCCTGCCAGTAGTCATATGCTTGTCTCAAGGATTAAGCCATGCATGTCTAAGTATAAGCCCTTGTAC
GGCTAGACTGCAGACGGCTCATTACAACGGTTGTAGCTTACAGAGCATCCGGGCGCAAGCCTTCTGGATAACCGCA
GTAAATCGGGGCTAATACATACAAACGGAGGGGTGGAGCGGGCAACCGCAAAGCTTCTGCGATGGACACTTAGCTA
TTCGACTAACCCTTCGGGTCAGGTTGGAACCGGTTCATATTGCCAATCGACTATGGCTTGCCATAGTCTGATAAGCTC
TATAGACAACCGCCCTATCAACTTGATGGTAAGGTATTGGCTTACCATGGTTGTAACGGGTAACGGGGAATCAGGGT
TCGATTCCGGAGAGGACGCCTGAGAAACGGCGTCCACATCTACGGGTGGCAGCAGGCGCGTAAATTGCCCAATCTC
AATAGAGAGGAGGCGGCGACAATAAATCCCGATGGCTATGGGGGCAACCCCGGGCCAATCAGAATAAGTACACAAT
AAATACCCTAACCAATATAATTGGAGGGCAGGTCTGGTGCCAGCAGCCGCGGTAATTCCAGCTCCAATAGCATATACT
AACGTTGTTGCAGTTAAAACGCTCGTAGCTGAACAACTTGATGGCAAATTCAGGGCTTGTCACGAGTACCGCCTTAA

AACGGATACATCGTGCTTGTCCGGCCAAGCAAGTAGGTAGTTTGATGTGAGGCAACTCGCGTCAGGCGATCGACTG
TGCATAAACCTTGATGCTTAAGGTAAAGACGATTCTAGTCAAAGATACGCAGTGCATGGCATTGTGGAACAAGGCGA
TCCGTAGCTTAGTTAGTGGGCTGCGGGTGCAATGATTAATAGGGAGGAGCGGGGGCCTTCATATTGCCGGGCGAGAG
GTGAAATTCGTTGACCCTGGCAAGATGTCCTACAGCGAAAGCATTGGCCAAGTGCCTCTCCATTAGTCAAGAACGA
AAGTTTGGGGATCAAAGACGATCAGATACCGTCGTAGTCCAGACCATAAACGATGTCGACCAGCGATTAGGTTCGCC
ACCTTCTTCGAGAGCAACCTGGCAGCTTGTGGGAAACCATGAGTTCTTGGACTCTGGGGGGAGTATGGTCGCAAGG
CTGAAACTTAAAGGAATTGACGGAAGGGCACACCATGGAGTGGAGCCTGCGGCTTAATTTGACTCAACACGGGAA
AGCTTACCAAGCTCAGATATGATAAGGATTGACAGACCAATAGATCTTTCATGATCTCATAAGTGGTGGTGCATGGCC
GTTCTTAGTTGGTGGAGTGATTTGTCAGGTCAATTCCGGTAACGGACGAGACCTCGACCTGCTAACTAGTGGCAGT
TGTCTTTTCGCTCGGCGAGGCGTCATTGGCATACGACTTTGGTAGGGGCAACTCTATCTTGGAAGTGTGACAGCGGC
GTAGTAGTCTGGAGAGGCAGCTGTCCCCAATAATTAATCTTCCTAGAGGTACTTCCAGCTCTAAGCTGGAGGAAGTC
CGAGGCAATAACAGGTCTGTGATGCCCTTAGATATCTTGGGCCGCACGCGTGCTACAATGTAGGCGCTAATATGATAT
CCCAATTACCCGGCACCGTCAAGGTGTCGGTAATCTCCAATCACCTGCGTGATTGGGCTTGGTCACTGTAACTGTGA
CCATTAACGAGGAATTCCTTGTATACGCGAGTCACTATCTCGCATAGAATCTGTCCCTGCCCTTTGTACACACCGCCC
GTCGCTCCTACCGATCGAACGGTAAGGTAAAGTGGACAGACCGCCGCCCTCATCGGTAGCTGGAAGTCCATTGAAC
CTCTTCGTTTAGAGGAAGGAGAAGTCGTAACAAGGTATCGGTAGGTGAACCTGCAGAAGGATCATTT

>AM168086.1 *Cavenderia fasciculata* SmokOW9A
AACCTGGTTGATCCTGCCAGTAGTCATATGCTTGTTTCAAGGATTAAGCCATGCATGTCTAAGTATAAGCTCTTGTAC
GGCTAGACTGCAGACGGCTCATTACAACGGTTGTATCTTACAGGGCATCCGGGTCGCAAGACCTTCTGGATAACCGC
AGTAAATCGGGGCTAATACATACAAACGGAGGGATGGAGAGGGCAACCTTGAAGTTTCTGCGATGGACATTTAGCTA
TTCGACCAACCCCGCAAGGGAATGGTTGGAACCGGTTCATATTGCTAATCGACTCTAGCTTGCTAGTAGTCTGATAA
GCCCTATAGACAACCGCCCTATCAACTTGATGGTAAGGTTTTGGCTTACCATGGTTGTAACGGGTAACGGGGAATCA
GGGTTCGATTCCGGAGAGGACGCCTGAGAAACGGCGTCCACATCTACGGGTGGCAGCAGGCGCGTAAATTGCCCA
ATCTCAACAGAGAGGAGGCGGTGACAATAAATCCCGATGGCTTTGGGGGCAACCCCAGGCTAATCAGAATAAGTAC
ACATTAAATCCCTTAACCAATATAATTGGAGGGCAAGTCTGGTGCCAGCAGCCGCGGCAATTCCAGCTCCAATAGCA
TATACTAATGTTGTTGCAGTTAAAAACGCTCGTAGCTTAATATCTTTTGAGCTACTATCGACTGTCATCCTCTTATGTAG
CCGCAAGGTGAAGTAGGAGGGCCAGTCGGCTCAAACACTGTAGGTCATGCGGCTAGCAATAGTTGCATAATCGACT
GTGCATAAACCTTGATGCTCAAGGTAGGCCTTTATAGGGTAGATACACAGTGCATGGCATTGTGGAACAAGGCATCT
CGCGGCTTAGTTGGTGGGCCGCGGGGGCAATGATTAATAGGGAGGAGCGGGGGCCTTCATATTGCAGGGCGAGAGG
TGAAATTCGTTGACCCTTGCAAGATGTCCTACAGCGAAAGCATTGGCCAAGTGCCTCCCCATTAGTCAAGAACGAA
AGTTTGGGGATCAAAGACGATCAGATACCGTCGTAGTCCAAACCATAAACTATGTCGACCAGCGATTAGGCGGGCTA
CCTTCTTCGAGAGCTGCCTAGCAGCTTGTGGGAAACCATGAGTGCTTGGACTCTGGGGGGAGTATGGTCGCAAGGC
TGAAACTTAAAGGAATTGACGGAAGGGCACACCATGGAGTGGAGCCTGCGGCTTAATTTTGACTCAACACGGGAA
AAGCTTACCAAGCTCAGATATGATTAGGATTGACAGAGCTAAAAGATCTTTCATGATCTCATAAGTGGTGGTGCATGG
TCGTTCTTAGGTTGGTGGAGTGATTTGTCAGGTCAATTCCGGTAACGGACGAGACCTCGACCTGCTAACTAGTGG
GATTCATTCTTTCGATTGACGAGGCAGGTATTGCTTTGATTATAGGGGGCAACTTTTATAGTCGGGTAGTGCTTGTATT
AGTCTGGGAGAGTGGGTTTCAAAAATTTAATTAATCTTCCTAGAGGTACTTCTGGCTCTAAGCCAGAGGAAGTCCGA
GGCAATAACAGGTCTGTGATGCCCTTAGATATCTTGGGCCGCACGCGTGCTACAATGTAGGCGCTAATGAGTTTATTT
ACATCCATCTCCGCAAGGAGTATGGTAATCTTGGAATCCCCTGCGTGATTGGGCTTGACCACTGTAACTGTGGTCATC
AACGAGGAATTCCTTGTATGCGCGAGTCACTATCTCGCGCAGAATCTGTCCCTGCCCTTTGTACACACCGCCCGTCG
CTCCTACCGATCGAACGATCGGTAAAGTGGACAGACTGAAACCCGCAAGGGTTTTTGGAAGTCCTATTGAACCTCG
CCGTTTAGAGGAAGG

>AM168087.1 *Cavenderia fasciculata* SH3
AACCTGGTTGATCCTGCCAGTAGTCATATGCTTGTTTCAAGGATTAAGCCATGCATGTCTAAGTATAAGCTCTTGTAC
GGCTAGACTGCAGACGGCTCATTACAACGGTTGTATCTTACAGGGCATCCGGGTCGCAAGACCTTCTGGATAACCGC
AGTAAATCGGGGCTAATACATATAAACGGAGGGATGGAGAGGGTAACCTTGAAGTTTCTGCGATGGACATTTAGCTA
TTCGACCAACCCCGCAAGGGAATGGTTGGAACCGGTTCATATTGCTAATCGACTCTAGCTTGCTAGTAGTCTGATAA
GCCCTATAGACAACCGCCCTATCAACTTGATGGTAAGGTTTTGGCTTACCATGGTTGTAACGGGTAACGGGGAATCA
GGGTTCGATTCCGGAGAGGACGCCTGAGAAACGGCGTCCACATCTACGGGTGGCAGCAGGCGCGTAAATTGCCCA
ATCTCAACAGAGAGGAGGCGGTGACAATAAATCCCGATGGCTTTGGGGGCAACCCCAGGCTAATCAGAATAAGTAC
ACATTAAATCCCTTAACCAATATAATTGGAGGGCAAGTCTGGTGCCAGCAGCCGCGGTAATTCCAGCTCCAATAGCAT
ATACTAATGTTGTTGCAGTTAAAACGCTCGTAGCTTAATATCTTTTGAGCTACTATCGACTGTCATCCTCTCTTGATCC
GTTAAAAGTGAGAGGGAGGACCAGTCGGCTCAAACACTGTAGGTCATGCGGCTAGCAATAGTTGCATAATCGACTG
TGCATAAACCTTGATGCTCAAGGTAGGCCTTTATAGGGTAGATACACAGTGCATGGCATTGTGGAACAAGGCATCTC
GCGGCTTAGTTGGTGGGCCGCGGGGGCAATGATTAATAGGGAGGAGCGGGGGCCTTCATATTGCAGGGCGAGAGGT
GAAATTCGTTGACCCTTGCAAGATGTCCTACAGCGAAAGCATTGGCCAAGTGCCTCCCCATTAGTCAAGAACGAAA
GTTTGGGGATCAAAGACGATCAGATACCGTCGTAGTCCAAACCATAAACTATGTCGACCAGCGATTAGGCGGGCTAC
CTTCTTCGAGAGCTGCCTAGCAGCTTGTGGGAAACCATGAGTGCTTGGACTCTGGGGGGAGTATGGTCGCAAGGCT
GAAACTTAAAGGAATTGACGGAAGGGCACACCATGGAGTGGAGCCTGCGGCTTAATTTGACTCAACACGGGAAAG
CTTACCAAGCTCAGATATGATTAGGATTGACAGACTAAAAGATCTTTCATGATCTCATAAGTGGTGGTGCATGGTCGT
TCTTAGTTGGTGGAGTGATTTGTCAGGTCAATTCCGGTAACGGACGAGACCTCGACCTGCTAACTAGTGGGATTC
ATTCTTTCGATTGACGAGGCAGGTATTGCTTTGATTATAGGGGGCAACTTTTATAGTCGGGTAGTGCTTGTATTAGTCT
GGGAGAGTGGGTTTCAAAAATTTAATTAATCTTCCTAGAGGTACTTCTGGCTCTAAGCCAGAGGAAGTCCGAGGCA
ATAACAGGTCTGTGATGCCCTTAGATATCTTGGGCCGCACGCGTGCTACAATGTAGGCGCTAATGAGTTTATTTACAT
CCATCTCCGCAAGGAGTATGGTAATCTTGGAATCCCCTGCGTGATTGGGCTTGACCACTGTAACTGTGGTCATCAAC
GAGGAATTCCTTGTATGCGCGAGTCACTATCTCGCGCAGAATCTGTCCCTGCCCTTTGTACACACCGCCCGTCGCTCC
TACCGATCGAACGATCAGGTAAAGTGGACAGACTTTGGCCCGCAAGGGTTGTTGGAAGTCCATTGAACCTCGCCGT
TTAGAGGAAGGAGAAGTCGTAACAAGGTATCGGTAGGTGAACCTGCAGAAGGATCAA

>GQ496157.1 *Cavenderia fasciculoidea*
TTAGGATTAGCCATGCATGTCTAAGTATAAGCTCTTGTACGGCTAGACTGCAGACGGCTCATTACAACGGTTGTATCT
TACAGGGCATCCGGGTCGCAAGACCTTCTGGATAACCGCAGTAAATCGGGGCTAATACATATAAACGGAGGGATGGA
GAGGGTAACCTTGAAGTTTCTGCGATGGACATTTAGCTATTCGACCAACCCCGCAAGGGAATGGTTGGAACCGGTTC

ATATTGCTAATCGACTCTAGCTTGCTAGTAGTCTGATAAGCCCTATAGACAACCGCCCTATCAACTTGATGGTAAGGTT
TTGGCTTACCATGGTTGTAACGGGTAACGGGGAATCAGGGTTCGATTCCGGAGAGGACGCCTGAGAAACGGCGTCC
ACATCTACGGGTGGCAGCAGGCGCGTAAATTGCCCAATCTCAACAGAGAGGAGGCGGTGACAATAAATCCCGATGG
CTTTGGGGGCAACCCCAGGCTAATCAGAATAAGTACACATTAAATCCCTTAACCAATATAATTGGAGGGCAAGTCTG
GTGCCAGCAGCCGCGGTAATTCCAGCTCCAATAGCATATACTAATGTTGTTGCAGTTAAAACGCTCGTAGCTTAATAT
CTTTTGAGCTACTATCGACTGTCATCCTCTCTTGATCCGTTAAAAGTGAGAGGGAGGACCAGTCGGCTCAAACACTG
TAGGTCATGCGGCTAGCAATAGTTGCATAATCGACTGTGCATAAACCTTGATGCTCAAGGTAGGCCTTTATAGGGTAG
ATACACAGTGCATGGCATTGTGGAACAAGGCATCTCGCGGCTTAGTTGGTGGGCCGCGGGGGCAATGATTAATAGGG
AGGAGCGGGGGCCTTCATATTGCAGGGCGAGAGGTGAAATTCGTTGACCCTTGCAAGATGTCCTACAGCGAAAGCA
TTGGCCAAGTGCCTCCCCATTAGTCAAGAACGAAAGTTTGGGGATCAAAGACGATCAGATACCGTCGTAGTCCAAA
CCATAAACTATGTCGACCAGCGATTAGGCGGGCTACCTTCTTCGAGAGCTGCCTAGCAGCTTGTGGGAAACCATGAG
TGCTTGGACTCTGGGGGGAGTATGGTCGCAAGGCTGAAACTTAAAGGAATTGACGGAAGGGCACACCATGGAGTG
GAGCCTGCGGCTTAATTTGACTCAACACGGGAAAGCTTACCAAGCTCAGATATGATTAGGATTGACAGACTAAAAG
ATCTTTCATGATCTCATAAGTGGTGGTGCATGGTCGTTCTTAGTTGGTGGAGTGATTTGTCAGGTCAATTCCGGTAAC
GGACGAGACCTCGACCTGCTAACTAGTGGGATTCATTCTTTCGATTGACGAGGCAGGTATTGCTTTGATTATAGGGG
GCAACTTTTATAGTCGGGTAGTGCTTGTATTAGTCTGGGAGAGTGGGTTTCAAAAATTTAATTAATCTTCCTAGAGGT
ACTTCTGGCTCTAAGCCAGAGGAAGTCCGAGGCAATAACAGGTCTGTGATGCCCTTAGATATCTTGGGCCGCACGCG
TGCTACAATGTAGGCGCTAATGAGTTTATTTACATCCATCTCCGCAAGGAGTATGGTAATCTTGGAATCCCCTGCGTG
ATTGGGCTTGACCACTGTAACTGTGGTCATCAACGAGGAATTCCTTGTATGCGCGAGTCACTATCTCGCGCAGAATC
TGTCCCTGCCCTTTGTACACACCGCCCGTCGCTCCTACCGATCGAACGATCAGGTAAAGTGGACAGACTTTGGCCCG
CAAGGGTTGTTGGAAGTCCATTGAACCTCGCCGTTAGAGGAAGGAGAAGT

>MH762953.1 *Cavenderia fulva* Krug6-5A
AAGCTCTTGTACGGCTAGACTGCAGACGGCTCATTACAACGGTTGTATCTTCCAGGACATCCGGGTCGCAAGACCTT
CTGGATAACCGCAGTAAATCGGGGCTAATACATATAAACGGAGGGGTAGAGAGGGTAACCTTGAAGCTTCTGCGATG
GACATTTAGCTATTCGACCAACCCCGCAAGGGAATGGTTGGAACCGGTTCATATTGCTAATCGACTCTAGCTTGCTAG
TAGTCTGATAAGTCCTATAGACAACCGCCCTATCAACTTGATGGTAAGGTTTTGGCTTACCATGGTTGTAACGGGTAA
CGGGGAATCAGGGTTCGATTCCGGAGAGGACGCCTGAGAAACGGCGTCCACATCTACGGGTGGCAGCAGGCGCGT
AAATTGCCCAATCTCAACAGAGAGGAGGCGGTGACAATAAATCCCGATGGCTTTGGGGGCAACCCCAGGCCAATCA
GAATAAGTACACATTAAATCCCTTAACCAATATAATTGGAGGGCAAGTCTGGTGCCAGCAGCCGCGGTAATTCCAGC
TCCAATAGCATATACTAATGTTGTTGCAGTTAAAACGCTCGTAGCTCAATATCTTTTGAGCGCTTTCGGCCGTTTTCTT
TCCCTGACGAAAGGATGGGGAAGTACGGTCGGCTCAAACACTGTAGGTCATGGCAAAGTTGGCAACAGCTTTGTCA
TAATCGACTGTGCATAAACCTTGATGCTCAAGGTAGGCCTTTTATAGGGTAGATACACAGTGCATGGCATTGTGGAAC
AAGGCATCTCGCGGCTTAGTTGGTGGGCCGCGGGGGCAATGATTAATAGGGAGGAGCGGGGGCCTTCATATTGCAG
GGCGAGAGGTGAAATTCGTTGACCCTTGCAAGATGTCCGACAGCGAAAGCATTGGCCAAGTGCCTCCCCATTAGTC
AAGAACGAAAGTTTGGGGATCAAAGACGATCAGATACCGTCGTAGTCCAAACCATAAACTATGTCGACCAGCGATT
AGGCGGGCTACCTTCTTCGAGAGCTGCCTAGCAGCTTGTGGGAAACCATGAGTGCTTGGACTCTGGGGGGAGTATG
GTCGCAAGGCTGAAACTTAAAGGAATTGACGGAAGGGCACACCATGGAGTGGAGCCTGCGGCTTAATTTGACTCAA
CACGGGAAAGCTTACCAAGCTCAGATATGATTAGGATTGACAGACTAAAAGATCTTTCATGATCTCATAAGTGGTGG
TGCATGGTCGTTCTTAGTTGGTGGAGTGATTTGTCAGGTCAATTCCGGTAACGGACGAGACCTCGACCTGCTAACTG
TGGGGTTCATTCTTTCGATTGACGAGGCAGGCTTTGCTTTGATTATAAGGGGCAACTTTTATAGTCGGGTAGAGTTTG
TATTAGTCTGGGAGAGTGGGCTTCAAAAATTAAAATTAATCTTCCTAGAGGTACTTCTGGCTCTAAGCCAGAGGAAG
TCCGAGGCAATAACAGGTCTGTGATGCCCTTAGATATCTTGGGCCGCACGCGTGCTACAATGTAGGCGCTAATGAGT
TTATTTACATCCATCTCCGCAAGGAGTATGGTAATCTTTGAATCACCTGCGTGATTGGGCTTGACCACTGTAACTGTG
GTCATCAACGAGGAATTCCTWGTATGCGCGAGTCACTATCTCGCGCAGAATCTGTCCCTGCCCTTTGTACACACCGC
CCGTCGCTCCTACCGATCGAACGATCAGGTAAAGTGGACAGACTGGAGCTCGCAAGAGTCCTGGGAAGT

>AM168072.1 *Cavenderia granulophora* CHII-4
AACCTGGTTGATCCTGCCAGTAGTCATATGCTTGTTTCAAGGATTAAGCCATGCATGTCTAAGTATAAGCTCTTGTAC
GGCTAGACTGCAGACGGCTCATTACAACGGTTGTATCTTCCAGGACATCCGGGTCGCAAGATCTTCTGGATAACCGC
AGTAAATCGGGGCTAATACATACAAACGAAGGGGCAGAGAGGGCAACCTTGAAGCTTCTGCGATGGACACTTAGCT
ATTCGACCAACCCCGCAAGGGAATGGTTGGAACCGGTTCATATTGCTAATCGACTCTGGTTTACCATGAGTCGATAA
GTCCTATAGACAACCGCCCTATCAACTTGATGGTAAGGTTTTGGCTTACCATGGTTGTAACGGGTAACGGGGAATCA
GGGTTCGATTCCGGAGAGGACGCCTGAGAAACGGCGTCCACATCTACGGGTGGCAGCAGGCGCGTAAATTGCCCA
ATCTCAACAGAGAGGAGGCGGTGACAATAAATCCCGATGGCTTTGGGGGCAACCCCAGGCCAATCAGAATAAGTAC
ACATTAAATCCCTTAACCAATATAATTGGAGGGCAAGTCTGGTGCCAGCAGCCGCGGTAATTCCAGCTCCAATAGCAT
ATACTAATGTTGTTGCAGTTAAAACGCTCGTAGCTTAATTTCTTTTGAGCTACTTACGGTTGTCACCCTGTGAAGTCTT
CGGACCTAGTAGGAACAGCCGGCTCAAACACTGTAGGTCATGGTGCTAGCAATAGCATCATAATCGACTGTGCATAA
ACCTTGATGCTCAAGGTAGGCCTTTTTAGGGTAGATACACAGTGCATGGCATTGTGGAACAAGGCATCTCGCGGCTT
AGTTGGTGGGCCGCGGGGGCAATGATTAATAGGGAGGAGCGGGGGCCTTCATATTGCAGGGCGAGAGGTGAAATTC
GTTGACCCTTGCAAGATGTCCGACAGCGAAAGCATTGGCCAAGTGCCTCCCCATTAGTCAAGAACGAAAGTTTGGG
GATCAAAGACGATCAGATACCGTCGTAGTCCAGACCATAAACTATGTCGACCAGCGATTAGGCGGGCTACCTTCTTC
GAGAGCTGCCTAGCAGCTTGTGGGAAACCATGAGTGCTTGGACTCTGGGGGGAGTATGGTCGCAAGGCTGAAACTT
AAAGGAATTGACGGAAGGGCACACCATGGAGTGGAGCCTGCGGCTTAATTTGACTCAACACGGGAAAGCTTACCA
AGCTCAGATATGATAAGGATTGACAGACTAAAAGATCTTTCATGATCTCATAAGTGGTGGTGCATGGTCGTTCTTAGT
TGGTGGAGTGATTTGTCAGGTCAATTCCGGTAACGGACGAGACCTCGACCTGCTAACTAGTGGGATTCATTCCTTCT
CTCGACGAGGCAGGTTCTGCTTTTATTGTAAGGGCAACTTTGCAGTAGGGTAGGATTTGTATTAGTCTGGGGGAGTG
GATTTCATAATTTAATTAATCTTCCTAGAGGTACTTCTGGCTCTAAGCCAGAGGAAGTTCGAGGCAATAACAGGTCTG
TGATGCCCTTAGATATCTTGGGCCGCACGCGTGCTACAATGTAGGCGCTAATGAGTCTTTTTACATCCAGCTCCGTAA
GGAGTCTGGTAATCTTGGAATCCCCTGCGTGATTGGGCTTGACTACTGTAACTGTGGTCATCAACGAGGAATTCCTT
GTATGCGCGAGTCACTATCTCGCGCAGAATCTGTCCCTGCCCTTTGTACACACCGCCCGTCGCTCCTACCGATCGAAC
GATCAGGTAAAGTGGACAGACTAGAGGCCGCAAGGCATTTGGAAAGTCCATTGAACCTCGCCGTTTAGAGGAAGG
AGAAGTCGTAACAAGGTATCGGTAGGTGAACCTGCAGAAGGATCAA

>OM677255.1 *Cavenderia helicoidea* Landolt TH19B

AGGGTTGTTTGTTTAGGATTAGCCATGCATGTCTAAGTATAAGCTCTTGTACGGCTAGACTGCAGACGGCTCATTACA
ACGGTTGTATCTTCCAGGGCATCCGGGTCGCAAGACCTTCTGGATAACCGCAGTAAATCGGGGCTAATACATACAAA
CGGAGGGATGGAGAGGGCAACCTTGAAGTTTCTGCGATGGACAATTAGTTATTCGACCAACCCCGCAAGGGAATGG
TTGGAACCGGTTCATATTGCTAATCGACTCTAGCTTGCTAGTAGTCTGATAAGCCCTATAGACAACCGCCCTATCAAC
TTGATGGTAAGGTTTTGGCTTACCATGGTTGTAACGGGTAACGGGGAATCAGGGTTCGATTCCGGAGAGGACGCCTG
AGAAACGGCGTCCACATCTACGGGTGGCAGCAGGCGCGTAAATTGCCCAATCTCAACAGAGAGGAGGCGGTGACA
ATAAATCCCGATGGCTTTGGGGGCAACCCCAGGCTAATCAGAATAAGTACACATTAAATCCCTTAACCAATATAATTG
GAGGGCAAGTCTGGTGCCAGCAGCCGCGGTAATTCCAGCTCCAATAGCATATACTAATGTTGTTGCAGTTAAAACGC
TCGTAGCTTAATATCTTTTGAGCTACTATCGATTGTTATCCTGCTGACCCGAAAGGTGATGTAGGAGGCAGTCGGCTC
AAACACTGTAGGTCATGCGGCTAGCAATAGTTGCATAATCGACTGTGCATAAACCTTGATGCTCAAGGTAGGCCTTTA
TAGGGTAGATACACAGTGCATGGCATTGTGGAACAAGGCATCTCGCGGCTTAGTTGGTGGGCCGCGGGGGCAATGA
TTAATAGGGAGGAGCGGGGGCCTTCATATTGCAGGGCGAGAGGTGAAATTCGTTGACCCTTGCAAGATGTCCTACA
GCGAAAGCATTGGCCAAGTGCCTCCCCATTAGTCAAGAACGAAAGTTTGGGGATCAAAGACGATCAGATACCGTCG
TAGTCCAAACCATAAACTATGTCGACCAGCGATTAGGCGGGCTACCTTCTTCGAGAGCTGCCTAGCAGCTTGTGGGA
AACCATGAGTGCTTGGACTCTGGGGGGAGTATGGTCGCAAGGCTGAAACTAAAAGGAATTGACGGAAGGGCACAC
CATGGAGTGGAGCCTGCGGCTTAATTTGACTCAACACGGGAAAGCTTACCAAGCTCAGATATGATTAGGATTGACAG
ACTAAAAGATCTTTCATGATCTCATAAGTGGTGGTGCATGGTCGTTCTTAGTTGGTGGAGTGATTTGTCAGGTCAATT
CCGGTAACGGACGAGACCTCGACCTGCTAACTAGTGGGATTCATTCTTTCGATTGACGAGGCAGGTCTTGCTTTGAT
TATAGGGGGCAACTTCTATAGTCGGGTACGGTTTGTAGTAGTCTGGAGGAGTGGATTTCAAAAATTAAATTAATCTTC
CTAGAGGTACTTCTGGCTCTAAGCCAGAGGAAGTCCGAGGCAATAACAGGTCTGTGATGCCCTTAGATATCTTGGGC
CGCACGCGTGCTACAATGTAGGCGCTAATGAGTTTATTTACATCCATCTCCGCAAGGAGTATGGTAATCTTGGAATCA
CCTGCGTGATTGGGCTTGACCACTGTAACTGTGGTCATCAACGAGGAATTCCTTGTATGCGCGAGTCACTATCTCGC
GCAGAATCTGTCCCTGCCCTTTGTACACACCGCCCGTCGCTCCTACCGATCGAACGATCAGGTAAAGTGGACAGACT
GGAGCCCGCAAGGGTTCTTGGAAGTCCATTGAACCTCGCCGTTAGAGAAGGAGAAGTCGAAACAATTTG

>HQ141519.1 *Cavenderia macrocarpa* MGE2
AACCTGGTTGATCCTGCCAGTAGTCATATGCTTGTCTCAAGGATTAAGCCATGCATGTCTAAGTATAAGCCCTTGTAC
GGCTAGACTGCAGACGGCTCATTACAACGGTTGCATCTTCCAGAGCATCCGGGTCGCAAGACCTTCTGGATAACCGC
AGTAAATCGGGGCTAATACATACAAACGGAGGGGTGGACCAGGCAACTGGGAAACTTCTGCGATGGACACTTAGCT
ATTCGACTGACCCTTTCGGGGAATGGTTGGAACCGGTTCATATTGCTAATCGATTTGTGGCTTGCCACAAGTCTGATA
AGCTCTATAGGCAACCGCCCTATCAACTTGATGGTAAGGTTTTGGCTTACCATGGTTGTAACGGGTAACGGGGAATC
AGGGTTCGATTCCGGAGAGGACGCCTGAGAAACGGCGTCCACATCTACGGGTGGCAGCAGGCGCGTAAATTGCCC
AATCTCAATAGAGAGGAGGCGGTGACAATAAATCCCAATGACCATGGGGGCAACCCCGGGTCAATTAGAATAAGTA
CACATTAAATACCCTAACCCAATATAATTGGAGGGCAAGTCTGGTGCCAGCAGCCGCGGTAATTCCAGCTCCAATAG
CGTATACTAATGTTGTTGCAGTTAAAACGCTCGTAGCTAAAGATCTTGATGGCAAATTCTTGTGAAGACTCCATAGTC
CTCTGGGGAAACCCATTGTGACTTTTGGACTTTACAGGCCAGCAAGTAGGTAGTCCTGGGCATGAGGGTAACCTTGT
GTTTAGCGACGATCGACTGTGCATAAACCTTGATGCTCAAGGTAAACAAAATCTTCTTGAAAGATACACGGTGCATG
GCATTGTGGAATATGGCGTCTTTGTGTCCTTGTTGGTTGGGACACAAGAGCAATGATTAATAGGGAGGAGCGGGGGC
CTTCATATTGCCGGGCGAGAGGTGAAATTCGTTGACCCTGGCAAGATGTCCTACAGCGAAAGCATTGGCCAAGTGC
CTCTCCATTAGTCAAGAACGAAAGTTTGGGGATCAAAGACGATCAGATACCGTCGTAGTCCAAACCATAAACTATGT
CGACCAGCGATTAGGTTCGCCACCTTCTTCGAGAGCAACCTGGCAGCTTGTGGGAAACCATGAGTTCTTGGACTCT
GGGGGGAGTATGGTCGCAAGGCTGAAACTTAAAGGAATTGACGGAAGGGCACACCATGGAGTGGAGCCTGCGGCT
TAATTTGACTCAACACGGGAAAGCTTACCAAGCTCAGATATGATAAGGATTGACAGACCAAAAGATCTTTCATGATC
TCATAAGTGGTGGTGCATGGCCGTTCTTAGTTGGTGGAGTGATTTGTCAGGTCAATTCCGGTAACGGACGAGACCT
CGACCTGCTAACTAGTGGCAGTTGTCTTTTCGATCGGCGAGGCGTCGTGGGCATGGAACTTCTATAGGGGCAACTCT
ATGGCTTGGGAAGTGTCCACGATGTAGTAGTCTGGAGAGGCAGCTGTCCCCAAAAATATAATCTTCCTAGAGGTACT
TCCAGCTCTAAGTTGGAGGAAGTTCGAGGCAATAACAGGTCTGTGATGCCCTTAGATATCTTGGGCCGCACGCGTGC
TACAATGTAGGCGCTAATAAGAAAAACATTTTATCCCCGGCACCGCCAAGGTGTCCGGTAATCCACAATCACCTGCG
TGATTGGGATTGGTCACTGTAACTGTGACCATCAACGAGGAATTCCTTGTATGCGCGGGTCACTATCCCGCGCAGAA
TCTGTCCCTGCCCTTTGTACACACCGCCCGTCGCTCCTACCGATCGAACGGTAAGGTAAAGTGGACAGACAGTCCCT
CTTTATTGAGGAGCTGAAAGTCCATTGAACCTCGTCGTTAGAGGAAGGAGAAGTCGTAACAAGGTATCGGTAGGTG
AACCTGCAGAAGGATCA

>AM168088.1 *Cavenderia medusoides* OH592
AACCTGGTTGATCCTGCCAGTAGTCATATGCTTGTTTCAAGGATTAAGCCATGCATGTCTAAGTATAAGCTCTTGTAC
GGCTAGACTGCAGACGGCTCATTACAACGGTTGTATCTTCCAGAACGTCCGGGTCGCAAGATCTTCTGGATAACCGC
AGTAAATCGGGGCTAATACATACAAACGAAGGGGTGGAGAGGGCAACCTTGAAGCTTCTGCGATGGACACTTAGCT
ATTCGACCAACCCCGCAAGGGAATGGTTGGAACCGGTTCATATTGCTAATCGACTCTGGTTTACCATGAGTCGATAA
GTTCTATAGACAACCGCCCTATCAACTTGATGGTAAGGTTTTGGCTTACCATGGTTGTAACGGGTAACGGGGAATCA
GGGTTCGATTCCGGAGAGGACGCCTGAGAAACGGCGTCCACATCTACGGGTGGCAGCAGGCGCGTAAATTGCCCA
ATCTCAACAGAGAGGAGGCGGTGACAATAAATCCCGATGGCTTTGGGGGCAACCCCAGGCCAATCAGAATAAGTAC
ACATTAAATCCCTTAACCAATATAATTGGAGGGCAAGTCTGGTGCCAGCAGCCGCGGTAATTCCAGCTCCAATAGCAT
ATACTAATGTTGTTGCAGTTAAAACGCTCGTAGCTTAATTTCTTTTGAGCTACTTACGGTTGTCACCCTGTGAAGTCTT
CGGACCTAATAGGAACAGCCGGCTCAAACACTGTAGGTCATGGTACTGGTAACAGTATCATAATCGACTGTGCATAA
ACCTTGATGCTCAAGGTAGGCCTTTTTAGGGTAGATACACAGTGCATGGCATTGTGGAACAAGGCATCTCGCGGCTT
AGTTGGTGGGCCGCGGGGGCAATGATTAATAGGGAGGAGCGGGGGCCTTCATATTGCAGGGCGAGAGGTGAAATTC
GTTGACCCTTGCAAGATGTCCGACAGCGAAAGCATTGGCCAAGTGCCTCCCCATTAGTCAAGAACGAAAGTTTGGG
GATCAAAGACGATCAGATACCGTCGTAGTCCAAACCATAAACTATGTCGACCAGCGATTAGGCGGGCTACCTTCTTC
GAGAGCTGCCTAGCAGCTTGTGGGAAACCATGAGTGCTTGGACTCTGGGGGGAGTATGGTCGCAAGGCTGAAACTT
AAAGGAATTGACGGAAGGGCACACCATGGAGTGGAGCCTGCGGCTTAATTTGACTCAACACGGGAAAGCTTACCA
AGCTCAGATATGATAAGGATTGACAGACTAAAAGATCTTTCATGATCTCATAAGTGGTGGTGCATGGTCGTTCTTAGT
TGGTGGAGTGATTTGTCAGGTCAATTCCGGTAACGGACGAGACCTCGACCTGCTAACTAGTGGGATTCATTCCTTCG
CTCGACGAGGCAGGTTCTGCTTTTATTGTAGGGGCAACTTTACAGTAGGGTAGGATTTGTATTAGTCTGGGGGAGTG
GATTTCATAATTTAATTAATCTTCCTAAAGGTACTTCTGGCTCTAAGCCAGAGGAAGTTCGAGGCAATAACAGGTCTG
TGATGCCCTTAGATATCTTGGGCCGCACGCGTGCTACAATGTAGGCGCTAATGAGTCTTTTTACATCCAGCTCCGTAA

GGAGTCTGGTAATCTTGGAATCCCCTGCGTGATTGGGCTTGACTACTGTAACTGTGGTCATCAACGAGGAATTCCTT
GTATGCGCGAGTCACTATCTCGCGCAGAATCTGTCCCTGCCCTTTGTACACACCGCCCGTCGCTCCTACCGATCGAAC
GATCAGGTAAAGTGGACAGACTAGAAGCCGCAAGGTTTTTGGAAAGTCCATTGAACCTCGCCGTTTAGAGGAAGG
AGAAGTCGTAACAAGGTATCGGTAGGTGAACCTGCAGAAGGATCAA

>AM168089.1 *Cavenderia mexicana* MexTF4B1
AACCTGGTTGATCCTGCCAGTAGTCATATGCTTGTTTCAAGGATTAAGCCATGCATGTCTAAGTATAAGCTCTTGTAC
GGCTAGACTGCAGACGGCTCATTACAACGGTTGTATCTTCCAGGACATCCGGGTCGAAAGACCTTCTGGATAACCGC
AGTAAATCGGGGCTAATACATACAAACGGAGGGGTGGATAGGGCAACCTTGAAGCTTCTGCGATGGACACTTAGCT
ATTCGACCAGCCCCGCAAGGGAACGGTTGGAACCGGTTCATATTGCTAATCGACTCTGGCTTGCCACGAGTCTGATA
AGTCCTATAGACAACCGCCCTATCAACTTGATGGTAAGGTTTTGGCTTACCATGGTTGTAACGGGTAACGGGGAATC
AGGGTTCGATTCCGGAGAGGACGCCTGAGAAACGGCGTCCACATCTACGGGTGGCAGCAGGCGCGTAAATTGCCC
AATCTCAACAGAGAGGAGGCGGTGACAATAAATCCCGATGGCTTTGGGGGCAACCCCAGGCCAATCAGAATAAGTA
CACATTAAATCCCTTAACCAATATAATTGGAGGGCAAGTCTGGTGCCAGCAGCCGCGGTAATTCCAGCTCCAATAGC
ATATACTAATGTTGTCGCAGTTAAAAACGCCTCGTAGCTTAATTACTTTTTGAGCTACTTACGGTTGTCCAACTTTGCG
AAAGACTTAACTTTTCCTAGTGAAGAACAACCGGCTCAGACACTGTAGGTCGTGGCATTGGGGGTAACTCTAGTGT
CACTATCGACTGTGCATAAACCTTGATGCTCAAGGTAGGCCTTTATAGGGTAGATACACAGTGCATGGCATTGTGGAA
CAAGGCATCTCGCGGCTTAGTTGGTGGGCCGCGGGGGCAATGATTAATAGGGAGGAGCGGGGACCTTCATATTGCA
GGGCGAGAGGTGAAATTCGTTGACCCTTGCAAGATGACCGACAGCGAAAGCATTGGTCAAGTGCCTTCCCATTAGT
CAAGAACGAAAGTTTGGGGATCAAAGACGATCAGATACCGTCGTAGTCCAAACCATAAACTATGTCGACCAGCGAT
TAGGCGGGCTACCTTCTTCGAGAGCTGCCTAGCAGCTTGTGGGAAACCATGAGTGCTTGGACTCTGGGGGGAGTAT
GGTCGCAAGGCTGAAACTTAAAGGAATTGACGGAAGGGCACACCATGGAGTGGAGCCTGCGGCTTAATTTGACTCA
ACACGGGAAAGCTTACCAAGCTCAGATATGATAAGGATTGACAGACTAAAAGATCTTTCATGATCTCATAAGTGGTG
GTGCATGGTCGTTCTTAGTTGGTGGAGTGATTTGTCAGGTCAATTCCGGTAACGGACGAGACCTCGACCTGCTAACT
AGTGGGGTTCATTCCTTCGCTCGATGAGGCAAGGTCTGCTTTTATTGCAGGGGGTAACTCTTGTAGTAGGGTAGGCT
TTGTATTAGTCTGGGGGAGTGGACTTCCAATATAATTTATAATCTTCCTAGAGGTACTTCTGGCTCTAAGCCAGAGGA
AGTCCGAGGCAATAACAGGTCTGTGATGCCCTTAGATATCTTGGGCCGCACGCGTGCTACAATGTAGGCGCTAATGA
GTCGTAACATATCCAGCTCCGTAAGGAGTCTGGTAATCTTGGAATCCCCTGCGTGATTGGGCTTGACTACTGTAACTG
TGGTCATCAACGAGGAATTCCTTGTATGCGTGAGTCACTATCTCACGCAGAATCTGTCCCTGCCCTTTGTACACACCG
CCCGTCGCTCCTACCGATCGAACGATCAGGTAAAGTGGACAGACTTTAGCCCGCAAGGGTTTTGGGAAGTCCATTG
AACCTCGCCGTTTAGAGGAAGGAGAAGTCGTAACAAGGTATCGGTAGGTGAACCTGCAGAAGGTCAA

>AM168090.1 *Cavenderia microspora* TNS-C-38
AACCTGGTTGATCCTGCCAGTAGTCATATGCTTGTCTCAAGGATTAAGCCATGCATGTCTAAGTATAAGCCCTAGTAC
GGCTAGACTGCAGACGGCTCATTACAACGGTTGCAGCTTACAGAGCATCCGGGTCGCAAGACCTTCTGGATAACCG
CAGTAAATCGGGGCTAATACATACAAACGGAGGGGCGGAGCGGGCAACCGCGAAGCTTCTGCGATGGACACTTAGC
TATTCGACTGACCCCTCACGGGAATGGTTGGAACCGGTTCATATTGCTAATCGACTTGTGGCTTGCCTCAAGTCTGAT
AAGCTCTATAGGCAACCGCCCTATCAACTTGATGGTAAGGTATTGGCTTACCATGGTTGTAACGGGTAACGGGGAAT
CAGGGTTCGATTCCGGAGAGGACGCCTGAGAAACGGCGTCCACATCTACGGGTGGCAGCAGGCGCGTAAATTGCCC
AATCTCAATAGAGAGGAGGCGGTGACAATAAATCCCGATGGCCATGGGGGCAACCCCGGGCCAATCAGAATAAGTA
CACATTAAATACCCTAACCAATATAATTGGAGGGCAAGTCTGGTGCCAGCAGCCGCGGTAATTCCAGCTCCAATAGC
GTATACTAATGTTGTTGCAGTTAAAACGCTCGTAGCTGAACAACTTGATGGCAAATTCAGGTCAATGTGGCACGTCC
ATGGGGGGAAACCTTTATGCGGCAGTGTCCTTGACCGGCTAAGCAGGTAGGTAGTTTGGCGGTGTAGCAATACATCG
TCAGGCGATCGACTGTGCATAAACCTTGATGCTCAAGGTAAAGACGATTCTAGTCAAAGATACGCAGTGCATGGCAT
TGTGGAACAAGGCGATCCGTAGCTTGGTTGGTGAGCTACGGGTGCAATGATTAATAGGGAGGAGCGGGGGCCTTCA
TATTGCCGGGCGAGAGGTGAAATTCGTTGACCCTGGCAAGATGTCCTACAGCGAAAGCATTGGCCAAGTGCCTCTC
CATTAGTCAAGAACGAAAGTTTGGGGATCAAAGACGATCAGATACCGTCGTAGTCCAAACCATAAACTATGTCGACC
AGCGATTAGGTTCGCCACCTTCTTCGAGAGCAACCTGGCAGCTTGTGGGAAACCATGAGTTCTTGGACTCTGGGGG
GAGTATGGTCGCAAGGCTGAAACTTAAAGGAATTGACGGAAGGGCACACCATGGAGTGGAGCCTGCGGCTTAATTT
GACTCAACACGGGAAAGCTTACCAAGCTCAGATATGATAAGGATTGACAGACCAAAAGATCTTTCATGATCTCATAA
GTGGTGGTGCATGGCCGTTCTTAGTTGGTGGAGTGATTTGTCAGGTCAATTCCGGTAACGGACGAGACCTCGACCTG
CTAACTAGTGGCAGTTGTCTTTTCGCTCGGCGAGGCGTTTTGGGCGTGGAACTTTGGTAGGGGCAACTCTATCTTTG
GGAAGCGAACGAGGCGTAGTAGTCTGGAGAGGCAGCTGTCCCCATATTAAAATAATCTTCCTAGAGGTACTTCCAGC
TCTAAGTTGGAGGAAGTCCGAGGCAATAACAGGTCTGTGATGCCCTTAGATATCTTGGGCCGCACGCGTGCTACAAT
GTAGGCGCTAATGAGTCGTTCTTAATATCCGGCACCGCAAGGTGTCTGGTAATCCCCAATCCCCTGCGTGATTGGGAT
TGGTCACTGTAACTGTGACCATCAACGAGGAATTCCTTGTATGCGCGGGTCACTATCCCGCGCAGAATCTGTCCCTG
CCCTTTGTACACACCGCCCGTCGCTCCTACCGATCGAACGGTAAGGTAAAGTGGACGGACAGCTGTCCTCACGGAT
GGCTGGAAGTCCATTGAACCTGCGTCGTTTAGAGGAAGGAGAAGTCGTAACAAGGTATCGGTAGGTGAACCTGCAG
AAGGATCATTT

>MH762954.1 *Cavenderia minima* Eden1
ATAAGCCCTTGTACGGCTAGACTGCAGACGGCTCATTACAACGGTTGTAGCTTACAGAGCATCCGGGTCGCAAGACC
TTCTGGATAACCGCAGTAAATCGGGGCTAATACATACAAACGGAGGGGTGGAGCGGGCAACCGCAAAGCTTCTGCG
ATGGACACTTAGCTATTCGACTAACCCCTTCGGGTCAGGTTGGAACCGGTTCATATTGCCAATCGACTATGGCTTGCCA
TAGTCTGATAAGCTCTATAGACAACCGCCCTATCAACTTGATGGTAAGGTATTGGCTTACCATGGTTGTAACGGGTAA
CGGGGAATCAGGGTTCGATTCCGGAGAGGACGCCTGAGAAACGGCGTCCACATCTACGGGTGGCAGCAGGCGCGT
AAATTGCCCAATCTCAATAGAGAGGAGGCGGCGACAATAAATCCCGATGGCTATGGGGGCAACCCCGGGCCAATCA
GAATAAGTACACAATAAATACCCTAACCAATATAATTGGAGGGCAAGTCTGGTGCCAGCAGCCGCGGTAATTCCAGC
TCCAATAGCATATACTAACGTTGTTGCAGTTAAAACGCTCGTAGCTGAACAACTTGATGGCAAATTCAGGGCAGTGC
CACGAGTACTGAGGGTCAAACCTTGGCTACATCGTGCCTGTCCGGCCAAGCAGGTAGGTAGTCTGTGGTAGGGGTA
ACTCTGCCATAGGCGATCGACTGTGCATAAACCTTGATGCTCAAGGTAAAGACGACTCTAGTCAAAGATACGCAGTG
CATGGCATTGTGGAACAAGGCGATCCGTAGCTTAGTTAGTGGGCTGCGGGTGCAATGATTAATAGGGAGGAGCGGG
GGCCTTCATATTGCCGGGCGAGAGGTGAAATTCGTTGACCCTGGCAAGATGCACGACAGCGAAAGCATTGGCCAAG
TGCCTCTCCATTAGTCAAGAACGAAAGTTTGGGGATCAAAGACGATCAGATACCGTCGTAGTCCAAACCATAAACG
ATGTCGACCAGCGATTAGGTTCGCCACCTTCTTCGAGAGCAACCTGGCAGCTTGTGGGAAACCATGAGTTCTTGGA

CTCTGGGGGGAGTATGGTCGCAAGGCTGAAACTTAAAGGAATTGACGGAAGGGCACACCATGGAGTGGAGCCTGC
GGCTTAATTTGACTCAACACGGGAAAGCTTACCAAGCTCAGATATGATAAGGATTGACAGACCAATAGATCTTTCAT
GATCTCATAAGTGGTGGTGCATGGCCGTTCTTAGTTGGTGGAGTGATTTGTCAGGTCAATTCCGGTAACGGACGAGA
CCTCGACCTGCTAACTAGTGGCAGTTGTCTTTTCGATCGGCGAGGCGTCATTGGCATACGACCTTGGTAGGGGCAAC
TCTATCTTGGAAGTGTGACAGTGGCGTAGTAGTCTGGAGAGGCAGCTGTCCCCATATAAATAATCTTCCTAGAGGTAC
TTCCAGCTCTAAGCTGGAGGAAGTCCGAGGCAATAACAGGTCTGTGATGCCCTTAGATATCTTGGGCCGCACGCGTG
CTACAATGTAGGCGCTAATATGATATCCCAATTACCCGGCACCGTCAAGGTGTCGGTAATCTCCAATCACCTGCGTGA
TTGGGCTTGGTCACTGTAACTGTGACCATTAACGAGGAATTCCTTGTATACGCGAGTCACTATCTCGCATAGAATCTG
TCCCTGCCCTTTGTACACACCGCCCGTCGCTCCTACCGATCGAACGGTAAGGTAAAGTGGACAGACAGCCGCCCTC
ACCGGTAGCTGGAAGTCCAT

>AM168070.1 *Cavenderia multistipes* UK26b
AACCTGGTTGATCCTGCCAGTAGTCATATGCTTGTCTAAAGGATTAAGCCATGCATGTCTAAGTATAGTCCTCACGGA
CGAAACTGCAGACGGCTCATTACAACGGTCGCTGGTCACAGGACACGCGGCGGGGCAACTCGCTGTGGATAACCG
CATTAAATCGGGGCTAATACATACAAAACCGAGTGGACGACTGGGCAACTGGAAGTTCGCGCGATGGATTAACCCTT
CGACTGACCTCTTGGTTTGGTTGGAACCGATTCATGTTGTTAGTCGGCGCTTCGGCGCCGATTTGTTCTAGACGCGA
CTGCCCTATCAGCTTGACGGCAAGGTCTTGGCTTGCCGTGGCTGTAACGGGTAACGGAGAATTAGGGTTCGATTCCG
GAGAGGACGCCTGAGAAACGGCGTCCACACCTACGGGTGGCAGCAGGCACGTAAATTTCCCAATGTCAATACGATG
AGGGAGCGACAATAAATACTTTTAGTCCTGGTCGCAAGATCAGGACTCAACAAGATTAAGCACAAAGTAAATAAATT
AGCCAGTATTATTGGAGGGCAAGTCTGGTGCCAGCAGCCGCGGTAATTCCAGCTCCAACAGTATATACTAAAGTTGT
TGCAGTTAAAACGTTCGTAGCTTAAATACAGGTCTGTGTAATTGCTTGTCACGTCGTTCGCGGCGTGACAAGCGCAG
GCGCGGTAGATTGTCATGTCTAGTCGGCAACGGCTAGGCGCGGCGTTCAACTGTGCATAAATCTTGATGCTCAAGGT
GAGTCCAGGCGTGGACGGGAAAACAGTGCATGGTATTGTAGAAGAAGACGTGGCAACGTCTAGGTTGGTTGGTTGT
CCAGGTAATGATTAATAGGGAGGAGCGGGGGCTGTCGTATTGACGGGCGAGAGGTGAAATTCGTTGACCCTGTCAA
GACGCACAACGGCGGAAGCAGCAGCCAAGTGCCTCTCCATTAGTCAAGAACGATAGTCTAGGGATCAAAGACGATC
AGATACCGTCGTAGTCTAGACCATAAACTATGTCGGCTAACACTTGGCGACGTGCTCTCCGAGGCGTCTCCAGGAGT
TTGTGGGAAACCATAAGCTTTTGGACTCTGGGGGGAGTATGGCCGCAAGACTGAAACTTAAAGGAATTGACGGAAG
GGCACACCATGGCGTGGAGCCTGCGGCTTAATTTGACTCAACACGGGAAACCTTACCAAGCTCAGATATGTATAGGA
TTGACAGACTAATAGATTTTTCATGATCGCATAAGTAGTGGTGCATGGCCGTTCTTAGTTGGTGGAGTGATTTGTCAG
GTCAATTCCGGTAACGGACGAGACCTCCATTTGCTAACTAGTCGCGGCGACGGGTTCGACTGGCGAGGGTCGGACG
GCGCGGCTGCCTGGTCGAAAGGCTGGGTAAGTGCGTCGTACGGTTAGTAGCCTGGGCTCGTCTTCGCGGAATAAAT
AAAGTAACTTCTTAGAAATACTTCCGACACCAAGCCGGAGGAAGTTGGAGGCAATAACAGGTCTGTGATGCCCTTA
GATATCTTGGGCCGCACGCGTGCTACAATGTAGACGCGAATAAGCTCTAAAAATGCGCGGCGCGAAAGCGTCGCGG
AAACAGCAATCGTCTACGTAACAGGGACTGACGGTTGCAATAACGATCACGAACGAGGAATGCCTTGTAGGCGCGA
GTCATCATCTCGCGCCGAATCTGTCCCTGCCCTTTGTACACACCGCCCGTCGCTCCTACCGATCGGACGGCGAGGTG
AAGACGACGGACTGTATCCTTCACGGGACGCGGAAAGTCGTTTAAACCTTGTTGTCTAGAGGAAGGAGAAGTCGTA
ACAAGGTATCGGTAGGTGAACCTGCAGAAGGATCAA

>HQ141522.1 *Cavenderia myxobasis* NT2A
AACCTGGTTGATCCTGCCAGTAGTCATATGCTTGTCTCAAGGATTAAGCCATGCATGTCTAAGTATAAGCTCTTGTAC
GGCTAGACTGCAGACGGCTCATTACAACGGTTGTATCTTCCAGGGCATCCGGGTCGCAAGACCTTCTGGATAACCGC
AGTAAATCGGGGCTAATACATACAAACGGAGGGATGGAGAGGGCAACCTTGAAGTTTCTGCGATGGACAATTAGTT
ATTCTACCAACCCCGCAAGGGAATGGTTGGAACCGGTTCATATTGCTAATCGACTCTAGCTTGCTAGTAGTCTGATAA
GCCCTATAGACAACCGCCCTATCAACTTGATGGTAAGGTTTTGGCTTACCATGGTTGTAACGGGTAACGGGGAATCA
GGGTTCGATTCCGGAGAGGACGCCTGAGAAACGGCGTCCACATCTACGGGTGGCAGCAGGCGCGTAAATTGCCCA
ATCTCAACAGAGAGGAGGCGGTGACAATAAATCCCGATGGCTTTGGGGGCAACCCCAGGCTAATCAGAATAAGTAC
ACATTAAATCCCTTAACCAATATAATTGGAGGGCAAGTCTGGTGCCAGCAGCCGCGGTAATTCCAGCTCCAATAGCAT
ATACTAATGTTGTTGCAGTTAAAACGCTCGTAGCTTAATATCTTTTGAGCTACTATCGACTGTTTTCCTTCTAACTCTTC
GGAGGAGGAAGGGGCAGTCGGCTCAAACACTGTAGGTCATGCGGCTAGCAATAGTTGCATAATCGACTGTGCATAA
ACCTTGATGCTCAAGGTAGGCCTTTATAGGGTAGATACACAGTGCATGGCATTGTGGAACAAGGCATCTCGCGGCTT
AGTTGGTGGGCCGCGGGGGCAATGATTAATAGGGAGGAGCGGGGGCCTTCATATTGCAGGGCGAGAGGTGAAATTC
GTTGACCCTTGCAAGATGTCCTACAGCGAAAGCATTGGCCAAGTGCCTCCCCATTAGTCAAGAACGAAAGTTTGGG
GATCAAAGACGATCAGATACCGTCGTAGTCCAAACCATAAACTATGTCGACCAGCGATTAGGCGGGCTACCTTCTTC
GAGAGCTGCCTAGCAGCTTGTGGGAAACCATGAGTGCTTGGACTCTGGGGGGAGTATGGTCGCAAGGCTGAAACTT
AAAGGAATTGACGGAAGGGCACACCATGGAGTGGAGCCTGCGGCTTAATTTGACTCAACACGGGAAAGCTTACCA
AGCTCAGATATGATTAGGATTGACAGACTAAAAGATCTTTCATGATCTCATAAGTGGTGGTGCATGGTCGTTCTTAGT
TGGTGGAGTGATTTGTCAGGTCAATTCCGGTAACGGACGAGACCTCGACCTGCTAACTAGTGGGATTCATTCTTTCG
ATTGACGAGGCAGGTATTGCTTTGATTATAGGGGGCAACTTCTATAGTCGGGTAGTGCTTGTAGTAGTCTGGAGGAGT
GGATTTCAAAAATTAAATTAATCTTCCTAGAGGTACTTCTGGCTCTAAGCCAGAGGAAGTCCGAGGCAATAACAGGT
CTGTGATGCCCTTAGATATCTTGGGCCGCACGCGTGCTACAATGTAGGCGCTAATGAGTTTATTTACATCCATCTCCGC
AAGTGAGTATGGTAATCTTGGAATCACCTGCGTGATTGGGCTTGACCACTGTAACTGTGGTCATCAACGAGGAATTC
CTTGTATGCGCGAGTCACTATCTCGCGCAGAATCTGTCCCTGCCCTTTGTACACACCGCCCGTCGCTCCTACCGATCG
AACGATCAGGTAAAGTGGACAGACTGGAACCCGCAAGGGTACTTGGAAGTCCATTGAACCTCGCCGTTTAGAGGA
AGGAGAAGTCGTAACAAGGTATCGGTAGGTGAACCTGCAGAAGGATCA

>OM677256.1 *Cavenderia parvibrachiata* Landolt TH20C
GAAGAAACCTTTGCCAGACGGGCTTCATTCACAACGGTTGTATCTTCCAGGGCATCCGGGTCGCAAGACCTTCTGG
ATAACCGCAGTAAATCGGGGCTAATACATACAAACGGAGGGATGGAGAGGGCAACCTTGAAGTTTCTGCGATGGAC
AATTAGTTATTCGACCAACCCCGCAAGGGAATGGTTGGAACCGGTTCATATTGCTAATCGACTCTAGCTTGCTAGTAG
TCTGATAAGCCCTATAGACAACCGCCCTATCAACTTGATGGTAAGGTTTTGGCTTACCATGGTTGTAACGGGTAACGG
GGAATCAGGGTTCGATTCCGGAGAGGACGCCTGAGAAACGGCGTCCACATCTACGGGTGGCAGCAGGCGCGTAAA
TTGCCCAATCTCAACAGAGAGGAGGCGGTGACAATAAATCCCGATGGCTTTGGGGGCAACCCCAGGCTAATCAGAA
TAAGTACACATTAAATCCCTTAACCAATATAATTGGAGGGCAAGTCTGGTGCCAGCAGCCGCGGTAATTCCAGCTCC
AATAGCATATACTAATGTTGTTGCAGTTAAAACGCTCGTAGCTTAATATCTTTTGAGCTACTATCGATTGTTATCCTGTT
GACCCGCAAGGTGATGCAGGAGGCAGTCGGCTCAAACACTGTAGGTCATGCGGCTAGCAATAGTTGCATAATCGAC

TGTGCATAAACCTTGATGCTCAAGGTAGGCCTTTATAGGGTAGATACACAGTGCATGGCATTGTGGAACAAGGCATC
TCGCGGCTTAGTTGGTGGGCCGCGGGGGCAATGATTAATAGGGAGGAGCGGGGGCCTTCATATTGCAGGGCGAGAG
GTGAAATTCGTTGACCCTTGCAAGATGTCCTACAGCGAAAGCATTGGCCAAGTGCCTCCCCATTAGTCAAGAACGA
AAGTTTGGGGATCAAAGACGATCAGATACCGTCGTAGTCCAAACCATAAACTATGTCGACCAGCGATTAGGCGGGCT
ACCTTCTTCGAGAGCTGCCTAGCAGCTTGTGGGAAACCATGAGTGCTTGGACTCTGGGGGGAGTATGGTCGCAAGG
CTGAAACTTAAAGGAATTGACGGAAGGGCACACCATGGAGTGGAGCCTGCGGCTTAATTTGACTCAACACGGGAA
AGCTTACCAAGCTCAGATATGATTAGGATTGACAGACTAAAAGATCTTTCATGATCTCATAAGTGGTGGTGCATGGTC
GTTCTTAGTTGGTGGAGTGATTTGTCAGGTCAATTCCGGTAACGGACGAGACCTCGACCTGCTAACTAGTGGGATTC
ATTCTTTCGATTGACGAGGCAGGTCTTGCTTTGATTATAGGGGGCAACTTCTATAGTCGGGTACGGTTTGTAGTAGTC
TGGAGGAGTGGATTTCAAAAATTAAATTAATCTTCCTAGAGGTACTTCTGGCTCTAAGCCAGAGGAAGTCCGAGGCA
ATAACAGGTCTGTGATGCCCTTAGATATCTTGGGCCGCACGCGTGCTACAATGTAGGCGCTAATGAGTTTATTTACAT
CCGTCTCCGCAAGGAGTACGGTAATCTTGGAATCACCTGCGTGATTGGGCTTGACCACTGTAACTGTGGTCATCAAC
GAGGAATTCCTTGTATGCGCGAGTCACTATCTCGCGCAGAATCTGTCCCTGCCCTTTGTACACACCGCCCGTCGCTCC
TACCGATCGAACGATCAGGTAAAGTGGACAGACTGGAGCCCGCAAGGGTTCTTGGAAGTCCATTGAACCTCGCCGT
TTAGAGGAAGGAGAAAGTTCA

>OM677257.1 *Cavenderia parvibrachiata* Landolt 2019TH20C
CGGGGATTTTGGTTAAGGGATTAAGCCATGCATGTCTAAGTATAAGCTCTTGTACGGCTAGACTGCAGACGGCTCATT
ACAACGGTTGTATCTTCCAGGGCATCCGGGTCGCAAGACCTTCTGGATAACCGCAGTAAATCGGGGCTAATACATAC
AAACGGAGGGATGGAGAGGGCAACCTTGAAGTTTCTGCGATGGACAATTAGTTATTCGACCAACCCCGCAAGGGAA
TGGTTGGAACCGGTTCATATTGCTAATCGACTCTAGCTTGCTAGTAGTCTGATAAGCCCTATAGACAACCGCCCTATC
AACTTGATGGTAAGGTTTTGGCTTACCATGGTTGTAACGGGTAACGGGGAATCAGGGTTCGATTCCGGAGAGGACG
CCTGAGAAACGGCGTCCACATCTACGGGTGGCAGCAGGCGCGTAAATTGCCCAATCTCAACAGAGAGGAGGCGGT
GACAATAAATCCCGATGGCTTTGGGGGCAACCCCAGGCTAATCAGAATAAGTACACATTAAATCCCTTAACCAATATA
ATTGGAGGGCAAGTCTGGTGCCAGCAGCCGCGGTAATTCCAGCTCCAAATAGCATATACTAATGTTGTTGCAGTTAA
AACGCTCGTAGCTTAATATCTTTTGAGCTACTATCGATTGTTATCCTGTTGACCCGCAAGGTGATGCAGGAGGCAGTC
GGCTCAAACACTGTAGGTCATGCGGCTAGCAATAGTTGCATAATCGACTGTGCATAAACCTTGATGCTCAAGGTAGG
CCTTTATAGGGTAGATACACAGTGCATGGCATTGTGGAACAAGGCATCTCGCGGCTTAGTTGGTGGGCCGCGGGGGC
AATGATTAATAGGGAGGAGCGGGGGCCTTCATATTGCAGGGCGAGAGGTGAAATTCGTTGACCCTTGCAAGATGTCC
TACAGCGAAAGCATTGGCCAAGTGCCTCCCCATTAGTCAAGAACGAAAGTTTGGGGATCAAAGACGATCAGATACC
GTCGTAGTCCAAACCATAAACTATGTCGACCAGCGATTAGGCGGGCTACCTTCTTCGAGAGCTGCCTAGCAGCTTGT
GGGAAACCATGAGTGCTTGGACTCTGGGGGGAGTATGGTCGCAAGGCTGAAACTTAAAGGAATTGACGGAAGGGC
ACACCATGGAGTGGAGCCTGCGGCTTAATTTGACTCAACACGGGAAAGCTTACCAAGCTCAGATATGATTAGGATTG
ACAGACTAAAAGATCTTTCATGATCTCATAAGTGGTGGTGCATGGTCGTTCTTAGTTGGTGGAGTGATTTGTCAGGTC
AATTCCGGTAACGGACGAGACCTCGACCTGCTAACTAGTGGGATTCATTCTTTCGATTGACGAGGCAGGTCTTGCTT
TGATTATAGGGGGCAACTTCTATAGTCGGGTACGGTTTGTAGTAGTCTGGAGGAGTGGATTTCAAAAATTAAATTAAT
CTTCCTAGAGGTACTTCTGGCTCTAAGCCAGAGGAAGTCCGAGGCAATAACAGGTCTGTGATGCCCTTAGATATCTT
GGGCCGCACGCGTGCTACAATGTAGGCGCTAATGAGTTTATTTACATCCGTCTCCGCAAGGAGTACGGTAATCTTGG
AATCACCTGCGTGATTGGGCTTGACCACTGTAACTGTGGTCATCAACGAGGAATTCCTTGTATGCGCGAGTCACTAT
CTCGCGCAGAATCTGTCCCTGCCCTTTGTACACACCGCCCGTCGCTCCTACCGATCGAACGATCAGGTAAAGTGGAC
AGACTGGAGCCCGCAAGGGTTCTTGGAAGTCCATTGAACCTCGCCGTTTAGAGGGAAGGAAGGAATTTCGAAAAC
CCA

>AM168091.1 *Cavenderia parvispora* OS126
AACCTGGTTGATCCTGCCAGTAGTCATATGCTTGTCTCAAGGATTAAGCCATGCATGTCTAAGTATAAGCCCTAGTAC
GGCTAGACTGCAGACGGCTCATTACAACGGTTGCAGCTTACAGAGCATCCGGGTCGCAAGACCTTCTGGATAACCG
CAGTAAATCGGGGCTAATACATACAAACGGAGGGGCGGAGCGGGCAACCGCGAAGCTTCTGCGATGGACACTTAGC
TATTCGACTGACCCCTCACGGGAATGGTTGGAACCGGTTCATATTGCTAATCGACTTGTGGCTTGCCTCAAGTCTGAT
AAGCTCTATAGGCAACCGCCCTATCAACTTGATGGTAAGGTATTGGCTTACCATGGTTGTAACGGGTAACGGGGAAT
CAGGGTTCGATTCCGGAGAGGACGCCTGAGAAACGGCGTCCACATCTACGGGTGGCAGCAGGCGCGTAAATTGCCC
AATCTCAATAGAGAGGAGGCGGTGACAATAAATCCCGATGGCCATGGGGGCAACCCCGGGCCAATCAGAATAAGTA
CACATTAAATACCCTAACCAATATAATTGGAGGGCAAGTCTGGTGCCAGCAGCCGCGGTAATTCCAGCTCCAATAGC
GTATACTAATGTTGTTGCAGTTAAAACGCTCGTAGCTGAACAACTTGATGGCAAATTCAGGTCAATGTGGCACGTCC
ATGGGGGGAAACCTCTATGCGGCAGTGTCCTTGACCGGCTAAGCAGGTAGGTAGTTTGGCGTGTGTAGTAATACATG
CGTCAGGCGATCGACTGTGCATAAACCTTGATGCTCAAGGTAAAGACGACTCTAGTCAAAGATACGCAGTGCATGG
CATTGTGGAACAAGGCGATCCGTAGCTTGGTTGGTGAGCTACGGGTGCAATGATTAATAGGGAGGAGCGGGGGCCT
TCATATTGCCGGGCGAGAGGTGAAATTCGTTGACCCTGGCAAGATGTCCTACAGCGAAAGCATTGGCCAAGTGCCT
CTCCATTAGTCAAGAACGAAAGTTTGGGGATCAAAGACGATCAGATACCGTCGTAGTCCAAACCATAAACTATGTCG
ACCAGCGATTAGGTTCGCCACCTTCTTCGAGAGCAACCTGGCAGCTTGTGGGAAACCATGAGTTCTTGGACTCTGG
GGGGAGTATGGTCGCAAGGCTGAAACTTAAAGGAATTGACGGAAGGGCACACCATGGAGTGGAGCCTGCGGCTTA
ATTTGACTCAACACGGGAAAGCTTACCAAGCTCAGATATGATAAGGATTGACAGACCAAAAGATCTTTCATGATCTC
ATAAGTGGTGGTGCATGGCCGTTCTTAGTTGGTGGAGTGATTTGTCAGGTCAATTCCGGTAACGGACGAGACCTCGA
CCTGCTAACTAGTAGCAGTTGTCTTTTCGCTCGGCGAGGCGTTTTGGGCGTGGAACTTTGATAGGGGCAACTCTATC
TTTGGGAAACGAACGAGGCGTAGTAGTCTGGAGAGGCAGCTGTTCCCATATTAAAATAATCTTCCTAGAGGTACTTC
CAGCTCTAAGTTGGAAGAAGTCCGAGGCAATAACAGGTCTGTGATGCCCTTAAATATCTTGGGCCGCACGCGTGCTA
CAATGTAGGCGCTAATGAGTCGTTCTTAATATCCGGCACCGCAAGGTGTCTGGTAATCCCCAATCCCCTGCGTGATTG
GGATTGGTCACTGTAACTGTGACCATCAACGAGGAATTCCTTGTATGCGCGGGTCACTATCCCGCGCAGAATCTGTC
CCTGCCCTTTGTACACACCGCCCGTCGCTCCTACCGATCGAACGGTAAGGTAAAGTGGACGGACAGCTGTCCTCAC
GGATGGCTGGAAGTCCATTGAACCTCGTCGTTTAGAGGAAGGAGAAGTCGTAACAAGGTATCGGTAGGTGAACCTG
CAGAAGGATCAA

>MH745572.1 *Cavenderia protodigitata* TH18BA
GTTGGGGGGATAGCCTGCATGTCTAAGTATAAGCTCTTGTACGGCTAGACTGCAGACGGCTCATTACAACGGTTGTAT
CTTCCAGGGCATCCGGGTCGCAAGACCTTCTGGATAACCGCAGTAAATCGGGGCTAATACATACAAACGGAGGGAT
GGAGAGGGCAACCTTGAAGTTTCTGCGATGGACAATTAGTTATTCGACCAACCCCGCAAGGGAATGGTTGGAACCG

GTTCATATTGCTAATCGACTCTAGCTTGCTAGTAGTCTGATAAGCCCTATAGACAACCGCCCTATCAACTTGATGGTAA
GGTTTTGGCTTACCATGGTTGTAACGGGTAACGGGGAATCAGGGTTCGATTCCGGAGAGGACGCCTGAGAAACGGC
GTCCACATCTACGGGTGGCAGCAGGCGCGTAAATTGCCCAATCTCAACAGAGAGGAGGCGGTGACAATAAATCCCG
ATGGCTTTGGGGGCAACCCCAGGCTAATCAGAATAAGTACACATTAAATCCCTTAACCAATATAATTGGAGGGCAAG
TCTGGTGCCAGCAGCCGCGGTAATTCCAGCTCCAATAGCATATACTAATGTTGTTGCAGTTAAAACGCTCGTAGCTTA
ATATCTTTTGAGCTACTATCGATTGTTATCCTGCTGACCCGAAAGGTGATGTAGGAGGCAGTCGGCTCAAACACTGTA
GGTCATGCGGCTAGCAATAGTTGCATAATCGACTGTGCATAAACCTTGATGCTCAAGGTAGGCCTTTATAGGGTAGAT
ACACAGTGCATGGCATTGTGGAACAAGGCATCTCGCGGCTTAGTTGGTGGGCCGCGGGGGCAATGATTAATAGGGA
GGAGCGGGGGCCTTCATATTGCAGGGCGAGAGGTGAAATTCGTTGACCCTTGCAAGATGTCCTACAGCGAAAGCAT
TGGCCAAGTGCCTCCCCATTAGTCAAGAACGAAAGTTTGGGGATCAAAGACGATCAGATACCGTCGTAGTCCAAAC
CATAAACTATGTCGACCAGCGATTAGGCGGGCTACCTTCTTCGAGAGCTGCCTAGCAGCTTGTGGGAAACCATGAGT
GCTTGGACTCTGGGGGGAGTATGGTCGCAAGGCTGAAACTTAAAGGAATTGACGGAAGGGCACACCATGGAGTGG
AGCCTGCGGCTTAATTTGACTCAACACGGGAAAGCTTACCAAGCTCAGATATGATTAGGATTGACAGACTAAAAGAT
CTTTCATGATCTCATAAGTGGTGGTGCATGGTCGTTCTTAGTTGGTGGAGTGATTTGTCAGGTCAATTCCGGTAACGG
ACGAGACCTCGACCTGCTAACTAGTGGGATTCATTCTTTCGATTGACGAGGCAGGTCTTGCTTTGATTATAGGGGGC
AACTTCTATAGTCGGGTACGGTTTGTAGTAGTCTGGAGGAGTGGATTTCAAAAATTAAATTAATCTTCCTAGAGGTAC
TTCTGGCTCTAAGCCAGAGGAAGTCCGAGGCAATAACAGGTCTGTGATGCCCTTAGATATCTTGGGCCGCACGCGTG
CTACAATGTAGGCGCTAATGAGTTTATTTACATCCATCTCCGCAAGGAGTATGGTAATCTTGGAATCACCTGCGTGATT
GGGCTTGACCACTGTAACTGTGGTCATCAACGAGGAATTCCTTGTATGCGCGAGTCACTATCTCGCGCAGAATCTGT
CCCTGCCCTTTGTACACACCGCCCGTCGCTCCTACCGATCGAACGATCAGGTAAAGTGGACAGACTGGAGCCCGCA
AGGGTTCTTGGAAGTCCATTGAACCTCGCCGTTAGAGGAAGGAGAATGCCCA

>OM677258.1 *Cavenderia protumula* Landolt TH20A
TGGGGTTTTTGGGTTTAGGAAAAAAGCCATGCATGTCTAAGTATAAGCTCTTGTACGGCTAGACTGCAGACGGCTCA
TTACAACGGTTGTATCTTCCAGGGCATCCGGGTCGCAAGACCTTCTGGATAACCGCAGTAAATCGGGGCTAATACAT
ACAAACGGAGGGATGGAGAGGGCAACCTTGAAGTTTCTGCGATGGACAATTAGTTATTCGACCAACCCCGCAAGGG
AATGGTTGGAACCGGTTCATATTGCTAATCGACTCTAGCTTGCTAGTAGTCTGATAAGCCCTATAGACAACCGCCCTA
TCAACTTGATGGTAAGGTTTTGGCTTACCATGGTTGTAACGGGTAACGGGGAATCAGGGTTCGATTCCGGAGAGGAC
GCCTGAGAAACGGCGTCCACATCTACGGGTGGCAGCAGGCGCGTAAATTGCCCAATCTCAACAGAGAGGAGGCGG
TGACAATAAATCCCGATGGCTTTGGGGGCAACCCCAGGCTAATCAGAATAAGTACACATTAAATCCCTTAACCAATAT
AATTGGAGGGCAAGTCTGGTGCCAGCAGCCGCGGTAATTCCAGCTCCAATAGCATATACTAATGTTGTTGCAGTTAA
AACGCTCGTAGCTTAATATCTTTTGAGCTACTATCGATTGTTATCCTGCTGACCCGAAAGGTGATGTAGGAGGCAGTC
GGCTCAAACACTGTAGGTCATGCGGCTAGCAATAGTTGCATAATCGACTGTGCATAAACCTTGATGCTCAAGGTAGG
CCTTTATAGGGTAGATACACAGTGCATGGCATTGTGGAACAAGGCATCTCGCGGCTTAGTTGGTGGGCCGCGGGGGC
AATGATTAATAGGGAGGAGCGGGGGCCTTCATATTGCAGGGCGAGAGGTGAAATTCGTTGACCCTTGCAAGATGTCC
TACAGCGAAAGCATTGGCCAAGTGCCTCCCCATTAGTCAAGAACGAAAGTTTGGGGATCAAAGACGATCAGATACC
GTCGTAGTCCAAACCATAAACTATGTCGACCAGCGATTAGGCGGGCTACCTTCTTCGAGAGCTGCCTAGCAGCTTGT
GGGAAACCATGAGTGCTTGGACTCTGGGGGGAGTATGGTCGCAAGGCTGAAACTTAAAGGAATTGACGGAAGGGC
ACACCATGGAGTGGAGCCTGCGGCTTAATTTGACTCAACACGGGAAAGCTTACCAAGCTCAGATATGATTAGGATTG
ACAGACTAAAAGATCTTTCATGATCTCATAAGTGGTGGTGCATGGTCGTTCTTAGTTGGTGGAGTGATTTGTCAGGTC
AATTCCGGTAACGGACGAGACCTCGACCTGCTAACTAGTGGGATTCATTCTTTCGATTGACGAGGCAGGTCTTGCTT
TGATTATAGGGGGCAACTTCTATAGTCGGGTACGGTTTGTAGTAGTCTGGAGGAGTGGATTTCAAAAATTAAATTAAT
CTTCCTAGAGGTACTTCTGGCTCTAAGCCAGAGGAAGTCCGAGGCAATAACAGGTCTGTGATGCCCTTAGATATCTT
GGGCCGCACGCGTGCTACAATGTAGGCGCTAATGAGTTTATTTACATCCATCTCCGCAAGGAGTATGGTAATCTTGGA
ATCACCTGCGTGATTGGGCTTGACCACTGTAACTGTGGTCATCAACGAGGAATTCCTTGTATGCGCGAGTCACTATCT
CGCGCAGAATCTGTCCCTGCCCTTTGTACACACCGCCCGTCGCTCCTACCGATCGAACGATCAGGTAAAGTGGACAG
ACTGGAGCCCGCAAGGGTTCTTGGAAGTCCATTGAACCTCGCCGTTAGAGGAAGGAAGAAAGTCGAAACCAA

>HQ141518.1 *Cavenderia pseudoaureostipes* TH39A
AACCTGGTTGATCCTGCCAGTAGTCATATGCTTGTTTCAAGGATTAAGCCATGCATGTCTAAGTATAAGCTCTTGTAC
GGCTAGACTGCAGACGGCTCATTACAACGGTTGTATCTTCCAGGGCATCCGGGTCGCAAGACCTTCTGGATAACCGC
AGTAAATCGGGGCTAATACATACAAACGGAGGGATGGAGAGGGCAACCTTGAAGTTTCTGCGATGGACAATTAGTT
ATTCGACCAACCCCGCAAGGGAATGGTTGGAACCGGTTCATATTGCTAATCGACTCTAGCTTGCTAGTAGTCTGATAA
GCCCTATAGACAACCGCCCTATCAACTTGATGGTAAGGTTTTGGCTTACCATGGTTGTAACGGGTAACGGGGAATCA
GGGTTCGATTCCGGAGAGGACGCCTGAGAAACGGCGTCCACATCTACGGGTGGCAGCAGGCGCGTAAATTGCCCA
ATCTCAACAGAGAGGAGGCGGTGACAATAAATCCCGATGGCTTTGGGGGCAACCCCAGGCTAATCAGAATAAGTAC
ACATTAAATCCCTTAACCAATATAATTGGAGGGCAAGTCTGGTGCCAGCAGCCGCGGTAATTCCAGCTCCAATAGCAT
ATACTAATGTTGTTGCAGTTAAAACGCTCGTAGCTTAATATCTTTTGAGCTACTATCGACTGTTATTTCTGTGGATCCG
CAAGGTGAATCAGGGAGGCAGTCGGCTCAAACACTGTAGGTCATGCGGCTAGCAATAGTTGCATAATCGACTGTGC
ATAAACCTTGATGCTCAAGGTAGGCCTTTATAGGGTAGATACACAGTGCATGGCATTGTGGAACAAGGCATCTCGCG
GCTTAGTTGGTGGGCCGCGGGGGCAATGATTAATAGGGAGGAGCGGGGGCCTTCATATTGCAGGGCGAGAGGTGAA
ATTCGTTGACCCTTGCAAGATGTCCTACAGCGAAAGCATTGGCCAAGTGCCTCCCCCTTAGTCAAGAACGAAAGTTT
GGGGATCAAAGACGATCAGATACCGTCGTAGTCCAAACCATAAACTATGTCGACCAGCGATTAGGCGGGCTACCTTC
TTCGAGAGCTGCCTAGCAGCTTGTGGGAAACCATGAGTGCTTGGACTCTGGGGGGAGTATGGTCGCAAGGCTGAAA
CTTAAAGGAATTGACGGAAGGGCACACCATGGAGTGGAGCCTGCGGCTTAATTTGACTCAACACGGGAAAGCTTAC
CAAGCTCAGATATGATTAGGATTGACAGACTAAAAGATCTTTCATGATCTCATAAGTGGTGGTGCATGGTCGTTCTTA
GTTGGTGGAGTGATTTGTCAGGTCAATTCCGGTAACGGACGAGACCTCGACCTGCTAACTAGTGGGATTCATTCTTT
CGATTGACGAGGCAGGTCTTGCTTTGATTACAGGGGGCAACTTCTGTAGTCGGGTACGGTTTGTAGTAGTCTGGAGG
AGTGGGTTTCAAAAATTAAATTAATCTTCCTAGAGGTACTTCTGGCTCTAAGCCAGAGGAAGTCCGAGGCAATAACA
GGTCTGTGATGCCCTTAGATATCTTGGGCCGCACGCGTGCTACAATGTAGGCGCTAATGAGTTTATTTACATCCATCTC
CGCAAGGAGTATGGTAATCTTGGAATCACCTGCGTGATTGGGCTTGACCACTGTAACTGTGGTCATCAACGAGGAAT
TCCTTGTATGCGCGAGTCACTATCTCGCGCAGAATCTGTCCCTGCCCTTTGTACACACCGCCCGTCGCTCCTACCGAT
CGAACGATCAGGTAAAGTGGACAGACTGGAACCCGCAAGGGTACTTGGAAGTCCATTGAACCTCGCCGTTAGAGG
AAGGAGAAGTCGTAACAAGGTATCGGTAGGTGAACCTGCAGAAGGATCA

>AM168081.1 *Cavenderia stellata* SAB7B
AACCTGGTTGATCCTGCCAGTAGTCATATGCTTGTTTCAAGGATTAAGCCATGCATGTCTAAGTATAAGCTCTTGTAC
GGCTAGACTGCAGACGGCTCATTACAACGGTTGTAGCTTCCAGGACATCCGGGTCGCAAGGCCTTCTGGATAACCG
CAGTAAATCGGGGCTAATACATATAAACGGAGGGGTAGAGGGGGCAACCTTGAAGCTTCTGCGATGGACACTTAGC
TATTCGACCAACCCCGCAAGGGAGTGGTTGGAACCGGTTCATATTGCTAATCGACTCTAGCTTGCTAGTAGTCTGATA
AGTTCTATAGACAACTGCCCTATCAACTTGATGGTAAGGTTTTGGCTTACCATGGTTATAACGGGTAACGGGGAATCA
GGGTTCGATTCCGGAGAGGACGCCTGAGAAACGGCGTCCACATCTACGGGTGGCAGCAGGCGCGTAAATTGCCCA
ATCTCAACAGAGAGGAGGCGGTGACAATAAATCCCGATGGCTTTGGGGGCAACCCCAGGCCAATCAGAATAAGTAC
ACATTAAATCCCCTAACCAATATAATTGGAGGGCAAGTCTGGTGCCAGCAGCCGCGGTAATTCCAGCTCCAATAGCAT
ATACTAATGTTGTTGCAGTTAAAACGCTCGTAGCTCAATATCTTTTGAGCTATTTACGATCGTGTCACTCACCTGATGG
CCCTTAAAGCTTGAGGGTGAGCGCGGTCGGCTCGAACACTGTAGGTCACGGTGGTCTTAGCAATAGGGCCATCGTA
ATCGACTGTGCATAAACCTTGATGCTCAAGGTAGGCCTCTGGGGGTAGATACGCAGTGCATGGCATTGTGGAACAAG
GCATCTCGCGGCTTAGTTGGTGGGCCGCGGGGGCAATGATTAATAGGGAGGAGCGGGGGCCTTCATATTGCAGGGC
GAGAGGTGAAATTCGTTGACCCTTGCAAGATGTCCTACAGCGAAAGCATTGGCCAAGTGCCTCTCCATTAGTCAAG
AACGAAAGTTTGGGGATCAAAGACGATCAGATACCGTCGTAGTCCAAACCATAAACTATGTCGACCAGTGATTAGGC
GGGCTACCTTCTTCGAGAGCTGCCTAGCAACTTGTGGGAAACCATGAGTGCTTGGACTCTGGGGGGAGTATGGTCG
CAAGGCTGAAACTTAAAGGAATTGACGGAAGGGCACACCATGGAGTGGAGCCTGCGGCTTAATTTGACTCAACAC
GGGAAAGCTTACCAAGCTCAGATATGATAAGGATTGACAGACTAAAAGATCTTTCATGATCTCATAAGTGGTGGTGC
ATGGTCGTTCTTAGTTGGTGGAGTGATTTGTCAGGTCAATTCCGGTAACGGACGAGACCTCGACCTGCTAACTAGT
GGGGCTCATTGGTTCAAATGGCGAAGCGAATTTTGCTTTTACTATGGGTGGGCAACCATTCATAGTGGGGGTAGGGA
GCGTATGTAGCCTGGGCCAGTGGGTTTCAAAAATTAAAATTAATCTTCCTAGAGGTACTTCTGGCTCTAAGCCAGAG
GAAGTCCGAGGCAATAACAGGTCTGTGATGCCCTTAGATATCTTGGGCCGCACGCGTGCTACAATGTAGGCGCTAAT
GAGTCGTAACATTCCAGCACCGCAAGGTGTCTGGTAATCTTGGAATCCCCTGCGTGATTGGGCTTGACCACTGTAAC
TGTGGTCATCAACGAGGAATTCCTTGTATGCGCGAGTCACTATCTCGCGCAGAATCTGTCCCTGCCCTTTGTACACAC
CGCCCGTCGCTCCTACCGATCGAACGATCAGGTAAAGTGGACAGACAGAGACCCGCAAGGGCTTTTGAAAGTCCAT
TGAACCTCGCCGTTTAGAGGAAGGAGAAGTCGTAACAAGGTATCGGTAGGTGAACCTGCAGAAGGATCAA

>HQ141515.1 *Cavenderia subdiscoidea* TH1A
AACCTGGTTGATCCTGCCAGTAGTCATATGCTTGTTTCAAGGATTAAGCCATGCATGTCTAAGTATAAGCTCTTGTAC
GGCTAGACTGCAGACGGCTCATTACAACGGTTGTATCTTCCAGGGCATCCGGGTCGCAAGACCTTCTGGATAACCGC
AGTAAATCGGGGCTAATACATACAAACGGAGGGATGGAGAGGGCAACCTTGAAGTTTCTGCGATGGACAATTAGTT
ATTCGACCAACCCCGCAAGGGAATGGTTGGAACCGGTTCATATTGCTAATCGACTCTAGCTTGCTAGTAGTCTGATAA
GCCCTATAGACAACCGCCCTATCAACTTGATGGTAAGGTTTTGGCTTACCATGGTTGTAACGGGTAACGGGGAATCA
GGGTTCGATTCCGGAGAGGACGCCTGAGAAACGGCGTCCACATCTACGGGTGGCAGCAGGCGCGTAAATTGCCCA
ATCTCAACAGAGAGGAGGCGGTGACAATAAATCCCGATGGCTTTGGGGGCAACCCCAGGCTAATCAGAATAAGTAC
ACATTAAATCCCTTAACCAATATAATTGGAGGGCAAGTCTGGTGCCAGCAGCCGCGGTAATTCCAGCTCCAATAGCAT
ATACTAATGTTGTTGCAGTTAAAACGCTCGTAGCTTAATATCTTTTGAGCTACTATCGACTGTTTTCCTCCTGGATCTT
CGGAAAAGGGAGGGGCAGTCGGCTCAAACACTGTAGGTCATGCGGCTAGCAATAGTTGCATAATCGACTGTGCATA
AACCTTGATGCTCAAGGTAGGCCTTTATAGGGTAGATACACAGTGCATGGCATTGTGGAACAAGGCATCTCGCGGCT
TAGTTGGTGGGCCGCGGGGGCAATGATTAATAGGGAGGAGCGGGGGCCTTCATATTGCAGGGCGAGAGGTGAAATT
CGTTGACCCTTGCAAGATGTCCTACAGCGAAAGCATTGGCCAAGTGCCTCCCCATTAGTCAAGAACGAAAGTTTGG
GGATCAAAGACGATCAGATACCGTCGTAGTCCAAACCATAAACTATGTCGACCAGCGATTAGGCGGGCTACCTTCTT
CGAGAGCTGCCTAGCAGCTTGTGGGAAACCATGAGTGCTTGGACTCTGGGGGGAGTATGGTCGCAAGGCTGAAACT
TAAAGGAATTGACGGAAGGGCACACCATGGAGTGGAGCCTGCGGCTTAATTTGACTCAACACGGGAAAGCTTACCA
AGCTCAGATATGATTAGGATTGACAGACTAAAAGATCTTTCATGATCTCATAAGTGGTGGTGCATGGTCGTTCTTAGT
TGGTGGAGTGATTTGTCAGGTCAATTCCGGTAACGGACGAGACCTCGACCTGCTAACTAGTGGGATTCATTCTTTCG
ATTGACGAGGCAGGTCTTGCTTTGATTATAGGGGGCAACTTCTATAGTCGGGTAGGTTTTGTAGTAGTCTGGAGGAG
TGGATTTCAAAAATTAAATTAATCTTCCTAGAGGTACTTCTGGCTCTAAGCCAGAGGAAGTCCGAGGCAATAACAGG
TCTGTGATGCCCTTAGATATCTTGGGCCGCACGCGTGCTACAATGTAGGCGCTAATGAGTTTATTTACATCCATCTCCG
CAAGGAGTATGGTAATCTTGGAATCACCTGCGTGATTGGGCTTGACCACTGTAACTGTGGTCATCAACGAGGAATTC
CTTGTATGCGCGAGTCACTATCTCGCGCAGAATCTGTCCTTGCCCTTTGTACACACCGCCCGTCGCTCCTACCGATCG
AACGATCAGGTAAAGTGGACAGACTGGAACCCGCAAGGGTACTTGGAAGTCCATTGAACCTCGCCGTTAGAGGAA
GGAGAAGTCGTAACAAGGTATCGGTAGGTGAACCTGCAGAAGGATCA

>OM677259.1 *Cavenderia ungulata* Landolt TH18B
CGGTGTTCTCTACTCCCACTGACATCGTTCTAAGGTATAAGCTCTTGTACGGCTAGACTGCAGACGGCTCATTACAAC
GGTTGTATCTTCCAGGGCATCCGGGTCGCAAGACCTTCTGGATAACCGCAGTAAATCGGGGCTAATACATACAAACG
GAGGGATGGAGAGGGCAACCTTGAAGTTTCTGCGATGGACAATTAGTTATTCGACCAACCCCGCAAGGGAATGGTT
GGAACCGGTTCATATTGCTAATCGACTCTAGCTTGCTAGTAGTCTGATAAGCCCTATAGACAACCGCCCTATCAACTT
GATGGTAAGGTTTTGGCTTACCATGGTTGTAACGGGTAACGGGGAATCAGGGTTCGATTCCGGAGAGGACGCCTGA
GAAACGGCGTCCACATCTACGGGTGGCAGCAGGCGCGTAAATTGCCCAATCTCAACAGAGAGGAGGCGGTGACAA
TAAATCCCGATGGCTTTGGGGGCAACCCCAGGCTAATCAGAATAAGTACACATTAAATCCCTTAACCAATATAATTGG
AGGGCAAGTCTGGTGCCAGCAGCCGCGGTAATTCCAGCTCCAATAGCATATACTAATGTTGTTGCAGTTAAAACGCT
CGTAGCTTAATATCTTTTGAGCTACTATCGATTGTTATCCTGCTGACCCGAAAGGTGATGTAGGAGGCAGTCGGCTCA
AACACTGTAGGTCATGCGGCTAGCAATAGTTGCATAATCGACTGTGCATAAACCTTGATGCTCAAGGTAGGCCTTTAT
AGGGTAGATACACAGTGCATGGCATTGTGGAACAAGGCATCTCGCGGCTTAGTTGGTGGGCCGCGGGGGCAATGAT
TAATAGGGAGGAGCGGGGGCCTTCATATTGCAGGGCGAGAGGTGAAATTCGTTGACCCTTGCAAGATGTCCTACAG
CGAAAGCATTGGCCAAGTGCCTCCCCATTAGTCAAGAACGAAAGTTTGGGGATCAAAGACGATCAGATACCGTCGT
AGTCCAAACCATAAACTATGTCGACCAGCGATTAGGCGGGCTACCTTCTTCGAGAGCTGCCTAGCAGCTTGTGGGAA
ACCATGAGTGCTTGGACTCTGGGGGGAGTATGGTCGCAAGGCTGAAACTTAAAGGAATTGACGGAAGGGCACACC
ATGGAGTGGAGCCTGCGGCTTAATTTGACTCAACACGGGAAAGCTTACCAAGCTCAGATATGATTAGGATTGACAGA
CTAAAAGATCTTTCATGATCTCATAAGTGGTGGTGCATGGTCGTTCTTAGTTGGTGGAGTGATTTGTCAGGTCAATTC
CGGTAACGGACGAGACCTCGACCTGCTAACTAGTGGGATTCATTCTTTCGATTGACGAGGCAGGTCTTGCTTTGATT
ATAGGGGGCAACTTCTATAGTCGGGTACGGTTTGTAGTAGTCTGGAGGAGTGGATTTCAAAAATTAAATTAATCTTCC
TAGAGGTACTTCTGGCTCTAAGCCAGAGGAAGTCCGAGGCAATAACAGGTCTGTGATGCCCTTAGATATCTTGGGCC

GCACGCGTGCTACAATGTAGGCGCTAATGAGTTTATTTACATCCATCTCCGCAAGGAGTATGGTAATCTTGGAATCAC
CTGCGTGATTGGGCTTGACCACTGTAACTGTGGTCATCAACGAGGAATTCCTTGTATGCGCGAGTCACTATCTCGCG
CAGAATCTGTCCCTGCCCTTTGTACACACCGCCCGTCGCTCCTACCGATCGAACGATCAGGTAAAGTGGACAGACTG
GAGCCCGCAAGGGTTCTTGGAAGTCCATTGAACCTCGCCGTTAGAGGAAGAGAAGTCGAAACCAAGGA

>*Cavendaria nanopodia* HOLOTYPE SMA
CGGGTAACGGAGAATCAGGGTTCGATTCCGGAGAGGACGCCTGAGAAACGGCGTCCACACCTACGGGTGGCAGCA
GGCACGTAAATTTCCCAATGCCAATACGGCGAGGGAGCGACAATAAATACTTTTAGCCCTAGGCGCAAGCTTAGGGC
TCAATGAGATTAAGCACAAAGTAAATAAATTAGCCAGTATAATTGGAGGGCAAGTCTGGTGCCAGCAGCCGCGGTAA
TTCCAGCTCCAACAGTATATACTAACGTTGCTGCAGTTAAAACGTTCGTAGCTTAAACACAGGTCTGTAATTTTTGTC
GCCAGTCTAACGCAAGTTAGTCTGGTGACACAGGCGCAGTAGATCGTCGTGTCTAGTCGGCAACGGCTAGGCAAGG
CAGTCAACTGTGCATAAACCTTGATGCTCAAGGTAAGTCCAGAATGGACAGGAAAGTGGTGCATGGCATTGTATAAT
AGGACGTGGAAACCTTATAGGTCAGCAAGTTTCCAGGTAATGATTAATAGGGAGGATCGGGGGCCA

>HQ141510.1 *Acytostelium amazonicum* landolt X
AACCTGGTTGATCCTGCCAGTAGTCATATGCTTGTCTCAAAGATTAAGCCATGCATGTCTAAGTATAAACCTCTATATG
GTGAACCTGCAGAAGGATCAACTACAACATGTGATAAACTACAAGACTTTCGCGCTTCGGCGTTATGGATAACCGCA
GTAAATCGGGGCTAATACATATAAACGAAAGACGAGCAAGCAATTGCGAGTTCTTGCGATTTTTAGCTACTAAAATC
GACCCTTTTAGGTCGTGTTGAAACCGAAAAATATTGCTGATCGAGAATTTCTCGACAAGTCTTATGTGTCACTGCCCT
ATCAACTCGATGGTACGGTATTGGCCTACCATGGTTGCAACGGGTAACGGGGAATTAGGGTTCGATTCCGGAGAGGG
AGCCTGAGAAATGGCTGCCACTTCTACGGAAGGCAGCAGGCGCGCAAATTACTCAATTCCGATACGGAGAAGTAGG
ACAATAAATACAAATGCTTTTCAATTTTTTTGAGAGCAATTTGAATAAGTACAACTTAAATCGCTTAGCTAAAGTGAT
TGGAGGGCAAGTCTGGTGCCAGCAGCCGCGGTAATTCCAGCTCCAATAGCGTATACTAAATTTGTTGCAGTTAAAAC
GCTCGTAGTTAAAGTAAAGGTTACTTGGATAAACAAGAATAATTTTTTTTAGAATTATTTAACGAATTTTTAATTATTTT
CATAGTTAAATTTTCACCAAGTTCCTTATTTTTGGACTTAAAAATTTATTTTTTTGTCTAGTCTACTGTGAGAAAATTGT
AGTGTTTAAAGCAAGCTATCAGCTTGATCAATGCAGCATGGTATAGAAAAATATGACACTAAGTATTTTGTTGGTTTA
ATACTCTAGTGTAATGATTAATAGGGAATGGCGGGGCCGTTCGTATTGATGGGCGAGAGGTGAAATTCGTTGACCCTA
TCAAGACGTACAACAGCGAAAGCATTCGGCAAGTATTTCTCCATTAATCAAGAACGAAAGTTTGGGGATCGAAGAC
GATCAGATACCGTCGTAGTCCAAACCATAAACAATGTCGACCAGGGATTGGACGGAATTTTTTTAAAAAACTCGCTC
AGAACCTTGTGAGAAATCATGAGTGTTTGGACTCTGGGGGGAGTATGGTCGCAAGTCTGAAACTTAAAGGAATTGA
CGGAAGGGCACACAATGGAGTGGAACCTGCGGCTTAATTTGACTCAACTCGGGAAAACTTACCAAGCTCAGATATA
ATAAGGATTGACAGACTAAAAGATCTTTCATGATTGTATAAGTGGTGGTGCATGGTCGTTCTTAGTTGGTGGAGTGAT
TTGTCTGGTCAATTCCGATAACGGACGAGACGTCTACTTGCTAACTAGTAAAATTAATTTTTTCGCTCGGGGATAGTT
AGTTTGTATTGATCTTTATCATCGCAAGGTGGTAATTTTCGTATAGATTGATGTGTACCTTGGAAAAATAAATTTTTTCA
TAAACATTAAACTTCTTAGAAGGACTACCTACCTCAAGTGGGGGGAAGTCGGACGCAATAACAGGTCTGTGATGCC
CTTAGATACCTTGGGCTGCACGCGCGTTACAATGTAGAAAAGAAAAAGGCTTCCCGGAGCCGTAAGGCTCTGGTAA
TCATATGAATTTTCTACGTAATAGGGATTGATCTTTGTAATTATCGATCATAAACGAGGAATTCCTTGTAAGCGTAAATC
ATTACTTTACGCTGAATATGTCCCTGCCCTTTGTACACACCGCCCGTCGCTCCTACCGATCGAACGATTAGGTAAAAT
TGACGGACTTTGACTAACTCATGAGATATGTATGCCTGGGTGAACCTGCAGAAGGATCACCAGTAGATACATATGTCT
TGTCTCAAAGATTAAGCCAGCA

>HQ141511.1 *Acytostelium amazonicum* HN1B1
AACCTGGTTGATCCTGCCAGTAGTCATATGCTTGTCTCAAAGATTAAGCCATGCATGTCTAAGTATAAACCTTTATACG
GTGAACCTGCAGAAGGATCAATACAACATGTGATAAACTACAAGACTTTCGCGCTTCGGCGTTTTGGATAACCGCAG
TAAATCGGGGCTAATACATATAAACGAAAGGCGAGCAAGTAATTGCGAGTCCTTGCGATTGTTAGCCATTTTAACCG
ACCCTCTTGAGGTTGTGTTGAACCCGAACAATATTGCTGATCGAGAATTTATTCTCGACAAGTCTTATGTGTCACTGC
CCTATCAACTCGATGGTACGGTATTGGCCTACCATGGTTGCAACGGGTAACGGGGAATCAGGGTTCGATTCCGGAGA
GGGAGCCTGAGAAATGGCTGCCACTTCTACGGAAGGCAGCAGGCGCGCAAATTACTCAATCCCAATACGGGGAAGT
AGTGACAATAAATACAAATGCCTTTCCATTTTATGGAGGGCAATTTGAATAAGTACAATCTAAATCGCTTAGCCAAAG
TGATTGGAGGGCAAGTCTGGTGCCAGCAGCCGCGGTAATTCCAGCTCCAATAGCGTATACTAAATTTGTTGCAGTTA
AAACGCTCGTAGTTGAAGTAGAAATTATTTGGAGAATAGAAGGATCTTTTTAAAGGTCACTTTAAAGACACTTTTCC
ACTTAGTGGTCAAGTTGTCACCAAATATTTCTTTAAGGGCAATGGAATTTTTATTCATTTGCCTTGTCTACTGTGAGAA
AATTGCAGTGTTCAAAGCAAGCGTAAAGCTTGATCAATGCAGCATGGTATAGAGAAACACGACACTAAATATTTTGT
TGGTTAATATTCTAGTGTAATGGTTAATAGGGAATGGCGGGGCCGTTCGTATTGATGGGCGAGAGGTGAAATTCGTTG
ACCCTATCAAGACGTACAACAGCGAAAGCATTCGGCAAGTAATTCTCCATTAATCAAGAACGAAAGTTTGGGGATCG
AAGACGATCAGATACCGTCGTAGTCCAAACTATAAACTATGTCGACCAGGGATTGGACGGATCCTTTTTAAAAAACC
CGCTCAGAACCTTGTGGGAAACCATGAGTGTTTGGACTCTGGGGGGAGTATGGTCGCAAGTCTGAAACTTAAAGGA
ATTGACGGAAGGGCACACAATGGAGTGGAACCTGCGGCTTAATTTGACTCAACTCGGGAAAACTTACCAAGCTCAG
ATATAATAAGGATTGACAGACTAAAAGATCTTTCATGATTGTATAAGTGGTGGTGCATGGTCGTTCTTAGTTGGTGGA
GTGATTTGTCTGGTCAATTCCGATAACGGACGAGACGTCTACTTGCTAACTAGTGTAAATTATTATGCCGAACGAGAG
ATAGCTAGTTTGTATTGATTTTTATTGACGCAAGTCGGTAGACTTCGTATAGATTAGTGTGTATCTTGGTAAAATAATTT
ATATCATAAACTTTAAACTTCTTAGAAGGACTACCTACCTCAAGTGGGGGGAAGTCGGACGCAATAACAGGTCTGTG
ATGCCCTTAGATACCTTGGGCTGCACGCGCGTTACAATGTAGAGTGGAAAAAGGCTTCCCGTGACCGGAAGGTCAT
GGTAATCATTTGAATACTCTACGTAATAGGGATTGATCTTTGTAATTATCGATCATAAACGAGGAATTCCTTGTAAGCG
TAAATCATTACTTTACGCTGAATATGTCCCTGCCCTTTGTACACACCGCCCGTCGCTCCTACCGATCGAACGATTAGGT
AAAATTGACGGACGGTGAACCTGCAGAAGGATCAAATGCCTAACCTGCGATTGATCCTGCCGAGTAGTCATATGCTT
GTCTCAAAGATAAGCC

>AM168115.1 *Acytostelium anastomosans* PP1
AACCTGGTTGATCCTGCCAGTAGTCATATGCTTGTCTCAAAGATTAAGCCATGCATGTCTAAGTATAAACTTTTATACG
GTGAAACTGCAGACGGCTCATTACAACAGTGATAAACTACAGAACTTTCGAGCGTAAGCTTTTTGGATAACCGCAGT
AAATCGGGGCTAATACATATAAACGGAAGGCGATTGGGCAACCAAGAGTCCTTGCGATTACTAGCTTCAAACCAACC
TCTATGAGATTGAGTTGAAACCGAATAATATTGCTGATCAGAATTTAATTCTGACAAGTTCTATGTGTCACTGCCCTAT
CAACTTTCGATGGTACGGTATTGGCCTACCATGGTTGTAACGGGTAACGGGGAATCAGGGTTCGATTCCGGAGAGGG
AGCCTGAGAAATGGCTACCACTTCTACGGAAGGCAGCAGGCGCGCAAATTACTCAATCCCAATACGGGGAAGTAGT

GACAATAAATACTAATGTTCTTCCATTTTATGGAGAACAATTGGAATAAGTACAACGTAAATAGCTTAGCAAAAGTAA
TTGGAGGGCAAGTCTGGTGCCAGCAGCCGCGGTAATTCCAGCTCCAATAGCGTATACTAAATTTGTTGCAGTTAAAA
AGCTCGTAGTTTAGGCAGAGGTCATTCGGATTCATTAATTTCACCTTCAAAAGTGACTTTAAAGGCTATATCACGCTT
CGGTTGTGTTTATGGTCACCGATGACTTGTATTATGACGTTATTTGTTAGTTTACTAGCTTATAATCGTCAATCCACTGT
GAGAAAATTGTGGTGTTTAAAGCAGGCGTTCTAGTCTGATCAATGCAGCATGGTATGGTAAAATATGACACTTAACAT
TTGTTGGTTTACTTTGTTATAGTGTAATGACTAATAGGGAAGGGCGGGGCCGTTCATATTGATGGGCGAGAGGTGAA
ATTCGTTGACCCTATCAAGATGCACTACAGCGAAAGCATTCGGCAAGTACTTCTCCATTAATCAAGAACGAAAGTTT
GGGGATCGAAGACGATCAGATACCGTCGTAGTCCAAACCATAAATGATGTCGATCAGGGATCGGTAGGATTTAATTG
TAAATTCTTATCGGAACCTTGTGAGAAATCATGAATGTTTGGACTCTGGGGGGAGTATGGTCGCAAGGCTGAAACTT
AAAGGAATTGACGGAAGGGCACACAATGGAGTGGAACCTGCGGCTTAATTTGACTCAACTCGGGAAAACTTACCA
AGCTCAGATATAATAAGGATTGACAGACTAAAAGATCTTTCATGATTGTATAAGTGGTGGTGCATGGTCGTTCTTAGT
TGGTGGAGTGATTTGTCTGGTCAATTCCGATAACGGACGAGACGTCTACTTGCTAACTAGTAGGATTTATTTTTCCAA
ATGGGGGATAGTTAGTTGGTGGTAGTATTCATTCGCAAGGGTGGGTATTCCATTGATTAGTGTGTACTCTGGATTGATA
GATTTTAGTAAAAACATAAACTTCTTAGAAGGACTACCTACCTCAAGTAGGGGGAAGTCGGACGCAATAACAGGTCT
GTGATGCCCTTAGATACCTTGGGCTGCACGCGCGTTACAATGCAGCATAGAAAAAGGCTCCGTTGCTGGAAAGCAAT
GGTAATCAAAAGAATATACTACGTAATAGGGATTGATCTTTGTAATTATCGATCATAAACGAGGAATTCCTTGTAAGCG
TAATTCATTACATTACGCTGAATATGTCCCTGCCCTTTGTACACACCGCCCGTCGCTCCTACCGATCGAATGATTAGGT
AAAACTGACAGATAAAAATTTTTGTAAAAAAATGTAGAAGTTATTTAAATCTCATTGTTTAGAGGAAGGAGAAGTCG
TAACAAGGTATCCGTAGGTGAACCTGCAGAAGGATCAA

>AM168114.1 *Acytostelium digitatum* OH517
AACCTGGTTGATCCTGCCAGTAGTCATATGCTTGTCTCAAAGATTAAGCCATGCATGTCTAAGTATAAACCTTTATACG
GTGAAACTGCGGACGGCTCATTACAACAGTGATAAACTAAAGAACTTTCGCGCGAAAGCGTTTTGGATAACCGCAG
TAAATCGGGGCTAATACATATAAACGAAAGGCGAATGGTTTTATTACCATAAGTTCTTGCGATTATTAGTCATTTTAAA
CCAACCTCTTCGGAGTTTGTGTTGAAACCGAATAATATTGCTGATCGAGAATTTATTCTCGACAAGTTCTATGTGTCA
CTGCCCTATCAACTTTGGATGGTACGGTATTGGCCTACCATGGTTGTAACGGGTGACGGGGAATCAGGGTTCGATTCC
GGAGAGGGAGCCTGAGAAATGGCTACCACTTCTACGGAAGGCAGCAGGCGCGCAAATTACTCAATCCCAATACGGG
GAAGTAGTGACAATAAATACTAATGTCTTTCCAAATTTTGGAGGACAATTGGAATAAGTACAATGTAAATAGCTTAAC
AAAAGTAATTGGAGGGCAAGTCTGGTGCCAGCAGCCGCGGTAATTCCAGCTCCAATAGCGTATACTAAATTTGTTGC
AGTTAAAAAGCTCGTAGTTAAGGTTGAAATCTTTCGGATTAAAAGAGTTACTCTTACCAAAGAGTTCTCCTTAGATC
ATTGCTTATCTAATAAGTTAATCGATCACCGAAAATTTTTTTAAGGTTACTTTATAGGCAACTATAATTTAAACCAATC
CACTGTGAGAAAATTGTAGTGTTTAAAGCAAGCTTTTATGCTTGTTCAATGCAGCATGGTATGGTAAAATAGGACACT
TAACATTTGTTGGTTTACCAATTGTTTAAGTGTAATGACTAATAGGGAAAGTCGGGGCCGTTCATATTGATGGGCGAG
AGGTGAAATTCGTTGACCCTATCAAGATGCACTACAGCGAAAGCATTCGGCAAGTATTTCTCCATTAATCAAGAACG
AAAGTTTGGGGATCGAAGACGATCAGATACCGTCGTAGTCCAAACCATAAACGATGTCGACCAGAGATTGGGTAGA
CAATAAATATAAATGCATTCAGAACCTTGTGAGAAATCATGAGTGTTTGGACTCTGGGGGGAGTATGGTCGCAAGGC
TGAAACTTAAAGGAATTGACGGAAGGGCACACAATGGAGTGGAACCTGCGGCTTAATTTGACTCAACTCGGGAAA
ACTTACCAAGCTCAGATATAATAAGGATTGACAGACTAAAAGATCTTTCATGATTGTATAAGTGGTGGTGCATGGTCG
TTCTTAGTTGGTGGAGTGATTTGTCTGGTCAATTCCGATAACGGACGAGACGTCTACTTACTAACTAGTGGGATTTAT
CTAGTCAATTTGGAGGATAAGTTTTTAGTGCTTCTATTCAAGTCTTTCGGGGTTTGGTAGTTCATTGAAGGCTGTGTA
CTCTGATTAGATAGATTCTAGTAAAAACATAAACTTCTTAGAAGGACTACCTACCTCAAGTGGGGGGAAGTTGGACG
CAATAACAGGTCTGTGATGCCCTTAGATACCTTGGGCTGCACGCGCGTTACAATGTAACAGAGAAAAAGGCTTTCCA
TGTCTGGAAAGACATGGTAATCAATTGAATCTACTACGTAATAGGGATTGATCTTTGTAATTATCGATCATCAACGAGG
AATTCCTTGTAAGCGTAAGTCATTACCTTACGCTGAATATGTCCCTGCCCTTTGTACACACCGCCCGTCGCTCCTACC
GATCGAACGATTAGGTAAAATTGACAGATTTGATTTAAAGCAGCAATGTTATTAAATCTTAGAAGTTATTTAAATCTCA
TTGTTTAGAGGAAGGAGAAGTCGTAACAAGGTATCCGTAGGTGAACCTGCAGAAGGATCAA

>HQ141512.1 *Acytostelium leptosomum* 212rjb
AACCTGGTTGATCCTGCCAGTAGTCATATGCTTGTCTCAAAGATTAAGCCATGCATGTCTAAGTATAAACTTTAATACG
GTGAACCTGCAGAAGGATCAATACAACATGTGATAAAGTACAGAACTTTCGCGCGTAAGCGTCTTTGGATAACCGCA
GTAAATCGGGGCTAATACATATAAACGAAAGATTAATAGTTTCTACTGTTGATTTTGCGATTGTTAGTCATTTCAAAAC
TAGCCTCTTCGGAGTTAGTGGTGAATCCGAACAATATTGCTGATCGAAATTTATTTCGACGAGTTCTATCTGTCACTG
CCCTATCAACTTTCGATGGTACGGTATTGGCCTACCATGGTTGTAACGGGTAACGGGGAATCAGGGTTCGATTCCGGA
GAGGGCGCCTGAGAAATGGCGACCACTTCTACGGAAGGCAGCAGGCGCGCAAATTACTCAATCCCAATACGGGGA
AGTAGTGACAATAAATACTAATTTCTTTCCATTTTATGGAGGAAAATTGGAATAAGTACAACGTAAATAGCTTAACCA
AAGTAATTGGAGGGCAAGTCTGGTGCCAGCAGCCGCGGTAATTCCAGCTCCAATAGCGTATACTAAATTTGTTGCAG
TTAAAAAGCTCGTAGTTCAGATAAAGGTTGTTCGGGTCAAGGTGTCTTTATAAAATAAGCATCTGATTCTATCTAATTT
ATTAGTTTAGTATCACCGACAACTTTATTTAGGGTCTGTTATCAATTTATTGGTGATTTGATCTATCCACTGTGAGAAA
ATTGTAGTGTTTAAAGCAAGCGTTTCTGCTTGTTCAATGCAGCATGGTATGGTAAAATAGGACACTTAACAATTGTTG
GTTTTTTGTTGAAGTGTAATGACTAATAGGGAAGGGCGGGATCGTACATATTGATGGGCGAGAGGTGAAATTCGTTG
ACCCTATCAAGATGCACTACAGCGAAAGCATTCGATAAGTACTTCTCCATTAATCAAGAACGAAAGTTTGGGGATCG
AAGACGATCAGATACCGTCGTAGTCCAAACTATAAACTATGTCGACCAGGGATCGGATAGATTAAATTTCTAACTCTA
TTCGGAACCTTGTGAGAAATCATGAGTGTTTGGACTCTGGGGGGAGTATGGTCGCAAGGCTGAAACTTAAAGGAAT
TGACGGAAGGGCACACAATGGAGTGGAGCCTGCGGCTTAATTTGACTCAACTCGGGAAAACTTACCAAGCTCAGAT
ATAATAAGGATTGACAGACTAAAAGATCTTTCATGATTGTATAAGTGGTGGTGCATGGTCGTTCTTAGTTGGTGGAGT
GATTTGTCTGGTCAATTCCGATAACGGACGAGACGTCTACTTACTAAATAGTAGGATTTATGGGTCAATATGTGGGAT
AGGTCTTAGGGGTTTGTATATATTTCCAAAAGATTTGTATGCTACCACTATGGCTGTGTAGCACGACTCTTTAAATTCG
AGTAAAAAACTAAACTTCTTAGAAGGACTACCCACCTCAAGTGGGGGGAAGTCGGACGCAATAACAGGTCTGTGAT
GCCCTTAGATACCTTGGGCTGCACGCGCGCTACAATGTAGAGATGAAAAAGGTTTCCGGGTACCGGAAGGTATCGGT
AATCATTTGAATACTCTACGTGATAGGGATTGATCTTTGTAATTATCGATCATCAACGAGGAATTCCTTGTAAGCGTAA
GTCATTACCTTACGCTGAATAGGTCCCTGCCCTTTGTACACACCGCCCGTCGCTCCTACCGATCGAACGATTAGGTAA
AACTGACGGACGGTGAACCTGCAGAAGGATCATTAGTCAACACTCGTGTTGATCCTGCTCAGTAGATACGATATGCT
TGTCTCCAAAGATAAGCCAGGCA

>AM168111.1 *Acytostelium leptosomum* FG12

AACCTGGTTGATCCTGCCAGTAGTCATATGCTTGTCTCAAAGATTAAGCCATGCATGTCTAAGTATAAACTTTTATACG
GTGAAACTGCAGACGGCTCATTACAACAGTGATAAACTACAGAACTTTCGAGCGTAAGCTTTTTGGATAACCGCAGT
AAATCGGGGCTAATACATATAAACGGAAGGCGATTGGGCAACCAAGAGTCCTTGCGATTACTAGCTTCAAACCAACC
TCTATGAGATTGAGTTGAAACCGAATAATATTGCTGATCAGAATTTAATTCTGACAAGTTCTATGTGTCACTGCCCTAT
CAACTTTCGATGGTACGGTATTGGCCTACCATGGTTGTAACGGGTAACGGGGAATCAGGGTTCGATTCCGGAGAGGG
AGCCTGAGAAATGGCTACCACTTCTACGGAAGGCAGCAGGCGCGCAAATTACTCAATCCCAATACGGGGAAGTAGT
GACAATAAATACTAATGTTCTTCCATTTTATGGAGAACAATTGGAATAAGTACAACGTAAATAGCTTCGCAAAAGTAA
TTGGAGGGCAAGTCTGGTGCCAGCAGCCGCGGTAATTCCAGCTCCAATAGCGTATACTAAATTTGTTGCAGTTAAAA
AGCTCGTAGTTTAGGCAGAGGTCATTCGGATTCATTAATTTCACCTTCAAAAGTGACTTTAAAGGCTATATCACGCTT
CGGTTGTGTTTATGGTCACCGATGACTTGTATTATGACGTTATTTGTTAGTTTACTAGCTTATAATCGTCAATCCACTGT
GAGAAAATTGTGGTGTTTAAAGCAGGCGTTCTAGCCTGATCAATGCAGCATGGTATGGTAAAATATGACACTTAACAT
TTGTTGGTTTACTTTGTTATAGTGTAATGACTAATAGGGAAGGGCGGGGCCGTTCATATTGATGGGCGAGAGGTGAA
ATTCGTTGACCCTATCAAGATGCACTACAGCGAAAGCATTCGGCAAGTACTTCTCCATTAATCAAGAACGAAAGTTT
GGGGATCGAAGCCGATCAGATACCGTCGTAGTCCAAACCATAAATGATGTCGATCAGGGATCGGTAGGATTTAATTG
TAAATTCTTATCGGAACCTTGTGAGAAATCATGAATGTTTGGACTCTGGGGGGAGTATGGTCGCAAGGCTGAAACTT
AAAGGAATTGACGGAAGGGCACACAATGGAGTGGAACCTGCGGCTTAATTTGACTCAACTCGGGAAAACTTACCA
AGCTCAGATATAATAAGGATTGACAGACTAAAAGATCTTTCATGATTGTATAAGTGGTGGTGCATGGTCGTTCTTAGT
TGGTGGAGTGATTTGTCTGGTCAATTCCGATAACGGACGAGACGTCTACTTGCTAACTAGTAGGATTTATTTTTCCAA
ATGGGGGATAGTTAGTTGGTGGTAGTATTCATTCGCAAGGGTGGGTATTCCATTGATTAGTGTGTACTCTGGATTGATA
GATTTTAGTAAAAACATAAACTTCTTAGAAGGACTACCTACCTCAAGTAGGGGGAAGTCGGACGCAATAACAGGTCT
GTGATGCCCTTAGATACCTTGGGCTGCACGCGCGTTACAATGCAGCATAGAAAAAGGCTCCGTTGCTGGAAAGCAAT
GGTAATCAAAAGAATATACTACGTAATAGGGATTGATCTTTGTAATTATCGATCATAAACGAGGAATTCCTTGTAAGCG
TAATTCATTACATTACGCTGAATATGTCCCTGCCCTTTGTACACACCGCCCGTCGCTCCTACCGATCGAATGATTAGGT
AAAACTGACAGATAAAAATTTTTGTAAAAAAATGTAGAAGTTATTTAAATCTCATTGTTTAGAGGAAGGAGAAGTCG
TAACAAGGTATCCGTAGGTGAACCTGCAGAAGGATCAA

>AM168109.1 *Acytostelium longisorophorum* DB10A
AACCTGGTTGATCCTGCCAGTAGTCATATGCTTGTCTCAAAGATTAAGCCATGCATGTCTAAGTATAAACTTTTATACG
GTGAAACTGCAGACGGCTCATTACAACAGTGATAAACTACAGAACTTTCGAGCGTCTTTTTGGATAACCGCAGTAAA
TCGGGGCTAATACATATAAACGGAAGGCGATTGAAGAGTCCTTGCGATTACTAGCTTAAACCAACCTGTTGAAACCG
AATAATATTGCTGATCAGAAATTCTGACAAGTTCTATGTGTCACTGCCCTATCAACTTGATGGTACGGTATTGGCCTAC
CATGGTTGTAACGGGTAACGGGGAATCAGGGTTCGATTCCGGAGAGGGAGCCTGAGAAATGGCTACCACTTCTACG
GAAGGCAGCAGGCGCGCAAATTACTCAATCCCAATACGGGGAATAGTGACAATAAATACTAATGTTCTTCCTTTATGG
AGAACAATTGGAATAAGTACAACGTAAATAGCTTAGCAAAAGTAATGGGAGGGCAAGTCTGGTGTCAATCCACTGT
GAGAAAATTGTGGTGTTTAAAGCAGGCGTTCAGTCTGATCAATGCAGCATGGTATGGTAAAATATGACACTTCATTTG
TTGGTTAGTGTAATGACTAATAGGGAAGGGCGGGGCCGTTCATATTGATGGGCGAGAGGTGAAATTCGTTGACCCTA
TCAAGATGCACTACAGCGAAAGCATTCGGCAAGTACTTCTCCATTAATCAAGAACGAAAGTTTGGGGTTCGAAGAC
GATCAGATACCGTCGTAGTCCAAACCATAAATGATGTCGATCAGGGATCGGTAGTAAATTCTTATCGGAACCTTGTGA
GAAATCATGAATGTTTGGACTCTGGGGGGAGTATGGTCGCAAGGCTGAAACTTAAAGGAATTGACGGAAGGGCACA
CAATGGAGTGGAACCTGCGGCTTAATTTGACTCAACTCGGGAAAACTTACCAAGCTCAGATATAATAAGGATTGACA
GACTAAAAGATCTTTCATGATTGTATAAGTGGTGGTGCATGGTCGTTCTTAGTTGGTGGAGTGATTTGTCTGGTCAAT
TCCGATAACGGACGAGACGTCTACTTGCTAACTAGTAGGATTTATTTTTCCAAATGGGGGATAGTTAGTTGGTGGTGT
GGGTATTCCATTGATTAGTGTGTACTCTGGATTGATAGATCTTCTTAGAAGGACTACCTACCTCAAGTAGGGGGAAAT
CGGACGCAATAACAGGTCTGTGATGCCCTTAGATACCTTGGGCTGCACGCGCGCTACAATGCAGCATAGAAAAAGG
CCGTTGCTGGAAAGCAATGGTAATCAAAAATATACTACGTAATAGGGATTGATCTTTGTAATTATCGATCATAAACGA
GGAATTCCTTGTAAGCGTAATTCATTACATTACGCTGAATATGTCCCTGCCCTTTGTACACACCGCCCGTCGCTCCTAC
CGATCGAATGATTGGGTAAAACTGACAGATTAGAAGTTATTTAAATCTCATTGTTTAGAGGAAGGAGAAGTCGTAAC
AAGGTATCCGTAGG

>HQ141513.1 *Acytostelium magnisorum* 08A
AACCTGGTTGATCCTGCCAGTAGTCATATGCTTGTCTCAAAGATTAAGCCATGCATGTCTAAGTATAAACCTCTATATG
GTGAACCTGCAGAAGGATCAACTACGAGCATGTGATAAACTACAAGACTTTCGCGCTTCGGCGTTATGGATAACCGC
AGTAAATCGGGGCTAATACATATAAACGAAAGACGAGCAAGCAATTGCGAGTTCTTGCGATTTTTAGCTACTAAAAT
CGACCCTTTTAGGTCGTGTTGAAACCGAAAAATATTGCTGATCGAGAATTTCTCGACAAGTCTTATGTGTCACTGCCC
TATCAACTCGATGGTACGGTATTGGCCTACCATGGTTGCAACGGGTAACGGGGAATTAGGGTTCGATTCCGGAGAGG
GAGCCTGAGAAATGGCTGCCACTTATACGGAAGGCAGCAGGCGCGCAAATTACTCAATTCCGATACGGAGAAGTAG
TGACAATAAATACAAATGCTTTTCAATTTTTTTGAGAGCAATTTGAATAAGTACAACTTAAATCGCTTAGCTAAAGTG
ATTGGAGGGCAAGTCTGGTGCCAGCAGCCGCGGTAATTCCAGCTCCAATAGCGTATACTAAATTTGTTGCAGTTAAA
ACGCTCGTAGTTAAAGTAAAGGTTACTTGGATAAACAAGAATAATTTTTTTTAGAATTATTTAACGAATTTTTAATTAT
TTTCATAGTTAAATTTTCACCAAGTTCCTTATTTTTGGACTTAAAAATTTATTTTTTTGTCTAGTCTACTGTGAGAAAAT
TGTAGTGTTTAAAGCAAGCTATCAGCTTGATCAATGCAGCATGGTATAGAAAAATATGACACTAAGTATTTTGTTGGT
TTAATACTCTAGTGTAATGATTAATAGGGAATGGCGGGGCCGTTCGTATTGATGGGCGAGAGGTGAAATTCGTTGACC
CTATCAAGACGTACAACAGCGAAAGCATTCGGCAAGTATTTCTCCATTAATCAAGAACGAAAGTTTGGGGATCGAAG
ACGATCAGATACCGTCGTAGTCCAAACCATAAACAATGTCGACCAGGGATTGGACGGAATTTTTTTAAAAAACTCGC
TCAGAACCTTGTGAGAAATCATGAGTGTTTGGACTCTGGGGGGAGTATGGTCGCAAGTCTGAAACTTAAAGGAATT
GACGGAAGGGCACACAATGGAGTGGAACCTGCGGCTTAATTTGACTCAACTCGGGAAAACTTACCAAGCTCAGATA
TAATAAGGATTGACAGACTAAAAGATCTTTCATGATTGTATAAGTGGTGGTGCATGGTCGTTCTTAGTTGGTGGAGTG
ATTTGTCTGGTCAATTCCGATAACGGACGAGACGTCTACTTGCTAACTAGTAAAATTAATTTTTTCGCTCGGGGATAG
TTAGTTTGTATTGATCTTTATCATCGCAATGTGGTAATTTTCGTATAGATTGATGTGTACCTTGGAAAAATAAATTTTTT
CATAAACATTAAACTTCTTAGAATGACTACCTACCTCAAGTGGGGGGAAGTCGGACGCAATAACAGGTCTGTGATGC
CCTTAGATACCTTGGGCTGCACGCGCGTTACAATGTATAAAAGAAAAAGGCTTCCCGGAGCCGTAAGGCTCTGGTAA
TCATATGAATTTTCTACGTAATAGGGATTGATCTTTGTAATTATCGATCATAAACGAGGAATTCCTTGTAAGCGTAAATC
ATTACTTTACGCTGAATATGTCCCTGCCCTTTGTACACACCGCCCGTCGCTCCTACCGATCGAACGATTAGGTAAAAT
TGACGGACGGTGAACCTGCAGAAGGATCATTTGACTAACCATGAGTTGATCCTGCCAGTAGTCGATATGCTTGTCTC
AAAGATAAGCCATGCAGTGTAAG

>AM168113.1 *Acytostelium serpentarium* SAB3A
AACCTGGTTGATCCTGCCAGTAGTCATATGCTTGTCTCAAAGATTAAGCCATGCATGTCTAAGTATAAACTTTAATACG
GTGAAACTGCGGACGGCTCATTACAACAGTGATAAAGTACAGAACTTTCGCGCGTAAGCGTCTTTGGATAACCGCA
GTAAATCGGGGCTAATACATATAAACGAAAGATTAATAGTTTCTACTGTTGATTTTGCGATTGTTAGTCATTTCAAAAC
TAGCCTCTTCGGAGTTAGTGGTGAATCCGAACAATATTGCTGATCGAAATTTATTTCGACGAGTTCTATCTGTCACTG
CCCTATCAACTTTCGATGGTACGGTATTGGCCTACCATGGTTGTAACGGGTAACGGGGAATCAGGGTTCGATTCCGGA
GAGGGCGCCTGAGAAATGGCGACCACTTCTACGGAAGGCAGCAGGCGCGCAAATTACTCAATCCCAATACGGGGA
AGTAGTGACAATAAATACTAATTTCTTTCCATTTTATGGAGGAAAATTGGAATAAGTACAACGTAAATAGCTTAACCA
AAGTAATTGGAGGGCAAGTCTGGTGCCAGCAGCCGCGGTAATTCCAGCTCCAATAGCGTATACTAAATTTGTTGCAG
TTAAAAAGCTCGTAGTTCAGATAAAGGTTGTTCGGGTCAAGGTGTCTTTATAAAATAAGCATCTGATTCTATCTAATTT
ATTAGTCTAGTATCACCGACAACTTTATTTAGGGTCTGTTATCAATTTATTGGTGATTTGATCTATCCACTGTGAGAAA
ATTGTAGTGTTTAAAGCAAGCGTTTCTGCTTGTTCAATGCAGCATGGTATGGTAAAATAGGACACTTAACAATTGTTG
GTTTTTTGTTGAAGTGTAATGACTAATAGGGAAGGGCGGGATCGTACATATTGATGGGCGAGAGGTGAAATTCGTTG
ACCCTATCAAGATGCACTACAGCGAAAGCATTCGATAAGTACTTCTCCATTAATCAAGAACGAAAGTTTGGGGATCG
AAGACGATCAGATACCGTCGTAGTCCAAACTATAAACTATGTCGACCAGGGATCGGATAGATTAAATTTCTAACTCTA
TTCGGAACCTTGTGAGAAATCATGAGTGTTTGGACTCTGGGGGGAGTATGGTCGCAAGGCTGAAACTTAAAGGAAT
TGACGGAAGGGCACACAATGGAGTGGAGCCTGCGGCTTAATTTGACTCAACTCGGGAAAACTTACCAAGCTCAGAT
ATAATAAGGATTGACAGACTAAAAGATCTTTCATGATTGTATAAGTGGTGGTGCATGGTCGTTCTTAGTTGGTGGAGT
GATTTGTCTGGTCAATTCCGATAACGGACGAGACGTCTACTTACTAAATAGTAGGATTTATGGGTCAATATGTGGGAT
AGGTCTTAGGGGTTTGTATATATTTCCAAAAGATTTGTATGCTACCACTATGGCTGTGTAGCACGACTCTTTAAATTCG
AGTAAAAAACTAAACTTCTTAGAAGGACTACCCACCTCAAGTGGGGGGAAGTCGGACGCAATAACAGGTCTGTGAT
GCCCTTAGATACCTTGGGCTGCACGCGCGCTACAATGTAGAGATGAAAAAGGTTTCCGGGTACCGGAAGGTATCGGT
AATCATTTGAATACTCTACGTGATAGGGATTGATCTTTGTAATTATCGATCATCAACGAGGAATTCCTTGTAAGCGTAA
GTCATTACCTTACGCTGAATAGGTCCCTGCCCTTTGTACACACCGCCCGTCGCTCCTACCGATCGAACGATTAGGTAA
AACTGACGGACTTAGACCTACCTTTTGGCATGGTCGGGGAAGTTATTTAAATCTCATTGTTTAGAGGAAGGAGAAGT
CGTAACAAGGTATCCGTAGGTGAACCTGCAGAAGGATCAA

>HQ141514.1 *Acytostelium singulare* FDIB
AACCTGGTTGATCCTGCCAGTAGTCATATGCTTGTCTCAAAGATTAAGCCATGCATGTCTAAGTATAAACTTTTATACG
GTGAAACTGCAGACGGATCAATACAACAGTGATAAACTACAGAACTTTCGAGCGTAAGCTTTTTGGATAACCGCAG
TAAATCGGGGCTAATACATATAAACGGAAGGCGATTGGGCAACCAAGAGTCCTTGCGATTACTAGCTTCAAACCAAC
CTCTATGAGATTGAGTTGAAACCGAATAATATTGCTGATCAGAATTTAATTCTGACAAGTTCTATGTGTCACTGCCCTA
TCAACTTTCGATGGTACGGTATTGGCCTACCATGGTTGTAACGGGTAACGGGGAATCAGGGTTCGATTCCGGAGAGG
GAGCCTGAGAAATGGCTACCACTTCTACGGAAGGCAGCAGGCGCGCAAATTACTCAATCCCAATACGGGGAAGTAG
TGACAATAAATACTAATGTTCTTCCATTTTATGGAGAACAATTGGAATAAGTACAACGTAAATAGCTTAGCAAAAGTA
ATTGGAGGGCAAGTCTGGTGCCAGCAGCCGCGGTAATTCCAGCTCCAATAGCGTATACTAAATTTGTTGCAGTTAAA
AAGCTCGTAGTTTAGGCAGAGGTCATTCGGATTCATTAATTTCACCTTCAAAAGTGACTTTAAAGGCTATATCACGCT
TCGGTTGTGTTTATGGTCACCGATGACTTGTATTATGACGTTATTTGTTAGTTTACTAGCTTATAATCGTCAATCCACTG
TGAGAAAATTGTGGTGTTTAAAGCAGGCGTTCTAGTCTGATCAATGCAGCATGGTATGGTAAAATATGACACTTAAC
ATTTGTTGGTTTACTTTGTTATAGTGTAATGACTAATAGGGAAGGGCGGGGCCGTTCATATTGATGGGCGAGAGGTGA
AATTCGTTGACCCTATCAAGATGCACTACAGCGAAAGCATTCGGCAAGTACTTCTCCATTAATCAAGAACGAAAGTT
TGGGGATCGAAGACGATCAGATACCGTCGTAGTCCAAACCATAAATGATGTCGATCAGGGATCGGTAGGATTTAATT
GTAAATTCTTATCGGAACCTTGTGAGAAATCATGAATGTTTGGACTCTGGGGGGAGTATGGTCGCAAGGCTGAAACT
TAAAGGAATTGACGGAAGGGCACACAATGGAGTGGAACCTGCGGCTTAATTTGACTCAACTCGGGAAAACTTACCA
AGCTCAGATATAATAAGGATTGACAGACTAAAAGATCTTTCATGATTGTATAAGTGGTGGTGCATGGTCGTTCTTAGT
TGGTGGAGTGATTTGTCTGGTCAATTCCGATAACGGACGAGACGTCTACTTGCTAACTAGTAGGATTTATTTTTCCAA
ATGGGGGATAGTTAGTTGGTGGTAGTATTCATTCGCAAGGGTGGGTATTCCATTGATTAGTGTGTACTCTGGATTGATA
GATTTTAGTAAAAACATAAACTTCTTAGAAGGACTACCTACCTCAAGTAGGGGGAAGTCGGACGCAATAACAGGTCT
GTGATGCCCTTAGATACCTTGGGCTGCACGCGCGTTACAATGCAGCATAGAAAAAGGCTTCGTTGCTGGAAAGCAAT
GGTAATCAAAAGAATATACTACGTAATAGGGATTGATCTTTGTAATTATCGATCATAAACGAGGAATTCCTTGTAAGCG
TAATTCATTACATTACGCTGAATATGTCCCTGCCCTTTGTACACACCGCCCGTCGCTCCTACCGATCGAATGATTAGGT
AAAACTGACAGATGGTGAACCTGCAGAAGGATCATAACATTGTGTTGTATACACATAGACACTAGGTAGATACGATA
TGTCTAGTCTCAATA

>AM168110.1 *Acytostelium subglobosum* LB1
AACCTGGTTGATCCTGCCAGTAGTCATATGCTTGTCTCAAAGATTAAGCCATGCATGTCTAAGTATAAACCTCTATATG
GTGAAACTGCAGACGGCTCATTACAACAGTGATAAACTACAAGACTTTCGCGCTTCGGCGTTATGGATAACCGCAGT
AAATCGGGGCTAATACATATAAACGAAAGACGAGCAAGCAATTGCGAGTTCTTGCGATTTTTAGCTACTAAAATCGA
CCCTTTTAGGTCGTGTTGAAACCGAAAAATATTGCTGATCGAGAATTTCTCGACAAGTCTTATGTGTCACTGCCCTAT
CAACTCGATGGTACGGTATTGGCCTACCATGGTTGCAACGGGTAACGGGGAATTAGGGTTCGATTCCGGAGAGGGA
GCCTGAGAAATGGCTGCCACTTCTACGGAAGGCAGCAGGCGCGCAAATTACTCAATTCCGATACGGAGAAGTAGTG
ACAATAAATACAAATGCTTTTCAATTTTTTTGAGAGCAATTTGAATAAGTACAACTTAAATCGCTTAGCTAAAGTGA
TTGGAGGGCAAGTCTGGTGCCAGCAGCCGCGGTAATTCCAGCTCCAATAGCGTATACTAAATTTGTTGCAGTTAAAA
CGCTCGTAGTTAAAGTAAAGGTTACTTGGATAAACAAGAATAATTTTTTTTAGAATTATTTAACGAATTTTTAATTATTT
TCATAGTTAAATTTTCACCAAGTTCCTTATTTTTGGACTTAAAAATTTATTTTTTTGTCTAGTCTACTGTGAGAAAATTG
TAGTGTTTAAAGCAAGCTATCAGCTTGATCAATGCAGCATGGTATAGAAAAATATGACACTAAGTATTTTGTTGGTTT
AATACTCTAGTGTAATGATTAATAGGGAATGGCGGGGCCGTTCGTATTGATGGGCGAGAGGTGAAATTCGTTGACCCT
ATCAAGACGTACAACAGCGAAAGCATTCGGCAAGTATTTCTCCATTAATCAAGAACGAAAGTTTGGGGATCGAAGA
CGATCAGATACCGTCGTAGTCCAAACCATAAACAATGTCGACCAGGGATTGGACGGAATTTTTTTAAAAAACTCGCT
CAGAACCTTGTGAGAAATCATGAGTGTTTGGACTCTGGGGGGAGTATGGTCGCAAGTCTGAAACTTAAAGGAATTG
ACGGAAGGGCACACAATGGAGTGGAACCTGCGGCTTAATTTGACTCAACTCGGGAAAACTTACCAAGCTCAGATAT
AATAAGGATTGACAGACTAAAAGATCTTTCATGATTGTATAAGTGGTGGTGCATGGTCGTTCTTAGTTGGTGGAGTGA
TTTGTCTGGTCAATTCCGATAACGGACGAGACGTCTACTTGCTAACTAGTAAAATTAATTTTTTCGCTCGGGGATAGT
TAGTTTGTATTGATCTTTATCATCGCAAGGTGGTAATTTTCGTATAGATTGATGTGTACCTTGGAAAAATAAATTTTTTC

ATAAACATTAAACTTCTTAGAAGGACTACCTACCTCAAGTGGGGGGAAGTCGGACGCAATAACAGGTCTGTGATGCC
CTTAGATACCTTGGGCTGCACGCGCGTTACAATGTAGAAAAGAAAAAGGCTTCCCGGAGCCGTAAGGCTCTGGTAA
TCATATGAATTTTCTACGTAATAGGGATTGATCTTTGTAATTATCGATCATAAACGAGGAATTCCTTGTAAGCGTAAATC
ATTACTTTACGCTGAATATGTCCCTGCCCTTTGTACACACCGCCCGTCGCTCCTACCGATCGAACGATTAGGTAAAAT
TGACGGACTTTGACATACTAGAAATAGCTTGTTTTTGAAAGTTATTTAAATCTCATTGTTTAGAGGAAGGAGAAGTCG
TAACAAGGTATCCGTAGGTGAACCTGCAGAAGGATCAA

>AM168112.1 *Rostrostelium ellipticum* AE2
AACCTGGTTGATCCTGCCAGTAGTCATATGCTTGTCTCAAAGATTAAGCCATGCATGTCTAAGTATAAACCTTTATACG
GTGAAACTGCAGACGGCTCATTACAACAGTGATAAACTAATGGACTTTCGCGCCTTTAAAAGCGTCTTGGATATCCG
CAGTAAATCGGGGCTAATACATGCAAACGAAAGGGTGAGCGGGTAACCGCGAGCCTTTGCAATTATTTAGCTTTTG
CTACCAACCTCTTAGGAGTTTGTGTTGAATCCGAATAATTTTGCTGATCGGAATTTTATTCCGACAAGTTCAAAGTGT
CACTGCCCTATCAACTTTCGATGGTACGGTATTGGCCTACCATGGTTGTAACGGGTAACGGGGAATCAGGGTTCGGTT
CCGGAGAGGGCGCCTGAGAAATGGCGACCACTTCTACGGAAGGCAGCAGGCGCGCAAATTACTCAATCCCAATACG
GGGAAGTAGTGACAATAAATACTGATGCCTTTCCATTTTATGGAGGGTAATTAGAATAAGTACAATTTAAATCGCTTA
GCCAAAGTGATTGGAGGGCAAGTCTGGTGCCAGCAGCCGCGGTAATTCCAGCTCCAATAGCATATACTAAATTTGTT
GCAGTTAAAAAGCTCGTAGTTTAGAATGAAATTTTTCGAGGGATTTTGATTTTACAATCTGAACCCTGTCTTAAATTG
CCTTACGGGTTAATTTTTGACTGTCTCGATAATTTCAATTTTGCCTTTAGGCATTTATGTCTATTAGGTAGGTCCACTGT
GAGAAAATTGTGGTGTTTAAAGCGGACGTTTTTGTTTGTTCATTGCAGCATGGTATGGTAAAACAAGACACTGTTCA
CTCGTTGGTTGTGAATCAGTGTAATGACTAATAGGGAAGGACGGGGCCGTTCATATTGATGGGCGAGAGGTGAAATT
CGTTGACCCTATCAAGATGCACTACAGCGAAAGCATTCGGCAAGTACTTCCCCATTAATCAAGAACGAAAGTTTGGG
GATCGAAGACGATCAGATACCGTCGTAGTCCAAACCATAAACTATGTCGACCAGGGACTGGACGGATGATTTTTAAA
AAACTCGCTCAGAACCTTGTGGGAAACCATGAGTGTTTGGATTCCGGGGGGAGTATGGTCGCAAGTCTGAAACTTA
AAGGAATTGACGGAAGGGCACACAATGGAGTGGAACCTGCGGCTTAATTTGACTCAACTCGGGAAAACTTACCAA
GCTCAGATATAGTAAGGATTGACAGACTAAAAGATTTTTCATGATTCTATAAGTGGTGGTGCATGGTCGTTCTTAGTT
GGTGGAGTGATTTGTCTGGTCAATTCCGATAACGGACGAGACCTCTACCTACTAAATAGTGGTATTTATTTTGTCGTTA
TGGGGGATAGTTAGTTGGCGAGAGTAGTAGGTTTTCGGATTTATTATTTCTCGTTGATTGGTGTGTACCTCGATAAGAT
AGATTACTTCTAAAAATAACTTCTTAGAGGGACTACCCACCTCAAGTGGGGGGAAGTCGGAGGCAATAACAGGTCT
GTGATGCCCTTAGATACCTTGGGCTGCACGCGCGTTACAATGAAAGCGTGAAAAAGGCTCCCAGCACCGGAGGGTG
TTGGAAATCATATGAATCACTTTCGTAATGGGGATTGACCTTTGTAATTATCGGTCATAAACGAGGAATTCCTTGTAAG
CGCAAGTCATTACCTTGCGTTGAATATGTCCCTGCCCTTTGTACACACCGCCCGTCGCTCCTACCGATCGAACGATTA
GGTAAAACTGACAGATTGTTTTGTAAGGTAGCAATATTTTACAGAATGAAAAGTTATTTAAATCTCATTGTTTAGAGG
AAGGAGAAGTCGTAACAAGGTATCAGTAGGTGAACCTGCAGAAGGATCAA

>AM168104.1 *Heterostelium album* PN500
AACCTGGTTGATCCTGCCAGTAGTCATATGCTTGTCTCAAAGATTAAGCCATGCATGTCTAAGTATAACCTTTATACGG
TGAAACTGCAGACGGCTCATTACAACAGTGATAAACTAAAGAACTTTCGCGCTTCGGCGTCTTGGATAACCGCAGTA
AATCGGGGCTAATACATGTAAACGAGAGGATGAGCAGGTAACTGCGAGTCTTTGCGATTGTTAGCTTTCATTCACAC
CAACCTCTTCGGAGTTTGTGGTGAGTCCGAACAATATTGCTGATCGGAAACTTGTTTCCGACGAGTTCTTTGTGTCA
CTGCCCTATCAACTTTCGATGGTAAGGTATTGGCTTACCATGGTTGTAACGGGTGACGGGGAATCAGGGTTCGATTCC
GGAGAGGGAGCCTGAGAAATGGCTACCACTTCTACGGAAGGCAGCAGGCGCGCAAATTACTCAATCCCAATACGGG
GAAGTAGTGACAAAAAATACTAATGCCTTTCCATTTATATGGGGGGCAATTGGAATAAGTACAACTTAAATCGCTTAG
CAAAAGTGATTGGAGGGCAAGTCTGGTGCCAGCAGCCGCGGTAATTCCAGCTCCAATAGCGTATACTAATTTTGTTG
CAGTTAAAAAGCTCGTAGTTGAGATTGAGATTTCTTAGGTTTAGAGTCGCTTGGCCTTCGGGTTTGAGTTTGATTCGT
AAAGCTCTATAGTAGAATTCCATTTTATTATTAGGGTTACTAAGAAATTTCTATCTGCCCATGGTAGCTAGCAATAGTTA
TCAATCGGGTGATCTACTGTGAGAAAATTGTAGTGTTCAAAGCAGGCGTCTTACGTTTGTTCAATGCAGCATGGTATA
GTAAAATATGACACTAAATATATGTTGGTTGTATATTCTTAGTGTAATGACTAATAGGGAAGGGCGGGGCCGTTCATAT
TGATGGGCGAGAGGTGAAATTCGTAGACCCTATCAAGATGCACTACAGCGAAAGCATTCGGCAAGTACTTCTCCATT
AATCAAGAACGAAAGTTTGGGGATCAAAGACGATCAGATACCGTCGTAGTCCAAACCATAAACTATGTCGACCAGG
GATTGGACGGATAATTTTTTAAAAACTCGCTCAGAACCTTGTGAGAAATCATGAGTGTTTGGACTCTGGGGGGAGTA
TGGTCGCAAGTCTGAAACTTAAAGGAATTGACGGAAGGGCACACAATGGAGTGGAACCTGCGGCTTAATTTGACTC
AACTCGGGAAAACTTACCAAGCTCAGATATAATAAGGATTGACAGACTAAAGGATCTTTCATGATTGTATAAGTGGT
GGTGCATGGTCGTTCTTAGTTGGTGGAGTGATTTGTCTGGTCAATTCCGATAACGGACGAGACCTCTACTTACTAACT
AGTGGTATTTATTTGGTCACTATGGGAGATAGTCATTTGGTGTTGGTAGTTAGGCCGTTAAAAGTTTAGCTATTCTTCA
TTGAGTGGTGTGTATTCTGATCAGATAGGTACTAATTTAAAAAATAAACTTCTTAGAAGGACTACCTACCTCAAGTGG
GGGGAAGTCGGAGGCAATAACAGGTCTGTGATGCCCTTAGATACCTTGGGCTGCACGCGCGTTACAATGTAGGCGA
GAAAAAGGTTTCCGGAATCGAAAGGTTTCGGTAATCATTTGAATTGCCTACGTAATGGGGATTAATTTTTGTAATTAT
CGATTATCAACGAGGAATTCCTTGTAAGCGTAAATCATTACTTTACGCTGAATATGTCCCTGCCCTTTGTACACACCGC
CCGTCGCTCCTACCGATCGAACGATTAGGTAAAACTGACGGACTGGATGACTTTTTCGCAAGGATTTGTCGTTTGGG
AAGTTAGTTAAATCTCATTGTTTAGAGGAAGGAGAAGTCGTAACAAGGTATCCGTAGGTGAACCTGCAGAAGGATC
AA

>KP167480.1 *Heterostelium ampliverticillatum*
AACCTGGTTGATCCTGCCAGTAGTCATATGCTTGTCTCAAAGATTAAGCCATGCATGTCCAAGTATAACCTTTCTACG
GTGAACCTGCAGAAGGATCAAGTACAACATGTAGATAAACTAAAGAACTTTCGCGCTTCGGCGTTCTGGATAACCG
CAGTAAATCGGGGCTAATACATGTAAACGAGAGGATGAGCGGGCAACTGCGAGTCTTTGCGATTGTTAGCTTTTATT
CACACCAACCTCTTCGGAGTTTGTGGTGAGTCCGAACAATATTGCTGATCGGAAACTTGTTTCCGACGAGTTCTTTG
TGTCACTGCCCTATCAACTTTCGATGGTAAGGTATTGGCTTACCATGGTTGTAACGGGTGACGGGGAATCAGGGTTC
GATTCCGGAGAGGGAGCCTGAGAAATGGCTACCACTTCTATGGAAGGCAGCAGGCGCGCAAATTACTCAATCCCAA
TACGGGGAAGTAGTGACAAAAAATACTAATGCCTTTCCATTTATATGGGGGGCAATTGGAATAAGTACAATTTAAATC
GCTTAGCAAAAGTGATTGGAGGGCAAGTCTGGTGCCAGCAGCCGCGGTAATTCCAGCTCCAATAGCGTATACTAAAT
TTGTTGCAGTTAAAAAGCTCGTAGTTGAGATAGAGATTTCTCGGGTTTAGCGGTCGTTATTGCCTTCACGGGTTAATA
ATTTGATTCGTAAAGCTTTATAGTAAGATTCCATCTTATTATTAAAGTTACCGAGGAATTTCCAACTGCCCATGGTAAC
TGGTAACAGTTATCAATCGGGTGATCTACTGTGAGAAAATTGTAGTGTTCAAAGCAGGCGTTTACGTTTGTTCAATGC
AGCATGGTATAGTAAAATATGACACTAAATATATGTTGGTTGTATATTCTTAGTGTAATGACTAATAGGGAAGGGCGGG

GCCGTTCATATTGATGGGCGAGAGGTGAAATTCGTTGACCCTATCAAGATGCACTACAGCGAAAGCATTCGGCAAGT
ACTTCTCCATTAATCAAGAACGAAAGTTTGGGGATCGAAGACGATCAGATACCGTCGTAGTCCAAACCATAAACTAT
GTCGACCAGGGATTGGACGGATAATTTTTAAAAACTCGCTCAGAACCTTGTGAGAAATCATGAGTGTTTGGACTCT
GGGGGGAGTATGGTCGCAAGTCTGAAACTTAAAGGAATTGACGGAAGGGCACACAATGGAGTGGAACCTGCGGCT
TAATTTGACTCAACTCGGGAAAACTTACCAAGCTCAGATATAATAAGGATTGACAGACTAAAAGATCTTTCATGATTG
TATAAGTGGTGGTGCATGGTCGTTCTTAGTTGGTGGAGTGATTTGTCTGGTCAATTCCGATAACGGACGAGACCTCTA
CCTACTAACTAGTGGTATTTATTTGGTCATTATGGGAGATAGTCATTTGGTGTTGGTAGTAGGCCGTCAAAAGTTTACT
ATTCTTCATTGAGTGGTGTGTATCCTGATCAGATAGGTACTATCTAAAAAAATAAACTTCTTAGAGGGACTACCTACCT
CAAGTGGGGGGAAGTCGGAGGCAATAACAGGTCTGTGATGCCCTTAGATACCTTGGGCTGCACGCGCGTTACAATG
TAGACGAGAAAAAGGCTTCCGACATCGAAAGGTGCCGGTAATCAATTGAATTGTCTACGTAATGGGGATTAATTTTT
GTAATTATCGATTATCAACGAGGAATTCCTTGTAAGCGTAAATCATTACTTTACGCTGAATATGTCCCTGCCCTTTGTA
CACACCGCCCGTCGCTCCTACCGATCGAACGATTAGGTAAAACTGACGGACTGAACGACTGTAACCTGGTTGATCCT
GCCAGTAGTCATATGCTTGTCTCAAAGATAAGCCATGCA

>AM168096.1 *Heterostelium anisocaule* NZ47B
AACCTGGTTGATCCTGCCAGTAGTCATATGCTTGTCTCAAAGATTAAGCCATGCATGTCTAAGTATAACCTTTATACGG
TGAAACTGCAGACGGCTCATTACAACAGTGATAAACTAAAGAACTTCCGCGCTTCGGCGTTTTGGATAACCGCAGTA
AATCGGGGCTAATACATGTAAACGAGAGGATGAACGGGCAACTGTGAGTCTTTGCGATTGTTAGCTTTAATCACCAA
CCTCTTCGGAGTCGGTGGTGAATCCCTACAATATTGCTGATCGGAAACTTGTTTCCGACGAGTTCTTTGTGTCACTGC
CCTATCAACTTTCGATGGTAAGGTATTGGCTTACCATGGTTGTAACGGGTGACGGGGAATCAGGGTTCGATTCCGGA
GAGGGAGCCTGAGAAATGGCTACCACTTCTACGGAAGGCAGCAGGCGCGCAAATTACCCAATCCCAATACGGGGAA
GTAGTGACAAAAAATACTAATGCCTTTCCATTTATATGGGGGGCAATTGGAATAAGTACAATTTAAATCGCTTAACGA
AAGTGATTGGAGGGCAAGTCTGGTGCCAGCAGCCGCGGTAATTCCAGCTCCAATAGCGTATACTAAATTTGTTGCAG
TTAAAAAGCTCGTAGTTGAGATTGAGATTTCTCAGGTTTAGCGGTCAACATTGCCTTCACGGGTTAATGTTTGATACG
TAAAGCTTTATAGTAGAATTTCATTTTATTATTAAAGTTACTGAGGAATTTCCAACTGCCCATGGTAGCCAGTAATGGT
TATCAATAGGGTGATCTACTGTGAGAAAATTGTAGTGTTCAAAGCAGGCGTCTTTCGTTTGTTCAATGCAGCATGGTA
TAGTTAAATATGACACTAGATATATGTTGGTTGTATATTGTTAGTGTAATGACTAATAGGGAAGGGCGGGGCCGTTCAT
ATTGATGGGCGAGAGGTGAAATTCGTTGACCCTATCAAGATGCACTACAGCGAAAGCATTCGGCAAGTACTTCTCCA
TTAATCAAGAACGAAAGTTTGGGGATCGAAGACGATCAGATACCGTCGTAGTCCAAACCATAAACTATGTCGACCAG
GGATTGGACGGATAATTTTTAAAAGCTCGCTCAGAACCTTGTGAGAAATCATGAGTGTTTGGACTCTGGGGGGAGT
ATGGTCGCAAGTCTGAAACTTAAAGGAATTGACGGAAGGGCACACAATGGAGTGGAACCTGCGGCTTAATTTGACT
CAACTCGGGAAAACTTACCAAGCTCAGATATAATAAGGATTGACAGACTAAAAGATCTTTCATGATTGTATAAGTGGT
GGTGCATGGTCGTTCTTAGTTGGTGGAGTGATTTGTCTGGTCAATTCCGATAACGGACGAGACCTCTACTTACTAACT
AGTGATATTTATTTGGTCAATATGGGAGATAGTCATTTGGTGTTGGTAGTAGGACGTCAAAATCTTACTATTCTTCATT
GAGTGGTGTGTATTCTGATCAGATAGGTACTAATTTTAAAAATAAACTTCTTAGAAGGACTACCTACCTCAAGTGGGG
GGAAGTCGGAGGCAATAACAGGTCTGTGATGCCCTTAGATACCTTGGGCTGCACGCGCGTTACAATGTAGGCGAGA
AAAAGGTTTCCGGAATCGAAAGGTTTCGGTAATCAATTGAATTGCCTACGTAATGGGGATTAATTTTTGTAATTATCG
ATTATCAACGAGGAATTCCTTGTAAGCGTAAATCATTACTTTACGCTGAATATGTCCCTGCCCTTTGTACACACCGCCC
GTCGCTCCTACCGATCGAACGATTAGGTAAAACTGACGGACTAGGCGACTCTTCCGCAAGGATTTGTTGTTTGGGAA
GTTAGTTAAATCTCATTGTTTAGAGGAAGGAGAAGTCGTAACAAGGTATCCGTAGGTGAACCTGCAGAAGGATCAA

>AM168102.1 *Heterostelium arachnoideum* YA1
AACCTGGTTGATCCTGCCAGTAGTCATATGCTTGTCTCAAAGATTAAGCCATGCATGTCTAAGTATAACCTTTATACGG
TGAAACTGCAGACGGCTCATTACAACAGTGATAAACTAAAGAACTTTCGCGCTTCGGCGTCTTGGATAACCGCAGTA
AATCGGGGCTAATACATGTAAACGAGAGGATGAGCGGGCAACTGCGAGTCTTTGCGATTGTTAGCTTTTATTCACAC
CAACCTCTTCGGAGTTTGTGGTGAGTCCGAACAATATTGCTGATCGGAAATTTATTTCCGACGAGTTCTTTGTGTCAC
TGCCCTATCAACTTTCGATGGTAAGGTATTGGCTTACCATGGTTGTAACGGGTGACGGGGAATCAGGGTTCGATTCCG
GAGAGGGAGCCTGAGAAATGGCTACCACTTCTATGGAAGGCAGCAGGCGCGCAAATTACTCAATCCCAATACGGGG
AAGTAGTGACAAAAAATACTAATGCCTTTCCATTTATATGGGGGGCAATTGGAATAAGTACAACTTAAATCGCTTAGC
AAAAGTGATTGGAGGGCAAGTCTGGTGCCAGCAGCCGCGGTAATTCCAGCTCCAATAGCGTATACTAAATTTGTTGC
AGTTAAAAAGCTCGTAGTTGAGATTGAGATTTCTTGGGTTTAGCATGGACTATGGCCTCACGGTTTATAATTTATGTC
AAGCTTTATAGTAAGATTTTACTCTTATTATTTAAGTTACCAAGAAATTTCCAACTGCCCATGCCAGTTAGCAATAATT
GGCTGATCGGGTGATCTACTGTGAGAAAATTGTAGTGTTCAAAGCAGGCGTCTTTCGCTTGTTCAATGCAGCATGGT
ATAGTAAAATATGACACTAAACATATGTTGGTTGTATGTATTAGTGTAATGACTAATAGGGAAGGGCGGGGCCGTTCA
TATTGATGGGCGAGAGGTGAAATTCGTTGACCCTATCAAGATGCACTACAGCGAAAGCATTCGGCAAGTACTTCTCC
ATTAATCAAGAACGAAAGTTTGGGGATCGAAGACGATCAGATACCGTCGTAGTCCAAACCATAAACTATGTCGACCA
GGGATTGGACGGATAATTTTTAAAAACTCGCTCAGAACCTTGTGAGAAATCATGAGTGTTTGGACTCTGGGGGGAG
TATGGTCGCAAGTCTGAAACTTAAAGGAATTGACGGAAGGGCACACAATGGAGTGGAACCTGCGGCTTAATTTGAC
TCAACTCGGGAAAACTTACCAAGCTCAGATATAATAAGGATTGACAGACTAAAAGATCTTTCATGATTGTATAAGTGG
TGGTGCATGGTCGTTCTTAGTTGGTGGAGTGATTTGTCTGGTCAATTCCGATAACGGACGAGACCTCTACCTGCTAAC
TAGTGGTATTTATTTGGTCAAATGGGAGATAGTCATTTGGTGTTGGCAGTTAGGCCGTTAAAAGTTTAATTGTCCTTC
ATTGAGTGGTGTGTATCCTGATCAGATAGGTACTAACTAAAAAAATAAACTTCTTAGAGGGACTACCTACCTCAAGTG
GGGGGAAGTCGGAGGCAATAACAGGTCTGTGATGCCCTTAGATACCTTGGGCTGCACGCGCGTTACAATGTAGGCG
AGAAAAAGGTTTCCGACATCGAAAGGTGCCGGTAATCAATTGAATTGCCTACGTAATGGGGATTAATTTTTGTAATTA
TCGATTATCAACGAGGAATTCCTTGTAAGCGTAAATCATTACTTTGCGCTGAATATGTCCCTGCCCTTTGTACACACCG
CCCGTCGCTCCTACCGATCGAACGATTAGGTAAAACTGACAGACTGGACGACTTCTCGCAAGGGATTGTTGTTTGGA
AAGTTAGTTAAATCTCATTGTTTAGAGGAAGGAGAAGTCGTAACAAGGTATCCGTAGGTGAACCTGCAGAAGGATC
AA

>AM168097.1 *Heterostelium asymetricum* OH567
AACCTGGTTGATCCTGCCAGTAGTCATATGCTTGTCTCAAAGATTAAGCCATGCATGTCTAAGTATAACCTTTATACGG
TGAAACTGCAGACGGCTCATTACAACAGTGATAAACTAAAGAACTTTCGCGCTTCGGCGTCTTGGATAACCGCAGTA
AATCGGGGCTAATACATGTAAACGAGAGGATGAGCAGGCAACTGCGAGTCTTTGCGATTGTTAGCTATTCATTCACA
CCAACCTCTTCGGAGTTTGTGGTGAGTCCGAACAATATTGCTGATCGGAAACTTGTTTCCGACGAGTTCTTTGTGTC
ACTGCCCTATCAACTTTCGATGGTAAGGTATTGGCTTACCATGGTTGTAACGGGTGACGGGGAATCAGGGTTCGATTC

CGGAGAGGGAGCCTGAGAAATGGCTACCACTTCTACGGAAGGCAGCAGGCGCGCAAATTACTCAATCCCAATACGG
GGAAGTAGTGACAAAAAATACTAATGCCTTTCCATTTATATGGGGGGCAATTGGAATAAGTACAACTTAAATCGCT
TAACGAAAGTGATTGGAGGGCAAGTCTGGTGCCAGCAGCCGCGGTAATTCCAGCTCCAATAGCGTATACTAAATTTG
TTGCAGTTAAAAAGCTCGTAGTTGAGATTGAGATTTCTTGGGTTTAGCGTCCAAGATTGCCTTCACGGGTTAATCTTT
TGGTACGTAAAGCTTTTCAGTGGAATTCTATTTCATTGTTAAAGTTACCAAGGAATTTCTATCTGCCCATTTAAGGTGG
CAACACTTTAAATTTAGGGTGATCTACTGTGAGAAAATTGTAGTGTTCAAAGCAGGCGTTTTCGCTTGTTCAATGCA
GCATGGTATAGTAAAATATGACACTAAATATATGTTGGTTGTATATTTTAGTGTAATGACTAATAGGGAAGGGCGGGGC
CGTTCATATTGATGGGCGAGAGGTGAAATTCGTTGACCCTATCAAGATGCACTACAGCGAAAGCATTCGGCAAGTAC
TTCTCCATTAATCAAGAACGAAAGTTTGGGGATCGAAGACGATCAGATACCGTCGTAGTCCAAACCATAAACTATGT
CGACCAGGGATTGGACGGATAATTTTTTAAAAACTCGCTCAGAACCTTGTGAGAAATCATGAGTGTTTGGACTCTGG
GGGGAGTATGGTCGCAAGTCTGAAACTTAAAGGAATTGACGGAAGGGCACACAATGGAGTGGAACCTGCGGCTTA
ATTTGACTCAACTCGGGAAAACTTACCAAGCTCAGATATAATAAGGATTGACAGACTAAAAGATCTTTCATGATTGTA
TAAGTGGTGGTGCATGGTCGTTCTTAGTTGGTGGAGTGATTTGTCTGGTCAATTCCGATAACGGACGAGACCTCTAC
TTACTAACTAGTGGTATTTATTTGGTCAATATGGGAGATAGCTATTTGGTGTTGGTGTTAGGCCGTCAAAAGTTTAGCA
TTCTTCATTGAGTGGTGTGTATTCTGATCAGATAGGTACTAAAATTTTAAATAAACTTCTTAGAAGGACTACCTACCTC
AAGTGGGGGGAAGTCGGAGGCAATAACAGGTCTGTGATGCCCTTAGATACCTTGGGCTGCACGCGCGTTACAATGT
AGGCGAGAAAAAGGTTTCCGGAACCGAAAGGTTTCGGTAATCATTTGAATTGCCTACGTAATGGGGATTAATTTTTG
TAATTATCGATTATCAACGAGGAATTCCTTGTAAGCGTAAATCATTACTTTACGCTGAATATGTCCCTGCCCTTTGTAC
ACACCGCCCGTCGCTCCTACCGATCGAACGATTAGGTAAAACTGACGGACTAGGCGACTTTCTCGCAAGGGATTGTT
GTTTGGGAAGTTAGTTAAATCTCATTGTTTAGAGGAAGGAGAAGTCGTAACAAGGTATCCGTAGGTGAACCTGCAA
AGGATCAA

>HQ141503.1 *Heterostelium asymetricum* HN20C
AACCTGGTTGATCCTGCCAGTAGTCATATGCTTGTCTCAAAGATTAAGCCATGCATGTCTAAGTATAACCTTTATACGG
TGAACCTGCAGAAGGATCAACTACGGCATGTAGATAAACTAAAGAACTTTCGCGCTTCCGGGTCTTGGATAACCGCA
GTAAATCGGGGCTAATACATGTAAACGAGAGGATGAGCAGGCAACTGCGAGTCTTTGCGATTGTTAGCTATTCATTC
ACACCAACCTCTTCGGAGTTTGTGGTGAGTCCGAACAATATTGCTGATCGGAAACTTGTTTCCGACGAGTTCTTTGT
GTCACTGCCCTATCAACTTTCGATGGTAAGGTATTGGCTTACCATGGTTGTAACGGGTGACGGGGAATCAGGGTTCG
ATTCCGGAGAGGGAGCCTGAGAAATGGCTACCACTTCTACGGAAGGCAGCAGGCGCGCAAATTACTCAATCCCAAT
ACGGGGAAGTAGTGACAAAAAATACTAATGCCTTTCCATTTATATGGGGGGCAATTGGAATAAGTACAACTTAAATC
GCTTAACGAAAGTGATTGGAGGGCAAGTCTGGTGCCAGCAGCCGCGGTAATTCCAGCTCCAATAGCGTATACTAAAT
TTGTTGCAGTTAAAAAGCTCGTAGTTGAGATTGAGATTTCTTGGGTTTAGCGTCCAAGATTGCCTTCACGGGTTAATC
TTTTGGTACGTAAAGCTTTTCAGTGGAATTCTATTTCATTGTTAAAGTTACCAAGGAATTTCTATCTGCCCATTTAAGG
TGGCAACACTTTAAATTTAGGGTGATCTACTGTGAGAAAATTGTAGTGTTCAAAGCAGGCGTTTTCGCTTGTTCAAT
GCAGCATGGTATAGTAAAATATGACACTAAATATATGTTGGTTGTATATTTTAGTGTAATGACTAATAGGGAAGGGCGG
GGCCGTTCATATTGATGGGCGAGAGGTGAAATTCGTTGACCCTATCAAGATGCACTACAGCGAAAGCATTCGGCAAG
TACTTCTCCATTAATCAAGAACGAAAGTTTGGGGATCGAAGACGATCAGATACCGTCGTAGTCCAAACCATAAACTA
TGTCGACCAGGGATTGGACGGATAATTTTTTAAAAACTCGCTCAGAACCTTGTGAGAAATCATGAGTGTTTGGACTC
TGGGGGGAGTATGGTCGCAAGTCTGAAACTTAAAGGAATTGACGGAAGGGCACACAATGGAGTGGAACCTGCGGC
TTAATTTGACTCAACTCGGGAAAACTTACCAAGCTCAGATATAATAAGGATTGACAGACTAAAAGATCTTTCATGATT
GTATAAGTGGTGGTGCATGGTCGTTCTTAGTTGGTGGAGTGATTTGTCTGGTCAATTCCGATAACGGACGAGACCTCT
ACTTACTAACTAGTGGTATTTATTTGGTCAATATGGGAGATAGCTATTTGGTGTTGGTGTTAGGCCGTCAAAAGTTTAG
CATTCTTCATTGAGTGGTGTGTATTCTGATCAGATAGGTACTAAAATTTTAAATAAACTTCTTAGAAGGACTACCTACC
TCAAGTGGGGGGAAGTCGGAGGCAATAACAGGTCTGTGATGCCCTTAGATACCTTGGGCTGCACGCGCGTTACAAT
GTAGGCGAGAAAAAGGTTTCCGGAACCGAAAGGTTTCGGTAATCATTTGAATTGCCTACGTAATGGGGATTAATTTT
TGTAATTATCGATTATCAACGAGGAATTCCTTGTAAGCGTAAATCATTACTTTACGCTGAATATGTCCCTGCCCTTTGT
ACACACCGCCCGTCGCTCCTACCGATCGAACGATTAGGTAAAACTGACGGACTAGGCGACTTTCAACCTGGTTGATC
CTGCGGTGAACCTGCAGAAGGATCACAGTAGTCATATGCTTGTCTCAAAGATTAAGCCATG

>HQ141508.1 *Heterostelium australicum* NB1AP
AACCTGGTTGATCCTGCCAGTAGTCATATGCTTGTCTCAAAGATTAAGCCATGCATGTCTAAGTATAACCTTTATACGG
TGAACCTGCAGAAGGATCAACTACGACTATGTGATAAACTAAAGAACTTTCGCGCTTCGGCGTCTTGGATAACCGCA
GTAAATCGGGGCTAATACATGTAAACGAGAGGATGAGCGGGCAACTGCGAGTCTTTGCGATTGTTAGCTTTTATTCA
CACCAACCTCTTCGGAGTTTGTGGTGAGTCCGAACAATATTGCTGATCGGAAATTTATTTCCGACGAGTTCTTTGTGT
CACTGCCCTATCAACTTTCGATGGTAAGGTATTGGCTTACCATGGTTGTAACGGGTGACGGGGAATCAGGGTTCGATT
CCGGAGAGGGAGCCTGAGAAATGGCTACCACTTCTATGGAAGGCAGCAGGCGCGCAAATTACTCAATCCCAATACG
GGGAAGTAGTGACAAAAAATACTAATGCCTTTCCATTTATATGGGGGGCAATTGGAATAAGTACAACTTAAATCGCTT
AGCAAAAGTGATTGGAGGGCAAGTCTGGTGCCAGCAGCCGCGGTAATTCCAGCTCCAATAGCGTATACTAAATTTGT
TGCAGTTAAAAAGCTCGTAGTTGAGATTGAGATTTCTTGGGTTTAGCATTGATTATGACTTTCGGGTTTATAATTTATG
TCAAGCTTTATAATAAGATTTCACTCTTGTTATTTAAGTTACCAAGAAATTTCCAACTGCCCATGCCAGTTAGCAATAA
TTGGCTGATCGGGTGATCTACTGTGAGAAAATTGTAGTGTTCAAAGCAGGCGTCTTTCGCTTGTTCAATGCAGCATG
GTATAGTAAAATATGACACTAAACATATGTTGGTTGTATGTATTAGTGTAATGACTAATAGGGAAGGGCGGGGCCGTT
CATATTGATGGGCGAGAGGTGAAATTCGTTGACCCTATCAAGATGCACTACAGCGAAAGCATTCGGCAAGTACTTCT
CCATTAATCAAGAACGAAAGTTTGGGGATCGAAGACGATCAGATACCGTCGTAGTCCAAACCATAAACTATGTCGAC
CAGGGATTGGACGGATAATTTTTTAAAAACTCGCTCAGAACCTTGTGAGAAATCATGAGTGTTTGGACTCTGGGGGG
AGTATGGTCGCAAGTCTGAAACTTAAAGGAATTGACGGAAGGGCACACAATGGAGTGGAACCTGCGGCTTAATTTG
ACTCAACTCGGGAAAACTTACCAAGCTCAGATATAATAAGGATTGACAGACTAAAAGATCTTTCATGATTGTATAAGT
GGTGGTGCATGGTCGTTCTTAGTTGGTGGAGTGATTTGTCTGGTCAATTCCGATAACGGACGAGACCTCTACCTGCT
AACTAGTGGTATTTATTTGGTCAATACGGGAGATAGTCATTTGGTGTTGGCAGTTAGACCCTTAAAAGTTTAATTGTC
CTTCATTGAGTGGTGTGTATCCTGATCAGATAGGTACTAACTAAAAAATAAACTTCTTAGAGGGACTACCTACCTCAA
GTGGGGGGAAGTCGGAGGCAATAACAGGTCTGTGATGCCCTTAGATACCTTGGGCTGCACGCGCGTTACAATGTAG
ACGAGAAAAAGGTTTCCGACATCGAAAGGTGCCGGTAATCAATTGAATTGTCTACGTAATGGGGATTAATTTTTGTA
ATTATCGATTATCAACGAGGAATTCCTTGTAAGCGTAAATCATTACTTTGCGCTGAATATGTCCCTGCCCTTTGTACAC
ACCGCCCGTCGCTCCTACCGATCGAACGATTAGGTAAAACTGACAGACTGGACGACTTCTAACCTGGTTGATCCTGG
TGAACCTGCAGAAGGATCAGCCAGTAGTCATATGCTGTCTCAAAGATTAAGCCATGC

>HQ141499.1 *Heterostelium boreale* BSB10A
AACCTGGTTGATCCTGCCAGTAGTCATATGCTTGTCTCAAAGATTAAGCCATGCATGTCTAAGTATAAACCTTTATACG
GTGAACCTGCAGAAGGATCAACTACAACTATGTGATAAACTAAAGAACTTTCGCGCTTCGGCGTCTTGGATAACCGC
AGTAAATCGGGGCTAATACATATAAACGAAAGGATGAACGGGCAACTGTGAGTCTTTGCGATTGTTAGTTTTCTTAC
ACCAACCTCTTCGGAGTTTGTGGTGAGTCCGAACAATATTGCTGATCGAAACTTAGTTTCGACGAGTTCTTTGTGTC
ACTGCCCTATCAACTTTCGATGGTAAGGTATTGGCTTACCATGGTTGTAACGGGTGACGGGGAATCAGGGTTCGATTC
CGGAGAGGGAGCCTGAGAAATGGCTACCACTTCTACGGAAGGCAGCAGGCGCGCAAATTACTCAATCCCAATACGG
GGAAGTAGTGACAAAAAATACTAATGCCCTTCCATTTTATGGGGGGCAATTGGAATAAGTACAACTTAAATCGCTT
AGCAAAAGTGATTGGAGGGCAAGTCTGGTGCCAGCAGCCGCGGTAATTCCAGCTCCAATAGCGTATACTAAATTTGT
TGCAGTTAAAAAGCTCGTAGTTGAGATTGAGATTTCTTAGGTTTAAAGCTTCTATTTTTAGCTTCGGTTAAATTTTAGT
TGTGTATAAAGCTTTTTAGTAGACTTTTGTTTGCTATTAAAGTTACTAAGAAATTTCTTTCTGCCCATGATTTAGGTTC
GCCCCTGAATCAATCGGGTGTTCTACTGTGAGAAAATTGTAGTGTTTAAAGCAGGCGTATTAGTTTGTTCAATGCAGC
ATGGTATAGTAAAACAAGACACTAAACATTTGTTGGTTAATGTTCTATAGTGTAATGACTAATAGGGAAGGGCGGGGC
CGTTCATATTGATGGGCGAGAGGTGAAATTCGTTGACCCTATCAAGATGCACTACAGCGAAAGCATTCGGCAAGTAC
TTCTCCATTAATCAAGAACGAAAGTTTGGGGATCGAAGACGATCAGATACCGTCGTAGTCCAAACCATAAACTATGT
CGACCAGGGACTGGACGGTTAATTTTATAAAAACTCATTCAGAACCTTGTGGGAAACCATGAGTGTTTGGACTCTGG
GGGGAGTATGGTCGCAAGTCTGAAACTTAAAGGAATTGACGGAAGGGCACACAATGGAGTGGAACCTGCGGCTTA
ATTTGACTCAACTCGGGAAAACTTACCAAGCTCAGATATAATAAGGATTGACAGACTAAAAGATCTTTCATGATTGTA
TAAGTGGTGGTGCATGGTCGTTCTTAGTTGGTGGAGTGATTTGTCTGGTCAATTCCGATAACGGACGAGACCTCTAC
CTACTAACTAGTGGTACTTATTTGGTCATTATGGGAGATAGTTAGTTGGTGTTGGTAGTTAGATTTCGGTTTAATTATTC
TTCATTGATCTAGTGTGTATCCTGGTCGGATAAGTACTAACTAAAAAAATAAAACTTCTTAGAGGGACTACCTACCTA
AGTGGGGGGAAGTCGGAGGCAATAACAGGTCTGTGATGCCCTTAGATACCTTGGGCTGCACGCGCGTTACAATGTA
AGTTGGAAAAAGGTTTCCGGTATCGAAAGGTATCGGTAATCATTTGAATAGCTTACGTAATGGGGATTAATCTTTGTA
ATTATCGGTTATCAACGAGGAATTCCTTGTAAGCGTAAATCATTACTTTACGCTGAATATGTCCCTGCCCTTTGTACAC
ACCGCCCGTCGCTCCTACCGATCGAACGATTAGGTAAAACTGACGGACTATGTGATTTTAGGTGAACCTGCAGAAG
GATCA

>HQ141498.1 *Heterostelium candidum* bsb6b
AACCTGGTTGATCCTGCCAGTAGTCATATGCTTGTCTCAAAGATTAAGCCATGCATGTCTAAGTATAACCTTTATACGG
TGAACCTGCAGAAGGATCAACCAGGTTTAGATAAACTAAAGAACTTTCGCGCTTCGGCGTCTTGGATAACCGCAGT
AAATCGGGGCTAATACATGTAAACGAGAGGATGAGCGGGTAACCGCGAGTCTTTGCGATTGTTAGCTTTTACACACA
CCAACCTCTTCGGAGATTGTGGTGAGTCCGAACAATATTGCTGATCGGAAATTTATTTCCGACGAGTTCTTTGTGTCA
CTGCCCTATCAACTTTCGATGGTAAGGTATTGGCTTACCATGGTTGTAACGGGTGACGGGGAATCAGGGTTCGATTCC
GGAGAGGGAGCCTGAGAAATGGCTACCACTTCTATGGAAGGCAGCAGGCGCGCAAATTACTCAATCCCAATACGGG
GAAGTAGTGACAAAAAATACTAATGCCTTACCATTTATATGGGGGGCAATTGGAATAAGTACAACCTAAATCGCTTAG
CAAAAGTGATTGGAGGGCAAGTCTGGTGCCAGCAGCCGCGGTAATTCCAGCTCCAATAGCGTATACTAAATTTGTTG
CAGTTAAAAAGCTCGTAGTTGAGATTGAGATTTCTTGGGTTTAGCGTTCATTATTGCCTTCGGGTAATAACCGTTCGT
AAAGCTTCTAATCGGGATTCGTTTCGTTTAGGAGTTACCAAGAAATTTCTATATGCCCATGTCAACTGGTAACAGTTG
TCTGATCGGGTGATCTACTGTGAGAAAATTGTAGTGTTCAAAGCAGGCGTCTTTCGCTTGTTCAATGCAGCATGGTAT
AGTAAAATAAGACACTAAATATATGTTGGTTGTATATTCTAGTGTAATGACTAATAGGGAAGGGCGGGGCCGTTCATAT
TGATGGGCGAGAGGTGAAATTCGTTGACCCTATCAAGATGCACTACAGCGAAAGCATTCGGCAAGTACTTCTCCATT
AATCAAGAACGAAAGTTTGGGGATCGAAGACGATCAGATACCGTCGTAGTCCAAACCATAAACTATGTCGACCAGG
GATTGGACGGATAATTTTTTAAAAACTCGCTCAGAACCTTGTGAGAAATCATGAGTGTTTGGACTCTGGGGGGAGTA
TGGTCGCAAGTCTGAAACTTAAAGGAATTGACGGAAGGGCACACAATGGAGTGGAACCTGCGGCTTAATTTGACTC
AACTCGGGAAAACTTACCAAGCTCAGATATAATAAGGATTGACAGACTAAAAGATCTTTCATGATTGTATAAGTGGT
GGTGCATGGTCGTTCTTAGTTGGTGGAGTGATTTGTCTGGTCAATTCCGATAACGGACGAGACCTCTACCTACTAAAT
AGTGGTATTTATTTGGTCAACATGGAAGATAGTCATTTGGTGTTGGCGTTAGGTGTCAAAGCTTAGCGTTCTTCATTG
AGTGGTGTGTATTTCGATCAGATAGGTACTAACTAAAAAATAAACTTCTTAGAGGGACTACCTACCTCAAGTGGGGG
GAAGTCGGAGGCAATAACAGGTCTGTGATGCCCTTAGATACCTTGGGCTGCACGCGCGTTACAATGTAGACTAGAAA
AAGGTTTCCGACATCGAAAGGTGCCGGTAATCAATTGAATAGTCTACGTAATGGGGATTAATTTTGTAATTATCGATT
ATCAACGAGGAATTCCTTGTAAGCGTAAATCATTACTTTACGCTGAATATGTCCCTGCCCTTTGTACACACCGCCCGT
CGCTCCTACCGATCGAACGATTAGGTAAAACTGACGGACTGAATGAACTCTTAACCTGGTTGATCCGGTGAACCTGC
AGAAGGATCATGCCCTAGTAGTCATATGCTTGTCTCAAAGATAAGCCCATGCATTCTAAGTAA

>AY040337.1 *Heterostelium candidum*
TCATATGCTTGTCTCAAAGATAAGCCATGCATGTCTAAGTATAACCTTTATACGGTGAAACTGCAGACGGCTCATTAC
AACAGTGATAAACTAAAGAACTTTCGCGCTTCGGCGTCTTGGATAACCGCAGTAAATCGGGGCTAATACATGTAAAC
GAGAGGATGAGCGGGTAACCGCGAGTCTTTGCGATTGTTAGCTTTTACACACACCAACCTCTTCGGAGATTGTGGTG
AGTCCGAACAATATTGCTGATCGGAAATTTATTTCCGACGAGTTCTTTGTGTCACTGCCCTATCAACTTTCGATGGTA
AGGTATTGGCTTACCATGGTTGTAACGGGTGACGGGGAATCAGGGTTCGATTCCGGAGAGGGAGCCTGAGAAATGG
CTACCACTTCTATGGAAGGCAGCAGGCGCGCAAATTACTCAATCCCAATACGGGGAAGTAGTGACAAAAAATACTA
ATGCCTTACCATTTATATGGGGGGCAATTGGAATAAGTACAACCTAAATCGCTTAGCAAAAGTGATTGGAGGGCAAG
TCTGGTGCCAGCAGCCGCGGTAATTCCAGCTCCAATAGCGTATACTAAATTTGTTGCAGTTAAAAAGCTCGTAGTTGA
GATTGAGATTTCTTGGGTTTAGCGTTCATTATTGCCTTCGGGTAATAACCGTTCGTAAAGCTTCTAATCGGGATTCGTT
TCGTTTAGGAGTTACCAAGAAATTTCTATATGCCCATGTCAACTGGTAACAGTTGTCTGATCGGGTGATCTACTGTGA
GAAAATTGTAGTGTTCAAAGCAGGCGTCTTTCGCTTGTTCAATGCAGCATGGTATAGTAAAATAAGACACTAAATATA
TGTTGGTTGTATATTCTAGTGTAATGACTAATAGGGAAGGGCGGGGCCGTTCATATTGATGGGCGAGAGGTGAAATTC
GTTGACCCTATCAAGATGCACTACAGCGAAAGCATTCGGCAAGTACTTCTCCATTAATCAAGAACGAAAGTTTGGGG
ATCGAAGACGATCAGATACCGTCGTAGTCCAAACCATAAACTATGTCGACCAGGGATTGGACGGATAATTTTTTAAA
AACTCGCTCAGAACCTTGTGAGAAATCATGAGTGTTTGGACTCTGGGGGGAGTATGGTCGCAAGTCTGAAACTTAA
AGGAATTGACGGAAGGGCACACAATGGAGTGGAACCTGCGGCTTAATTTGACTCAACTCGGGAAAACTTACCAAG
CTCAGATATAATAAGGATTGACAGACTAAAAGATCTTTCATGATTGTATAAGTGGTGGTGCATGGTCGTTCTTAGTTG
GTGGAGTGATTTGTCTGGTCAATTCCGATAACGGACGAGACCTCTACCTACTAAATAGTGGTATTTATTTGGTCAACA
TGGAAGATAGTCATTTGGTGTTGGCGTTAGGTGTCAAAGCTTAGCGTTCTTCATTGAGTGGTGTGTATTTCGATCAGA

TAGGTACTAACTAAAAAATAAACTTCTTAGAGGGACTACCTACCTCAAGTGGGGGGAAGTCGGAGGCAATAACAGG
TCTGTGATGCCCTTAGATACCTTGGGCTGCACGCGCGTTACAATGTAGACTAGAAAAAGGTTTCCGACATCGAAAG
GTGCCGGTAATCAATTGAATAGTCTACGTAATGGGGATTAATTTTTGTAATTATCGATTATCAACGAGGAATTCCTTGT
AAGCGTAAATCATTACTTTACGCTGAATATGTCCCTGCCCTTTGTACACACCGCCCGTCGCTCCTACCGATCGAACGA
TTAGGTAAAACTGACGGACTGAATGACTTCTCGCAAGGGATTGTCGTTTGGAAAGTTAGTTAAATCTCATTGTTTAG
AGGAAGGAGAAGTCGTAACAAGGTATCCGTAGGTGAACCTGCGGATGGATCATTTC

>HQ141505.1 *Heterostelium colligatum* HN13C1
AACCTGGTTGATCCTGCCAGTAGTCATATGCTTGTCTCAAAGATTAAGCCATGCATGTCTAAGTATAACCTTTATACGG
TGAACCTGCAGAAGGATCAACTACAGAGCATGTGATAAACTAAAGAACTTTCGCGCTTCGGCGTCTTGGATAACCG
CAGTAAATCGGGGCTAATACATGTAAACGAGAGGATGAGCGGGCAACTGCGAGTCTTTGCGATTGTTAGCTATTCAC
ACACCAACCTCTTCGGAGTTTGTGGTGAGTCCGAACAATATTGCTGATCGGAAATTTATTTCCGACAAGTTCTTTGTG
TCACTGCCCTATCAACTTTCGATGGTAAGGTATTGGCTTACCATGGTTGTAACGGGTGACGGGGAATCAGGGTTCGAT
TCCGGAGAGGGAGCCTGAGAAATGGCTACCACTTCTATGGAAGGCAGCAGGCGCGCAAATTACTCAATCCCAATAC
GGGGAAGTAGTGACAAAAAATACTAATGCCTTTCCATTTATATGGGGGGCAATTGGAATAAGTACAACTTAAATCGCT
TAGCAAAAGTGATTGGAGGGCAAGTCTGGTGCCAGCAGCCGCGGTAATTCCAGCTCCAATAGCGTATACTAAATTTG
TTGCAGTTAAAAAGCTCGTAGTTGAGATTGAGATTTCTTGGGTTTAGCAGGCATTGTCGCCTTCGGGTTGGCAATCG
TTTGTAAAGCGCTATAATGGAATTTCACTTTCATTATATGTGTTACCAAGAAATTTCCAACTGCCCATGTCAAGTGGCA
ACACTTGTCTGATAGGGTGATCTACTGTGAGAAAATTGTAGTGTTCAAAGCAGGCGTCTTACGTTTGTTCAATGCAG
CATGGTATAGTAAAATATGACACTAAATATATGTTGGTTGTATATTCTTAGTGTAATGACTAATAGGGAAGGGCGGGGC
CGTTCATATTGATGGGCGAGAGGTGAAATTCGTTGACCCTATCAAGATGCACTACAGCGAAAGCATTCGGCAAGTAC
TTCTCCATTAATCAAGAACGAAAGTTTGGGGATCGAAGACGATCAGATACCGTCGTAGTCCAAACCATAAACTATGT
CGACCAGGGATTGGACGGATAATTTTTTAAAAACTCGCTCAGAACCTTGTGAGAAATCATGAGTGTTTGGACTCTGG
GGGGAGTATGGTCGCAAGTCTGAAACTTAAAGGAATTGACGGAAGGGCACACAATGGAGTGGAACCTGCGGCTTA
ATTTGACTCAACTCGGGAAAACTTACCAAGCTCAGATATAATAAGGATTGACAGACTAAAAGATCTTTCATGATTGTA
TAAGTGGTGGTGCATGGTCGTTCTTAGTTGGTGGAGTGATTTGTCTGGTCAATTCCGATAACGGACGAGACCTCTAC
CTGCTAACTAGTGGTATTTATTTGGTCAATATGGGAGATAGCTATTTGGTGTTGGCAGTCAGGGTCAAACTTGATTGT
CCTTCATTGAGTGGTGTGTATTCTGATCAGATAGGTACTAACTAAAAAATAAACTTCTTAGAGGGACTACCTACCTCA
AGTGGGGGGAAGTCGGAGGCAATAACAGGTCTGTGATGCCCTTAGATACCTTGGGCTGCACGCGCGTTACAATGTA
GACGAGAAAAAGGTCTCCGGCACCGAAAGGTGTCGGTAATCATATGAATTGTCTACGTAATGGGGATTAATTTTTGT
AATTATCGATTATCAACGAGGAATTCCTTGTAAGCGTAAATCATTACTTTGCGCTGAATATGTCCCTGCCCTTTGTACA
CACCGCCCGTCGCTCCTACCGATCGAACGATTAGGTAAAACTGACAGACTGAGTGACTTCTAACCATGGTTGATCCT
GGTGAACCTGCAGAAGGATCAGCCAGTAGTCATATGCTTGTCTCAAAGATTAAGCCATGCAGTCTAAG

>AM168098.1 *Heterostelium colligatum* OH538
AACCTGGTTGATCCTGCCAGTAGTCATATGCTTGTCTCAAAGATTAAGCCATGCATGTCTAAGTATAACCTTTATACGG
TGAAACTGCAGACGGCTCATTACAACAGTGATAAACTAAAGAACTTTCGCGCTTCGGCGTCTTGGATAACCGCAGTA
AATCGGGGCTAATACATGTAAACGAGAGGATGAGCGGGCAACTGCGAGTCTTTGCGATTGTTAGCTATTCACACACC
AACCTCTTCGGAGTTTGTGGTGAGTCCGAACAATATTGCTGATCGGAAATTTATTTCCGACAAGTTCTTTGTGTCACT
GCCCTATCAACTTTCGATGGTAAGGTATTGGCTTACCATGGTTGTAACGGGTGACGGGGAATCAGGGTTCGATTCCG
GAGAGGGAGCCTGAGAAATGGCTACCACTTCTATGGAAGGCAGCAGGCGCGCAAATTACTCAATCCCAATACGGGG
AAGTAGTGACAAAAAATACTAATGCCTTTCCATTTATATGGGGGGCAATTGGAATAAGTACAACTTAAATCGCTTAGC
AAAAGTGATTGGAGGGCAAGTCTGGTGCCAGCAGCCGCGGTAGTTCCAGCTCCAATAGCGTATACTAAATTTGTTGC
AGTTAAAAAGCTCGTAGTTGAGATTGAGATTTCTTGGGTTTAGCAGGCATTGTCGCCTTCGGGTTGGCAATCGTTTG
TAAAGCGCTATAATGGAATTTCACTTTCATTATATGTGTTACCAAGAAATTTCCAACTGCCCATGTCAAGTGGCAACA
CTTGTCTGATAGGGTGATCTACTGTGAGAAAATTGTAGTGTTCAAAGCAGGCGTCTTACGTTTGTTCAATGCAGCATG
GTATAGTAAAATATGACACTAAATATATGTTGGTTGTATATTCTTAGTGTAATGACTAATAGGGAAGGGCGGGGCCGTT
CATATTGATGGGCGAGAGGTGAAATTCGTTGACCCTATCAAGATGCACTACAGCGAAAGCATTCGGCAAGTACTTCT
CCATTAATCAAGAACGAAAGTTTGGGGATCGAAGACGATCAGATACCGTCGTAGTCCAAACCATAAACTATGTCGAC
CAGGGATTGGACGGATAATTTTTTAAAAACTCGCTCAGAACCTTGTGAGAAATCATGAGTGTTTGGACTCTGGGGGG
AGTATGGTCGCAAGTCTGAAACTTAAAGGAATTGACGGAAGGGCACACAATGGAGTGGAACCTGCGGCTTAATTTG
ACTCAACTCGGGAAAACTTACCAAGCTCAGATATAATAAGGATTGACAGACTAAAAGATCTTTCATGATTGTATAAGT
GGTGGTGCATGGTCGTTCTTAGTTGGTGGAGTGATTTGTCTGGTCAATTCCGATAACGGACGAGACCTCTACCTGCT
AACTAGTGGTATTTATTTGGTCAATATGGGAGATAGCTATTTGGTGTTGGCAGTCAGGGTCAAACTTGATTGTCCTTC
ATTGAGTGGTGTGTATTCTGATCAGATAGGTACTAACTAAAAAATAAACTTCTTAGAGGGACTACCTACCTCAAGTGG
GGGGAAGTCGGAGGCAATAACAGGTCTGTGATGCCCTTAGATACCTTGGGCTGCACGCGCGTTACAATGTAGACGG
AAAAAGGTCTCCGGCACCGAAAGGTGTCGGTAATCATATGAATTGTCTACGTAATGGGGATTAATTTTTGTAATTATC
GATTATCAACGAGGAATTCCTTGTAAGCGTAAATCATTACTTTGCGCTGAATATGTCCCTGCCCTTTGTACACACCGC
CCGTCGCTCCTACCGATCGAACGATTAGGTAAAACTGACAGACTGAGTGACTTCCCGCAAGGGATTGTTGTTTGGA
AAGTTAGTTAAATCTCATTGTTTAGAGGAAGGAGAATCGTAACAAGGTATCCGTAGGTGAACCTGCAGAAGGATCAA

>KP167479.1 *Heterostelium cumulocystum*
ATACAACAGTGATAAACTAAAGAACTTTCGCGCTTCGGCGTTCTGGATAACCGCAGTAAATCGGGGCTAATACATGT
AAACGAGAGGATGAGCGGGCAACTGCGAGTCTTTGCGATTGTTAGCTTTTATTCACACCAACCTCTTCGGAGTTTGT
GGTGAGTCCGAACAATATTGCTGATCGGAAACTTGTTTCCGACGAGTTCTTTGTGTCACTGCCCTATCAACTTTCGAT
GGTAAGGTATTGGCTTACCATGGTTGTAACGGGTGACGGGGAATCAGGGTTCGATTCCGGAGAGGGAGCCTGAGAA
ATGGCTACCACTTCTATGGAAGGCAGCAGGCGCGCAAATTACTCAATCCCAATACGGGGAAGTAGTGACAAAAAAT
ACTAATGCCTTTCCATTTATATGGGGGGCAATTGGAATAAGTACAATTTAAATCGCTTAGCAAAAGTGATTGGAGGGC
AAGTCTGGTGCCAGCAGCCGCGGTAATTCCAGCTCCAATAGCGTATACTAAATTTGTTGCAGTTAAAAAGCTCGTAG
TTGAGATAGAGATTTCTCGGGTTTAGCGGTCGTTATTGCCTTCACGGGTTAATAATTTGATTCGTAAAGCTTTATAGTA
AGATTCCATCTTATTATTAAAGTTACCGAGGAATTTCCAACTGCCCATGGTAACTGGTAACAGTTATCAATCGGGTGAT
CTACTGTGAGAAAATTGTAGTGTTCAAAGCAGGCGTTTACGTTTGTTCAATGCAGCATGGTATAGTAAAATATGACAC
TAAATATATGTTGGTTGTATATTCTTAGTGTAATGACTAATAGGGAAGGGCGGGGCCGTTCATATTGATGGGCGAGAG
GTGAAATTCGTTGACCCTATCAAGATGCACTACAGCGAAAGCATTCGGCAAGTACTTCTCCATTAATCAAGAACGAA
AGTTTGGGGATCGAAGACGATCAGATACCGTCGTAGTCCAAACCATAAACTATGTCGACCAGGGATTGGACGGATAA

TTTTTTAAAAACTCGCTCAGAACCTTGTGAGAAATCATGAGTGTTTGGACTCTGGGGGGAGTATGGTCGCAAGTCTG
AAACTTAAAGGAATTGACGGAAGGGCACACAATGGAGTGGAACCTGCGGCTTAATTTGACTCAACTCGGGAAAAC
TTACCAAGCTCAGATATAATAAGGATTGACAGACTAAAAGATCTTTCATGATTGTATAAGTGGTGGTGCATGGTCGTT
CTTAGTTGGTGGAGTGATTTGTCTGGTCAATTCCGATAACGGACGAGACCTCTACCTACTAACTAGTGGTATTTATTT
GGTCATTATGGGAGATAGTCATTTGGTGTTGGTAGTAGGCCGTCAAAAGTTTACTATTCTTCATTGAGTGGTGTGTATC
CTGATCAGATAGGTACTATCTAAAAAAATAAACTTCTTAGAGGGACTACCTACCTCAAGTGGGGGGAAGTCGGAGGC
AATAACAGGTCTGTGATGCCCTTAGATACCTTGGGCTGCACGCGCGTTACAATGTAGACGAGAAAAAGGCTTCCGAC
ATCGAAAGGTGCCGGTAATCAATTGAATTGTCTACGTAATGGGGATTAATTTTTGTAATTATCGATTATCAACGAGGAA
TTCCTTGTAAGCGTAAATCATTACTTTACGCTGAATATGTCCCTGCCCTTTGTACACACCGCCCGTCGCTCCTACCGAT
CGAACGATTAGGTAAAACTGACGGACTGAACGACT

>AM168099.1 *Heterostelium equisetoides* B7JB
AACCTGGTTGATCCTGCCAGTAGTCATATGCTTGTCTCAAAGATTAAGCCATGCATGTCTAAGTATAACCTTTATACGG
TGAAACTGCAGACGGCTCATTACAACAGTGATAAACTAAAGAACTTTCGCGCTTCGGCGTCTTGGATAACCGCAGTA
AATCGGGGCTAATACATGTAAACGAGAGGATAAGCGGGCAACTGCGAGTCTTTGCGATTGTTAGCTTTTATTCACAC
CAACCTCTTCGGAGTTTGTGGTGAGTCCGAACAATATTGCTGATCGGAAACTTGTTTCCGACGAGTTCTTTGTGTCA
CTGCCCTATCAACTTTCGATGGTAAGGTATTGGCTTACCATGGTTGTAACGGGTGACGGGGAATCAGGGTTCGATTCC
GGAGAGGGAGCCTGAGAAATGGCTACCACTTCTATGGAAGGCAGCAGGCGCGCAAATTACTCAATCCCAATACGGG
GAAGTAGTGACAAAAAATACTAATGCCTTTCCCTTTATAGGGGGGGCAATTGGAATAAGTACAACTTAAATCGCTTA
ACAAAAGTGATTGGAGGGCAAGTCTGGTGCCAGCAGCCGCGGTAATTCCAGCTCCAATAGCGTATACTAAATTTGTT
GCAGTTAAAAAGCTCGTAGTTGAGATTGAGATTTCTTGGGTTTAGCGTATTTTATTGCCTTCGGGTTTATAATTGCGTA
AAGCTTTTTAATAGGATTCACTCTTATTTTAAAGTTACCAAGAAATTTCCAACTGCCCATGCCAGTTAGCAATAATTGG
CTGATCGGGTGATCTACTGTGAGAAAATTGTAGTGTTCAAAGCAGGCGTTTTAAGCTTGTTCAATGCAGCATGGTATA
GTAAAATATGACACTAAACATATGTTGGTAGTATGTATTAGTGTAATGACTAATAGGGAAGGGCGGGGCCGTTCATATT
GATGGGCGAGAGGTGAAATTCGTTGACCCTATCAAGATGCACTACAGCGAAAGCATTCGGCAAGTACTTCTCCATTA
ATCAAGAACGAAAGTTTGGGGATCGAAGACGATCAGATACCGTCGTAGTCCAAACCATAAACTATGTCGACCAGGG
ATTGGACGGATAATTTTTTAAAAACTCGCTCAGAACCTTGTGAGAAATCATGAGTGTTTGGACTCTGGGGGGAGTAT
GGTCGCAAGTCTGAAACTTAAAGGAATTGACGGAAGGGCACACAATGGAGTGGAACCTGCGGCTTAATTTGACTCA
ACTCGGGAAAACTTACCAAGCTCAGATATAATAAGGATTGACAGACTAAAAGATCTTTCATGATTGTATAAGTGGTG
GTGCATGGTCGTTCTTAGTTGGTGGAGTGATTTGTCTGGTCAATTCCGATAACGGACGAGACCTCTACCTGCTAACTA
GTGGTATTTATTTGGTCAATACGGGAGATAGTTATTTGGTGTTGGCAGTTAGGCCGTTAAAAGTTTAATTGTCCTTCAT
TGAGTAGTGTGTATCCTGATCAGATAGGTACTAACTAAAAAAATAAACTTCTTAGAGGGACTACCTACCTCAAGTGG
GGGGAAGTCGGAGGCAATAACAGGTCTGTGATGCCCTTAGATACCTTGGGCTGCACGCGCGTTACAATGTAGGCGA
GAAAAAGGTTTCCGACATCGAAAGGTGCCGGTAATCAATTGAATTGCCTACGTAATGGGGATTAATTTTTGTAATTAT
CGATTATCAACGAGGAATTCCTTGTAAGCGTAAATCATTACTTTGCGCTGAATATGTCCCTGCCCTTTGTACACACCG
CCCGTCGCTCCTACCGATCGAACGATTAGGTAAAACTGACAGACTGGATGACTTCTCGCAAGGGATTGTCGTTTGGA
AAGTTAGTTAAATCTCATTGTTTAGAGGAAGGAGAAGTCGTAACAAGGTATCCGTAGGTGAACCTGCAGAAGGATC
AA

>AM168100.1 *Heterostelium filamentosum* SU-1
AACCTGGTTGATCCTGCCAGTAGTCATATGCTTGTCTCAAAGATTAAGCCATGCATGTCTAAGTATAACCTTTATACGG
TGAAACTGCAGACGGCTCATTACAACAGTGATAAACTAAAGAACTTTCGCGCTTCGGCGTCTTGGATAACCGCAGTA
AATCGGGGCTAATACATGTAAACGAGAGGATGAGCGGGCAACCGCGAGTCTTTGCGATTGTTAGCTTTTACACACAC
CAACCTCCTCGGAGATTGTGGTGAGTCCGAACAATATTGCTGATCGGAAATTTATTTCCGACGAGTTCTTTGTGTCAC
TGCCCTATCAACTTTCGATGGTAAGGTATTGGCTTACCATGGTTGTAACGGGTGACGGGGAATCAGGGTTCGATTCCG
GAGAGGGAGCCTGAGAAATGGCTACCACTTCTATGGAAGGCAGCAGGCGCGCAAATTACTCAATCCCAATACGGGA
AGTAGTGACAAAAAATACTAATGCCTTACCATTTTATGGGGGGCAATTGGAATAAGTACAACCTAAATCGCTTAGCAA
AAGTGATTGGAGGGCAAGTCTGGTGCCAGCAGCCGCGGTAATTCCAGCTCCAATAGCGTATACTAAATTTGTTGCAG
TTAAAAAGCTCGTAGTTGAGATTGAGATTTCTTGGGTTTAGCGTTCATTATTGCCTCACGGTAATAACCGTTCGTAAA
GCTTTTAATCGGGACTCGTTTCGTTTAAGAGTTACCAAGAAATTTCTATATGCCCATGTCAACTGGTAACAGTTGTCT
GATCGGGTGATCTACTGTGAGAAAATTGTAGTGTTCAAAGCAGGCGTCTTTCGCTTGTTCAATGCAGCATGGTATAGT
AAAATAAGACACTAAATATATGTTGGTTGTATATTCTAGTGTAATGACTAATAGGGAAGGGCGGGGCCGTTCATACTG
ATGGGCGAGAGGTGAAATTCGTTGACCCTATCAAGATGCACTACAGCGAAAGCATTCGGCAAGTACTTCTCCATTAA
TCAAGAACGAAAGTTTGGGGATCGAAGACGATCAGATACCGTCGTAGTCCAAACCATAAACTATGTCGACCAGGGA
TTGGACGGATAATTTTTTAAAAACTCGCTCGGAACCTTGTGAGAAATCATGAGTGTTTGGACTCTGGGGGGAGTATG
GTCGCAAGTCTGAAACTTAAAGGAATTGACGGAAGGGCACACAATGGAGTGGAACCTGCGGCTTAATTTGACTCAA
CTCGGGAAAACTTACCAAGCTCAGATATAATAAGGATTGACAGACTAAAAGATCTTTCATGATTGTATAAGTGGTGGT
GCATGGTCGTTCTTAGTTGGTGGAGTGATTTGTCTGGTCAATTCCGATAACGGACGAGACCTCTACCTACTAAATAGT
GGTATTTATTTGGTCAACATGGAAGATAGTCATTTGGTGTTGGCGTTAGGTGTCAAAGCTTAGCGTTCTTCATTGAGT
GGTGTGTATTTCGATCAGATAGGTACTAACTAAAAAATAAACTTCTTAGAGGGACTACCTACCTCAAGTGGGGGGA
GTCGGAGGCAATAACAGGTCTGTGATGCCCTTAGATACCTTGGGCTGCACGCGCGTTACAATGTAGACTAGAAAAAG
GTTTCCGACATCGAAAGGTGCCGGTAATCAATTGAATAGTCTACGTAATGGGGATTAATTTTTGTAATTATCGATTATC
AACGAGGAATTCCTTGTAAGCGTAAATCATTACTTTACGCTGAATATGTCCCTGCCCTTTGTACACACCGCCCGTCGC
TCCTACCGATCGAACGATTAGGTAAAACTGACGGACTGAATGACTTCTCGCAAGGGATTGTCGTTTGGAAAGTTAG
TTAAATCTCATTGTTTAGAGGAAGGAGAAGTCGTAACAAGGTATCCGTAGGTGAACCTGCAGAAGGATCAA

>HQ141500.1 *Heterostelium flexuosum* AU4B
AACCTGGTTGATCCTGCCAGTAGTCATATGCTTGTCTCAAAGATTAGCCATGCATGTCTAAGTATAACCTTTATACGGT
GAACCTGCAGAAGGATCAACTACAACTATGTGATAAACTATAGAACTTTCGCGCTTCGGCGTCTTGGATAACCGCAT
AAATCGGGGCTAATACATGTAAACGAAAGGATGAGCGGGCAACTGCGAGTCTTTGCGATTGTTAGCTTTCACACACC
AACCTCTTCGGAGTTTGTGGTGAGTCCGAACAATATTGCTGATCGGAAACTTGTTTCCGACAAGTTCTTTGTGTCAC
TGCCCTATCAACTTTCGATGGTAAGGTATTGGCTTACCATGGTTGTAACGGGTGACGGGGAATCAGGGTTCGATTCCG
GAGAGGGAGCCTGAGAAATGGCTACCACTTCTACGGAAGGCAGCAGGCGCGCAAATTACTCAATCCCAATACGGGG
AAGTAGTGACAAAAAATACTAATGCCTTTCCATTTATATGGGGGGCAATTGGAATAAGTACAACTTAAATCGCTTAGC
AAAAGTGATTGGAGGGCAAGTCTGGTGCCAGCAGCCGCGGTAATTCCAGCTCCAATAGCGTATACTAAATTTGTTGC

AGTTAAAAAGCTCGTAGTTTAGACTGAGATTTCTTAGGTTTAGCGGTCGGCATTGCCTTAACCGGTTAATGTTTTGAT
TCGTAAAGCTTTATAGTAGAATTTCATTTTATTATTAAAGTTACTAAGGAGTTTCCAACTGCCCATGGTAGCTAGCAAT
AGTTATCAATCGGGTGATCCACTGTGAGAAAATTGTAGTGTTCAAAGCAGACGTCTTCGTTTGTTCAATGCAGCATG
GTATGGTAAAATATGACACTAAATGTATGTTGGTTGTATATATTAGTGTAATGACTAATAGGGAAGGGCGGGGCCGTTT
ATATTGATGGGCGAGAGGTGAAATTCGTTGACCCTATCAAGATACACAACAGCGAAAGCATTCGGCAAGTACTTCTC
CATTAATCAAGAACGAAAGTTTGGGGATCGAAGACGATCAGATACCGTCGTAGTCCAAACCATAAACTATGTCGACC
AGGGATTGGACGGATAATTTTTTAAAAACTCGCTCAGAACCTTGTGAGAAATCATGAGTGTTTGGACTCTGGGGGGA
GTATGGTCGCAAGTCTGAAACTTAAAGGAATTGACGGAAGGGCACACAATGGAGTGGAACCTGCGGCTTAATTTGA
CTCAACTCGGGAAAACTTACCAAGCTCAGATATAATAAGGATTGACAGACTAAAAGATCTTTCATGATTGTATAAGTG
GTGGTGCATGGTCGTTCTTAGTTGGTGGAGTGATTTGTCTGGTCAATTCCGATAACGGACGAGACCTCTACTTACTAA
CTAGTGGTATTTATTTGGTCAATATGGGAGATAGTCATTTGGTGTTGGTAGTAGGCCGTCAAAAGTTTGCTATTCTTCA
TTGAGTGGTGTGTATTCTGATCAGATAGGTACTAACAAAAAAAAATAAACTTCTTAGAAGGACTACCTACCTCAAGTG
GGGGGAAGTCGGAGGCAATAACAGGTCTGTGATGCCCTTAGATACCTTGGGCTGCACGCGCGTTACAATGTAGGCG
AGAAAAAGGTTTCCGGAATCGAAAGGTTTCGGTAATCATTTGAATTGCCTACGTAATGGGGATTAATTTTTGTAATTA
TCGATTATCAACGAGGAATTCCTTGTAAGCGTAAATCATTACTTTACGCTGAATATGTCCCTGCCCTTTGTACACACCG
CCCGTCGCTCCTACCGATCGAACGATTAGGTAAAACTGACGGACTAGGCGATTTTTTAACCATGGTTGATCCTGGTAA
CCTGCAGAAGGATCAGCCAGTAGTCATAGTGCTTGTCTCAAAGATTAAGCCATGCATCTA

>AM168074.1 *Heterostelium gloeosporum* TCK52
AACCTGGTTGATCCTGCCAGTAGTCATATGCTTGTCTCAAAGATTAAGCCATGCATGTCTAAGTATAACCTTTATACGG
TGAAACTGCAGACGGCTCATTACAACAGTGATAAACTATAGAACTTTCGCGCTTCGGCGTCTTGGATAACCGCAGTA
AATCGGGGCTAATACATGTAAACGAAAGGATGAGCGGGCAACTGCGAGTCTTTGCGATTGTTAGCTTTCACACACCA
ACCTCTTCGGAGTTTGTGGTGAGTCCGAACAATATTGCTGATCGGAAACTTGTTTCCGACAAGTTCTTTGTGTCACT
GCCCTATCAACTTTCGATGGTAAGGTATTGGCTTACCATGGTTGTAACGGGTGACGGGGAATCAGGGTTCGATTCCG
GAGAGGGAGCCTGAGAAATGGCTACCACTTCTACGGAAGGCAGCAGGCGCGCAAATTGCTCAATCCCAATACGGG
GAAGTAGTGACAAAAAATACTAATGCCTTTCCATTTATATGGGGGGCAATTGGAATAAGTACAACTTAAATCGCTTAG
CAAAAGTGATTGGAGGGCAAGTCTGGTGCCAGCAGCCGCGGTAATTCCAGCTCCAATAGCGTATACTAAATTTGTTG
CAGTTAAAAAGCTCGTAGTTTAGATTGAGATTTCTTAGGTTTAGCGGTCGGTATTGCCTTAACCGGTTAATACTTTGAT
TCGTAAAGCTTTATAGTAGAATTTCATTTTATTATTAAAGTTACTAAGGAGTTTCCAACTGCCCATGGTAGCTAGCAAT
AGTTATCAATCGGGTGATCCACTGTGAGAAAATTGTAGTGTTCAAAGCAGACGTCTTCGTTTGTTCAATGCAGCATG
GTATGGTAAAATATGACACTAAATGTATGTTGGTTGTATATATTAGTGTAATGACTAATAGGGAAGGGCGGGGCCGTTT
ATATTGATGGGCGAGAGGTGAAATTCGTTGACCCTATCAAGATACACAACAGCGAAAGCATTCGGCAAGTACTTCTC
CATTAATCAAGAACGAAAGTTTGGGGATCGAAGACGATCAGATACCGTCGTAGTCCAAACCATAAACTATGTCGACC
AGGGATTGGACGGATAATTTTTTAAAAACTCGCTCAGAACCTTGTGAGAAATCATGAGTGTTTGGACTCTGGGGGGA
GTATGGTCGCAAGTCTGAAACTTAAAGGAATTGACGGAAGGGCACACAATGGAGTGGAACCTGCGGCTTAATTTGA
CTCAACTCGGGAAAACTTACCAAGCTCAGATATAATAAGGATTGACAGACTAAAAGATCTTTCATGATTGTATAAGTG
TGGTGCATGGTCGTTCTTAGTTGGTGGAGTGATTTGTCTGGTCAATTCCGATAACGGACGAGACCTCTACTTACTAAC
TAGTGGTATTTATTTGGTCAATATGGGAGATAGTCATTTGGTGTTGGTAGTAGGCCGTTAAAAGTTTGCTATTCTTCAT
TGAGTGGTGTGTATTCTGATCAGATAGGTACTAACAAAAAAAAATAAACTTCTTAGAAGGACTACCTACCTCAAGTGG
GGGGAAGTCGGAGGCAATAACAGGTCTGTGATGCCCTTAGATACCTTGGGCTGCACGCGCGTTACAATGTAGGCGA
GAAAAAGGTTTCCGGAATCGAAAGGTTTCGGTAATCATTTGAATTGCCTACGTAATGGGGATTAATTTTTGTAATTAT
CGATTATCAACGAGGAATTCCTTGTAAGCGTAAATCATTACTTTACGCTGAATATGTCCCTGCCCTTTGTACACACCGC
CCGTCGCTCCTACCGATCGAACGATTAGGTAAAACTGACGGACTAAACGATTTTTTGGCAACAAATTTTTGTTTGGG
AAGTTAGTTAAATCTCATTGTTTAGAGGAAGGAGAAGTCGTAACAAGGTATCCGTAGGTGAACCTGCAGAAGGATC
AA

>HQ141502.1 *Heterostelium granulosum* MF5A
AACCTGGTTGATCCTGCCAGTAGTCATATGCTTGTCTCAAAGATTAGCCATGCATGTCTAAGTATAACCTTTATACGGT
GAACCTGCAGAAGGATCAACTACAACATGTGATAAACTATAGAACTTTCGCGCTTCGGCGTCTTGGATAACCGCAGT
AAATCGGGGCTAATACATGTAAACGAAAGGATGAGCGGGTAACTGCGAGTCTTTGCGATTGTTAGCTTTCACACACC
AACCTCTTCGGAGTTTGTGGTGAGTCCGAACAATATTGCTGATCGGAAATTTATTTCCGACAAGTTCTTTGTGTCACT
GCCCTATCAACTTTCGATGGTAAGGTATTGGCTTACCATGGTTGTAACGGGTGACGGGGAATCAGGGTTCGATTCCG
GAGAGGGAGCCTGAGAAATGGCTACCACTTCTACGGAAGGCAGCAGGCGCGCAAATTACTCAATCCCAATACGGGG
AAGTAGTGACAAAAAATACTAATGCCTTTCCATTTATATGGGGGGCAATTGGAATAAGTACAACTTAAATCGCTTAGC
AAAAGTGATTGGAGGGCAAGTCTGGTGCCAGCAGCCGCGGTAATTCCAGCTCCAATAGCGTATACTAAATTTGTTGC
AGTTAAAAAGCTCGTAGTTTAGACTGAGATTTCTTGGGTCTAGCGGTCTGCATTGCCTTTACCGGTTAATGTTTGATT
CGTAAAGCTTTATAGTAGAATTTCATTTTATTATTAAAGTTACCAAGGAGTTTCCAACTGCCCATGGTAGCTAGCAATA
GTTATCAATCGGGTGATCCACTGTGAGAAAATTGTAGTGTTCAAAGCAGACGTTTCGTTTGTTCAATGCAGCATGGT
ATGGTAAAATATGACACTAAATGTATGTTGGTTGTATATATTAGTGTAATGACTAATAGGGAAGGGCGGGGCCGTTTAT
ATTGATGGGCGAGAGGTGAAATTCGTTGACCCTATCAAGATACACAACAGCGAAAGCATTCGGCAAGTACTTCTCCA
TTAATCAAGAACGAAAGTTTGGGGATCGAAGACGATCAGATACCGTCGTAGTCCAAACCATAAACTATGTCGACCAG
GGATTGGACGGATAATTTTTTAAAAACTCGCTCAGAACCTTGTGAGAAATCATGAGTGTTTGGACTCTGGGGGGAGT
ATGGTCGCAAGTCTGAAACTTAAAGGAATTGACGGAAGGGCACACAATGGAGTGGAACCTGCGGCTTAATTTGACC
AACTCGGGAAAACTTACCAAGCTCAGATATAATAAGGATTGACAGACTAAAAGATCTTTCATGATTGTATAAGTGGT
GGTGCATGGTCGTTCTTAGTTGGTGGAGTGATTTGTCTGGTCAATTCCGATAACGGACGAGACCTCTACTTACTAACT
AGTGGTATTTATTTGGTCAATATGGGAGATAGTCATTTGGTGTTGGTAGTAGGCCGTCAAAAGCTTGCTATTCTTCATT
GAGTGGTGTGTATTCTGATCAGATAGGTACTAACAAAAAAAAATAAACTTCTTAGAAGGACTACCTACCTCAAGTGGG
GGGAAGTCGGAGGCAATAACAGGTCTGTGATGCCCTTAGATACCTTGGGCTGCACGCGCGTTACAATGTAGGCGAG
AAAAAGGTTTCCGGAATCGAAAGGTTTCGGTAATCATTTGAATTGCCTACGTAATGGGGATTAATTTTTGTAATTATC
GATTATCAACGAGGAATTCCTTGTAAGCGTAAATCATTACTTTACGCTGAATATGTCCCTGCCCTTTGTACACACCGCC
CGTCGCTCCTACCGATCGAACGATTAGGTAAAACTGACGGACTAGGCGATTTTTTAAGCCATGGTTGATCCTGGTGA
ACCTGCAGAAGGATCAGCCAGTAGATCATAGTGCTTGTCTCAAAGATTAAGCCGTGGCA

>MH762955.1 *Heterostelium irregularibrachiatum* Krug6-5B
CTAAAGAACTTTCGCGCTTCGGCGTCTTGGATAACCGCAGTAAATCGGGGCTAATACATGTAAACGAGAGGATGAGC

GGGCAACTGCGAGTCTTTGCGATTGTTAGCTATTCACACACCAACCTCTTCGGAGTTTGTGGTGAGTCCGAACAATA
TTGCTGATCGGAAATTTATTTCCGACAAGTTCTTTGTGTCACTGCCCTATCAACTTTCGATGGTAAGGTATTGGCTTAC
CATGGTTGTAACGGGTGACGGGGAATCAGGGTTCGATTCCGGAGAGGGAGCCTGAGAAATGGCTACCACTTCTATG
GAAGGCAGCAGGCGCGCAAATTACTCAATCCCAATACGGGGAAGTAGTGACAAAAAATACTAATGCCTTTCCATTTA
TATGGGGGGCAATTGGAATAAGTACAACTTAAATCGCTTAGCAAAAGTGATTGGAGGGCAAGTCTGGTGCCAGCAG
CCGCGGTAATTCCAGCTCCAATAGCGTATACTAAATTTGTTGCAGTTAAAAAGCTCGTAGTTGAGATTGAGATTTCTT
GGGTTTAGCAGGCATTGTCGCCTTCGGGTTGGCAATCGTTTGTAAAGCGCTATAATGGAATTTCACTTTCATTATATGT
GTTACCAAGAAATTTCCAACTGCCCATGTCAAGTGGCAACACTTGTCTGATAGGGTGATCTACTGTGAGAAAATTGG
TGTTCAAAGCAGGCGTCTTACGTTTGTTCAATGCAGCATGGTATAGTAAAATAAGACACTAAATATATGTTGGTTGTAT
ATTCTTAGTGTAATGACTAATAGGGAAGGGCGGGGCCGTTCATATTGATGGGCGAGAGGTGAAATTCGTTGACCCTAT
CAAGATGCACTACAGCGAAAGCATTCGGCAAGTACTTCTCCATTAATCAAGAACGAAAGTTTGGGGATCGAAGACG
ATCAGATACCGTCGTAGTCCAAACCATAAACTATGTCGACCAGGGATTGGACGGATAATTTTTTAAAAACTCGCTCAG
AACCTTGTGAGAAATCATGAGTGTTTGGACTCTGGGGGGAGTATGGTCGCAAGTCTGAAACTTAAAGGAATTGACG
GAAGGGCACACAATGGAGTGGAACCTGCGGCTTAATTTGACTCAACTCGGGAAAACTTACCAAGCTCAGATATAAT
AAGGATTGACAGACTAAAAGATCTTTCATGATTGTATAAGTGGTGGTGCATGGTCGTTCTTAGTTGGTGGAGTGATTT
GTCTGGTCAATTCCGATAACGGACGAGACCTCTACCTGCTAACTAGTGGTATTTATTTGGTCAATATGGGAGATAGCT
ATTTGGTGTTGGCAGTCAGGGTCAAACTTGATTGTCCTTCATTGAGTGGTGTGTATTCTGATCAGATAGGTACTAACT
AAAAAATAAACTTCTTAGAGGGACTACCTACCTCAAGTGGGGGGAAGTCGGAGGCAATAACAGGTCTGTGATGCCC
TTAGATACCTTGGGCTGCACGCGCGTTACAATGTAGACGAGAAAAAGGTCTCCGGCACCGAAAGGTGTCGGTAATC
ATATGAATTGTCTACGTAATGGGGATTAATTTTTGTAATTATCGATTATCAACGAGGAATTCCTTGTAAGCGTAAATCAT
TACTTTGCGCTGAATATGTCCCTGCCCTTTGTACACACCGCCCGTCGCTCCTACCGATCGAACGATTAGGTAAAACTG
ACAGA

>KP167477.1 *Heterostelium lapidosum*
AACCTGGTTGATCCTGCCAGTAGTCATATGCTTGTCTCAAAGATTAAGCCATGCATGTCTAAGTATAACCTTTATACGG
TGAACCTGCAGAAGGATCATCCCGGTTTAGATAAACTATAAGAACTTTCGCGCTTCGGCGTCTTGGATAACCGCAGT
AAATCGGGGCTAATACATGTAAACGAGAGGATGAGCGGGCAACTGCGAGTCTTTGCGATTGTTAGCTATTCACACAC
CAACCTCTTCGGAGTTTGTGGTGAGTCCGAACAATATTGCTGATCGGAAATTTATTTCCGACAAGTTCTTTGTGTCAC
TGCCCTATCAACTTTCGATGGTAAGGTATTGGCTTACCATGGTTGTAACGGGTGACGGGGAATCAGGGTTCGATTCCG
GAGAGGGAGCCTGAGAAATGGCTACCACTTCTATGGAAGGCAGCAGGCGCGCAAATTACTCAATCCCAATACGGGG
AAGTAGTGACAAAAAATACTAATGCCTTTCCATTTATATGGGGGGCAATTGGAATAAGTACAACTTAAATCGCTTAGC
AAAAGTGATTGGAGGGCAAGTCTGGTGCCAGCAGCCGCGGTAATTCCAGCTCCAATAGCGTATACTAAATTTGTTGC
AGTTAAAAAGCTCGTAGTTGAGATTGAGATTTCTTGGGTTTAGCAGGCATTGTCGCCTTCGGGTTGGCAATCGTTTG
TAAAGCGCTATAATGGGATTTCACTCTCATTATATGTGTTACCAAGAAATTTCCAACTGCCCATGTCAAGTGGCAACA
CTTGTCTGATAGGGTGATCTACTGTGAGAAAATTGTAGTGTTCAAAGCAGGCGTCTTACGTTTGTTCAATGCAGCATG
GTATAGTAAAATATGACACTAAATATATGTTGGTTGTATATTCTTAGTGTAATGACTAATAGGGAAGGGCGGGGCCGTT
CATATTGATGGGCGAGAGGTGAAATTCGTTGACCCTATCAAGATGCACTACAGCGAAAGCATTCGGCAAGTACTTCT
CCATTAATCAAGAACGAAAGTTTGGGGATCGAAGACGATCAGATACCGTCGTAGTCCAAACCATAAACTATGTCGAC
CAGGGATTGGACGGATAATTTTTTAAAAACTCGCTCAGAACCTTGTGAGAAATCATGAGTGTTTGGACTCTGGGGGG
AGTATGGTCGCAAGTCTGAAACTTAAAGGAATTGACGGAAGGGCACACAATGGAGTGGAACCTGCGGCTTAATTTG
ACTCAACTCGGGAAAACTTACCAAGCTCAGATATAATAAGGATTGACAGACTAAAAGATCTTTCATGATTGTATAAGT
GGTGGTGCATGGTCGTTCTTAGTTGGTGGAGTGATTTGTCTGGTCAATTCCGATAACGGACGAGACCTCTACCTGCT
AACTAGTGGTATTTATTTGGTCAATATGGGAGATAGCTATTTGGTGTTGGCAGTCAGGGTCAAACTTGATTGTCCTTC
ATTGAGTGGTGTGTATTCTGATCAGATAGGTACTAACTAAAAAATAAACTTCTTAGAGGGACTACCTACCTCAAGTGG
GGGGAAGTCGGAGGCAATAACAGGTCTGTGATGCCCTTAGATACCTTGGGCTGCACGCGCGTTACAATGTAGACGA
GAAAAAGGTCTCCGGCACCGAAAGGTGTCGGTAATCATATGAATTGTCTACGTAATGGGGATTAATTTTTGTAATTAT
CGATTATCAACGAGGAATTCCTTGTAAGCGTAAATCATTACTTTGCGCTGAATATGTCCCTGCCCTTTGTACACACCG
CCCGTCGCTCCTACCGATCGAACGATTAGGTAAAACTGACAGACTGTAACGGATCTGTAACCTGGTTGATCCTGCCA
GTAGTCATATGCTTGTCTCAAAGATAAGCCATGCATCTAAGTAACCT

>AM168101.1 *Heterostelium luridum* LR-2
AACCTGGTTGATCCTGCCAGTAGTCATATGCTTGTCTCAAAGATTAAGCCATGCATGTCTAAGTATAACCTTTATACGG
TGAAACTGCAGACGGCTCATTACAACAGTGATAAACTAAAGAACTTTCGCGCTTCGGCGTCTTGGATAACCGCAGTA
AATCGGGGCTAATACATGTAAACGAGAGGATGAGCGGGCAACCGCGAGTCTTTGCGATTGTTAGCTTTTACACACAC
CAGCCTCCTCGGAGATTGTGGTGAGTCCGAACAATATTGCTGATCGGAAATTTATTTCCGACGAGTTCTTTGTGTCAC
TGCCCTATCAACTTTCGATGGTAAGGTATTGGCTTACCATGGTTGTAACGGGTGACGGGGAATCAGGGTTCGATTCCG
GAGAGGGAGCCTGAGAAATGGCTACCACTTCTATGGAAGGCAGCAGGCGCGCAAATTACTCAATCCCAATACGGGG
AAGTAGTGACAAAAAATACTAATGCCTTACCATTTTATGGGGGGCAATTGGAATAAGTACAACCTAAATCGCTTAGCA
AAAGTGATTGGAGGGCAAGTCTGGTGCCAGCAGCCGCGGTAATTCCAGCTCCAATAGCGTATACTAAATTTGTTGCA
GTTAAAAAGCTCGTAGTTGAGATTGAGATTTCTTGGGTTTAGCGTTCATTATTGCCTCACGGTAATAACCGTTCGTAA
AGCTTTTAATCGGGACTCGTTTCGTTTAAGAGTTACCAAGAAATTTCTATATGCCCATGTCAACTGGTAACAGTTGTC
TGATCGGGTGATCTACTGTGAGAAAATTGTAGTGTTCAAAGCAGGCGTCTTTCGCTTGTTCAATGCAGCATGGTATAG
TAAAATAAGACACTAAATATATGTTGGTTGTATATTCTAGTGTAATGACTAATAGGGAAGGGCGGGGCCGTTCATATTG
ATGGGCGAGAGGTGAAATTCGTTGACCCTATCAAGATGCACTACAGCGAAAGCATTCGGCAAGTACTTCTCCATTAA
TCAAGAACGAAAGTTTGGGGATCGAAGACGATCAGATACCGTCGTAGTCCAAACCATAAACTATGTCGACCAGGGA
TTGGACGGATAATTTTTTAAAAACTCGCTCAGAACCTTGTGAGAAATCATGAGTGTTTGGACTCTGGGGGGAGTATG
GTCGCAAGTCTGAAACTTAAAGGAATTGACGGAAGGGCACACAATGGAGTGGAACCTGCGGCTTAATTTGACTCAA
CTCGGGAAAACTTACCAAGCTCAGATATAATAAGGATTGACAGACTAAAAGATCTTTCATGATTGTATAAGTGGTGGT
GCATGGTCGTTCTTAGTTGGTGGAGTGATTTGTCTGGTCAATTCCGATAACGGACGAGACCTCTACCTACTAAATAGT
GGTATTTATTTGGTCAACATGGAAGATAGTCATTTGGTGTTGGCGTTAGGTGTCAAAGCTTAGCGTTCTTCATTGAGT
GGTGTGTATTTCGATCAGATAGGTACTAACTAAAAAATAAACTTCTTAGAGGGACTACCTACCTCAAGTGGGGGGAA
GTCAGAGGCAATAACAGGTCTGTGATGCCCTTAGATACCTTGGGCTGCACGCGCGTTACAATGTAGACTAGAAAAAG
GTTTCCGACATCGAAAGGTGCCGGTAATCAATTGAATAGTCTACGTAATGGGGATTAATTTTTGTAATTATCGATTATC
AACGAGGAATTCCTTGTAAGCGTAAATCATTACTTTACGCTGAATATGTCCCTGCCCTTTGTACACACCGCCCGTCGC
TCCTACCGATCGAACGATTAGGTAAAACTGACGGACTGAATGACTTCTCGCAAGGGATTGTCGTTTGGAAAGTTAG

TTAAATCTCATTGTTTAGAGGAAGGAGAAGTCGTAACAAGGTATCCGTAGGTGAACCTGCAGAAGGATCAA

>KP167481.1 *Heterostelium migratissimum*
GATAAACTAAAGAACTTCCGCGCTTCGGCGTTTTGGATAACCGCAGTAAATCGGGGCTAATACATGTAAACGAGAGG
ATGAACGGGCAACTGTGAGTCTTTGCGATTGTTAGCTTTAATCACCAACCTCTTCGGAGTCAGTGGTGAATCCCTAC
AATATTGCTGATCGGAAACTTGTTTCCGACGAGTTCTTTGTGTCACTGCCCTATCAACTTTCGATGGTAAGGTATTGG
CTTACCATGGTTGTAACGGGTGACGGGGAATCAGGGTTCGATTCCGGAGAGGGAGCCTGAGAAATGGCTACCACTT
CTACGGAAGGCAGCAGGCGCGCAAATTACTCAATCCCAATACGGGGAAGTAGTGACAAAAAATACTAATGCCTTTC
CATTTATATGGGGGGCAATTGGAATAAGTACAATTTAAATCGCTTAACGAAAGTGATTGGAGGGCAAGTCTGGTGCC
AGCAGCCGCGGTAATTCCAGCTCCAATAGCGTATACTAAATTTGTTGCAGTTAAAAAGCTCGTAGTTGAGATTGAGAT
TTCTCAGGTTTAGCGGTCGGCATGCCTTCACGGGTTTTGCTTGATACGTAAAGCTTTATAGTAGAATTTCATTTTATTA
TTAAAGTTACTGAGGAATTTCCAACTGCCCATGGTTGTCAGCAATGGCTTCCAATAGGGTGATCTACTGTGAGAAAA
TTGTAGTGTTCAAAGCAGGCGTCTTTCGTTTGTTCAATGCAGCATGGTATAGTTAAATATGACACTAAATATATGTTGG
TTGTATATTCTTAGTGTAATGACTAATAGGGAAGGGCGGGGCCGTTCATATTGATGGGCGAGAGGTGAAATTCGTTGA
CCCTATCAAGATGCACTACAGCGAAAGCATTCGGCAAGTACTTCTCCATTAATCAAGAACGAAAGTTTGGGGATCGA
AGACGATCAGATACCGTCGTAGTCCAAACCATAAACTATGTCGACCAGGGATTGGACGGATAATTTTTTAAAAACTC
GCTCAGAACCTTGTGAGAAATCATGAGTGTTTGGACTCTGGGGGGAGTATGGTCGCAAGTCTGAAACTTAAAGGAA
TTGACGGAAGGGCACACAATGGAGTGGAACCTGCGGCTTAATTTGACTCAACTCGGGAAAACTTACCAAGCTCAGA
TATAATAAGGATTGACAGACTAAAAGATCTTTCATGATTGTATAAGTGGTGGTGCATGGTCGTTCTTAGTTGGTGGAG
TGATTTGTCTGGTCAATTCCGATAACGGACGAGACCTCTACTTACTAACTAGTGATATTTATTTGGCCAATATGGGAGA
TAGTCATTTGGTGTTGGTAGTAGGACGTCAAAATCTTACTATTCTTCATTGAGTGGTGTGTATTCTGGTCAGATAGGTA
CTAATTTTAAAAATAAACTTCTTAGAAGGACTACCTACCTCAAGTGGGGGGAAGTCGGAGGCAATAACAGGTCTGTG
ATGCCCTTAGATACCTTGGGCTGCACGCGCGTTACAATGTAGGCGAGAAAAAGGTTTCCGGAATCGAAAGGTTTCG
GTAATCAATTGAATTGCCTACGTAATGGGGATTAATTTTTGTAATTATCGATTATCAACGAGGAATTCCTTGTAAGCGT
AAATCATTACTTTACGCTGAATATGTCCCTGCCCTTTGTACACACCGCCCGTCGCTCCTACCGATCGAACGATTAGGT
AAAACTGACGGACTAGGCGAC

>HQ141506.1 *Heterostelium multicystogenum* AS2
AACCTGGTTGATCCTGCCAGTAGTCATATGCTTGTCTCAAAGATTAAGCCATGCATGTCTAAGTATAACCTTTATACGG
TGAACCTGCAGAAGGATCAACTACAAGCATGTAGATAAACTAAAGAACTTTCGCGCTTCGGCGTTTTGGATAACCGC
AGTAAATCGGGGCTAATACATATAAACGAGAGGATGAGCGGGCAACTGCGAGTCTTTGCGATTGTTAGCTTTTTCAC
CAATCTCTTCGGAGTTTGTGGTGAATCCCTACAATATTGCTGATCGGAAACTTGTTTCCGACGAGTTCTTTGTGTCAC
TGCCCTATCAACTTTCGATGGTAAGGTATTGGCTTACCATGGTTGTAACGGGTGACGGGGAATCAGGGTTCGATTCCG
GAGAGGGAGCCTGAGAAATGGCTACCACTTCTACGGAAGGCAGCAGGCGCGCAAATTACTCAATCCCAATACGGGG
AAGTAGTGACAAAAAATACTAATGCCTTTCCATTTATATGGGGGGCAATTGGAATAAGTACAACTTAAATCGCTTAAC
AAAAGTGATTGGAGGGCAAGTCTGGTGCCAGCAGCCGCGGTAATTCCAGCTCCAATAGCGTATACTAAATTTGTTGC
AGTTAAAAAGCTCGTAGTTGAGATTGAGATTCTTTGGGTTTAACAGTCAGCATTGCCTTCACCGGTTAATGCTTTGAT
CTGTAAAGCTTTATAGCAGAATTTCATTTTGTTATTAAAGTTACCAAAGAATTTCTATCTGCCCATGGTAGCCAGCAAT
GGTTATCAATCGGGTGATCTACTGTGAGAAAATTGTAGTGTTCAAAGCAGGCGTCTTACGTTTGTTCAATGCAGCATG
GTATAGTAAAATATGACACTTAACATATGTTGGTTGTATGTTTTAGTGTAATGACTAATAGGGAAGGGCGGGGCCGTT
CATATTGATGGGCGAGAGGTGAAATTCGTTGACCCTATCAAGATGCACTACAGCGAAAGCATTCGGCAAGTACTTCT
CCATTAATCAAGAACGAAAGTTTGGGGATCGAAGACGATCAGATACCGTCGTAGTCCAAACCATAAACTATGTCGAC
CAGGGATTGGACGGATAATTTTTTAAAAACTCGCTCAGAACCTTGTGAGAAATCATGAGTGTTTGGACTCTGGGGGG
AGTATGGTCGCAAGTCTGAAACTTAAAGGAATTGACGGAAGGGCACACAATGGAGTGGAACCTGCGGCTTAATTTG
ACTCAACTCGGGAAAACTTACCAAGCTCAGATATAATAAGGATTGACAGACTAAAAGATCTTTCATGATTGTATAAGT
GGTGGTGCATGGTCGTTCTTAGTTGGTGGAGTGATTTGTCTGGTCAATTCCGATAACGGACGAGACCTCTACTTACTA
ACTAGTGGTATTTATTTGGTCAATACGGGAGATAGTCATTTGGTGTTGGTAGTAGGACGTCAAAATCTTATTATTCTTC
ATTGAGTGGTGTGTATCCTGATCAGATAGGTACTAACTTAAAAAATAAACTTCTTAGAAGGACTACCTACCTCAAGTG
GGGGGAAGTCGGAGGCAATAACAGGTCTGTGATGCCCTTAGATACCTTGGGCTGCACGCGCGTTACAATGTAGGCG
AGAAAAAGGTTTCCGGAATCGAAAGGTTTCGGTAATCATTTGAATTGCCTACGTAATGGGGATTAATTTTTGTAATTA
TCGATTATCAACGAGGAATTCCTTGTAAGCGTAAATCATTACTTTACGCTGAATATGTCCCTGCCCTTTGTACACACCG
CCCGTCGCTCCTACCGATCGAACGATTAGGTAAAACTGACGGACTAGGCGACTTTTAACCATGGTTGATCCGGTGAA
CCTGCAGAAGGATCATGCCAGTAGTCATATGCTTGTCTCAAAGATTAAGCC

>HQ141497.1 *Heterostelium oculare*
AACCTGGTTGATCCTGCCAGTAGTCATATGCTTGTCTCAAAGATTAAGCCATGCATGTCTAAGTATAAACCTTTATACG
GTGAACCTGCAGAAGGATCAACTCACAGTTTGTGATAAACTAAAGAACTTTCGCGCTTAGGCGTCTTGGATAACCGC
AGTAAATCGGGGCTAATACATATAAACGAAAGGATGAGCGGGTAACCGCGAGTCTTTGCGATTGTTAGTTTTGCTTTT
TCATACCAACCTCCTCGGAGTTTGTGTTGAATCCGAACAATATTGCTGATCGGAAACTTGTTTCCGACGAGTTCTATG
TGTCACTGCCCTATCAACTTTCGATGGTAAGGTATTGGCTTACCATGGTTGTAACGGGTGACGGGGAATCAGGGTTC
GATTCCGGAGAGGGAGCCTGAGAAATGGCTACCACTTCTACGGAAGGCAGCAGGCGCGCAAATTACTCAATCCCAA
TACGGGGAAGTAGTGACAAAAAATACTAATGCCCTTCCATTTTTATGGGGGGCAATTGGAATAAGTACAACTTAAAT
CGCTTAGCAAAAGTGATTGGAGGGCAAGTCTGGTGCCAGCAGCCGCGGTAATTCCAGCTCCAATAGCGTATACTAAA
TTTGTTGCAGTTAAAAAGCTCGTAGTTTAGATTGAGATTTTTTGGGTTTAAAGCTCATCTTTTTGCTTAACGGTAATTA
GGTGTAGTGTATAAAGCTTTTATGTAGACTTCGCGTTTATATATAAAGTTACCAAAAAATTTCATTCTGCCCATACTAA
AATTTATTTTAGTAATCGGGTGTTCTACTGTGAGAAAATTGTAGTGTTTAAAGCAGGCGTCTTTGTTTGTTCAATGCA
GCATGGTATAGTAAAAAAAGACACTAAACATTTGTTGGTTAATGTTATAGTGTAATGACTAATAGGGAAGGGCGGGG
CCGTTCATATTGATGGGCGAGAGGTGAAATTCGTTGACCCTATCAAGATGCACTACAGCGAAAGCATTCGGCAAGTA
CTTCTCCATTAATCAAGAACGAAAGTTTGGGGATCGAAGACGATCAGATACCGTCGTAGTCCAAACCATAAACTATG
TCGACCAGGGACTGGACGGTTAATTTTATAAAAACTCGCTCAGAACCTTGTGGGAAACCATGAGTGTTTGGACTCTG
GGGGGAGTATGGTCGCAAGTCTGAAACTTAAAGGAATTGACGGAAGGGCACACAATGGAGTGGAACCTGCGGCTT
AATTTGACTCAACTCGGGAAAACTTACCAAGCTCAGATATAATAAGGATTGACAGACTAAAAGATCTTTCATGATTGT
ATAAGTGGTGGTGCATGGTCGTTCTTAGTTGGTGGAGTGATTTGTCTGGTCAATTCCGATAACGGACGAGACCTCTA
CCTACTAACTAGTGGTACTTATTTAGTCAATATGGGGGATAGCTAGTTGGTGTTGGTAATTAGTCTTCACGGATTAATT
ATCTTTCATTGATCTAGTGTGTACTCTGATTAGATAAGTACTAACTAAAAAAATAAAACTTCTTAGAGGGACTACCTAC

CTCAAGTGGGGGGAAGTCGGAGGCAATAACAGGTCTGTGATGCCCTTAGATACCTTGGGCTGCACGCGCGTTACAA
TGTAAGTGAGAAAAAGGTTTCCGGTATCGAAAGGTATCGGTAATCATTTGAATTACCTACGTAATGGGGATTGATCTT
TGTAATTATCGATCATCAACGAGGAATTCCTTGTAAGCGTAAATCATTACTTTACGCTGAATATGTCCCTGCCCTTTGT
ACACACCGCCCGTCGCTCCTACCGATCGAACGCTTAGGTAAAACTGACGGACTATATGATATTAACGCTGGTTGATAC
TCTGTCCGATGTAGATACGATATGTCTTGTCTCCAAAGATTAAGCCAGCAGTGTTAAGGGTGAACCTGCAGAAGGAT
CA

>AM168079.1 *Heterostelium oculare* DB4B
AACCTGGTTGATCCTGCCAGTAGTCATATGCTTGTCTCAAAGATTAAGCCATGCATGTCTAAGTATAAACCTTTATACG
GTGAAACTGCAGACGGCTCATTACAACAGTGATAAACTAAAGAACTTTCGCGCTTAGGCGTCTTGGATAACCGCAG
TAAATCGGGGCTAATACATATAAACGAAAGGATGAGCGGGTAACCGCGAGTCTTTGCGATTGTTAGTTTTGCTTTTTC
ATACCAACCTCCTCGGAGTTTGTGTTGAATCCGAACAATATTGCTGATCGGAAACTTGTTTCCGACGAGTTCTATGTG
TCACTGCCCTATCAACTTTCGATGGTAAGGTATTGGCTTACCATGGTTGTAACGGGTGACGGGGAATCAGGGTTCGAT
TCCGGAGAGGGAGCCTGAGAAATGGCTACCACTTCTACGGAAGGCAGCAGGCGCGCAAATTACTCAATCCCAATAC
GGGGAAGTAGTGACAAAAAATACTAATGCCCTTCCATTTTTATGGGGGGCAATTGGAATAAGTACAACTTAAATCGC
TTAGCAAAAGTGATTGGAGGGCAAGTCTGGTGCCAGCAGCCGCGGTAATTCCAGCTCCAATAGCGTATACTAAATTT
GTTGCAGTTAAAAAGCTCGTAGTTTAGATTGAGATTTTTTGGGTTTAAAGCTCATCTTTTTGCTTAACGGTAATTAGG
TGTAGTGTATAAAGCTTTTATGTAGACTTCGCGTTTATATATAAAGTTACCAAAAAATTTCATTCTGCCCATACTAAAAT
TTATTTTAGTAATCGGGTGTTCTACTGTGAGAAAATTGTAGTGTTTAAAGCAGGCGTCTTTGTTTGTTCAATGCAGCA
TGGTATAGTAAAAAAAGACACTAAACATTTGTTGGTTAATGTTATAGTGTAATGACTAATAGGGAAGGGCGGGGCCG
TTCATATTGATGGGCGAGAGGTGAAATTCGTTGACCCTATCAAGATGCACTACAGCGAAAGCATTCGGCAAGTACTT
CTCCATTAATCAAGAACGAAAGTTTGGGGATCGAAGACGATCAGATACCGTCGTAGTCCAAACCATAAACTATGTCG
ACCAGGGACTGGACGGTTAATTTTATAAAAACTCGCTCAGAACCTTGTGGGAAACCATGAGTGTTTGGACTCTGGG
GGGAGTATGGTCGCAAGTCTGAAACTTAAAGGAATTGACGGAAGGGCACACAATGGAGTGGAACCTGCGGCTTAAT
TTGACTCAACTCGGGAAAACTTACCAAGCTCAGATATAATAAGGATTGACAGACTAAAAGATCTTTCATGATTGTATA
GTGGTGGTGCATGGTCGTTCTTAGTTGGTGGAGTGATTTGTCTGGTCAATTCCGATAACGGACGAGACCTCTACCTA
CTAACTAGTGGTACTTATTTAGTCAATATGGGGGATAGCTAGTTGGTGTTGGTAATTAGTCTTCACGGATTAATTATCTT
TCATTGATCTAGTGTGTACTCTGATTAGATAAGTACTAACTAAAAAAATAAAACTTCTTAGAGGGACTACCTACCTCA
AGTGGGGGGAAGTCGGAGGCAATAACAGGTCTGTGATGCCCTTAGATACCTTGGGCTGCACGCGCGTTACAATGTA
AGTGAGAAAAAGGTTTCCGGTATCGAAAGGTATCGGTAATCATTTGAATTACCTACGTAATGGGGATTGATCTTTGTA
ATTATCGATCATCAACGAGGAATTCCTTGTAAGCGTAAATCATTACTTTACGCTGAATATGTCCCTGCCCTTTGTACAC
ACCGCCCGTCGCTCCTACCGATCGAACGCTTAGGTAAAACTGACGGACTATATGATATTATAGCAATATGATACTTGCT
GTGGGAAGTTAGTTAAATCTCATTGTTTAGAGGAAGGAGAAGTCGTAACAAGGTATCCGTAGGTGAACCTGCAGAA
GGATCAA

>EU004605.1 *Heterostelium pallidum* PPHU8
TCCGAATTCGTCGACACCTGGTTGATCCTGCCAGTAGTCATATGCTTGTCTCAAAGATTAAGCCATGCATGTCTAAGT
ATAACCTTTATACGGTGAAACTGCAGACGGCTCATTACAACAGTGATAAACTAAAGAACTTTCGCGCTTCGGCGTCT
TGGATAACCGCAGTAAATCGGGGCTAATACATGTAAACGAGAGGATGAGCAGGTAACTGCGAGTCTTTGCGATTGTT
AGCTTTCATTCACACCAACCTCTTCGGAGTTTGTGGTGAGTCCGAACAATATTGCTGATCGGAAACTTGTTTCCGAC
GAGTTCTTTGTGTCACTGCCCTATCAACTTTCGATGGTAAGGTATTGGCTTACCATGGTTGTAACGGGTGACGGGGA
ATCAGGGTTCGATTCCGGAGAGGGAGCCTGAGAAATGGCTACCACTTCTACGGAAGGCAGCAGGCGCGCAAATTAC
TCAATCCCAATACGGGGAAGTAGTGACAAAAAATACTAATGCCTTTCCATTTATATGGGGGGCAATTGGAATAAGTAC
AACTTAAATCGCTTAGCAAAAGTGATTGGAGGGCAAGTCTGGTGCCAGCAGCCGCGGTAATTCCAGCTCCAATAGC
GTATACTAAATTTGTTGCAGTTAAAAAGCTCGTAGTTGAGATTGAGATTTCTTAGGTTTAGAGTCGCTTGGCCTTCGG
GTTTGAGTTTGATTCGTAAAGCTCTATAGTAGAATTCCATTTTATTATTAGGGTTACTAAGAAATTTCTATCTGCCCATG
GTAGCTAGCAATAGTTATCAATCGGGTGATCTACTGTGAGAAAATTGTAGTGTTCAAAGCAGGCGTCTTACGTTTGTT
CAATGCAGCATGGTATAGTAAAATATGACACTAAATATATGTTGGTTGTATATTCTTAGTGTAATGACTAATAGGGAAG
GGCGGGGCCGTTCATATTGATGGGCGAGAGGTGAAATTCGTTGACCCTATCAAGATGCACTACAGCGAAAGCATTCG
GCAAGTACTTCTCCATTAATCAAGAACGAAAGTTTGGGGATCGAAGACGATCAGATACCGTCGTAGTCCAAACCATA
AACTATGTCGACCAGGGATTGGACGGATAATTTTTAAAAACTCGCTCAGAACCTTGTGAGAAATCATGAGTGTTTG
GACTCTGGGGGGAGTATGGTCGCAAGTCTGAAACTTAAAGGAATTGACGGAAGGGCACACAATGGAGTGGAACCT
GCGGCTTAATTTGACTCAACTCGGGAAAACTTACCAAGCTCAGATATAATAAGGATTGACAGACTAAAAGATCTTTC
ATGATTGTATAAGTGGTGGTGCATGGTCGTTCTTAGTTGGTGGAGTGATTTGTCTGGTCAATTCCGATAACGGACGAG
ACCTCTACTTACTAACTAGTGGTATTTATTTGGTCACTATGGGAGATAGTCATTTGGTGTTGGTAGTTAGGCCGTTAAA
AGTTTAGCTATTCTTCATTGAGTGGTGTGTATTCTGATCAGATAGGTACTAATTTAAAAAATAAACTTCTTAGAAGGAC
TACCTACCTCAAGTGGGGGGAAGTCGGAGGCAATAACAGGTCTGTGATGCCCTTAGATACCTTGGGCTGCACGCGC
GTTACAATGTAGGCGAGAAAAAGGTTTCCGGAATCGAAAGGTTTCGGTAATCATTTGAATTGCCTACGTAATGGGGA
TTAATTTTTGTAATTATCGATTATCAACGAGGAATTCCTTGTAAGCGTAAATCATTACTTTACGCTGAATATGTCCCTGC
CCTTTGTACACACCGCCCGTCGCTCCTACCGATCGAACGATTAGGTAAAACTGACGGACTGGATGACTTTTTCGCAA
GGATTTGTCGTTTGGGAAGTTAGTTAAATCTCATTGTTTAGAGGAAGGAGAAGTCGTAACAAGGTATCCGTAGGTGA
ACCTGCAGAAGGATCAAGCTTGGATCCCGGG

>AM168103.1 *Heterostelium pallidum* TNS-C-98
AACCTGGTTGATCCTGCCAGTAGTCATATGCTTGTCTCAAAGATTAAGCCATGCATGTCTAAGTATAACCTTTATACGG
TGAAACTGCAGACGGCTCATTACAACAGTGATAAACTAAAGAACTTCCGCGCTTCGGCGTCTTGGATAACCGCAGT
AAATCGGGGCTAATACATATAAACGAGAGGGTGAGCGGGCAACTGCGAACCTTTGCGATTGTTAGCTATCTTTTTCA
CCAACCTCTTCGGAGTTTGTGGTGAATCCGAACAATATTGCTGATCGAAAATTTATTTTCGACGAGTTCTTTGTGTCA
CTGCCCTATCAACTTTCGATGGTAAGGTATTGGCTTACCATGGTTGTAACGGGTGACGGGGAATCAGGGTTCGATTCC
GGAGAGGGAGCCTGAGAAATGGCTACCACTTCTACGGAAGGCAGCAGGCGCGCAAATTACTCAATCCCAATACGGG
GAAGTAGTGACAAAAAATACTAATGCCTTTCCATATTATGGGGGGCAATTGGAATAAGTACAACTTAAATCGCTTAGC
AAAAGTGATTGGAGGGCAAGTCTGGTGCCAGCAGCCGCGGTAATTCCAGCTCCAATAGCGTATACTAAATTTGTTGC
AGTTAAAAAGCTCGTAGTTGAGATTGAGATTTCTTGGGTTTAAGCCAGTCATAGTAGCTTTCGGGTTATTATGATTTC
GGTTAAAGCTTTTGAGTGGATTTTATTTTTCACTTTTAAAGTTACCAAGGGATTTCCAACTGCCCATGTAAGCTGGCA
ACAGTTTACAATCGGGTGATCTACTGTGAGAAAATTGTAGTGTTCAAAGCAGGCGTCTTACGTTTGTTCAATGCAGC

ATGGTATAGTAAAATATGACACTAAATATATGTTGGTTGTATATTCTTAGTGTAATGACTAATAGGGAAGGGCGGGGCC
GTTCATATTGATGGGCGAGAGGTGAAATTCGTTGACCCTATCAAGATGCACTACAGCGAAAGCATTCGGCAAGTACT
TCTCCATTAATCAAGAACGAAAGTTTGGGGATCGAAGACGATCAGATACCGTCGTAGTCCAAACCATAAACTATGTC
GACCAGGGATTGGACGGATAATTTTTAAAAACTCGCTCAGAACCTTGTGAGAAATCATGAGTGTTTGGACTCTGGG
GGGAGTATGGTCGCAAGTCTGAAACTTAAAGGAATTGACGGAAGGGCACACAATGGAGTGGAACCTGCGGCTTAAT
TTGACTCAACTCGGGAAAACTTACCAAGCTCAGATATAATAAGGATTGACAGACTAAAAGATCTTTCATGATTGTATA
AGTGGTGGTGCATGGTCGTTCTTAGTTGGTGGAGTGATTTGTCTGGTCAATTCCGATAACGGACGAGACCTCTACCT
GCTAACTAGTGGTATTTATTTGGTCAATATGGAAGATAGTCATTTGGTGTTGGTGTCAGGGTCAAACTTGGCATTCTTC
ATTGAGTGGTGTGTATTCTGGTCAGATAGGTACTAACTAAAAAAATAAACTTCTTAGAGGGACTACCTACCTCAAGT
GGGGGGAAGTCGGAGGCAATAACAGGTCTGTGATGCCCTTAGATACCTTGGGCTGCACGCGCGTTACAATGTAGAT
GAGAAAAAGGTTTCCGACATCGAAAGGTGCCGGTAATCAATTGAATTGTCTACGTAATGGGGATTAATTTTTGTAATT
ATCGATTATCAACGAGGAATTCCTTGTAAGCGTAAATCATTACTTTACGCTGAATATGTCCCTGCCCTTTGTACACACC
GCCCGTCGCTCCTACCGATCGAACGATTAGGTAAAACTGACGGATTAGATGATTTCCTCGCAAGGGGTTATCGTTTG
AGAAGTTAGTTAAATCTCATTGTTTAGAGGAAGGAGAAGTCGTAACAAGGTATCCGTAGGTGAACCTGCAGAAGGA
TCAA

>KP167483.1 *Heterostelium parvimigratum*
TGCATGTCTAAGTATAACCTTTATACGGTGAAACTGCAGACGGATCAATACAACAGTGATAAACTAAAGAACTTCCG
CGCTTCGGCGTTTTGGATAACCGCAGTAAATCGGGGCTAATACATGTAAACGAGAGGATGAACGGGCAACTGTGAG
TCTTTGCGATTGTTAGCTTTAATCACCAACCTCTTCGGAGTCAGTGGTGAATCCCTACAATATTGCTGATCGGAAACT
TGTTTCCGACGAGTTCTTTGTGTCACTGCCCTATCAACTTTCGATGGTAAGGTATTGGCTTACCATGGTTGTAACGGG
TGACGGGGAATCAGGGTTCGATTCCGGAGAGGGAGCCTGAGAAATGGCTACCACTTCTACGGAAGGCAGCAGGCG
CGCAAATTACTCAATCCCAATACGGGGAAGTAGTGACAAAAAATACTAATGCCTTTCCATTTATATGGGGGGCAATTG
GAATAAGTACAATTTAAATCGCTTAACGAAAGTGATTGGAGGGCAAGTCTGGTGCCAGCAGCCGCGGTAATTCCAG
CTCCAATAGCGTATACTAAATTTGTTGCAGTTAAAAAGCTCGTAGTTGAGATTGAGATTTCTCAGGTTTAGCGGTCGG
CATGCCTTCACGGGTTTTGCTTGATACGTAAAGCTTTATAGTAGAATTTCATTTTATTATTAAAGTTACTGAGGAATTTC
CAACTGCCCATGGTTGTCAGCAATGGCTTCCAATAGGGTGATCTACTGTGAGAAAATTGTAGTGTTCAAAGCAGGCG
TCTTTCGTTTGTTCAATGCAGCATGGTATAGTTAAATATGACACTAAATATATGTTGGTTGTATATTCTTAGTGTAATGA
CTAATAGGGAAGGGCGGGGCCGTTCATATTGATGGGCGAGAGGTGAAATTCGTTGACCCTATCAAGATGCACTACAG
CGAAAGCATTCGGCAAGTACTTCTCCATTAATCAAGAACGAAAGTTTGGGGATCGAAGACGATCAGATACCGTCGTA
GTCCAAACCATAAACTATGTCGACCAGGGATTGGACGGATAATTTTTAAAAACTCGCTCAGAACCTTGTGAGAAAT
CATGAGTGTTTGGACTCTGGGGGGAGTATGGTCGCAAGTCTGAAACTTAAAGGAATTGACGGAAGGGCACACAATG
GAGTGGAACCTGCGGCTTAATTTGACTCAACTCGGGAAAACTTACCAAGCTCAGATATAATAAGGATTGACAGACTA
AAAGATCTTTCATGATTGTATAAGTGGTGGTGCATGGTCGTTCTTAGTTGGTGGAGTGATTTGTCTGGTCAATTCCGA
TAACGGACGAGACCTCTACTTACTAACTAGTGATATTTATTTGGCCAATATGGGAGATAGTCATTTGGTGTTGGTAGTA
GGACGTCAAAATCTTGCTATTCTTCATTGAGTGGTGTGTATTCTGGTCAGATAGGTACTAATTTTAAAAATAAACTTCT
TAGAAGGACTACCTACCTCAAGTGGGGGGAAGTCGGAGGCAATAACAGGTCTGTGATGCCCTTAGATACCTTGGGC
TGCACGCGCGTTACAATGTAGGCGAGAAAAAGGTTTCCGGAATCGAAAGGTTTCGGTAATCAATTGAATTGCCTACG
TAATGGGGATTAATTTTTGTAATTATCGATTATCAACGAGGAATTCCTTGTAAGCGTAAATCATTACTTTACGCTGAATT
GTCCCTGCCCTTTGTACACACCGCCCGTCGCTCCTACCGATCGAACGATTAGGTAAAACTGACGGACTAGGCGACTC
TTTA

>KP167475.1 *Heterostelium plurimicrocystogenum*
CATGCATGTCTAAGTATAACCTTTATACGGTGAAACTGCAGACGGCTCAATACAACAGTGATAAACTAAAGAACTTT
CGCGCTTCGGCGTCTTGGATAACCGCAGTAAATCGGGGCTAATACATGTAAACGAGAGGATGAGCGGGCAACTGCG
AGTCTTTGCGATTGTTAGCTATTCACACACCAACCTCTTCGGAGTTTGTGGTGAGTCCGAACAATATTGCTGATCGGA
AATTTATTTCCGACAAGTTCTTTGTGTCACTGCCCTATCAACTTTCGATGGTAAGGTATTGGCTTACCATGGTTGTAAC
GGGTGACGGGGAATCAGGGTTCGATTCCGGAGAGGGAGCCTGAGAAATGGCTACCACTTCTATGGAAGGCAGCAG
GCGCGCAAATTACTCAATCCCAATACGGGGAAGTAGTGACAAAAAATACTAATGCCTTTCCATTTATATGGGGGGCA
ATTGGAATAAGTACAACTTAAATCGCTTAGCAAAAGTGATTGGAGGGCAAGTCTGGTGCCAGCAGCCGCGGTAATTC
CAGCTCCAATAGCGTATACTAAATTTGTTGCAGTTAAAAAGCTCGTAGTTGAGATTGAGATTTCTTGGGTTTAGCAGG
CATTGTCGCCTTCGGGTTGGCAATCGTTTGTAAAGCGCTATAATGGAATTTCACTTTCATTATATGTGTTACCAAGAAA
TTTCCAACTGCCCATGTCAAGTGGCAACACTTGTCTGATAGGGTGATCTACTGTGAGAAAATTGTAGTGTTCAAAGC
AGGCGTCTTACGTTTGTTCAATGCAGCATGGTATAGTAAAATAAGACACTAAATATATGTTGGTTGTATATTCTTAGTG
TAATGACTAATAGGGAAGGGCGGGGCCGTTCATATTGATGGGCGAGAGGTGAAATTCGTTGACCCTATCAAGATGCA
CTACAGCGAAAGCATTCGGCAAGTACTTCTCCATTAATCAAGAACGAAAGTTTGGGGATCGAAGACGATCAGATACC
GTCGTAGTCCAAACCATAAACTATGTCGACCAGGGATTGGACGGATAATTTTTAAAAACTCGCTCAGAACCTTGTG
AGAAATCATGAGTGTTTGGACTCTGGGGGGAGTATGGTCGCAAGTCTGAAACTTAAAGGAATTGACGGAAGGGCAC
ACAATGGAGTGGAACCTGCGGCTTAATTTGACTCAACTCGGGAAAACTTACCAAGCTCAGATATAATAAGGATTGAC
AGACTAAAAGATCTTTCATGATTGTATAAGTGGTGGTGCATGGTCGTTCTTAGTTGGTGGAGTGATTTGTCTGGTCAA
TTCCGATAACGGACGAGACCTCTACCTGCTAACTAGTGGTATTTATTTGGTCAATATGGGAGATAGCTATTTGGTGTTG
GCAGTCAGGGTCAAACTTGATTGTCCTTCATTGAGTGGTGTGTATTCTGATCAGATAGGTACTAACTAAAAAAATAAAC
TTCTTAGAGGGACTACCTACCTCAAGTGGGGGGAAGTCGGAGGCAATAACAGGTCTGTGATGCCCTTAGATACCTTG
GGCTGCACGCGCGTTACAATGTAGACGAGAAAAAGGTCTCCGGCACCGAAAGGTGTCGGTAATCATATGAATTGTC
TACGTAATGGGGATTAATTTTTGTAATTATCGATTATCAACGAGGAATTCCTTGTAAGCGTAAATCATTACTTTGCGCG
AATATGTCCCTGCCCTTTGTACACACCGCCCGTCGCTCCTACCGATCGAACGATTAGGTAAAACTGACAGACTGAGT
GGCTTCTCAGCA

>AM168107.1 *Heterostelium pseudocandidum* TNS-C-91
AACCTGGTTGATCCTGCCAGTAGTCATATGCTTGTCTCAAAGATTAAGCCATGCATGTCTAAGTATAACCTTTATACGG
TGAAACTGCAGACGGCTCATTACAACAGTGATAAACTAAAGAACTTCCGCGCTTCGGCGTTTTGGATAACCGCAGTA
AATCGGGGCTAATACATGTAAACGAGAGGATGAACGGGCAACTGTGAGTCTTTGCGATTGTTAGCTTTAATCACCAA
CCTCTTCGGAGTCAGTGGTGAATCCCTACAATATTGCTGATCGGAAACTTGTTTCCGACGAGTTCTTTGTGTCACTGC
CCTATCAACTTTCGATGGTAAGGTATTGGCTTACCATGGTTGTAACGGGTGACGGGGAATCAGGGTTCGATTCCGGA
GAGGGAGCCTGAGAAATGGCTACCACTTCTACGGAAGGCAGCAGGCGCGCAAATTACTCAATCCCAATACGGGGAA

GTAGTGACAAAAAATACTAATGCCTTTCCATTTATATGGGGGGCAATTGGAATAAGTACAATTTAAATCGCTTAACGA
AAGTGATTGGAGGGCAAGTCTGGTGCCAGCAGCCGCGGTAATTCCAGCTCCAATAGCGTATACTAAATTTGTTGCAG
TTAAAAAGCTCGTAGTTGAGATTGAGATTTCTCAGGTTTAGCGGTCAACATTGCCTTTACGGGTTAATGTTTGATACG
TAAAGCTTTATAGTAGAATTTCATTTTATTATTAAAGTTACTGAGGAATTTCCAACTGCCCATGGTAGCCAGCAATGGT
TATCAATAGGGTGATCTACTGTGAGAAAATTGTAGTGTTCAAAGCAGGCGTCTTTCGTTTGTTCAATGCAGCATGGTA
TAGTTAAATATGACACTAAATATATGTTGGTTGTATATCTTAGTGTAATGACTAATAGGGAAGGGCGGGGCCGTTCATA
TTGATGGGCGAGAGGTGAAATTCGTTGACCCTATCAAGATGCACTACAGCGAAAGCATTCGGCAAGTACTTCTCCAT
TAATCAAGAACGAAAGTTTGGGGATCGAAGACGATCAGATACCGTCGTAGTCCAAACCATAAACTATGTCGACCAG
GGATTGGACGGATAATTTTTTAAAAACTCGCTCAGAACCTTGTGAGAAATCATGAGTGTTTGGACTCTGGGGGGAGT
ATGGTCGCAAGTCTGAAACTTAAAGGAATTGACGGAAGGGCACACAATGGAGTGGAACCTGCGGCTTAATTTGACT
CAACTCGGGAAAACTTACCAAGCTCAGATATAATAAGGATTGACAGACTAAAAGATCTTTCATGATTGTATAAGTGGT
GGTGCATGGTCGTTCTTAGTTGGTGGAGTGATTTGTCTGGTCAATTCCGATAACGGACGAGACCTCTACTTACTAACT
AGTGATATTTATTTGGTCAATATGGGAGATAGTCATTTGGTGTTGGTAGTAGGACGTCAAAATCTTACTATTCTTCATT
GAGTGGTGTGTATTCTGATCAGATAGGTACTAATTTTAAAAATAAACTTCTTAGAAGGACTACCTACCTCAAGTGGGG
GGAAGTCGGAGGCAATAACAGGTCTGTGATGCCCTTAGATACCTTGGGCTGCACGCGCGTTACAATGTAGGCGAGA
AAAAGGTTTCCGGAATCGAAAGGTTTCGGTAATCAATTGAATTGCCTACGTAATGGGGATTAATTTTTGTAATTATCG
ATTATCAACGAGGAATTCCTTGTAAGCGTAAATCATTACTTTACGCTGAATATGTCCCTGCCCTTTGTACACACCGCCC
GTCGCTCCTACCGATCGAACGATTAGGTAAAACTGACGGACTAGGCGACTCTTCCGCAAGGATTTGTTGTTTGGGAA
GTTAGTTAAATCTCATTGTTTAGAGGAAGGAGAAGTCGTAACAAGGTATCCGTAGGTGAACCTGCAGAAGGATCAA

>KP167474.1 *Heterostelium pseudocolligatum*
ACGGTGGACACCTCGGCAGACGGGACACAGCAGGTTATAGATAAACTAAAGAACTTTCGCGCTTCGGCGTCTTGGA
TAACCGCAGTAAATCGGGGCTAATACATGTAAACGAGAGGATGAGCGGGCAACTGCGAGTCTTTGCGATTGTTAGCT
ATTCACACACCAACCTCTTCGGAGTTTGTGGTGAGTCCGAACAATATTGCTGATCGGAAATTTATTTCCGACAAGTTC
TTTGTGTCACTGCCCTATCAACTTTCGATGGTAAGGTATTGGCTTACCATGGTTGTAACGGGTGACGGGGAATCAGG
GTTCGATTCCGGAGAGGGAGCCTGAGAAATGGCTACCACTTCTATGGAAGGCAGCAGGCGCGCAAATTACTCAATC
CCAATACGGGGAAGTAGTGACAAAAAATACTAATGCCTTTCCATTTATATGGGGGGCAATTGGAATAAGTACAACTTA
AATCGCTTAGCAAAAGTGATTGGAGGGCAAGTCTGGTGCCAGCAGCCGCGGTAATTCCAGCTCCAATAGCGTATACT
AAATTTGTTGCAGTTAAAAAGCTCGTAGTTGAGATTGAGATTTCTTGGGTTTAGCAGGCATTGTCGCCTTCGGGTTG
GCAATCGTTTGTAAAGCGCTATAATGGAATTTCACTTTCATTATATGTGTTACCAAGAAATTTCCAACTGCCCATGTCA
AGTGGCAACACTTGTCTGATAGGGTGATCTACTGTGAGAAAATTGTAGTGTTCAAAGCAGGCGTCTTACGTTTGTTC
AATGCAGCATGGTATAGTAAAATAAGACACTAAATATATGTTGGTTGTATATTCTTAGTGTAATGACTAATAGGGAAGG
GCGGGGCCGTTCATATTGATGGGCGAGAGGTGAAATTCGTTGACCCTATCAAGATGCACTACAGCGAAAGCATTCGG
CAAGTACTTCTCCATTAATCAAGAACGAAAGTTTGGGGATCGAAGACGATCAGATACCGTCGTAGTCCAAACCATAA
ACTATGTCGACCAGGGATTGGACGGATAATTTTTTAAAAACTCGCTCAGAACCTTGTGAGAAATCATGAGTGTTTGG
ACTCTGGGGGGAGTATGGTCGCAAGTCTGAAACTTAAAGGAATTGACGGAAGGGCACACAATGGAGTGGAACCTG
CGGCTTAATTTGACTCAACTCGGGAAAACTTACCAAGCTCAGATATAATAAGGATTGACAGACTAAAAGATCTTTCAT
GATTGTATAAGTGGTGGTGCATGGTCGTTCTTAGTTGGTGGAGTGATTTGTCTGGTCAATTCCGATAACGGACGAGAC
CTCTACCTGCTAACTAGTGGTATTTATTTGGTCAATATGGGAGATAGCTATTTGGTGTTGGCAGTCAGGGTCAAACTT
GATTGTCCTTCATTGAGTGGTGTGTATTCTGATCAGATAGGTACTAACTAAAAAATAAACTTCTTAGAGGGACTACCT
ACCTCAAGTGGGGGGAAGTCGGAGGCAATAACAGGTCTGTGATGCCCTTAGATACCTTGGGCTGCACGCGCGTTAC
AATGTAGACGAGAAAAAGGTCTCCGGCACCGAAAGGTGTCGGTAATCATATGAATTGTCTACGTAATGGGGATTAAT
TTTTGTAATTATCGATTATCAACGAGGAATTCCTTGTAAGCGTAAATCATTACTTTGCGCTGAATATGTCCCTGCCCTTT
GTACACACCGCCCGTCGCTCCTACCGATCGAACGATTAGGTAAAACTGACAGACTGAGTGGCTCTGTAACCCTGGTT
GATCCCTGCCAGTAGTCATATGCTTGTCTCAAAGATAAGCCCATGCATTCTAA

>KP167482.1 *Heterostelium pseudoplasmodiofascium*
AACCTGGTTGATCCTGCCAGTAGTCATATGCTTGTCTCAAAGATTAAGCCATGCATGTCTAAGTATAACCTTTCTACG
GTGAAGCTGCAGAAGGATCAAGTACAACATGTAGATAAACTAAAGAACTTCCGCGCTTCGGCGTTTTGGATAACCG
CAGTAAATCGGGGCTAATACATGTAAACGAGAGGATGAACGGGCAACTGTGAGTCTTTGCGATTGTTAGCTTTAATC
ACCAACCTCTTCGGAGTCAGTGGTGAATCCCTACAATATTGCTGATCGGAAACTTGTTTCCGACGAGTTCTTTGTGTC
ACTGCCCTATCAACTTTCGATGGTAAGGTATTGGCTTACCATGGTTGTAACGGGTGACGGGGAATCAGGGTTCGATTC
CGGAGAGGGAGCCTGAGAAATGGCTACCACTTCTACGGAAGGCAGCAGGCGCGCAAATTACTCAATCCCAATACGG
GGAAGTAGTGACAAAAAATACTAATGCCTTTCCATTTATATGGGGGGCAATTGGAATAAGTACAATTTAAATCGCTTA
ACGAAAGTGATTGGAGGGCAAGTCTGGTGCCAGCAGCCGCGGTAATTCCAGCTCCAATAGCGTATACTAAATTTGTT
GCAGTTAAAAAGCTCGTAGTTGAGATTGAGATTTCTCAGGTTTAGCGGTCGGCATGCCTTCACGGGTTTTGCTTGAT
ACGTAAAGCTTTATAGTAGAATTTCATTTTATTATTAAAGTTACTGAGGAATTTCCAACTGCCCATGGTTGTCAGCAAT
GGCTTCCAATAGGGTGATCTACTGTGAGAAAATTGTAGTGTTCAAAGCAGGCGTCTTTCGTTTGTTCAATGCAGCAT
GGTATAGTTAAATATGACACTAAATATATGTTGGTTGTATATTCTTAGTGTAATGACTAATAGGGAAGGGCGGGGCCGT
TCATATTGATGGGCGAGAGGTGAAATTCGTTGACCCTATCAAGATGCACTACAGCGAAAGCATTCGGCAAGTACTTC
TCCATTAATCAAGAACGAAAGTTGGGGATCGAAGACGATCAGATACCGTCGTAGTCCAAACCATAAACTATGTCGA
CCAGGGATTGGACGGATAATTTTTTAAAAACTCGCTCAGAACCTTGTGAGAAATCATGAGTGTTTGGACTCTGGGGG
GAGTATGGTCGCAAGTCTGAAACTTAAAGGAATTGACGGAAGGGCACACAATGGAGTGGAACCTGCGGCTTAATTT
GACTCAACTCGGGAAAACTTACCAAGCTCAGATATAATAAGGATTGACAGACTAAAAGATCTTTCATGATTGTATAA
GTGGTGGTGCATGGTCGTTCTTAGTTGGTGGAGTGATTTGTCTGGTCAATTCCGATAACGGACGAGACCTCTACTTAC
TAACTAGTGATATTTATTTGGCCAATATGGGAGATAGTCATTTGGTGTTGGTAGTAGGACGTCAAAATCTTGCTATTCT
TCATTGAGTGGTGTGTATTCTGGTCAGATAGGTACTAATTTTAAAAATAAACTTCTTAGAAGGACTACCTACCTCAAG
TGGGGGGAAGTCGGAGGCAATAACAGGTCTGTGATGCCCTTAGATACCTTGGGCTGCACGCGCGTTACAATGTAGG
CGAGAAAAAGGTTTCCGGAATCGAAAGGTTTCGGTAATCAATTGAATTGCCTACGTAATGGGGATTAATTTTTGTAAT
TATCGATTATCAACGAGGAATTCCTTGTAAGCGTAAATCATTACTTTACGCTGAATATGTCCCTGCCCTTTGTACACAC
CGCCCGTCGCTCCTACCGATCGAACGATTAGGTAAAACTGACGGACTAGGCGACTCTCTGTACCTGGTTGATCCTGC
CAGTAGTCATATGCTGTCTCAAAGATAAGCCATGCA

>KP167472.1 *Heterostelium pseudoplasmodiomagnum*
GCCATGCATGTCTAAGTATAACCTTTATACGGTGAAACTGCAGACGGATCAATACAACAGTGATAAACTAAAGAACT

TTCGCGCTTCGGCGTCTTGGATAACCGCAGTAAATCGGGGCTAATACATGTAAACGAGAGGATGAGCGGGCAACTG
CGAGTCTTTGCGATTGTTAGCTATTCACACACCAACCTCTTCGGAGTTTGTGGTGAGTCCGAACAATATTGCTGATCG
GAAATTTATTTCCGACAAGTTCTTTGTGTCACTGCCCTATCAACTTTCGATGGTAAGGTATTGGCTTACCATGGTTGTA
ACGGGTGACGGGGAATCAGGGTTCGATTCCGGAGAGGGAGCCTGAGAAATGGCTACCACTTCTATGGAAGGCAGC
AGGCGCGCAAATTACTCAATCCCAATACGGGGAAGTAGTGACAAAAAATACTAATGCCTTTCCATTTATATGGGGGG
CAATTGGAATAAGTACAACTTAAATCGCTTAGCAAAAGTGATTGGAGGGCAAGTCTGGTGCCAGCAGCCGCGGTAA
TTCCAGCTCCAATAGCGTATACTAAATTTGTTGCAGTTAAAAAGCTCGTAGTTGAGATTGAGATTTCTTGGGTTTAGC
AGGCATTGTCGCCTTCGGGTTGGCAATCGTTTGTAAAGCGCTATAATGGAATTTCACTTTCATTATATGTGTTACCAAG
AAATTTCCAACTGCCCATGTCAAGTGGCAACACTTGTCTGATAGGGTGATCTACTGTGAGAAAATTGTAGTGTTCAA
AGCAGGCGTCTTACGTTTGTTCAATGCAGCATGGTATAGTAAAATAAGACACTAAATATATGTTGGTTGTATATTCTTA
GTGTAATGACTAATAGGGAAGGGCGGGGCCGTTCATATTGATGGGCGAGAGGTGAAATTCGTTGACCCTATCAAGAT
GCACTACAGCGAAAGCATTCGGCAAGTACTTCTCCATTAATCAAGAACGAAAGTTTGGGGATCGAAGACGATCAGA
TACCGTCGTAGTCCAAACCATAAACTATGTCGACCAGGGATTGGACGGATAATTTTTTAAAAACTCGCTCAGAACCT
TGTGAGAAATCATGAGTGTTTGGACTCTGGGGGGAGTATGGTCGCAAGTCTGAAACTTAAAGGAATTGACGGAAGG
GCACACAATGGAGTGGAACCTGCGGCTTAATTTGACTCAACTCGGGAAAACTTACCAAGCTCAGATATAATAAGGAT
TGACAGACTAAAAGATCTTTCATGATTGTATAAGTGGTGGTGCATGGTCGTTCTTAGTTGGTGGAGTGATTTGTCTGG
TCAATTCCGATAACGGACGAGACCTCTACCTGCTAACTAGTGGTATTTATTTGGTCAATATGGGAGATAGCTATTTGGT
GTTGGCAGTCAGGGTCAAACTTGATTGTCCTTCATTGAGTGGTGTGTATTCTGATCAGATAGGTACTAACTAAAAAAT
AAACTTCTTAGAGGGACTACCTACCTCAAGTGGGGGGAAGTCGGAGGCAATAACAGGTCTGTGATGCCCTTAGATA
CCTTGGGCTGCACGCGCGTTACAATGTAGACGAGAAAAAGGTCTCCGGCACCGAAAGGTGTCGGTAATCATATGAA
TTGTCTACGTAATGGGGATTAATTTTTGTAATTATCGATTATCAACGAGGAATTCCTTGTAAGCGTAAATCATTACTTTG
CGCTGAATATGTCCCTGCCCTTTGTACACACCGCCCGTCGCTCCTACCGATCGAACGATTAGGTAAAACTGACAGAC
TGAGTGGCTTCTAAC

>KP167476.1 *Heterostelium racemiferum*
AGCCATGCATGTCTAAGTATAACCTTTATACGGTGAACCTGCAGAAGGCTCATTACAACAGTGATAAACTAAAGAAC
TTTCGCGCTTCGGCGTCTTGGATAACCGCAGTAAATCGGGGCTAATACATGTAAACGAGAGGATGAGCGGGCAACTG
CGAGTCTTTGCGATTGTTAGCTATTCACACACCAACCTCTTCGGAGTTTGTGGTGAGTCCGAACAATATTGCTGATCG
GAAATTTATTTCCGACAAGTTCTTTGTGTCACTGCCCTATCAACTTTCGATGGTAAGGTATTGGCTTACCATGGTTGTA
ACGGGTGACGGGGAATCAGGGTTCGATTCCGGAGAGGGAGCCTGAGAAATGGCTACCACTTCTATGGAAGGCAGC
AGGCGCGCAAATTACTCAATCCCAATACGGGGAAGTAGTGACAAAAAATACTAATGCCTTTCCATTTATATGGGGGG
CAATTGGAATAAGTACAACTTAAATCGCTTAGCAAAAGTGATTGGAGGGCAAGTCTGGTGCCAGCAGCCGCGGTAA
TTCCAGCTCCAATAGCGTATACTAAATTTGTTGCAGTTAAAAAGCTCGTAGTTGAGATTGAGATTTCTTGGGTTTAGC
AGGCATTGTCGCCTTCGGGTTGGCAATCGTTTGTAAAGCGCTATAATGGGATTTCACTCTCATTATATGTGTTACCAAG
AAATTTCCAACTGCCCATGTCAAGTGGCAACACTTGTCTGATAGGGTGATCTACTGTGAGAAAATTGTAGTGTTCAA
AGCAGGCGTCTTACGTTTGTTCAATGCAGCATGGTATAGTAAAATATGACACTAAATATATGTTGGTTGTATATTCTTA
GTGTAATGACTAATAGGGAAGGGCGGGGCCGTTCATATTGATGGGCGAGAGGTGAAATTCGTTGACCCTATCAAGAT
GCACTACAGCGAAAGCATTCGGCAAGTACTTCTCCATTAATCAAGAACGAAAGTTTGGGGATCGAAGACGATCAGA
TACCGTCGTAGTCCAAACCATAAACTATGTCGACCAGGGATTGGACGGATAATTTTTTAAAAACTCGCTCAGAACCT
TGTGAGAAATCATGAGTGTTTGGACTCTGGGGGGAGTATGGTCGCAAGTCTGAAACTTAAAGGAATTGACGGAAGG
GCACACAATGGAGTGGAACCTGCGGCTTAATTTGACTCAACTCGGGAAAACTTACCAAGCTCAGATATAATAAGGAT
TGACAGACTAAAAGATCTTTCATGATTGTATAAGTGGTGGTGCATGGTCGTTCTTAGTTGGTGGAGTGATTTGTCTGG
TCAATTCCGATAACGGACGAGACCTCTACCTGCTAACTAGTGGTATTTATTTGGTCAATATGGGAGATAGCTATTTGGT
GTTGGCAGTCAGGGTCAAACTTGATTGTCCTTCATTGAGTGGTGTGTATTCTGATCAGATAGGTACTAACTAAAAAAT
AAACTTCTTAGAGGGACTACCTACCTCAAGTGGGGGGAAGTCGGAGGCAATAACAGGTCTGTGATGCCCTTAGATA
CCTTGGGCTGCACGCGCGTTACAATGTAGACGAGAAAAAGGTCTCCGGCACCGAAAGGTGTCGGTAATCATATGAA
TTGTCTACGTAATGGGGATTAATTTTTGTAATTATCGATTATCAACGAGGAATTCCTTGTAAGCGTAAATCATTACTTTG
CGCTGAATATGTCCCTGCCCTTTGTACACACCGCCCGTCGCTCCTACCGATCGAACGATTAGGTAAAACTGACAGAC
TGAATGGCTT

>MN338953.1 *Heterostelium radiatum* M26B
AACCTGGTTGATCCTGCCAGTAGTCATATGCTTGTCTCAAAGATTAAGCCATGCATGTCTAAGTATAACCTTTATACGG
TGAACTGAGAGGGACACGCCGGCAGTAGATAAACTAAAGAACTTTCGCGCTTCGGCGTCTTGGATAACCGCAGTAA
ATCGGGGCTAATACATATAAACGAGAGGATGAGCAGGCAACTGCGAGTCTTTGCGATTGTTAGCTATCTTTTTCACAC
AACCTCCTCGGAGTTTGTGGTGAATCCGAACAATATTGCTGATCGAAAATTTATTTTCGACGAGTTCTTTGTGTCACT
GCCCTATCAACTTTCGATGGTAAGGTATTGGCTTACCATGGTTGTAACGGGTGACGGGGAATCAGGGTTCGATTCCG
GAGAGGGAGCCTGAGAAATGGCTACCACTTCTACGGAAGGCAGCAGGCGCGCAAATTACTCAATCCCAATACGGGG
AAGTAGTGACAAAAAATACTAATGCCTTTCCATATTATGGGGGGCAATTGGAATAAGTACAACTTAAATCGCTTAGCA
AAAGTGATTGGAGGGCAAGTCTGGTGCCAGCAGCCGCGGTAATTCCAGCTCCAATAGCGTATACTAAATTTGTTGCA
GTTAAAAAGCTCGTAGTTGAGATTGAGATTTCTTGGGTTTAAGCCAGTTATAGTAGCTTTCGGGTTATTATAATTCGGT
TAAAGCTCTTGAGTGGATTTTATTTTTCACTTTTAGAGTTACCAAGGAATTTCCAACTGCCCATGTAGACTGGCAACA
GTTTACAATCGGGTGATCTACTGTGAGAAAATTGTAGTGTTCAAAGCAGGCGTCTTAAGCTTGTTCAATGCAGCATG
GTATAGTAAAATATGACACTAAATATATGTTGGTTGTATATTCTTAGTGTAATGACTAATAGGGAAGGGCGGGGCCGTT
CATATTGATGGGCGAGAGGTGAAATTCGTTGACCCTATCAAGATGCACTACAGCGAAAGCATTCGGCAAGTACTTCT
CCATTAATCAAGAACGAAAGTTTGGGGATCGAAGACGATCAGATACCGTCGTAGTCCAAACCATAAACTATGTCGAC
CAGGGATTGGACGGATAATTTTTTAAAAACTCGCTCAGAACCTTGTGAGAAATCATGAGTGTTTGGACTCTGGGGGG
AGTATGGTCGCAAGTCTGAAACTTAAAGGAATTGACGGAAGGGCACACAATGGAGTGGAACCTGCGGCTTAATTTG
ACTCAACTCGGGAAAACTTACCAAGCTCAGATATAATAAGGATTGACAGACTAATAAGATCTTTCATGATTGTATAAG
TGGTGGTGCATGGTCGTTCTTAGTTGGTGGAGTGATTTGTCTGGTCAATTCCGATAACGGACGAGACCTCTACCTGC
TAACTAGTGGTATTTATTTGGTCAATATGGAAGATAGTCATTTGGTGTTGGTGTTAGGTGTCAAAGCTTAGCATTCTTC
ATTGAGTGGTGTGTATTCTGGTCAGATAGGTACTAACTAAAAAATAAACTTCTTAGAGGGACTACCTACCTCAAGTG
GGGGGAAGTCGGAGGCAATAACAGGTCTGTGATGCCCTTAGATACCTTGGGCTGCACGCGCGTTACAATGTAGATG
AGAAAAAGGTTTCCGACGTCGAAAGGCGCCGGTAATCAATTGAATTGTCTACGTAATGGGGATTAATTTTTGTAATTA
TCGATTATCAACGAGGAATTCCTTGTAAGCGTAAATCATTACTTTGCGCTGAATATGTCCCTGCCCTTTGTACACACCG
CCCGTCGCTCCTACCGATCGAACGATTAGGTAAAACTGACGGATTAGATGATTTCTGGTGAACCCTGGTTTGGATTC

CCGCCCGTTGTTCCATTATGCCTGTTCTCCAAAGGAATTAGCCGGTGAACCTGCA

>HQ141501.1 *Heterostelium rotatum* QC2C
AACCTGGTTGATCCTGCCAGTAGTCATATGCTTGTCTCAAAGATTAAGCCATGCATGTCTAAGTATAACCTTTATACGG
TGAACCTGCAGAAGGATCAACTACAACATGTGATAAACTATAGAACTTTCGCGCTTCGGCGTCTTGGATAACCGCAG
TAAATCGGGGCTAATACATGTAAACGAAAGGATGAGCGGGCAACTGCGAGTCTTTGCGATTGTTAGCTTTCACACAC
CAACCTCTTCGGAGTTTGTGGTGAGTCCGAACAATATTGCTGATCGGAAACTTGTTTCCGACAAGTTCTTTGTGTCA
CTGCCCTATCAACTTTCGATGGTAAGGTATTGGCTTACCATGGTTGTAACGGGTGACGGGGAATCAGGGTTCGATTCC
GGAGAGGGAGCCTGAGAAATGGCTACCACTTCTACGGAAGGCAGCAGGCGCGCAAATTACTCAATCCCAATACGGG
GAAGTAGTGACAAAAAATACTAATGCCTTTCCATTTATATGGGGGGCAATTGGAATAAGTACAACTTAAATCGCTTAG
CAAAAGTGATTGGAGGGCAAGTCTGGTGCCAGCAGCCGCGGTAATTCCAGCTCCAATAGCGTATACTAAATTTGTTG
CAGTTAAAAAGCTCGTAGTTTAGACTGAGATTTCTTAGGTTTAGCGGTCGGCATTGCCTTAACCGGTTAATGTTTTGA
TTCGTAAAGCTTTATAGTAGAATTTCATTTTATTATTAAAGTTACTAAGGAGTTTCCAACTGCCCATGGTAGCTAGCAA
TAGTTATCAATCGGGTGATCCACTGTGAGAAAATTGTAGTGTTCAAAGCAGACGTCTTCGTTTGTTCAATGCAGCAT
GGTATGGTAAAATATGACACTAAATGTATGTTGGTTGTATATATTAGTGTAATGACTAATAGGGAAGGGCGGGGCCGTT
TATATTGATGGGCGAGAGGTGAAATTCGTTGACCCTATCAAGATACACAACAGCGAAAGCATTCGGCAAGTACTTCT
CCATTAATCAAGAACGAAAGTTTGGGGATCGAAGACGATCAGATACCGTCGTAGTCCAAACCATAAACTATGTCGAC
CAGGGATTGGACGGATAATTTTTTAAAAACTCGCTCAGAACCTTGTGAGAAATCATGAGTGTTTGGACTCTGGGGGG
AGTATGGTCGCAAGTCTGAAACTTAAAGGAATTGACGGAAGGGCACACAATGGAGTGGAACCTGCGGCTTAATTTG
ACTCAACTCGGGAAAACTTACCAAGCTCAGATATAATAAGGATTGACAGACTAAAAGATCTTTCATGATTGTATAAGT
GGTGGTGCATGGTCGTTCTTAGTTGGTGGAGTGATTTGTCTGGTCAATTCCGATAACGGACGAGACCTCTACTTACTA
ACTAGTGGTATTTATTTGGTCAATATGGGAGATAGTCATTTGGTGTTGGTAGTAGGCCGTCAAAAGTTTACTATTCTTC
ATTGAGTGGTGTGTATTCTGATCAGATAGGTACTAACAAAAAAAATAAACTTCTTAGAAGGACTACCTACCTCAAGT
GGGGGGAAGTCGGAGGCAATAACAGGTCTGTGATGCCCTTAGATACCTTGGGCTGCACGCGCGTTACAATGTAGGC
GAGAAAAAGGTTTCCGGAATCGAAAGGTTTCGGTAATCATTTGAATTGCCTACGTAATGGGGATTAATTTTTGTAATT
ATCGATTATCAACGAGGAATTCCTTGTAAGCGTAAATCATTACTTTACGCTGAATATGTCCCTGCCCTTTGTACACACC
GCCCGTCGCTCCTACCGATCGAACGATTAGGTAAAACTGACGGACTAGGCGATTTTTTAAGCCATGGTTGATCCTGG
TGAACCTGCAGAAGGATCAGCCAGTAGTCATATGCTTGTCTCAAAGATTAAGCCATGCATTCTA

>HQ141507.1 *Heterostelium stolonicoideum* K12A
AACCTGGTTGATCCTGCCAGTAGTCATATGCTTGTCTCAAAGATTAAGCCATGCATGTCTAAGTATAACCTTTATACGG
TGAACCTGCAGAAGGATCAACTCACGGCTTGTGATAAACTAAAGAACTTCCGCGCTTCGGCGTCTTGGATAACCGC
AGTAAATCGGGGCTAATACATATAAACGAGAGGGTGAGCGGGCAACTGCGAACCTTTGCGATTGTTAGCTATCACTC
ACCAACCTCTTCGGAGTTTGTGGTGAATCCGAACAATATTGCTGATCGAAAATTTATTTTCGACGAGTTCTTTGTGTC
ACTGCCCTATCAACTTTCGATGGTAAGGTATTGGCTTACCATGGTTGTAACGGGTGACGGGGAATCAGGGTTCGATTC
CGGAGAGGGAGCCTGAGAAATGGCTACCACTTCTACGGAAGGCAGCAGGCGCGCAAATTACTCAATCCCAATACGG
GGAAGTAGTGACAAAAAATACTAATGCCTTTCCATATTATGGGGGGCAATTGGAATAAGTACAACTTAAATCGCTTAG
CAAAAGTGATTGGAGGGCAAGTCTGGTGCCAGCAGCCGCGGTAATTCCAGCTCCAATAGCGTATACTAAATTTGTTG
CAGTTAAAAAGCTCGTAGTTGAGATTGAGATTTCTTGGGTTTAAGCCAGTCATAGTAGCTTTCGGGTTATTATGATTT
CGGTTAAAGCTTTTGAGTGGATTTTATTTTTCACTTTTAAAGTTACCAAGGGATTTCCAACTGCCCATGTAAGCTGGC
AACAGTTTACAATCGGGTGATCTACTGTGAGAAAATTGTAGTGTTCAAAGCAGGCGTCTTACGTTTGTTCAATGCAG
CATGGTATAGTAAAATATGACACTAAATATATGTTGGTTGTATATTCTTAGTGTAATGACTAATAGGGAAGGGCGGGGC
CGTTCATATTGATGGGCGAGAGGTGAAATTCGTTGACCCTATCAAGATGCACTACAGCGAAAGCATTCGGCAAGTAC
TTCTCCATTAATCAAGAACGAAAGTTTGGGGATCGAAGACGATCAGATACCGTCGTAGTCCAAACCATAAACTATGT
CGACCAGGGATTGGACGGATAATTTTTTAAAAACTCGCTCAGAACCTTGTGAGAAATCATGAGTGTTTGGACTCTGG
GGGGAGTATGGTCGCAAGTCTGAAACTTAAAGGAATTGACGGAAGGGCACACAATGGAGTGGAACCTGCGGCTTA
ATTTGACTCAACTCGGGAAAACTTACCAAGCTCAGATATAATAAGGATTGACAGACTAAAAGATCTTTCATGATTGTT
AAGTGGTGGTGCATGGTCGTTCTTAGTTGGTGGAGTGATTTGTCTGGTCAATTCCGATAACGGACGAGACCTCTACC
TGCTAACTAGTGGTATTTATTTGGTCAATATGGAAGATAGTCATTTGGTGTTGGTGTCAGGGTCAAACTTGGCATTCTT
CATTGAGTGGTGTGTATTCTGGTCAGATAGGTACTAACTAAAAAAATAAACTTCTTAGAGGGACTACCTACCTCAAGT
GGGGGGAAGTCGGAGGCAATAACAGGTCTGTGATGCCCTTAGATACCTTGGGCTGCACGCGCGTTACAATGTAGAT
GAGAAAAAGGTTTCCGACATCGAAAGGTGCCGGTAATCAATTGAATTGTCTACGTAATGGGGATTAATTTTTGTAATT
ATCGATTATCAACGAGGAATTCCTTGTAAGCGTAAATCATTACTTTACGCTGAATATGTCCCTGCCCTTTGTACACACC
GCCCGTCGCTCCTACCGATCGAACGATTAGGTAAAACTGACGGATTAGATGATTTCCTAACCTGGTTGATCCTGGTG
AACCTGCAGAAGGATCAGCCAGTAGTCATATGCTGTCTCAAAGATTAAGCCATG

>AM168105.1 *Heterostelium tenuissimum* TNS-C-97
AACCTGGTTGATCCTGCCAGTAGTCATATGCTTGTCTCAAAGATTAAGCCATGCATGTCTAAGTATAACCTTTATACGG
TGAAACTGCAGACGGCTCATTACAACAGTGATAAACTAAAGAACTTTCGCGCTTCGGCGTCTTGGATAACCGCAGTA
AATCGGGGCTAATACATGTAAACGAGAGGATGAGCAGGCAACTGCGAGTCTTTGCGATTGTTAGCTTTTCTTCACCA
ACCTCTTCGGAGTTTGTGGTGAGTCCGAACAATATTGCTGATCGGAAACTTGTTTCCGACGAGTTCTTTGTGTCACT
GCCCTATCAACTTTCGATGGTAAGGTATTGGCTTACCATGGTTGTAACGGGTGACGGGGAATCAGGGTTCGATTCCG
GAGAGGGAGCCTGAGAAATGGCTACCACTTCTACGGAAGGCAGCAGGCGCGCAAATTACTCAATCCCAATACGGGG
AAGTAGTGACAAAAAATACTAATGCCTTTCCATTTATATGGGGGGCAATTGGAATAAGTATAACTTAAATCGCTTAG
CAAAAGTGATTGGAGGGCAAGTCTGGTGCCAGCAGCCGCGGTAATTCCAGCTCCAATAGCGTATACTAAATTTGTTG
CAGTTAAAAAGCTCGTAGTTGAGATTGAGATTTTTAGGTTAGGGTCGGCATTGCCTTCGGGTTAGTGCTTTGATTCGT
AAAGCTTTGTAGTAGAATTTTTATTTTATTATTAAAGTTACTAAGGAATTTCCAACTGCCCAATTGTAATTAGCAATAAT
TACATTTAGGGTGATCTACTGTGAGAAAATTGTAGTGTTCAAAGCAGGCGTCTTACGTTTGTTCAATGCAGCATGGTA
TAGTAAAATATGACACTAAATATATGGTGGTTGTATATTCTTAGTGTAATGACTAATAGGGAAGGGCGGGGCCGTTCAT
ATTGATGGGCGAGAGGTGAAATTCGTTGACCCTATCAAGATGCACTACAGCGAAAGCATTCGGCAAGTACTTCTCCA
TTAATCAAGAACGAAAGTTTGGGGATCGAAGACGATCAGATACCCTCGTAGTCCAACCCATAAACTATGTCGACCAG
GGATTGGACGGATAATTTTTTAAAAACTCGCTCAGAACCTTGTGAGAAATCATGAGTGTTTGGACTCTGGGGGGAGT
ATGGTCGCAAGTCTGAAACTTAAAGGAATTGACGGAAGGGCACACAATGGAGTGGAACCTGCGGCTTAATTTGACT
CAACTCGGGAAAACTTACCAAGCTCAGATATAATAAGGATTGACAGACTAAAAGATCTTTCATGATTGTATAAGTGGT
GGTGCATGGTCGTTCTTAGTTGGTGGAGTGATTTGTCTGGTCAATTCCGATAACGGACGAGACCTCTACTTACTAACT

AGTGGTATTTATTTGGTCAATATGGGAGATAGTCATTTGGTGTTGGTAGTTAGACCGTTAAAAGTTTAGCTATTCTTCA
TTGAGTGGTGTGTATTCTGATCAGATAGGTACTAACTTAAAAAATAAACTTCTTAGAAGGACTACCTACCTCAAGTGG
GGGGAAGTCGGAGGCAATAACAGGTCTGTGATGCCCTTAGATACCTTGGGCTGCACGCGCGTTACAATGTAGGCGA
GAAAAAGGTTTCCGGAATCGAAAGGTTTCGGTAATCATTTGAATTGCCTACGTAATGGGGATTAATTTTTGTAATTAT
CGATTATCAACGAGGAATTCCTTGTAAGCGTAAATCATTACTTTACGCTGAATATGTCCCTGCCCTTTGTACACACCGC
CCGTCGCTCCTACCGATCGAACGATTAGGTAAAACTGACGGACTAGGCGACTTTTCCGCAAGGATTTGTTGTTTGGG
AAGTTAGTTAAATCTCATTGTTTAGAGGAAGGAGAAGTCGTAACAAGGTATCCGTAGGTGAACCTGCAGAAGGATC
AA

>AY040339.1 *Heterostelium tenuissimum*
TCATATGCTTGTCTCAAAGATTAAGCCTGCATGTCTAAGTATAACCTTTATACGGTGAAACTGCAGACGGCTCATTAC
AACAGTGATAAACTAAAGAACTTTCGCGCTTCGGCGTCTTGGATAACCGCAGTAAATCGGGGCTAATACATGTAAAC
GAAAGGATGAGCAGGCAACTGCGAGTCTTTGCGATTGTTAGCTTTCTTCACCAACCTCTTCGGAGTTTGTGGTGAA
TCCGAACAATATTGCTGATCGGAAACTTGTTTCCGACGAGTTCTTTGTGTCACTGCCCTATCAACTTTCGATGGTAAG
GTATTGGCTTACCATGGTTGTAACGGGTGACGGGGAATCAGGGTTCGATTCCGGAGAGGGAGCCTGAGAAATGGCT
ACCACTTCTACGGAAGGCAGCAGGCGCGCAAATTACTCAATCCCAATACGGGGAAGTAGTGACAAAAAATACTAAT
GCCTTTCCATTTATATGGGGGGCAATTGGAATAAGTACAACTTAAATCGCTTAGCAAAAGTGATTGGAGGGCAAGTC
TGGTGCCAGCAGCCGCGGAATTCCAGCTCCAATAGCGTATACTAAATTTGTTGCAGTTAAAAAGCTCGTAGTTGAGA
CAGAGATTTCTTAGGTTTAGGGTCGGCATTGCCTTCGGGTTAGTGCTTTGATTCGTAAAGCTTTGTAGTAGAATTTCA
TTTTATTATTAAAGTTACTAAGGAATTTCCAACTGCCCATGGTAGCTAGCAATAGTTATCAATCGGGTGATCTACTGTG
AGAAAATTGTAGTGTTCAAAGCAGGCCGTCTTACGTTTGTTCAATGCAGCATGGTATAGTAAAATATGACACTAAATA
TATGTTGGTTGTATATTCTTAGTGTAATGACTAATAGGGAAGGGCGGGGCCGTTCATATTGATGGGCGAGAGGTGAAA
TTCGTTGACCCTATCAAGATGCACTACAGCGAAAGCATTCGGCAAGTACTTCTCCATTAATCAAGAACGAAAGTTTG
GGGATCGAAGACGATCAGATACCGTCGTAGTCCAAACCATAAACTATGTCGACCAGGGATTGGACGGATAATTTTTT
AAAAACTCGCTCAGAACCTTGTGAGAAATCATGAGTGTTTGGACTCTGGGGGGAGTATGGTCGCAAGTCTGAAACT
TAAAGGAATTGACGGAAGGGCACACAATGGAGTGGAACCTGCGGCTTAATTTGACTCAACTCGGGAAAACTTACCA
AGCTCAGATATAATAAGGATTGACAGACTAAAAGATCTTTCATGATTGTATAAGTGGTGGTGCATGGTCGTTCTTAGT
TGGTGGAGTGATTTGTCTGGTCAATTCCGATAACGGACGAGACCTCTACTTACTAACTAGTGGTATTTATTTGGTCAA
TATGGGAGATAGTCATTTGGTGTTGGTAGTTAGACCGTTAAAAGTTTAGCTATTCTTCATTGAGTGGTGTGTATTCTGA
TCAGATAGGTACTAACTTAAAAAATAAACTTCTTAGAAGGACTACCTACTCAAGTGGGGGGAAGTCGGAGGCAATA
ACAGGTCTGTGATGCCCTTAGATACCTTGGGCTGCACGCGCGTTACAATGTAGGCGAGAAAAAGGTTTCCGGAATCG
AAAGGTTTCGGTAATCATTTGAATTGCCTACGTAATGGGGATTAATTTTTGTAATTATCGATTATCAACGAGGAATTCC
TTGTAAGCGTAAATCATTACTTTACGCTGAATATGTCCCTGCCCTTTGTACACACCGCCCGTCGCTCCTACCGATCGA
ACGATTAGGTAAAACTGACGGATTAGATGATTTCCTCGCAAGGGGTTATTGTTTGAGAAGTTAGTTAAATCTCATTGT
TTAGAGGAAGGAGAAGTCGTAACAAGGTATCCGTAGTGAACCTGCGGATGGATCATTTC

>AM168106.1 *Heterostelium tikalense* OH595
AACCTGGTTGATCCTGCCAGTAGTCATATGCTTGTCTCAAAGATTAAGCCATGCATGTCTAAGTATAACCTTTATACGG
TGAAACTGCAGACGGCTCATTACAACAGTGATAAACTAAAGAACTTTCGCGCTTCGGCGTCTTGGATAACCGCAGTA
AATCGGGGCTAATACATGTAAACGAGAGGATGAGCGGGCAACTGCGAGTCTTTGCGATTGTTAGCTATTCACACACC
AACCTCTTCGGAGTTTGTGGTGAGTCCGAACAATATTGCTGATCGGAAATTTATTTCCGACAAGTTCTTTGTGTCACT
GCCCTATCAACTTTCGATGGTAAGGTATTGGCTTACCATGGTTGTAACGGGTGACGGGGAATCAGGGTTCGATTCCG
GAGAGGGAGCCTGAGAAATGGCTACCACTTCTATGGAAGGCAGCAGGCGCGCAAATTACTCAATCCCAATACGGGG
AAGTAGTGACAAAAAATACTAATGCCTTTCCATTTATATGGGGGGCAATTGGAATAAGTACAACTTAAATCGCTTAGC
AAAAGTGATTGGAGGGCAAGTCTGGTGCCAGCAGCCGCGGTAATACCAGCTCCAATAGCGTATACTAAATTTGTTGC
AGTTAAAAAGCTCGTAGTTGAGATTGAGATTTCTTGGGTTTAGCAGGCATTGTCGCCTTCGGGTTGGCAATCGTTTG
TAAAGCGCTATAATGGAATTTCACTTTCATTATATGTGTTACCAAGAAATTTCCAACTGCCCATGTCAAGTGGCAACA
CTTGTCTGATAGGGTGATCTACTGTGAGAAAATTGTAGTGTTCAAAGCAGGCGTCTTACGTTTGTTCAATGCAGCATG
GTATAGTAAAATATGACACTAAATATATGTTGGTTGTATATTCTTAGTGTAATGACTAATAGGGAAGGGCGGGGCCGTT
CATATTGATGGGCGAGAGGTGAAATTCGTTGACCCTATCAAGATGCACTACAGCGAAAGCATTCGGCAAGTACTTCT
CCATTAATCAAGAACGAAAGTTTGGGGATCGAAGACGATCAGATACCGTCGTAGTCCAAACCATAAACTATGTCGAC
CAGGGATTGGACGGATAATTTTTTAAAAACTCGCTCAGAACCTTGTGAGAAATCATGAGTGTTTGGACTCTGGGGGG
AGTATGGTCGCAAGTCTGAAACTTAAAGGAATTGACGGAAGGGCACACAATGGAGTGGAACCTGCGGCTTAATTTG
ACTCAACTCGGGAAAACTTACCAAGCTCAGATATAATAAGGATTGACAGACTAAAAGATCTTTCATGATTGTATAAGT
GGTGGTGCATGGTCGTTCTTAGTTGGTGGAGTGATTTGTCTGGTCAATTCCGATAACGGACGAGACCTCTACCTGCT
AACTAGTGGTATTTATTTGGTCAATATGGGAGATAGCTATTTGGTGTTGGCAGTCAGGGTCAAACTTGATTGTCCTTC
ATTGAGTGGTGTGTATTCTGATCAGATAGGTACTAACTAAAAAATAAACTTCTTAGAGGGACTACCTACCTCAAGTGG
GGGGAAGTCGGAGGCAATAACAGGTCTGTGATGCCCTTAGATACCTTGGGCTGCACGCGCGTTACAATGTAGACGA
GAAAAAGGTCTCCGGCACCGAAAGGTGTCGGTAATCATATGAATTGTCTACGTAATGGGGATTAATTTTTGTAATTAT
CGATTATCAACGAGGAATTCCTTGTAAGCGTAAATCATTACTTTGCGCTGAATATGTCCCTGCCCTTTGTACACACCG
CCCGTCGCTCCTACCGATCGAACGATTAGGTAAAACTGACAGACTGAGTGACTTCCCGCAAGGGATTGTTGTTTGGA
AAGTTAGTTAAATCTCATTGTTTAGAGGAAGGAGAAGTCGTAACAAGGTATCCGTAGGTGAACCTGCAGAAGGATC
AA

>HQ141509.1 *Heterostelium tikalense* HN1C1
AACCTGGTTGATCCTGCCAGTAGTCATATGCTTGTCTCAAAGATTAAGCCATGCATGTCTAAGTATAACCTTTATACGG
TGAACCTGCAGAAGGATCAACTACGAGCATGTAGATAAACTAAAGAACTTTCGCGCTTCGGCGTCTTGGATAACCGC
AGTAAATCGGGGCTAATACATGTAAACGAGAGGATGAGCGGGCAACTGCGAGTCTTTGCGATTGTTAGCTATTCACA
CACCAACCTCTTCGGAGTTTGTGGTGAGTCCGAACAATATTGCTGATCGGAAATTTATTTCCGACAAGTTCTTTGTGT
CACTGCCCTATCAACTTTCGATGGTAAGGTATTGGCTTACCATGGTTGTAACGGGTGACGGGGAATCAGGGTTCGATT
CCGGAGAGGGAGCCTGAGAAATGGCTACCACTTCTATGGAAGGCAGCAGGCGCGCAAATTACTCAATCCCAATACG
GGGAAGTAGTGACAAAAAATACTAATGCCTTTCCATTTATATGGGGGGCAATTGGAATAAGTACAACTTAAATCGCTT
AGCAAAAGTGATTGGAGGGCAAGTCTGGTGCCAGCAGCCGCGGTAATTCCAGCTCCATAGCGTATACTAAATTTGTT
GCAGTTAAAAAGCTCGTAGTTGAGATTGAGATTTCTTGGGTTTAGCAGGCATTGTCGCCTTCGGGTTGGCAATCGTT
TGTAAAGCGCTATAATGGAATTTCACTTTCATTATATGTGTTACCAAGAAATTTCCAACTGCCCATGTCAAGTGGCAA

CACTTGTCTGATAGGGTGATCTACTGTGAGAAAATTGTAGTGTTCAAAGCAGGCGTCTTACGTTTGTTCAATGCAGC
ATGGTATAGTAAAATATGACACTAAATATATGTTGGTTGTATATTCTTAGTGTAATGACTAATAGGGAAGGGCGGGGCC
GTTCATATTGATGGGCGAGAGGTGAAATTCGTTGACCCTATCAAGATGCACTACAGCGAAAGCATTCGGCAAGTACT
TCTCCATTAATCAAGAACGAAAGTTTGGGGATCGAAGACGATCAGATACCGTCGTAGTCCAAACCATAAACTATGTC
GACCAGGGATTGGACGGATAATTTTTTAAAAACTCGCTCAGAACCTTGTGAGAAATCATGAGTGTTTGGACTCTGGG
GGGAGTATGGTCGCAAGTCTGAAACTTAAAGGAATTGACGGAAGGGCACACAATGGAGTGGAACCTGCGGCTTAAT
TTGACTCAACTCGGGAAAACTTACCAAGCTCAGATATAATAAGGATTGACAGACTAAAAGATCTTTCATGATTGTATA
AGTGGTGGTGCATGGTCGTTCTTAGTTGGTGGAGTGATTTGTCTGGTCAATTCCGATAACGGACGAGACCTCTACCT
GCTAACTAGTGGTATTTATTTGGTCAATATGGGAGATAGCTATTTGGTGTTGGCAGTCAGGGTCAAACTTGATTGTCC
TTCATTGAGTGGTGTGTATTCTGATCAGATAGGTACTAACTAAAAAATAAACTTCTTAGAGGGACTACCTACCTCAAG
TGGGGGGAAGTCGGAGGCAATAACAGGTCTGTGATGCCCTTAGATACCTTGGGCTGCACGCGCGTTACAATGTAGA
CGAGAAAAAGGTCTCCGGCACCGAAAGGTGTCGGTAATCATATGAATTGTCTACGTAATGGGGATTAATTTTTGTAAT
TATCGATTATCAACGAGGAATTCCTTGTAAGCGTAAATCATTACTTTGCGCTGAATATGTCCCTGCCCTTTGTACACAC
CGCCCGTCGCTCCTACCGATCGAACGATTAGGTAAAACTGACAGACTGAGTGACTTCGGTGAACCTGCAGAAGGAT
CA

>KP167473.1 *Heterostelium unguliferum*
AGCCATGCATGTCTAAGTATAACCTTTATACGGTGAAACTGCAGACGGATCAATACAACAGTGATAAACTAAAGAAC
TTTCGCGCTTCGGCGTCTTGGATAACCGCAGTAAATCGGGGCTAATACATGTAAACGAGAGGATGAGCGGGCAACTG
CGAGTCTTTGCGATTGTTAGCTATTCACACACCAACCTCTTCGGAGTTTGTGGTGAGTCCGAACAATATTGCTGATCG
GAAATTTATTTCCGACAAGTTCTTTGTGTCACTGCCCTATCAACTTTCGATGGTAAGGTATTGGCTTACCATGGTTGTA
ACGGGTGACGGGGAATCAGGGTTCGATTCCGGAGAGGGAGCCTGAGAAATGGCTACCACTTCTATGGAAGGCAGC
AGGCGCGCAAATTACTCAATCCCAATACGGGGAAGTAGTGACAAAAAATACTAATGCCTTTCCATTTATATGGGGGG
CAATTGGAATAAGTACAACTTAAATCGCTTAGCAAAAGTGATTGGAGGGCAAGTCTGGTGCCAGCAGCCGCGGTAA
TTCCAGCTCCAATAGCGTATACTAAATTTGTTGCAGTTAAAAAGCTCGTAGTTGAGATTGAGATTTCTTGGGTTTAGC
AGGCATTGTCGCCTTCGGGTTGGCAATCGTTTGTAAAGCGCTATAATGGAATTTCACTTTCATTATATGTGTTACCAAG
AAATTTCCAACTGCCCATGTCAAGTGGCAACACTTGTCTGATAGGGTGATCTACTGTGAGAAAATTGTAGTGTTCAA
AGCAGGCGTCTTACGTTTGTTCAATGCAGCATGGTATAGTAAAATAAGACACTAAATATATGTTGGTTGTATATTCTTA
GTGTAATGACTAATAGGGAAGGGCGGGGCCGTTCATATTGATGGGCGAGAGGTGAAATTCGTTGACCCTATCAAGAT
GCACTACAGCGAAAGCATTCGGCAAGTACTTCTCCATTAATCAAGAACGAAAGTTTGGGGATCGAAGACGATCAGA
TACCGTCGTAGTCCAAACCATAAACTATGTCGACCAGGGATTGGACGGATAATTTTTTAAAAACTCGCTCAGAACCT
TGTGAGAAATCATGAGTGTTTGGACTCTGGGGGGAGTATGGTCGCAAGTCTGAAACTTAAAGGAATTGACGGAAGG
GCACACAATGGAGTGGAACCTGCGGCTTAATTTGACTCAACTCGGGAAAACTTACCAAGCTCAGATATAATAAGGAT
TGACAGACTAAAAGATCTTTCATGATTGTATAAGTGGTGGTGCATGGTCGTTCTTAGTTGGTGGAGTGATTTGTCTGG
TCAATTCCGATAACGGACGAGACCTCTACCTGCTAACTAGTGGTATTTATTTGGTCAATATGGGAGATAGCTATTTGGT
GTTGGCAGTCAGGGTCAAACTTGATTGTCCTTCATTGAGTGGTGTGTATTCTGATCAGATAGGTACTAACTAAAAAAT
AAACTTCTTAGAGGGACTACCTACCTCAAGTGGGGGGAAGTCGGAGGCAATAACAGGTCTGTGATGCCCTTAGATA
CCTTGGGCTGCACGCGCGTTACAATGTAGACGAGAAAAAGGTCTCCGGCACCGAAAGGTGTCGGTAATCATATGAA
TTGTCTACGTAATGGGGATTAATTTTTGTAATTATCGATTATCAACGAGGAATTCCTTGTAAGCGTAAATCATTACTTTG
CGCTGAATATGTCCCTGCCCTTTGTACACACCGCCCGTCGCTCCTACCGATCGAACGATTAGGTAAAACTGACAGAC
TGAGTGGCTTCTAACC

>MN338954.1 *Heterostelium versatile* Mad52
ATGTCTAAGTATAACCTTTATACGGTGAAACTGCAGACGGCTCATTACAACAGTGATAAACTATAGAACTTTCGCGCT
TCGGCGTCTTGGATAACCGCAGTAAATCGGGGCTAATACATGTAAACGAAAGGATGAGCGGGCAACTGCGAATCTTT
GCGATTGTTAGCTTTCACACACCAACCTCTTCGGAGTTTGTGGTGAGTCCGAACAATATTGCTGATCGGAAACTTGT
TTCCGACAAGTTCTTTGTGTCACTGCCCTATCAACTTTCGATGGTAAGGTATTGGCTTACCATGGTTGTAACGGGTGA
CGGGGAATCAGGGTTCGATTCCGGAGAGGGAGCCTGAGAAATGGCTACCACTTCTACGGAAGGCAGCAGGCGCGC
AAATTACTCAATCCCAATACGGGGAAGTAGTGACAAAAAATACTAATGCCTTTCCATTTATATGGGGGGCAATTGGAA
TAAGTACAACTTAAATCGCTTAGCAAAAGTGATTGGAGGGCAAGTCTGGTGCCAGCAGCCGCGGTAATTCCAGCTC
CAATAGCGTATACTAAATTTGTTGCAGTTAAAAAGCTCGTAGTTTAGACTGAGATTTCTTAGGTTTAGCGGTCTGCAT
TGCTTTAATCGGTTAATGTTTGATTCGTAAAGCTTTATAGTAGAATTTCATTTTATTATTAAAGTTACTAAGGAATTTCC
AACTGCCCATGGTAGCCAGCAATGGTTATCAATCGGGCGATCCACTGTGAGAAAATTGTAGTGTTCAAAGCAGACGT
CTTCGTTTGTTCAATGCAGCATGGTATGGTAAAATATGACACTAAATGTATGTTGGTTGTATATATTAGTGTAATGACTA
ATAGGGAAGGGCGGGGCCGTTTATATTGATGGGCGAGAGGTGAAATTCGTTGACCCTATCAAGATACACAACAGCG
AAAGCATTCGGCAAGTACTTCTCCATTAATCAAGAACGAAAGTTTGGGGATCGAAGACGATCAGATACCGTCGTAGT
CCAAACCATAAACTATGTCGACCAGGGATTGGACGGATAATTTTTTAAAAACTCGCTCAGAACCTTGTGAGAAATCA
TGAGTGTTTGGACTCTGGGGGGAGTATGGTCGCAAGTCTGAAACTTAAAGGAATTGACGGAAGGGCACACAATGG
AGTGGAAACCTGCGGCTTTAATT

>KP167478.1 *Heterostelium violaceotypum*
AGCCATGCATGTCTAAGTATAACCTTTATACGGTGAAACTGCAGACGGCTCAATACAACAGTGATAAACTAAAGAAC
TTTCGCGCTTCGGCGTCTTGGATAACCGCAGTAAATCGGGGCTAATACATATAAACGAGAGGATGAGCAGGCAACTG
CGAGTCTTTGCGATTGTTAGCTATCTTTTCACACCAACCTCTTCGGAGTTTGTGGTGAATCCGAACAATATTGCTGAC
GAAAATTTATTTTCGACGAGTTCTTTGTGTCACTGCCCTATCAACTTTCGATGGTAAGGTATTGGCTTACCATGGTTGT
AACGGGTGACGGGGAATCAGGGTTCGATTCCGGAGAGGGAGCCTGAGAAATGGCTACCACTTCTACGGAAGGCAG
CAGGCGCGCAAATTACTCAATCCCAATACGGGGAAGTAGTGACAAAAAATACTAATGCCTTTCCATATTATGGGGGG
CAATTGGAATAAGTACAACTTAAATCGCTTAGCAAAAGTGATTGGAGGGCAAGTCTGGTGCCAGCAGCCGCGGTAA
TTCCAGCTCCAATAGCGTATACTAAATTTGTTGCAGTTAAAAAGCTCGTAGTTGAGATTGAGATTTCTTGGGTTTAAG
CCAGTCATAGTAGCTTTCGGGTTATTATGGTTCGGTTAAAGCTTTTGAGTGGATTTTATTTTTCACTTTTAAAGTTACC
AAGGAATTTCCAACTGCCCATGTAGACTGGCAACAGTTTACAATCGGGTGATCTACTGTGAGAAAATTGTAGTGTTC
AAAGCAGGCGTCTTACGTTTGTTCAATGCAGCATGGTATAGTAAAATATGACACTAAATATATGTTGGTTGTATATTCT
TAGTGTAATGACTAATAGGGAAGGGCGGGGCCGTTCATATTGATGGGCGAGAGGTGAAATTCGTTGACCCTATCAAA
TGCACTACAGCGAAAGCATTCGGCAAGTACTTCTCCATTAATCAAGAACGAAAGTTTGGGGATCGAAGACGATCAG
ATACCGTCGTAGTCCAAACCATAAACTATGTCGACCAGGGATTGGACGGATAATTTTTTAAAAACTCGCTCAGAACC

TTGTGAGAAATCATGAGTGTTTGGACTCTGGGGGGAGTATGGTCGCAAGTCTGAAACTTAAAGGAATTGACGGAAG
GGCACACAATGGAGTGGAACCTGCGGCTTAATTTGACTCAACTCGGGAAAACTTACCAAGCTCAGATATAATAAGG
ATTGACAGGCTAAAAGATCTTTCATGATTGTATAAGTGGTGGTGCATGGTCGTTCTTAGTTGGTGGAGTGAATTGTCT
GGTCAATTCCGATAACGGACGAGACCTCTACCTGCTAACTAGTGGTATTTATTTGGTCAATATGGAAGATAGTCATTT
GGTGTTGGTGTCAGGTGTCAAAGCTTGGCATTCTTCATTGAGTGGTGTGTATTCTGGTCAGATAGGTACTAACTAAA
AACATAACTTCTAGAGGGACTACCACCTCAGTGGGGGGAATCGAAGCATAACAGGCTGTGAGCCCTAAAACCTTGG
CTGCCCCGCGTACAATGAGATGAAAAAAGGTTCCGACTCGAAGGTGCCGTATCAATGGATTGCTACGAATGGGGATA
ATTTTGAATATCGATATCACGAGGATTCTTGAAGCGTAA

>MW857293.1 *Heterostelium recretum* 5756-1-17
CATGCATGTCTAAGTATAACCTTTATACGGTGAAACTGCAGACGGCTCATTACAACAGTGATAAACTAAAGAACTTCC
GCGCTTCGGCGTTTTGGATAACCGCAGTAAATCGGGGCTAATACATGTAAACGAGAGGATGAACGGGCAACTGTGA
GTCTTTGCGATTGTTAGCTTTAATCACCAACCTCTTCGGAGTCAGTGGTGAATCCCTACAATATTGCTGATCGGAAAC
TTGTTTCCGACGAGTTCTTTGTGTCACTGCCCTATCAACTTTCGATGGTAAGGTATTGGCTTACCATGGTTGTAACGG
GTGACGGGGAATCAGGGTTCGATTCCGGAGAGGGAGCCTGAGAAATGGCTACCACTTCTACGGAAGGCAGCAGGC
GCGCAAATTACTCAATCCCAATACGGGGAAGTAGTGACAAAAAATACTAATGCCTTTCCATTTATATGGGGGGCAATT
GGAATAAGTACAATTTAAATCGCTTAACGAAAGTGATTGGAGGGCAAGTCTGGTGCCAGCAGCCGCGGTAATTCCA
GCTCCAATAGCGTATACTAAATTTGTTGCAGTTAAAAAGCTCGTAGTTGAGATTGAGATTTCTCAGGTTTAGCGGTCA
ACATTGCCTTCACGGGTTAATGTTTGATACGTAAAGCTTTATAGTAGAATTTCATTTTATTATTAAAGTTACTGAGGAA
TTTCCAACTGCCCATGGTAGCCAGCAATGGTTATCAATAGGGTGATCTACTGTGAGAAAATTGTAGTGTTCAAAGCA
GGCGTCTTTCGTTTGTTCAATGCAGCATGGTATAGTTAAATATGACACTAAATATATGTTGGTTGTATATCTTAGTGTAA
TGACTAATAGGGAAGGGCGGGGCCGTTCATATTGATGGGCGAGAGGTGAAATTCGTTGACCCTATCAAGATGCACTA
CAGCGAAAGCATTCGGCAAGTACTTCTCCATTAATCAAGAACGAAAGTTTGGGGATCGAAGACGATCAGATACCGT
CGTAGTCCAAACCATAAACTATGTCGACCAGGGATTGGACGGATAATTTTTTAAAAACTCGCTCAGAACCTTGTGAG
AAATCATGAGTGTTTGGACTCTGGGGGGAGTATGGTCGCAAGTCTGAAACTTAAAGGAATTGACGGAAGGGCACAC
AATGGAGTGGAACCTGCGGCTTAATTTGACTCAACTCGGGAAAACTTACCAAGCTCAGATATAATAAGGATTGACAG
ACTAAAAGATCTTTCATGATTGTATAAGTGGTGGTGCATGGTCGTTCTTAGTTGGTGGAGTGATTTGTCTGGTCAATT
CCGATAACGGACGAGACCTCTACTTACTAACTAGTGATATTTATTTGGTCAATATGGGAGATAGTCATTTGGTGTTGGT
AGTAGGACGTCAAAATCTTACTATTCTTCATTGAGTGGTGTGTATTCTGATCAGATAGGTACTAATTTTAAAAATAAAC
TTCTTAGAAGGACTACCTACCTCAAGTGGGGGGAAGTCGGAGGCAATAACAGGTCTGTGATGCCCTTAGATACCTTG
GGCTGCACGCGCGTTACAATGTAGGCGAGAAAAAGGTTTCCGGAATCGAAAGGTTTCGGTAATCAATTGAATTGCC
TACGTAATGGGGATTAATTTTTGTAATTATCGATTATCAACGAGGAATTCCTTGTAAGCGTAAATCATTACTTTACGCT
GAATATGTCCCTGCCCTTTGTACACACCGCCCGTCGCTCCTACCGATCGAACGATTAGGTAAAACTGACGGACTAGG
CGACTCTTCCGCAAGGATTTGTTGTTTGGGAAGTTAGTTAAATCTCATTGTTAGAGGAAG

>MN752217.1 *Heterostelium multibrachiatum* 5916lun
TGCATGTCTAAGTATAACCTTTATACGGTGAAACTGCAGACGGCTCATTACAACAGTGATAAACTAAAGAACTTCCG
CGCTTCGGCGTTTTGGATAACCGCAGTAAATCGGGGCTAATACATGTAAACGAGAGGATGAACGGGCAACTGTGAG
TCTTTGCGATTGTTAGCTTTAATCACCAACCTCTTCGGAGTCAGTGGTGAATCCCTACAATATTGCTGATCGGAAACT
TGTTTCCGACGAGTTCTTTGTGTCACTGCCCTATCAACTTTCGATGGTAAGGTATTGGCTTACCATGGTTGTAACGGG
TGACGGGGAATCAGGGTTCGATTCCGGAGAGGGAGCCTGAGAAATGGCTACCACTTCTACGGAAGGCAGCAGGCG
CGCAAATTACTCAATCCCAATACGGGGAAGTAGTGACAAAAAATACTAATGCCTTTCCATTTATATGGGGGGCAATTG
GAATAAGTACAATTTAAATCGCTTAACGAAAGTGATTGGAGGGCAAGTCTGGTGCCAGCAGCCGCGGTAATTCCAG
CTCCAATAGCGTATACTAAATTTGTTGCAGTTAAAAAGCTCGTAGTTGAGATTGAGATTTCTCAGGTTTAGCGGTCAA
CATTGCCTTCACGGGTTAATGTTTGATACGTAAAGCTTTATAGTAGAATTTCATTTTATTATTAAAGTTACTGAGGAATT
TCCAACTGCCCATGGTAGCCAGCAATGGTTATCAATAGGGTGATCTACTGTGAGAAAATTGTAGTGTTCAAAGCAGG
CGTCTTTCGTTTGTTCAATGCAGCATGGTATAGTTAAATATGACACTAAATATATGTTGGTTGTATATCTTAGTGTAATG
ACTAATAGGGAAGGGCGGGGCCGTTCATATTGATGGGCGAGAGGTGAAATTCGTTGACCCTATCAAGATGCACTACA
GCGAAAGCATTCGGCAAGTACTTCTCCATTAATCAAGAACGAAAGTTTGGGGATCGAAGACGATCAGATACCGTCG
TAGTCCAAACCATAAACTATGTCGACCAGGGATTGGACGGATAATTTTTTAAAAACTCGCTCAGAACCTTGTGAGAA
ATCATGAGTGTTTGGACTCTGGGGGGAGTATGGTCGCAAGTCTGAAACTTAAAGGAATTGACGGAAGGGCACACAA
TGGAGTGGAACCTGCGGCTTAATTTGACTCAACTCGGGAAAACTTACCAAGCTCAGATATAATAAGGATTGACAGAC
TAAAAGATCTTTCATGATTGTATAAGTGGTGGTGCATGGTCGTTCTTAGTTGGTGGAGTGATTTGTCTGGTCAATTCC
GATAACGGACGAGACCTCTACTTACTAACTAGTGATATTTATTTGGTCAATATGGGAGATAGTCATTTGGTGTTGGTAG
TAGGACGTCAAAATCTTACTATTCTTCATTGAGTGGTGTGTATTCTGATCAGATAGGTACTAATTTTAAAAATAAACTT
CTTAGAAGGACTACCTACCTCAAGTGGGGGGAAGTCGGAGGCAATAACAGGTCTGTGATGCCCTTAGATACCTTGG
GCTGCACGCGCGTTACAATGTAGGCGAGAAAAAGGTTTCCGGAATCGAAAGGTTTCGGTAATCAATTGAATTGCCTA
CGTAATGGGGATTAATTTTTGTAATTATCGATTATCAACGAGGAATTCCTTGTAAGCGTAAATCATTACTTTACGCTGA
ATATGTCCCTGCCCTTTGTACACACCGCCCGTCGCTCCTACCGATCGAACGATTAGGTAAAACTGACGGACTAGGCG
ACTCTTCCGCAAGGATTTGTTGTTTGGGAAGTTAGTTAAATCTCATTGTTTAGAGGAAGGAGAAGTCGTAACAAGGT
ATCCGTAGGTGAACCTGCAGAAAGGATCAA

>*Heterostelium naviculare* JC SMA
CGGGTGACGGGGAATCAGGGTTCGATTCCGGAGAGGGAGCCTGAGAAATGGCTACCACTTCTACGGAAGGCAGCA
GGCGCGCAAATTACTCAATCCCAATACGGGGAAGTAGTGACAAAAAATACTAATGCCCTTCCATTTTTATGGGGGGC
AATTGGAATAAGTACAACTTAAATCGCTTAGCAAAAGTGATTGGAGGGCAAGTCTGGTGCCAGCAGCCGCGGTAAT
TCCAGCTCCAATAGCGTATACTAAATTTGTTGCAGTTAAAAAGCTCGTAGTTTAGATTGAGATTTTTTGGGTTTAAAG
CTCATCTTTTTGCTTAACGGTAATTAGGTGTAGTGTATAAAGCTTTTATGTAGACTTCGCGTTTATATATAAAGTTACCA
AAAAATTTCATTCTGCCCATACTAAAATTTATTTAGTAATCGGGTGTTCTACTGTGAGAAAATTGTAGTGTTTAAAGC
AGGCGTCTTTGTTTGTTCAATGCAGCATGGTATAGTAAAAAAAGACACTAAACATTTGTTGGTTAATGTTATAGTGTA
ATGACTAATAGGGAAGGGCGGGGCCGT

>JF892716.1 *Tieghemostelium angelicum* 38B0
AACCTGGTTGATCCTGCCAGTAGTCATATGCTTGTCTAAAGGATTAAGCCATGCATGTCTAAGTATAAATTTTTATACG
GTGAACCTGCAGACGGATCAACTCACGAGCATGTAGATAAACTAATAGAGTTTCGGGTCTAACCTACATGGATATCC

GCAGTAAATCGGGGCTAATACATACAAACGAGGGGTGACTGTTTACGGAAGCTCCGCGATTATTAGTCTAGCCAATA
CCCGCAAGGGTTTTGTGGTGAAACCGAATAATATTGCAGATCGAAATCTGATTTCGACAATTCTATTGTGTCACTGCC
CTATCAACTTTCGATGGTACGGTATTGGCCTACCATGGTTGTAACGGGTAACGGAGAATTAGGGTTCGATTCCGGAGA
GGGCGCCTGAGAAATGGCGACCACTTCTACGGAAGGCAGCAGGCGCGCAAATTACTCAATCCCAATACGGGGAAGT
AGTGACAATAAATATTAATGCCTATTCGTTTTTCGAAAGGTAATTAAAATGGGTACAAATTAAATCCATTAACTAATAC
AATTGGAGGGCAAGTCTGGTGCCAGCAGCCGCGGTAATTCCAGCTCCAATAGCATATACTAAAGTTGTTGCGGTTAA
AAAGCTCGTAGTTGAAACTAAATTTGCATTGGGTCAAAGTTTCTAGCCACTTTGGTGGTTACGAAATTCCAGTGCAT
TTTTTTAAATCTGCCCTTAAAGCCTCTACTTTGTAGTTGGTTTTCTTGGGTACTTCACTGTGAGAAAATTGTGGTGTTT
AAAGCGGGCGTCTCGCCTGATCTTTTGCAGCATGGTATGATAAAACATGACATTTTGTGCAATTGGTTGCATTTAAAG
TGTAATGATTAATAGGGATGGATGGGGGTGTTCATATTGGTGGGCGAGAGGTGAAATTCGTTGACCCTATCAAGATGA
ACTTCTGCGAAAGCATTCATCAAATACTTCCCCATTAATCAAGAACGAAAGTTTGGGGATCAAAGACGATCAGATAC
CGTCGTAGTCCAAACTATAAACTATGTCGACCAGGGATCGGTTAATATTTTTTAAAAATTTACTCGGCACCTTGTGAG
AAATCATGAGTGTTTAGATTCTGGGGGGAGTATGGTCGCAAGTCTGAAACTTAAAGGAATTGACGGAAGGGCACAC
AATGGAGTGGAGCCTGCGGCTTAATTTGACTCAACTCGGGAAAACTTACCAAGCTAAGATATAGTAAGGATTGACAG
ACTAAAAGATTTTTCATGATTCTATAAGTGGTGGTGCATGGTCGTTCTTAGTTGGTGGAGCAATCTGTCTGGTCAATT
CCGATAACGGACGAGATCTCGACCTGCTAACTAGTAGTACTTATTCGGTCGATATGAGTGAAGGCTCTCAGGGAGTA
AGTTGTGCGTCGAAAGGTGTATGACACTTCGGGGGGTTTGTAACTTGACCAGATATGTACGATTTTAAAAACTTCTTA
GAGGGACTACCTGTGGCAAACAGGGGGAAGTTCGAGGCAATAACAGGTCTGTGATGCCCTTAGATACCTTGGGCCG
CACGCGCGCTACAATGTAATAGGCAAAAAGCTCTCCTGGTCCGGAAGGATTGGGTAATCATATGAATTTATTACGTAA
CTGGGCTTGATCTTTGTAATTATTGATCATCAACGAGGAATTCCTTGTAAGCGCAAATCATTACTTTGCGCTGAATATG
TCCCTGCCCTTTGTACACACCGCCCGTCGCTCCTACCGATCGAATGATACGGTAAAGCCAACGGATAAGATCTTAACC
TGGTTGATCCTGCCAGTAGTCATATGCTTGTCTCAAAGATAAGCCGGAGAAGTCGTAACAAGGTATCCGTAGGTGAA
CCTGCAGAAGGATCA

>JF892722.1 *Tieghemostelium dumosum* OH602
ATAAACTAATAGAGTTTCGGGTCTAACCTACTGGATATCCGCAGTAAATCGGGGCTAATACATACAAACGAGGGGTG
ACTGTTTACGGGAGCTCCGCGATTATTAGTCTAGCCAATACCCGCAAGGGTTTTGTGGTGAAACCGAATAATATTGCA
GATCGAAATCTAATTTCGACAATTCTATTGTGTCACTGCCCTATCAACTTTCGATGGTACGGTATTGGCCTACCATGGT
TGTAACGGGTAACGGAGAATTAGGGTTCGATTCCGGAGAGGGCGCCTGAGAAATGGCGACCACTTCTACGGAAGGC
AGCAGGCGCGCAAATTACTCAATCCCAATACGGGGAAGTAGTGACAATAAATATTAATGCCTATTCGTTTTTCGAAAG
GTAATTAAAATGGGTACAAATTAAATCCCTTAACTAATACAATTGGAGGGCAAGTCTGGTGCCAGCAGCCGCGGTAA
TTCCAGCTCCAATAGCATATACTAAAGTTGTTGCGGTTAAAAAGCTCGTAGTTGAAACTAAATTTGTATTGGGTCCAA
GTTCCTAGCCACTTTGGTGGTTACGGAATTCCAGTGCATTTTTTTAAACCTGCCCTTAAAGCCTCTACTTTGTAGTTG
GTTTTCTTGGTACTTCACTGTGAGAAAATTGTGGTGTTCAAAGCGGGCGTCTCGCCTGATCTTTTGCAGCATGGTATG
ATAAAACATGACATTTTGTGCAATTGGTTGCATTTAAGTGTAATGATTAATAGGGATGGATGGGGGTGTTCATATTGGT
GGGCGAGAGGTGAAATTCGTTGACCCTATCAAGATGAACTTCTGCGAAAGCATTCATCAAATACTTCCCCATTAATC
AAGAACGAAAGTTTGGGGATCAAAGACGATCAGATACCGTCGTAGTCCAAACTATAAACTATGTCGACCAGGGATC
GGTTAATATTTTTTAAAAATTTACTCGGCACCTTGTGAGAAATCATGAGTGTTTAGATTCTGGGGGGAGTATGGTCGC
AAGTCTGAAACTTAAAGGAATTGACGGAAGGGCACACAATGGAGTGGAGCCTGCGGCTTAATTTGACTCAACTCGG
GAAAACTTACCAAGCTAAGATATAGTAAGGATTGACAGACTAAAAGATTTTTCATGATTCTATAAGTGGTGGTGCATG
GTCGTTCTTAGTTGGTGGAGCAATCTGTCTGGTCAATTCCGATAACGGACGAGATCTCGACCTGCTAACTAGTAGTAC
TTATTCGGTCGATATGAGTGAAGGCTCTCAAGGAGTAAGTTGTGCGTCGCAAGGTGCATGACACTTCGGGGGGTTTG
TAACTTGATCAGATATGTACGATTTTAAAAACTTCTTAGAGGGACTACCTGTGGCAAACAGGGGGAAGTTCGAGGCA
ATAACAGGTCTGTGATGCCCTTAGATACCTTGGGCCGCACGCGCGCTACAATGTAATAGGCAAAAAGCTCTCCTGGT
CCGGAAGGATTGGGTAATCATATGAATTTATTACGTAACTGGGCTTGATCTTTGTAATTATTGATCATCAACGAGGAAT
TCCTTGTAAGCGCAAATCATTACTTTGCGCTGAATATGTCCCTGCCCTTTGTACACACCGCCCGTCGCTCCTACCGAT
CGAATGATACGGTAAAGCCAACGGAAACCTCTGTAACCTGGTTGATCCTGCCAGTAGTCATATGCTTGTCTCAAAGT
AAGCCGGAGAAGTCGTAACAAGGTATCCGTAGG
TGAACCTGCAGAAGGATCA

>AM168045.1 *Tieghemostelium lacteum*
AACCTGGTTGATCCTGCCAGTAGTCATATGCTTGTCTCAAAGATTAAGCCATGCATGTCTAAGTATAAATCTTTATACG
GTGAAACTGCAGACGGCTCATTACAACAGTGATAAACTAATAGAGTTTCGGGTCTAACCTACCTGGATATCCGCAGT
AAATCGGGGCTAATACATACAAACGAGGGGTGACTGTTTACGGGAGCTCCGCGATTATTAGTCTAGCCAATGCCCGC
AAGGGTTTTGTGGTGAAACCGAATAATATTGCAGATCGAAATCTGATTTCGACAATTCTATTGTGTCACTGCCCTATC
AACTTTCGATGGTACGGTATTGGCCTACCATGGTTGTAACGGGTAACGGAGAATTAGGGTTCGATTCCGGAGAGGGC
GCCTGAGAAATGGCGACCACTTCTACGGAAGGCAGCAGGCGCGCAAATTACTCAATCCCAATACGGGGAAGTAGTG
ACAATAAATATTAATGCCTATTCGTTTTTCGAAAGGTAATTAAAATGGGTACAAATTAAATCCATTAACTAATACAATG
GGAGGGCAAGTCTGGTGCCAGCAGCCGCGGTAATTCCAGCTCCAATAGCATATACTAAAGTTGTTGCGGTTAAAAAG
CCCGTAGTTGAAATTAAATTTGCATTGGGCCAAAGTTTCTAGCCACTTTGGTGGTTACGAAATTCCAGTGCATTTTTTT
TAAACCTACCCTTAAAGCCTCTACTTTGTAGTTGGTTTTCTTGGGTACTTCACTGTGAGAAAATTGTGGTGTTTAAAG
CGGGCGTCTCGCCTGATCTTTTGCAGCATGGTATGATAAAACATGACATTTTGTGCAATTGGTTGCATTTAAAGTGTA
ATGATTAATAGGGATGGATGGGGGTGTTCATATTGGTGGGCGAGAGGTGAAATTCGTTGACCCTATCAAGATGAACTT
CTGCGAAAGCATTCATCAAATACTTCCCCATTAATCAAGAACGAAAGTTTGGGGATCAAAGACGACCAGATACCGTC
GTAGTCCAAACTATAAACTATGTCGACCAGGGATCGGTTAATATTTTTTAAAAATTTACTCGGCACCTTGTGAGAAAT
CATGAGTGTTTAGATTCTGGGGGGAGTATGGTCGCAAGTCTGAAACTTAAAGGAGTTGACGGAAGGGCACACAATG
GAGTGGAGCCTGCGGCTTAATTTGACTCAACTCGGGAAAACTTACCAAGCTAAGATATAGTAAGGATTGACAGACTA
AAAGATTTTTCATGATTCTATAAGTGGTGGTGCATGGTCGTTCTTAGTTGGTGGAGCAATCTGTCTGGTCAATTCTGA
TAACGGACGAGATCTCGACACGCTAACTAGTAGTACTTATTCGGTCGATATGAGTGAAGGCTCTCAAGGAGTAAGTT
GTGCGTCGAAAGGTGTATGACACTTCGGGGGGTTTGTAACTTGACCAGATATGTACGATTTTAAAAACTTCTTAGAG
GGACTACCTGTGGCAAACAGGGGGAAGTTCGAGGCAATAACAGGTCTGTGATGCCCTTAGATACCTTGGGCCGCAC
GCGCGCTACAATGTAATAGGCAAAAAGCTCTCCTGGTCCGGAAGGATTGGGTAATCATATGAATTTATTACGTAACTG
GGCTTGATCTTTGTAATTATTGATCATCAACGAGGAATTCCTTGTAAGCGCAAATCATTACTTTGTGCTGAATATGTCC
CTGCCCTTTGTACACACCGCCCGTCGCTCTTACCGATCGAATGATACGGTAAAGCCAACGGATAAGATTCTGTAGCA
ATACGTGAATTTTAAAAGTTGTTTAAATCTCATTGTTTAGAGGAAGGAGAAGTCGTAACAAGGTATCCGTAGGTGAA

CCTGCAGAAGGATCAA

>AM168073.1 *Tieghemostelium menorah* M1
AACCTGGTTGATCCTGCCAGTAGTCATATGCTTGTCTCAAAGATTAAGCCATGCATGTCTAAGTATAAATCTTTATACG
GTGAAACTGCAGACGGCTCATTACAACAGTGATAAACTAATAGAGTTTCGGGTCTAACCTACCTGGATATCCGCAGT
AAATCGGGGCTAATACATACAAACGAGGGGTGACTGTTTACGGAAGCTCCGCGATTATTAGATTAACCAATGCCCGC
AAGGGTTTTGTGGTGAAACCGAATAATATTGCAGATCGAAGCCTAGCTTCGACAATTCTATTGTGTCACTGCCCTATC
AACTTTCGATGGTACGGTATTGGCCTACCATGGTTTTTACGGGTAACGGAGAATTAGGGTTCGATTCCGGAGAGGGC
GCCTGAGAAATGGCGACCACTTCTATGGAAGGCAGCAGGCGCGCAAATTACTCAATCCCAATACGGGGAAGTAGTG
ACAATAAATATTGATGCCTATTCGTTTTTCGAAAGGTAATTGAAATGAGTACAAATTAAATCTCTTAACTAATACAATT
GGAGGGCAAGTCTGGTGCCAGCAGCCGCGGTAATTCCAGCTCCAATAGCATATACTAAAGTTGTTGCGGTTAAAAAG
CTCGTAGTTGAAAGTAAATTTACATTGGGTTAAAGTCCACGTCCGCTTTGGTGGTCGCTGGTCTCCATTGTATCTTTT
TAAACTTCACCCTTAAGGCCTCTACTTTGTAGCGGGTTCTTTTTAGGGTACTTCACTGTGAGAAAATTGTGGTGTTCA
AAGCGGGCGTCTCGCCTGATCTTTTGCAGCATGGTATGATGGAACAAGACATTTTGTGTAATTGGTTGCATTTAAAGT
GTAATGATTAATAGGGGTGGATGGGGGTGTTCATATTGGTGGGCGAGAGGTGAAATTCGCTGACCCTATCAAGATGA
ACTTCTGCGAAAGCATTCACCAAATACTCCCCCATTAATCAAGAACGAAAGTTTGGGGATCAAAGACGATCAGATAC
CGTCGTAGTCCAAACTATAAACTATGTCGACCAGGGATCGGTTACTATTTTTTAAAAATTTGATCGGCACCTTGTGAG
AAATCATGAGTGTTTAGATTCTGGGGGGAGTATGGTCGCAAGTCTGAAACTTAAAGGAATTGACGGAAGGGCACAC
AATGGAGTGGAGCCTGCGGCTTAATTTGACTCAACTCGGGAAAACTTACCAAGCTAAGATATAGTAAGGATTGACAG
ACTAAAAGATTTTTCATGATTCTATAAGTGGTGGTGCATGGTCGTTCTTAGTTGGTGGAGCAATCTGTCTGGTCAATT
CCGATAACGGACGAGATCTCGACCTGCTAACTAGTAGTACCTATTAATTCGATATGAATGAAAGCTCATTCGGAGTAA
GTTAGTCATCGCAAGGTGTCTGACACTTCGGTGGGTCGGTAATTTGATTTGATAGGTACGATATATAAACTTCTTAGA
GGGACTACCTGTGGCAAACAGGGGGAAGTTCGAGGCAATAACAGGTCTGTGATGCCCTTAGATACCTTGGGCCGCA
CGCGCGCTACAATGTAATAGGCAAAAAGCTATCCTGGTCCGGAAGGATTGGGTAATCATATGAATTTATTACGTAACT
GGGCTTGATCTTTGTAATTATTGATCATCAACGAGGAATTCCTTGTAAGCGCAAATCATTACTTTGTGCTGAATATGTC
CCTGCCCTTTGTACACACCGCCCGTCGCTCCTACCGATCGAATGATACGGTAAAGCCAACGGATAAGGCTCTGCAGC
AATGCGTGAGTTTCAAAAGTTGTTTAAATCTCATTGTTTAGAGGAAGGAGAAGTCGTAACAAGGTATCCGTAGGTGA
ACCTGCAGAAGGATCAA

>JF892717.1 *Tieghemostelium montium* 57a
GATAAACTAATAGAGTTTCGGGTCTAACCTACATGGATATCCGCAGTAAATCGGGGCTAATACATACAAACGAGGGGT
GACTGTTTACGGAAGCTCCGCGATTATTAGTCTAGCCAATACCCGCAAGGGTTTTGTGGTGAAACCGAATAATATTGC
AGATCGAAATCTGATTTCGACAATTCTATTGTGTCACTGCCCTATCAACTTTCGATGGTACGGTATTGGCCTACCATGG
TTGTAACGGGTAACGGAGAATTAGGGTTCGATTCCGGAGAGGGCGCCTGAGAAATGGCGACCACTTCTACGGAAGG
CAGCAGGCGCGCAAATTACTCAATCCCAATACGGGGAAGTAGTGACAATAAATATTAATGCCTATTCGTTTTTCGAAA
GGTAATTAAAATGGGTACAAATTAAATCCATTAACTAATACAATTGGAGGGCAAGTCTGGTGCCAGCAGCCGCGGTA
ATTCCAGCTCCAATAGCATATACTAAAGTTGTTGCGGTTAAAAAGCTCGTAGTTGAAACTAAATTTGCATTGGGTCAA
AGTTTCTAGCCACTTTGGTGGTTACGAAATTCCAGTGCATTTTTTTAAATCTGCCCTTAAAGCCTCTACTTTGTAGTTG
GTTTTCTTGGGTACTTCACTGTGAGAAAATTGTGGTGTTTAAAGCGGGCGTCTCGCCTGATCTTTTGCAGCATGGTAT
GATAAAACATGACATTTTGTGCAATTGGTTGCATTTAAAGTGTAATGATTAATAGGGATGGATGGGGGTGTTCATATTG
GTGGGCGAGAGGTGAAATTCGTTGACCCTATCAAGATGAACTTCTGCGAAAGCATTCATCAAATACTTCCCCATTAA
TCAAGAACGAAAGTTTGGGGATCAAAGACGATCAGATACCGTCGTAGTCCAAACTATAAACTATGTCGACCAGGGA
TCGGTTAATATTTTTTAAAAATTTACTCGGCACCTTGTGAGAAATCATGAGTGTTTAGATTCTGGGGGGAGTATGGTC
GCAAGTCTGAAACTTAAAGGAATTGACGGAAGGGCACACAATGGAGTGGAGCCTGCGGCTTAATTTGACTCAACTC
GGGAAAACTTACCAAGCTAAGATATAGTAAGGATTGACAGACTAAAAGATTTTTCATGATTCTATAAGTGGTGGTGC
ATGGTCGTTCTTAGTTGGTGGAGCAATCTGTCTGGTCAATTCCGATAACGGACGAGATCTCGACCTGCTAACTAGTA
GTACTTATTCGGTCGATATGAGTGAAGGCTCTCAGGGAGTAAGTTGTGCGTCGAAAGGTGTATGACACTTCGGGGGG
TTTGTAACTTGACCAGATATGTACGATTTTAAAAACTTCTTAGAGGGACTACCTGTGGCAAACAGGGGGAAGTTCGA
GGCAATAACAGGTCTGTGATGCCCTTAGATACCTTGGGCCGCACGCGCGCTACAATGTAATAGGCAAAAAGCTCTCC
TGGTCCGGAAGGATTGGGTAATCATATGAATTTATTACGTAACTGGGCTTGATCTTTGTAATTATTGATCATCAACGAG
GAATTCCTTGTAAGCGCAAATCATTACTTTGCGCTGAATATGTCCCTGCCCTTTGTACACACCGCCCGTCGCTCCTAC
CGATCGAATGATACGGTAAAGCCAACGGATAAGATCTGGTGAACCTGCGTTGTCCGGACCCGTTAGTTCGATTGGTG
AACCTGCAGAAGGATCA

>JF892720.1 *Tieghemostelium simplex* OH598
AACCTGGTTGATCCTGCCAGTAGTCATATGCTTGTCTCAAAGATTAAGCCATGCATGTCTAAGTATAAATCTTTATACG
GTGAACCTGCAGAAGGATCAACCCCGGTTTAGATAAACTAATAGAGTTTCGGGTCTAACCTACCTGGATATCCGCAGT
AAATCGGGGCTAATACATACAAACGAGGGGTGACTGTTTACGGGAGCTCCGCGATTATTAGTCTAGCCAATACCCGC
AAGGGTTTTGTGGTGAAACCGAATAATATTGCAGATCGAAATCTAATTTCGACAATTCTATTGTGTCACTGCCCTATC
AACTTTCGATGGTACGGTATTGGCCTACCATGGTTGTAACGGGTAACGGAGAATTAGGGTTCGATTCCGGAGAGGGC
GCCTGAGAAATGGCGACCACTTCTACGGAAGGCAGCAGGCGCGCAAATTACTCAATCCCAATACGGGGAAGTAGTG
ACAATAAATATTAATGCCTATTCGTTTTTCGAAAGGTAATTAAAATGGGTACAAATTAAATCCCTTAACTAATACAATT
GGAGGGCAAGTCTGGTGCCAGCAGCCGCGGTAATTCCAGCTCCAATAGCATATACTAAAGTTGTTGCGGTTAAAAAG
CTCGTAGTTGAAACTAAATTTGTATTGGGTCCAAGTTCCTAGCCACTTTGGTGGTTACGAATTCCAGTGCATTTTTTTA
AACCTGCCCTTAAAGCCTCTACTTTGTAGTTGTTTTCTTGGGTACTTCACTGTGAGAAAATTGTGGTGTTCAAAGCG
GGCGTCTCGCCTGATCTTTTGCAGCATGGTATGATAAAACATGACATTTTGTGCAATTGGTTGCATTTAAGTGTAATG
ATTAATAGGGATGGATGGGGGTGTTCATATTGGTGGGCGAGAGGTGAAATTCGTTGACCCTATCAAGATGAACTTCT
GCGAAAGCATTCATCAAATACTTCCCCATTAATCAAGAACGAAAGTTTGGGGATCAAAGACGATCAGATACCGTCGT
AGTCCAAACTATAAACTATGTCGACCAGGGATCGGTTAATATTTTTTAAAAATTTACTCGGCACCTTGTGAGAAATCA
TGAGTGTTTAGATTCTGGGGGGAGTATGGTCGCAAGTCTGAAACTTAAAGGAATTGACGGAAGGGCACACAATGGA
GTGGAGCCTGCGGCTTAATTTGACTCAACTCGGGAAAACTTACCAAGCTAAGATATAGTAAGGATTGACAGACTAAA
AGATTTTTCATGATTCTATAAGTGGTGGTGCATGGTCGTTCTTAGTTGGTGGAGCAATCTGTCTGGTCAATTCCGATAA
CGGACGAGATCTCGACCTGCTAACTAGTAGTACTTATTCGGTCGATATGAGTGAAGGCTCTCAAGGAGTAAGTTGTG
CGTCGCAAGGTGCATGACACTTCGGGGGGTTTGTAACTTGATCAGATATGTACGATTTTAAAAACTTCTTAGAGGGA
CTACCTGTGGCAAACAGGGGGAAGTTCGAGGCAATAACAGGTCTGTGATGCCCTTAGATACCTTGGGCCGCACGCG

CGCTACAATGTAATAGGCAAAAAGCTCTCCTGGTCCGGAAGGATTGGGTAATCATATGAATTTATTACGTAACTGGGC
TTGATCTTTGTAATTATTGATCATCAACGAGGAATTCCTTGTAAGCGCAAATCATTACTTTGCGCTGAATATGTCCCTG
CCCTTTGTACACACCGCCCGTCGCTCCTACCGATCGAATGATACGGTAAAGCCAACGTGAATCAAGATCTAACCTGG
TTGATCCTGCCAGTAGTCATATGCTTGTCTCAAAGATTAAGCCATGCATTCTAAGGGTGAACCTGCAGAAGGATCA

>JF892725.1 *Tieghemostelium unicornutum* OH599
AGATAAACTAATAGAGTTTCGGGTCTAACCTTTATGGATATCCGCAGTAAATCGGGGCTAATACATACAAACGAGGGG
TGACTGTTTACAGAAGCTTCGCGATTATTAGTCTAACCAATACCCGCAAGGGTCTAGTGGTGAAACCGAATAATATTG
CAGATCGAAACCTAGTTTCGACGATTCTATTGTGTCACTGCCCTATCAACTTTCGATGGTACGGTATTGGCCTACCATG
GTTGTAACGGGTAACGGGGAATTAGGGTTCGATTCCGGAGAGGGCGCCTGAGAAATGGCGACCACTTCTACGGAAG
GCAGCAGGCGCGCAAATTACTCAATCCCAATACGGGGAAGTAGTGACAATAAATATCAATGCCTATTCGTTTTTCGAA
AGGTAATTGAAATGGGTACAAATTAAACCCCTTAACCAATACAATTGGAGGGCAAGTCTGGTGCCAGCAGCCGCGG
TAATTCCAGCTCCAATAGCATATACTAAAGTTGTTGCGGTTAAAAAGCTCGTAGTTGAAACTAAATTTGTATTGGGCT
CAAGCTTGTAGCCACTTTGGTGGTTATCAAGTTCCAGTGCATTTTTTTGAAGCGGCCCTTAAAGCCTCTACCTCGTAG
TTGGTTTTCTTGGGTTTTTCACTGTGAGAAAATTGTGGTGTTCAAAGCGGGCGTTTCGCCTGATCTTTTGCAGCATGG
GATGATAAAACATGACATTCAATGCAATTGGTTGCATCTGAGTGTAATGATTAATAGGGATGGATGGGGGTGTTCATA
TTGGTGGGCGAGAGGTGAAATTCGTTGACCCTATCAAGATGAACTTCTGCGAAAGCATTCATCAAATACTTCCCCAT
TAATCAAGAACGAAAGTTTGGGGATCAAAGACGATCAGATACCGTCGTAGTCCAAACTATAAACTATGTCGACCAGG
GATCGGGTAGCATTTTTCAAAAATCTATTTGGCACCTTGTGAGAAATCATGAGTGTTTAGATTCTGGGGGGAGTATGG
TCGCAAGTCTGAAACTTAAAGGAATTGACGGAAGGGCACACAATGGAGTGGAGCCTGCGGCTTAATTTGACTCAAC
TCGGGAAAACTTACCAAGCTAAGATATAGTAAGGATTGACAGACTAAAAGATTTTTCATGATTCTATAAGTGGTGGTG
CATGGTCGTTCTTAGTTGGTGGAGCAATCTGTCTGGTCAATTCCGATAACGGACGAGATCTCGACCTGCTAACTAGTA
GTACGTATTTGGTCGATATGGGTGAAGGCTCTTTTGGAGTAAGTTGTGCGTCGCAAGGTGTACAGCACTTCTGGGGG
TTTGTAACCTGGTCAGATACGTACGATTAAAAAAACTTCTTAGAGGGACTACCTGTGGCAAACAGGGGGAAGTTCG
AGGCAATAACAGGTCTGTGATGCCCTTAGATACCTTGGGCCGCACGCGCGCTACAATGTAAAAGGCAAAAAGCTTT
CCTGGTCCGGAAGGACTGGGTAATCATCTGAATTTTTTACGTAACTGGGATTGATCTTTGTAATTATTGATCATCAACG
AGGAATTCCTTGTAAGCGCAAATCATTACTTTGTGCTGAATAGGTCCCTGCCCTTTGTACACACCGCCCGTCGCTCCT
ACCGATCGAATGATACGGTAAAGCCAACAGATACGGTCGGTGCGGCGGTGAACCTGCAGAAGGATCA

>AM168036.1 *Hagiwaraea coeruleostipes* CRLC53B
AACCTGGTTGATCCTGCCAGTAGTCATATGCTTGTCTCAAAGATTAAGCCATGCATGTCTAAGTATAAATTTTTATATG
ATGAAACTGCAGAAGGCTCATTACAACAGTGATAAACTACTAGACTTTCGGGCCTCGGCCTTTTGGATAACCGCAGT
AAATCGGGGCTAATACATACAATCGATGGGTGACTGTTTACGGAATCTCAGCGATTATTAGCTTTCAGCCAACACTCT
TCGGAGTTTTGGGTGAACCCGGATAATATTGCAGATCGAAGCTTACGCTTTCGACAAGTCTAATGTGTCACTGCCCTA
TCAACTTTCGATGGTACGGTATTGGCCTACCATGGTTGTAACGGGTAACGGGGAATTAGGGTTCGATTCCGGAGAGG
GCGCCTGAGAAATGGCGACCACTTCTACGGAAGGCAGCAGGCGCGCAAATTACTCAATCCCAATACGGGGAAGTAG
TGACAAGAAATATTAATGCCTATCCTTTCAAGGAAGGTAATTAAAATGGATTTAAACTAAACCCATTTTTTAATACAAT
TGGAGGGCAAGTCTGGTGCCAGCAGCCGCGGTAATTCCAGCTCCAATAGCATATACTAAATTTGTTGCGGTTAAAAA
GCTCGTAGTTGAAATTGAAATTATATTGGGTTCATAGGTTTTAGTCGACTTCGCGTCGGTTTAAAACTTCCAGTGTAA
ATTTTTAACTTTAATCCAGGTTGCTAGTTACTTTGTAATTAGTTTTCTTTGGATACTTCACTGTGAGGAAATTGTGGTG
CTTAAAGCAGGCGTTTTCGCTTGATCTTTTGCAGCATGGTATGATAGAATATGACATTTTATGTAGTTGGCTCGCATAA
AGTGTAATGATTAATAGGGATGGATGGGGGTGTTCATATTGATGGGCGAGAGGTGAAATTCGTTGACCCTATCAAGAT
GAACTTCTGCGAAAGCATTCACCAAATACTTCTCCATTAATCAAGAACGAAAGTTTGGGGATCAAAGACGATCAGAT
ACCGTCGTAGTCCAAACTATAAACTATGTCGACCAGGGATCAGTTACTATTTTTAAAAAATATAATTGGCACCTTGTG
AGAAATCATGAGTGTTTAGATTCTGGGGGGAGTATGGTCGCAAGTCTGAAACTTAAAGGAATTGACGGAAGGGCAC
ACAATGGAGTGGAGCCTGCGGCTTAATTTGACTCAACTCGGGAAAACTTACCAAGCTAAGATATAATAAGGATTGAC
AGACTAAAAGATCTTTCATGATTCTATAAGTGGTGGTGCATGGTCGTTCTTAGTTGGTGGAGCAATCTGTCTGGTCA
ATTCCGATAACGGACGAGATCTCGACCTGCTAACTAGTAGTACATATTGATTCGATATGCAAGAAAGATTTGTTGGAG
CAAGTTAAAGATCGAAAGGTTTTTAGCTCTTCGCTGGTTGAGTATTGTGGATTGATATAGTGCGATAAATTTAAAAAA
AACTTCTTAGAGGGACTACCTGTGGCAAACAGGGGGAAGTTCGAGGCAATAACAGGTCTGTGATGCCCTTAGATAC
CTTGGGCCGCACGCGCGCTACAATGTAGTACGCAAAATGTTCTAAAAACCAAACCCGGGAGGGTAAGGTAATCATAT
AATTTACTACGTAACTGGGATTGATCTTTGTAATTTTTGATCATCAACGAGGAATTCCTTGTAAGCGCAAATCATTACT
TTGTGCTGAATCTGTCCCTGCCCTTTGTACACACCGCCCGTCGCTCCTACCGATCGAATGATACGGTAAAGCTAACAG
ATACGATTCGCAAGAATTGTAAAAGTTATTTAAATCTCATTGTTTAGAGGAAGGAGAAGTCGTAACAAGGTATCCGTA
GGTGAACCTGCAGAAGGATCAA

>AM168047.1 *Hagiwaraea lavandula* B15
AACCTGGTTGATCCTGCCAGTAGTCATATGCTTGTCTCAAAGATTAAGCCATGCATGTCTAAGTATAAATTTTTATATG
ATGAAACTGCAGAAGGCTCATTACAACAGTGATAAACTACTAGACTTTCGGGCTTCGGCCTTTTTGGATAACCGCAG
TAAATCGGGGCTAATACATACAATCGAGGGGTGACTGTTTACGGAATCTCCGCGATTATTAGCTTTCAGCCAACACTC
TTCGGAGTTTTGGGTGAACCCGGATAATATTGCAGATCGAAGCTTACGCTTTCGACAAGTCTAATGTGTCACTGCCCT
ATCAACTTTCGATGGTACGGTATTGGCCTACCATGGTTGTAACGGGTAACGGAGAATTAGGGTTCGATTCCGGAGAG
GGCGCCTGAGAAATGGCGACCACTTCTACGGAAGGCAGCAGGCGCGCAAATTACTCAATCCCAATACGGGGAAGTA
GTGACAAGAAATATTAATGCCTATCCCTTTCGGGAAGGTAATTAAAATGGATTTAAACTAAACCCATTTTTTAATACAA
TTGGAGGGCAAGTCTGGTGCCAGCAGCCGCGGTAATTCCAGCTCCAATAGCATATACTAAATTTGTTGCGGTTAAAA
AGCTCGTAGTTGAAGTTAAAATTATACTGGGTTCAAGGTTTTAGCCGACTTTGTCGGTTTAAAACTTCCAGTGTAGCT
TTTACAACTCGTCCAGGTGTCTTGGTACTTTGTATCAGGTCTTCTTTGGACATTTCACTGTGAGGAAATTGTGGTGCT
TAAAGCAGGCGTTTTCGCTTGATCTTTTGCAGCATGGTATGATAGAATATGACATTTTATGTAGTTGGCTCGCATAAAG
TGTAATGATTAATAGGGATGGATGGGGGTGTTCATATTGATGGGCGAGAGGTGAAATTCGTTGACCCTATCAAGATGA
ACTTCTGCGAAAGCATTCACCAAATACTTCTCCATTAATCAAGAACGAAAGTTTGGGGATCAAAGACGATCAGATAC
CGTCGTAGTCCAAACTATAAACTATGTCGACCAGGGATCAGCTAATATTTTTAAAAAATATAGTTGGCACCTTGTGAG
AAATCATGAGTGTTTAGATTCTGGGGGGAGTATGGCCGCAAGTCTGAAACTTAAAGGAATTGACGGAAGGGCACAC
AATGGAGTGGAGCCTGCGGCTTAATTTGACTCAACTCGGGAAAACTTACCAAGCTAAGATATAATAAGGATTGACAG
ACTAAAAGATCTTTCATGATTCTATAAGTGGTGGTGCATGGTCGTTCTTAGTTGGTGGAGCAATCTGTCTGGTCAATT
CCGATAACGGACGAGATCTCGACCTGCTAACTAGTAGTACATATTAGCTCAATATGCAAGAAAGGCTTGTTGGAGTA

GGTCATGGATCGCAAGGTTCATGGCTCTTCGCTGGTCGAGTATTGTGAGTTAATATGTGCGATAAAATAAAACTTCTT
AGAGGGACTACCTGTGGCAAACAGGGGGAAGTTCGAGGCAATAACAGGTCTGTGGTGCCCTTAGATACCTTGGGCC
GCACGCGCGCTACAATGTAGTACGCAAAAGGTATTTTGCCAAGCCCGGAAGGGTAAGGTAATCCTATAATTTACTAC
GTAACTGGGCTTGATCTTTGTAATTTTTGATCATCAACGAGGAATTCCTTGTAAGCGCAAATCATTACTTTGTGCTGA
ATCTGTCCCTGCCCTTTGTACACGCCGCCCGTCGCTCCTACCGATCGAATGATACGGTAAAGCTAACAGATATAACTC
GCAAGAGTTGTAAAAGTTATTTAAATCTCATTGTTTAGAGGAAGGAGAAGTCGTAACAAGGTATCCGTAGGTGAAC
CTGCAGAAGGATCAA

>HQ141494.1 *Hagiwaraea radiculata* ML5A
AACCTGGTTGATCCTGCCAGTAGTCATATGCTTGTCTCAAAGATTAAGCCATGCATGTCTAAGTATAAATTTTTATATG
ATGAAACTGCAGAAGGCTCATTACAACAGTGATAAACTACCAGACTTTCGGGCCTTTGGCCTTTTGGATAACCGCAG
TAAATCGGGGCTAATACATACAATCGAGGGGTGACTGTTTACGGAATCTCCGCGATTATTAGCTTTCAGCCAACACTC
TTCGAGTTTTGGGTGAACCCGGATAATATTGCAGATCGAAGCTTACGCTTTCGACAAGTCTGATGTGTCACTGCCCTA
TCAACTTTCGATGGTACGGTATTGGCCTACCATGGTTGTAACGGGTAACGGAGAATTAGGGTTCGATTCCGGAGAGG
GCGCCTGAGAAATGGCGACCACTTCTACGGAAGGCAGCAGGCGCGCAAATTACTCAATCCCAATACGGGGAAGTAG
TGACAAGAAATATTAATGCCTATCCCTCACGGGAAGGTAATTAAAATGGATTTAAACTAAACCCATTTTTTAATACAAT
GGAGGGCAAGTCTGGTGCCAGCAGCCGCGGTAATTCCAGCTCCAATAGCATATACTAAATTTGTTGCGGTTAAAAAG
CTCGTAGTTGAAATTAAAATTATACTGGGTTCAAGGTTTTAGTCGACTTCGGTCGGTTTAAAACTTCCAGTGTAAATT
TTAAAACTAATCCAGGTTGCCAGTTACTTTGTAATTGGTTTTCTTTGGATACTTCACTGTGAGGAAATTGTGGTGCTT
AAAGCAAGCGTTTTCGCTTGATCTTTTGCAGCATGGTATGATAGAATATGACATTTTATGTAGTTGGCTCGCATAAAGT
GTAATGATTAATAGGGATGGATGGGGGTGTTCATATTGATGGGCGAGAGGTGAAATTCGTTGACCCTATCAAGATGAA
CTTCTGCGAAAGCATTCACCAAATACTTCTCCATTAATCAAGAACGAAAGTTTGGGGATCAAAGACGATCAGATACC
GTCGTAGTCCAAACTATAAACTATGTCGACCAGGGATCAGCTAATATTTTTACAAAATATAGTTGGCACCTTGTGAGA
AATCATGAGTGTTTAGATTCTGGGGGGAGTATGGTCGCAAGTCTGAAACTTAAAGGAATTGACGGAAGGGCACACA
ATGGAGTGGAGCCTGCGGCTTAATTTGACTCAACTCGGGAAAACTTACCAAGCTAAGATATAATAAGGATTGACAGA
CTAAAAGATCTTTCATGATTCTATAAGTGGTGGTGCATGGTCGTTCTTAGTTGGTGGAGCAATCTGTCTGGTCAATTC
CGATAACGGACGAGATCTCGACCTGCTAACTAGTAGTACATATTAGCTCGATATGCAAGAAAGGCTTGTTGGAGTAA
GTTAGAGATCGCAAGGTTTCTAGCCCTTCGCTGGTCGAGTATTGTGGGTTAATATAGTGCGATAAATTTAAACTTCTTA
GAGGGACTACCTGTGGCAAACAGGGGGAAGTTCGAGGCAATAACAGGTCTGTGATGCCCTTAGATACCTTGGGCCG
CACGCGCGCTACAATGTAGTACGCAAAAGGTACTTTTCCAAACCCGGAAGGGTGAGGTAATCCTATAATTTACTACG
TAACTGGGCTTGATCTTTGTAATTTTTGATCATCAACGAGGAATTCCTTGTAAGCGCAAATCATTACTTTGTGCTGAAT
CTGTCCCTGCCCTTTGTACACACCGCCCGTCGCTCCTACCGATCGAATGATACGGTAAAGCTAACAGATATGTCCGCA
AGGACTGTAAAAGTTATTTAAATCTCATGTTTTAGAGGAAGGAGAAGTCGTAACAAGGTATCCGTAGGTGAACCTGC
AGAAGGATCA

>AM168063.1 *Hagiwaraea rhizopodium* AusKY-4
AACCTGGTTGATCCTGCCAGTAGTCATATGCTTGTCTCAAAGATTAAGCCATGCATGTCTAAGTATAAATTTTTATATG
ATGAAACTGCAGAAGGCTCATTACAACAGTGATAAACTACCAGACTTTCGGGCCTTTGGCCTTTTGGATAACCGCGG
TAAATCGGGGCTAATACATACAATCGAGGGGTGACTGTTTACGGAATCTCCGCGATTATTAGCTTTCAGCCAACACTC
TTCGGAGTTTTGGGTGAACCCGGATAATATTGCAGATCGAAGCTTACGCTTTCGACAAGTCTGATGTGTCACTGCCC
TATCAACTTTCGATGGTACGGTATTGGCCTACCATGGTTGTAACGGGTAACGGAGAATTAGGGTTCGATTCCGGAGA
GGGCGCCTGAGAAATGGCGACCACTTCTACGGAAGGCAGCAGGCGCGCAAATTACTCAATCCCAATACGGGGAAGT
AGTGACAAGAAATATTAATGCCTATCCCTCACGGGAAGGTAATTAAAATGGATTTAAACTAAACCCATTTTTTAATAC
AATTGGAGGGCAAGTCTGGTGCCAGCAGCCGCGGTAATTCCAGCTCCAATAGCATATACTAAATTTGTTGCGGTTAA
AAAGCTCGTAGTTGAAATTAAAATTATACTGGGTTCAAGGTTTTAGTCGACTTCGGTCGGTTTAAAACTTCCAGTGTA
AATTTTAAAACTAATCCAGGTTGCCAGTTACTTTGTAATTGGTTTTCTTTGGATACTTCACTGTGAGGAAATTGTGGT
GCTTAAAGCAAGCGTTTTCGCTTGATCTTTTGCAGCATGGTATGATAGAATATGACATTTTATGTAGTTGGCTCGCATA
AAGTGTAATGATTAATAGGGATGGATGGGGGTGTTCATATTGATGGGCGAGAGGTGAAATTCGTTGACCCTATCAAG
ATGAACTTCTGCGAAAGCATTCACCAAATACTTCTCCATTAATCAAGAACGAAAGTTTGGGGATCAAAGACGATCAG
ATACCGTCGTAGTCCAAACTATAAACTATGTCGACCAGGGATCAGCTAATATTTTTACAAAATATAGTTGGCACCTTGT
GAGAAATCATGAGTGTTTAGATTCTGGGGGGAGTATGGTCGCAAGTCTGAAACTTAAAGGAATTGACGGAAGGGCA
CACAATGGAGTGGAGCCTGCGGCTTAATTTGACTCAACTCGGGAAAACTTACCAAGCTAAGATATAATAAGGATTGA
CAGACTAAAAGATCTTTCATGATTCTATAAGTGGTGGTGCATGGTCGTTCTTAGTTGGTGGAGCAATCTGTCTGGTCA
ATTCCGATAACGGACGAGATCTCGACCTGCTAACTAGTAGTACATATTAGCTCGATATGCAAGAAAGGCTTGTTGGAG
TAAGTTAGAGATCGCAAGGTTTCTAGCCCTTCGCTGGTCGAGTATTGTGGGTTAATATAGTGCGATAAATTTAAACTT
CTTAGAGGGACTACCTGTGGCAAACAGGGGGAAGTTCGAGGCAATAACAGGTCTGTGATGCCCTTAGATACCTTGG
GCCGCACGCGCGCTACAATGTAGTACGCAAAAGGTACTTTTCCAAACCCGGAAGGGTGAGGTAATCCTATAATTTAC
TACGTAACTGGGCTTGATCTTTGTAATTTTTGATCATCAACGAGGAATTCCTTGTAAGCGCAAATCATTACTTTGTGCT
GAATCTGTCCCTGCCCTTTGTACACACCGCCCGTCGCTCCTACCGATCGAATGATACGGTAAAGCTAACAGATATGTC
CGCAAGGATGTAAAAGTTATTTAAATCTCATTGTTTAGAGGAAGGAGAAGTCGTAACAAGGTATCCGTAGGTGAACC
TGCAGAAGGATCAA

>MH762956.1 *Hagiwaraea tenebrica* Ong2
AAATTTTTATATGANTGAAACTGCAGAAGGCATCANNTACAACAGTGATAAACTACCAGACTTTCGGGCCTTGGCCT
TATGGATAACCGCAGTAAATCGGGGCTAATACATACAATCGAGGGGTGACTGTTTACGGAATCTCCGCGATTATTAGC
TTTCAGCCAACACTCTTCGGAGTCTTGGGTGAACCCGGATAATATTGCAGATCGAAGCTTACGCTTTCGACAAGTCT
GATGTGTCACTGCCCTATCAACTTTCGATGGTACGGTATTGGCCTACCATGGTTGTAACGGGTAACGGAGAATTAGGG
TTCGATTCCGGAGAGGGCGCCTGAGAAATGGCGACCACTTCTACGGAAGGCAGCAGGCGCGCAAATTACTCAATCC
CAATACGGGGAAGTAGTGACAAGAAATATTAATGCCTATCCCTCACGGGAAGGTAATTAAAATGGATTTAAACTAAA
CCCATTTTTTAATACAATTGGAGGGCAAGTCTGGTGCCAGCAGCCGCGGTAATTCCAGCTCCAATAGCATATACTAAA
TTTGTTGCGGTTAAAAAGCTCGTAGTTGAAACTAAAATTATACTGGGTTAAAGGTTTTAGCCGACTTCGGTCGGTTTA
AAACTTCCAGTGTAAATTTTAAAACTAGTCCAGGTTGCTAGTTACTTTGTAATTAGTTTTCTTTGGACATTTCACTGTG
AGGAAATTGTGGTGCTTAAAGCAGGCGTTTTCGCTTGATCTTTTGCAGCATGGTATGATAGAATATGACATTTTATGTA
GTTGGCTCGCATAAAGTGTAATGATTAATAGGGATGGATGGGGGTGTTCATATTGATGGGCGAGAGGTGAAATTCGTT
GACCCTATCAAGATGAACTTCTGCGAAAGCATTCACCAAATACTTCTCCATTAATCAAGAACGAAAGTTTGGGGATC

AAAGACGATCAGATACCGTCGTAGTCCAAACTATAAACTATGTCGACCAGGGATCAGCTAATATTTTTACAAAATATA
GTTGGCACCTTGTGAGAAATCATGAGTGTTTAGATTCTGGGGGGAGTATGGTCGCAAGTCTGAAACTTAAAGGAATT
GACGGAAGGGCACACAATGGAGTGGAGCCTGCGGCTTAATTTGACTCAACTCGGGAAAACTTACCAAGCTAAGATA
TAATAAGGATTGACAGACTAAAAGATCTTTCATGATTCTATAAGTGGTGGTGCATGGTCGTTCTTAGTTGGTGGAGCA
ATCTGTCTGGTCAATTCCGATAACGGACGAGATCTCGACCTGCTAACTAGTAGTACATATTAGCTCGATATGCAAGAA
AGGCTTGTTGGAGTAAGTTAGAGATCGCAAGGTTTCTAGCCCTTCGCTGGTCGAGTATTGTGGGTTGATATAGTGCG
ATAAATTTAAACTTCTTAGAGGGACTACCTGTGGCAAGCAGGGGGAAGTTCGAGGCAATAACAGGTCTGTGATGCC
CTTAGATACCTTGGGCCGCACGCGCGCTACAATGTAGTACGCAAAAGGTACTTTTCCAAACCCGGAAGGGTGAGGT
AATCCTATAATTTACTACGTAACTGGGCTTGATCTTTGTAATTTTTGATCATCAACGAGGAATTCCTTGTAAGCGCAAA
TCATTACTTTGTGCTGAATCTGTCCCTGCCCTTTGTACACACCGCCCGTCGCTCCTACCGATCGAATGATACGGTAAA
GCTAAYYAGATATGCCCGCAAGGGYYGTAAAAGTTATTTAAAT

>AM168062.1 *Hagiwaraea vinaceofusca* CC4
AACCTGGTTGATCCTGCCAGTAGTCATATGCTTGTCTCAAAGATTAAGCCATGCATGTCTAAGTATAAATTTTTATATG
ATGAAACTGCAGAAGGCTCATTACAACAGTGATAAACTACTAGACTTTCGGGCCTTCGGCCTTTTGGATAACCGCAG
TAAATCGGGGCTAATACATACAATCGAGGGGTGACTGTTTACGGAATCTCCGCGATTATTAGCTTTCAGCCAACACTC
TTCGGAGTTTTGGGTGAACCCGGATAATATTGCAGATCGAAGCTTACGCTTTCGACAAGTCTAATGTGTCACTGCCCT
ATCAACTTTCGATGGTACGGTATTGGCCTACCATGGTTGTAACGGGTAACGGAGAATTAGGGTTCGATTCCGGAGAG
GGCGCCTGAGAAATGGCGACCACTTCTACGGAAGGCAGCAGGCGCGCAAATTACTCAATCCCAATACGGGGAAGTA
GTGACAAGAAATATTAATGCCTATCCCTTTCGGGAAGGTAATTAAAATGGATTTAAACTAAACCCATTTTTTAATACAA
TTGGAGGGCAAGTCTGGTGCCAGCAGCCGCGGTAATTCCAGCTCCAATAGCATATACTAAATTTGTTGCGGTTAAAA
AGCTCGTAGTTGAAGTTAAAATTACACTGGGTTCAAGGTTTTAATCGACTCTGTCGGTTTAAAACTTCCAGTGTAGCT
TTTACAACTCATCCAGGTTTCTTGGTACTTTGTATCAGGTTTTCTTTGGATATTTCACTGCGAGGAAATTGTGGTGCTT
AAAGCAGGCGTTTTCGCTTGATCTTTTGCAGCATGGTATGATAGAATATGACATTTTATGTAGTTGGCTCGCATAAAGT
GTAATGATTAATAGGGATGGATGGGGGTGTTCATATTGATGGGCGAGAGGTGAAATTCGTTGACCCTATCAAGATGAA
CTTCTGCGAAAGCATTCACCAAATACTTCTCCATTAATCAAGAACGAAAGTTTGGGGATCAAAGACGATCAGATACC
GTCGTAGTCCAAACTATAAACTATGTCGACCAGGGATCAGCTAATATTTTTAAAAAATATAGTTGGCACCTTGTGAGA
AATCATGAGTGTTTAGATTCTGGGGGGAGTATGGTCGCAAGTCTGAAACTTAAAGGAATTAACGGAAGGGCACACA
ATGGAGTGGAGCCTGCGGCTTAATTTGACTCAACTCGGGAAAACTTACCAAGCTAAGATATAATAAGGATTGACAGA
CTAAAAGATCTTTCATGATTCTATAAGTGGTGGTGCATGGTCGTTCTTAGTTGGTGGAGCAATCTGTCTGGTCAATTC
CGATAACGGACGAGATCTCGACCTGCTAACTAGTAGTACATATTAGCTCGATATGCAAGAAAGGCTTGTTGGAGTAA
GTTATGGATCGCAAGGTTCATAGCCCTTCGCTGGTCGAGTATTGTGAGTTAATATAGTGCGATACAATAAAACTTCTTA
GAGGGACTACCTGTGGCAAACAGGGGGAAGTTCGAGGCAATAACAGGTCTGTGATGCCCTTAGATACCTTGGGCCG
CACGCGCGCTACAATGTAGTACGCAAAAGGTTTTTTGCCAAGCCCGGAAGGGTGAGGTAATCCTATAATTTACTACG
TAACTGGGATTGATCTTTGTAATTTTTGATCATCAACGAGGAATTCCTTGTAAGCGCAAATCATTACTTTGTGCTGAAT
CTGTCCCTGCCCTTTGTACACACCGCCCGTCGCTCCTACCGATCGAATGATACGGTAAAGCTAACAGATACAATTCGC
AAGAGTTGTAAAAGTTATTTAAATCTCATTGTTTAGAGGAAGGAGAAGTCGTAACAAGGTATCCGTAGGTGAACCTG
CAGAAGGATCAA

>AM168029.1 *Raperostelium australe* NZ80B
AACCTGGTTGATCCTGCCAGTAGTCATATGCTTGTCTCAAAGATTAAGCCATGCATGTCTAAGTATAAATTTTTATATG
ATGAAACTGCAGACGGCTCATTACAACAGTAATAAACTAATAGACTTTCGGGTTTCATTACCTTTTGGATAACCGCAG
TAAATCGGGGCTAATACATACAATCGAGGGCTGACTGTTTACGGAATGTCCGCGATTATTAGCATTTAACCAATACCC
TTCGGGGTTTTGTGGTGAAACCGAATAATATTGCAGATCGAAGCTTCGGCTTCGACAAGTCTATTGTGTTACTGCCCT
ATCAACTTTCGATGGTACGGTATTGGCCTACCATGGTTGTAACGGGTAACGGGGAATTAGGGTTCGATTCCGGAGAG
GGCGCCTGAGAAATGGCGACCACTTCTACGGAAGGCAGCAGGCGCGCAAATTACTCAATCCCAATACGGGGAAGTA
GTGACAAGAAATATTAATGCCTATCCTTTTAGGAAGGTAATTAAAATGGGTCTAAACTAAATCCATTTTCTAATACAAT
TGGAGGGCAAGTCTGGTGCCAGCAGCCGCGGTAATTCCAGCTCCAATAGCATATACTAAATTTGTTGCGGTTAAAAG
CTCGTAGTTGAAGTAAAAACCTTATTGGGTTAATAGGTCTTAGTCGATTTTATCGGTTTAAGGCTTCCAATACGCGTTT
TTTAAACTAGCTCATGGATTAGGTTACTTTTGTAATTCTGGTTCTTTTGGGTGTTTCACTGTGAGAAAATTGTGGTGCT
TAAAGCGGGCGTTTTGCCTGATCTTTTGCAGCATGGTATGATAGAACATGACATTTTGCGCTATTGGTTGCGTTAAAG
TGTAATGATTAATAGGGATGGATGGGGGTGTTCATATTGGTGGGCGAGAGGTGAAATTCGTTGACCCTATCAAGATGA
CTTCTGCGAAAGCATTCACCAAATACTTCCCCATTAATCAAGAACGAAAGTTTGGGGATCAAAGACGATCAGATACC
GTCGTAGTCCAAACTATAAACTATGTCGACCAAGGATCAGCTAAAATTTTACAAAAATTTAGTTGGCACTTTGTGAG
AAATCATGAGTGTTTAGATTCTGGGGGGAGTATGGTCGCAAGTCTGAAACTTAAAGGAATTGACGGAAGGGCACAC
AATGGAGTGGAGCCTGCGGCTTAATTTGACTCAACTCGGGAAAACTTACCAGGCTAAGATATAATAAGGATTGACAG
ACTAAAAGATCTTTCATGATTGTATAAGTGGTGGTGCATGGTCGTTCTTAGTTGGTGGAGCAATCTGTCTGGTCAATT
CCGATAACGGACGAGATCTCGACCTGCTAACTAGTAGTATATATCGATTCGATATGCATGAAAGCTTTGTTGGAGTAA
GTTGTAGGTTCGCAAGGGTCTATGACACTTCACTGGGTCGGTAATGTGGGTTGATAAATACGAAAAATTAAAAAAAC
TTCTTAGAGGGACTACCTGTGGCAAACAGGGGGAAGTTCGAGGCAATAACAGGTCTGTGATGCCCTTAGATACCTT
GGGCCGCACGCGCGCTACAATGTAGTACGCAAAAAGCAATCCTGGTCCGGGAGGACTGGGTAATCATAAATTTACTA
CGTAACTGGGATTGATCTTTGTAATTTTTGATCATTAACGAGGAATTCCTTGTAAGCGCAAATCATTACTTTGTGCTGA
ATATGTCCCTGCCCTTTGTACACACCGCCCGTCGCTCCTACCGATCGAATGATACGGTAAAGCCAACGGATGGAGTC
AGTAGCAATACATGACTCTAAAAGTTGTTTAAATCTCATTGTTTAGAGGAAGGAGAAGTCGTAACAAGGTATACCGT
AGGTGAACCTGCAGAAGGATCAA

>JF892721.1 *Raperostelium capillare* 37A
AACCTGGTTGATCCTGCCAGTAGTCATATGCTTGTCTCAAAGATTAAGCCATGCATGTCTAAGTATAAATTTTTATATG
ATGAAACTGCAGACGGCTCATTACAACAGTAATAAACTAATAGACTTTCGGGTTTAATTACCTTTTGGATAACCGCAG
TAAATCGGGGCTAATACATACAATCGAGGGGTGACTGTTTACGGAATCTCCGCGATTATTAGCATTCAACCAATACCT
TTCGAGGTTTTGTGGTGAAACCGAATAATATTGCAGATCAAAGCTTTCGGCTTTGACAAGTCTATTGTGTTACTGCCC
TATCAACTTTCGATGGTACGGTATTGGCCTACCATGGTTGTAACGGGTAACGGGGAATTAGGGTTCGATTCCGGAGA
GGGCGCCTGAGAAATGGCGACCACTTCTACGGAAGGCAGCAGGCGCGCAAATTACTCAATCCCAATACGGGGAAGT
AGTGACAAGAAATATTAATGCCTATCCTTTTAGGAAGGTAATTAAAATGGGTCTAAACTAAATCCATTTTCTAATACAA
TTGGAGGGCAAGTCTGGTGCCAGCAGCCGCGGTAATTCCAGCTCCAATAGCATATACTAAATTTGTTGCGGTTAAAA

AGCTCGTAGTTTAAACAAAAATTATATTGGGCTAATAGGTGTTAGCCGGATTTTTATCCCGGTTTAATACTTCCAATAT
ATATTTTTTAAATATTAGCTCTTGGATTTGGTTACTTTTGTAATTCAAGTTCGTTAGAGTCCTTCACTGTGAGAAAATT
GTGGTGCTTAAAGCAGGCGTTTCGCTTGATCTTTTGCAGCATGGTATGATAGAACATGACATTTTGTGCTATTGGTTG
CATTAAAGTGTAATGATTAATAGGGATGGATGGGGGTGTTCATATTGGTGGGCGAGAGGTGAAATTCGTTGACCCTAT
CAAGATGAACTTCTGCGAAAGCATTCACCAAATACTTCCCCATTAATCAAGAACGAAAGTTTGGGGATCAAAGACG
ATCAGATACCGTCGTAGTCCAAACTATAAACTATGTCGACCAGGGATCAGCTAAAATTTCTAAAAAATTTAGTTGGCA
CCTTGTGAGAAATCACGAGTGTTTAGATTCTGGGGGGAGTATGGTCGCAAGTCTGAAACTTAAAGGAATTGACGGA
AGGGCACACAATGGAGTGGAGCCTGCGGCTTAATTTGACTCAACTCGGGAAAACTTACCAAGCTAAGATATAATAA
GGATTGACAGACTAAAAGATCTTTCATGATTGTATAAGTGGTGGTGCATGGTCGTTCTTAGTTGGTGGAGCAATCTGT
CTGGTCAATTCCGATAACGGACGAGATCTCGACCTGCTAACTAGTAGTACATATTAGCTCAATATGCATGAAAGATTA
GTTGGAGTAAGTTGTAGGTCGAAAGGTTTACAGCACTTCACTGGTTCGGTAATGTGAATTAATATGGTACGATAAATA
AAACTTCTTAGAGGGACTACCTGTGGCAAACAGGGGGAAGTTCGAGGCAATAACAGGTCTGTGATGCCCTTAGATA
CCTTGGGCCGCACGCGCGCTACAATGTAGTACGCAAAAAGCTCTCCTGGTCCGGAAGGATTGGGTAATCAAAAAAT
TTACTACGTAACTGGGATTGATCTTTGTAATTATTGATCATCAACGAGGAATTCCTTGTAAGCGCAAATCATTACTTTG
TGCTGAATATGTCCCTGCCCTTTGTACACACCGCCCGTCGCTCCTACCGATCGAATGATACGGTAAAGCCAACAGATT
GGATCTGTAGCAATACAGAGCTGAAAAGTTGTTTAAATCTCATTGTTAGAGAAGGAGAAGTCGTAACAAGGTATCCG
TAGGTGAACCTGCAGAAGGATCA

>MH762957.1 *Raperostelium crispum* Eden2
TAAATTTTTATATGATGAAACTGCAGACGGCTCATTACAACAGTAATAAACTAATAGATTTTCGGGTTTTATTACCTTT
TGGATAACCGCAGTAAATCGGGGCTAATACATACAATCGAGGGGTGACTGTTTACGGAATCTCCGCGATTATTAGCTT
TCAACCAATACCCTTCGGGGTTTTGTGGCGAGACCGAATAATATTGCAGATCAAGGCTTCGGCTTTGACAAGTCTATT
GTGTTACTGCCCTATCAACTTTCGATGGTACGGTATTGGCCTACCATGGTTGTAACGGGTAACGGGGAATTAGGGTTC
GATTCCGGAGAGGGCGCCTGAGAAATGGCGACCACTTCTACGGAAGGCAGCAGGCGCGCAAATTACTCAATCCCAA
TACGGGGAAGTAGTGACAAGAAATATTAATGCCTATCCTTCGGGAAGGTAATTAAAATGGGTCTAAACTAAATCCATT
TTCTAATACAATTGGAGGGCAAGTCTGGTGCCAGCAGCCGCGGTAATTCCAGCTCCAATAGCATATACTAAATTTGTT
GCGGTTAAAAAGCTCGTAGTTGAAATTAAAATTACATTGGGTCAAGGGCTTTAGTCGATTTTTCGTCGGTTTAAAGCT
TCCAATGTATTTTTTTTAAAATTAACTCATAATCTTGGTTACTTTGTAATTCAGGGTTTTTTGGGTATTTCACTGTGAGA
AAATTGTGGTGCTTAAAGCGGGCGTTTTGCTTGATCTTTTGCAGCATGGTATGATAGAACATGACATTTTGTGCGAT
TGGTTGCATTAAAGTGTAATGATTAATAGGGATGGATGGGGGTGTTCATATTGGTGGGCGAGAGGTGAAATTCGTTG
ACCCTATCAAGATGAACTTCTGCGAAAGCATTCACCAAATACTTCCCCATTAATCAAGAACGAAAGTTTGGGGATCA
AAGACGATCAGATACCGTCGTAGTCCAAACTATAAACTATGTCGACCAGGGATCAGCTAAAATTTTACAAAAATTTA
GTTGGCACCTTGTGAGAAATCACGAGTGTTTAGATTCTGGGGGGAGTATGGTCGCAAGTCTGAAACTTAAAGGAAT
TGACGGAAGGGCACACAATGGAGTGGAGCCTGCGGCTTAATTTGACTCAACTCGGGAAAACTTACCAAGCTAAGAT
ATAATAAGGATTGACAGACTAAAAGATTTTTCATGATTGTATAAGTGGTGGTGCATGGTCGTTCTTAGTTGGTGGAGC
AATCTGTCTGGTCAATTCCGATAACGGACGAGATCTCGACCTGCTAACTAGTAGTACTTATCAGTTCGATATGCATGA
AAGGCTTGTTGGAGTAAGTTGTAGGTCGAAAGGTTTACAACACTTCACTGGTTCGGTAATGTGAGCAGATAAGTACG
AAAAATAACACTTCTTAGAGGGACTACCTGTGGCAAACAGGGGGAAGTTCGAGGCAATAACAGGTCTGTGATGCCC
TTAGATACCTTGGGCCGCACGCGCGCTACAATGTAGTACGCAAAAAGCTAACTGGTCTGGGAAGATTGGTTAATCAT
AAATTTACTACGTAACTGGGATTGATCTTTGTAATTATTGATCATCAACGAGGAATTCCTTGTAAGCGCAAATCATTAC
TTTGTGCTGAATATGTCCCTGCCCTTTGTACACACCGCCCGTCGCTCCTACCGATCGAATGATACGGTAAAGCCAACG
GATGAGATCATGTAGCAATACAATGGTCTTAAAAGTTGTTTAAAT

>MH762958.1 *Raperostelium cymosum* Krug15A
TAAATTTTTATATGATGAAACTGCAGACGGCTCATTACAACAGTAATAAACTAATAGACTTTCGGGTTTCATTACCTTT
TGGATAACCGCAGTAAATCGGGGCTAATACATACAATCGAGGGCTGACTGTTTACGGAATGTCCGCGATTATTAGCAT
TCAACCAATACCTCTCGGGGTTTTGTGGTGAAACCGAATAATATTGCAGATCGAAGCTTCGGCTTTGACAAGTCTATT
GTGTTACTGCCCTATCAACTTTCGATGGTACGGTATTGGCCTACCATGGTTGTAACGGGTAACGGGGAATTAGGGTTC
GATTCCGGAGAGGGCGCCTGAGAAATGGCGACCACTTCTACGGAAGGCAGCAGGCGCGCAAATTACTCAATCCCAA
TACGGGGAAGTAGTGACAAGAAATATTAATGCCTATCCTTTTAGGAAGGTAATTAAAATGGGTCTAAATTAAATCTAT
TTTCTAATACAATTGGAGGGCAAGTCTGGTGCCAGCAGCCGCGGTAATTCCAGCTCCAATAGCATATACTAAATTTGT
TGCGGTTAAAAAGCTCGTAGTTGAAATTAAAGCCTTATTGGGTTAATAGGTCTTAGTCGATTTCATCGGTTTAAGGCT
TCCAATATGTGTTTTTTAAATTAACTCATGGATTGGGTTACTTTTGTAATTCCAGTTCGTTTGGGTGCTTCACTGTGAG
AAAATTGTGGTGCTTAAAGCAGGCGTTTTGTCTGATCTTTTGCAGCATGGTATGATAGAACATGACATTTTGCGCTAT
TGGTTGCGTTAAAGTGTAATGATTAATAGGGATGGATGGGGGTGTTCATATTGGTGGGCGAGAGGTGAAATTCGTTG
ACCCTATCAAGATGAACTTCTGCGAAAGCATTCACCAAATACTTCCCCATTAATCAAGAACGAAAGTTTGGGGATCA
AAGACGATCAGATACCGTCGTAGTCCAAACTATAAACTATGTCGACCAAGGATCAGCTAAAATTTTACAAAAATTTA
GTTGGCACTTTGTGAGAAATCATGAGTGTTTAGATTCTGGGGGGAGTATGGTCGCAAGTCTGAAACTTAAAGGAATT
GACGGAAGGGCACACAATGGAGTGGAGCCTGCGGCTTAATTTGACTCAACTCGGGAAAACTTACCAAGCTAAGATA
TAATAAGGATTGACAGACTAAAAGATCTTTCATGATTGTATAAGTGGTGGTGCATGGTCGTTCTTAGTTGGTGGAGCA
ATCTGTCTGGTCAATTCCGATAACGGACGAGATCTCGACCTGCTAACTAGTAGTATATATCGATTCGATATGCATGAA
AGCCTTGTCGGAGTAAGTTGTAGGTTCGCAAGGGTCTATGGCACTTCGCTGGGTCGGTAATGTGAGTTGATAAATAC
GAAAAATTAAAAAAACTTCTTAGAGGGACTACCTGTGGCAAACAGGGGGAAGTTCGAGGCAATAACAGGTCTGTG
ATGCCCTTAGATACCTTGGGCCGCACGCGCGCTACAATGTAGTACGCAAAAAGCAATCCTGGTCCGGGAGGACTGG
GTAATCATAAATTTACTACGTAACTGGGATTGATCTTTGTAATTTTTGATCATCAACGAGGAATTCCTTGTAAGCGCAA
ATCATTACTTTGTGCTGAATATGTCCCTGCCCTTTGTACACACCGCCCGTCGCTCCTACCGATCGAATGATACGGTAAG
CCAACGGATGGAGTCAGTAGCAATACATGACTCTAAAAGTT

>JF892724.1 *Raperostelium filiforme* OH603
AACCTGGTTGATCCTGCCAGTAGTCATATGCTTGTCTCAAAGATTAAGCCATGCATGTCTAAGTATAAATTTTTATATG
ATGAAACTGCAGACGGCTCAATACAACAGTAATAAACTAATAGACTTTCGGGTTTAATTACCTTTTGGATAACCGCAG
TAAATCGGGGCTAATACATACAATCGAGGGCTGACTGTTTACGGAATGTCCGCGATTATTAGCATTCAACCAATACCT
TTCGAGGTTTTGTGGTGAAACCGAATAATATTGCAGATCAAAGCTTCGGCTTTGACAAGTCTATTGTGTTACTGCCCT
ATCAACTTTCGATGGTACGGTATTGGCCTACCATGGTTGTAACGGGTAACGGGGAATTAGGGTTCGATTCCGGAGAG
GGCGCCTGAGAAATGGCGACCACTTCTACGGAAGGCAGCAGGCGCGCAAATTACTCAATCCCAATACGGGGAAGTA

GTGACAAGAAATATTAATGCCTATCCTTTTAGGAAGGTAATTAAAATGGGTCTAAACTAAATCCATTTTCTAATACAAT
TGGAGGGCAAGTCTGGTGCCAGCAGCCGCGGTAATTCCAGCTCCAATAGCATATACTAAATTTGTTGCGGTTAAAAA
GCTCGTAGTTTAAACAAAAATTATATTGGGCTAATAGGTGTTAGCCGGATTTTTATCCCGGTTTAATACTTCCAATATAT
ATTTTTTAAATATTAGCTCTTGGATCTGGTTACTTTTGTAATTCAGGTTCGTTAGAGTCCTTCACTGTGAGAAAATTGT
GGTGCTTAAAGCAGGCGTTTCGCTTGATCTTTTGCAGCATGGTATGATAGAACATGACATTTTGTGCTATTGGTTGCA
TTAAAGTGTAATGATTAATAGGGATGGATGGGGGTGTTCATATTGGTGGGCGAGAGGTGAAATTCGTTGACCCTATCA
AGATGAACTTCTGCGAAAGCATTCACCAAATACTTCCCCATTAATCAAGAACGAAAGTTTGGGGATCAAAGACGATC
AGATACCGTCGTAGTCCAAACTATAAACTATGTCGACCAGGGATCAGCTAAAATTTCTAAAAAATTTAGTTGGCACCT
TGTGAGAAATCACGAGTGTTTAGATTCTGGGGGGAGTATGGTCGCAAGTCTGAAACTTAAAGGAATTGACGGAAGG
GCACACAATGGAGTGGAGCCTGCGGCTTAATTTGACTCAACTCGGGAAAACTTACCAAGCTAAGATATAATAAGGAT
TGACAGACTAAAAGATCTTTCATGATTGTATAAGTGGTGGTGCATGGTCGTTCTTAGTTGGTGGAGCAATCTGTCTGG
TCAATTCCGATAACGGACGAGATCTCGACCTGCTAACTAGTAGTACATATTAGCTCAATATGCATGAAAGATTAGTTG
GAGTAAGTTGTAGGTCGAAAGGTTTACAGCACTTCACTGGTTCGGTAATGTGAATTAATATGGTACGATAAATAAAAC
TTCTTAGAGGGACTACCTGTGGCAAACAGGGGGAAGTTCGAGGCAATAACAGGTCTGTGATGCCCTTAGATACCTT
GGGCCGCACGCGCGCTACAATGTAGTACGCAAAAAGCTCTCCTGGTCCGGAAGGATTGGGTAATCAAAAAATTTAC
TACGTAACTGGGATTGATCTTTGTAATTATTGATCATCAACGAGGAATTCCTTGTAAGCGCAAATCATTACTTTGTGCT
GAATATGTCCCTGCCCTTTGTACACACCGCCCGTCGCTCCTACCGATCGAATGATACGGTAAAGCCAACAGATTGGTT
TCGTAGGCAATTCAGAGCTGAAAAGATATTTAAATCTCATTGTTTAGAGGAAGGTGAACCTGCAGAAGGATCA

>AM168078.1 *Raperostelium gracile* TNS-C-183
AACCTGGTTGATCCTGCCAGTAGTCATATGCTTGTCTCAAAGATTAAGCCATGCATGTCTAAGTATAAATTTTTATATG
ATGAAACTGCAGACGGCTCATTACAACAGTAATAAACTAATAGACTTTCGGGTTTTATTACCTTTTGGATAACCGCAG
TAAATCGGGGCTAATACATACAATCGAGGGGTGACTGTTTACGGAATCCCCGCGATTATTAGCTTTTCAACCAATACT
CCTTTTGGAGTTTTGTGGTGAAACCGAATAATATTGCAGATCGAAGCTTAGGCATCGACAAGTCTACTGTGTTACTGC
CCTATCAACTTTCGATGGTACGGTATTGGCCTACCATGGTTGTAACGGGTAACGGGGAATTAGGGTTCGATTCCGGAG
AGGGCGCCTGAGAAATGGCGACCACTTCTACGGAAGGCAGCAGGCGCGCAAATTACTCAATCCCAATACGGGGAA
GTAGTGACAAGAAATATTAATGCCTATCCTTTTAGGAAGGTAATTAAAATGGGTCTAAACTAAATCCATTTTCTAATAC
AATTGGAGGGCAAGTCTGGTGCCAGCAGCCGCGGTAATTCCAGCTCCAATAGCATATACTAAATTTGTTGCGGTTAA
AAAGCTCGTAGTTGAAATTAAAATTATATTGGGCTAAATAGGTTTTAGCCGATTTTTCATCGGTTTAAAACTTCCAATA
TATATTTTTTAAATTTTGGCTCATGAATTTGGTTACTTTTGTAATTCAAGTTCTTTTTGGGTTCTTCACTGTGAGAAAAT
TGTGGTGCTTAAAGCAGGCGTTTTCGCTTGATCTTTTGCAGCATGGGATGATAGAACATGACATTTTGCGCTATTGGT
TGCGTTAAAGTGTAATGATTAATAGGGATGGATGGGGGTGTTCATATTGGTGGGCGAGAGGTGAAATTCGTTGACCC
TATCAAGATGAACTTCTGCGAAAGCATTCACCAAATACTTCCCCATTAATCAAGAACGAAAGTTTGGGGATCAAAGA
CGATCAGATACCGTCGTAGTCCAAACTATAAACTATGTCGACCAGGGATCAGCTAAAATTTCTAAAAAATTTAGTTGG
CACCTTGTGAGAAATCACGAGTGTTTAGATTCTGGGGGGAGTATGGTCGCAAGTCTGAAACTTAAAGGAATTGACG
GAAGGGCACACAATGGAGTGGAGCCTGCGGCTTAATTTGACTCAACTCGGGAAAACTTACCAAGCTAAGATATAAT
AAGGATTGACAGACTAAAAGATCTTTCATGATTGTATAAGTGGTGGTGCATGGTCGTTCTTAGTTGGTGGAGCAATCT
GTCTGGTCAATTCCGATAACGGACGAGATCTCGACCTGCTAACTAGTAGTACTTATCAGCTCAATATGCACGAAAGCT
TTGTTGGAGTAAGTTGTAGGTCGAAAGGTTTACAACACTTCGCTTGGTCGGTAGTGTGAATTGATAAGTACGCTTAA
ATAAAACTTCTTAGAGGGACTACCTGTGGCAAACAGGGGGAAGTTCGAGGCAATAACAGGTCTGTGATGCCCTTAA
TACCTTGGGCCGCACGCGCGCTACAATGTAGTACGCAAAAAGCAATCCTGGTCCGGAAGGACTGGGTAATCATAAAT
TTACTACGTAACTGGGATTGATCTTTGTAATTATTGATCATCAACGAGGAATTCCTTGTAAGCGCAAATCATTACTTTG
TGCTGAATATGTCCCTGCCCTTTGTACACACCGCCCGTCGCTCCTACCGATCGAATGATACGGTAAAGCCAACAGATT
AATGTATTATGGGCAACCATTTTACGTTGAAAAGTTGTTTAAATCTCATTGTTTAGAGGAAGGAGAAGTCGTAACAA
GGTATCCGTAGGTGAACCTGCAGAAGGATCAA

>HQ141495.1 *Raperostelium ibericum* 214rjb
AACCTGGTTGATCCTGCCAGTAGTCATATGCTTGTCTCAAAGATTAAGCCATGCATGTCTAGTATAAATTTTTATATGA
TGAAACTGCAGACGGCTCATTACAACAGTAATAAACTAATAGACTTTCAGGTTTTATTACCTTTTGGATAACCGCAGT
AAATCGGGGCTAATACATACAATTGAGGGCTGACTGTTTACGGAATGTCCGCGATTATTAGCATTCAACCAATATCCT
CCGGGATTTTGTGGTGAAACCGAATAATATTGCAGATCGGAGCCTTGGCTCCGACAAGTCTATTGTGTTACTGCCCTA
TCAACTTTCGATGGTACGGTATTGGCCTACCATGGTTGTAACGGGTAACGGGGAATTAGGGTTCGATTCCGGAGAGG
GCGCCTGAGAAATGGCGACCACTTCTACGGAAGGCAGCAGGCGCGCAAATTACTCAATCCCAATACGGGGAAGTAG
TGACAAGAAATATTAATGCCTATCCTTTTAGGAAGGTAATTAAAATGGGTCTAAACTAAATCCATTTTCTAATACAATT
GGAGGGCAAGTCTGGTGCCAGCAGCCGCGGTAATTCCAGCTCCAATAGCATATACTAAATTTGTTGCGGTTAAAAAG
CTCGTAGTTGAAATTAAAGCCATATTGGGTTAATGGTCTTAGCCGATTTTACGTCGGCCTAGGACTTCCAGTATGTGTT
TTTTAAATTTATCCTATAAGTTTGGTTACTTTTGTAATTCAGACGATCTAGGTGTTTCACTGTGAGAAAATTGTGGTGC
TTAAAGCGAGCGTTTTGCTTGATCTTTTGCAGCATGGTATGATAGAACATGACATTTTGCGCTATTGGTTGCGTTAAA
GTGTAATGATTAATAGGGATGGATGGGGGTGTTCATATTGGTGGGCGAGAGGTGAAATTCGTTGACCCTATCAAGATG
AACTTCTGCGAAAGCATTCACCAAATACTTCCCCATTAATCAAGAACGAAAGTTTGGGGATCAAAGACGATCAGATA
CCGTCGTAGTCCAAACTATAAACTATGTCGACCAAGGATCAGCTAAAATTTTACAAAAATTTAGTTGGCACTTTGTGA
GAAATCATGAGTGTTTAGATTCTGGGGGGAGTATGGTCGCAAGTCTGAAACTTAAAGGAATTGACGGAAGGGCACA
CAATGGAGTGGAGCCTGCGGCTTAATTTGACTCAACTCGGGAAAACTTACCAAGCTAAGATATAATAAGGATTGACA
GACTAAAAGATCTTTCATGATTGTATAAGTGGTGGTGCATGGTCGTTCTTAGTTGGTGGAGCAATCTGTCTGGTCA
ATTCCGATAACGGACGAGATCTCGACCTGCTAACTAGTAGTACATATTAGCTCAATATGCGTGAAAGCTTTGTTGGAG
TAAGTTGTAGGTCGAAAGGTTTATGACACTTCGCTGGGTCGGTAACGTGAATTAATATGTACGATAAAAACAAAACT
TCTTAGAGGGACTACCTGTGGCAAACAGGGGGAAGTTCGAGGCAATAACAGGTCTGTGATGCCCTTAGATACCTTG
GGCCGCACGCGCGCTACAATGTAGTACGCAAAAAGCTATCCTGGTCCGGGAGGACTGGGTAATCATTATAATTTACT
ACGTAACTGGGATTGATCTTTGTAATTATTGATCATCAACGAGGAATTCCTTGTAAGCGCAAATCATTACTTTGTGCTG
AATATGTCCCTGCCCTTTGTACACACCGCCCGTCGCTCCTACCGATCGAATGATACGGTAAAGCCAACAGATGAATTT
GGTAGCAATACTAAGTTTAAAAGTTGTTTAAATCTCATGTTAGAGGAAGAGAAGAAGTCGTAACAAGGTATCCGTAG
GTGAACCTGCAGAAGGATCA

>JF892719.1 *Raperostelium maeandriforme* OH604
AACCTGGTTGATCCTGCCAGTAGTCATATGCTTGTCTCAAAGATTAAGCCATGCATGTCTAAGTATAAATTTTTATATG

ATGAAACTGCAGACGGCTCATTACAACAGTAATAAACTAATAGACTTTCGGGTTTAATTACCTTTTGGATAACCGCAG
TAAATCGGGGCTAATACATACAATCGAGGGGTGACTGTTTACGGAATCTCCGCGATTATTAGCATTCAACCAATACCT
TTCGAGGTTTTGTGGTGAAACCGAATAATATTGCAGATCGAAGCTTCGGCTTTGACAAGTCTATTGTGTTACTGCCCT
ATCAACTTTCGATGGTACGGTATTGGCCTACCATGGTTGTAACGGGTAACGGGGAATTAGGGTTCGATTCCGGAGAG
GGCGCCTGAGAAATGGCGACCACTTCTACGGAAGGCAGCAGGCGCGCAAATTACTCAATCCCAATACGGGGAAGTA
GTGACAAGAAATATTAATGCCTATCCTTTTAGGAAGGTAATTAAAATGGGTCTAAACTAAATCCATTTTCTAATACAAT
TGGAGGGCAAGTCTGGTGCCAGCAGCCGCGGTAATTCCAGCTCCAATAGCATATACTAAATTTGTTGCGGTTAAAAA
GCTCGTAGTTTAAACAAAAATTATATTGGGCTAATAGGTGTTAGCCGGATTTTTATAAGCCGGTTTAATACTTCCAATA
TATATTTTTTAAATATTAGCTCTTGGATTTGGTTACTTTTGTAATTCAAGTTCGTTAGAGTCCTTCACTGTGAGAAAAT
TGTGGTGCTTAAAGCAGGCGTTTCGCTTGATCTTTTGCAGCATGGTATGATAGAACATGACATTTTGTGCTATTGGTT
GCATTAAAGTGTAATGATTAATAGGGATGGATGGGGGTGTTCATATTGGTGGGCGAGAGGTGAAATTCGTTGACCCTA
TCAAGATGAACTTCTGCGAAAGCATTCACCAAATACTTCCCCATTAATCAAGAACGAAAGTTTGGGGATCAAAGAC
GATCAGATACCGTCGTAGTCCAAACTATAAACTATGTCGACCAGGGATCAGCTAAAATTTCTAAAAAATTTAGTTGGC
ACCTTGTGAGAAATCACGAGTGTTTAGATTCTGGGGGGAGTATGGTCGCAAGTCTGAAACTTAAAGGAATTGACGG
AAGGGCACACAATGGAGTGGAGCCTGCGGCTTAATTTGACTCAACTCGGGAAAACTTACCAAGCTAAGATATAATA
AGGATTGACAGACTAAAAGATCTTTCATGATTGTATAAGTGGTGGTGCATGGTCGTTCTTAGTTGGTGGAGCAATCTG
TCTGGTCAATTCCGATAACGGACGAGATCTCGACCTGCTAACTAGTAGTACATATTAGCTCAATATGCATGAAAGATT
AGTTGGAGTAAGTTGTAGGTCGAAAGGTTTACAGCACTTCACTGGTTCGGTAATGTGAATTAATATGGTACGATAAAT
AAAACTTCTTAGAGGGACTACCTGTGGCAAACAGGGGGAAGTTCGAGGCAATAACAGGTCTGTGATGCCCTTAGAT
ACCTTGGGCCGCACGCGCGCTACAATGTAGTACGCAAAAAGCTCTCCTGGTCCGGAAGGATTGGGTAATCAAAAAA
TTTACTACGTAACTGGGATTGATCTTTGTAATTATTGATCATCAACGAGGAATTCCTTGTAAGCGCAAATCATTACTTT
GTGCTGAATATGTCCCTGCCCTTTGTACACACCGCCCGTCGCTCCTACCGATCGAATGATACGGTAAAGCCAACAGAT
TGGATCTGTAGCAATACAAGGTCTGAAAGTTGTTTAAATCTCATTGTTTAGAGGAAGGAGAAGTCGTAACAAGGTAT
CCGTAGGTGAACCTGCAGAAGGATCA

>AM168051.1 *Raperostelium minutum* 71-2
AACCTGGTTGATCCTGCCAGTAGTCATATGCTTGTCTCAAAGATTAAGCCATGCATGTCTAAGTATAAATTTTTATATG
ATGAAACTGCAGACGGCTCATTACAACAGTAATAAACTAATAGACTTTCGGGTTTTATTACCTTTTGGATAACCGCAG
TAAATCGGGGCTAATACATACAATCGAGGGGTGACTGTTTACGGAATCTCCGCGATTATTAGCTTTCAACCAATACCC
TTCGGGGTTTTGTGGCGAGACCGAATAATATTGCAGATCAAGGCTTCGGCTTTGACAAGTCTATTGTGTTACTGCCCT
ATCAACTTTCGATGGTACGGTATTGGCCTACCATGGTTGTAACGGGTAACGGGGAATTAGGGTTCGATTCCGGAGAG
GGCGCCTGAGAAATGGCGACCACTTCTACGGAAGGCAGCAGGCGCGCAAATTACTCAATCCCAATACGGGGAAGTA
GTGACAAGAAATATTAATGCCTATCCTTCGGGAAGGTAATTAAAATGGGTCTAAACTAAATCCATTTTCTAATACAATT
GGAGGGCAAGTCTGGTGCCAGCAGCCGCGGTAATTCCAGCTCCAATAGCATATACTAAATTTGTTGCGGTTAAAAAG
CTCGTAGTTGAAATTAAAATTACATTGGGTCAAGGGCTTTAGTCGATTTTTCGTCGGTTTAAAGCTTCCAATGTATTTT
TTTTAAAATTAACTCATAATCTTGGTTACTTTGTAATTCAGGGTTTTTTGGGTATTTCACTGTGAGAAAATTGTGGTGC
TTAAAGCGGGCGTTTTGCTTGATCTTTTGCAGCATGGTATGATAGAACATGACATTTTGTGCGATTGGTTGCATTAAA
GTGTAATGATTAATAGGGATGGATGGGGGTGTTCATATTGGTGGGCGAGAGGTGAAATTCGTTGACCCTATCAAGATG
AACTTCTGCGAAAGCATTCACCAAATACTTCCCCATTAATCAAGAACGAAAGTTTGGGGATCAAAGACGATCAGATA
CCGTCGTAGTCCAAACTATAAACTATGTCGACCAGGGATCAGCTAAAATTTTACAAAAATTTAGTTGGCACCTTGTGA
GAAATCACGAGTGTTTAGATTCTGGGGGGAGTATGGTCGCAAGTCTGAAACTTAAAGGAATTGACGGAAGGGCACA
CAATGGAGTGGAGCCTGCGGCTTAATTTGACTCAACTCGGGAAAACTTACCAAGCTAAGATATAATAAGGATTGACA
GACTAAAAGATTTTTCATGATTGTATAAGTGGTGGTGCATGGTCGTTCTTAGTTGGTGGAGCAATCTGTCTGGTCAAT
TCCGATAACGGACGAGATCTCGACCTGCTAACTAGTAGTACTTATCAGTTCGATATGCATGAAAGGCTTGTTGGAGTA
AGTTGTAGGTCGAAAGGTTTACAACACTTCACTGGTTCGGTAATGTGAGCAGATAAGTACGAAAAATAACACTTCTT
AGAGGGACTACCTGTGGCAAACAGGGGGAAGTTCGAGGCAATAACAGGTCTGTGATGCCCTTAGATACCTTGGGCC
GCACGCGCGCTACAATGTAGTACGCAAAAAGCTAACTGGTCTGGGAAGATTGGTTAATCATAAATTTACTACGTAAC
TGGGATTGATCTTTGTAATTATTGATCATCAACGAGGAATTCCTTGTAAGCGCAAATCATTACTTTGTGCTGAATATGT
CCCTGCCCTTTGTACACACCGCCCGTCGCTCCTACCGATCGAATGATACGGTAAAGCCAACGGATGAGATCATGTAG
CAATACAATGGTCTTAAAAGTTGTTTAAATCTCATTGTTTAGAGGAAGGAGAAGTCGTAACAAGGTATCCGTAGGTG
AACCTGCAGAAGGATCAA

>AM168052.1 *Raperostelium monochasioides* HAG653
AACCTGGTTGATCCTGCCAGTAGTCATATGCTTGTCTCAAAGATTAAGCCATGCATGTCTAAGTATAAATTTTTATATG
ATGAAACTGCAGACGGCTCATTACAACAGTAATAAACTAATAGACTTTCGGGTTTCATTACCTTTTGGATAACCGCAG
TAAATCGGGGCTAATACATACAATCGAGGGCTGACTGTTTACGGAATGTCCGCGATTATTAGCATTCAACCAATACCT
TTCGGGGTTTTGTGGTGAAACCGAATAATATTGCAGATCGAAGCTTCGGCTTCGACAAGTCTATTGTGTTACTGCCCT
ATCAACTTTCGATGGTACGGTATTGGCCTACCATGGTTGTAACGGGTAACGGGGAATTAGGGTTCGATTCCGGAGAG
GGCGCCTGAGAAATGGCGACCACTTCTACGGAAGGCAGCAGGCGCGCAAATTACTCAATCCCAATACGGGGAAGTA
GTGACAAGAAATATTAATGCCTATCCTTTTAGGAAGGTAATTAAAATGGGTCTAAACTAAATCCATTTTCTAATACAAT
TGGAGGGCAAGTCTGGTGCCAGCAGCCGCGGTAATTCCAGCTCCAATAGCATATACTAAATTTGTTGCGGTTAAAAA
GCTCGTAGTTGAAATAAAAGCCTTATTGGGTTAATAGGTCTTAGTCGATTTTTATCGGTTTAAGACTTCCAATATGTGT
TTTTTAAATTAACTCATGGATTAGGTTACTTTTGTAATTCTAGTTCGTTTGGGTGCTTCACTGTGAGAAAATTGTGGTG
CTTAAAGCAGGCGTTTTGCCTGATCTTTTGCAGCATGGTATGATAGAACATGACATTTTGCGCTATTGGTTGCGTTAA
AGTGTAATGATTAATAGGGATGGATGGGGGTGTTCATATTGGTGGGCGAGAGGTGAAATTCGTTGACCCTATCAAGAT
GAACTTCTGCGAAAGCATTCACCAAATACTTCCCCATTAATCAAGAACGAAAGTTTGGGGATCAAAGACGATCAGAT
ACCGTCGTAGTCCAAACTATAAACTATGTCGACCAAGGATCAGCTAAAATTTTACAAAAATTTAGTTGGCACTTTGTG
AGAAATCATGAGTGTTTAGATTCTGGGGGGAGTATGGTCGCAAGTCTGAAACTTAAAGGAATTGACGGAAGGGCAC
ACAATGGAGTGGAGCCTGCGGCTTAATTTGACTCAACTCGGGAAAACTTACCAAGCTAAGATATAATAAGGATTGAC
AGACTAAAAGATCTTTCATGATTGTATAAGTGGTGGTGCATGGTCGTTCTTAGTTGGTGGAGCAATCTGTCTGGTCAA
TTCCGATAACGGACGAGATCTCGACCTGCTAACTAGTAGTATATATCGATTCGATATGCATGAAAGCTTAGTCGGAGA
AGTTGTAGGTTCGCAAGGGTCTATGGCACTTCGCTGGGTCGGTAATGTGAGTTGATAAATACGAAAAATTAAAAAAA
CTTCTTAGAGGGACTACCTGTGGCAAACAGGGGGAAGTTCGAGGCAATAACAGGTCTGTGATGCCCTTAGATACCTT
GGGCCGCACGCGCGCTACAATGTAGTACGCAAAAAGCATTCCTGGTCCGGGAGGACTGGGTAATCATAAATTTACTA
CGTAACTGGGATTGATCTTTGTAATTTTTGATCATCAACGAGGAATTCCTTGTAAGCGCAAATCATTACTTTGTGCTGA

ATATGTCCCTGCCCTTTGTACACACCGCCCGTCGCTCCTACCGATCGAATGATACGGTAAAGCCAACGGACGGAGTC
AGTAGCAATACATGACTCTAAAAGTTGTTTAAATCTCGTTGTTTAGAGGAAGGAGAAGTCGTAACAAGGTATCCGTA
GGTGAACCTGCAGAAGGATCAA

>HQ141493.1 *Raperostelium ohioense* Okla4C
AACCTGGTTGATCCTGCCAGTAGTCATATGCTTGTCTCAAAGATTAAGCCATGCATGTCTAAGTATAAATTTTTATATG
ATGAAACTGCAGACGGCTCATTACAACAGTAATAAACTAATAGACTTTCGGGTTTTATTACCTTTGGATAACCGCAG
TAAATCGGGGCTAATACATACAATCGAGGGGTGACTGTTTACGGAATCTCCGCGATTATTAGCTTTCAACCAATACCC
TTCGGGTTTTGTGGCGAGACCGAATAATATTGCAGATCAAGGCTTCGGCTTTGACAAGTCTATTGTGTTACTGCCCTA
TCAACTTTCGATGGTACGGTATTGGCCTACCATGGTTGTAACGGGTAACGGGGAATTAGGGTTCGATTCCGGAGAGG
GCGCCTGAGAAATGGCGACCACTTCTACGGAAGGCAGCAGGCGCGCAAATTACTCAATCCCAATACGGGGAAGTAG
TGACAAGAAATATTAATGCCTATCCTTCGGGAAGGTAATTAAAATGGGTCTAAACTAAATCCATTTTCTAATACAATTG
GAGGGCAAGTCTGGTGCCAGCAGCCGCGGTAATTCCAGCTCCAATAGCATATACTAAATTTGTTGCGGTTAAAAAGC
TCGTAGTTGAAATTAAAATTACATTGGGTCAAGGGCTTTAGTCGATTTTTCGTCGGTTTAAAGCTTCCAATGTATTTTT
TTTAAAATTAACTCATAATCTTGGTTACTTTGTAATTCAGGGTTTTTTGGGTATTTCACTGTGAGAAAATTGTGGTGCT
TAAAGCGGGCGTTTTGCTTGATCTTTTGCAGCATGGTATGATAGAACATGACATTTTGTGCGATTGGTTGCATTAAAG
TGTAATGATTAATAGGGATGGATGGGGGTGTTCATATTGGTGGCGAGAGGTGAAATTCGTTGACCCTATCAAGATGA
ACTTCTGCGAAAGCATTCACCAAATACTTCCCCATTAATCAAGAACGAAAGTTTGGGGATCAAAGACGATCAGATAC
CGTCGTAGTCCAAACTATAAACTATGTCGACCAGGGATCAGCTAAAATTTTACAAAAATTTAGTTGGCACCTTGTGA
GAAATCACGAGTGTTTAGATTCTGGGGGGAGTATGGTCGCAAGTCTGAAACTTAAAGGAATTGACGGAAGGGCACA
CAATGGAGTGGAGCCTGCGGCTTAATTTGACTCAACTCGGGAAAACTTACCAAGCTAAGATATAATAAGGATTGACA
GACTAAAAGATTTTTCATGATTGTATAAGTGGTGGTGCATGGTCGTTCTAAGTTGGTGGAGCAATCTGTCTGGTCAAT
TCCGATAACGGACGAGATCTCGACCTGCTAACTAGTAGTACTTATCAGTTCGATATGCATGAAAGGCTTGTTGGAGTA
AGTTGTAGGTCGAAAGGTTTACAACACTTCACTGGTTCGGTAATGTGAGCAGATAAGTACGAAAAATAACACTTCTT
AGAGGGACTACCTGTGGCAAACAGGGGGAAGTTCGAGGCAATAACAGGTCTGTGATGCCCTTAGATACCTTGGGCC
GCACGCGCGCTACAATGTAGTACGCAAAAAGCTAACTGGTCTGGGAAGATTGGTTAATCATAAATTTACTACGTAAC
TGGGATTGATCTTTGTAATTATTGATCATCAACGAGGAATTCCTTGTAAGCGCAAATCATTACTTTGTGCTGAATATGC
CCTGCCCTTTGTACACACCGCCCGTCGCTCCTACCGATCGAATGATACGGTAAAGCCAACGGATGAGATCATGTAGC
AATACAATGGTCTTAAAAGTTGTTTAAATCTCATGTTTTAGAGGAAGGAGAAGTCGTAACAAGGTATCCGTAGGTGA
ACCTGCAGAAGGATCA

>AM168069.1 *Raperostelium potamoides* FP1A
AACCTGGTTGATCCTGCCAGTAGTCATATGCTTGTCTCAAAGATTAAGCCATGCATGTCTAAGTATAAATTTTTATATG
ATGAAACTGCAGACGGCTCATTACAACAGTAATAAACTAATAGACTTTCAGGTTTTATTACCTTTGGATAACCGCAG
TAAATCGGGGCTAATACATACAATTGAGGGCTGACTGTTTACGGGATGTCCGCGATTATTAGCATTCAACCAATACCT
CTCGGGGTTTTGTGGTGAAACCGAATAATATTGCAGATCGAGGCTTCGGCTTCGACAAGTCTATTGTGTTACTGCCCT
ATCAACTTTCGATGGTACGGTATTGGCCTACCATGGTTGTAACGGGTAACGGGGAATTAGGGTTCGATTCCGGAGAG
GGCGCCTGAGAAATGGCGACCACTTCTACGGAAGGCAGCAGGCGCGCAAATTACTCAATCCCAATACGGGGAAGTA
GTGACAAGAAATATTAATGCCTATCCTTTTAGGAAGGTAATTAAAATGGGTCTAAACTAAATCCATTTTCTAATACAAT
TGGAGGGCAAGTCTGGTGCCAGCAGCCGCGGTAATTCCAGCTCCAATAGCATATACTAAATTTGTTGCGGTTAAAAA
GCTCGTAGTTGAAATTAAAGCTATATTGGGTCAAGGGTTCTAGCCGGTTTTACACCGGTTTAGTAACTTCCAGTATAT
GTTTTTTAAATTTTTCCCATAAGTTTGGTTACTTTTGTAATTTCAAACGGTTCGGGTACTTCACTGTGAGAAAATTGTG
GTGCTTAAAGCGAGCGTTTTGCTTGATCTTTTGCAGCCTGGTATGATAGAACATGACATTTTACGCTATTGGTTGCGT
TAGAGTGTAATGATTAATAGGGATGGATGGGGGTGTTCATATTGGTGGGCGAGAGGTGAAATTCGTTGACCCTATCAA
GATGAACTTCTGCGAAAGCATTCACCAAATACTTCCCCATTAATCAAGAACGAAAGTTTGGGGATCAAAGACGATCA
GATACCGTCGTAGTCCAAACTATAAACTATGTCGACCAAGGATCAGCTAAAATTTTACAAAAATTTAGTTGGCACTTT
GTGAGAAATCATGAGTGTTTAGATTCTGGGGGGAGTATGGTCGCAAGTCTGAAACTTAAAGGAATTGACGGAAGGG
CACACAATGGAGTGGAGCCTGCGGCTTAATTTGACTCAACTCGGGAAAACTTACCAAGCTAAGATATAATAAGGATT
GACAGACTAAAAGATCTTTCATGATTGTATAAGTGGTGGTGCATGGTCGTTCTTAGTTGGTGGAGCAATCTGTCTGGT
CAATTCCGATAACGGACGAGATCTCGACCTGCTAACTAGTAGTACATATTAGCTCAATATGCGTGAAAGTTTCGGCGG
AGTAAGTTGTAGGTCGAAAGGTTTATGACACTTCACGGGATCGGTAACGTGAATTAATATTGTACGATAAAAATAAA
ACTTCTTAGAGGGACTACCTGTGGCAAACAGGGGGAAGTTCGAGGCAATAACAGGTCTGTGATGCCCTTAGATACC
TTGGGCCGCACGCGCGCTACAATGTAGTACGCAAAAAGCTTTCCTGGTCCGGGAGGACTGGGTAATCATTATAATTT
ACTACGTAACTGGGATTGATCTTTGTAATTATTGATCATCAACGAGGAATTCCTTGTAAGCGCAAATCATTACTTTGTG
CTGAATATGTCCCTGCCCTTTGTACACACCGCCCGTCGCTCCTACCGATCGAATGATACGGTAAAGCCAACAGATGA
ATTGGTAGTAATACTAAGTTTAAAAGTTGTTTAAATCTCATTGTTTAGAGGAAGGAGAAGTCGTAACAAGGTATCCG
TAGGTGAACCTGCAGAAGGATCAA

>JF892718.1 *Raperostelium reciprocatum* 38A
AACCTGGTTGATCCTGCCAGTAGTCATATGCTTGTCTCAAAGATTAAGCCATGCATGTCTAAGTATAAATTTTTATATG
ATGAAACTGCAGACGGCTCATTACAACAGTAATAAACTAATAGACTTTCGGGTTTCATTACCTTTGGATAACCGCAG
TAAATCGGGGCTAATACATACAATCGAGGGCTGACTGTTTACGGAATGTCCGCGATTATTAGCATTCAACCAATACCT
CTCGGGGTTTTGTGGTGAAACCGAATAATATTGCAGATCGAAGCTTCGGCTTCGACAAGTCTATTGTGTTACTGCCCT
ATCAACTTTCGATGGTACGGTATTGGCCTACCATGGTTGTAACGGGTAACGGGGAATTAGGGTTCGATTCCGGAGAG
GGCGCCTGAGAAATGGCGACCACTTCTACGGAAGGCAGCAGGCGCGCAAATTACTCAATCCCAATACGGGGAAGTA
GTGACAAGAAATATTAATGCCTATCCTCTTAGGAAGGTAATTAAAATGGGTCTAAACTAAATCCATTTTCTAATACAAT
TGGAGGGCAAGTCTGGTGCCAGCAGCCGCGGTAATTCCAGCTCCAATAGCATATACTAAATTTGTTGCGGTTAAAAA
GCTCGTAGTTGAAGTAAAAGCCTTATTGGGTTAATAGGTCTTAGCCGACTTCGTCGGTTTAAGGCTTCCAATATGTGT
TTTTTAAATTAATTCATGGATTAGGTTACTTTTGTAATTCTAGTTCTTTTGGGTGTTTCACTGTGAGAAAATTGTGGTG
CTTAAAGCGGGCGTTTAGCCTGATCTTTTGCAGCATGGTATGATAGAACATGACATTTGCGCTATTGGTTGCGTTAA
AGTGTAATGATTAATAGGGATGGATGGGGGTGTTCATATTGGTGGGCGAGAGGTGAAATTCGTTGACCCTATCAAGAT
GAACTTCTGCGAAAGCATTCACCAAATACTTCCCCATTAATCAAGAACGAAAGTTTGGGGATCAAAGACGATCAGAT
ACCGTCGTAGTCCAAACTATAAACTATGTCGACCAAGGATCAGCTAAAATTTTACAAAAATTTAGTTGGCACTTTGTG
AGAAATCATGAGTGTTTAGATTCTGGGGGGAGTATGGTCGCAAGTCTGAAACTTAAAGGAATTGACGGAAGGGCAC
ACAATGGAGTGGAGCCTGCGGCTTAATTTGACTCAACTCGGGAAAACTTACCAAGCTAAGATATAATAAGGATTGAC

AGACTAAAAGATCTTTCATGATTGTATAAGTGGTGGTGCATGGTCGTTCTTAGTTGGTGGAGCAATCTGTCTGGTCAA
TTCCGATAACGGACGAGATCTCGACCTGCTAACTAGTAGTATATATCGATTCAATATGCATGAAAGCCTTGTTGGAGA
AGTTGTAGGTCGCAAGGTCTATGACACTTCGCTGGGTCGGTAATGTGAGTTGATAAATACGAAAAATTAAAAAAACT
TCTTAGAGGGACTACCTGTGGCAAACAGGGGGAAGTTCGAGGCAATAACAGGTCTGTGATGCCCTTAGATACCTTG
GGCCGCACGCGCGCTACAATGTAGTACGCAAAAAGCAATCCTGGTCCGGGAGGACTGGGTAATCATAAATTTACTAC
GTAACTGGGATTGATCTTTGTAATTTTTGATCATCAACGAGGAATTCCTTGTAAGCGCAAATCATTACTTTGTGCTGA
ATATGTCCCTGCCCTTTGTACACACCGCCCGTCGCTCCTACCGATCGAATGATACGGTAAAGCCAACAGATGGAGTC
AGTAGCAATACATGACTTTAAAAGTTGTTTAAATCTCATTGTTAGAGGAAGGAGAAGTCGTAACAAGGTATCCGTAG
GTGAACCTGCAGAAGGATCA

>JF892723.1 *Raperostelium reciprocatum* var. *transitum* OH601
AACCTGGTTGATCCTGCCAGTAGTCATATGCTTGTCTCAAAGATTAAGCCATGCATGTCTAAGTATAAATTTTTATATG
ATGAAACTGCAGACGGCTCATTACAACAGTAATAAACTAATAGACTTTCGGGTTTCATTACCTTTTGGATAACCGCAG
TAAATCGGGGCTAATACATACAATCGAGGGCTGACTGTTTACGGAATGTCCGCGATTATTAGCATTCAACCAATACCT
CTCGGGGTTTTGTGGTGAAACCGAATAATATTGCAGATCGAAGCTTCGGCTTCGACAAGTCTATTGTGTTACTGCCCT
ATCAACTTTCGATGGTACGGTATTGGCCTACCATGGTTGTAACGGGTAACGGGGAATTAGGGTTCGATTCCGGAGAG
GGCGCCTGAGAAATGGCGACCACTTCTACGGAAGGCAGCAGGCGCGCAAATTACTCAATCCCAATACGGGGAAGTA
GTGACAAGAAATATTAATGCCTATCCTCTTAGGAAGGTAATTAAAATGGGTCTAAACTAAATCCATTTTCTAATACAAT
TGGAGGGCAAGTCTGGTGCCAGCAGCCGCGGTAATTCCAGCTCCAATAGCATATACTAAATTTGTTGCGGTTAAAAA
GCTCGTAGTTGAAGTAAAAGCCTTATTGGGTTAATAGGTCTTAGCCGACTTCGTCGGTTTAAGGCTTCCAATATGTGT
TTTTTAAATTAATTCATGGATTAGGTTACTTTTGTAATTCTAGTTCTTTTGGGTGTTTCACTGTGAGAAAATTGTGGTG
CTTAAAGCGGGCGTTTAGCCTGATCTTTTGCAGCATGGTATGATAGAACATGACATTTTGCGCTATTGGTTGCGTTAA
AGTGTAATGATTAATAGGGATGGATGGGGGTGTTCATATTGGTGGGCGAGAGGTGAAATTCGTTGACCCTATCAAGAT
GAATTCTGCGAAAGCATTCACCAAATACTTCCCCATTAATCAAGAACGAAAGTTTGGGGATCAAAGACGATCAGATA
CCGTCGTAGTCCAAACTATAAACTATGTCGACCAAGGATCAGCTAAAATTTTACAAAAATTTAGTTGGCACTTTGTGA
GAAATCATGAGTGTTTAGATTCTGGGGGGAGTATGGTCGCAAGTCTGAAACTTAAAGGAATTGACGGAAGGGCACA
CAATGGAGTGGAGCCTGCGGCTTAATTTGACTCAACTCGGGAAAACTTACCAAGCTAAGATATAATAAGGATTGACA
GACTAAAAGATCTTTCATGATTGTATAAGTGGTGGTGCATGGTCGTTCTTAGTTGGTGGAGCAATCTGTCTGGTCAAT
TCCGATAACGGACGAGATCTCGACCTGCTAACTAGTAGTATATATCGATTCAATATGCATGAAAGCCTTGTTGGAGTA
AGTTGTAGGTCGCAAGGTCTATGACACTTCGCTGGGTCGGTAATGTGAGTTGATAAATACGAAAAATTAAAAAAACT
TCTTAGAGGGACTACCTGTGGCAAACAGGGGGAAGTTCGAGGCAATAACAGGTCTGTGATGCCCTTAGATACCTTG
GGCCGCACGCGCGCTACAATGTAGTACGCAAAAAGCAATCCTGGTCCGGGAGGACTGGGTAATCATAAATTTACTAC
GTAACTGGGATTGATCTTTGTAATTTTTGATCATCAACGAGGAATTCCTTGTAAGCGCAAATCATTACTTTGTGCTGA
ATATGTCCCTGCCCTTTGTACACACCGCCCGTCGCTCCTACCGATCGAATGATACGGTAAAGCCAACAGATGGAGTC
AGTAGCAATACATGACTTTAAAAGTTGTTTAAATCTCATTGTTAGAGGAAGGAGAAGTCGTAACAAGGTATCCGTAG
GTGAACCTGCAGAAGGATCA

>MN338957.1 *Raperostelium stabile* M12A
AACCTGGTTGATCCTGCCAGTAGTCATATGCTTGTCTCAAAGATTAAGCCATGCATGTCTAAGTATAAATTTTTATATG
ATGAAACTGCAGACGGCTCATTACAACAGTAATAAACTAATAGACTTTCGGGTTTCATTACCTTTTGGATAACCGCAG
TAAATCGGGGCTAATACATACAATCGAGGGCTGACTGTTTACGGAATGTCCGCGATTATTAGTCTTAACCAGTACCCT
TCGGGGTTTTGATGGTGAAACCGAATAATATTGCAGATCGAAGCTTACGCTTTGACAAGTCTATTGTGTTACTGCCCT
ATCAACTTTCGATGGTACGGTATTGGCCTACCATGGTTGTAACGGGTAACGGGGAATTAGGGTTCGATTCCGGAGAG
GGCGCCTGAGAAATGGCGACCACTTCTACGGAAGGCAGCAGGCGCGCAAATTACTCAATCCCAATACGGGGAAGTA
GTGACAAGAAATATTAATGCCTATCCTTTTAGGAAGGTAATTAAAATGGGTCTAAACTAAATCCATTTTCTAATACAAT
TGGAGGGCAAGTCTGGTGCCAGCAGCCGCGGTAATTCCAGCTCCAATAGCATATACTAAATTTGTTGCGGTTAAAAA
GCTCGTAGTTGAAGTTAAAGCCTTATTGGGTTAATAGGTTTTAGCCGATTTATTCGGTTTAAAGCTTCCAATATGTGTT
TTTTAAATTAACTCATGGATTAGGTTACTTTTGTAATTCTAGTTCTTTTGGGTGTTTCACTGTGAGAAAATTGTGGTGC
TTAAAGCGGGCGTTTTGCCTGATCTTTTGCAGCATGGTATGATAGAACATGACATTTTGCGCTATTGGTTGCGTTAAG
TGTAATGATTAATAGGGATGGATGGGGGTGTTCATATTGGTGGGCGAGAGGTGAAATTCGTTGACCCTATCAAGATGA
ACTTCTGCGAAAGCATTCACCAAATACTTCCCCATTAATCAAGAACGAAAGTTTGGGGATCAAAGACGATCAGATAC
CGTCGTAGTCCAAACTATAAACTATGTCGACCAAGGATCAGCTAAAATTTTACAAAAATTTAGTTGGCACTTTGTGAG
AAATCATGAGTGTTTAGATTCTGGGGGGAGTATGGTCGCAAGTCTGAAACTTAAAGGAATTGACGGAAGGGCACAC
AATGGAGTGGAGCCTGCGGCTTAATTTGACTCAACTCGGGAAAACTTACCAAGCTAAGATATAATAAGGATTGACAG
ACTAAAAGATTTTTCATGATTGTATAAGTGGTGGTGCATGGTCGTTCTTAGTTGGAGGAGCAATCTGTCTGGTCAATT
CCGATAACGGACGAGATCTCGACCTGCTAACTAGTAGTATATATCGATTCGATATGCATGAAAGCCTTGTCGGAGTAA
GTTGTAGGTTCGCAAGGGTCTATGGCACTTCGCTGGGTCGGTAATGTGAGTTGATAGGTACGAAAAATTAAAAAAAC
TTCTTAGAGGGACTACCTGTGGCAAACAGGGGGAAGTTCGAGGCAATAACAGGTCTGTGATGCCCTTAGATACCTT
GGGCCGCACGCGCGCTACAATGTAGTACGCAAAAAGCAATCCTGGTCCGGGAGGACTGGGTAATCATAAATTTACTA
CGTAACTGGGATTGATCTTTGTAATTTTTGATCATCAACGAGGAATTCCTTGTAAGCGCAAATCATTACTTTGTGCTGA
ATATGTCCCTGCCCTTTGTACACACCGCCCGTCGCTCCTACCGATCGAATGATACGGTAAAGCCAACCGATAGGCCA
GTAGCAATACATGGCTTTAAAAGTTGTTTAAATCTCATTGTTTAGAGGAAGGAGAAGTCGTAACAAGGTATCCGTAG
GTGAACCTGCAGAAGGATCA

>AM168076.1 *Raperostelium tenue* Pan52
AACCTGGTTGATCCTGCCAGTAGTCATATGCTTGTCTCAAAGATTAAGCCATGCATGTCTAAGTATAAATTTTTATATG
ATGAAACTGCAGACGGCTCATTACAACAGTAATAAACTAATAGACTTTCGGGTTTCATTACCTTTTGGATAACCGCAG
TAAATCGGGGCTAATACATACAATCGAGGGCTGACTGTTTACGGAATGTCCGCGATTATTAGCATTCAACCAATACCC
TCGGGGTTTTGTGGTGAAACCGAATAATATTGCAGATCGAAGCTTCGGCTTCGACAAGTCTATTGTGTTACTGCCCTA
TCAACTTTCGATGGTACGGTATTGGCCTACCATGGTTGTAACGGGTAACGGGGAATTAGGGTTCGATTCCGGAGAGG
GCGCCTGAGAAATGGCGACCACTTCTACGGAAGGCAGCAGGCGCGCAAATTACTCAATCCCAATACGGGGAAGTAG
TGACAAGAAATATTAATGCCTATCCTCTTAGGAAGGTAATTAAAATGGGTCTAAACTAAATCCATTTTCTAATACAATT
GGAGGGCAAGTCTGGTGCCAGCAGCCGCGGTAATTCCAGCTCCAATAGCATATACTAAATTTGTTGCGGTTAAAAAG
CTCGTAGTTGAAGTAAAAGCCTTATTGGGTTAATAGGTCTTAGCCGACTTCGTCGGTTTAAGGCTTCCAATATGTGTT
TTTTAAATTAATTCATGGATTAGGTTACTTTTGTAATTCTAGTTCTTTTGGGTGTTTCACTGTGAGAAAATTGTGGTGC

TTAAAGCGGGCGTTTAGCCTGATCTTTTGCAGCATGGTATGATAGAACATGACATTTTGCGCTATTGGTTGCGTTAAA
GTGTAATGATTAATAGGGATGGATGGGGGTGTTCATATTGGTGGGCGAGAGGTGAAATTCGTTGACCCTATCAAGATG
AACTTCTGCGAAAGCATTCACCAAATACTTCCCCATTAATCAAGAACGAAAGTTTGGGGATCAAAGACGATCAGATA
CCGTCGTAGTCCAAACTATAAACTATGTCGACCAAGGATCAGCTAAAATTTTACAAAAATTTAGTTGGCACTTTGTGA
GAAATCATGAGTGTTTAGATTCTGGGGGGAGTATGGTCGCAAGTCTGAAACTTAAAGGAATTGACGGAAGGGCACA
CAATGGAGTGGAGCCTGCGGCTTAATTTGACTCAACTCGGGAAAACTTACCAAGCTAAGATATAATAAGGATTGACA
GACTAAAAGATCTTTCATGATTGTATAAGTGGTGGTGCATGGTCGTTCTTAGTTGGTGGAGCAATCTGTCTGGTCAAT
TCCGATAACGGACGAGATCTCGACCTGCTAACTAGTAGTATATATCGATTCAATATGCATGAAAGCCTTGTTGGAGTA
AGTTGTAGGTCGCAAGGTCTATGACACTTCGCTGGGTCGGTAATGTGAGTTGATAAATACGAAAAATTAAAAAAACT
TCTTAGAGGGACTACCTGTGGCAAACAGGGGGAAGTTCGAGGCAATAACAGGTCTGTGATGCCCTTAGATACCTTG
GGCCGCACGCGCGCTACAATGTAGTACGCAAAAAGCAATCCTGGTCCGGGAGGACTGGGTAATCATAAATTTACTAC
GTAACTGGGATTGATCTTTGTAATTTTTGATCATCAACGAGGAATTCCTTGTAAGCGCAAATCATTACTTTGTGCTGA
ATATGTCCCTGCCCTTTGTACACACCGCCCGTCGCTCCTACCGATCGAATGATACGGTAAAGCCAACAGATGGAGTC
AGTAGCAATACATGACTTTAAAAGTTGTTTAAATCTCATTGTTTAGAGGAAGGAGAAGTCGTAACAAGGTATCCGTAT
GGTGAACCTGCAGAAGGATCAA

>AM168094.1 *Raperostelium tenue* PJ6
AACCTGGTTGATCCTGCCAGTAGTCATATGCTTGTCTCAAAGATTAAGCCATGCATGTCTAAGTATAAATTTTTATATG
ATGAAACTGCAGACGGCTCATTACAACAGTAATAAACTAATAGACTTTCGGGTTTTATTACCTTTTGGATAACCGCAG
TAAATCGGGGCTAATACATACAATCGAGGGGTGACTGTTTACGGAATCTCCGCGATTATTAGCTTTCTCAACCAATAC
CCTTCGGGGTTTTGATGGTGAAACCGAATAATATTGCAGATCGAAGCTTCGGCATCGACAAGTCTATTGTGTTACTGC
CCTATCAACTTTCGATGGTACGGTATTGGCCTACCATGGTTGTAACGGGTAACGGGGAATTAGGGTTCGATTCCGGAG
AGGGCGCCTGAGAAATGGCGACCACTTCTACGGAAGGCAGCAGGCGCGCAAATTACTCAATCCCAATACGGGGAA
GTAGTGACAAGAAATATTAATGCCTATCCTCATAGGAAGGTAATTAAAATGGGTCTAAACTAAATCCATTTTCTAATAC
AATTGGAGGGCAAGTCTGGTGCCAGCAGCCGCGGTAATTCCAGCTCCAATAGCATATACTAAATTTGTTGCGGTTAA
AAAGCTCGTAGTTGAAATTAAAGTCATATTGGGTTAATAGGTGTTAGCCGATTTTAATGTCGGTTTAATGCTTCCAGTA
TGTATTTTTTAAATTGACTCTTGGGTTCGGTTACTTTGTAGTCGGGCTCGTTAGGGTTCTTCACTGTGAGAAAATTGT
GGTGCTTAAAGCAGGCGTTTCGCTTGATCTTTTGCAGCATGGTATGATAGAACATGACATTTTGCGCTATTGGTTGCG
TTAAAGTGTAATGATTAATAGGGATGGATGGGGGTGTTCATATCGGTGGGCGAGAGGTGAAATTCGTTGACCCTATCA
AGATGAACTTCTGCGAAAGCATTCACCAAATACTTCCCCATTAATCAAGAACGAAAGTTTGGGGATCAAAGACGATC
AGATACCGTCGTAGTCCAAACTATAAACTATGTCGACCAGGGATCAGCTAAAATTTCTAAAAAATTTAGTTGGCACCT
TGTGAGAAATCATGAGTGTTTAGATTCTGGGGGGAGTATGGTCGCAAGTCTGAAACTTAAAGGAATTGACGGAAGG
GCACACAATGGAGTGGAGCCTGCGGCTTAATTTGACTCAACTCGGGAAAACTTACCAAGCTAAGATATAATAAGGAT
TGACAGACTAAAAGATCTTTTATGATTGTATAAGTGGTGGTGCATGGTCGTTCTTAGTTGGTGGAGCAATCTGTCTGG
TCAATTCCGATAACGGACGAGATCTCGACCTGCTAACTAGTAGTACCTATTAGCTCAATATGCATGAAAGCTTTGTTG
GAGTAAGTTGTAGGTCGAAAGGTTTACAACACTTCGCTGGGTCGGTAATGTGAATTAATAGGTACGATAAACAAAAC
TTCTTAGAGGGACTACCTGTGGCAAACAGGGGGAAGTTCGAGGCAATAACAGGTCTGTGATGCCCTTAGATACCTT
GGGCCGCACGCGCGCTACAATGTAGTACGCAAAAAGCTATCCTGGTCCGGAAGGATTGGGTAATCATAAATTTACTA
CGTAACTGGGATTGATCTTTGTAATTATTGATCATCAACGAGGAATTCCTTGTAAGCGCAAATCATTACTTTGTGCTGA
ATATGTCCCTGCCCTTTGTACACACCGCCCGTCGCTCCTACCGATCGAATGATACGGTAAAGCCAACAGATTGGGTCT
GTAGCAATACTTGATCCGAAAAGTTGTTTAAATCTCATTGTTTAGAGGAAGGAGAAGTCGTAACAAGGTATCCGTAG
GTGAACCTGCAGAAGGATCAA

>AM168075.1 *Raperostelium tenue* PR4
AACCTGGTTGATCCTGCCAGTAGTCATATGCTTGTCTCAAAGATTAAGCCATGCATGTCTAAGTATAAATTTTTATATG
ATGAAACTGCAGACGGCTCATTACAACAGTAATAAACTAATAGACTTTCGGGTTTTATTACCTTTTGGATAACCGCAG
TAAATCGGGGCTAATACATACAATCGAGGGGTGACTGTTTACGGAATCTCCGCGATTATTAGCTTTCTCAACCAATAC
CCTTCGGGGTTTTGATGGTGAAACCGAATAATATTGCAGATCGAAGCTTCGGCATCGACAAGTCTATTGTGTTACTGC
CCTATCAACTTTCGATGGTACGGTATTGGCCTACCATGGTTGTAACGGGTAACGGGGAATTAGGGTTCGATTCCGGAG
AGGGCGCCTGAGAAATGGCGACCACTTCTACGGAAGGCAGCAGGCGCGCAAATTACTCAATCCCAATACGGGGAGT
AGTGACAAGAAATATTAATGCCTATCCTCATAGGAAGGTAATTAAAATGGGTCTAAACTAAATCCATTTTCTAATACA
ATTGGAGGGCAAGTCTGGTGCCAGCAGCCGCGGTAATTCCAGCTCCAATAGCATATACTAAATTTGTTGCGGTTAAA
AAGCTCGTAGTTGAAATTAAAGTCATATTGGGTTAATAGGTGTTAGCCGATTTTAATGTCGGTTTAATGCTTCCAGTAT
GTATTTTTTAAATTGACTCTTGGGTTCGGTTACTTTGTAGTCGGGCTCGTTAGGGTTCTTCACTGTGAGAAAAATTGT
GGTGCTTAAGCAGGCGTTTCGCTTGATCTTTTGCAGCATGGTATGATAGAACATGACATTTTGCGCTATTGGTTGCGT
TAAAGTGTAATGATTAATAGGGATGGATGGGGGTGTTCATATCGGTGGGCGAGAGGTGAAATTCGTTGACCCTATCA
AGATGAACTTCTGCGAAAGCATTCACCAAATACTTCCCCATTAATCAAGAACGAAAGTTTGGGGATCAAAGACGATC
AGATACCGTCGTAGTCCAAACTATAAACTATGTCGACCAGGGATCAGCTAAAATTTCTAAAAAATTTAGTTGGCACCT
TGTGAGAAATCATGAGTGTTTAGATTCTGGGGGGAGTATGGTCGCAAGTCTGAAACTTAAAGGAATTGACGGAAGG
GCACACAATGGAGTGGAGCCTGCGGCTTAATTTGACTCAACTCGGGAAAACTTACCAAGCTAAGATATAATAAGGAT
TGACAGACTAAAAGATCTTTCATGATTGTATAAGTGGTGGTGCATGGTCGTTCTTAGTTGGTGGAGCCATCTGTCTGG
TCAATTCCGATAACGGACGAGATCTCGACCTGCTAACTAGTAGTACCTATTAGCTCAATATGCATGAAAGCTTTGTTG
GAGTAAGTTGTAGGTCGAAAGGTTTACAACACTTCGCTGGGTCGGTAATGTGAATTAATAGGTACGATAAACAAAAC
TTCTTAGAGGGACTACCTGTGGCAAACAGGGGGAAGTTCGAGGCAATAACAGGTCTGTGATGCCCTTAGATACCTT
GGGCCGCACGCGCGCTACAATGTAGTACGCAAAAAGCTATCCTGGTCCGGAAGGATTGGGTAATCATAAATTTACTA
CGTAACTGGGATTGATCTTTGTAATTATTGATCATCAACGAGGAATTCCTTGTAAGCGCAAATCATTACTTTGTGCTGA
ATATGTCCCTGCCCTTTGTACACACCGCCCGTCGCTCCTACCGATCGAATGATACGGTAAAGCCAACAGATTGGGTCT
GTAGCAATACTTGATCCGAAAAGTTGTTTAAATCTCATTGTTTAGAGGAAGGAGAAGTCGTAACAAGGTATCCGTAG
GTGAACCTGCAGAAGGATCAA

>AM168077.1 *Speleostelium caveatum* WS695
AACCTGGTTGATCCTGCCAGTAGTCATATGCTTGTCTCAAGGATTAAGCCATGCATGTCTAAGTATAAATCTTTATACG
GTGAAACTGCAGACGGCTCATTACAATAGTGATAAACTAATAGATTTTCGGGTTTACCTTTTGGATAACCGCAGTAAA
TCGGGGCTAATACATAGAAGCGAGGGGTGACTGGCAACGGAAGCTCCGCGATTATTAGTTTATTACCAATCCCGCAA
GGGCTAGTGGTGAAACCGAATAATATTGCTGATCGACATGTATTTGTCGACAAATCTACTGTGTCACTACCCTATCAA

CTTTCGATGGTACGGTATTGGCCTACCATGGTTGTAACGGGTAACGGGGAATCAGGATTCGATTCCGGAGAGGGAGC
CTGAGAAATGGCTACCACTTCTACGGAAGGCAGCAGGCGCGCAAATTACTCAATCCCAATACGGGGAAGTAGCGAC
AATAAATATTAATGCCCATCTATTTTATAGAGGGCAATTAAAATGGACAAAATATAAACTACATAGTTAATACAATTGG
AGGGCAAGTCTGGTGCCAGCAGCCGCGGTAATTCCAGCTCCAATAGCATATACTAAAGTTGTTGCAGTTAAAAAGCT
CGTAGTTGAAATTTGGGCTATTAAGGGCTCAAACCAACATCCACTTTATAGTGTTTGTATGGTTCCTTATAGACTCAAC
TTGACTCAGTATAGTCTACTTTTGTAGTTCTGTATCTGAGTACTTCACTGTGAGAAAATTGTGGTGTTTAAAGCAAGC
GTATCGCTTGATCTTTTGCAGCATGGTATGATAAAACATGATCTGCGTCAACCATTGGTAGTTGACTGTATGTAATGAT
TAATAGGGGAGGATGGGGATGTTCATATTGATGGGCGAGAGGTGAAATTCGTTGACCCTATCAAGATGAACTTCTGC
GAAAGCATTCATCAAATACTCCCCCATTAATCAAGAACGAAAGTTTGGGGATCAAAGACGTTCAGATACCGTCGTAG
TCCAAACTATAAACGATGTCGACCAGGGATCAGCCGATATTTATAAAAATGCGGTTGGCACCTTGTGGGAAACCATG
AGTTTTTAGACTCTGGGGGAAGTATGGTCGCAAGTCTGAAACTTAAAGGAATTGACGGAAGGGCACACAATGGAGT
GGAGCCTGCGGCTTAATTTGACTCAACTCGGGAAAACTTACCAAGCTAAGATATAGTTAGGATTGACAGACTAAAAG
ATCTTTCATGATTCTATAAGTGGTGGTGCATGGTCGTTCTTAGTTGGTGGAGCAATCTGTCTGGTCAATTCCGATAACG
GACGAGATCTCGACCTGCTAACTAGTATCACTTATCAACCAATATAGTCGGTACCTTTTCCTATGCTGACTTTGTTTAC
TCAGGGTGACGGGTGGGTTTAGAATATTGGCTGGTTAGATAAGTGAGATAAAATTAAAACTTCTTAGAGGGACTACC
TTGCGGTAAGCAGGTGGAAGTTCGAGGCAATAACAGGTCTGTGATGCCCTTAGATA

>HQ141478.1 *Dictyostelium ammophilum* KBK4A
AACCTGGTTGATCCTGCCAGTAGTCATATGCTTGTCTCAAAGATTAAGCCATGCATGTCTAAGTATAAATTCTTGTACG
ATGAAACTGCAGACGGCTCATTACAACAGTGATAAACTAATAGACTTTCGGGTTTTTAACCTTTTGGATAACCGCAGT
AAATCGGGGCTAATACATAGAAGCGATGGGTGACTGGCAACGGAAGCTCAGCGATTATTAGCATTACTACCAATACC
TTCGGGTCTTGTGGTGAAACCGAATAATATTGCAGATCGAGGATTTATCTTCGACAAGTCTACTGTGTCACTGCCCTA
TCAACTTTCGATGGTACGGTATTGGCCTACCATGGTTGTAACGGGTAACGGGGAATTAGGGTTCGATTCCGGAGAGG
GAGCCTGAGAAATGGCTACCACTTCTACGGAAGGCAGCAGGCGCGCAAATTACTCAATCCCAATACGGGGAAGTAG
TGACAATAAATATCAATACCTATCCTTAACGGAGGGCAATTGAAATGAACACAAATTAAAACTCTTAATTAACACAAT
TGGAGGGCAAGTCTGGTGCCAGCAGCCGCGGTAATTCCAGCTCCAATAGCATATACTAAAGTTGTTGCAGTTAAAAA
GCTCGTAGTTGAAGTTAAAGGTTTACTGGGCTAAAGTTATTTGCCGCTCTGGTGGTTAAATATACTCCAGTATCTCTTT
TTTTAATAGTTCAGCTTCTATTATCTTTGATAGTAGTTGTTTGGACATTTCACTGTGAGAAAATTGTGGTGTTTAAAGC
AGGCGTTTCGCCTGATCTTTTGCAGCATGGTATGATGGAACATGACATTTTACGCTATTGGTTTGCGTCTAAAGTGTA
ATGATTAATAGGGATGGATGGGGGTGTTCATATTGGTGGGCGAGAGGTGAAATTCGTTGACCCTATCAAGATGAACTT
CTGCGAAAGCATTCACCAAATACTTCCCCATTAATCAAGAACGAAAGTTTGGGGATCGAAGACGATCAGATACCGTC
GTAGTCCAAACTATAAACTATGTCGACCAGGGATCGGTTAAAATTTTTTTAAAATTTAATCGGCACCTTGTGAGAAAT
CACGAGTGTTTAGATTCCGGGGGGAGTATGGTCGCAAGTCTGAAACTTAAAGGAATTGACGGAAGGGCACACAATG
GAGTGGAGCCTGCGGCTTAATTTGACTCAACTCGGGAAAACTTACCAAGCTAAGATATAGTAAGGATTGACAGACTA
AAAGATCTTTCATGATTCTATAAGTGGTGGTGCATGGTCGTTCTTAGTTGGTGGAGCGATTTGTCTGGTCAATTCCGA
TAACGGACGAGACCTCGACCTGCTAACTAGTAGTATTTATTAGTCAATATGGGCGATAGCTTTTCTGGGGTTTGGAAT
GATTTCGGTCATCTCCTGCTTCAAGGAGTGTGTAGTCTGACTTGATAGGTACGAATTAAAAAACTTCTTAGAGGGAC
TACCTGCCTCAAGCAGGCGGAAGTCCGAGGCAATAACAGGTCTGTGATGCCCTTAGATACCTTGGGCCGCACGCGC
GCTACAATGTAGAAAACAAAAAGGTTCCTGGTCCGGAAGGATTGGGTAATCAATTGAATTTTCTACGTAACTGGGAT
TGATCTTTGTAATTATTGATCATCAACGAGGAATTCCTTGTAAGCGTAGGTCATTACCCTATGCTGAATATGTCCCTGC
CCTTTGTACACACCGCCCGTCGCTCCTACCGATCGAATGATACGGTAAAGTTAACGGATCGTTTTATCTGTGGCAACA
CTGATATAAACTAAAAGTTATTTAAATCTCATGTTAGAGGAAGGAGAAGTCGTAACAAGGTATCCGTAGGTGAACCT
GCAGAAGGATCA

>AM167876.1 *Dictyostelium aureocephalum* TNS-C-180
AACCTGGTTGATCCTGCCAGTAGTCATATGCTTGTCTCAAAGATTAAGCCATGCATGTCTAAGTATAAATTCTTGTACG
ATGAAACTGCAGACGGCTCATTACAACAGTGATAAACTGCTAGACTTTCGGGTTTTTAACCTTTTGGATAACCGCAG
TAAATCGGGGCTAATACATAGAAGCGATGGGCGACTGGTAACGGAAGCTCAGCGATTATTAGCATAACTACCAATAC
CTTCGGGTCTTGTGGTGAAACCGAATAATATTGCAGATCGAAGATTTATCTTCGACAAGTCTAATGTGTCACTGCCCT
ATCAACTTTCGATGGTACGGTATTGGCCTACCATGGTTGTAACGGGTAACGGGGAATTAGGGTTCGATTCCGGAGAG
GGAGCCTGAGAAATGGCTACCACTTCTACGGAAGGCAGCAGGCGCGCAAATTACTCAATCCCAATACGGGGAAGTA
GTGACAATAAATATCAATACCTATCCTTAATGGAGGGCAATTGAAATGAACACAAATTAAAACTCTTAATTAACACAA
TTGGAGGGCAAGTCTGGTGCCAGCAGCCGCGGTAATTCCAGCTCCAATAGCATATACTAAAGTTGTTGCAGTTAAAA
AGCTCGTAGTTGAAGTTAAAGGTTTATTGGGTCAAAGCTATTTGCCGCTCTGGTGGTTAAATACGCTCCAGTATTTCT
TTTTTTTGAAAAATTGTTCAGCTTGTGTCAACCTAGTTGGCGCTCGTTTGGACATTTCACTGTGAGAAAATTGTGGTG
TTTAAAGCAGGCGTCTCGCCTGATCTTTTGCAGCATGGTATGATGAAACATGACATTTTGCGCTATTGGTTTGCGTCT
AAAGTGTAATGATTAATAGGGATGGATGGGGGTGTTCATATTGGTGGGCGAGAGGTGAAATTCGTTGACCCTATCAA
GATGAACTTCTGCGAAAGCATTCACCAAATACTTCTCCATTAATCAAGAACGAAAGTTTGGGGATCGAAGACGATCA
GATACCGTCGTAGTCCAAACTATAAACTATGTCGACCAGGGATCGGTTAAAATTTTTTTAAAATTTAATCGGCACCTT
GTGAGAAATCACGAGTGTTTAGATTCCGGGGGGAGTATGGTCGCAAGTCTGAAACTTAAAGGAATTGACGGAAGGG
CACACAATGGAGTGGAGCCTGCGGCTTAATTTGACTCAACTCGGGAAAACTTACCAAGCTAAGATATAGTTAGGATT
GACAGACTAAAAGATCTTTCATGATTCTATAAGTGGTGGTGCATGGTCGTTCTTAGTTGGTGGAGCGATTTGTCTGGT
CAATTCCGATAACGGACGAGACCTCGACCTGCTAACTAGTAGTATTTATTAGTCAATATGGGCGATAGCTTTTCTGGG
ATGGAGAGCCCTTTCGGGGGTTTCTTATTTCAAGGAGTGTGTAGTCTGACTTGATAGGTACGAATTAAAAAAACTTC
TTAGAGGGACTACCTGCCTCAAGCAGGCGGAAGTCCGAGGCAATAACAGGTCTGTGATGCCCTTAGATACCTTGGG
CCGCACGCGCGCTACAATGTATAAAACAAAAAGGTTCCTGGTCCGGAAGGATTGGGTAATCAATTGAATTTTCTACG
TAACTGGGATTGATCTTTGTAATTATTGATCATAAACGAGGAATTCCTTGTAAGCGTAGGTCATTACCCTATGCTGAAT
ATGTCCCTGCCCTTTGTACACACCGCCCGTCGCTCCTACCGATCGAATGATACGGTAAAGTTAACGGATAGTTTTATC
TTTGGCAACATTGATATAAATTAAAAGTTATTTAAATCTCATTGTTTAGAGGAAGGAGAAGTCGTAACAAGGTATCCG
TAGGTGAACCTGCAGAAGGATCAA

>AM168028.1 *Dictyostelium aureum* SL1
AACCTGGTTGATCCTGCCAGTAGTCATATGCTTGTCTCAAAGATTAAGCCATGCATGTCTAAGTATAAATTCTTGTACG
ATGAAACTGCAGACGGCTCATTACAACAGTGATAAACTGCTAGACTTTCGGGTTTTTAACCTTTTGGATAACCGCAG
TAAATCGGGGCTAATACATAGAAGCGATGGGCGACTGGTAACGGAAGCTCAGCGATTATTAGCATAACTACCAATAC

CTTCGGGTCTTGTGGTGAAACCGAATAATATTGCAGATCGAAGATTTATCTTCGACAAGTCTAATGTGTCACTGCCCT
ATCAACTTTCGATGGTACGGTATTGGCCTACCATGGTTGTAACGGGTAACGGGGAATTAGGGTTCGATTCCGGAGAG
GGAGCCTGAGAAATGGCTACCACTTCTATGGAAGGCAGCAGGCGCGCAAATTACTCAATCCCAATACGGGGAAGTA
GTGACAATAAATATCAATACCTATCCTTAATGGAGGGCAATTGAAATGAACACAAATTAAAACTCTTAATTAACACAA
TTGGAGGGCAAGTCTGGTGCCAGCAGCCGCGGTAATTCCAGCTCCAATAGCATATACTAAAGTTGTTGCAGTTAAAA
AGCTCGTAGTTGAAGTTAAAGGTTTATTGGGTCAAAGCCATTTGCCGCTCTGGTGGTTAAATGTGCTCCAGTATTTCT
TTTTTTTGAAAAATTGTTCAGCTTGTATCAACCTAGTTGGTACTCGTTTGGACATTTCACTGTGAGAAAATTGTGGTG
TTTAAAGCAGGCGTCTCGCCTGATCTTTTGCAGCATGGTATGATGAAACATGACATTTTGCGCTATTGGTTTGCGTCT
AAAGTGTAATGATTAATAGGGATGGATGGGGGTGTTCATATTGGTGGGCGAGAGGTGAAATTCGTTGACCCTATCAA
GATGAACTTCTGCGAAAGCATTCATCAAATACTTCCCCATTAATCAAGAACGAAAGTTTGGGGATCGAAGACGATCA
GATACCGTCGTAGTCCAAACTATAAACTATGTCGACCAGGGATCGGTTAAAATTTTTTTAAAATTTAATCGGCACCTT
GTGAGAAATCACGAGTGTTTAGATTCCGGGGGGAGTATGGTCGCAAGTCTGAAACTTAAAGGAATTGACGGAAGGG
CACACAATGGAGTGGAGCCTGCGGCTTAATTTGACTCAACTCGGGAAAACTTACCAAGCTAAGATATAGTTAGGATT
GACAGACTAAAAGATCTTTCATGATTCTATAAGTGGTGGTGCATGGTCGTTCTTAGTTGGTGGAGCGATTTGTCTGGT
CAATTCCGATAACGGACGAGACCTCGACCTGCTAACTAGTAGTATTTATTAGTCAACATGGGCGATAGCTTTTCTGGG
ATTTAGAACGATTTCGGTCGTTTCTGGTTTCAAGGAGTGTGTAGTCTGACTTGATAGGTACGAATTAAAAAAACTTCT
TAGAGGGACTACCTGCCTCAAGCAGGCGGAAGTCCGAGGCAATAACAGGTCTGTGATGCCCTTAGATACCTTGGGC
CGCACGCGCGCTACAATGTATAAAACAAAAAGGTTCCTGGTCCGGAAGGATTGGGTAATCAATTGAATTTTCTACGT
AACTGGGATTGATCTTTGTAATTATTGATCATAAACGAGGAATTCCTTGTAAGCGTAGGTCATTACCCTATGCTGAATA
TGTCCCTGCCCTTTGTACACACCGCCCGTCGCTCCTACCGATCGAATGATACGGTAAAGTTAACGGATAGTTTTATCT
TTGGCAACATTGATATAAATTAAAAGTTATTTAAATCTCATTGTTTAGAGGAAGGAGAAGTCGTAACAAGGTATCCGT
AGGTGAACCTGCAGAAGGATCAA

>GQ496158.1 *Dictyostelium austroandinum*
GTCTCAAGATTAGCCATGCATGTCTAAGTATAAATTCTTGTACGATGAAACTGCAGACGGCTCATTACAACAGTGATA
AACTGCTAGACTTTCGGGTTTTTAACCTTTTGGATAACCGCAGTAAATCGGGGCTAATACATAGAAGCGATGGGCGA
CTGGTAACGGAAGCTCAGCGATTATTAGCATAACTACCAATACCTTCGGGTCTTGTGGTGAAACCGAATAATATTGCA
GATCGAAGATTTATCTTCGACAAGTCTAATGTGTCACTGCCCTATCAACTTTCGATGGTACGGTATTGGCCTACCATGG
TTGTAACGGGTAACGGGGAATTAGGGTTCGATTCCGGAGAGGGAGCCTGAGAAATGGCTACCACTTCTACGGAAGG
CAGCAGGCGCGCAAATTACTCAATCCCAATACGGGGAAGTAGTGACAATAAATATCAATACCTATCCTTAATGGAGG
GCAATTGAAATGAACACAAATTAAAACTCTTAATTAACACAATTGGAGGGCAAGTCTGGTGCCAGCAGCCGCGGTA
ATTCCAGCTCCAATAGCATATACTAAAGTTGTTGCAGTTAAAAAGCTCGTAGTTGAAGTTAAAGGTTTATTGGGTCAA
AGCTATTTGCCGCTCTGGTGGTTAAATATGCTCCAGTATTTCTTTTTTTTGAAAAATTGTTCAGCTTGTATCAACTTTG
TTGGTACTCGTTTGGACATTTCACTGTGAGAAAATTGTGGTGTTTAAAGCAGGCGTCTCGCCTGATCTTTTGCAGCAT
GGTATGATGAAACATGACATTTTGCGCTATTGGTTTGCGTCTAAAGTGTAATGATTAATAGGGATGGATGGGGGTGTT
CATATTGGTGGGCGAGAGGTGAAATTCGTTGACCCTATCAAGATGAACTTCTGCGAAAGCATTCACCAAATACTTCT
CCATTAATCAAGAACGAAAGTTTGGGGATCGAAGACGATCAGATACCGTCGTAGTCCAAACTATAAACTATGTCGAC
CAGGGATCGGTTAAAATTTTTTTAAAATTTAATCGGCACCTTGTGAGAAATCACGAGTGTTTAGATTCCGGGGGGAG
TATGGTCGCAAGTCTGAAACTTAAAGGAATTGACGGAAGGGCACACAATGGAGTGGAGCCTGCGGCTTAATTTGAC
TCAACTCGGGAAAACTTACCAAGCTAAGATATAGTTAGGATTGACAGACTAAAAGATCTTTCATGATTCTATAAGTGG
TGGTGCATGGTCGTTCTTAGTTGGTGGTGCGATTTGTCTGGTCAATTCCGATAACGGACGAGACCTCGACCTGCTAA
CTAGTAGTATTTATTAGTCAATATGGGCGATAGCTTTTCTGGGATTTAGAGCGATTTCGGTCGTTTCTGGTTTCAAGGA
GTGTGTAGTCTGACTTGATAGGTACGAATTAAAAAAACTTCTTAGAGGGACTACCTGCCTCAAGCAGGCGGAAGTC
CGAGGCAATAACAGGTCTGTGATGCCCTTAGATACCTTGGGCCGCACGCGCGCTACAATGTATAAAACAAAAAGGTT
CCTGGTCCGGAAGGATTGGGTAATCAATTGAATTTTCTACGTAACTGGGATTGATCTTTGTAATTATTGATCATAAACG
AGGAATTCCTTGTAAGCGTAGGTCATTACCCTATGCTGAATATGTCCCTGCCCTTTGTACACACCGCCCGTCGCTCCT
ACCGATCGAATGATACGGTAAAGTTAACGGATAGTTTTATCTTTGGCAACATTGATATAAATTAAAAGTTATTTAAATC
TCATTGTTAGAGGAAGGAGAAGTCGAACC

>MK322959.1 *Dictyostelium barbarae* 1-5
GTCTCAGATAGCCATGCATGTCTAAGTATAAATTCTTGTACGATGAAACTGCAGACGGCTCATTACAACAGTGATAAA
CTAATAGACTTTCGGGTTTTTAACCTTTTGGATAACCGCAGTAAATCGGGGCTAATACATACAAGCGATGGGTGACTG
GCAACGGAAGCTCAGCGATTATTAGCATTACTACCAATACCTTCGGGTCTTGTGGTGAAACCGAATAATATTGCAGAT
CGAGGATTTATCTTCGACAAGTCTACTGTGTCACTGCCCTATCAACTTTCGATGGTACGGTATTGGCCTACCATGGTT
GTAACGGGTAACGGGGAATTAGGGTTCGATTCCGGAGAGGGAGCCTGAGAAATGGCTACCACTTCTACGGAAGGCA
GCAGGCGCGCAAATTACTCAATCCCAATACGGGGAAGTAGTGACAATAAATATCAATACCTATCCTTAACGGAGGGC
AATTGAAATGAACACAAATTAAAACTCTTAATTAACACAATTGGAGGGCAAGTCTGGTGCCAGCAGCCGCGGTAA
TTCCAGCTCCAATAGCATATACTAAAGTTGTTGCAGTTAAAAAGCTCGTAGTTGAAGTTAAAGGTTTACTGGGCTAA
AGTTATTTGCCACTCTGGTGGTTAAATCAACTCCAGTATTCCTTTTTTTAATAGCTCTGCTTCTAGCATCTTTGATGTTA
GTTGTTTGGGCATTTCACTGTGAGAAAATTGTGGTGTTTAAAGCAGGCGTCTCGCCTGATCTTTTGCAGCATGGTATG
ATGAAACATGACATTTTACGCTATTGGTTTGCGTCTAAAGTGTAATGATTAATAGGGATGGATGGGGGTGTTCATATTG
GTGGGCGAGAGGTGAAATTCGTTGACCCTATCAAGATGAACTTCTGCGAAAGCATTCACCAAATACTTCCCCATTAA
TCAAGAACGAAAGTTTGGGGATCGAAGACGATCAGATACCGTCGTAGTCCAAACTATAAACTATGTCGACCAGGGA
TCGGTCAAAATTTTTTTAAAATTTGATCGGCACCTTGTGAGAAATCATGAGTGTTTAGATTCCGGGGGGAGTATGGTC
GCAAGTCTGAAACTTAAAGGAATTGACGGAAGGGCACACAATGGAGTGGAGCCTGCGGCTTAATTTGACTCAACTC
GGGAAAACTTACCAAGCTAAGATATAGTAAGGATTGACAGACTAAAAGATCTTTCATGATTCTATAAGTGGTGGTGC
ATGGTCGTTCTTAGTTGGTGGAGCGATTTGTCTGGTCAATTCCGATAACGGACGAGACCTCGACCTGCTAACTAGTA
GTATTTATTAGCCGATATGGGCGATAGCTTCTCTGGGGTTTGGAATGATTTCGGTCATCTCCTACTTCAAGGAGTGTGT
AGTCTGGCTTGATAGGTACGAATTAAAAAACTTCTTAGAGGGACTACCTGCCTCAAGCAGGCGGAAGTCCGAGGCA
ATAACAGGTCTGTGATGCCCTTAGATACCTTGGGCCGCACGCGCGCTACAATGTAGAAAACAAAAAGGCTCCTGGTC
CGGAAGGATTGGGTAATCATTTGAATTTTCTACGTAACTGGGATTGATCTTTGTAATTATTGATCATCAACGAGGAATT
CCTTGTAAGCGCAGGTCATTACCCTGTGCTGAATATGTCCCTGCCCTTTGTACACACCGCCCGTCGCTCCTACCGATC
GAATGATACGGTAAAGTTAACGGATCGTTTTATCTGTGGCAACACTGATATAAACTAAAAGTTATTTAAATCTCATTGT
TTAGAGGAAGGAGAAGTCGTAACAAGGTATCCGTAGGTGAACCTGCAGAAGGATCAA

>JX173878.1 *Dictyostelium barbibulus* Sweden-4R
AACCTGGTTGATCCTGCCAGTAGTCATATGCTTGTCTCAAAGATTAAGCCATGCATGTCTAAGTATAAATTCTTGTACG
ATGAAACTGCAGACGGCTCATTACAACAGTGATAAACTAATAGACTTTCGGGTTTTTAACCTTTTGGATAACCGCAGT
AAATCGGGGCTAATACATAGAAGCGATGGGTGACTGGCAACGGAATCTCAGCGATTATTAGCATTACTACCAATACCT
TCGGGTCTTGTGGTGAAACCGAATAATATTGCAGATCGAGGATTTATCTTCGACAAGTCTACTGTGTCACTGCCCTAT
CAACTTTCGATGGTACGGTATTGGCCTACCATGGTTGTAACGGGTAACGGGGAATTAGGGTTCGATTCCGGAGAGGG
AGCCTGAGAAATGGCTACCACTTCTACGGAAGGCAGCAGGCGCGCAAATTACTCAATCCCAATACGGGGAAGTAGT
GACAATAAATATTAATACCTATCCTTAATGGAGGGCAATTGAAATGAACACAAATTAAAACTCTTAATTAACACAATT
GGAGGGCAAGTCTGGTGCCAGCAGCCGCGGTAATTCCAGCTCCAATAGCATATACTAAAGTTGTTGCAGTTAAAAAG
CTCGTAGTTGAAGTCAAAGGTTTGTTGGGTCAAAGTTATTTGCCGCTCTGGTGGTTAAATATACTCCAGCATCTCTTT
TTTTAATAGTTCAGCTTGTATTAACTTTGTTGGTACTTGTTTGGACATTTCACTGTGAGAAAATTGTGGTGTTTAAAGC
AGGCGTCTCGCCTGATCTTTTGCAGCATGGTATGATGAAACATGACATTTTACGCTATTGGTTTGCGTCTAAAGTGTA
ATGATTAATAGGGATGGATGGGGGTGTTCATATTGGTGGGCGAGAGGTGAAATTCGTTGACCCTATCAAGATGAACTT
CTGCGAAAGCATTCACCAAATACTTCCCCATTAATCAAGAACGAAAGTTTGGGGATCGAAGACGATCAGATACCGTC
GTAGTCCAAACTATAAACTATGTCGACCAGGGATCGGTTAAAATTTTTTTAAAATTTAATCGGCACCTTGTGAGAAAT
CACGAGTGTTTAGATTCCGGGGGGAGTATGGTCGCAAGTCTGAAACTTAAAGGAATTGACGGAAGGGCACACAATG
GAGTGGAGCCTGCGGCTTAATTTGACTCAACTCGGGAAAACTTACCAAGCTAAGATATAGTAAGGATTGACAGACTA
AAAGATCTTTCATGATTCTATAAGTGGTGGTGCATGGTCGTTCTTAGTTGGTGGAGCGATTTGTCTGGTCAATTCCGA
TAACGGACGAGACCTCGACCTGCTAACTAGTAGTATTTATTAGTCAATATGGGCGATAGCTTTTCTGGGATGTAGAGC
GACTTCGGTTGTTTCTTATTTCAAGGAGTGTGTAGTCTGGCTTGATAGGTACGAATTAAAAAAACTTCTTAGAGGGA
CTACCTGCCTCAAGCAGGCGGAAGTCCGAGGCAATAACAGGTCTGTGATGCCCTTAGATACCTTGGGCCGCACGCG
CGCTACAATGTAGAAAACAAAAAGGTTCCTGGTCCGGAAGGATTGGGTAATCAATTGAATTTTCTACGTAACTGGGA
TTGATCTTTGTAATTATTGATCATCAACGAGGAATTCCTTGTAAGCGTAGGTCATTACCCTATGCTGAATATGTCCCTG
CCCTTTGTACACACCGCCCGTCGCTCCTACCGATCGAATGATACGGTAAAGTTAACGGATCGTTTTATCTTTGGCAAC
ATTGATATAAATTAAAAGTTATTTAAATCTCATTGTTAGAGGAAGGAGAAGTCGTAACAAGGTATCCGTAGGTGAACC
TGCAGAAGGATCA

>AM168030.1 *Dictyostelium brefeldianum* TNS-C-115
AACCTGGTTGATCCTGCCAGTAGTCATATGCTTGTCTCAAAGATTAAGCCATGCATGTCTAAGTATAAATTCTTGTACG
ATGAAACTGCAGACGGCTCATTACAACAGTGATAAACTGCTAGACTTTCGGGTTTTTAACCTTTTGGATAACCGCAG
TAAATCGGGGCTAATACATAGAAGCGATGGGCGACTGGTAACGGAAGCTCAGCGATTATTAGCATAACTACCAATAC
CTTCGGGTCTTGTGGTGAAACCGAATAATATTGCAGATCGAAGATTTATCTTCGACAAGTCTAATGTGTCACTGCCCT
ATCAACTTTCGATGGTACGGTATTGGCCTACCATGGTTGTAACGGGTAACGGGGAATTAGGGTTCGATTCCGGAGAG
GGAGCCTGAGAAATGGCTACCACTTCTACGGAAGGCAGCAGGCGCGCAAATTACTCAATCCCAATACGGGGAAGTA
GTGACAATAAATATCAATACCTATCCTTAATGGAGGGCAATTGAAATGAACACAAATTAAAACTCTTAATTAACACAA
TTGGAGGGCAAGTCTGGTGCCAGCAGCCGCGGTAATTCCAGCTCCAATAGCATATACTAAAGTTGTTGCAGTTAAAA
AGCTCGTAGTTGAAGTTAAAGGTTTATTGGGTCAAAGCTATTTGCCGCTCTGGTGGTTAAATATGCTCCAGTATTTCT
TTTTTTTGAAAAATTGTTCAGCTTGTATCAACTTTGTTGGTACTCGTTTGGACATTTCACTGTGAGAAAATTGTGGTG
TTTAAAGCAGGCGTCTCGCCTGATCTTTTGCAGCATGGTATGATGAAACATGACATTTTGCGCTATTGGTTTGCGTCT
AAAGTGTAATGATTAATAGGGATGGATGGGGGTGTTCATATTGGTGGGCGAGAGGTGAAATTCGTTGACCCTATCAA
GATGAACTTCTGCGAAAGCATTCACCAAATACTTCTCCATTAATCAAGAACGAAAGTTTGGGGATCGAAGACGATCA
GATACCGTCGTAGTCCAAACTATAAACTATGTCGACCAGGGATCGGTTAAAATTTTTTTAAAATTTAATCGGCACCTT
GTGAGAAATCACGAGTGTTTAGATTCCGGGGGGAGTATGGTCGCAAGTCTGAAACTTAAAGGAATTGACGGAAGGG
CACACAATGGAGTGGAGCCTGCGGCTTAATTTGACTCAACTCGGGAAAACTTACCAAGCTAAGATATAGTTAGGATT
GACAGACTAAAAGATCTTTCATGATTCTATAAGTGGTGGTGCATGGTCGTTCTTAGTTGGTGGAGCGATTTGTCTGGT
CAATTCCGATAACGGACGAGACCTCGACCTGCTAACTAGTAGTATTTATTAGTCAATATGGGCGATAGCTTTTCTGGG
ATTTAGAGCGATTTCGGTCGTTTCTGGTTTCAAGGAGTGTGTAGTCTGACTTGATAGGTACGAATTAAAAAAACTTCT
TAGAGGGACTACCTGCCTCAAGCAGGCGGAAGTCCGAGGCAATAACAGGTCTGTGATGCCCTTAGATACCTTGGGC
CGCACGCGCGCTACAATGTATAAAACAAAAAGGTTCCTGGTCCGGAAGGATTGGGTAATCAATTGAATTTTCTACGT
AACTGGGATTGATCTTTGTAATTATTGATCATAAACGAGGAATTCCTTGTAAGCGTAGGTCATTACCCTATGCTGAATA
TGTCCCTGCCCTTTGTACACACCGCCCGTCGCTCCTACCGATCGAATGATACGGTAAAGTTAACGGATAGTTTTATCT
TTGGCAACATCGATATAAATTAAAAGTTATTTAAATCTCATTGTTTAGAGGAAGGAGAAGTCGTAACAAGGTATCCGT
AGGTGAACCTGCAGAAGGATCAA

>AM168031.1 *Dictyostelium brunneum* WS700
AACCTGGTTGATCCTGCCAGTAGTCATATGCTTGTCTCAAAGATTAAGCCATGCATGTCTAAGTATAAATTCTTGTACG
ATGAAACTGCAGACGGCTCATTACAACAGTGATAAACTAATAGACTTTCGGGTTTTTAACCTTTTGGATAACCGCAGT
AAATCGGGGCTAATACATAGAAGCGATGGGTGACTGGCAACGGAAGCTCAGCGATTATTAGCATTACTACCAATACC
TTCGGGTCTTGTGGTGAAACCGAATAATATTGCAGATCGAGGATTTATCTTCGACAAGTCTACTGTGTCACTGCCCTA
TCAACTTTCGATGGTACGGTATTGGCCTACCATGGTTGTAACGGGTAACGGGGAATTAGGGTTCGATTCCGGAGAGG
GAGCCTGAGAAATGGCTACCACTTCTACGGAAGGCAGCAGGCGCGCAAATTACTCAATCCCAATACGGGGAAGTAG
TGACAATAAATATCAATACCTATCCTTTTTGGAGGGCAATTGAAATGAACACAAATTAAAACTCTTAATTAACACAAT
TGGAGGGCAAGTCTGGTGCCAGCAGCCGCGGTAATTCCAGCTCCAATAGCATATACTAAAGTTGTTGCAGTTAAAAA
GCTCGTAGTTGAAGTTAAGGGTTTATCGGGCTAAAGTTATTTGCCACTTTGTGGTTAAATTCAACTCCGGTATTCCTTT
TCTTAATAGTTCAGCTTCTATTATCTTTGATAGTAGTTGTTTGGGCATTTCACTGTGAGAAAATTGTGGTGTTTAAAGC
AGGCGTCTCGCCTGATCTTTTGCAGCATGGTATGATGAAACATGACATTTTACGCTATTGGTTTGCGTCTAAAGTGTA
ATGATTAATAGGGATGGATGGGGGTGTTCATATTGGTGGGCGAGAGGTGAAATTCGTTGACCCTATCAAGATGAACTT
CTGCGAAAGCATTCACCAAATACTTCCCCATTAATCAAGAACGAAAGTTTGGGGATCGAAGACGATCAGATACCGTC
GTAGTCCAAACTATAAACTATGTCGACCAGGGATCGGTTAAAATTTTTTTAAAATTTAATCGGCACCTTGTGAGAAAT
CACGAGTGTTTAGATTCCGGGGGGAGTATGGTCGCAAGTCTGAAACTTAAAGGAATTGACGGAAGGGCACACAATG
GAGTGGAGCCTGCGGCTTAATTTGACTCAACTCGGGAAAACTTACCAAGCTAAGATATAGTAAGGATTGACAGACTA
AAAGATCTTTCATGATTCTATAAGTGGTGGTGCATGGTCGTTCTTAGTTGGTGGAGCGATTTGTCTGGTCAATTCCGA
TAACGGACGAGACCTCGACCTGCTAACTAGTAGTATTTATTAGTCAACATAGGCGATAGCTTTTCTGGGGTTTGGAAT
GATTTCGGTCATCTCCTGCTTCAAGGAGTGTGTAGTCCGGCTTGATAGGTACGAATTCAAAAACTTCTTAGAGGGAC
TACCTGCCTCAAGCAGGCGGAAGTCCGAGGCAATAACAGGTCTGTGATGCCCTTAGATACCTTGGGCCGCACGCGC

GCTACAATGTAGAAAACAAAAAGGTTCCTGGTCCGGAAGGATTGGGTAATCAATTGAATTTTCTACGTAACTGGGAT
TGATCTTTGTAATTATTGATCATCAACGAGGAATTCCTTGTAAGCGCAAGTCATTACCTTGTGCTGAATATGTCCCTGC
CCTTTGTACACACCGCCCGTCGCTCCTACCGATCGAATGATACGGTAAAGTTAACGGATCGTTTTATTTGTGGCAACA
CATTATAAGACTAAAAGTTATTTAAATCTCATTGTTTAGAGGAAGGAGAAGTCGTAACAAGGTATCCGTAGGTGAAC
CTGCAGAAGGATCAA

>AM168032.1 *Dictyostelium capitatum* 91HO-50
AACCTGGTTGATCCTGCCAGTAGTCATATGCTTGTCTCAAAGATTAAGCCATGCATGTCTAAGTATAAATTCTTGTACG
ATGAAACTGCAGACGGCTCATTACAACAGTGATAAACTGCTAGACTTTCGGGTTTTTAACCTTTTGGATAACCGCAG
TAAATCGGGGCTAATACATAGAAGCGATGGGCGACTGGTAACGGAAGCTCAGCGATTATTAGCATAACTACCAATAC
CTTCGGGTCTTGTGGTGAAACCGAATAATATTGCAGATCGAAGATTTATCTTCGACAAGTCTAATGTGTCACTGCCCT
ATCAACTTTCGATGGTACGGTATTGGCCTACCATGGTTGTAACGGGTAACGGGGAATTAGGGTTCGATTCCGGAGAG
GGAGCCTGAGAAATGGCTACCACTTCTACGGAAGGCAGCAGGCGCGCAAATTACTCAATCCCAATACGGGGAAGTA
GTGACAATAAATATCAATACCTATCCTTAATGGAGGGCAATTGAAATGAACACAAATTAAAACTCTTAATTAACACAA
TTGGAGGGCAAGTCTGGTGCCAGCAGCCGCGGTAATTCCAGCTCCAATAGCATATACTAAAGTTGTTGCAGTTAAAA
AGCTCGTAGTTGAAGTTAAAGGTTTATTGGGTCAAAGCTATTTGCCGCTCTGGTGGTTAAATATGCTCCAGTATTTCT
TTTTTTTGAAAAATTGTTCAGCTTGTATCAACTTTGTTGGTACTCGTTTGGACATTTCACTGTGAGAAAATTGTGGTG
TTTAAAGCAGGCGTCTCGCCTGATCTTTTGCAGCATGGTATGATGAAACATGACATTTTGCGCTATTGGTTTGCGTCT
AAAGTGTAATGATTAATAGGGATGGATGGGGGTGTTCATATTGGTGGGCGAGAGGTGAAATTCGTTGACCCTATCAA
GATGAACTTCTGCGAAAGCATTCACCAAATACTTCTCCATTAATCAAGAACGAAAGTTTGGGGATCGAAGACGATCA
GATACCGTCGTAGTCCAAACTATAAACTATGTCGACCAGGGATCGGTTAAAATTTTTTTAAAATTTAATCGGCACCTT
GTGAGAAATCACGAGTGTTTAGATTCCGGGGGGAGTATGGTCGCAAGTCTGAAACTTAAAGGAATTGACGGAAGGG
CACACAATGGAGTGGAGCCTGCGGCTTAATTTGACTCAACTCGGGAAAACTTACCAAGCTAAGATATAGTTAGGATT
GACAGACTAAAAGATCTTTCATGATTCTATAAGTGGTGGTGCATGGTCGTTCTTAGTTGGTGGAGCGATTTGTCTGGT
CAATTCCGATAACGGACGAGACCTCGACCTGCTAACTAGTAGTATTTATTAGTCAATATGGGCGATAGCTTTTCTGGG
ATTTAGAGCGATTTCGGTCGTTTCTGGTTTCAAGGAGTGTGTAGTCTGACTTGATAGGTCCGAATTAAAAAAACTTCT
TAGAGGGACTACCTGCCTCAAGCAGGCGGAAGTCCGAGGCAATAACAGGTCTGTGATGCCCTTAGATACCTTGGGC
CGCACGCGCGCTACAATGTATAAAACAAAAAGGTTCCTGGTCCGGAAGGATTGGGTAATCAATTGAATTTTCTACGT
AACTGGGATTGATCTTTGTAATTATTGATCATAAACGAGGAATTCCTTGTAAGCGCAGGTCATTACCCTGTGCTGAAT
ATGTCCCTGCCCTTTGTACACACCGCCCGTCGCTCCTACCGATCGAATGATACGCTAAAGTTAACGGATAGTTTTATC
TTTGGCAACATTGATATAAATTAAAAGTTATTTAAATCTCATTGTTTAGAGGAAGGAGAAGTCGTAACAAGGTATCCG
TAGGTGAACCTGCAGAAGGATCAA

>GQ496159.1 *Dictyostelium chordatum*
AGATTAGCCATGCATGTCTAAGTATAAATTCTTGTACGATGAAACTGCAGACGGCTCATTACAACAGTGATAAACTAA
TAGACTTTCGGGTTTTTAACCTTTTGGATAACCGCAGTAAATCGGGGCTAATACATAGAAGCGATGGGTGACTGGCA
ACGGAATCTCAGCGATTATTAGCATTACTACCAATACCTTCGGGTCTTGTGGTGAAACCGAATAATATTGCAGATCGA
GGATTTATCTTCGACAAGTCTACTGTGTCACTGCCCTATCAACTTTCGATGGTACGGTATTGGCCTACCATGGTTGTAA
CGGGTAACGGGGAATTAGGGTTCGATTCCGGAGAGGGAGCCTGAGAAATGGCTACCACTTCTACGGAAGGCAGCA
GGCGCGCAAATTACTCAATCCCAATACGGGGAAGTAGTGACAATAAATATTAATACCTATCCTTAATGGAGGGCAATT
GAAATGAACACAAATTAAAACTCTTAATTAACACAATTGGAGGGCAAGTCTGGTGCCAGCAGCCGCGGTAATTCCA
GCTCCAATAGCATATACTAAAGTTGTTGCAGTTAAAAAGCTCGTAGTTGAAGTCAAAGGTTTGTTGGGTCAAAGTTA
TTTGCCGCTCTGGTGGTTAAATATACTCCAGCATCTCTTTTTTTAATAGTTCAGCTTGTATTGACTTTGTTAGTACTTGT
TTGGACATTTCACTGTGAGAAAATTGTGGTGTTTAAAGCAGGCGTCTCGCCTGATCTTTTGCAGCATGGTATGATGA
AACATGACATTTTACGCTATTGGTTTGCGTCTAAAGTGTAATGATTAATAGGGATGGATGGGGGTGTTCATATTGGTGG
GCGAGAGGTGAAATTCGTTGACCCTATCAAGATGAACTTCTGCGAAAGCATTCACCAAATACTTCCCCATTAATCAA
GAACGAAAGTTTGGGGATCGAAGACGATCAGATACCGTCGTAGTCCAAACTATAAACTATGTCGACCAGGGATCGG
TTAAAATTTTTTTAAAATTTAATCGGCACCTTGTGAGAAATCACGAGTGTTTAGATTCCGGGGGGAGTATGGTCGCAA
GTCTGAAACTTAAAGGAATTGACGGAAGGGCACACAATGGAGTGGAGCCTGCGGCTTAATTTGACTCAACTCGGGA
AAACTTACCAAGCTAAGATATAGTAAGGATTGACAGACTAAAAGATCTTTCATGATTCTATAAGTGGTGGTGCATGGT
CGTTCTTAGTTGGTGGAGCGATTTGTCTGGTCAATTCCGATAACGGACGAGACCTCGACCTGCTAACTAGTAGTATTT
ATTAGTCAATATGGGCGATAGCTTTTCTGGGATGTAGAGCGATTTCGGTCGTTTCTTATTTCAAGGAGTGTGTAGTCTG
GCTTGATAGGTACGAATTAAAAAAACTTCTTAGAGGGACTACCTGCCTCAAGCAGGCGGAAGTCCGAGGCAATAAC
AGGTCTGTGATGCCCTTAGATACCTTGGGCCGCACGCGCGCTACAATGTAGAAAACAAAAAGGTTCCTGGTCCGGA
AGGATTGGGTAATCAATTGAATTTTCTACGTAACTGGGATTGATCTTTGTAATTATTGATCATCAACGAGGAATTCCTT
GTAAGCGTAGGTCATTACCCTATGCTGAATATGTCCCTGCCCTTTGTACACACCGCCCGTCGCTCCTACCGATCGAAT
GATACGGTAAAGTTAACGGATCGTTTTATCTTTGGCAACATTGATATAAATTAAAAGTTATTTAAATCTCATGTTAGAG
AAGGAGAAGT

>AM168033.1 *Dictyostelium citrinum* OH494
AACCTGGTTGATCCTGCCAGTAGTCATATGCTTGTCTCAAAGATTAAGCCATGCATGTCTAAGTATAAATTCTTGTACG
ATGAAACTGCAGACGGCTCATTACAACAGTGATAAACTAATAGACTTTCGGGTTTTACCTTTTGGATAACCGCAGTA
AATCGGGGCTAATACATACAAGCGATGGGTGACTGGCAACGGAAGCTCAGCGATTATTAGCGTTCTACCAATACCTT
CGGGTTTTGGGTGAGACCGAATAATATTGCAGATCGAGGATTTATCTTCGACAAGTCTACTGTGTCACTGCCCTATCA
ACTTTCGATGGTACGGTATTGGCCTACCATGGTTGTAACGGGTAACGGGGAATTAGGGTTCGATTCCGGAGAGGGAG
CCTGAGAAATGGCTACCACTTCTACGGAAGGCAGCAGGCGCGCAAATTACTCAATCCCAATACGGGGAAGTAGTGA
CAATAAATATCAATACCTATCCTTTTTGGAGGGCAATTGAAATGAACACAAATTAAAACTCTTAATTAACACAATTGG
AGGGCAAGTCTGGTGCCAGCAGCCGCGGTAATTCCAGCTCCAATAGCATATACTAAAGTTGTTGCAGTTAAAAAGCT
CGTAGTTGAAGTTTAAGGTTTACTGGGTTTATGTCGTTTGTCACTTTTGTGGCCAAACTGACACCAGTATCTCTTTCT
TAATAGTTCAGCTTGCATTATCTTTGATAGTGCTTGTTTGGACATTTCACTGTGAGAAAATTGTGGTGTTTAAAGCAG
GCGTCTCGCCTGATCTTTTGCAGCATGGTATGATGAAACATGACATTTTACGCTATTGGTTTGCGTTTAAAGTGTAATG
ATTAATAGGGATGGATGGGGGTGTTCATATTGGTGGGCGAGAGGTGAAATTCGTTGACCCTATCAAGATGAACTTCT
GCGAAAGCATTCACCAAATACTTCCCCATTAATCAAGAACGAAAGTTTGGGGATCGAAGACGATCAGACACCGTCG
TAGTCCAAACTATAAACTATGTCGACCAGGGATCGGTTAAAATTTTTTTAAAATTTAATCGGCACCTTGTGAGAAATC
ATGAGTGTTTAGATTCCGGGGGGAGTATGGTCGCAAGTCTGAAACTTAAAGGAATTGACGGAAGGGCACACAATGG

AGTGGAGCCTGCGGCTTAATTTGACTCAACTCGGGAAAACTTACCAAGCTAAGATATAGTAAGGATTGACAGACTAA
AAGATCTTTCATGATTCTATAAGTGGTGGTGCATGGTCGTTCTTAGTTGGTGGAGCGATTTGTCTGGTCAATTCCGATA
ACGGACGAGACCTCGACCTGCTAACTAGTAGTATTTATTAGTCGATATGGACGATAGCTTCTCTGGGGTTTGGAATGA
TTTCGGTCATCTCCTGCTTCAAGGAGTGTGTAGTCTGACTTGATAGGTACGAATCAAAACTTCTTAGAGGGACTACC
TGCCTCAAGCAGGCGGAAGTCCGAGGCAATAACAGGTCTGTGATGCCCTTAAATACCTTGGGCCGCACGCGCGCTA
CAATGTAGGAAACAAAAAGGCTCCTGGTCCGGAAGGATTGGGTAATCATTTGAATTTCCTACGTAACTGGGCTTGAT
CTTTGTAATTATTGATCATAAACGAGGAATTCCTTGTAAGCGTAAGTCATTACCTTATGCTGAATATGTCCCTGCCCTT
TGTACACACCGCCCGTCGCTCCTACCGATCGAATGATACGGTAAAGTTAACGGATCGTTTTATCTGTGGCAACACTGA
TATAAATTAAAAGTTATTTAAATCTCATTGTTTAGAGGAAGGAGAAGTCGTAACAAGGTATCCGTAGGTGAACCTGCA
GAAGGATCAA

>AM168034.1 *Dictyostelium clavatum* TNS-C-189
AACCTGGTTGATCCTGCCAGTAGTCATATGCTTGTCTCAAAGATTAAGCCATGCATGTCTAAGTATAAATTTTTGTACG
ATGAAACTGCAGACGGCTCATTACAACAGTGATAAACTAATAGACTTTCGGGTTTTTAACCTTTTGGATAACCGCAGT
AAATCGGGGCTAATACATAGAAGCGATGGGTGACTGGCAACGGAAGCTCAGCGATTATTAGCATTACTACCAATACC
TTCGGGTCTTGTGGTGAAACCGAATAATATTGCAGATCGAGGATTTATCTTCGACAAGTCTACTGTGTCACTGCCCTA
TCAACTTTCGATGGTACGGTATTGGCCTACCATGGTTGTAACGGGTAACGGGGAATTAGGGTTCGATTCCGGAGAGG
GAGCCTGAGAAATGGCTACCACTTCTACGGAAGGCAGCAGGCGCGCAAATTACTCAATCCCAATACGGGGAAGTAG
TGACAATAAATATCAATACCTATCCTTAACGGAGGGCAATTGAAATGAACACAAATTAAAACTCTTAATTAACACAAT
TGGAGGGCAAGTCTGGTGCCAGCAGCCGCGGTAATTCCAGCTCCAATAGCATATACTAAAGTTGTTGCAGTTAAAAA
GCTCGTAGTTGAAGTTAAAGGTTTACTGGGCTAAAGTTATTTGCCGCTCTGGTGGTTAAATCAACTCCAGTATCTCTT
TTTTTAATAGTTCAGCTTCTATTATCTTTGATAGTAGTTGTTTGGACATTTCACTGTGAGAAAATTGTGGTGTTTAAAG
CAGGCGTTTCGCCTGATCTTTTGCAGCATGGTATGATGGAACATGACATTTTACGCTATTGGTTTGCGTCTAAAGTGT
AATGATTAATAGGGATGGATGGGGGTGTTCATATTGGTGGGCGAGAGGTGAAATTCGTTGACCCTATCAAGATGAAC
TTCTGCGAAAGCATTCACCAAATACTTCCCCATTAATCAAGAACGAAAGTTTGGGGATCGAAGACGATCAGATACCG
TCGTAGTCCAAACTATAAACTATGTCGACCAGGGATCGGTTAAAATTTTTTTAAAATTTAATCGGCACCTTGTGAGAA
ATCACGAGTGTTTAGATTCCGGGGGGAGTATGGTCGCAAGTCTGAAACTTAAAGGAATTGACGGAAGGGCACACAA
TGGAGTGGAGCCTGCGGCTTAATTTGACTCAACTCGGGAAAACTTACCAAGCTAAGATATAGTAAGGATTGACAGAC
TAAAAGATCTTTCATGATTCTATAAGTGGTGGTGCATGGTCGTTCTTAGTTGGTGGAGCGATTTGTCTGGTCAATTCC
GATAACGGACGAGACCTCGACCTGCTAACTAGTAGTATTTATTAGTCAATATGGGCGATAGCTTCTCTGGGGTTTGGA
ATGGTTTCGGCCATCTCCTGCTTCAAGGAGTGTGTAGTCTGACTTGATAGGTACGAATTAAAAAACTTCTTAGAGGG
ACTACCTGCCTCAAGCAGGCGGAAGTCCGAGGCAATAACAGGTCTGTGATGCCCTTAGATACCTTGGGCCGCACGC
GCGCTACAATGTAGAAAACAAAAAGGTTCCTGGTCCGGAAGGATTGGGTAATCAATTGAATTTTCTACGTAACTGGG
ATTGATCTTTGTAATTATTGATCATCAACGAGGAATTCCTTGTAAGCGTAGGTCATTACCCTATGCTGAATATGTCCCT
GCCCTTTGTACACACCGCCCGTCGCTCCTACCGATCGAATGATACGGTAAAGTTAACGGATCGTTTTATCTGTGGCAA
CACTGATATAAACTAAAAGTTATTTAAATCTCATTGTTTAGAGGAAGGAGAAGTCGTAACAAGGTATCCGTAGGTGA
ACCTGCAGAAGGATCAA

>AM168035.1 *Dictyostelium clavatum* TNS-C-220
AACCTGGTTGATCCTGCCAGTAGTCATATGCTTGTCTCAAAGATTAAGCCATGCATGTCTAAGTATAAATTCTTGTACG
ATGAAACTGCAGACGGCTCATTACAACAGTGATAAACTAATAGACTTTCGGGTTTTTAACCTTTTGGATAACCGCAGT
AAATCGGGGCTAATACATAGAAGCGATGGGTGACTGGCAACGGAAGCTCAGCGATTATTAGCATTACTACCAATACC
TTCGGGTCTTGTGGTGAAACCGAATAATATTGCAGATCGAGGATTTATCTTCGACAAGTCTACTGTGTCACTGCCCTA
TCAACTTTCGATGGTACGGTATTGGCCTACCATGGTTGTAACGGGTAACGGGGAATTAGGGTTCGATTCCGGAGAGG
GAGCCTGAGAAATGGCTACCACTTCTACGGAAGGCAGCAGGCGCGCAAATTACTCAATCCCAATACGGGGAAGTAG
TGACAATAAATATCAATCCCTATCCTTAACGGAGGGCAATTGAAATGAACACAAATTAAAACTCTTAATTAACACAAT
TGGAGGGCAAGTCTGGTGCCAGCAGCCGCGGTAATTCCAGCTCCAATAGCATATACTAAAGTTGTTGCAGTTAAAAA
GCTCGTAGTTGAAGTTAAAGGTTTACTGGGCTAAAGTTATTTGCCGCTCTGGTGGTTAAATCAACTCCAGTATCTCTT
TTTTTAATAGTTCAGCTTCTATTATCTTTGATAGTAGTTGTTTGGACATTTCACTGTGAGAAAATTGTGGTGTTTAAAG
CAGGCGTTTCGCCTGATCTTTTGCAGCATGGTATGATGGAACATGACATTTTACGCTATTGGTTTGCGTCTAAAGTGT
AATGATTAATAGGGATGGATGGGGGTGTTCATATTGGTGGGCGAGAGGTGAAATTCGTTGACCCTATCAAGATGAAC
TTCTGCGAAAGCATTCACCAAATACTTCCCCATTAATCAAGAACGAAAGTTTGGGGATCGAAGACGATCAGATACCG
TCGTAGTCCAAACTATAAACTATGTCGACCAGGGATCGGTTAAAATTTTTTTAAAATTTAATCGGCACCTTGTGAGAA
ATCACGAGTGTTTAGATTCCGGGGGGAGTATGGTCGCAAGTCTGAAACTTAAAGGAATTGACGGAAGGGCACACAA
TGGAGTGGAGCCTGCGGCTTAATTTGACTCAACTCGGGAAAACTTACCAAGCTAAGATATAGTAAGGATTGACAGAC
TAAAAGATCTTTCATGATTCTATAAATGGTGGTGCATGGTCGTTCTTAGTTGGTGGAGCGATTTGTCTGGTCAATTCCG
ATAACGGACGAGACCTCGACCTGCTAACTAGTAGTATTTATTAGTCAATATGGGCGATAGCTTCTCTGGGGTTTGGAA
TGGCTGCGGTCATCTCCTGCTTCAAGGAGTGTGTAGTCTGACTTGATAGGTACGAATTAAAAAACTTCTTAGAGGGA
CTACCTGCCTCAAGCAGGCGGAAGTCCGAGGCAATAACAGGTCTGTGATGCCCTTAGATACCTTGGGCCGCACGCG
CGCTACAATGTAGAAAACAAAAAGGTTCCTGGTCCGGAAGGATTGGGTAATCAATTGAATTTTCTACGTAACTGGGA
TTGATCTTTGTAATTATTGATCATCAACGAGGAATTCCTTGTAAGCGTAGGTCATTACCCTATGCTGAATATGTCCCTG
CCCTTTGTACACACCGCCCGTCGCTCCTACCGATCGAATGATACGGTAAAGTTAACGGATCGTTTTATCTGTGGCAAC
ACTGATATAAACTAAAAGTTATTTAAATCTCATTGTTTAGAGGAAGGAGAAGTCGTAACAAGGTATCCGTAGGTGAA
CCTGCAGAAGGATCAA

>AM168037.1 *Dictyostelium crassicaule* 93HO-33
AACCTGGTTGATCCTGCCAGTAGTCATATGCTTGTCTCAAAGATTAAGCCATGCATGTCTAAGTATAAATTCTTGTACG
ATGAAACTGCAGACGGCTCATTACAACAGTGATAAACTAATAGACTTTCGGGTTTTTAACCTTTTGGATAACCGCAGT
AAATCGGGGCTAATACATAGAAGCGATGGGTGACTGGCAACGGAATCTCAGCGATTATTAGCATAACTACCAATACC
TTCGGGTCTTGTGGTGAAACCGAATAATATTGCAGATCGAAGATTTATCTTCGACAAGTCTACTGTGTCACTGCCCTA
TCAACTTTCGATGGTACGGTATTGGCCTACCATGGTTGTAACGGGTAACGGGGAATTAGGGTTCGATTCCGGAGAGG
GAGCCTGAGAAATGGCTACCACTTCTACGGAAGGCAGCAGGCGCGCAAATTGCTCAATCCCAATACGGGGAAGTAG
TGACAATAAATATCAATACCTATCCTTAATGGAGGGCAATTGAAATGAACACAAATTAAAACTCTTAATTAACACAGT
TGGAGGGCAAGTCTGGTGCCAGCAGCCGCGGTAATTCCAGCTCCAATAGCATATACTAAAGTTGTTGCAGTTAAAAA
GCTCGTAGTTGAAGTTAAAGGTTTACTGGGCTCAAGTTATTTGCCACTCTGGTGGTTAAATATACTCCAGTATCTCTTT

TTTTTCTAATAGTTCAGCTTGTATCAACCTAGTTGGTACTCGTTTGGACATTTCACTGTGAGAAAATTGTGGTGTTTAA
AGCAGGCGTCTCGCCTGATCTTTTGCAGCATGGTATGATGAAACATGACATTTTGCGCTATTGGTTTGCGTCTAAAGT
GTAATGATTAATAGGGATGGATGGGGGTGTTCATATTGGTGGGCGAGAGGTGAAACTCGTTGACCCTATCAAGATGA
ACTTCTGCGAAAGCATTCACCAAATACTTCCCCATTAATCAAGAACGAAAGTTTGGGGATCGAAGACGATCAGATAC
CGTCGTAGTCCAAACTATAAACTATGTCGACCAGGGATCGGTTAAAATTTTTTTAAAATTTAATCGGCACCTTGTGAG
AAATCACGAGTGTTTAGATTCCGGGGGGAGTATGGTCGCAAGTCTGAAACTTAAAGGAATTGACGGAAGGGCACAC
AATGGAGTGGAGCCTGCGGCTTAATTTGACTCAACTCGGGAAAACTTACCAAGCTAAGATATAGTAAGGATTGACAG
ACTAAAAGATCTTTCATGATTCTATAAGTGGTGGTGCATGGTCGTTCTTAGTTGGTGGAGCGATTTGTCTGGTCAATT
CCGATAACGGACGAGACCTCGACCTGCTAACTAGTAGTATTTATTAGTCAATATGGGCGATAGCTTTTCTGGGGTGGA
GTAATAGCCTCACGGTTATTATTTACTTCAAGGAGTGTGTAGTCTGACTTGATAGGTACGAATTAAAAAAACTTCTTA
GAGGGACTACCTGCCTCAAGCAGGCGGAAGTCCGAGGCAATAACAGGTCTGTGATGCCCTTAGATACCTTGGGCCG
CACGCGCGCTACAATGTAGAAAACAAAAAGGTTCCTGGTCCGGAAGGATTGGGTAATCAATTGAATTTTCTACGTAA
CTGGGATTGATCTTTGTAATTATTGATCATCAACGAGGAATTCCTTGTAAGCGTAGGTCATTACCCTATGCTGAATATG
TCCCTGCCCTTTGTACACACCGCCCGTCGCTCCTACCGATCGAATGATACGGTAAACTTAACGGATCGTTTTATCTTT
GGCAACATTGATATAAACTAAAAGTTATTTAAATCTCATTGTTTAGAGGAAGGAGAAGTCGTAACAAGGTATCCGTAG
GTGAACTTGCAGAAGGTTCAA

>AM168038.1 *Dictyostelium dimigraformum* AR5b
AACCTGGTTGATCCTGCCAGTAGTCATATGCTTGTCTCAAAGATTAAGCCATGCATGTCTAAGTATAAATTCTTGTACG
ATGAAACTGCAGACGGCTCATTACAACAGTGATAAACTAATAGACTTTCGGGTTTTACCTTTTGGATAACCGCAGTA
AATCGGGGCTAATACATACAAGCGATGGGTGACTGGCAACGGAAGCTCAGCGATTATTAGCATTCTACCAATACCTTC
GGGTTTTGGGTGAGACCGAATAATATTGCAGATCGAGGATTTATCTTCGACAAGTCTACTGTGTCACTGCCCTATCAA
CTTTCGATGGTACGGTATTGGCCTACCATGGTTGTAACGGGTAACGGGGAATTAGGGTTCGATTCCGGAGAGGGAGC
CTGAGAAATGGCTACCACTTCTACGGAAGGCAGCAGGCGCGCAAATTACTCAATCCCAATACGGGGAAGTAGTGAC
AATAAATATCAATACCTATCCTTTTTGGAGGGCAATTGAAATGAACACAAATTAGAACTCTTAATTAACACAATTGGA
GGGCAAGTCTGGTGCCAGCAGCCGCGGTAATTCCAGCTCCAATAGCATATACTAAAGTTGTTGCAGTTAAAAAGCTC
GTAGTTGAAGTTTAAGGTTTACTGGGTTTTATGTGATTGTCACTTCGTGATTAATCACACCAGTATCTCTTTCTTAATA
GTTCAGCTTGTATTATCTTTGATAGTGCTTGTTTGGACATTTCACTGTGAGAAAATTGTGGTGTTTAAAGCAGGCGTC
TCGCCTGATCTTTTGCAGCATGGTATGATGAAACATGACATTTTACGCTATTGGTTTGCGTTTAAAGTGTAATGATTAA
TAGGGATGGATGGGGGTGTTCATATTGGTGGGCGAGAGGTGAAATTCCTTGACCCTATCAAGATGAACTTCTGCGAA
AGCATTCACCAAATACTTCCCCATTAATCAAGAACGAAAGTTTGGGGATCGAAGACGATCAGATACCGTCGTAGTCC
AAACTATAAACTATGTCGACCAGGGATCGGTTAAAATTTTTATAAAATTTAATCGGCACCTTGTGAGAAATCATGAGT
GTTTAGATTCCGGGGGGAGTATGGTCGCAAGTCTGAAACTTAAAGGAATTGACGGAAGGGCACACAATGGAGTGGA
GCCTGCGGCTTAATTTGACTCAACTCGGGAAAACTTACCAAGCTAAGATATAGTAAGGATTGACAGACTAAAAGATC
TTTCATGATTCTATAAGTGGTGGTGCATGGTCGTTCTTAGTTGGTGGAGCGATTTGTCTGGTCAATTCCGATAACGGA
CGAGACCTCGACCTGCTAACTAGTAGTATTTATTAGTCGATATGGACGATAGCTTTTCTGGGGTTTGGAATGATTTCGG
TCATCTCCTGCTTCAAGGAGTGTGTAGTCTGACTCGATAGGTACGAATTAAAACTTCTTAGAGGGACTACCTGCCTC
AAGCAGGCGGAAGTCCGAGGCAATAACAGGTCTGTGATGCCCTTAGATACCTTGGGCCGCACGCGCGCTACAATGT
AGGAAACAAAAAGGCTCCTGGTCCGGAAGGATTGGGTAATCATTTGAATTTCCTACGTAACTGGGCTTGATCTTTGT
AATTATTGATCATAAACGAGGAATTCCTTGTAAGCGTAAGTCATTACCTTATGCTGAATATGTCCCTGCCCTTTGTACA
CACCGCCCGTCGCTCCTACCGATCGAATGATACGGTAAAGTTAACGGATCGTTTTATCTGTGGCAACACTGATATAAA
TTAAAAGTTATTTAAATCTCATTGTTTAGAGGAAGGAGAAGTCGTAACAAGGTATCCGTAGGTGAACCTGCAGAAGG
ATCAA

>AM168071.1 *Dictyostelium discoideum* NC4
AACCTGGTTGATCCTGCCAGTAGTCATATGCTTGTCTCAAAGATTAAGCCATGCATGTCTAAGTATAAATTCTTGTACG
ATGAAACTGCAGACGGCTCATTACAACAGTGATAAACTAATAGACTTTCGGGTTTTACCTTTTGGATAACCGCAGTA
AATCGGGGCTAATACATACAAGCGATGGGTGACTGGCAACGGAAGCTCAGCGATTATTAGCATTCTACCAATGCCTT
CGGGTTTTGGGTGATACCGAATAATATTGCAGATCGAGGATTTATCTTCGACAAGTCTACTGTGTCACTGCCCTATCA
ACTTTCGATGGTACGGTATTGGCCTACCATGGTTGTAACGGGTAACGGGGAATTAGGGTTCGATTCCGGAGAGGGAG
CCTGAGAAATGGCTACCACTTCTACGGAAGGCAGCAGGCGCGCAAATTACTCAATCCCAATACGGGGAAGTAGTGA
CAATAAATATCAATACCTATCCTTTTTGGAGGGCAATTGAAATGAACACAAATTAAAACTCTTAATTAACACAATTGG
AGGGCAAGTCTGGTGCCAGCAGCCGCGGTAATTCCAGCTCCAATAGCATATACTAAAGTTGTTGCAGTTAAAAAGCT
CGTAGTTGAAGTTTAAGGTTTACCGGGTTTATGTCATTTACCACTTCGTGGTTAAATCGACACCGGTATCTCTTTCTTA
ATAGTTCAGCTTGTATTATCTTTGATAGTGCTTGTTTGGACATTTCACTGTGAGAAAATTGTGGTGTTTAAAGCAGGC
GTCTCGCCTGATCTTTTGCAGCATGGTATGATGAAACATGACATTTTACGCTATTGGTTTGCGTTTAAAGTGTAATGAT
TAATAGGGATGGATGGGGGTGTTCATATTGGTGGGCGAGAGGTGAAATTCGTTGACCCTATCAAGATGAACTTCTGC
GAAAGCATTCACCAAATACTTCCCCATTAATCAAGAACGAAAGTTTGGGGATCGAAGACGATCAGATACCGTCGTAG
TCCAAACTATAAACTATGTCGACCAGGGATCGGTTAAAATTTTTTCAAAATTTAATCGGCACCTTGTGAGAAATCATG
AGTGTTTAGATTCCGGGGGGAGTATGGTCGCAAGTCTGAAACTTAAAGGAATTGACGGAAGGGCACACAATGGAGT
GGAGCCTGCGGCTTAATTTGACTCAACTCGGGAAAACTTACCAAGCTAAGATATAGTAAGGATTGACAGACTAAAA
GATCTTTCATGATTCTATAAGTGGTGGTGCATGGTCGTTCTTAGTTGGTGGAGCGATTTGTCTGGTCAATTCCGATAAC
GGACGAGACCTCGACCTGCTAACTAGTAGTATTTATTAGTCGATATAGACGATAGCTTTTCTGGGGTTTGGAATGATTT
CGGTCATCTCCTGCTTCAAGGAGTGTGTAGTCTGACTCGATAGGTACGAATTAAAACTTCTTAGAGGGACTACCTGC
CTCAAGCAGGCGGAAGTCCGAGGCAATAACAGGTCTGTGATGCCCTTAGATACCTTGGGCCGCACGCGCGCTACAA
TGTAGGAAACAAAAAGGCTCCTGGTCCGGAAGGATTGGGTAATCATTTGAATTTCCTACGTAACTGGGCTTGATCTT
TGTAATTATTGATCATAAACGAGGAATTCCTTGTAAGCGTAAGTCATTACCTTATGCTGAATATGTCCCTGCCCTTTGT
ACACACCGCCCGTCGCTCCTACCGATCGAATGATACGGTAAAGTTAACGGATCGTTTTATCTGTGGCAACACTGATAT
AAATTAAAAGTTATTTAAATCTCATTGTTTAGAGGAAGGAGAAGTCGTAACAAGGTATCCGTAGGTGAACCTGCAG
AAGGATCAA

>AM168039.1 *Dictyostelium discoideum* V34
AACCTGGTTGATCCTGCCAGTAGTCATATGCTTGTCTCAAAGATTAAGCCATGCATGTCTAAGTATAAATTCTTGTACG
ATGAAACTGCAGACGGCTCATTACAACAGTGATAAACTAATAGACTTTCGGGTTTTACCTTTTGGATAACCGCAGTA
AATCGGGGCTAATACATACAAGCGATGGGTGACTGGCAACGGAAGCTCAGCGATTATTAGCATTCTACCAATGCCTT

CGGGTTTTGGGTGAGACCGAATAATATTGCAGATCGAGGATTTATCTTCGACAAGTCTACTGTGTCACTGCCCTATCA
ACTTTCGATGGTACGGTATTGGCCTACCATGGTTGTAACGGGTAACGGGGAATTAGGGTTCGATTCCGGAGAGGGAG
CCTGAGAAATGGCTACCACTTCTACGGAAGGCAGCAGGCGCGCAAATTACTCAATCCCAATACGGGGAAGTAGTGA
CAATAAATATCAATACCTATCCTTTTTGGAGGGCAATTGAAATGAACACAAATTAAAACTCTTAATTAACACAATTGG
AGGGCAAGTCTGGTGCCAGCAGCCGCGGTAATTCCAGCTCCAATAGCATATACTAAAGTTGTTGCAGTTAAAAAGCT
CGTAGTTGAAGTTTAAGGTTTACTGGGTTTATGTCGTTTGTCACTTTTGTGGTAAACTGACACCAGTATCTCTTTCTTA
ATAGTTCAGCTTACATTATCTTTGATAGTGTTTGTTTGGACATTTCACTGTGAGAAAATTGTGGTGTTTAAAGCAGGC
GTCTCGCCTGATCTTTTGCAGCATGGTATGATGAAACATGACATTTTACGCTATTGGTTTGCGTTTAAAGTGTAATGAT
TAATAGGGATGGATGGGGGTGTTCATATTGGTGGGCGAGAGGTGAAATTCGTTGACCCTATCAAGATGAACTTCTGC
GAAAGCATTCACCAAATACTTCCCCATTAATCAAGAACGAAAGTTTGGGGATCGAAGACGATCAGATACCGTCGTAG
TCCAAACTATAAACTATGTCGACCAGGGATCGGTTAAAATTTTTTTAAAATTTAATCGGCACCTTGTGAGAAATCATG
AGTGTTTAGATTCCGGGGGGAGTATGGTCGCAAGTCTGAAACTTAAAGGAATTGACGGAAGGGCACACAATGGAGT
GGAGCCTGCGGCTTAATTTGACTCAACTCGGGAAAACTTACCAAGCTAAGATATAGTAAGGATTGACAGACTAAAA
GATCTTTCATGATTCTATAAGTGGTGGTGCATGGTCGTTCTTAGTTGGTGGAGCGATTTGTCTGGTCAATTCCGATAAC
GGACGAGACCTCGACCTGCTAACTAGTAGTATTTATTAGTCGATATGGACGATAGCTTCTCTGGGGTTTGGAATGATT
TCGGTCATCTCCTGCTTCAAGGAGTGTGTAGTCTGACTTGATAGGTACGAATTAAAACTTCTTAGAGGGACTACCTG
CCTCAAGCAGGCGGAAGTCCGAGGCAATAACAGGTCTGTGATGCCCTTAGATACCTTGGGCCGCACGCGCGCTACA
ATGTAGGAAACAAAAAGGCTCCTGGTCCGGAAGGATTGGGTAATCATTTGAATTTCCTACGTAACTGGGCTTGATCT
TTGTAATTATTGATCATAAACGAGGAATTCCTTGTAAGCGTAAGTCATTACCTTATGCTGAATATGTCCCTGCCCTTTG
TACACACCGCCCGTCGCTCCTACCGATCGAATGATACGGTAAAGTTAACGGATCGTTTTATCTGTGGCAACACTGATA
TAAATTAAAAGTTATTTAAATCTCATTGTTTAGAGGAAGGAGAAGTCGTAACAAGGTATCCGTAGGTGAACCTGCAG
AAGGATCAA

>AM168041.1 *Dictyostelium firmibasis* TNS-C-14
AACCTGGTTGATCCTGCCAGTAGTCATATGCTTGTCTCAAAGATTAAGCCATGCATGTCTAAGTATAAATTCTTGTACG
ATGAAACTGCAGACGGCTCATTACAACAGTGATAAACTAATAGACTTTCGGGTTTTACCTTTTGGATAACCGCAGTA
AATCGGGGCTAATACATACAAGCGATGGGCGACTGGCAACGGAAGCTCAGCGATTATTAGCATTCTACCAATGCCTT
CGGGTTTTGGGTGAGACCGAATAATATTGCAGATCGAGGATTTATCTTCGACAAGTCTACTGTGTCACTGCCCTATCA
ACTTTCGATGGTACGGTATTGGCCTACCATGGTTGTAACGGGTAACGGGGAATTAGGGTTCGATTCCGGAGAGGGAG
CCTGAGAAATGGCTACCACTTCTACGGAAGGCAGCAGGCGCGCAAATTACTCAATCCCAATACGGGGAAGTAGTGA
CAATAAATATCAATGCTTATCCTTTTTGGAGGGCAATTGAAATGAACACAAATTAAAACTCTTAATTAACACAATTGG
AGGGCAAGTCTGGTGCCAGCAGCCGCGGTAATTCCAGCTCCAATAGCATATACTAAAGTTGTTGCAGTTAAAAAGCT
CGTAGTTGAAGTTAAAGGTTTATTGGGTAAACGTCGTTTGTCACTTCGTGATTAAACTGACTCCAGTATCTCTTTCTT
AATAGTTCAGCTTTCGTTATCTTTGATAGCGGTTGTTTGGACATTTCACTGTGAGAAAATTGTGGTGTTTAAAGCAGG
CGTCTCGCCTGATCTTTTGCAGCATGGTATGATGAAACATGACATTTTACGCTATTGGTTTGCGTCTAAAGTGTAATGA
TTAATAGGGATGGATGGGGGTGTTCATATTGGTGGGCGAGAGGTGAAATTCGTTGACCCTATCAAGATGAACTTCTG
CGAAAGCATTCACCAAATACTTCCCCATTAATCAAGAACGAAAGTTTGGGGATCGAAGACGATCAGATACCGTCGTA
GTCCAAACTATAAACTATGTCGACCAGGGATCGGTTAAAATTTTTTTAAAATTTAATCGGCACCTTGTGAGAAATCAC
GAGTGTTTAGATTCCGGGGGGAGTATGGTCGCAAGTCTGAAACTTAAAGGAATTGACGGAAGGGCACACAATGGAG
TGGAGCCTGCGGCTTAATTTGACTCAACTCGGGAAAACTTACCAAGCTAAGATATAGTAAGGATTGACAGACTAAAA
GATCTTTCATGATTCTATAAGTGGTGGTGCATGGTCGTTCTTAGTTGGTGGAGCGATTTGTCTGGTCAATTCCGATAAC
GGACGAGACCTCGACCTGCTAACTAGTAGTATTTATTAGTCGATATAGGCGATAGCTTTTCTGGGGTTTGGAATGATTT
CGGTCATCTCCGGCTTCAAGGAGTGTGTAGTCTGACTCGATAGGTACGAATTTAAAAACTTCTTAGAGGGACTACCT
GCCTCAAGCAGGCGGAAGTCCGAGGCAATAACAGGTCTGTGATGCCCTTAGATACCTTGGGCCGCACGCGCGCTAC
AATGTAGGAAACAAAAAGGCTCCTGGTCCGGAAGGATTGGGTAATCATTTGAATTTCCTACGTAACTGGGATTGATC
TTTGTAATTATTGATCATAAACGAGGAATTCCTTGTAAGCATAAGTCATTACCTTATGCTGAATATGTCCCTGCCCTTTG
TACACACCGCCCGTCGCTCCTACCGATCGAATGATACGGTAAAGTTAACGGATCGTTTTATCTGTGGCAACACTGATA
TAAATTAAAAGTTATTTAAATCTCATTGTTTAGAGGAAGGAGAAGTCGTAACAAGGTATCCGTAGGTGAACCTGCAG
AAGGATCAA

>GQ496161.1 *Dictyostelium gargantuum*
AACCTGGTTGATCCTGCCAGTAGTCATATGCTTGTCTCAAAGATTAAGCCATGCATGTCTAAGTATAAATTCTTGTACG
ATGAAACTGCAGACGGCTCATTACAACAGTGATAAACTAATAGACTTTCGGGTTTTTAACCTTTTGGATAACCGCAGT
AAATCGGGGCTAATACATAGAAGCGATGGGTGACTGGCAACGGAAGCTCAGCGATTATTAGCATTACTACCAATACC
TTCGGGTCTTGTGGTGAAACCGAATAATATTGCAGATCGAGGATTTATCTTCGACAAGTCTATTGTGTCACTGCCCTA
TCAACTTTCGATGGTACGGTATTGGCCTACCATGGTTGTAACGGGTAACGGGGAATTAGGGTTCGATTCCGGAGAGG
GAGCCTGAGAAATGGCTACCACTTCTACGGAAGGCAGCAGGCGCGCAAATTACTCAATCCCAATACGGGGAAGTAG
TGACAATAAATATCAATACCTATCCTTTTTGGAGGGCAATTGAAATGAACACAAATTAAAACTCTTAATTAACACAAT
TGGAGGGCAAGTCTGGTGCCAGCAGCCGCGGTAATTCCAGCTCCAATAGCATATACTAAAGTTGTTGCAGTTAAAAA
GCTCGTAGTTGAAGTTAAGGGTTTATCGGGCTAAAGTTATTTGCCACTTTGTGGTTAAATTCAACTCCGGTATTCCTTT
TCTTAATAGTTCAGCTTCTATTATCTTTGATAGTAGTTGTTTGGGCATTTCACTGTGAGAAAATTGTGGTGTTTAAAGC
AGGCGTCTCGCCTGATCTTTTGCAGCATGGTATGATGAAACATGACATTTTACGCTATTGGTTTGCGTCTAAAGTGTA
ATGATTAATAGGGATGGATGGGGGTGTTCATATTGGTGGGCGAGAGGTGAAATTCGTTGACCCTATCAAGATGAACTT
CTGCGAAAGCATTCACCAAATACTTCCCCATTAATCAAGAACGAAAGTTTGGGGATCGAAGACGATCAGATACCGTC
GTAGTCCAAACTATAAACTATGTCGACCAGGGATCGGTTAAAATTTTTTTAAAATTTAATCGGCACCTTGTGAGAAAT
CACGAGTGTTTAGATTCCGGGGGGAGTATGGTCGCAAGTCTGAAACTTAAAGGAATTGACGGAAGGGCACACAATG
GAGTGGAGCCTGCGGCTTAATTTGACTCAACTCGGGAAAACTTACCAAGCTAAGATATAGTAAGGATTGACAGACTA
AAAGATCTTTCATGATTCTATAAGTGGTGGTGCATGGTCGTTCTTAGTTGGTGAGCGATTTGTCTGGTCAATTCCGATA
ACGGACGAGACCTCGACCTGCTAACTAGTAGTATTTATTAGTCGATATGGGCGATAGCTTCTCTGGGGTTTGGAACGG
CTTCGGCCGTCTCCTGCTTCAAGGAGTGTGTAGTCTGACTTGATAGGTACGAATTCAAAAACTTCTTAGAGGGACTA
CCTGCCTCAAGCAGGCGGAAGTCCGAGGCAATAACAGGTCTGTGATGCCCTTAGATACCTTGGGCCGCACGCGCGC
TACAATGTATAAAACAAAAAGGTTCCTGGTCCGGAAGGATTGGGTAATCAATTGAATTTTCTACGTAACTGGGATTG
ATCTTTGTAATTATTGATCATCAACGAGGAATTCCTTGTAAGCGTAAGTCATTACCTTATGCTGAATATGTCCCTGCCC
TTTGTACACACCGCCCGTCGCTCCTACCGATCGAATGATACGGTAAAGTTAACGGATCGTTTTATTTGTGGCAACACA
AATTAAGACTAAAAGTTATTTAAATCTCATTGTTAGAGGAAGAGAAGTCGTAACAAGGTATCCGTAGGTGAACCTGC

AGAAGGATCA

>AM168042.1 *Dictyostelium giganteum* WS589
AACCTGGTTGATCCTGCCAGTAGTCATATGCTTGTCTCAAAGATTAAGCCATGCATGTCTAAGTATAAATTCTTGTACG
ATGAAACTGCAGACGGCTCATTACAACAGTGATAAACTAATAGACTTTCGGGTTTTTAACCTTTTGGATAACCGCAGT
AAATCGGGGCTAATACATAGAAGCGATGGGTGACTGGCAACGGAAGCTCAGCGATTATTAGCATTACTACCAATACC
TTCGGGTCTTGTGGTGAAACCGAATAATATTGCAGATCGAGGATTTATCTTCGACAAGTCTACTGTGTCACTGCCCTA
TCAACTTTCGATGGTACGGTATTGGCCTACCATGGTTGTAACGGGTAACGGGGAATTAGGGTTCGATTCCGGAGAGG
GAGCCTGAGAAATGGCTACCACTTCTACGGAAGGCAGCAGGCGCGCAAATTACTCAATCCCAATACGGGGAAGTAG
TGACAATAAATATCAATACCTATCCTTTTTGGAGGGCAATTGAAATGAACACAAATTAAAACTCTTAATTAACACAAT
TGGAGGGCAAGTCTGGTGCCAGCAGCCGCGGTAATTCCAGCTCCAATAGCATATACTAAAGTTGTTGCAGTTAAAAA
GCTCGTAGTTGAAGTTAAGGGTTTATCGGGCTAAAGTTATTTGCCACTTTGTGGTTAAATTCAACTCCGGTATTCCTTT
TCTTAATAGTTCAGCTTCCATTATCTTTGATAGTGGTTGTTTGGGCATTTCACTGTGAGAAAATTGTGGTGTTTAAAGC
AGGCGTCTCGCCTGATCTTTTGCAGCATGGTATGATGAAACATGACATTTTACGCTATTGGTTTGCGTCTAAAGTGTA
ATGATTAATAGGGATGGATGGGGGTGTTCATATTGGTGGGCGAGAGGTGAAATTCGTTGACCCTATCAAGATGAACTT
CTGCGAAAGCATTCACCAAATACTTCCCCATTAATCAAGAACGAAAGTTTGGGGATCGAAGACGATCAGATACCGTC
GTAGTCCAAACTATAAACTATGTCGACCAGGGATCGGTTAAAATTTTTTTAAAATTTAATCGGCACCTTGTGAGAAAT
CACGAGTGTTTAGATTCCGGGGGGAGTATGGTCGCAAGTCTGAAACTTAAAGGAATTGACGGAAGGGCACACAATG
GAGTGGAGCCTGCGGCTTAATTTGACTCAACTCGGGAAAACTTACCAAGCTAAGATATAGTAAGGATTGACAGACTA
AAAGATCTTTCATGATTCTATAAGTGGTGGTGCATGGTCGTTCTTAGTTGGTGGAGCGATTTGTCTGGTCAATTCCGA
TAACGGACGAGACCTCGACCTGCTAACTAGTAGTATTTATTAGTCGATATGGGCGATAGCTTCTCTGGGGTTTGGAAT
GATTTCGGTCATCTCCTGCTTCAAGGAGTGTGTAGTCTGACTTGATAGGTACGAATTCAAAAACTTCTTAGAGGGAC
TACCTGCCTCAAGCAGGCGGAAGTCCGAGGCAATAACAGGTCTGTGATGCCCTTAGATACCTTGGGCCGCACGCGC
GCTACAATGTAGAAAACAAAAAGGTTCCTGGTCCGGAAGGATTGGGTAATCAATTGAATTTTCTACGTAACTGGGAT
TGATCTTTGTAATTATTGATCATCAACGAGGAATTCCTTGTAAGCGCAAGTCATTACCTTGTGCTGAATATGTCCCTGC
CCTTTGTACACACCGCCCGTCGCTCCTACCGATCGAATGATACGGTAAAGTTAACGGATCGTTTTATTTGTGGCAACA
CATTATAAGACTAAAAGTTATTTAAATCTCATTGTTTAGAGGAAGGAGAAGTCGTAACAAGGTATCCGTAGGTGAAC
CTGCAGAAGGATCAA

>AM168043.1 *Dictyostelium implicatum* 93HO-1
AACCTGGTTGATCCTGCCAGTAGTCATATGCTTGTCTCAAAGATTAAGCCATGCATGTCTAAGTATAAATTCTTGTACG
ATGAAACTGCAGACGGCTCATTACAACAGTGATAAACTAATAGACTTTCGGGTTTTTAACCTTTTGGATAACCGCAGT
AAATCGGGGCTAATACATAGAAGCGATGGGTGACTGGCAACGGAATCTCAGCGATTATTAGCATTACTACCAATACCT
TCGGGTCTTGTGGTGAAACCGAATAATATTGCAGATCGAGGATTTATCTTCGACAAGTCTACTGTGTCACTGCCCTAT
CAACTTTCGATGGTACGGTATTGGCCTACCATGGTTGTAACGGGTAACGGGGAATTAGGGTTCGATTCCGGAGAGGG
AGCCTGAGAAATGGCTACCACTTCTACGGAAGGCAGCAGGCGCGCAAATTACTCAATCCCAATACGGGGAAGTAGT
GACAATAAATATTAATACCTATCCTTAATGGAGGGCAATTGAAATGAACACAAATTAAAACTCTTAATTAACACAATT
GGAGGGCAAGTCTGGTGCCAGCAGCCGCGGTAATTCCAGCTCCAATAGCATATACTAAAGTTGTTGCAGTTAAAAAG
CTCGTAGTTGAAGTCAAAGGTTTATTGGGTCAAAGTTATTTGCCGCTCTGGTGGTTAAATATACTCCAGTATCTCTTTT
TTTAATAGTTCAGCTTGTATTAACTTTGTTAGTACTTGTTTGGACATTTCACTGTGAGAAAATTGTGGTGTTTAAAGCA
GGCGTTTCGCCTGATCTTTTGCAGCATGGTATGATGAAACATGACATTTTACGCTATTGGTTTGCGTCTAAAGTGTAAT
GATTAATAGGGATGGATGGGGGTGTTCATATTGGTGGGCGAGAGGTGAAATTCGTTGACCCTATCAAGATGAACTTC
TGCGAAAGCATTCACCAAATACTTCCCCATTAATCAAGAACGAAAGTTTGGGGATCGAAGACGATCAGATACCGTCG
TAGTCCAAACTATAAACTATGTCGACCAGGGATCGGTTAAAATTTTTTTAAAATTTAATCGGCACCTTGTGAGAAATC
ACGAGTGTTTATATTCCGGGGGGAGTATGGTCGCAAGTCTGAAACTTAAAGGAATTGACGGAAGGGCACACAATGG
AGTGGAGCCTGCGGCTTAATTTGACTCAACTCGGGAAAACTTACCAAGCTAAGATATAGTAAGGATTGACAGACTAA
AAGATCTTTCATGATTCTATAAGTGGTGGTGCATGGTCGTTCTTAGTTGGTGGAGCGATTTGTCTGGTCAATTCCGATA
ACGGACGAGACCTCGACCTGCTAACTAGTAGTATTTATTAGTCAATATGGGCGATAGCTTTTCTGGGATTTAGAGCGA
TTTCGGTCGTTTCTTGTTTCAAGGAGTGTGTAGTCTGGCTTGATAGGTACGAATTAAAAAAACTTCTTAGAGGGACT
ACCTGCCTCAAGCAGGCGGAAGTCCGAGGCAATAACAGGTCTGTGATGCCCTTAGATACCTTGGGCCGCACGCGCG
CTACAATGTAGAAAACAAAAAGGTTCCTGGTCCGGAAGGATTGGGTAATCAATTGAATTTTCTACGTAACTGGGATT
GATCTTTGTAATTATTGATCATCAACGAGGAATTCCTTGTAAGCGTAGGTCATTACCCTATGCTGAATATGTCCCTGCC
CTTTGTACACACCGCCCGTCGCTCCTACCGATCGAATGATACGGTAAAGTTAACGGATCGTTTTATCTTTGGCAACAT
TGATATAAATTAAAAGTTATTTAAATCTCATTGTTTAGAGGAAGGAGAAGTCGTAACAAGGTATCCGTAGGTGAACCT
GCAGAAGGATCAA

>MK322958.1 *Dictyostelium insulinativitatis*
TGTCTAAGTATAAATTCTTGTACGATGAAACTGCAGACGGCTCATTACAACAGTGATAAACTAATAGACTTTCGGGTT
TTTAACCTTTTGGATAACCGCAGTAAATCGGGGCTAATACATACAAGCGATGGGTGACTGGCAACGGAAGCTCAGCG
ATTATTAGCATTACTACCAATACCTTCGGGTCTTGTGGTGAAACCGAATAATATTGCAGATCGAGGATTTATCTTCGAC
AAGTCTACTGTGTCACTGCCCTATCAACTTTCGATGGTACGGTATTGGCCTACCATGGTTGTAACGGGTAACGGGGA
ATTAGGGTTCGATTCCGGAGAGGGAGCCTGAGAAATGGCTACCACTTCTACGGAAGGCAGCAGGCGCGCAAATTAC
TCAATCCCAATACGGGGAAGTAGTGACAATAAATATCAATACCTATCCTTAACGGAGGGCAATTGAAATGAACACAA
ATTAAAACTCTTAATTAACACAATTGGAGGGCAAGTCTGGTGCCAGCAGCCGCGGTAATTCCAGCTCCAATAGCATA
TACTAAAGTTGTTGCAGTTAAAAAGCTCGTAGTTGAAGTTAAAGGTTTACTGGGCTAAAGTTATTTGCCACTCTGGT
GGTTAAATCAACTCCAGTATTCCTTTTTTTAATAGCTCTGCTTCTAGCATCTTTGATGTTAGTTGTTTGGGCATTTCACT
GTGAGAAAATTGTGGTGTTTAAAGCAGGCGTCTCGCCTGATCTTTTGCAGCATGGTATGATGAAACATGACATTTTAC
GCTATTGGTTTGCGTCTAAAGTGTAATGATTAATAGGGATGGATGGGGGTGTTCATATTGGTGGGCGAGAGGTGAAAT
TCGTTGACCCTATCAAGATGAACTTCTGCGAAAGCATTCACCAAATACTTCCCCATTAATCAAGAACGAAAGTTTGG
GGATCGAAGACGATCAGATACCGTCGTAGTCCAAACTATAAACTATGTCGACCAGGGATCGGTCAAAATTTTTTTAA
AATTTGATCGGCACCTTGTGAGAAATCATGAGTGTTTAGATTCCGGGGGGAGTATGGTCGCAAGTCTGAAACTTAAA
GGAATTGACGGAAGGGCACACAATGGAGTGGAGCCTGCGGCTTAATTTGACTCAACTCGGGAAAACTTACCAAGCT
AAGATATAGTAAGGATTGACAGACTAAAAGATCTTTCATGATTCTATAAGTGGTGGTGCATGGTCGTTCTTAGTTGGT
GGAGCGATTTGTCTGGTCAATTCCGATAACGGACGAGACCTCGACCTGCTAACTAGTAGTATTTATTAGCCGATATGG
GCGATAGCTTCTCTGGGGTTTGGAATGATTTCGGTCATCTCCTACTTCAAGGAGTGTGTAGTCTGGCTTGATAGGTAC

GAATTAAAAAACTTCTTAGAGGGACTACCTGCCTCAAGCAGGCGGAAGTCCGAGGCAATAACAGGTCTGTGATGCC
CTTAGATACCTTGGGCCGCACGCGCGCTACAATGTAGAAAACAAAAAGGCTCCTGGTCCGGAAGGATTGGGTAATC
ATTTGAATTTCTACGTAACTGGGATTGATCTTTGTAATTATTGATCATCAACGAGGAATTCCTTGTAAGCGCAGGTCA
TTACCCTGTGCTGAATATGTCCCTGCCCTTTGTACACACCGCCCGTCGCTCCTACCGATCGAATGATACGGTAAAGTT
AACGGATCGTTTTATCTGTGGCAACACTGATATAAACTAAAAGTTATTTAAATCTCATTGTTTAGAGGAAGGAGAAGT
CGTAACAAGGTATCCGTAGGTGAAC

>AM168044.1 *Dictyostelium intermedium* PJ11
AACCTGGTTGATCCTGCCAGTAGTCATATGCTTGTCTCAAAGATTAAGCCATGCATGTCTAAGTATAAATTCTTGTACG
ATGAAACTGCAGACGGCTCATTACAACAGTGATAAACTAATAGACTTTCGGGTTTTACCTTTTGGATAACCCCAGTAA
ATCGGGGCTAATACATACAAGCGATGGGTGACTGGTAACGGAAGCTCAGCGATTATTAGCATTCTACCAATACCTTCG
GGTTTTGGGTGAGACCGAATAATATTGCAGATCGAGGATTTATCTTCGACAAGTCTACTGTGTCACTGCCCTATCAAC
TTTCGATGGTACGGTATTGGCCTACCATGGTTGTAACGGGTAACGGGGAATTAGGGTTCGATTCCGGAGAGGGAGCC
TGAGAAATGGCTACCACTTCTACGGAAGGCAGCAGGCGCGCAAATTACTCAATCCCAATACGGGGAAGTAGCGACA
ATAAATATCAATACCTATCCTTTTTGGAGGGCAATTGAAATGAACACAAATTAAAACTCTTAATTAACACAATTGGAG
GGCAAGTCTGGTGCCAGCAGCCGCGGTAATTCCAGCTCCAATAGCATATACTAAAGTTGTTGCAGTTAAAAAGCTCG
TAGTTGAAGTTAAAGGTTTACCGGGTTTACGTCATTTGTCACTTCGTGGCTAAATCGACTCCGGTATCTCTTTCTTAAT
AGTTCAGCTTTCATTATCTTTGATAGTGGTTGTTTGGACATTTCACTGTGAGAAAATTGTGGTGTTTAAAGCAGGCGT
CTCGCCTGATCTTTTGCAGCATGGTATGATGAAACATGACATTTTACGTTATTGGTTTGCGTCTAAAGTGTAATGATTA
ATAGGGATGGATGGGGGTGTTCATATTGGTGGGCGAGAGGTGAAATTCGTTGACCCTATCAAGATGAACTTCTGCGA
AAGCATTCACCAAATACTTCCCCATTAATCAAGAACGAAAGTTTGGGGATCGAAGACGATCAGATACCGTCGTAGTC
CAAACTATAAACTATGTCGACCAGGGATCGGTTAAAATTTTTTTAAAATTTAATCGGCACCTTGTGAGAAATCACGAG
TGTTTAGATTCCGGGGGGAGTATGGTCGCAAGTCTGAAACTTAAAGGAATTGACGGAAGGGCACACAATGGAGTGG
AGCCTGCGGCTTAATTTGACTCAACTCGGGAAAACTTACCAAGCTAAGATATAGTAAGGATTGACAGACTAAAAGAT
CTTTCATGATTCTATAAGTGGTGGTGCATGGTCGTTCTTAGTTGGTGGAGCGATTTGTCTGGTCAATTCCGATAACGG
ACGAGACCTCGACCTGCTAACTAGTAGTATTTATTAGTCGATATGGACGATAGCTTTTCTGGGGTTTGGAATGATTTCG
GTCATCTCCTGCTTCAAGGAGTGTGTAGTCTGACTCGATAGGTACGAATTCAAAAACTTCTTAGAGGGACTACCTGC
CTCAAGCAGGCGGAAGTCCGAGGCAATAACAGGTCTGTGATGCCCTTAGATACCTTGGGCCGCACGCGCGCTACAA
TGTAGGAAACAAAAAGGCTCCTGGTCCGGAAGGATTGGGTAATCATTTGAATTTCCTACGTAACTGGGATTGATCTT
TGTAATTATTGATCATAAACGAGGAATTCCTTGTAAGCATAAGTCATTACCTTATGCTGAATATGTCCCTGCCCTTTGTA
CACACCGCCCGTCGCTCCTACCGATCGAATGATACGGTAAAGTTAACGGATCGTTTTATCTGTGGCAACACTGATATA
AATTAAAAGTTATTTAAATCTCATTGTTTAGAGGAAGGAGAAGTCGTAACAAGGTATCTGTAGGTGAACCTGCAGAA
GGATCAA

>HM159992.1 *Dictyostelium leptosomopsis* Araucaria 1
AACCTGGTTGATCCTGCCAGTAGTCATATGCTTGTCTCAAAGATTAAGCCATGCATGTCTAAGTATAAATTCTTGTACG
ATGAAACTGCAGACGGCTCATTACAACAGTGATAAACTGCTAGACTTTCGGGTTTTTAACCTTTTCGGATAACCGCA
GTAAATCGGGGCTAATACATAGAAGCGATGGGCGACTGGTAACGGAAGCTCAGCGATTATTAGCATAACTACCAATA
CCTTCGGGTCTTGTGGTGAAACCGAATAATATTGCAGATCGAAGATTTATCTTCGACAAGTCTAATGTGTCACTGCCC
TATCAACTTTCGATGGTACGGTATTGGCCTACCATGGTTGTAACGGGTAACGGGGAATTAGGGTTCGATTCCGGAGA
GGGAGCCTGAGAAATGGCTACCACTTCTACGGAAGGCAGCAGGCGCGCAAATTACTCAATCCCAATACGGGGAAGT
AGTGACAATAAATATCAATACCTATCCTTAATGGAGGGCAATTGAAATGAACACAAATTAAAACTCTTAATTAACACA
ATTGGAGGGCAAGTCTGGTGCCAGCAGCCGCGGTAATTCCAGCTCCAATAGCATATACTAAAGTTGTTGCAGTTAAA
AAGCTCGTAGTTGAAGTTAAAGGTTTATTGGGTCAAAGCTATTTGCCGCTCTGGTGGTTAAATATGCTCCAGTATTTC
TTTTTTTTGAAAAATTGTTCAGCTTGTATCAACTTTGTTGGTACTCGTTTGGACATTTCACTGTGAGAAAATTGTGGT
GTTTAAAGCAGGCGTCTCGCCTGATCTTTTGCAGCATGGTATGATGAAACATGACATTTTGCGCTATTGGTTTGCGTC
TAAAGTGTAATGATTAATAGGGATGGATGGGGGTGTTCATATTGGTGGGCGAGAGGTGAAATTCGTTGACCCTATCAA
GATGAACTTCTGCGAAAGCATTCACCAAATACTTCTCCATTAATCAAGAACGAAAGTTTGGGGATCGAAGACGATCA
GATACCGTCGTAGTCCAAACTATAAACTATGTCGACCAGGGATCGGTTAAAATTTTTTTAAAATTTAATCGGCACCTT
GTGAGAAATCACGAGTGTTTAGATTCCGGGGGGAGTATGGTCGCAAGTCTGAAACTTAAAGGAATTGACGGAAGGG
CACACAATGGAGTGGAGCCTGCGGCTTAATTTGACTCAACTCGGGAAAACTTACCAAGCTAAGATATAGTTAGGATT
GACAGACTAAAAGATCTTTCATGATTCTATAAGTGGTGGTGCATGGTCGTTCTTAGTTGGTGGAGCGATTTGTCTGGT
CAATTCCGATAACGGACGAGACCTCGACCTGCTAACTAGTAGTATTTATTAGTCAATATGGGCGATAGCTTTTCTGGG
ATTTAGAGCGATTTCGGTCGTTTCTGGTTTCAAGGAGTGTGTAGTCTGACTTGATAGGTACGAATTAAAAAAACTTCT
TAGAGGGACTACCTGCCTCAAGCAGGCGGAAGTCCGAGGCAATAACAGGTCTGTGATGCCCTTAGATACCTTGGGC
CGCACGCGCGCTACAATGTATAAAACAAAAAGGTTCCTGGTCCGGAAGGATTGGGTAATCAATTGAATTTCTACGT
AACTGGGATTGATCTTTGTAATTATTGATCATAAACGAGGAATTCCTTGTAAGCGTAGGTCATTACCCTATGCTGAATA
TGTCCCTGCCCTTTGTACACACCGCCCGTCGCTCCTACCGATCGAATGATACGGTAAAGTTAACGGATAGTTTTATCT
TTGGCAACATTGATATAAATTAAAAGTTATTTAAATCTCATTGTTAGAGGAAGAGAAGTCGA

>HQ141480.1 *Dictyostelium leptosomum* NZN49A
AACCTGGTTGATCCTGCCAGTAGTCATATGCTTGTCTCAAAGATTAAGCCATGCATGTCTAAGTATAAATTCTTGTACG
ATGAAACTGCAGACGGCTCATTACAACAGTGATAAACTGCTAGACTTTCGGGTTTTTAACCTTTTGGATAACCGCAG
TAAATCGGGGCTAATACATAGAAGCGATGGGTGACTGGTAACGGAAGCTCAGCGATTATTAGCATAACTACCAATAC
CTTCGGGTCTTGTGGTGAAACCGAATAATATTGCAGATCGAGGATTTATCTTCGACAAGTCTAATGTGTCACTGCCCT
ATCAACTTTCGATGGTACGGTATTGGCCTACCATGGTTGTAACGGGTAACGGGGAATTAGGGTTCGATTCCGGAGAG
GGAGCCTGAGAAATGGCTACCACTTCTACGGAAGGCAGCAGGCGCGCAAATTACTCAATCCCAATACGGGGAAGTA
GTGACAATAAATATCAATACCTATCCTTAATGGAGGGCAATTGAAATGAACACAAATTAAAACTCTTAATTAACACAA
TTGGAGGGCAAGTCTGGTGCCAGCAGCCGCGGTAATTCCAGCTCCAATAGCATATACTAAAGTTGTTGCAGTTAAAA
AGCTCGTAGTTGAAGTTAAAGGTTTATTGGGTCAAAGCTATTTGCCGCTCTGGTGGTTAAATATGCTCCAGTATTTCT
TTTTTTTGAAAAATTGTTCAGCTTGTATCAACTTTGTTGGTACTCGTTTGGACATTTCACTGTGAGAAAATTGTGGTG
TTTAAAGCAGGCGTCTCGCCTGATCTTTTGCAGCATGGTATGATGAAACATGACATTTTGCGCTATTGGTTTGCGTCT
AAAGTGTAATGATTAATAGGGATGGATGGGGGTGTTCATATTGGTGGGCGAGAGGTGAAATTCGTTGACCCTATCAA
GATGAACTTCTGCGAAAGCATTCACCAAATACTTCTCCATTAATCAAGAACGAAAGTTTGGGGATCGAAGACGATCA
GATACCGTCGTAGTCCAAACTATAAACTATGTCGACCAGGGATCGGTTAAAATTTTTTTAAAATTTAATCGGCACCTT

GTGAGAAATCACGAGTGTTTAGATTCCGGGGGGAGTATGGTCGCAAGTCTGAAACTTAAAGGAATTGACGGAAGGG
CACACAATGGAGTGGAGCCTGCGGCTTAATTTGACTCAACTCGGGAAAACTTACCAAGCTAAGATATAGTTAGGATT
GACAGACTAAAAGATCTTTCATGATTCTATAAGTGGTGGTGCATGGTCGTTCTTAGTTGGTGGAGCGATTTGTCTGGT
CAATTCCGATAACGGACGAGACCTCGACCTGCTAACTAGTAGTATTTATTAGTCAATATGGGCGATAGCTTTTCTGGG
ATTTAGAGCGATTTCGGTCGTTTCTGGTTTCAAGGAGTGTGTAGTCTGGCTTGATAGGTACGAATTAAAAAAACTTCT
TAGAGGGACTACCTGCCTCAAGCAGGCGGAAGTCCGAGGCAATAACAGGTCTGTGATGCCCTTAGATACCTTGGGC
CGCACGCGCGCTACAATGTATAAAACAAAAAGGTTCCTGGTCCGGAAGGATTGGGTAATCAATTGAATTTTCTACGT
AACTGGGATTGATCTTTGTAATTATTGATCATAAACGAGGAATTCCTTGTAAGCGTAGGTCATTACCCTATGCTGAATA
TGTCCCTGCCCTTTGTACACACCGCCCGTCGCTCCTACCGATCGAATGATACGGTAAAGTTAACGGATAGTTTTATCT
TTGGCAACATTGATATAAATTAAAAGTTATTTAAATCTCATGTTAGAGAAGAGAAAGTCGTAACAAGGTATCCGTAGG
TGAACCTGCAGAAGGATCA

>AM168048.1 *Dictyostelium longosporum* TNS-C-109
AACCTGGTTGATCCTGCCAGTAGTCATATGCTTGTCTCAAAGATTAAGCCATGCATGTCTAAGTATAAATTCTTGTACG
ATGAAACTGCAGACGGCTCATTACAACAGTGATAAACTAATAGACTTTCGGGTTTTTAACCTTTTGGATAACCGCAGT
AAATCGGGGCTAATACATAGAAGCGATGGGTGACTGGCAACGGAAGCTCAGCGATTATTAGCATTACTACCAATACC
TTCGGGTCTTGTGGTGAAACCGAATAATATTGCAGATCGAGGATTTATCTTCGACAAGTCTACTGTGTCACTGCCCTA
TCAACTTTCGATGGTACGGTATTGGCCTACCATGGTTGTAACGGGTAACGGGGAATTAGGGTTCGATTCCGGAGAGG
GAGCCTGAGAAATGGCTACCACTTCTACGGAAGGCAGCAGGCGCGCAAATTACTCAATCCCAATACGGGGAAGTAG
TGACAATAAATATCAATACCTATCCTTAACGGAGGGCAATTGAAATGAACACAAATTAAAACTCTTAATTAACACAAT
TGGAGGGCAAGTCTGGTGCCAGCAGCCGCGGTAATTCCAGCTCCAATAGCATATACTAAAGTTGTTGCAGTTAAAAA
GCTCGTAGTTGAAGTTAAAGGTTTACTGGGCTAAAGTTATTTGCCGCTCTGGTGGTTAAATCAACTCCAGTATCTCTT
TTTTTAATAGTTCAGCTTCTATTATCTTTGATAGTAGTTGTTTGGACATTTCACTGTGAGAAAATTGTGGTGTTTAAAG
CAGGCGTTTCGCCTGATCTTTTGCAGCATGGTATGATGGAACATGACATTTTACGCTATTGGTTTGCGTCTAAAGTGT
AATGATTAATAGGGATGGATGGGGGTGTTCATATTGGTGGGCGAGAGGTGAAATTCGTTGACCCTATCAAGATGAAC
TTCTGCGAAAGCATTCACCAAATACTTCCCCATTCATCAAGAACGAAAGTTTGGGGATCGAAGACGATCAGATACCG
TCGTAGTCCAAACTATAAACTATGTCGACCAGGGATCGGTTAAAATTTTTTTACAATTTAATCGGCACCTTGTGAGAA
ATCACGAGTGTTTAGATTCCGGGGGGAGTATGGTCGCAAGTCTGAAATTAAAGGAATTGACGGAAGGGCACACAAT
GGAGTGGAGCCTGCGGCTTAATTTGACTCAACTCGGGAAAACTTACCAAGCTAAGATATAGTAAGGATTGACAGACT
AAAAGATCTTTCATGATTCTATAAGTGGTGGTGCATGGTCGTTCTTAGTTGGTGGAGCGATTTGTCTGGTCAATTCCG
ATAACGGACGAGACCTCGACCTGCTAACTAGTAGTATTTATTAGTCAATATGGGCGATAGCTTCTCTGGGGTTTGGAA
TGGCTTCGGTCATCTCCTGCTTCAAGGAGTGTGTAGTCTGACTTGATAGGTACGAATTAAAAAACTTCTTAGAGGGA
CTACCTGCCTCAAGCAGGCGGAAGTCCGAGGCAATAACAGGTCTGTGATGCCCTTAGATACCTTGGGCCGCACGCG
CGCTACAATGTAGAAAACAAAAAGGTTCCTGGTCCGGAAGGATTGGGTAATCAATTGAATTTTCTACGTAACTGGGA
TTGATCTTTGTAATTATTGATCATCAACGAGGAATTCCTTGTAAGCGTAGGTCATTACCCTATGCTGAATATGTCCCTG
CCCTTTGTACACACCGCCCGTCGCTCCTACCGATCGAATGATACGGTAAAGTTAACGGATCGTTTTATCTGTGGCAAC
ACTGATATAAACTAAAAGTTATTTAAATCTCATTGTTTAGAGGAAGGAGAAGTCGTAACAAGGTATCCGTAGGTGAA
CCTGCAGAAGGATCAA

>AM168049.1 *Dictyostelium macrocephalum* B33
AACCTGGTTGATCCTGCCAGTAGTCATATGCTTGTCTCAAAGATTAAGCCATGCATGTCTAAGTATAAATTCTTGTACG
ATGAAACTGCAGACGGCTCATTACAACAGTGATAAACTAATAGACTTTCGGGTTTTTAACCTTTTGGATAACCGCAGT
AAATCGGGGCTAATACATACAAGCGATGGGTGACTGGCAACGGAAGCTCAGCGATTATTAGCATTACTACCAATACC
TTCGGGTCTTGTGGTGAAACCGAATAATATTGCAGATCGAGGATTTATCTTCGACAAGTCTACTGTGTCACTGCCCTA
TCAACTTTCGATGGTACGGTATTGGCCTACCATGGTTGTAACGGGTAACGGGGAATTAGGGTTCGATTCCGGAGAGG
GAGCCTGAGAAATGGCTACCACTTCTACGGAAGGCAGCAGGCGCGCAAATTACTCAATCCCAATACGGGGAAGTAG
TGACAATAAATATCAATACCTATCCTTAACGGAGGGCAATTGAAATGAACACAAATTAAAACTCTTAATTAACACAAT
TGGAGGGCAAGTCTGGTGCCAGCAGCCGCGGTAATTCCAGCTCCAATAGCATATACTAAAGTTGTTGCAGTTAAAAA
GCTCGTAGTTGAAGTTAAAGGTTTACTGGGCTAAAGTTATTTGCCACTCTGGTGGTTAAATCAACTCCAGTATTCCTT
TTTTTAATAGCTCTGCTTCTAGCATCTTTGATGTTAGTTGTTTGGGCATTTCACTGTGAGAAAATTGTGGTGTTTAAAG
CAGGCGTCTCGCCTGATCTTTTGCAGCATGGTATGATGAAACATGACATTTTACGCTATTGGTTTGCGTCTAAAGTGT
AATGATTAATAGGGATGGATGGGGGTGTTCATATTGGTGGGCGAGAGGTGAAATTCGTTGACCCTATCAAGATGAAC
TTCTGCGAAAGCATTCACCAAATACTTCCCCATTAATCAAGAACGAAAGTTTGGGGATCGAAGACGATCAGATACCG
TCGTAGTCCAAACTATAAACTATGTCGACCAGGGATCGGTCAAAATTTTTTTAAAATTTGATCGGCACCTTGTGAGAA
ATCATGAGTGTTTAGATTCCGGGGGGAGTATGGTCGCAAGTCTGAAACTTAAAGGAATTGACGGAAGGGCACACAA
TGGAGTGGAGCCTGCGGCTTAATTTGACTCAACTCGGGAAAACTTACCAAGCTAAGATATAGTAAGGATTGACAGAC
TAAAAGATCTTTCATGATTCTATAAGTGGTGGTGCATGGTCGTTCTTAGTTGGTGGAGCGATTTGTCTGGTCAATTCC
GATAACGGACGAGACCTCGACCTGCTAACTAGTAGTATTTATTAGCCGATATGGGCGATAGCTTCTCTGGGGTTTGGA
ATGATTTCGGTCATCTCCTATTTCAAGGAGTGTGTAGTCTGGCTTGATAGGTACGAATTAAAAAACTTCTTAGAGGGA
CTACCTGCCTCAAGCAGGCGGAAGTCCGAGGCAATAACAGGTCTGTGATGCCCTTAGATACCTTGGGCCGCACGCG
CGCTACAATGTAGAAAACAAAAAGGCTCCTGGTCCGGAAGGATTGGGTAATCATTTGAATTTTCTACGTAACTGGGA
TTGATCTTTGTAATTATTGATCATCAACGAGGAATTCCTTGTAAGCGCAGGTCATTACCCTGTGCTGAATATGTCCCTG
CCCTTTGTACACACCGCCCGTCGCTCCTACCGATCGAATGATACGGTAAAGTTAACAGATCGTTTTATCTGTGGCAAC
ACTGATATAAACTAAAAGTTATTTAAATCTCATTGTTTAGAGGAAGGAGAAGTCGTAACAAGGTATCCGTAGGTGA
ACCTGCAGAAGGATCAA

>AM168050.1 *Dictyostelium medium* TNS-C-205
AACCTGGTTGATCCTGCCAGTAGTCATATGCTTGTCTCAAAGATTAAGCCATGCATGTCTAAGTATAAATTCTTGTACG
ATGAAACTGCAGACGGCTCATTACAACAGTGATAAACTAATAGACTTTCGGGTTTTTAACCTTTTGGATAACCGCAGT
AAATCGGGGCTAATACATAGAAGCGATGGGTGACGGCAACGAAGCTCAGCGATTATTAGCATTACTACCAATACCTT
CGGGTCTTGTGGTGAAACCGAATAATATTGCAGATCGAGGATTTATCTTCGACAAGTCTACTGTGTCACTGCCCTATC
AACTTTCGATGGTACGGTATTGGCCTACCATGGTTGTAACGGGTAACGGGGAATTAGGGTTCGATTCCGGAGAGGGA
GCCTGAGAAATGGCTACCACTTCTACGGAAGGCAGCAGGCGCGCAAATTACTCAATCCCAATACGGGGAAGTAGTG
ACAATAAATATCAATACCTATCCTTAATGGAGGGCAATTGAAATGAACACAAATTAAAACTCTTAATTAACACAATTG
GAGGGCAAGTCTGGTGCCAGCAGCCGCGGTAATTCCAGCTCCAATAGCATATACTAAAGTTGTTGCAGTTAAAAAGC

TCGTAGTTGAAGTTAAAGGTTTACTGGGCTAAAGTTATTTGCCGCTCTGGTGGTTAAATCAACTCCAGTATCTCTTTC
GCTCAATAGTTCAGCTTGTATCAACCTAGTTGGTACTCGTTTGGACATTTCACTGTGAGAAAATTGTGGTGTTTAAAG
CAGGCGTCTCGCCTGATCTTTTGCAGCATGGTATGATGGAACATGACATTTTGCGCTATTGGTTTGCGTCTAAAGTGT
AATGATTAATAGGGATGGATGGGGGTGTTCATATTGGTGGGCGAGAGGTGAAATTCGTTGACCCTATCAAGATGAAC
TTCTGCGAAAGCATTCACCAAATACTTCCCCATTAATCAAGAACGAAAGTTTGGGGATCGAAGACGATCAGATACCG
TCGTAGTCCAAACTATAAACTATGTCGACCAGGGATCGGTTAAAATTTTTTTAAAATTTAATCGGCACCTTGTGAGAA
ATCACGAGTGTTTAGATTCCGGGGGGAGTATGGTCGCAAGTCTGAAACTTAAAGGAATTGACGGAAGGGCACACAA
TGGAGTGGAGCCTGCGGCTTAATTTGACTCAACTCGGGAAAACTTACCAAGCTAAGATATAGTAAGGATTGACAGAC
TAAAAGATCTTTCATGATTCTATAAGTGGTGGTGCATGGTCGTTCTTAGTTGGTGGAGCGATTTGTCTGGTCAATTCC
GATAACGGACGAGACCTCGACCTGCTAACTAGTAGTATTTATTAGTCGATATAGGCGATAGCTTTTCTGGGGTTTGGA
ATGATTTCGGTCATCTCCTATTTCAAGGAGTGTGTAGTCTGGCTTGATAGGTACGAATTAAAAAACTTCTTAGAGGGA
CTACCTGCCTCAAGCAGGCGGAAGTCCGAGGCAATAACAGGTCTGTGATGCCCTTAGATACCTTGGGCCGCACGCG
CGCTACAATGTAGAAAACAAAAAGGCTCCTGGTCCGGAAGGATTGGGTAATCATTTGAATTTCTACGTAACTGGGA
TTGATCTTTGTAATTATTGATCATCAACGAGGAATTCCTTGTAAGCGCAGGTCATTACCCTGTGCTGAATATGTCCCTG
CCCTTTGTACACACCGCCCGTCGCTCCTACCGATCGAATGATACGGTAAAGTTAACGGATCGTTTTATCTGTGGCAAC
ACTGATATAAACTAAAAGTTATTTAAATCTCATTGTTTAGAGGAAGGAGAAGTCGTAACAAGGTATCCGTAGGTGAA
CCTGCAGAAGGATCAA

>MG490369.1 *Dictyostelium minimum*
GCGGATCTGTCTCAGATAAGCCATGCATGTCTAAGTATAAATTCTTGTACGATGAAACTGCAGACGGCTCATTACAAC
AGTGATAAACTAATAGACTTTCGGGTTTTTAACCTTTTGGATAACCGCAGTAAATCGGGGCTAATACATAGAAGCGAT
GGGTGACTGGCAACGGAAGCTCAGCGATTATTAGCATTACTACCAATACCTTCGGGTCTTGTGGTGAAACCGAATAA
TATTGCAGATCGAGGATTTATCTTCGACAAGTCTACTGTGTCACTGCCCTATCAACTTTCGATGGTACGGTATTGGCCT
ACCATGGTTGTAACGGGTAACGGGGAATTAGGGTTCGATTCCGGAGAGGGAGCCTGAGAAATGGCTACCACTTCTA
CGGAAGGCAGCAGGCGCGCAAATTACTCAATCCCAATACGGGGAAGTAGTGACAATAAATATCAATACCTATCCTTA
ACGGAGGGCAATTGAAATGAACACAAATTAAAACTCTTAATTAACACAATTGGAGGGCAAGTCTGGTGCCAGCAG
CCGCGGTAATTCCAGCTCCAATAGCATATACTAAAGTTGTTGCAGTTAAAAAGCTCGTAGTTGAAGTTAAAGGTTTAC
TGGGCTAAAGTTATTTGCCGCTCTGGTGGTTAAATCAACTCCAGTATCTCTTTTTTTAATAGTTCAGCTTCTATTATCTT
TGATAGTAGTTGTTTGGACATTTCACTGTGAGAAAATTGTGGTGTTTAAAGCAGGCGTTTCGCCTGATCTTTTGCAGC
ATGGTATGATGAAACATGACATTTTACGCTATTGGTTTGCGTCTAAAGTGTAATGATTAATAGGGATGGATGGGGGTG
TTCATATTGGTGGGCGAGAGGTGAAATTCGTTGACCCTATCAAGATGAACTTCTGCGAAAGCATTCACCAAATACTT
CCCCATTAATCAAGAACGAAAGTTTGGGGATCGAAGACGATCAGATACCGTCGTAGTCCAAACTATAAACTATGTCG
ACCAGGGATCGGTTAAAATTTTTTTAAAATTTAATCGGCACCTTGTGAGAAATCACGAGTGTTTAGATTCCGGGGGG
AGTATGGTCGCAAGTCTGAAACTTAAAGGAATTGACGGAAGGGCACACAATGGAGTGGAGCCTGCGGCTTAATTTG
ACTCAACTCGGGAAAACTTACCAAGCTAAGATATAGTAAGGATTGACAGACTAAAAGATCTTTCATGATTCTATAAGT
GGTGGTGCATGGTCGTTCTTAGTTGGTGGAGCGATTTGTCTGGTCAATTCCGATAACGGACGAGACCTCGACCTGCT
AACTAGTAGTATTTATTAGTCAATATGGGCGATAGCTTCTCTGGGGTTTGGAATGGTTTCGGCCATCTCCTGCTTCAAG
GAGTGTGTAGTCTGACTTGATAGGTACGAATTAAAAAACTTCTTAGAGGGACTACCTGCCTCAAGCAGGCGGAAGT
CCGAGGCAATAACAGGTCTGTGATGCCCTTAGATACCTTGGGCCGCACGCGCGCTACAATGTAGAAAACAAAAAGG
TTCCTGGTCCGGAAGGATTGGGTAATCATTTGAATTTCTACGTAACTGGGATTGATCTTTGTAATTATTGATCATCAA
CGAGGAATTCCTTGTAAGCGTAGGTCATTACCCTATGCTGAATATGTCCCTGCCCTTTGTACACACCGCCCGTCGCTC
CTACCGATCGAATGATACGGTAAAGTTAACGGATCGTTTATCTTTGGCAACATTGATATAAACTAAAAGTTATTTAAA
TCTCATTGTTTAGAGGAAGGAGAAGTCGTAACAAGGTATCCGTAGGTGAACCTGCAGAAAGGATCAAA

>AM168054.1 *Dictyostelium brefeldianum* S28b
AACCTGGTTGATCCTGCCAGTAGTCATATGCTTGTCTCAAAGATTAAGCCATGCATGTCTAAGTATAAATTCTTGTACG
ATGAAACTGCAGACGGCTCATTACAACAGTGATAAACTGCTAGACTTTCGGGTTTTTAACCTTTTGGATAACCGCAG
TAAATCGGGGCTAATACATAGAAGCGATGGGCGACTGGTAACGGAAGCTCAGCGATTATTAGCATAACTACCAATAC
CTTCGGGTCTTGTGGTGAAACCGAATAATATTGCAGATCGAAGATTTATCTTCGACAAGTCTAATGTGTCACTGCCCT
ATCAACTTTCGATGGTACGGTATTGGCCTACCATGGTTGTAACGGGTAACGGGGAATTAGGGTTCGATTCCGGAGAG
GGAGCCTGAGAAATGGCTACCACTTCTACGGAAGGCAGCAGGCGCGCAAATTACTCAATCCCAATACGGGGAAGTA
GTGACAATAAATATCAATACCTATCCTTAATGGAGGGCAATTGAAATGAACACAAATTAAAACTCTTAATTAACACAA
TTGGAGGGCAAGTCTGGTGCCAGCAGCCGCGGTAATTCCAGCTCCAATAGCATATACTAAAGTTGTTGCAGTTAAAA
AGCTCGTAGTTGAAGTTAAAGGTTTATTGGGTCAAAGCTATTTGCCGCTCTGGTGGTTAAATATGCTCCAGTATTTCT
TTTTTTTGAAAAATTGTTCAGCTTGTATCAACTTTGTTGGTACTCGTTTGGACATTTCACTGTGAGAAAATTGTGGTG
TTTAAAGCAGGCGTCTCGCCTGATCTTTTGCAGCATGGTATGATGAAACATGACATTTTGCGCTATTGGTTTGCGTCT
AAAGTGTATTGATTAATAGGGATGGATGGGGGTGTTCATATTGGTGGGCGAGAGGTGAAATTCGTTGACCCTATCAA
GATGAACTTCTGCGAAAGCATTCACCAAATACTTCTCCATTAATCAAGAACGAAAGTTTGGGGATCGAAGACGATCA
GATACCGTCGTAGTCCAAACTATAAACTATGTCGACCAGGGATCGGTTAAAATTTTTTTAAAATTTAATCGGCACCTT
GTGAGAAATCACGAGTGTTTAGATTCCGGGGGGAGTATGGTCGCAAGTCTGAAACTTAAAGGAATTGACGGAAGGG
CACACAATGGAGTGGAGCCTGCGGCTTAATTTGACTCAACTCGGGAAAACTTACCAAGCTAAGATATAGTTAGGATT
GACAGACTAAAAGATCTTTCATGATTCTATAAGTGGTGGTGCATGGTCGTTCTTAGTTGGTGGAGCGATTTGTCTGGT
CAATTCCGATAACGGACGAGACCTCGACCTGCTAACTAGTAGTATTTATTAGTCAATATGGGCGATAGCTTTTCTGGG
ATTTAGAGCGATTTCGGTCGTTTCTGGTTTCAAGGAGTGTGTAGTCTGACTTGATAGGTACGAATTAAAAAAACTTCT
TAGAGGGACTACCTGCCTCAAGCAGGCGGAAGTCCGAGGCAATAACAGGTCTGTGATGCCCTTAGATACCTTGGGC
CGCACGCGCGCTACAATGTATAAAACAAAAAGGTTCCTGGTCCGGAAGGATTGGGTAATCAATTGAATTTCTACGT
AACTGGGATTGATCTTTGTAATTATTGATCATAAACGAGGAATTCCTTGTAAGCGTAGGTCATTACCCTATGCTGAATA
TGTCCCTGCCCTTTGTACACACCGCCCGTCGCTCCTACCGATCGAATGATACGGTAAAGTTAACGGATAGTTTTATCT
TTGGCAACATTGATATAAATTAAAAGTTATTTAAATCTCATTGTTTAGAGGAAGGAGAAGTCGTAACAAGGTATCCGT
AGGTGAACCTGCAGAAGGATCAA

>AM168055.1 *Dictyostelium mucoroides* var. *stoloniferum* FOII-1
AACCTGGTTGATCCTGCCAGTAGTCATATGCTTGTCTCAAAGATTAAGCCATGCATGTCTAAGTATAAATTCTTGTACG
ATGAAACTGCAGACGGCTCATTACAACAGTGATAAACTAATAGACTTTCGGGTTTTTAACCTTTTGGATAACCGCAGT
AAATCGGGGCTAATACATAGAAGCGATGGGTGACGGCAACGAAGCTCAGCGATTATTAGCATTACTACCAATACCTT

CGGGTCTTGTGGTGAAACCGAATAATATTGCAGATCGAGGATTTATCTTCGACAAGTCTACTGTGTCACTGCCCTATC
AACTTTCGATGGTACGGTATTGGCCTACCATGGTTGTAACGGGTAACGGGGAATTAGGGTTCGATTCCGGAGAGGGA
GCCTGAGAAATGGCTACCACTTCTACGGAAGGCAGCAGGCGCGCAAATTACTCAATCCCAATACGGGGAAGTAGTG
ACAATAAATATCAATACCTATCCTTAATGGAGGGCAATTGAAATGAACACAAATTAAAACTCTTAATTAACACAATTG
GAGGGCAAGTCTGGTGCCAGCAGCCGCGGTAATTCCAGCTCCAATAGCATATACTAAAGTTGTTGCAGTTAAAAAGC
TCGTAGTTGAAGTTAAAGGTTTACTGGGCTAAAGTTATTTGCCGCTCTGGTGGTTAAATCAACTCCAGTATCTCTTTC
GCTCAATAGTTCAGCTTGTATCAACCTAGTTGGTACTCGTTTGGACATTTCACTGTGAGAAAATTGTGGTGTTTAAAG
CAGGCGTCTCGCCTGATCTTTTGCAGCATGGTATGATGGAACATGACATTTTGCGCTATTGGTTTGCGTCTAAAGTGT
AATGATTAATAGGGATGGATGGGGGTGTTCATATTGGTGGGCGAGAGGTGAAATTCGTTGACCCTATCAAGATGAAC
TTCTGCGAAAGCATTCACCAAATACTTCCCCATTAATCAAGAACGAAAGTTTGGGGATCGAAGACGATCAGATACCG
TCGTAGTCCAAACTATAAACTATGTCGACCAGGGATCGGTTAAAATTTTTTTAAAATTTAATCGGCACCTTGTGAGAA
ATCACGAGTGTTTAGATTCCGGGGGGAGTATGGTCGCAAGTCTGAAACTTAAAGGAATTGACGGAAGGGCACACAA
TGGAGTGGAGCCTGCGGCTTAATTTGACTCAACTCGGGAAAACTTACCAAGCTAAGATATAGTAAGGATTGACAGAC
TAAAAGATCTTTCATGATTCTATAAGTGGTGGTGCATGGTCGTTCTTAGTTGGTGGAGCGATTTGTCTGGTCAATTCC
GATAACGGACGAGACCTCGACCTGCTAACTAGTAGTATTTATTAGTCGATATGGGCGATAGCTTTTCTGGGGTGGAGA
ACGATTTCGGTCGCTTCTTACTTCAAGGAGTGTGTAGTCTGACTTGATAGGTACGAATTAAAAAAACTTCTTAGAGG
GACTACCTGCCTCAAGCAGGCGGAAGTCCGAGGCAATAACAGGTCTGTGATGCCCTTAGATACCTTGGGCCGCACG
CGCGCTACAATGTAGAAAACAAAAAGGCTCCTGGTCCGGAAGGATTGGGTAATCAATTGAATTTCTACGTAACTGG
GATTGATCTTTGTAATTATTGATCATCAACGAGGAATTCCTTGTAAGCGTAAGTCATTACCTTATGCTGAATATGTCCCT
GCCCTTTGTACACACCGCCCGTCGCTCCTACCGATCGAATGATACGGTAAAGTTAACGGATCGTTTTACCTTTGGCAA
CATTGGTATAAACTAAAAGTTATTTAAATCTCATTGTTTAGAGGAAGGAGAAGTCGTAACAAGGTATCCGTAGGTGA
ACCTGCAGAAGGATCAA

>MG490370.1 *Dictyostelium multiforme* 4007
TGTCTAAGTATAAATTCTTGTACGATGAAACTGCAGACGGCTCATTACAACAGTGATAAACTAATAGACTTTCGGGTT
TTTAACCTTTTGGATAACCGCAGTAAATCGGGGCTAATACATAGAAGCGATGGGTGACTGGCAACGGAAGCTCAGCG
ATTATTAGCATTACTACCAATACCTTCGGGTCTTGTGGTGAAACCGAATAATATTGCAGATCGAGGATTTATCTTCGAC
AAGTCTACTGTGTCACTGCCCTATCAACTTTCGATGGTACGGTATTGGCCTACCATGGTTGTAACGGGTAACGGGGA
ATTAGGGTTCGATTCCGGAGAGGGAGCCTGAGAAATGGCTACCACTTCTACGGAAGGCAGCAGGCGCGCAAATTAC
TCAATCCCAATACGGGGAAGTAGTGACAATAAATATCAATACCTATCCTTAACGGAGGGCAATTGAAATGAACACAA
ATTAAAACTCTTAATTAACACAATTGGAGGGCAAGTCTGGTGCCAGCAGCCGCGGTAATTCCAGCTCCAATAGCATA
TACTAAAGTTGTTGCAGTTAAAAAGCTCGTAGTTGAAGTTAAAGGTTTACTGGGCTAAAGTTATTTGCCGCTCTGGT
GGTTAAATCAACTCCAGTATCTCTTTTTTTAATAGTTCAGCTTCTATTATCTTTGATAGTAGTTGTTTGGACATTTCACT
GTGAGAAAATTGTGGTGTTTAAAGCAGGCGTTTCGCCTGATCTTTTGCAGCATGGTATGATGAAACATGACATTTTAC
GCTATTGGTTTGCGTCTAAAGTGTAATGATTAATAGGGATGGATGGGGGTGTTCATATTGGTGGGCGAGAGGTGAAAT
TCGTTGACCCTATCAAGATGAACTTCTGCGAAAGCATTCACCAAATACTTCCCCATTAATCAAGAACGAAAGTTTGG
GGATCGAAGACGATCAGATACCGTCGTAGTCCAAACTATAAACTATGTCGACCAGGGATCGGTTAAAATTTTTTTAAA
ATTTAATCGGCACCTTGTGAGAAATCACGAGTGTTTAGATTCCGGGGGGAGTATGGTCGCAAGTCTGAAACTTAAAG
GAATTGACGGAAGGGCACACAATGGAGTGGAGCCTGCGGCTTAATTTGACTCAACTCGGGAAAACTTACCAAGCTA
AGATATAGTAAGGATTGACAGACTAAAAGATCTTTCATGATTCTATAAGTGGTGGTGCATGGTCGTTCTTAGTTGGTG
GAGCGATTTGTCTGGTCAATTCCGATAACGGACGAGACCTCGACCTGCTAACTAGTAGTATTTATTAGTCAATATGGG
CGATAGCTTCTCTGGGGTTTGGAATGGTTTCGGCCATCTCCTGCTTCAAGGAGTGTGTAGTCTGACTTGATAGGTACG
AATTAAAAAAACTTCTTAGAGGGACTACCTGCCTCAAGCAGGCGGAAGTCCGAGGCAATAACAGGTCTGTGATGCCC
TTAGATACCTTGGGCCGCACGCGCGCTACAATGTAGAAAACAAAAAGGTTCCTGGTCCGGAAGGATTGGGTAATCAT
TTGAATTTTCTACGTAACTGGGATTGATCTTTGTAATTATTGATCATCAACGAGGAATTCCTTGTAAGCGTAGGTCATT
ACCCTATGCTGAATATGTCCCTGCCCTTTGTACACACCGCCCGTCGCTCCTACCGATCGAATGATACGGTAAAGTTAA
CGGATCGTTTTATCTTTGGCAACATTGATATAAACTAAAAGTTATTTAAATCTCATTGTTTAGAGGAAGGAGAAGTCG
TAACAAGGTATCCGTAGGTGAACCTGCAGAAGGATCAA

>AM168059.1 *Dictyostelium pseudobrefeldianum* 91HO-8
AACCTGGTTGATCCTGCCAGTAGTCATATGCTTGTCTCAAAGATTAAGCCATGCATGTCTAAGTATAAATTCTTGTACG
ATGAAACTGCAGACGGCTCATTACAACAGTGATAAACTGCTAGACTTTCGGGTTTTTAACCTTTTGGATAACCGCAG
TAAATCGGGGCTAATACATAGAAGCGATGGGCGACTGGTAACGGAAGCTCAGCGATTATTAGCATAACTACCAATAC
CTTCGGGTCTTGTGGTGAAACCGAATAATATTGCAGATCGAAGATTTATCTTCGACAAGTCTAATGTGTCACTGCCCT
ATCAACTTTCGATGGTACGGTATTGGCCTACCATGGTTGTAACGGGTAACGGGGAATTAGGGTTCGATTCCGGAGAG
GGAGCCTGAGAAATGGCTACCACTTCTACGGAAGGCAGCAGGCGCGCAAATTACTCAATCCCAATACGGGGAAGTA
GTGACAATAAATATCAATACCTATCCTTAATGGAGGGCAATTGAAATGAACACAAATTAAAACTCTTAATTAACACAA
TTGGAGGGCAAGTCTGGTGCCAGCAGCCGCGGTAATTCCAGCTCCAATAGCATATACTAAAGTTGTTGCAGTTAAAA
AGCTCGTAGTTGAAGTTAAAGGTTTATTGGGTCAAAGCTATTTGCCGCTCTGGTGGTTAAATATGCTCCAGTATTTCT
TTTTTTTGAAAAATTGTTCAGCTTGTATCAACTTTGTTGGTACTCGTTTGGACATTTCACTGTGAGAAAATTGTGGTG
TTTAAAGCAGGCGTCTCGCCTGATCTTTTGCAGCATGGTATGATGAAACATGACATTTTGCGCTATTGGTTTGCGTCT
AAAGTGTAATGATTAATAGGGATGGATGGGGGTGTTCATATTGGTGGGCGAGAGGTGAAATTCGTTGACCCTATCAA
GATGAACTTCTGCGAAAGCATTCACCAAATACTTCTCCATTAATCAAGAACGAAAGTTTGGGGATCGAAGACGATCA
GATACCGTCGTAGTCCAAACTATAAACTATGTCGACCAGGGATCGGTTAAAATTTTTTTAAAATTTAATCGGCACCTT
GTGAGAAATCACGAGTGTTTAGATTCCGGGGGGAGTATGGTCGCAAGTCTGAAACTTAAAGGAATTGACGGAAGGG
CACACAATGGAGTGGAGCCTGCGGCTTAATTTGACTCAACTCGGGAAAACTTACCAAGCTAAGATATAGTTAGGATT
GACAGACTAAAAGATCTTTCATGATTCTATAAGTGGTGGTGCATGGTCGTTCTTAGTTGGTGGAGCGATTTGTCTGGT
CAATTCCGATAACGGACGAGACCTCGACCTGCTAACTAGTAGTATTTATTAGTCAATATGGGCGATAGCTTTTCTGGG
ATTTAGAGCGACTTCGGTTGTTTCTGGTTTCAAGGAGTGTGTAGTCTGACTTGATAGGTACGAATTAAAAAAACTTC
TTAGAGGGACTACCTGCCTCAAGCAGGCGGAAGTCCGAGGCAATAACAGGTCTGTGATGCCCTTAGATACCTTGGG
CCGCACGCGCGCTACAATGTATAAAACAAAAAGGTTCCTGGTCCGGAAGGATTGGGTAATCAATTGAATTTTCTACG
TAACTGGGATTGATCTTTGTAATTATTGATCATAAACGAGGAATTCCTTGTAAGCGTAGGTCATTACCCTATGCTGAAT
ATGTCCCTGCCCTTTGTACACACCGCCCGTCGCTCCTACCGATCGAATGATACGGTAAAGTTAACGGATAGTTTTATC
TTTGGCAACATTGATATAAATTAAAAGTTATTTAAATCTCATTGTTTAGAGGAAGGAGAAGTCGTAACAAGGTATCCG
TAGGTGAACCTGCAGAAGGATCAA

>FJ424829.1 *Dictyostelium purpureum* QSpu1
cATATGCTTGTCTCAAAGATTAAGCCATGCATGTCTAAGTATAAATTCTTGTACGATGAAACTGCAGACGGCTCATTAC
AACAGTGATAAACTGCTAGACTTTCGGGTTTTAACCTTTTGGATAACCGCAGTAAATCGGGGCTAATACATACAAGC
GATGGGTGACTGGCAACGGAAGCTCAGCGATTATTAGCGTTACTACCAATACCTTCGGGTCTTGTGGTGAAACCGAA
TAATATTGCAGATCGGGGATTTATCTTCGACAAGTCTACTGTGTCACTGCCCTATCAACTTTCGATGGTACGGTATTGG
CCTACCATGGTTGTAACGGGTAACGGGGAATTAGGGTTCGATTCCGGAGAGGGAGCCTGAGAAATGGCTACCACTT
CTACGGAAGGCAGCAGGCGCGCAAATTACTCAATCCCAATACGGGGAAGTAGTGACAATAAATATCAATACCTATCC
TTTTTGGAGGGCAATTGAAATGAACACAAATTAAAACTCTTAATTAACACAATTGGAGGGCAAGTCTGGTGCCAGC
AGCCGCGGTAATTCCAGCTCCAATAGCATATACTAAAGTTGTTGCAGTTAAAAAGCTCGTAGTTGAAGTTAAAGTTAT
ATTGGGCTCAAGTTTTTTACCACGTCTGTGGCTAAAATAACTCCAATATTTCTTTTTTTAATAGCTCAGTTTCTAGGTC
TTTGACTCTAGTTATTTGGGCATTTCACTGTGAGAAAATTGTGGTGTTTAAAGCAGGCGTCTCGTCTGATCTTTTGCA
GCATGGTATGATGGAACATGACATTTTACGCTATTGGTTTGCGTCTAAAGTGTAATGATTAATAGGGATGGATGGGGAT
GTTCATATTGGTGGGCGAGAGGTGAAATTCGTTGACCCTATCAAGATGAACTTCTGCGAAAGCATTCATCAAATACTT
CCCCATTAATCAAGAACGAAAGTTTGGGGATCGAAGACGATCAGATACCGTCGTAGTCCAAACTATAAACTATGTCG
ACCAGGGATCGGCTAAAATTTTTTTAAAATTTAGTCGGCACCTTGTGAGAAATCATGAGTGTTTAGATTCCGGGGGG
AGTATGGTCGCAAGTCTGAAACTTAAAGGAATTGACGGAAGGGCACACAATGGAGTGGAGCCTGCGGCTTAATTTG
ACTCAACTCGGGAAAACTTACCAAGCTAAGATATAGTAAGGATTGACAGACTAAAAGATCTTTCATGATTCTATAAGT
GGTGGTGCATGGTCGTCTTAGTTGGNNNNNNNNNNNNNNNNNNNNNNNTCCGATAACGGACGAGACCTCGACCTGC
TAACTAGTAGTATTTATTAGCCGATATGGGCGATAGCTTCTCTGGGGTTTGAGTGCGGTAACGTATCTTGGCTTCAAG
GAGTGTGTAGTCTGGCTTGATAGGTACGATATTAAAAAACTTCTTAGAGGGACTACCTGCCTCAAGCAGGCGGAAGT
CCGAGGCAATAACAGGTCTGTGATGCCCTTAGATACCTTGGGCCGCACGCGCGCTACAATGCAGATAGCAAAAAGG
TTCCTGGCCTGGAAAGGTTGGGTAATCAATTGAATTTCTGCGTAACTGGGATTGATCTTTGTAATTATTGATCATCAA
CGAGGAATTCCTTGTAAGCGTAAGTCATTACCTTATGCTGAATATGTCCCTGCCCTTTGTACACACCGCCCGTCGCTC
CTACCGATCGAATGATACGGTAAAGTTAACGGATTGTTTTTTTGTGGCAACACAATTAAAATTAAAAGTTATTTAAAT
CTCATTGTTTAGAGGAAGGAGAAGTCGTAACAAGGTATCCGTAGGTGAACCTGCGGATGGATCATTTTTT

>FJ424839.1 *Dictyostelium purpureum* QSpu2
aCAACAGTGATAAACTGCTAGACTTTCGGGTTTTAACCTTTTGGATAACCGCAGTAAATCGGGGCTAATACATAGAA
GCGATGGGTGACTGGCAACGGAAGCTCAGCGATTATTAGCGTTACTACCAATACCTTCGGGTCTTGTGGTGAAACCG
AATAATATTGCAGATCGAGGATTTATCTTCGACAAGTCTACTGTGTCACTGCCCTATCAACTTTCGATGGTACGGTATT
GGCCTACCATGGTTGTAACGGGTAACGGGGAATTAGGGTTCGATTCCGGAGAGGGAGCCTGAGAAATGGCTACCAC
TTCTACGGAAGGCAGCAGGCGCGCAAATTACTCAATCCCAATACGGGGAAGTAGTGACAATAAATATCAATACCTAT
CCTTTTTGGAGGGCAATTGAAATGAACACAAATTAAAACTCTTAATTAACACAATTGGAGGGCAAGTCTGGTGCCAG
CAGCCGCGGTAATTCCAGCTCCAATAGCATATACTAAAGTTGTTGCAGTTAAAAAGCTCGTAGTTGAAGTTAAAGTT
ATATTGGGCTCAAGTTTTTTACCACTCTGGTGGCTAAAACAACTCCAATATTTCTTTTTTTAATAGCTCAGTTTCTAGG
TCTTTGACTCTAGTTATTTGGGCATTTCACTGTGAGAAAATTGTGGTGTTTAAAGCAGGCGTCTCGTCTGATCTTTTG
CAGCATGGTATGATGAAACATGACATTTTACGCTATTGGTTTGCGTCTAAAGTGTAATGATTAATAGGGATGGATGGG
GATGTTCATATTGGTGGGCGAGAGGTGAAATTCGTTGACCCTATCAAGATGAACTTCTGCGAAAGCATTCATCAAAT
ACTTCCCCATTAATCAAGAACGAAAGTTTGGGGATCGAAGACGATCAGATACCGTCGTAGTCCAAACTATAAACTAT
GTCGACCAGGGATCGGCTAAAATTTTTTTAAAATTTAGTCGGCACCTTGTGAGAAATCATGAGTGTTTAGATTCCGGG
GGGAGTATGGTCGCAAGTCTGAAACTTAAAGGAATTGACGGAAGGGCACACAATGGAGTGGAGCCTGCGGCTTAAT
TTGACTCAACTCGGGAAAACTTACCAAGCTAAGATATAGTAAGGATTGACAGACTAAAAGATCTTTCATGATTCTATA
AGTGGTGGTGCATGGTCGTTCTTAGTTGGTGGAGCGATTTGTCTGGTCAATTCCGATAACGGACGAGACCTCGACCT
GCTAACTAGTAGTATTTATTAGTCGATATGGGCGATAGCTTTTCTGGGGTTAGGATGCGAGCAATCGTATTCCTGCTTC
AAGGAGTGTGTAGTCTGACTTGATAGGTACGCTTTCAAAAAACTTCTTAGAGGGACTACCTGCCTCAAGCAGGCGG
AAGTCCGAGGCAATAACAGGTCTGTGATGCCCTTAGATACCTTGGGCCGCACGCGCGCTACAATGCAGATAGCAAA
AAGGTTCCTGGCCCGGAAGGGTTGGGTAATCAATTGAATTTCTGCGTAACTGGGATTGATCTTTGTAATTATTGATC
ATCAACGAGGAATTCCTTGTAAGCGTAAGTCATTACCTTATGCTGAATATGTCCCTGCCCTTTGTACACACCGCCCGT
CGCTCCTACCGATCGAATGATACGGTAAAGTTAACGGATAGTTTTTTTGTGGCAACACAACTAAAATTAAAAGTTATT
TAAATCTCATTGTTTAGAGGAAGGAGAAGTCGTAACAAGGTATCCGTAGGTGAACCTGCGGATGGATCATTTTTT

>FJ424832.1 *Dictyostelium purpureum* QSpu23
tTCTTGTACGATGAAACTGCAGACGGCTCATTACAACAGTGATAAACTGCTAGACTTTCGGGTTTTAACCTTTTGGAT
AACCGCAGTAAATCGGGGCTAATACATAGAAGCGATGGGTGACTGGCAACGGAAGCTCAGCGATTATTAGCGTTACT
ACCAATACCTTCGGGTCTTGTGGTGAAACCGAATAATATTGCAGATCGGGGATTTATCTTCGACAAGTCTACTGTGTC
ACTGCCCTATCAACTTTCGATGGTACGGTATTGGCCTACCATGGTTGTAACGGGTAACGGGGAATTAGGGTTCGATTC
CGGAGAGGGAGCCTGAGAAATGGCTACCACTTCTACGGAAGGCAGCAGGCGCGCAAATTACTCAATCCCAATACGG
GGAAGTAGTGACAATAAATATCAATACCTATCCTTTTTGGAGGGCAATTGAAATGAACACAAATTAAAACTCTTAATT
AACACAATTGGAGGGCAAGTCTGGTGCCAGCAGCCGCGGTAATTCCAGCTCCAATAGCATATACTAAAGTTGTTGCA
GTTAAAAAGCTCGTAGTTGAAGTTAAAGTTATATTGGGCTCAAGTTTTTTACCACGTCTGTGGCTAAAATAACTCCAA
TATTTCTTTTTTTAATAGCTCAGTTTCTAGGTCTTTGACTCTAGTTATTTGGGCATTTCACTGTGAGAAAATTGTGGTG
TTTAAAGCAGGCGTCTCGTCTGATCTTTTGCAGCATGGTATGATGGAACATGACATTTTACGCTATTGGTTTGCGTCTA
AAGTGTAATGATTAATAGGGATGGATGGGGATGTTCATATTGGTGGGCGAGAGGTGAAATTCGTTGACCCTATCAAG
ATGAACTTCTGCGAAAGCATTCATCAAATACTTCCCCATTAATCAAGAACGAAAGTTTGGGGATCGAAGACGATCAG
ATACCGTCGTAGTCCAAACTATAAACTATGTCGACCAGGGATCGGCTAAAATTTTTTTAAAATTTAGTCGGCACCTTG
TGAGAAATCATGAGTGTTTAGATTCCGGGGGGAGTATGGTCGCAAGTCTGAAACTTAAAGGAATTGACGGAAGGGC
ACACAATGGAGTGGAGCCTGCGGCTTAATTTGACTCAACTCGGGAAAACTTACCAAGCTAAGATATAGTAAGGATTG
ACAGACTAAAAGATCTTTCATGATTCTATAAGTGGTGGTGCATGGTCGTCNNNNNNNNNNNNNNNNNNNNNNNNN
NNNNTTCCGATAACGGACGAGACCTCGACCTGCTAACTAGTAGTATTTATTAGCCGATATGGGCGATAGCTTCTCTGG
GGTTTGAGTGCGGCAACGTATCTTGGCTTCAAGGAGTGTGTAGTCTGGCTTGATAGGTACGATATTAAAAAACTTCTT
AGAGGGACTACCTGCCTCAAGCAGGCGGAAGTCCGAGGCAATAACAGGTCTGTGATGCCCTTAGATACCTTGGGCC
GCACGCGCGCTACAATGCAGATAGCAAAAAGGTTCCTGGCCTGGAAAGGTTGGGTAATCAATTGAATTTCTGCGTA
ACTGGGATTGATCTTTGTAATTATTGATCATCAACGAGGAATTCCTTGTAAGCGTAAGTCATTACCTTATGCTGAATAT
GTCCCTGCCCTTTGTACACACCGCCCGTCGCTCCTACCGATCGAATGATACGGTAAAGTTAACGGATTGTTTTTTTGT

GGCAACACAATTAAAATTAAAAGTTATTTAAATCTCATTGTTTAGAGGAAGGAGAAGTCGTAACAAGGTATCCGTAG
GTGAACCTGCGGATGGATCATTTTAT

>FJ424836.1 *Dictyostelium purpureum* QSpu28
tATAAATTCTTGTACGATGAAACTGCAGACGGCTCATTACAACAGATGATAAACTGCTAGACTTTCGGGTTTTAACCT
TTTGGATAACCGCAGTAAATCGGGGCTAATACATAGAAGCGATGGGTGACTGGCAACGGAAGCTCAGCGATTATTAG
CGTTACTACCAATACCTTCGGGTCTTGTGGTGAAACCGAATAATATTGCAGATCGGGGATTTATCTTCGACAAGTCTA
CTGTGTCACTGCCCTATCAACTTTCGATGGTACGGTATTGGCCTACCATGGTTGTAACGGGTAACGGGGAATTAGGGT
TCGATTCCGGAGAGGGAGCCTGAGAAATGGCTACCACTTCTACGGAAGGCAGCAGGCGCGCAAATTACTCAATCCC
AATACGGGGAAGTAGTGACAATAAATATCAATACCTATCCTTTTTGGAGGGCAATTGAAATGAACACAAATTAAAAC
TCTTAATTAACACAATTGGAGGGCAAGTCTGGTGCCAGCAGCCGCGGTAATTCCAGCTCCAATAGCATATACTAAAG
TTGTTGCAGTTAAAAAGCTCGTAGTTGAAGTTAAAGTTATATTGGGCTCAAGTTTTTTACCACGTCTGTGGCTAAAAT
AACTCCAATATTTCTTTTTTAATAGCTCAGTTTCTAGGTCTTTGACTCTAGTTATTTGGGCATTTCACTGTGAGAAAA
TTGTGGTGTTTAAAGCAGGCGTCTCGTCTGATCTTTTGCAGCATGGTATGATGGAACATGACATTTTACGCTATTGGT
TTGCGTCTAAAGTGTAATGATTAATAGGGATGGATGGGGATGTTCATATTGGTGGGCGAGAGGTGAAATTCGTTGACC
CTATCAAGATGAACTTCTGCGAAAGCATTCATCAAATACTTCCCCATTAATCAAGAACGAAAGTTTGGGGATCGAAG
ACGATCAGATACCGTCGTAGTCCAAACTATAAACTATGTCGACCAGGGATCGGCTAAAATTTTTTTAAAATTTAGTCG
GCACCTTGTGAGAAATCATGAGTGTTTAGATTCCGGGGGGAGTATGGTCGCAAGTCTGAAACTTAAAGGAATTGAC
GGAAGGGCACACAATGGAGTGGAGCCTGCGGCTTAATTTGACTCAACTCGGGAAAACTTACCAAGCTAAGATATAG
TAAGGATTGACAGACTAAAAGATCTTTCATGATTCTATAAGTGGTGGTGCATGGTCGTTCTTAGTTGGTGGAGCGATT
TGTCTGGTCAATTCCGATAACGGACGAGACCTCGACCTGCTAACTAGTAGTATTTATTAGCCGATATGGGCGATAGCT
TCTCTGGGGTTTGAGTGCGGCAACGTATCTTGGCTTCAAGGAGTGTGTAGTCTGGCTTGATAGGTACGATATTAAAA
AACTTCTTAGAGGGACTACCTGCCTCAAGCAGGCGGAAGTCCGAGGCAATAACAGGTCTGTGATGCCCTTAGATAC
CTTGGGCCGCACGCGCGCTACAATGCAGATAGCAAAAAGGTTCCTGGCCTGGAAAGGTTGGGTAATCAATTGAATT
TTCTGCGTAACTGGGATTGATCTTTGTAATTATTGATCATCAACGAGGAATTCCTTGTAAGCGTAAGTCATTACCTTAT
GCTGAATATGTCCCTGCCCTTTGTACACACCGCCCGTCGCTCCTACCGATCGAATGATACGGTAAAGTTAACGGATTG
TTTTTTTGTGGCAACACAATTAAAATTAAAAGTTATTTAAATCTCATTGTTTAGAGGAAGGAGAAGTCGTAACAAGGT
ATCCGTAGGTGAACCTGCGGATGGATCATTTTAT

>DQ340386.1 *Dictyostelium purpureum*
cGTACTGTTACAGGTGCTTTATAATTCATTAACTGGTTGATCCTGCCAGTAGTCATATGCTTGTCTCAAAGATTAAGCC
ATGCATGTCTAAGTATAAATTCTTGTACGATGAAACTGCAGACGGCTCATTACAACAGTGATAAACTGCTAGACTTTC
GGGTTTTTAACCTTTTGGATAACCGCAGTAAATCGGGGCTAATACATAGAAGCGATGGGTGACTGGCAACGGAAGCT
CAGCGATTATTAGCATTACTACCAATACCTTCGGGTCTTGTGGTGAAACCGAATAATATTGCAGATCGGGGATTTATCT
TCGACAAGTCTACTGTGTCACTGCCCTATCAACTTTCGATGGTACGGTATTGGCCTACCATGGTTGTAACGGGTAACG
GGGAATTAGGGTTCGATTCCGGAGAGGGAGCCTGAGAAATGGCTACCACTTCTACGGAAGGCAGCAGGCGCGCAA
ATTACTCAATCCCAATACGGGGAAGTAGTGACAATAAATATCAATACCTATCCTTTTTGGAGGGCAATTGAAATGAAC
ACAAATTAAAACTCTTAATTAACACAATTGGAGGGCAAGTCTGGTGCCAGCAGCCGCGGTAATTCCAGCTCCAATAG
CATATACTAAAGTTGTTGCAGTTAAAAAGCTCGTAGTTGAAGTTAAAGTTTTATTGGGCTTAAGTTTTTTACCACTCT
GTGGCTAAAATAACTCCAATAATTCTTTTTTTAATAGCTCAGTTTCTAGGTCTTTGACTCTAGTTATTTGGGCATTTCAC
TGTGAGAAAATTGTGGTGTTTAAAGCAGGCGTCTCGTCTGATCTTTTGCAGCATGGTATGATGGAACATGACATTTTA
CGCTATTGGTTTGCGTCTAAAGTGTAATGATTAATAGGGATGGATGGGGATGTTCATATTGGTGGGCGAGAGGTGAAA
TTCGTTGACCCTATCAAGATGAACTTCTGCGAAAGCATTCATCAAATACTTCCCCATTAATCAAGAACGAAAGTTTGG
GGATCGAAGACGATCAGATACCGTCGTAGTCCAAACTATAAACTATGTCGACCAGGGATCGGCTAAAATTTTTTTAA
AATTTAGTCGGCACCTTGTGAGAAATCATGAGTGTTTAGATTCCGGGGGGAGTATGGTCGCAAGTCTGAAACTTAAA
GGAATTGACGGAAGGGCACACAATGGAGTGGAGCCTGCGGCTTAATTTGACTCAACTCGGGAAAACTTACCAAGCT
AAGATATAGTAAGGATTGACAGACTAAAAGATCTTTCATGATTCTATAAGTGGTGGTGCATGGTCGTTCTTAGTTGGT
GGAGCGATTTGTCTGGTCAATTCCGATAACGGACGAGACCTCGACCTGCTAACTAGTAGTATTTATTAGTCGATATGG
GCGATAGCTTCTCTGGGGTTGGAGTGCGGCAACGTATCTCTGCTTCAAGGAGTGTGTAGTCTGACTTGATAGGTACG
TTATTTAAAAACTTCTTAGAGGGACTACCTGCCTCAAGCAGGCGGAAGTCCGAGGCAATAACAGGTCTGTGATGCCC
TTAGATACCTTGGGCCGCACGCGCGCTACAATGCAGATAGCAAAAAGGTTCCTGGCCTGGAAAGGTTGGGTAATCA
ATTGAATTTTCTGCGTAACTGGGATTGATCTTTGTAATTATTGATCATCAACGAGGAATTCCTTGTAAGCGTAAGTCAT
TACCTTATGCTGAATATGTCCCTGCCCTTTGTACACACCGCCCGTCGCTCCTACCGATCGAATGATACGGTAAAGTTA
ACGGATAGTTTTTTTGTGGCAACACAATTAAAATTAAAAGTTATTTAAATCTCATTGTTTAGAGGAAGGAGAAGTCGT
AACAAGGTATCCGTAGGTGAACCTGCGGATGGATCATTTTTT

>FJ424826.1 *Dictyostelium purpureum* QSpu4
cTTGTACGATGAAACTGCAGACGGCTCATTACAACAGTGATAAACTGCTAGACTTTCGGGTTTTTAACCTTTTGGATA
ACCGCAGTAAATCGGGGCTAATACATAGAAGCGATGGGTGACTGGCAACGGAAGCTCAGCGATTATTAGCATTACTA
CCAATACCTTCGGGTCTTGTGGTGAAACCGAATAATATTGCAGATCGAGGATTTATCTTCGACAAGTCTACTGTGTCA
CTGCCCTATCAACTTTCGATGGTACGGTATTGGCCTACCATGGTTGTAACGGGTAACGGGGAATTAGGGTTCGATTCC
GGAGAGGGAGCCTGAGAAATGGCTACCACTTCTACGGAAGGCAGCAGGCGCGCAAATTACTCAATCCCAATACGGG
GAAGTAGTGACAATAAATATCAATACCTATCCTTTTTGGAGGGCAATTGAAATGAACACAAATTAAAACTCTTAATTA
ACACAATTGGAGGGCAAGTCTGGTGCCAGCAGCCGCGGTAATTCCAGCTCCAATAGCATATACTAAAGTTGTTGCAG
TTAAAAAGCTCGTAGTTGAAGTTAAAGTTTTATTGGGCTTAAGTTTTTTACCACCTAGTGGCTAAAATAACTCCAATA
ATTCTTTTTTTAATAGCTCAGTTTCTAGGTCTTTGACTCTAGTTATTTGGGCATTTCACTGTGAGAAAATTGTGGTGTT
TAAAGCAGGCGTCTCGTCTGATCTTTTGCAGCATGGTATGATGGAACATGACATTTTACGCTATTGGTTTGCGTCTAA
AGTGTAATGATTAATAGGGATGGATGGGGATGTTCATATTGGTGGGCGAGAGGTGAAATTCGTTGACCCTATCAAGAT
GAACTTCTGCGAAAGCATTCATCAAATACTTCCCCATTAATCAAGAACGAAAGTTTGGGGATCGAAGACGATCAGAT
ACCGTCGTAGTCCAAACTATAAACTATGTCGACCAGGGATCGGCTAAAATTTTTTTAAAATTTAGTCGGCACCTTGTG
AGAAATCATGAGTGTTTAGATTCCGGGGGGAGTATGGTCGCAAGTCTGAAACTTAAAGGAATTGACGGAAGGGCAC
ACAATGGAGTGGAGCCTGCGGCTTAATTTGACTCAACTCGGGAAAACTTACCAAGCTAAGATATAGTAAGGATTGAC
AGACTAAAAGATCTTTCATGATTCTATAAGTGGTGGTGCATGGTCGTTCTTAGTTGGTGGAGCGATTTGTCTGGTCAA
TTCCGATAACGGACGAGACCTCGACCTGCTAACTAGTAGTATTTATTAGTCGATATGGGCGATAGCTTCTCTGGGGTT
GGAGTGCGGCAACGTATCTCTGCTTCAAGGAGTGTGTAGTCTGACTTGATAGGTACGTTATTAAAAAACTTCTTAGA

GGGACTACCTGCCTCAAGCAGGCGGAAGTCCGAGGCAATAACAGGTCTGTGATGCCCTTAGATACCTTGGGCCGCA
CGCGCGCTACAATGCAGATAGCAAAAAGGTTCCTGGCCTGGAAAGGTTGGGTAATCAATTGAATTTCTGCGTAACT
GGGATTGATCTTTGTAATTATTGATCATCAACGAGGAATTCCTTGTAAGCGTAAGTCATTACCTTATGCTGAATATGTC
CCTGCCCTTTGTACACACCGCCCGTCGCTCCTACCGATCGAATGATACGGTAAAGTTAACGGATAGTTTTTTTGTGGC
AACACAATTAAAATTAAAAGTTATTTAAATCTCATTGTTTAGAGGAAGGAGAAGTCGTAACAAGGTATCCGTAGGTG
AACCTGCGGATGGATCATTTTTT

>AY040335.1 *Dictyostelium purpureum*
TCATATGCTTGTCTCAAAGATTAAGCCATGCATGTCTAAGTATAAATTCTTGTACGATGAAACTGCAGACGGCTCATTA
CAACAGTGATAAACTGCTAGACTTTCGGGTTTTTAACCTTTTGGATAACCGCAGTAAATCGGGGCTAATACATAGAA
GCGATGGGTGACTGGCAACGGAAGCTCAGCGATTATTAGCATTACTACCAATACCTTCGGGTCTTGTGGTGAAACCG
AATAATATTGCAGATCGAGGATTTATCTTCGACAAGTCTACTGTGTCACTGCCCTATCAACTTTCGATGGTACGGTATT
GGCCTACCATGGTTGTAACGGGTAACGGGGAATTAGGGTTCGATTCCGGAGAGGGAGCCTGAGAAATGGCTACCAC
TTCTACGGAAGGCAGCAGGCGCGCAAATTACTCAATCCCAATACGGGGAAGTAGTGACAATAAATATCAATACCTAT
CCTTAACGGAGGGCAATTGAAATGAACACAAATTAAAACTCTTAATTAACACAATTGGAGGGCAAGTCTGGTGCCA
GCAGCCGCGGTAATTCCAGCTCCAATAGCATATACTAAAGTTGTTGCAGTTAAAAAGCTCGTAGTTGAAGTTAAAGT
TTTATTGGGCTAACGTTTTTTACCACCCTGTGGCTAAAATAACTCCAATATTTCTTTTTTTAATAGCTCAGTTTCTAGGT
CTTTGACTCTAGTTATTTGGGCATTTCACTGTGAGAAAATTGTGGTGTTTAAAGCAGGCGTCTCGTCTGATCTTTTGC
AGCATGGTATGATGGAACATGACATTTTACGCTATTGGTTTGCGTCTAAAGTGTAATGATTAATAGGGATGGATGGGG
ATGTTCATATTGGTGGGCGAGAGGTGAAATTCGTTGACCCTATCAAGATGAACTTCTGCGAAAGCATTCATCAAATAC
TTCCCCATTAATCAAGAACGAAAGTTTGGGGATCGAAGACGATCAGATACCGTCGTAGTCCAAACTATAAACTATGT
CGACCAGGGATCGGCTAATATTTTTTTAAAATTTAGTCGGCACCTTGTGAGAAATCATGAGTGTTTAGATTCCGGGGG
GAGTATGGTCGCAAGTCTGAAACTTAAAGGAATTGACGGAAGGGCACACAATGGAGTGGAGCCTGCGGCTTAATTT
GACTCAACTCGGGAAAACTTACCAAGCTAAGATATAGTAAGGATTGACAGACTAAAAGATCTTTCATGATTCTATAA
GTGGTGGTGCATGGTCGTTCTTAGTTGGTGGAGCGATTTGTCTGGTCAATTCCGATAACGGACGAGACCTCGACCTG
CTAACTAGTAGTATTTATTAGTCGATATAGGCGATAGCTTCTCTGGGGTTGGAGTGCGGCAACGTATCTCTGCTTCAA
GGAGTGTGTAGTCTGACTTGATAGGTACGTTAACAAAAAACTTCTTAGAGGGACTACCTGCCTCAAGCAGGCGGAA
GTCCGAGGCAATAACAGGTCTGTGATGCCCTTAGATACCTTGGGCCGCACGCGCGCTACAATGCAGATAGCAAAAA
GGTTCCTGGTCTGGAAAGATTGGGTAATCAATTGAATTTTCTGCGTAACTGGGATTGATCTTTGTAATTATTGATCATC
AACGAGGAATTCCTTGTAAGCGTAAGTCATTACCTTATGCTGAATATGTCCCTGCCCTTTGTACACACCGCCCGTCGC
TCCTACCGATCGAATGATACGGTAAAGTTAACGGATAGTTTTTTTGTGGCAACACAATTAAAATTAAAAGTTATTTAA
ATCTCATTGTTTAGAGGAaGGAGAAGTCGTAACAAGGTATCCGTAGTGAACCTGCGGATGGATCATTTT

>FJ424828.1 *Dictyostelium purpureum* QSpu36
AAGCCATGCATGTCTAAGTATAAATTCTTGTACGATGAAACTGCAGACGGCTCATTACAACAGTGATAAACTGCTAG
ACTTTCGGGTTTTTAACCTTTTGGATAACCGCAGTAAATCGGGGCTAATACATAGAAGCGATGGGTGACTGGCAACG
GAAGCTCAGCGATTATTAGCATTACTACCAATACCTTCGGGTCTTGTGGTGAAACCGAATAATATTGCAGATCGAGGA
TTTATCTTCGACAAGTCTACTGTGTCACTGCCCTATCAACTTTCGATGGTACGGTATTGGCCTACCATGGTTGTAACGG
GTAACGGGGAATTAGGGTTCGATTCCGGAGAGGGAGCCTGAGAAATGGCTACCACTTCTACGGAAGGCAGCAGGC
GCGCAAATTACTCAATCCCAATACGGGGAAGTAGTGACAATAAATATCAATACCTATCCTTAACGGAGGGCAATTGA
AATGAACACAAATTAAAACTCTTAATTAACACAATTGGAGGGCAAGTCTGGTGCCAGCAGCCGCGGTAATTCCAGCT
CCAATAGCATATACTAAAGTTGTTGCAGTTAAAAAGCTCGTAGTTGAAGTTAAAGTTTTATTGGGCTAACGTTTTTTA
CCACCCTGTGGCTAAAATAACTCCAATATTTCTTTTTTTAATAGCTCAGTTTCTAGGTCTTTGACTCTAGTTATTTGGG
CATTTCACTGTGAGAAAATTGTGGTGTTTAAAGCAGGCGTCTCGTCTGATCTTTTGCAGCATGGTATGATGGAACATG
ACATTTTACGCTATTGGTTTGCGTCTAAAGTGTAATGATTAATAGGGATGGATGGGGATGTTCATATTGGTGGGCGAG
AGGTGAAATTCGTTGACCCTATCAAGATGAACTTCTGCGAAAGCATTCATCAAATACTTCCCCATTAATCAAGAACG
AAAGTTTGGGGATCGAAGACGATCAGATACCGTCGTAGTCCAAACTATAAACTATGTCGACCAGGGATCGGCTAATA
TTTTTTTAAAATTTAGTCGGCACCTTGTGAGAAATCATGAGTGTTTAGATTCCGGGGGGAGTATGGTCGCAAGTCTGA
AACTTAAAGGAATTGACGGAAGGGCACACAATGGAGTGGAGCCTGCGGCTTAATTTGACTCAACTCGGGAAAACTT
ACCAAGCTAAGATATAGTAAGGATTGACAGACTAAAAGATCTTTCATGATTCTATAAGTGGTGGTGCATGGTCGTTCT
TAGTTGGTGGAGCGATTTGTCTGGTCAATTCCGATAACGGACGAGACCTCGACCTGCTAACTAGTAGTATTTATTAGT
CGATATAGGCGATAGCTTCTCTGGGGTTGGAGTGCGGCAACGTATCTCTGCTTCAAGGAGTGTGTAGTCTGACTTGA
TAGGTACGTTAACAAAAAACTTCTTAGAGGGACTACCTGCCTCAAGCAGGCGGAAGTCCGAGGCAATAACAGGTCT
GTGATGCCCTTAGATACCTTGGGCCGCACGCGCGCTACAATGCAGATAGCAAAAAGGTTCCTGGTCTGGAAAGATTG
GGTAATCAATTGAATTTTCTGCGTAACTGGGATTGATCTTTGTAATTATTGATCATCAACGAGGAATTCCTTGTAAGCG
TAAGTCATTACCTTATGCTGAATATGTCCCTGCCCTTTGTACACACCGCCCGTCGCTCCTACCGATCGAATGATACGGT
AAAGTTAACGGATAGTTTTTTTGTGGCAACACAATTAAAATTAAAAGTTATTTAAATCTCATTGTTTAGAGGAAGGAG
AAGTCGTAACAAGGTATcCGTAGGTGAACCTGCGGATGGATCATTTTTT

>AM168060.1 *Dictyostelium purpureum* C143
AACCTGGTTGATCCTGCCAGTAGCCATATGCTTGTCTCAAAGATTAAGCCATGCATGTCTAAGTATAAATTCTTGTACG
ATGAAACTGCAGACGGCTCATTACAACAGTGATAAACTGCTAGACTTTCGGGTTTTTAACCTTTTGGATAACCGCAG
TAAATCGGGGCTAATACATAGAAGCGATGGGTGACTGGCAACGGAAGCTCAGCGATTATTAGCATTACTACCAATAC
CTTCGGGTCTTGTGGTGAAACCGAATAATATTGCAGATCGAGGATTTATCTTCGACAAGTCTACTGTGTCACTGCCCT
ATCAACTTTCGATGGTACGGTATTGGCCTACCATGGTTGTAACGGGTAACGGGGAATTAGGGTTCGATTCCGGAGAG
GGAGCCTGAGAAATGGCTACCACTTCTACGGAAGGCAGCAGGCGCGCAAATTACTCAATCCCAATACGGGGAAGTA
GTGACAATAAATATCAATACCTATCCTTTTTGGAGGGCAATTGAAATGAACACAAATTAAAACTCTTAATTAACACAA
TTGGAGGGCAAGTCTGGTGCCAGCAGCCGCGGTAATTCCAGCTCCAATAGCATATACTAAAGTTGTTGCAGTTAAAA
AGCTCGTAGTTGAAGTTAAAGTTTTATTGGGCTTAAGTTTTTTACCACCTAGTGGCTAAAACAACTCCAATAATTCTT
TTTTTAATAGCTCAGTTTCTAGGTCTTTGACTCTAGTTATTTGGGCATTTCACTGTGAGAAAATTGTGGTGTTTAAAGC
AGGCGTCTCGTCTGATCTTTTGCAGCATGGTATGATGGAACATGACATTTTACGCTATTGGTTTGCGTCTAAAGTGTA
ATGATTAATAGGGATGGATGGGGATGTTCATATTGGTGGGCGAGAGGTGAAATTCGTTGACCCTATCAAGATGAACTT
CTGCGAAAGCATTCATCAAATACTTCCCCATTAATCAAGAACGAAAGTTTGGGGATCGAAGACGATCAGATACCGTC
GTAGTCCAAACTATAAACTATGTCCACCAGGGATCGGTTAAAATTTTTTTAAAATTTAGTCGGCACCTTGTGAGAAAT
CATGAGTGTTTAGATTCCGGGGGGAGTATGGTCGCAAGTCTGAAATTAAAGGAATTGACGGAAGGGCACACAATGG

AGTGGAGCCTGCGGCTTAATTTGACTCAACTCGGGAAAACTTACCAAGCTAAGATATAGTAAGGATTGACAGACTAA
AAGATCTTTCATGATTCTATAAGTGGTGGTGCATGGTCGTTCTTAGTTGGTGGAGCGATTTGTCTGGTCAATTCCGATA
ACGGACGAGACCTCGACCTGCTAACTAGTAGTATTTATTAGTCAATATGGGCGATAGCTTTTCTGGGATTTAGAGCGA
TTTCGGTCGTTTCTGGTTTCAAGGAGTGTGTAGTCTGACTTGATAGGTACGAATTAAAAAAACTTCTTAGAGGGACT
ACCTGCCTCAAGCAGGCGGAAGTCCGAGGCAATAACAGGTCTGTGATGCCCTTAGATACCTTGGGCCGCACGCGCG
CTACAATGTATAAAACAAAAAGGTTCCTGGTCCGGAAGGATTGGGTAATCAATTGAATTTTCTACGTAACTGGGATTG
ATCTTTGTAATTATTGATCATAAACGAGGAATTCCTTGTAAGCGCAGGTCATTACCCTGTGCTGAATATGTCCCTGCCC
TTTGTACACACCGCCCGTCGCTCCTACCGATCGAATGATACGGTAAAGTTAACGGATAGTTTTATCTTTGGCAACATT
GATATAAATTAAAAGTTATTTAAATCTCATTGTTTAGAGGAAGGAGAAGTCGTAACAAGGTATCCGTAGGTGAACCTG
CAGAAGGATCAA

>AM168061.1 *Dictyostelium purpureum* WS321
AACCTGGTTGATCCTGCCAGTAGTCATATGCTTGTCTCAAAGATTAAGCCATGCATGTCTAAGTATAAATTCTTGTACG
ATGAAACTGCAGACGGCTCATTACAACAGTGATAAACTGCTAGACTTTCGGGTTTTTAACCTTTTGGATAACCGCAG
TAAATCGGGGCTAATACATAGAAGCGATGGGTGACTGGCAACGGAAGCTCAGCGATTATTAGCATTACTACCAATAC
CTTCGGGTCTTGTGGTGAAACCGAATAATATTGCAGATCGAGGATTTATCTTCGGCAAGTCTACTGTGTCACTGCCCT
ATCAACTTTCGATGGTACGGTATTGGCCTACCATGGTTGTAACGGGTAACGGGGAATTAGGGTTCGATTCCGGAGAG
GGAGCCTGAGAAATGGCTACCACTTCTACGGAAGGCAGCAGGCGCGCAAATTACTCAATCCCAATACGGGGAAGTA
GTGACAATAAATATCAATACCTATCCTTAACGGAGGGCAATTGAAATGAACACAAATTAAAACTCTTAATTAACACAA
TTGGAGGGCAAGTCTGGTGCCAGCAGCCGCGGTAATTCCAGCTCCAATAGCATATACTAAAGTTGTTGCAGTTAAAA
AGCTCGTAGTTGAAGTTAAAGTTTTATTGGGCTAACGTTTTTTACCGCTCTGCGGCTAAAATAACTCCAATAATTCTTT
TTTTAATAGCTCAGTTTCTAGGTCTTTGACTCTAGTTATTTGGGCATTTCACTGTGAGAAAATTGTGGTGTTTAAAGCA
GGCGTCTCGTCTGATCTTTTGCAGCATGGTATGATGGAACATGACATTTTACGCTATTGGTTTGCGTCTAAAGTGTAAT
GATTAATAGGGATGGATGGGGATGTTCATATTGGTGGGCGAGAGGTGAAATTCGTTGACCCTATCAAGATGAACTTCT
GCGAAAGCATTCATCAAATACTTCCCCATTAATCAAGAACGAAAGTTTGGGGATCGAAGACGATCAGATACCGTCGT
AGTCCAAACTATAAACTATGTCGACCAGGGATCGGCTAATATTTTTTTAAAATTTAGTCGGCACCTTGTGAGAAATCA
TGAGTGTTTAGATTCCGGGGGGAGTATGGTCGCAAGTCTGAAACTTAAAGGAATTGACGGAAGGGCACGCAATGGA
GTGGAGCCTGCGGCTTAATTTGACTCAACTCGGGAAAACTTACCAAGCTAAGATATAGTAAGGATTGACAGACTAAA
AGATCTTTCATGATTCTATAAGTGGTGGTGCATGGTCGTTCTTAGTTGGTGGAGCGATTTGTCTGGTCAATTCCGATAA
CGGACGAGACCTCGACCTGCTAACTAGTAGTATTTATTGGTCAATACGGGCGATAGCTTTTCTGGGGTTAGAGTGCG
GCAACGTATCTCTGCTTCAAGGAGTGTGTAGTCTGACTTGATAGGTACGTTAACAAAAAACTTCTTAGAGGGACTAC
CTGCCTCAAGCAGGCGGAAGTCCGAGGCAATAACAGGTCTGTGATGCCCTTAGATACCTTGGGCCGCACGCGCGCT
ACAATGCAGATAGCAAAAAGGTTCCTGGTCTGGAAAGATTGGGTAATCAATTGAATTTTCTGCGTAACTGGGATTGA
TCTTTGTAATTATTGATCATCAACGAGGAATTCCTTGTAAGCGTAAGTCATTACCTTATGCTGAATATGTCCCTGCCCT
TTGTACACACCGCCCGTCGCTCCTACCGATCGAATGATACGGTAAAGTTAACGGATAGTTTTTTTGTGGCAACACAAT
TAAAATTAAAAGTTATTTAAATCTCATTGTTTAGAGGAAGGAGAAGTCGTAACAAGGTATCCGTAGGTGAACCTGCA
GAAGGATCAA

>HQ141481.1 *Dictyostelium purpureum* cavender
AACCTGGTTGATCCTGCCAGTAGTCATATGCTTGTCTCAAAGATTAAGCCATGCATGTCTAGTATAAATTCTTGTACGA
TGAAACTGCAGACGGCTCATTACAACAGTGATAAACTGCTAGACTTTCGGGTTTTTAACCTTTTGGATAACCGCAGT
AAATCGGGGCTAATACATAGAAGCGATGGGTGACTGGCAACGGAAGCTCAGCGATTATTAGCATTACTACCAATACC
TTCGGGTCTTGTGGTGAAACCGAATAATATTGCAGATCGAGGATTTATCTTCGACAAGTCTACTGTGTCACTGCCCTA
TCAACTTTCGATGGTACGGTATTGGCCTACCATGGTTGTAACGGGTAACGGGGAATTAGGGTTCGATTCCGGAGAGG
GAGCCTGAGAAATGGCTACCACTTCTACGGAAGGCAGCAGGCGCGCAAATTACTCAATCCCAATACGGGGAAGTAG
TGACAATAAATATCAATACCTATCCTTAACGGAGGGCAATTGAAATGAACACAAATTAAAACTCTTAATTAACACAAT
TGGAGGGCAAGTCTGGTGCCAGCAGCCGCGGTAATTCCAGCTCCAATAGCATATACTAAAGTTGTTGCAGTTAAAAA
GCTCGTAGTTGAAGTTAAAGTTTTATTGGGCTAACGTTTTTTACCGCTCTGCTGGCTAAAATAACTCCAATAATTCTTT
TTTTAATAGCTCAGTTTCTAGGTCTTTGACTCTAGTTATTTGGGCATTTCACTGTGAGAAAATTGTGGTGTTTAAAGCA
GGCGTCTCGTCTGATCTTTTGCAGCATGGTATGATGGAACATGACATTTTACGCTATTGGTTTGCGTCTAAAGTGTAAT
GATTAATAGGGATGGATGGGGATGTTCATATTGGTGGGCGAGAGGTGAAATTCGTTGACCCTATCAAGATGAACTTCT
GCGAAAGCATTCATCAAATACTTCCCCATTAATCAAGAACGAAAGTTTGGGGATCGAAGACGATCAGATACCGTCGT
AGTCCAAACTATAAACTATGTCGACCAGGGATCGGCTAATATTTTTTTAAAATTTAGTCGGCACCTTGTGAGAAATCA
TGAGTGTTTAGATTCCGGGGGGAGTATGGTCGCAAGTCTGAAACTTAAAGGAATTGACGGAAGGGCACACAATGGA
GTGGAGCCTGCGGCTTAATTTGACTCAACTCGGGAAAACTTACCAAGCTAAGATATAGTAAGGATTGACAGACTAAA
AGATCTTTCATGATTCTATAAGTGGTGGTGCATGGTCGTTCTTAGTTGGTGGAGCGATTTGTCTGGTCAATTCCGATAA
CGGACGAGACCTCGACCTGCTAACTAGTAGTATTTATTGGTCAATACGGGCGATAGCTTTTCTGGGGTTAGAGTGCG
GCAACGTATCTCTGCTTCAAGGAGTGTGTAGTCTGACTTGATAGGTACGTTAACAAAAACTTCTTAGAGGGACTACC
TGCCTCAAGCAGGCGGAAGTCCGAGGCAATAACAGGTCTGTGATGCCCTTAGATACCTTGGGCCGCACGCGCGCTA
CAATGCAGATAGCAAAAAGGTTCCTGGTCTGGAAAGATTGGGTAATCAATTGAATTTTCTGCGTAACTGGGATTGAT
CTTTGTAATTATTGATCATCAACGAGGAATTCCTTGTAAGCGTAAGTCATTACCTTATGCTGAATATGTCCCTGCCCTT
TGTACACACCGCCCGTCGCTCCTACCGATCGAATGATACGGTAAAGTTAACGGATAGTTTTTTTGTGGCAACACAATT
AAAAATTAAAAGTTATTTAAATCTCATTGTTTAGAGGAAGGAGAAGTCGTAACAAGGTATCCGTAGGTGAACCTGCA
GAAGGATCA

>MH280022.1 *Dictyostelium purpureum* var. *pseudosessile* MR273 (4637)
TGCATGTCTAAGTATAAATTCTTGTACGATGAAACTGCAGACGGCTCATTACAACAGTGATAAACTGCTAGACTTTCG
GGTTTTAACCTTTTGGATAACCGCAGTAAATCGGGGCTAATACATACAAGCGATGGGTGACTGGCAACGGAAGCTCA
GCGATTATTAGCGTTACTACCAATACCTTCGGGTCTTGTGGTGAAACCGAATAATATTGCAGATCGGGGATTTATCTTC
GACAAGTCTACTGTGTCACTGCCCTATCAACTTTCGATGGTACGGTATTGGCCTACCATGGTTGTAACGGGTAACGGG
GAATTAGGGTTCGATTCCGGAGAGGGAGCCTGAGAAATGGCTACCACTTCTACGGAAGGCAGCAGGCGCGCAAATT
ACTCAATCCCAATACGGGGAAGTAGTGACAATAAATATCAATACCTATCCTTTTTGGAGGGCAATTGAAATGAACACA
AATTAAAACTCTTAATTAACACAATTGGAGGGCAAGTCTGGTGCCAGCAGCCGCGGTAATTCCAGCTCCAATAGCAT
ATACTAAAGTTGTTGCAGTTAAAAAGCTCGTAGTTGAAGTTAAAGTTATATTGGGCTCAAGTTTTTTACCACGTCTGT
GGCTAAAATAACTCCAATATTTCTTTTTTTAATAGCTCAGTTTCTAGGTCTTTGACTCTAGTTATTTGGGCATTTCACTG

TGAGAAAATTGTGGTGTTTAAAGCAGGCGTCTCGTCTGATCTTTTGCAGCATGGTATGATGGAACATGACATTTTACG
CTATTGGTTTGCGTCTAAAGTGTAATGATTAATAGGGATGGATGGGGATGTTCATATTGGTGGGCGAGAGGTGAAATT
CGTTGACCCTATCAAGATGAACTTCTGCGAAAGCATTCATCAAATACTTCCCCATTAATCAAGAACGAAAGTTTGGG
GATCGAAGACGATCAGATACCGTCGTAGTCCAAACTATAAACTATGTCGACCAGGGATCGGCTAAAATTTTTTTAAA
ATTTAGTCGGCACCTTGTGAGAAATCATGAGTGTTTAGATTCCGGGGGGAGTATGGTCGCAAGTCTGAAACTTAAAG
GAATTGACGGAAGGGCACACAATGGAGTGGAGCCTGCGGCTTAATTTGACTCAACTCGGGAAAACTTACCAAGCTA
AGATATAGTAAGGATTGACAGACTAAAAGATCTTTCATGATTCTATAAGTGGTGGTGCATGGTCGTTCTTAGTTGGTG
GAGCGATTTGTCTGGTCAATTCCGATAACGGACGAGACCTCGACCTGCTAACTAGTAGTATTTATTAGCCGATATGGG
CGATAGCTTCTCTGGGGTTTGAGTGCGGTAACGTATCTTGGCTTCAAGGAGTGTGTAGTCTGGCTTGATAGGTACGAT
ATTAAAAAACTTCTTAGAGGGACTACCTGCCTCAAGCAGGCGGAAGTCCGAGGCAATAACAGGTCTGTGATGCCCT
TAGATACCTTGGGCCGCACGCGCGCTACAATGCAGATAGCAAAAAGGTTCCTGGCCTGGAAAGGTTGGGTAATCAA
TTGAATTTTCTGCGTAACTGGGATTGATCTTTGTAATTATTGATCATCAACGAGGAATTCCTTGTAAGCGTAAGTCATT
ACCTTATGCTGAATATGTCCCTGCCCTTTGTACACACCGCCCGTCGCTCCTACCGATCGAATGATACGGTAAAGTTAA
CGGATTGTTTTTTGTGGCAACACAATTAAAATTAAAAGTTATTTAAATCTCATTGTTTAGAGGAAGGAGAAGTCGTA
ACAAGGTATCCGTAGGTGAAC

>MH280023.1 *Dictyostelium purpureum* var. *pseudosessile* MR273 (4446)
TAAATTCTTGTACGATGAAACTGCAGACGGCTCATTACAACAGTGATAAACTGCTAGACTTTCGGGTTTTAACCTTTT
GGATAACCGCAGTAAATCGGGGCTAATACATACAAGCGATGGGTGACTGGCAACGGAAGCTCAGCGATTATTAGCGT
TACTACCAATACCTTCGGGTCTTGTGGTGAAACCGAATAATATTGCAGATCGGGGATTTATCTTCGACAAGTCTACTG
TGTCACTGCCCTATCAACTTTCGATGGTACGGTATTGGCCTACCATGGTTGTAACGGGTAACGGGGAATTAGGGTTCG
ATTCCGGAGAGGGAGCCTGAGAAATGGCTACCACTTCTACGGAAGGCAGCAGGCGCGCAAATTACTCAATCCCAAT
ACGGGGAAGTAGTGACAATAAATATCAATACCTATCCTTTTTGGAGGGCAATTGAAATGAACACAAATTAAAACTCT
TAATTAACACAATTGGAGGGCAAGTCTGGTGCCAGCAGCCGCGGTAATTCCAGCTCCAATAGCATATACTAAAGTTG
TTGCAGTTAAAAAGCTCGTAGTTGAAGTTAAAGTTATATTGGGCTCAAGTTTTTTACCACGTCTGTGGCTAAAATAAC
TCCAATATTTCTTTTTTTAATAGCTCAGTTTCTAGGTCTTTGACTCTAGTTATTTGGGCATTTCACTGTGAGAAAATTGT
GGTGTTTAAAGCAGGCGTCTCGTCTGATCTTTTGCAGCATGGTATGATGGAACATGACATTTTACGCTATTGGTTTGC
GTCTAAAGTGTAATGATTAATAGGGATGGATGGGGATGTTCATATTGGTGGGCGAGAGGTGAAATTCGTTGACCCTAT
CAAGATGAACTTCTGCGAAAGCATTCATCAAATACTTCCCCATTAATCAAGAACGAAAGTTTGGGGATCGAAGACGA
TCAGATACCGTCGTAGTCCAAACTATAAACTATGTCGACCAGGGATCGGCTAAAATTTTTTTAAAATTTAGTCGGCAC
CTTGTGAGAAATCATGAGTGTTTAGATTCCGGGGGGAGTATGGTCGCAAGTCTGAAACTTAAAGGAATTGACGGAA
GGGCACACAATGGAGTGGAGCCTGCGGCTTAATTTGACTCAACTCGGGAAAACTTACCAAGCTAAGATATAGTAAG
GATTGACAGACTAAAAGATCTTTCATGATTCTATAAGTGGTGGTGCATGGTCGTTCTTAGTTGGTGGAGCGATTTGTC
TGGTCAATTCCGATAACGGACGAGACCTCGACCTGCTAACTAGTAGTATTTATTAGCCGATATGGGCGATAGCTTCTC
TGGGGTTTGAGTGCGGTAACGTATCTTGGCTTCAAGGAGTGTGTAGTCTGGCTTGATAGGTACGATATTAAAAAACT
TCTTAGAGGGACTACCTGCCTCAAGCAGGCGGAAGTCCGAGGCAATAACAGGTCTGTGATGCCCTTAGATACCTTG
GGCCGCACGCGCGCTACAATGCAGATAGCAAAAAGGTTCCTGGCCTGGAAAGGTTGGGTAATCAATTGAATTTTCT
GCGTAACTGGGATTGATCTTTGTAATTATTGATCATCAACGAGGAATTCCTTGTAAGCGTAAGTCATTACCTTATGCTG
AATATGTCCCTGCCCTTTGTACACACCGCCCGTCGCTCCTACCGATCGAATGATACGGTAAAGTTAACGGATTGTTTT
TTTGTGGCAACACAATTAAAATTAAAAGTTATTTAAATCTCATTGTTTAGAGGAAGGAGAAGTCGTAACAAGGTATC
CGTAGGTGAACCTGCA

>HQ141479.1 *Dictyostelium quercibrachium* NZ201B
AACCTGGTTGATCCTGCCAGTAGTCATATGCTTGTCTCAAAGATTAAGCCATGCATGTCTAAGTATAAATTCTTGTACG
ATGAAACTGCAGACGGCTCATTACAACAGTGATAAACTAATAGACTTTCGGGTTTTTAACCTTTTGGATAACCGCAGT
AAATCGGGGCTAATACATAGAAGCGATGGGTGACTGGCAACGGAAGCTCAGCGATTATTAGCATTACTACCAATACC
TTCGGGTCTTGTGGTGAAACCGAATAATATTGCAGATCGAGGATTTATCTTCGACAAGTCTACTGTGTCACTGCCCTA
TCAACTTTCGATGGTACGGTATTGGCCTACCATGGTTGTAACGGGTAACGGGGAATTAGGGTTCGATTCCGGAGAGG
GAGCCTGAGAAATGGCTACCACTTCTACGGAAGGCAGCAGGCGCGCAAATTACTCAATCCCAATACGGGGAAGTAG
TGACAATAAATATCAATACCTATCCTTAACGGAGGGCAATTGAAATGAACACAAATTAAAACTCTTAATTAACACAAT
TGGAGGGCAAGTCTGGTGCCAGCAGCCGCGGTAATTCCAGCTCCAATAGCATATACTAAAGTTGTTGCAGTTAAAAA
GCTCGTAGTTGAAGTTAAAGGTTTACTGGGCTAAAGTTATTTGCCGCTCTGGTGGTTAAATCAACTCCAGTATCTCTT
TTTTTAATAGTTCAGCTTCTATTATCTTTGATAGTAGTTGTTTGGACATTTCACTGTGAGAAAATTGTGGTGTTTAAAG
CAGGCGTTTCGCCTGATCTTTTGCAGCATGGTATGATGGAACATGACATTTTACGCTATTGGTTTGCGTCTAAAGTGT
AATGATTAATAGGGATGGATGGGGGTGTTCATATTGGTGGGCGAGAGGTGAAATTCGTTGACCCTATCAAGATGAAC
TTCTGCGAAAGCATTCACCAAATACTTCCCCATTAATCAAGAACGAAAGTTTGGGGATCGAAGACGATCAGATACCG
TCGTAGTCCAAACTATAAACTATGTCGACCAGGGATCGGTTAAAATTTTTTTAAAATTTAATCGGCACCTTGTGAGAA
ATCACGAGTGTTTAGATTCCGGGGGGAGTATGGTCGCAAGTCTGAAACTTAAAGGAATTGACGGAAGGGCACACAA
TGGAGTGGAGCCTGCGGCTTAATTTGACTCAACTCGGGAAAACTTACCAAGCTAAGATATAGTAAGGATTGACAGAC
TAAAAGATCTTTCATGATTCTATAAGTGGTGGTGCATGGTCGTTCTTAGTTGGTGGAGCGATTTGTCTGGTCAATTCC
GATAACGGACGAGACCTCGACCTGCTAACTAGTAGTATTTATTAGTCAATATGGGCGATAGCTTCTCTGGGGTTTGGA
ATGGTTTCGGCCATCTCCTGCTTCAAGGAGTGTGTAGTCTGACTTGATAGGTACGAATTAAAAAAACTTCTTAGAGG
GACTACCTGCCTCAAGCAGGCGGAAGTCCGAGGCAATAACAGGTCTGTGATGCCCTTAGATACCTTGGGCCGCACG
CGCGCTACAATGTAGAAAACAAAAAGGTTCCTGGTCCGGAAGGATTGGGTAATCAATTGAATTTTCTACGTAACTGG
GATTGATCTTTGTAATTATTGATCATCAACGAGGAATTCCTTGTAAGCGTAGGTCATTACCCTATGCTGAATATGTCCC
TGCCCTTTGTACACACCGCCCGTCGCTCCTACCGATCGAATGATACGGTAAAGTTAACGGATCGTTTTATCTGTGGCA
ACACTGATATAAACTAAAAGTTATTTAAATCTCATTGTTAGAGAAGGAGAAGTCGTAACAAGGTATCCGTAGGTGAA
CCTGCAGAAGGATCA

>MW931857.1 *Dictyostelium robusticaule* 5729-bai-2021
CAGGAATCTTTGTCCAGATTAGCCATGCATGTCTAAGTATAAATTCTTGTACGATGAAACTGCAGACGGCTCATTACA
ACAGTGATAAACTAATAGACTTTCGGGTTTTTAACCTTTTGGATAACCGCAGTAAATCGGGGCTAATACATAGAAGCG
ATGGGTGACTGGCAACGGAAGCTCAGCGATTATTAGCATTACTACCAATACCTTCGGGTCTTGTGGTGAAACCGAAT
AATATTGCAGATCGAGGATTTATCTTCGACAAGTCTACTGTGTCACTGCCCTATCAACTTTCGATGGTACGGTATTGGC
CTACCATGGTTGTAACGGGTAACGGGGAATTAGGGTTCGATTCCGGAGAGGGAGCCTGAGAAATGGCTACCACTTC

TACGGAAGGCAGCAGGCGCGCAAATTACTCAATCCCAATACGGGGAAGTAGTGACAATAAATATCAATACCTATCCT
TAACGGAGGGCAATTGAAATGAACACAAATTAAAACTCTTAATTAACACAATTGGAGGGCAAGTCTGGTGCCAGCA
GCCGCGGTAATTCCAGCTCCAATAGCATATACTAAAGTTGTTGCAGTTAAAAAGCTCGTAGTTGAAGTTAAAGGTTTA
CTGGGCTAAAGTTATTTGCCGCTCTGGTGGTTAAATCAACTCCAGTATCTCTTTTTTTAATAGTTCAGCTTCTATTATCT
TTGATAGTAGTTGTTTGGACATTTCACTGTGAGAAAATTGTGGTGTTTAAAGCAGGCGTTTCGCCTGATCTTTTGCAG
CATGGTATGATGAAACATGACATTTTACGCTATTGGTTTGCGTCTAAAGTGTAATGATTAATAGGGATGGATGGGGGT
GTTCATATTGGTGGGCGAGAGGTGAAATTCGTTGACCCTATCAAGATGAACTTCTGCGAAAGCATTCACCAAATACT
TCCCCATTAATCAAGAACGAAAGTTTGGGGATCGAAGACGATCAGATACCGTCGTAGTCCAAACTATAAACTATGTC
GACCAGGGATCGGTTAAAATTTTTTTAAAATTTAATCGGCACCTTGTGAGAAATCACGAGTGTTTAGATTCCGGGGG
GAGTATGGTCGCAAGTCTGAAACTTAAAGGAATTGACGGAAGGGCACACAATGGAGTGGAGCCTGCGGCTTAATTT
GACTCAACTCGGGAAAACTTACCAAGCTAAGATATAGTAAGGATTGACAGACTAAAAGATCTTTCATGATTCTATAA
GTGGTGGTGCATGGTCGTTCTTAGTTGGTGGAGCGATTTGTCTGGTCAATTCCGATAACGGACGAGACCTCGACCTG
CTAACTAGTAGTATTTATTAGTCAATATGGGCGATAGCTTCTCTGGGGTTTGGAATGGTTTCGGCCATCTCCTGCTTCA
AGGAGTGTGTAGTCTGACTTGATAGGTACGAATTAAAAAACTTCTTAGAGGGACTACCTGCCTCAAGCAGGCGGAA
GTCCGAGGCAATAACAGGTCTGTGATGCCCTTAGATACCTTGGGCCGCACGCGCGCTACAATGTAGAAAACAAAAA
GGTTCCTGGTCCGGAAGGATTGGGTAATCATTTGAATTTTCTACGTAACTGGGATTGATCTTTGTAATTATTGATCATC
AACGAGGAATTCCTTGTAAGCGTAGGTCATTACCCTATGCTGAATATGTCCCTGCCCTTTGTACACACCGCCCGTCGC
TCCTACCGATCGAATGATACGGTAAAGTTAACGGATCGTTTTATCTTTGGCAACATTGATATAAACTAAAAGTTATTTA
AATCTCATTGTTAGAGAAGAGAAGTCGAAAACATGGCCCC

>MW931856.1 *Dictyostelium robusticaule* 5729-huang-2021
GGAATGTTTTGTCCCAAGATTAGCCATGCATGTCTAAGTATAAATTCTTGTACGATGAAACTGCAGACGGCTCATTAC
AACAGTGATAAACTAATAGACTTTCGGGTTTTTAACCTTTTGGATAACCGCAGTAAATCGGGGCTAATACATAGAAGC
GATGGGTGACTGGCAACGGAAGCTCAGCGATTATTAGCATTACTACCAATACCTTCGGGTCTTGTGGTGAAACCGAA
TAATATTGCAGATCGAGGATTTATCTTCGACAAGTCTACTGTGTCACTGCCCTATCAACTTTCGATGGTACGGTATTGG
CCTACCATGGTTGTAACGGGTAACGGGGAATTAGGGTTCGATTCCGGAGAGGGAGCCTGAGAAATGGCTACCACTT
CTACGGAAGGCAGCAGGCGCGCAAATTACTCAATCCCAATACGGGGAAGTAGTGACAATAAATATCAATACCTATCC
TTAACGGAGGGCAATTGAAATGAACACAAATTAAAACTCTTAATTAACACAATTGGAGGGCAAGTCTGGTGCCAGC
AGCCGCGGTAATTCCAGCTCCAATAGCATATACTAAAGTTGTTGCAGTTAAAAAGCTCGTAGTTGAAGTTAAAGGTT
TACTGGGCTAAAGTTATTTGCCGCTCTGGTGGTTAAATCAACTCCAGTATCTCTTTTTTTAATAGTTCAGCTTCTATTAT
CTTTGATAGTAGTTGTTTGGACATTTCACTGTGAGAAAATTGTGGTGTTTAAAGCAGGCGTTTCGCCTGATCTTTTGC
AGCATGGTATGATGAAACATGACATTTTACGCTATTGGTTTGCGTCTAAAGTGTAATGATTAATAGGGATGGATGGGG
GTGTTCATATTGGTGGGCGAGAGGTGAAATTCGTTGACCCTATCAAGATGAACTTCTGCGAAAGCATTCACCAAATA
CTTCCCCATTAATCAAGAACGAAAGTTTGGGGATCGAAGACGATCAGATACCGTCGTAGTCCAAACTATAAACTATG
TCGACCAGGGATCGGTTAAAATTTTTTTAAAATTTAATCGGCACCTTGTGAGAAATCACGAGTGTTTAGATTCCGGGG
GGAGTATGGTCGCAAGTCTGAAACTTAAAGGAATTGACGGAAGGGCACACAATGGAGTGGAGCCTGCGGCTTAATT
TGACTCAACTCGGGAAAACTTACCAAGCTAAGATATAGTAAGGATTGACAGACTAAAAGATCTTTCATGATTCTATAA
GTGGTGGTGCATGGTCGTTCTTAGTTGGTGGAGCGATTTGTCTGGTCAATTCCGATAACGGACGAGACCTCGACCTG
CTAACTAGTAGTATTTATTAGTCAATATGGGCGATAGCTTCTCTGGGGTTTGGAATGGTTTCGGCCATCTCCTGCTTCA
AGGAGTGTGTAGTCTGACTTGATAGGTACGAATTAAAAAACTTCTTAGAGGGACTACCTGCCTCAAGCAGGCGGAA
GTCCGAGGCAATAACAGGTCTGTGATGCCCTTAGATACCTTGGGCCGCACGCGCGCTACAATGTAGAAAACAAAAA
GGTTCCTGGTCCGGAAGGATTGGGTAATCATTTGAATTTTCTACGTAACTGGGATTGATCTTTGTAATTATTGATCATC
AACGAGGAATTCCTTGTAAGCGTAGGTCATTACCCTATGCTGAATATGTCCCTGCCCTTTGTACACACCGCCCGTCGC
TCCTACCGATCGAATGATACGGTAAAGTTAACGGATCGTTTTATCTTTGGCAACATTGATATAAACTAAAAGTTATTTA
AATCTCATTGTTAGAGAAGGAGAAGTCGAAGCAATTTCGGGGTTC

>AM168064.1 *Dictyostelium robustum* TNS-C-219
AACCTGGTTGATCCTGCCAGTAGTCATATGCTTGTCTCAAAGATTAAGCCATGCATGTCTAAGTATAAATTCTTGTACG
ATGAAACTGCAGACGGCTCATTACAACAGTGATAAACTAATAGACTTTCGGGTTTTTAACCTTTTGGATAACCGCAGT
AAATCGGGGCTAATACATAGAAGCGATGGGTGACTGGCAACGGAAGCTCAGCGATTATTAGCATTACTACCAATACC
TTCGGGTCTTGTGGTGAAACCGAATAATATTGCAGATCGAGGATTTATCTTCGACAAGTCTATTGTGTCACTGCCCTA
TCAACTTTCGATGGTACGGTATAGGCCTACCATGGTTGTAACGGGTAACGGGGAATTAGGGTTCGATTCCGGAGAGG
GAGCCTGAGAAATGGCTACCACTTCTACGGAAGGCAGCAGGCGCGCAAATTACTCAATCCCAATACGGGGAAGTAG
TGACAATAAATATCAATACCTATCCTTTTTGGAGGGCAATTGAAATGAACACAAATTAAAACTCTTAATTAACACAAT
TGGAGGGCAAGTCTGGTGCCAGCAGCCGCGGTAATTCCAGCTCCAATAGCATATACTAAAGTCGTTGCAGTTAAAAA
GCTCGTAGTTGAAGTTAAGGGTTTATCGGGCTAAAGTTATTTGCCACTTTGTGGTTAAATTCAACTCCGGTATTCCTTT
TCTTAATAGTTCAGCTTCTATTATCTTTGATAGTAGTTGTTTGGGCATTTCACTGTGAGAAAATTGTGGTGTTTAAAGC
AGGCGTCTCGCCTGATCTTTTGCAGCATGGTATGATGAAACATGACATTTTACGCTATTGGTTTGCGTCTAAAGTGTA
ATGATTAATAGGGATGGATGGGGGTGTTCATATTGGTGGGCGAGAGGTGAAATTCGTTGACCCTATCAAGATGAACTT
CTGCGAAAGCATTCACCAAATACTTCCCCATTAATCAAGAACGAAAGTTTGGGGATCGAAGACGATCAGATACCGTC
GTAGTCCAAACTATAAACTATGTCGACCAGGGATCGGTTAAAATTTTTTTAAAATTTAATCGGCACCTTGTGAGAAAT
CACGAGTGTTTAGATTCCGGGGGGAGTATGGTCGCAAGTCTGAAACTTAAAGGAATTGACGGAAGGGCACACAATG
GAGTGGAGCCTGCGGCTTAATTTGACTCAACTCGGGAAAACTTACCAAGCTAAGATATAGTAAGGATTGACAGACTA
AAAGATCTTTCATGATTCTATAAGTGGTGGTGCATGGTCGTTCTTAGTGGGTGGAGCGATTTGTCTGGTCAATTCCGA
TAACGGACGAGACCTCGACCTGCTAACTAGTAGTATTTATTAGTCGATATGGGCGATAGCTTCTCTGGGGTTTGGAAC
GGCTTCGGCCGTCTCCTGCTTCAAGGAGTGTGTAGTCTGACTTGATAGGTACGAATTCAAAAACTTCTTAGAGGGAC
TACCTGCCTCAAGCAGGCGGAAGTCCGAGGCAATAACAGGTCTGTGATGCCCTTAGATACCTTGGGCCGCACGCGC
GCTACAATGTAGAAAACAAAAAGGTTCCTGGTCCGGAAGGATTGGGTAATCAATTGAATTTTCTACGTAACTGGGAT
TGATCTTTGTAATTATTGATCATCAACGAGGAATTCCTTGTAAGCGTAAGTCATTACCTTATGCTGAATATGTCCCTGC
CCTTTGTACACACCGCCCGTCGCTCCTACCGATCGAATGATACGGTAAAGTTAACGGATCGTTTTATTTGTGGCAACA
CAAATTAAGACTAAAAGTTATTTAAATCTCATTGTTTAGAGGAAGGAGAAGTCGTAACAAGGTATCCGTAGGTGAAC
CTGCAGAAGGATCAA

>AM168065.1 *Dictyostelium rosarium* M45
AACCTGGTTGATCCTGCCAGTAGTCATATGCTTGTCTCAAAGATTAAGCCATGCATGTCTAAGTATAAATTCTTGTACG

ATGAAACTGCAGACGGCTCATTACAACAGTGATAAACTAATAGACTTTCGGGTTTTAACCTTTTGGATAACCGCAGT
AAATCGGGGCTAATACATAGAAGCGATGGGCGACTGGCAACGGAAGCTCAGCGATTATTAGCATTACTACCAATACC
TTCGGGTCTTGTGGTGAAACCGAATAATATTGCAGATCGAGGATTTATCTTCGACAAGTCTACTGTGTCACTGCCCTA
TCAACTTTCGATGGTACGGTATTGGCCTACCATGGTTGTAACGGGTAACGGGGAATTAGGGTTCGATTCCGGAGAGG
GAGCCTGAGAAATGGCTACCACTTCTACGGAAGGCAGCAGGCGCGCAAATTACTCAATCCCAATACGGGGAAGTAG
TGACAATAAATATCAATACCTATCCTTAACGGAGGGCAATTGAAATGAACACAAATTAAAACTCTTAATTAACACAAT
TGGAGGGCAAGTCTGGTGCCAGCAGCCGCGGTAATTCCAGCTCCAATAGCATATACTAAAGTTGTTGCAGTTAAAAA
GCTCGTAGTTGAAGTTAAAGGTTTACTGGGCTAAAGTTATTTGCCGCTCTGGTGGTTAAATCAACTCCAGTATCTCTT
TTTTTAATAGTTCAGCTTCTATTATCTTTGATAGTAGTTGTTTGGACATTTCACTGTGAGAAAATTGTGGTGTTTAAAG
CAGGCGTTTCGCCTGATCTTTTGCAGCATGGTATGATGAAACATGACATTTTACGCTATTGGTTTGCGTCTAAAGTGT
AATGATTAATAGGGATGGATGGGGGTGTTCATATTGGTGGGCGAGAGGTGAAATTCGTTGACCCTATCAAGATGAAC
TTCTGCGAAAGCATTCACCAAATACTTCCCCATTAATCAAGAACGAAAGTTTGGGGATCGAAGACGATCAGATACCG
TCGTAGTCCAAACTATAAACTATGTCGACCAGGGATCGGTTAAAATTTTTTTAAAATTTAATCGGCACCTTGTGAGAA
ATCACGAGTGTTTAGATTCCGGGGGGAGTATGGTCGCAAGTCTGAAACTTAAAGGAATTGACGGAAGGGCACACAA
TGGAGTGGAGCCTGCGGCTTAATTTGACTCAACTCGGGAAAACTTACCAAGCTAAGATATAGTAAGGATTGACAGAC
TAAAAGATCTTTCATGATTCTATAAGTGGTGGTGCATGGTCGTTCTTAGTTGGTGGAGCGATTTGTCTGGTCAATTCC
GATAACGGACGAGACCTCGACCTGCTAACTAGTAGTATTTATTAGTCAATATGGGCGATAGCTTCTCTGGGGTTTGGA
ATGGCTTCGGTCATCTCCTGCTTCAAGGAGTGTGTAGTCTGACTTGATAGGTACGAATTAAAAAACTTCTTAGAGGG
ACTACCTGCCTCAAGCAGGCGGAAGTCCGAGGCAATAACAGGTCTGTGATGCCCTTAGATACCTTGGGCCGCACGC
GCGCTACAATGTAGAAAACAAAAAGGTTCCTGGTCCGGAAGGATTGGGTAATCATTTGAATTTTCTACGTAACTGGG
ATTGATCTTTGTAATTATTGATCATCAACGAGGAATTCCTTGTAAGCGTAGGTCATTACCCTATGCTGAATATGTCCCT
GCCCTTTGTACACACCGCCCGTCGCTCCTACCGATCGAATGATACGGTAAAGTTAACGGATCGTTTTATCTTTGGCAA
CATTGATATAAACTAAAAGTTATTTAAATCTCATTGTTTAGAGGAAGGAGAAGTCGTAACAAGGTATCCGTAGGTGAA
CCTGCAGAAGGATCAA

>AM168066.1 *Dictyostelium septentrionale* IY49
AACCTGGTTGATCCTGCCAGTAGTCATATGCTTGTCTCAAAGATTAAGCCATGCATGTCTAAGTATAAGTTCTTGTAC
GATGAAACTGCAGACGGCTCATTACAACAGTGATAAACTATTAGACTTTCGGGTTTTTACCTTTTGGATAACCGCAGT
AAATCGGGGCTAATACATAGAAGCGATGGGTAACTGGCAACGGAAGCTCAGCGATTATTAGCATCTATCAATACCTTC
GGGTCTTGTGGTGAAACCGAATAATATTGCAGATCGAAGATTTATCTTCGACAAGTCTATTGTGTCACTGCCCTATCA
ACTTTCGATGGTACGGTATTGGCCTACCATGGTTGTAACGGGTAACGGGGAATTAGGGTTCGATTCCGGAGAGGGAG
CCTGAGAAATGGCTACCACTTCTACGGAAGGCAGCAGGCGCGCAAATTACTCAATCCCAATACGGGGAAGTAGTGA
CAATAAATATCAATGCCTATCCTTAACGGAGGGCAATTGAAATGAACACAAATTAAAACTCTTAATTAACACAATTGG
AGGGCAAGTCTGGTGCCAGCAGCCGCGGTAATTCCAGCTCCAATAGCATATACTAAAGTTGTTGCAGTTAAAAAGCT
CGTAGTTGAAGTCAAAGGTTTGTCGGGTCAAAGTTGTTTCTCCGCTCTGGTGGTTAAATATACTCCGGCATCTCTTTT
TTTAATAGTTCTGCTTGTGCTATCTTTGATGGTACTTGTTTGAACAGTTCACCGTGAGAAAATTGTGGTGTTCAAAGC
AGGCGTTTCGCCTGATCTTTTGCAGCATGGTATGATGAAACATGACATTTTACGCTATTGGTTTGCGTCTAGAGTGTA
ATGATTAATAGGGATGGATGGGGGTGTTCATATTGGTGGGCGAGAGGTGAAATTCGTTGACCCTATCAAGATGAACTT
CTGCGAAAGCATTCGCCAAATACTTCTCCATTAATCAAGAACGAAAGTTTGGGGATCGAAGACGATCAGATACCGTC
GTAGTCCAAACTATAAACTATGTCGACCAGGGATCGGTTAAAATTTTTTTAAAATTTAATCGGCACCTTGTGAGAAAT
CACGAGTGTTTAGATTCCGGGGGGAGTATGGTCGCAAGTCTGAAACTTAAAGGAATTGACGGAAGGGCACACAATG
GAGTGGAGCCTGCGGCTTAATTTGACTCAACTCGGGAAAACTTACCAAGCTAAGATATAGTAAGGATTGACAGACTA
AAAGATCTTTCATGATTCTATAAGTGGTGGTGCATGGTCGTTCTTAGTTGGTGGAGCGATTTGTCTGGTCAATTCCGA
TAACGGACGAGACCTCGACCTGCTAACTAGTAGTGTTTATTTCGCCAATATGGGCGATAGCTTTTTTGGGATTTCGGT
TCATTTCGGTGGATTCGGGTTTCAGGGAGTGTGTAGTCTGGTGGGATAGGCACGAATTCAAAAAACTTCTTAAAGGG
ACTACCTGCCTCAAGCAGGCGGAAGTCCGAGGCAATAACAGGTCTGTGATGCCCTTAGATACCTTGGGCCGCACGC
GCGCTACAATGTAGAAAACAAAAAGGTTCCTGGTCTGGAAGGATTGGGTAATCAATTGAATTTTCTACGTAACTGGG
ATTGATCTTTGTAATTATCGATCATCAACGAGGAATTCCTTGTAAGCGTAGGTCATTACCCTATGCTGAATATGTCCCT
GCCCTTTGTACACACCGCCCGTCGCTCCTACCGATCGAATGATACGGTAAAGTTAACGGATTGTTTTGCTTTCGTTAT
AGGGGCATTATGACAAGTTATTTAAATCTCATTGTTTAGAGGAAGGAGAAGTCGTAACAAGGTATCCGTAGGTGAAC
CTGCAGAAGGATCAA

>AM168067.1 *Dictyostelium septentrionale* AK2
AACCTGGTTGATCCTGCCAGTAGTCATATGCTTGTCTCAAAGATTAAGCCATGCATGTCTAAGTATAAATTCTTGTACG
ATGAAACTGCAGACGGCTCATTACAACAGTGATAAACTATTAGACTTTCGGGTTTTTACCTTTTGGATAACCGCAGTA
AATCGGGGCTAATACATAGAAGCGATGGGTAACTGGCAACGGAAGCTCAGCGATTATTAGCATCTATCAATACCTTCG
GGTCTTGTGGTGAAACCGAATAATATTGCAGATCGAAGATTTATCTTCGACAAGTCTATTGTGTCACTGCCCTATCAA
CTTTCGATGGTACGGTATTGGCCTACCATGGTTGTAACGGGTAACGGGGAATTAGGGTTCGATTCCGGAGAGGGAGC
CTGAGAAATGGCTACCACTTCTACGGAAGGCAGCAGGCGCGCAAATTACTCAATCCCAATACGGGGAAGTAGTGAC
AATAAATATCAATGCCTATCCTTATGGAGGGCAATTGAAATGAACACAAATTAAAACTCTTAATTAACACAATTGGAG
GGCAAGTCTGGTGCCAGCAGCCGCGGTAATTCCAGCTCCAATAGCATATACTAAAGTTGTTGCAGTTAAAAAGCTCG
TAGTTGAAGTCAAAGGTTTGTCGGGTCAAAGTTGTTTTTCCGCTCTGGTGGTAAACATACTCCGGCATCTCTTTTTTT
AATAGTTCTGCTTGTGCTATCTTTGATGGTACTTGTTTGAACAGTTCACCGTGAGAAAATTGTGGTGTTCAAAGCAG
GCGTTTCGCCTGATCTTTTGCAGCATGGTATGATGAAACATGACATTTTACGCTATTGGTTTGCGTCTAGAGTGTAATG
ATTAATAGGGATGGATGGGGGTGTTCATATTGGTGGGCGAGAGGTGAAATTCGTTGACCCTATCAAGATGAACTTCT
GCGAAAGCATTCACCAAATACTTCTCCATTAATCAAGAACGAAAGTTTGGGGATCGAAGACGATCAGATACCGTCGT
AGTCCAAACTATAAACTATGTCGACCAGGGATCGGTTAAAATTTTTTTAAAATTTAATCGGCACCTTGTGAGAAATCA
CGAGTGTTTAGATTCCGGGGGGAGTATGGTCGCAAGTCTGAAACTTAAAGGAATTGACGGAAGGGCACACAATGGA
GTGGAGCCTGCGGCTTAATTTGACTCAACTCGGGAAAACTTACCAAGCTAAGATATAGTAAGGATTGACAGACTAAA
AGATCTTTCATGATTCTATAAGTGGTGGTGCATGGTCGTTCTTAGTTGGTGGAGCGATTTGTCTGGTCAATTCCGATAA
CGGACGAGACCTCGACCTGCTAACTAGTAGTGTTTATTTCGCCAATATGGGCGATAGCTTTTTTGGGATTTCGGTTCA
TTTCGGTGGATTCGGGTTTCAGGGAGTGTGTAGTCTGGTGGGATAGGCACGAATTCAAAAAACTTCTTAGAGGGAC
TACCTGCCTCAAGCAGGCGGAAGTCCGAGGCAATAACAGGTCTGTGATGCCCTTAGATACCTTGGGCCGCACGCGC
GCTACAATGTAGAAAACAAAAAGGTTCCTGGTCTGGAAGGATTGGGTAATCAATTGAATTTTCTACGTAACTGGGAT
TGATCTTTGTAATTATCGATCATCAACGAGGAATTCCTTGTAAGCGTAGGTCATTACCCTATGCTGAATATGTCCCTGC

CCTTTGTACACACCGCCCGTCGCTCCTACCGATCGAATGATACGGTAAAGTTAACGGATTGTTTTGCTTTCGTTATAG
GGGCATTATGAAAAGTTATTTAAATCTCATTGTTTAGAGGAAGGAGAAGTCGTAACAAGGTATCCGTAGGTGAACCT
GCAGAAGGATCAA

>AM168068.1 *Dictyostelium sphaerocephalum* GR11
AACCTGGTTGATCCTGCCAGTAGTCATATGCTTGTCTCAAAGATTAAGCCATGCATGTCTAAGTATAAATTCTTGTACG
ATGAAACTGCAGACGGCTCATTACAACAGTGATAAACTAATAGACTTTCGGGTTTTTAACCTTTTGGATAACCGCAGT
AAATCGGGGCTAATACATAGAAGCGATGGGTGACTGGCAACGGAAGCTCAGCGATTATTAGCATTACTACCGATACC
TTCGGGTCTTGTGGTGAAACCGAATAATATTGCAGATCGAGGATTTATCTTCGACAAGTCTACTGTGTCACTGCCCTA
TCAACTTTCGATGGTACGGTATTGGCCTACCATGGTTGTAACGGGTAACGGGGAATTAGGGTTCGATTCCGGAGAGG
GAGCCTGAGAAATGGCTACCACTTCTACGGAAGGCAGCAGGCGCGCAAATTACTCAATCCCAATACGGGGAAGTAG
TGACAATAAATATCAATACCTATCCTTAACGGAGGGCAATTGAAATGAACACAAATTAAAACTCTTAATTAACACAAT
TGGAGGGCAAGTCTGGTGCCAGCAGCCGCGGTAATTCCAGCTCCAATAGCATATACTAAAGTTGTTGCAGTTAAAAA
GCTCGTAGTTGAAGTTAAAGGTTTACTGGGCTAAAGTTATTTGCCGCTCTGGTGGTTAAATCAACTCCAGTATCTCTT
TTTTTAATAGTTCAGCTTCTATTATCTTTGATAGTAGTTGTTTGGACATTTCACTGTGAGAAAATTGTGGTGTTTAAAG
CAGGCGTTTCGCCTGATCTTTTGCAGCATGGTATGATGAAACATGACATTTTACGCTATTGGTTTGCGTCTAAAGTGT
AATGATTAATAGGGATGGATGGGGGTGTTCATATTGGTGGGCGAGAGGTGAAATTCGTTGACCCTATCAAGATGAAC
TTCTGCGAAAGCATTCACCAAATACTTCCCCATTAATCAAGAACGAAAGTTTGGGGATCGAAGACGATCAGATACCG
TCGTAGTCCAAACTATAAACTATGTCGACCAGGGATCGGTTAAAATCTTTTTAAAATTTAATCGGCACCTTGTGAGAA
ATCACGAGTGTTTAGATTCCGGGGGGAGTATGGTCGCAAGTCTGAAACTTAAAGGAATTGACGGAAGGGCACACAA
TGGAGTGGAGCCTGCGGCTTAATTTGACTCAACTCGGGAAAACTTACCAAGCTAAGATATAGTAAGGATTGACAGAC
TAAAAGATCTTTCATGATTCTATAAGTGGTGGTGCATGGTCGTTCTTAGTTGGTGGAGCGATTTGTCTGGTCAATTCC
GATAACGGACGAGACCTCGACCTGCTAACTAGTAGTATTTATTAGTCAATATGGGCGATAGCTTCTCTGGGGTTTGGA
ATGGTTTCGGCCATCTCCTGCTTCAAGGAGTGTGTAGTCTGACTTGATAGGTACGAATTAAAAAACTTCTTAGAGGG
ACTACCTGCCTCAAGCAGGCGGAAGTCCGAGGCAATAACAGGTCTGTGATGCCCTTAGATACCTTGGGCCGCACGC
GCGCTACAATGTAGAAAACAAAAAGGTTCCTGGTCCGGAAGGATTGGGTAATCATTTGAATTTTCTACGTAACTGGG
ATTGATCTTTGTAATTATTGATCATCTACGAGGAATTCCTTGTAAGCGTAGGTCATTACCCTATGCTGAATATGTCCCTG
CCCTTTGTACACACCGCCCGTCGCTCCTACCGATCGAATGATACGGTAAAGTTAACGGATCGTTTTATCTTTGGCAAC
ATTGATATAAACTAAAAGTTATTTAAATCTCATTGTTTAGAGGAAGGAGAAGTCGTAACAAGGTATCCGTAGGTGAAC
CTGCAGAAGGATCAA

>GQ496155.1 *Dictyostelium valdivianum*
AGATAGCCATGCATGTCTAAGTATAAATTCTTGTACGATGAAACTGCAGACGGCTCATTACAACAGTGATAAACTAAT
AGACTTTCGGGTTTTTAACCTTTTGGATAACCGCAGTAAATCGGGGCTAATACATAGAAGCGATGGGTGACTGGCAA
CGGAAGCTCAGCGATTATTAGCATTACTACCAATACCTTCGGGTCTTGTGGTGAAACCGAATAATATTGCAGATCGAG
GATTTATCTTCGACAAGTCTACTGTGTCACTGCCCTATCAACTTTCGATGGTACGGTATTGGCCTACCATGGTTGTAAC
GGGTAACGGGGAATTAGGGTTCGATTCCGGAGAGGGAGCCTGAGAAATGGCTACCACTTCTACGGAAGGCAGCAG
GCGCGCAAATTACTCAATCCCAATACGGGGAAGTAGTGACAATAAATATCAATACCTATCCTTAACGGAGGGCAATTG
AAATGAACACAAATTAAAACTCTTAATTAACACAATTGGAGGGCAAGTCTGGTGCCAGCAGCCGCGGTAATTCCAG
CTCCAATAGCATATACTAAAGTTGTTGCAGTTAAAAAGCTCGTAGTTGAAGTTAAAGGTTTACTGGGCTAAAGTTATT
TGCCGCTCTGGTGGTTAAATCAACTCCAGTATCTCTTTTTTTAATAGTTCAGCTTCTATTATCTTTGATAGTAGTTGTTT
GGACATTTCACTGTGAGAAAATTGTGGTGTTTAAAGCAGGCGTTTCGCCTGATCTTTTGCAGCATGGTATGATGGAA
CATGACATTTTACGCTATTGGTTTGCGTCTAAAGTGTAATGATTAATAGGGATGGATGGGGGTGTTCATATTGGTGGGC
GAGAGGTGAAATTCGTTGACCCTATCAAGATGAACTTCTGCGAAAGCATTCACCAAATACTTCCCCATTAATCAAGA
ACGAAAGTTTGGGGATCGAAGACGATCAGATACCGTCGTAGTCCAAACTATAAACTATGTCGACCAGGGATCGGTTA
AAATTTTTTTAAAATTTAATCGGCACCTTGTGAGAAATCACGAGTGTTTAGATTCCGGGGGGAGTATGGTCGCAAGT
CTGAAACTTAAAGGAATTGACGGAAGGGCACACAATGGAGTGGAGCCTGCGGCTTAATTTGACTCAACTCGGGAA
AACTTACCAAGCTAAGATATAGTAAGGATTGACAGACTAAAAGATCTTTCATGATTCTATAAGTGGTGGTGCATGGTC
GTTCTTAGTTGGTGGAGCGATTTGTCTGGTCAATTCCGATAACGGACGAGACCTCGACCTGCTAACTAGTAGTATTTA
TTAGTCAATATGGGCGATAGCTTCTCTGGGGTTTGGAATGACTTCGGTCATCTCCTGCTTCAAGGAGTGTGTAGTCTG
ACTTGATAGGTACGAATTAAAAAACTTCTTAGAGGGACTACCTGCCTCAAGCAGGCGGAAGTCCGAGGCAATAACA
GGTCTGTGATGCCCTTAGATACCTTGGGCCGCACGCGCGCTACAATGTAGAAAACAAAAAGGTTCCTGGTCCGGAA
GGATTGGGTAATCAATTGAATTTTCTACGTAACTGGGATTGATCTTTGTAATTATTGATCATCAACGAGGAATTCCTTG
TAAGCGTAGGTCATTACCCTATGCTGAATATGTCCCTGCCCTTTGTACACACCGCCCGTCGCTCCTACCGATCGAATG
ATACGGTAAAGTTAACGGATCGTTTTATCTGTGGCAACACTGATATAAACTAAAAGTTATTTAAATCTCATGTTAGAG
GAAGGAGAAGTC

>*Dictyostelium brevicaule* SMA
CGGGTAACGGGGAATTAGGGTTCGATTCCGGAGAGGGAGCCTGAGAAATGGCTACCACTTCTACGGAAGGCAGCA
GGCGCGCAAATTACTCAATCCCAATACGGGGAAGTAGTGACAATAAATATCAATACCTATCCTTAACGGAGGGCAATT
GAAATGAACACAAATTAAAACTCTTAATTAACACAATTGGAGGGCAAGTCTGGTGCCAGCAGCCGCGGTAATTCCA
GCTCCAATAGCATATACTAAAGTTGTTGCAGTTAAAAAGCTCGTAGTTGAAGTTAAAGGTTTACTGGGCTAAAGTTAT
TTGCCACTCTGGTGGTTAAATCAACTCCAGTATTCCTTTTTTTAATAGCTCTGCTTCTAGCATCTTTGATGTTAGTTGTT
TGGGCATTTCACTGTGAGAAAATTGTGGTGTTTAAAGCAGGCGTCTCGCCTGATCTTTTGCAGCATGGTATGATGAA
ACATGACATTTTACGCTATTGGTTTGCGTCTAAAGTGTAATGATTAATAGGGATGGATGGGGGTGT

>JX173877.1 *Polysphondylium fuscans* Sweden-11D
AACCTGGTTGATCCTGCCAGTAGTCATATGCTTGTCTCAAAGATTAAGCCATGCATGTCTAAGTATAAATTCTTGTACG
ATGAAACTGCAGACGGCTCATTACAACAGTGATAAACTAATAGACTTTCGGGTTTTACCTTTTGGATAACCGCAGTA
AATCGGGGCTAATACATACAAAGCGAGGGGTGATTGATTTATCAAGAGCTCCGCGATTATTAGCATTCAACCAATACCC
GCAAGGGTTCTGTTGGTGAAACCGAATAATATTGCAGATCGAAGATTTATCTTCGACAAGTCTACTGTGTCACTGCCC
TATCAACTTTCGATGGTACGGTATTGGCCTACCATGGTTGTAACGGGTAACGGGGAATTAGGGTTCGATTCCGGAGA
GGGAGCCTGAGAAATGGCTACCACTTCTACGGAAGGCAGCAGGCGCGCAAATTACTCAATCCCAATACGGGGAAGT
AGTGACAATAAATATCGATGCCTAACCATTTTTGGAAGGGTAATTGAAATGAACACAAATTAAAACTCTTAATTAATA
CAATTGGAGGGCAAGTCTGGTGCCAGCAGCCGCGGTAATTCCAGCTCCAATAGCATATACTAAAGTTGTTGCAGTTA

AAAAGCTCGTAGTTGAAGTTAAAGCTTTATTTGGGTTAAAAATTCATTTACCGTTTATTGGTTAAATCGAATTCCAGTA
TTGCTTTTTTAAAGTTCAGTTTGTATTGCCTTTGGTAGTATTTATTTGGACATTTCACTGTGAGAAAATTGTGGTGTTT
AAAGCAGGCGTCTCTGCCTGATCTTTTGCAGCATGGTATGATGGAACATGACATTTTGCGCAATTGGTTGCGATTAAA
GTGTAATGATTAATAGGGATGGATGGGGGTGTTCATATTGGTGGGCGAGAGGTGAAATTCGTTGACCCTATCAAGATG
AACTTCTGCGAAAGCATTCACCAAATACTTCCCCATTAATCAAGAACGAAAGTTTGGGGATCGAAGACGATCAGATA
CCGTCGTAGTCCAAACTATAAACTATGTCGACCAGGGATCGGTTAAAACTTTTTAAAAGTTTAATCGGCACCTTGTGA
GAAATCACGAGTGTTTAGATTCCGGGGGGAGTATGGTCGCAAGTCTGAAACTTAAAGGAATTGACGGAAGGGCACA
CAATGGAGTGGAGCCTGCGGCTTAATTTGACTCAACTCGGGAAAACTTACCAAGCTAAGATATAGTAAGGATTGACA
GACTAAGAGATCTTTCATGATTCTATAAGTGGTGGTGCATGGTCGTTCTTAGTTGGTGGAGCGATTTGTCTGGTCAAT
TCCGATAACGGACGAGACCTCGACCTGCTAACTAGTAGTATTTATTGGCCGTTATGGATGATAGTCATTCGGGGTTTG
GAAGGGCTTCGGTTCTGTCCGCTTCGTGTGGTGTGTAATCTGGTCGGATAGGTACGAATTAAAACTTCTTAGAGGGA
CTACCTGCCTCAAGCAGGCGGAAGTCCGAGGCAATAACAGGTCTGTGATGCCCTTAGATACCTTGGGCCGCACGCG
CGCTACAATGTAGAAAGCAAAAAGGTTCCTGGTCCGGAAGGATTGGGTAATCAATTGAATTTCTACGTAACTGGGA
TTGATCTTTGTAATTATTGATCATCAACGAGGAATTCCTTGTAAGCGTAAGTCATTACCTTATGCTGAATATGTCCCTG
CCCTTTGTACACACCGCCCGTCGCTCCTACCGATCGAATGATACGGTAAAGCCAACGGATAAGGTCCTATTGGGCAA
CCAGTATGGATTTTAAAAGTTGTTTAAATCTCATTGTTAGAGGAAGGAGAAGTCGTAACAAGGTATCGGTAGGTGAA
CCTGCAGAAGGATCA

>AM168046.1 *Polysphondylium laterosorum* AE4
AACCTGGTTGATCCTGCCAGTAGTCATATGCTTGTCTCAAAGATTAAGCCATGCATGTCTAAGTATAAATTCTTGTACG
ATGAAACTGCAGACGGCTCATTACAACAGTGATAAACTAATAGACTTTCGGGTTTTACCTTTTGGATAACCGCAGTA
AATCGGGGCTAATACATAGAAGCGAGGGGTGACTGATTTATCGGAAGCTCCGCGATTATTAGCATTCAACCAATACCC
GCAAGGGTTCTGTTGGTGAAACCGAATAATATTGCAGATCGAAGATTTATCTTCGACAAGTCTACTGTGTCACTGCCC
TATCAACTTTCGATGGTACGGTATTGGCCTACCATGGTTGTAACGGGTAACGGGGAATTAGGGTTCGATTCCGGAGA
GGGAGCCTGAGAAATGGCTACCACTTCTACGGAAGGCAGCAGGCGCGCAAATTACTCAATCCCAATACGGGGAAGT
AGTGACAATAAATATCGATGCCTATCCATTTATGGAAGGGTAATTGAAATGAACACAAATTAAAACTCTTAATTAATAC
AATTGGAGGGCAAGTCTGGTGCCAGCAGCCGCGGTAATTCCAGCTCCAATAGCATATACTAAAGTTGTTGCAGTTAA
AAAGCTCGTAGTTGAAGTTAAAGCTTTATTTGGGTTAAAAATTCATTTGCCTTTTTTTGGTTAAATAGAATTCCAGTAT
TGCTTTTTTAAAGTTCAGTTTGTAGTACTTTGTATTACTTATTTGGACATTTCACTGTGAGAAAATTGTGGTGTTTAAA
GCAGGCGTCTCTGCCTGATCTTTTGCAGCATGGTATGATGGAACATGACATTTTGCGCAATTGGTTGCGATTAAAGTG
TAATGATTAATAGGGATGGATGGGGGTGTTCATATTGGTGGGCGAGAGGTGAAATTCGTTGACCCTATCAAGATGAA
CTTCTGCGAAAGCATTCACCAAATACTTCCCCATTAATCAAGAACGAAAGTTTGGGGATCGAAGACGATCAGATACC
GTCGTAGTCCAAACTATAAACTATGTCGACCAGGGATCGGTTAAAACTTTTTAAAAGTTTAATCGGCACCTTGTGAG
AAATCACGAGTGTTTAGATTCCGGGGGGAGTATGGTCGCAAGTCTGAAACTTAAAGGAATTGACGGAAGGGCACAC
AATGGAGTGGAGCCTGCGGCTTAATTTGACTCAACTCGGGAAAACTTACCAAGCTAAGATATAGTAAGGATTGACAG
ACTAAGAGATCTTTCATGATTCTATAAGTGGTGGTGCATGGTCGTTCTTAGTTGGTGGAGCGATTTGTCTGGTCAATT
CCGATAACGGACGAGACCTCGACCTGCTAACTAGTAGTATTTATTGGCCGTTATGGATGATAGCTATTTCGGGGTTTG
GAAGGTCTTCGGATCTCTCCGCTTCGTGTGGTGTGTAATCTGGTCGGATAGGTACGAATTAAAACTTCTTAGAGGGA
CTACCTGCCTCAAGCAGGCGGAAGTCCGAGGCAATAACAGGTCTGTGATGCCCTTAGATACCTTGGGCCGCACGCG
CGCTACAATGTAGAAAGCAAAAAGGTTCCTGGTCCGGAAGGATTGGGTAATCAATTGAATTTCTACGTAACTGGGA
TTGATCTTTGTAATTATTGATCATCAACGAGGAATTCCTTGTAAGCGTAAGTCATTACCTTATGCTGAATATGTCCCTG
CCCTTTGTACACACCGCCCGTCGCTCCTACCGATCGAATGATACGGTAAAGCCAACGGATAAGATCCTATTGGGCAA
CTAATACGGATTTTAAAAGTTGTTTAAATCTCATTGTTTAGAGGAAGGAGAAGTCGTAACAAGGTATCCGTAGGTGA
ACCTGCAGAAGGATCAA

>GQ496156.1 *Polysphondylium patagonicum*
AGATTAGCCATGCATGTCTAAGTATAAATTCTTGTACGATGAAACTGCAGACGGCTCATTACAACAGTGATAAACTAA
TAGACTTTCGGGTTTTACCTTTTGGATAACCGCAGTAAATCGGGGCTAATACATACAAGCGAGGGGTGACTGATTTAT
CGGAAGCTCCGCGATTATTAGCATTCAACCAATACCCGCAAGGGTTCTGTTGGTGAAACCGAATAATATTGCAGATC
GAAGATTTATCTTCGACAAGTCTACTGTGTCACTGCCCTATCAACTTTCGATGGTACGGTATTGGCCTACCATGGTTGT
AACGGGTAACGGGGAATTAGGGTTCGATTCCGGAGAGGGAGCCTGAGAAATGGCTACCACTTCTACGGAAGGCAG
CAGGCGCGCAAATTACTCAATCCCAATACGGGGAAGTAGTGACAATAAATATCGATGCCTATCCATTTTGGAAGGGTA
ATTGAAATGAACACAAATTAAAACTCTTAATTAATACAATTGGAGGGCAAGTCTGGTGCCAGCAGCCGCGGTAATTC
CAGCTCCAATAGCATATACTAAAGTTGTTGCAGTTAAAAAGCTCGTAGTTGAAGTTAAAGCTTTATTTGGGTTAAAAA
TCCATTTTGCCTATACGGTTTAATAGGATTCCAGTATTGCTTTTTTAAAGTTCAGTTTGTATTGCCTTTGGTAGTATTTAT
TTGGACATTTCACTGTGAGAAAATTGTGGTGTTTAAAGCAGGCGTCTCTGTCTGATCTTTTGCAGCATGGTATGATGG
AACATGACATTTTGCGCAATTGGTTGCGATTAAAGTGTAATGATTAATAGGGATGGATGGGGGTGTTCATATTGGTGG
GCGAGAGGTGAAATTCGTTGACCCTATCAAGATGAACTTCTGCGAAAGCATTCACCAAATACTTCCCCATTAATCAA
GAACGAAAGTTTGGGGATCGAAGACGATCAGATACCGTCGTAGTCCAAACTATAAACTATGTCGACCAGGGATCGG
TTAAAACTTTTTAAAAGTTTAATCGGCACCTTGTGAGAAATCACGAGTGTTTAGATTCCGGGGGGAGTATGGTCGCA
AGTCTGAAACTTAAAGGAATTGACGGAAGGGCACACAATGGAGTGGAGCCTGCGGCTTAATTTGACTCAACTCGGG
AAAACTTACCAAGCTAAGATATAGTAAGGATTGACAGACTAAGAGATCTTTCATGATTCTATAAGTGGTGGTGCATGG
TCGTTCTTAGTTGGTGGAGCGATTTGTCTGGTCAATTCCGATAACGGACGAGACCTCGACCTGCTAACTAGTAGTATT
TATTGGCCGTTATGGATGATAGCTATTCGGGGTTTGGAAGGGTTTCGGCTCTCTCCGCTTCGTGTGGTGTGTAATCTG
GTCGGATAGGTACGAATTAAAACTTCTTAGAGGGACTACCTGCCTCAAGCAGGCGGAAGTCCGAGGCAATAACAGG
TCTGTGATGCCCTTAGATACCTTGGGCCGCACGCGCGCTACAATGTAGAAAGCAAAAAGGTTCCTGGTCCGGAAGG
ATTGGGTAATCAATTGAATTTCTACGTAACTGGGATTGATCTTTGTAATTATTGATCATCAACGAGGAATTCCTTGTA
AGCGTAAGTCATTACCTTATGCTGAATATGTCCCTGCCCTTTGTACACACCGCCCGTCGCTCCTACCGATCGAATGATA
CGGTAAAGCCAACGGATAAGATCCTATTGGGCAACTAATATGGATTTTAAAAGTTGTTTAAATCTCATTGTTAGAGGA
AGGAGAAGTC

>HQ141486.1 *Polysphondylium violaceum* 209
AACCTGGTTGATCCTGCCAGTAGTCATATGCTTGTCTCAAAGATTAAGCCATGCATGTCTAGTATAAATTCTTGTACGA
TGAAACTGCAGACGGCTCATTACAACAGTGATAAACTAATAGACTTTCGGGTTTTACCTTTTGGATAACCGCAGTAA
ATCGGGGCTAATACATAGAAGCGAGGGGTGACTGATTTATCGGAAGCTCCGCGATTATTAGCATTCAACCAATACCCG

CAAGGGTTCTGTTGGTGAAACCGAATAATATTGCAGATCGAAGATTTATCTTCGACAAGTCTACTGTGTCACTGCCCT
ATCAACTTTCGATGGTACGGTATTGGCCTACCATGGTTGTAACGGGTAACGGGGAATTAGGGTTCGATTCCGGAGAG
GGAGCCTGAGAAATGGCTACCACTTCTACGGAAGGCAGCAGGCGCGCAAATTACTCAATCCCAATACGGGGAAGTA
GTGACAATAAATATCGATGCCTATCCATTTTTGGAAGGGTAATTGAAATGAACACAAATTAAAACTCTTAATTAATAC
AATTGGAGGGCAAGTCTGGTGCCAGCAGCCGCGGTAATTCCAGCTCCAATAGCATATACTAAAGTTGTTGCAGTTAA
AAAGCTCGTAGTTGAAGTTAAAGCTTTATTTGGGTTAAAAATCCATTTGCCTTTTAACGGTTAAATTGGATTCCAGTA
TTGCTTTTTTAAAGTTCAGTTTGTATTGCCTTTGGTAGTATTTATTTGGACATTTCACTGTGAGAAAATTGTGGTGTTT
AAAGCAGGCGTCTCTGTCTGATCTTTTGCAGCATGGTATGATGGAACATGACATTTTGCGCAATTGGTTGCGATTAAA
GTGTAATGATTAATAGGGATGGATGGGGGTGTTCATATTGGTGGGCGAGAGGTGAAATTCGTTGACCCTATCAAGATG
AACTTCTGCGAAAGCATTCACCAAATACTTCCCCATTAATCAAGAACGAAAGTTTGGGGATCGAAGACGATCAGATA
CCGTCGTAGTCCAAACTATAAACTATGTCGACCAGGGATCGGTTAAAACTTTTTAAAAGTTTAATCGGCACCTTGTGA
GAAATCACGAGTGTTTAGATTCCGGGGGGAGTATGGTCGCAAGTCTGAAACTTAAAGGAATTGACGGAAGGGCACA
CAATGGAGTGGAGCCTGCGGCTTAATTTGACTCAACTCGGGAAAACTTACCAAGCTAAGATATAGTAAGGATTGACA
GACTAAGAGATCTTTCATGATTCTATAAGTGGTGGTGCATGGTCGTTCTTAGTTGGTGGAGCGATTTGTCTGGTCAAT
TCCGATAACGGACGAGACCTCGACCTGCTAACTAGTAGTATTTATTGGCCGTTATGGATGATAGCTATTCGGGGTTTG
GAAGGGCTTCGGTTCTCTCCGCTTCGTGTGGTGTGTAATCTGGTCGGATAGGTACGAATTAAAACTTCTTAGAGGGA
CTACCTGCCTCAAGCAGGCGGAAGTCCGAGGCAATAACAGGTCTGTGATGCCCTTAGATACCTTGGGCCGCACGCG
CGCTACAATGTAGAAAGCAAAAAGGTTCCTGGTCCGGAAGGATTGGGTAATCAATTGAATTTCTACGTAACTGGGA
TTGATCTTTGTAATTATTGATCATCAACGAGGAATTCCTTGTAAGCGTAAGTCATTACCTTATGCTGAATATGTCCCTG
CCCTTTGTACACACCGCCCGTCGCTCCTACCGATCGAATGATACGGTAAAGCCAACGGATAAGATCCTATTGGGCAA
CTAATATGGATTTAAAAGTTGTTTAAATCTCATTGTTTAGAGGAAGGAGAAGTCGTAACAAGGTATCCGTAGGTGAA
CCTGCAGAAGGATCA

>AM168108.1 *Polysphondylium violaceum* P6
AACCTGGTTGATCCTGCCAGTAGTCAAGTCTTGTCTCAAAGATTAAGCCATGCATGTCTAAGATAAATCTTGTACGAT
GAAACTGCAGACGGCTCATTACAACAGTGATAAACTAATAGACTTTCGGGTTTTACCNCNTTGGAAAACCGCAGTAA
ATCGGGGCTAATACATAGAAGCGAGGGGTGACTGATTTATCGGAAGCTCCGCGATTATTAGCATTCAACCAATACCCG
CAAGGGTTCTGTTGGTGAAACCGAATAATATTGCAGATCGAAGATTTATCTTCGACAAGTCTACTGTGTCACTGCCCT
ATCAACTTTCGATGGTACGGTATTGGCCTACCATGGTTGTAACGGGTAACGGGGAATTAGGGTTCGATTCCGGAGAG
GGAGCCTGAGAAATGGCTACCACTTCTACGGAAGGCAGCAGGCGCGCAAATTACTCAATCCCAATACGGGGAAGTA
GTGACAATAAATATCGATGCCTATCCATTTTTGGAAGGGTAATTGAAATGAACACAAATTAAAACTCTTAATTAATAC
AATTGGAGGGCAAGTCTGGTGCCAGCAGCCGCGGTAATTCCAGCTCCAATAGCATATACTAAAGTTGTTGCAGTTAA
AAAGCTCGTAGTTGAAGTTAAAGCTTTATTTGGGTTAAAAATCCATTTGCCTTTTAACGGTTAAATTGGATTCCAGTA
TTGCTTCTTTAAAGTTCAGTTTGTATTGCCTTTGGTAGTATTTATTTGGACATTTCACTGTGAGAAAATTGTGGTGTTT
AAAGCAGGCGTCTCTGTCTGATCTTTTGCAGCATGGTATGATGGAACATGACATTTTGCGCAATTGGTTGCGATTAAA
GTGTAATGATTAATAGGGATGGATGGGGGTGTTCATATTGGTGGGCGAGAGGTGAAATTCGTTGACCCTATCAAGATG
AACTTCTGCGAAAGCATTCACCAAATACTTCCCCATTAATCAAGAACGAAAGTTTGGGGATCGAAGACGATCAGATA
CCGTCGTAGTCCAAACTATAAACTATGTCGACCAGGGATCGGTTAAAACTTTTTAAAAGTTTAATCGGCACCTTGTGA
GAAATCACGAGTGTTTAGATTCCGGGGGGAGTATGGTCGCAAGTCTGAAACTTAAAGGAATTGACGGAAGGGCACA
CAATGGAGTGGAGCCTGCGGCTTAATTTGACTCAACTCGGGAAAACTTACCAAGCTAAGATATAGTAAGGATTGACA
GACTAAGAGATCTTTCATGATTCTATAAGTGGTGGTGCATGGTCGTTCTTAGTTGGTGGAGCGATTTGTCTGGTCAAT
TCCGATAACGGACGAGACCTCGACCTGCTAACTAGTAGTATTTATTGGCCGTTATGGATGATAGCTATTCGGGGTTTG
GAAGGGCTTCGGTTCTCTCCGCTTCGTGTGGTGTGTAATCTGGTCGGATAGGTACGAATTAAAACTTCTTAGAGGGA
CTACCTGCCTCAAGCAGGCGGAAGTCCGAGGCAATAACAGGTCTGTGATGCCCTTAGATACCTTGGGCCGCACGCG
CGCTACAATGTAGAAAGCAAAAAGGTTCCTGGTCCGGAAGGATTGGGTAATCAATTGAATTTCTACGTAACTGGGA
TTGATCTTTGTAATTATTGATCATCAACGAGGAATTCCTTGTAAGCGTAAGTCATTACCTTATGCTGAATATGTCCCTG
CCCTTTGTACACACCGCCCGTCGCTCCTACCGATCGAATGATACGGTAAAGCCAACGGATAAGATCCTATTGGGCAA
CTAATATGGATTTAAAAGTTGTTTAAATCTCATTGTTTAGAGGAAGGAGAAGTCGTAACAAGGTATCCGTAGGTGAA
CCTGCAGAAGGATCAA

>*Polysphondylium acuminatum* OH500 SML
CGGGTAACGGGGAATTAGGGTTCGATTCCGGAGAGGGAGCCTGAGAAATGGCTACCACTTCTACGGAAGGCAGCA
GGCGCGCAAATTACTCAATCCCAATACGGGGAAGTAGTGACAATAAATATCGATGCCTATCCATTTTTGGAAGGGTAA
TTGAAATGAACACAAATTAAAACTCTTAATTAATACAATTGGAGGGCAAGTCTGGTGCCAGCAGCCGCGGTAATTCC
AGCTCCAATAGCATATACTAAAGTTGTTGCAGTTAAAAAGCTCGTAGTTGAAGTTAAAGCTTTATTTGGGTTAAAAAT
CCATTTACCTTTATGGCTAAATAGGATTCCAGTATTGCTTTTTTAAAGTTCAGTTTGTATTACTTTGGTAGTACTCATTT
GGACATTTCACTGTGAGAAAATTGTGGTGTTTAAAGCAGGCGTATCTGCCTGATCTTTTGCAGCATGGTATGATGGA
ACATGACATTTTGCGCAATTGGTTGCGATTAAAGTGTAATGATTAATAGGGATGGATGGGGGTGT

>HQ141488.1 *Coremiostelium polycephalum* Landolt #1130 SS3B
AACCTGGTTGATCCTGCCAGTAGTCATATGCTTGTCTCAAGGATTAAGCCATGCATGTCTAAGTATAAATCTTTATACG
ATGAAACTGCAGACGGCTCATTACAACAGTGATAAACTACTAGACTTTCGGGTTTCACGACCTTTTGGATAACCGCA
GTAAATCGGGGCTAATACATGTAAACGAGGGGTGACTGGGCAACTGGAAGCTCCGCGATTATTAGCTTTTTACCAAT
CCCCGCAAGGGGTCAGTTGGTGAGACCAAATAATGCTGCCGATCGAGATCTAATCTCGACAAGTCTACTGTGTCACT
GCCCTATCAACTTTCGATGGTACGGTATTGGCCTACCATGGTTGTAACGGGTAACGGGGAATCAGGGTTCGATTCCGG
AGAGGGAGCCTGAGAAATGGCTACCACTTCTATGGAAGGCAGCAGGCGCGCAAATTACTCAATCCCAATACGGGGA
AGTAGTGACAATAAATATCAATGCTCATCCTGATAAAGGAGGGTAATTGAAATGAACACAAACTAAACCTCTTAATTA
ATACAATTGGAGGGCAAGTCTGGTGCCAGCAGCCGCGGTAATTCCAGCTCCAATAGCATATACTAAAGTTGTTGCAG
TTAAAAAGCTCGTAGTTTAAGTAAAGGTCTGGGGGGGCTCAATATTCCCTATTGGGTCTCGCATTCAATACGGGGATC
CCCTCAGAACTTTTTAAACTGCCCATTTCAGGCGCTCGCGCTCTGACTTCGGGTGATTCACTGTGAGAAAATTGTGG
TGTTCAAAGCAGGCGTCTCGCCTGATCCTTTGCAGCATGGTATGATAGAACACGACACCTAACGCCACATTGGTTGC
GATTAAGTGTAATGATTAATAGGGATGGATGGGGATGTTCATATTGGTGGGCGAGAGGTGAAATTCGTTGACCCTATC
AAGATGAACTTCTGCGAAAGCATTCATCAAATACTTCTCCATTAATCAAGAACGAAAGTTTGGGGATCGAAGACGAT
CAGATACCGTCGTAGTCCAAACTATAAACTATGTCGACCAGGGATCGGCTGGAGTTCTTTAAAAATCCAGTCGGCAC
CTTGTGAGAAATCACGAGTGTTTAGATTCCGGGGGGAGTATGGTCGCAAGTCTGAAACTTAAAGGAATTGACGGAA

GGGCACACAATGGAGTGGAGCCTGCGGCTTAATTTGACTCAACTCGGGAAAACTTACCAAGCTAAGATATAGCTAG
GATTGACAGACTAAAAGATCTTTCATGATTCTATAAGTGGTGGTGCATGGTCGTTCTTAGTTGGTGGAGCGATTTGTC
TGGTCAATTCCGATAACGGACGAGACCTCGACCTGCTAACTAGTGGTGTCTATCCTATCGCCATGGGTGATAGCCAG
ATGGGTGTGTTTTCGTTCAGCAATGAGCGGGATCCCTCGCCTGGTGTGTAGCCTGATTGGATAGGCATCCAAAATCA
AAAACTTCTTAGAGGGACTACCTGCCTCAAGCAGGCGGAAGTCCGAGGCAATAACAGGTCTGTGATGCCCTTAGAT
ACCTTGGGCCGCACGCGCGCTACATTGCGATGCGCAAAAAGGTTGCCTGGTCCGGAAGGATTGGGTAATCAGGAAT
TCATCACGTAACAGGGATTGATCTTTGTAATTATCGATCATCAACGAGGAATTCCTTGTAAGCGTAAGTCATTACCTTA
CGCTGAATATGTCCCTGCCCTTTGTACACACCGCCCGTCGCTCCTACCGATCGAATGATACGGTAAAACCAACGGCAT
TGCTGGCTGGCTTAACCTCTGGTCTAGCGATAATGTTGTTTAAACCTCATTGTTAGAGGAAGGAGAAGTCGTAACAA
GGTATCCGTAGGTGAACCTGCAGAAGGATCA

>HQ141489.1 *Coremiostelium polycephalum* Landolt #2132 B-9c
AACCTGGTTGATCCTGCCAGTAGTCATATGCTTGTCTCAAAGATTAAGCCATGCATGTCTAAGTATAAATCTTTATACG
ATGAAACTGCAGACGGCTCATTACAACAGTGATAAACTACTAGACTTTCGGGTTTCACGACCTTTTGGATAACCGCA
GTAAATCGGGGCTAATACATGTAAACGAGGGGTGACTGGGCAACTGGAAGCTCCGCGATTATTAGCTTTTTACCAAT
CCCCGCAAGGGGTCAGTTGGTGAGACCAAATAATGCTGCCGATCGAGATTTAATCTCGACAAGTCTACTGTGTCACT
GCCCTATCAACTTTCGATGGTACGGTATTGGCCTACCATGGTTGTAACGGGTAACGGGGAATCAGGGTTCGATTCCGG
AGAGGGAGCCTGAGAAATGGCTACCACTTCTATGGAAGGCAGCAGGCGCGCAAATTACTCAATCCCAATACGGGGA
AGTAGTGACAATAAATATCAATGCTCATCCTGACAAAGGAGGGTAATTGAAATGAACACAAACTAAACCTCTTAATT
AATACAATTGGAGGGCAAGTCTGGTGCCAGCAGCCGCGGTAATTCCAGCTCCAATAGCATATACTAAAGTTGTTGCA
GTTAAAAAGCTCGTAGTTTAAGTAAAGGTCTAGGGGGGCTCAATATTCCCTATTGGGTCTCGCATTCAATATGGGGAT
CCCCTTAGAACTTTTTAAACTGCCCATTTCAGGCGTCCCGCAAGGGCTCCGTCTGGCTTCGGGTGATTCACTGTGAG
AAAATTGTGGTGTTCAAAGCAGGCGTCTCGCCTGATCCTTTGCAGCATGGTATGATAAAACACGACACCTAACGCCA
CATTGGTTGCGATTAAGTGTAATGATTAATAGGGATGGATGGGGATGTTCATATTGGTGGGCGAGAGGTGAAATTCGT
TGACCCTATCAAGATGAACTTCTGCGAAAGCATTCATCAAATACTTCTCCATTAATCAAGAACGAAAGTTTGGGGAT
CGAAGACGATCAGATACCGTCGTAGTCCAAACTATAAACTATGTCGACCAGGGATCGGCTGGAGTTCTTTAAAAATC
CAGTCGGCACCTTGTGAGAAATCACGAGTGTTTAGATTCCGGGGGGAGTATGGTCGCAAGTCTGAAACTTAAAGGA
ATTGACGGAAGGGCACACAATGGAGTGGAGCCTGCGGCTTAATTTGACTCAACTCGGGAAAACTTACCAAGCTAAG
ATATAGCTAGGATTGACAGACTAAAAGATCTTTCATGATTCTATAAGTGGTGGTGCATGGTCGTTCTTAGTTGGTGGA
GCGATTTGTCTGGTCAATTCCGATAACGGACGAGACCTCGACCTGCTAACTAGTGGTGTCTATCCTATCGCCATGGGT
GATAGTTAGCTGGGTGTCTGGCTCGCTCGCAAGAGCGGGTCTGCCTCGGCTGGCGTGTAGCCTGATTGGATAGGCAT
CCAAAAACAAAAAACTTCTTAGAGGGACTACCTGCCTCAAGCAGGCGGAAGTCCGAGGCAATAACAGGTCTGTGA
TGCCCTTAGATACCTTGGGCCGCACGCGCGCTACATTGCGATGCGCAAAAAGGTTGCCTGGTCCGGAAGGATTGGG
TAATCAGGAATTCATCACGTAACAGGGATTGATCTTTGTAATTATCGATCATCAACGAGGAATTCCTTGTAAGCGTAA
GTCATTACCTTACGCTGAATATGTCCCTGCCCTTTGTACACACCGCCCGTCGCTCCTACCGATCGAATGATACGGTAA
AACCAACGGCATTGCTGGCTGGCTTAAACTCTGGTCTAGCGATAATGTTGTTTAAACCTCATTGTTAGAGGAAGGAG
AAGTCGTAACAAGGTATCCGTAGGTGAACCTGCAGAAGGATCA

>HQ141490.1 *Coremiostelium polycephalum* Landolt #1675 GUAM
AACCTGGTTGATCCTGCCAGTAGTCATATGCTTGTCTCAAAGATTAAGCCATGCATGTCTAAGTATAAATCTTTATACG
ATGAAACTGCAGACGGCTCATTACAACAGTGATAAACTACTAGACTTTCGGGTTTCACGACCTTTTGGATAACCGCA
GTAAATCGGGGCTAATACATATAAACGAAGGGCGACTGGGCAACTGGAAGCTCTGCGATTATTAGCTTTTTACCAATC
CCCGCAAGGGGCCAGTTGGTGAGACCAAATAATGCTGCCGATCGAGATCTAATCTCGACAAGTCTATTGTGTCACTG
CCCTATCAACTTTCGATGGTACGGTATTGGCCTACCATGGTTGTAACGGGTAACGGGGAATCAGGGTTCGATTCCGGA
GAGGGAGCCTGAGAAATGGCTACCACTTCTATGGAAGGCAGCAGGCGCGCAAATTACTCAATCCCAATACGGGGAA
GTAGTGACAATAAATATCAATGCTCATCCTGATAAAGGAGGGCAATTGAAATGAACACAAACTAAACCTCTTAATTA
ATACAATTGGAGGGCAAGTCTGGTGCCAGCAGCCGCGGTAATTCCAGCTCCAATAGCATATACTAAAGTTGTTGCAG
TTAAAAAGCTCGTAGTTTAAACTAAGGTCTAGGAGGGCTCAATATCACCTATCGGGCCTTGCGTTCAATAGGGGATC
CTCTTAGAACTTTTTAAACTGCCCATTCCTGGCTAGCAATAGCCCGGTTTAGGGTGATTCACTGTGAGAAAATTGTGG
TGTTCAAAGCAGGCGTCTCGCCTGATCCTTTGCAGCATGGTATGATAAAACACGACACCTAACGCCATATTGGTTGC
GATATTAAGTGTAATGATTAATAGGGATGGATGGGGATGTTCATATTGGTGGGCGAGAGGTGAAATTCGTTGACCCTA
TCAAGATGAACTTCTGCGAAAGCATTCATCAAATACTTCCCCATTAATCAAGAACGAAAGTTTGGGGATCGAAGACG
ATCAGATACCGTCGTAGTCCAAACTATAAACTATGTCGACCAGGGATCGGCTGGAGTTTTTTAAAAATCCAGCCGGC
ATCTTGTGAGAAATCACGAGTGTTTAGATTCCGGGGGGAGTATGGTCGCAAGTCTGAAACTTAAAGGAATTGACGG
AAGGGCACACAATGGAGTGGAGCCTGCGGCTTAATTTGACTCAACTCGGGAAAACTTACCAAGCTAAGATATAGCT
ATGATTGACAGACTAAAAGATCTTTCATGATTCTATAAGTGGTGGTGCATGGTCGTTCTTAGTTGGTGGAGCGATTTG
TCTGGTCAATTCCGATAACGGACGAGACCTCGACCTGCTAACTAGTGGTGCCTATCCTATCGCCATGGGTGATAGGTA
GTTGGGTGTGCCGGTTCCAGCAATGGTGCCGACCCTCGGCTATTGTGTAGCCTGATTGGATAGGCATCCAAATTCAA
AAAACTTCTTAGAGGGACTACCTGCCTCAAGCAGGCGGAAGTCCGAGGCAATAACAGGTCTGTGATGCCCTTAGAT
ACCTTGGGCCGCACGCGCGCTACATTGCGATGCGCAAAAAGGTTGCCTGGTCCGGAAGGATTGGGTAATCAGGAAT
TCATCACGTAACAGGGATTGATCTTTGTAATTATCGATCATCAACGAGGAATTCCTTGTAAGCGTAAGTCATTACCTTA
TGCTGAATATGTCCCTGCCCTTTGTACACACCGCCCGTCGCTCCTACCGATCGAATGATACGGTAAAACCAACGGCAT
TGTTGGCTTGCGTAAAACCAGGTCAGCGATAATGTTGTTTAAACCTCATTGTTAGAGGAAGGAGAAGTCGTAACAA
GGTATCCGTAGGTGAACCTGCAGAAGGATCA

>AM168056.1 *Coremiostelium polycephalum* MY1-1
AACCTGGTTGATCCTGCCAGTAGTCATATGCTTGTCTCAAAGATTAAGCCATGCATGTCTAAGTATAAATCTTTATACG
ATGAAACTGCAGACGGCTCATTACAACAGTGATAAACTACTAGACTTTCGGGTTTCACGACCTTTTGGATAACCGCA
GTAAATCGGGGCTAATACATGTAAACGAGGGGCGACTGGGCAACTAGAAACTCCGCGATTATTAGCTTTTTACCAAT
CCCCGCAAGGGGCTAGTTGGTGAAACCAAATAATGCTGCCGATCGAGATTTAATCTCGACAAGTCTATTGTGTCACT
GCCCTATCAACTTTCGATGGTACGGTATTGGCCTACCATGGTTGTAACGGGTAACGGGGAATCAGGGTTCGATTCCGG
AGAGGGAGCCTGAGAAATGGCTACCACTTCTATGGAAGGCAGCAGGCGCGCAAATTACTCAATCCCAATACGGGGA
AGTAGTGACAATAAATATCAATGCTCATCCTGACAAAGGAGGGTAATTGAAATGAACACAAACTAAACCTCTTAATT
AATACAATTGGAGGGCAAGTCTGGTGCCAGCAGCCGCGGTAATTCCAGCTCCAATAGCATATACTAAAGTTGTTGCA
GTTAAAAAGCTCGTAGTTTAAATAAAGGTCTAGGGGGGCTCAATATTTCCTATTGGGTCTTGCATTCAATATGGGAAT

CCCCTTAGAACTTTTTAAACTGCCCATTTCAGCCGGGCAACCGGTTGTCTTCGGGTGATTCACTGTGAGAAAATTGT
GGTGTTCAAAGCAGGCGTCTCGCCTGATCCTTTGCAGCATGGTATGATAGAACACGACACATAACGCCACATTGGTT
GCGATTAAGTGTAATGATTAATAGGGATGGATGGGGATGTTCATATTGGTGGGCGAGAGGTGAAATTCGTTGACCCTA
TCAAGATGAACTTCTGCGAAAGCATTCATCAAATACTTCTCCATTAATCAAGAACGAAAGTTTGGGGATCGAAGACG
ATCAGATACCGTCGTAGTCCAAACTATAAACTATGTCGACCAGGGATCGGCTGGAGTTCTTTAAAAATCCAGTCGGC
ACCTTGTGAGAAATCACGAGTGTTTAGATTCCGGGGGGAGTATGGTCGCAAGTCTGAAACTTAAAGGAATTGACGG
AAGGGCACACAATGGAGTGGAGCCTGCGGCTTAATTTGACTCAACTCGGGAAAACTTACCAAGCTAAGATATAGCT
AGGATTGACAGACTAAAAGATCTTTCATGATTCTATAAGTGGTGGTGCATGGTCGTTCTTAGTTGGTGGAGCGATTTG
TCTGGTCAATTCCGATAACGGACGAGACCTCGACCTGCTAACTAGTGGTGTCTATCCTATCGCCATGGGTGATAGCTA
GGTGGGTGTGTTGTAGACGGGCAACCGTTTGCTTCCCTCGCCTGGTGTGTAGCCTGATTGGATAGGCATCCAAAAAC
AAAATACTTCTTAGAGGGACTACCTGCCTCAAGCAGGCGGAAGTCCGAGGCAATAACAGGTCTGTGATGCCCTTAG
ATACCTTGGGCCGCACGCGCGCTACATTGCGATGCGCAAAAAGGTTGCCTGGTCCGGAAGGATTGGGTAATCAGGA
ATTCATCACGTAACAGGGATTGATCTTTGTAATTATCGATCATCAACGAGGAATTCCTTGTAAGCGTAAGTCATTACCT
TACGCTGAATATGTCCCTGCCCTTTGTACACACCGCCCGTCGCTCCTACCGATCGAATGATACGGTAAAACCAACGGC
ATCGTTGGCTGGTTTAATCGCTAGTCTGTGATAATGTTGTTTAAACCTCATTGTTTAGAGGAAGGAGAAGTCGTAACA
AGGTATCCGTAGGTGAACCTGCAGAAGGATCAA

>AM168057.1 *Synstelium polycarpum* VE1b
AACCTGGTTGATCCTGCCAGTAGTCATATGCTTGTCTCAAAGATTAAGCCATGCATGTCTAAGTATAAATCTTTGTACG
GTGAAACTGCAGACGGCTCATTATAACAGTGATAAACTACAGAACTTTCGCGTTAATTCGTTATGGATAACCGCAGTA
AATCGGGGCTAATACATACAAACGAAAGGCGAGCGGGCAACCGTGAGTCCTTGCGATTATTAACCATTCTAACCAAT
CTCTTCGGAGTTTGTGGCGAAACCGAATAATATTGCTGATCGAAACTAGTTTCGACAAGTTCTATGTGTCACTGCCCT
ATCAACTTTCGATGGTACGGTATTGGCCTACCATGGTTGTAACGGGTAACGGGGAATTAGGGTTCGATTCCGGAGAG
GGCGCCTGAGAAATGGCGACCACTTCTACGGAAGGCAGCAGGCGCGCAAATTACTCAATCCCGACACGGGGAAGT
AGTGACAATAAATATTAATGCCTATCAGTTTTACTGAGGGCAATTGAAATAAGTACAATCTAAATCGCTTAACCAATTT
AATTGGAGGGCAAGTCTGGTGCCAGCAGCCGCGGTAATTCCAGCTCCAATAGCGTATACTAAATTTGTTGCAGTTAA
AAAGCTCGTAGTTAAAGTTGAAATTCTTCGGATAAAACTTGAGTAGCATCATTATTGGTGTTTACTCTTGCCGTTTGT
TTCTTTTTATTCAACTCCTCGTCTAACGTTGGATGTTTGAATTCTCCACTGTGAGAAAAATGTAGTGTTTAAAGCAGG
CGATTAGTCTGATCTTTTGCAGCATGGTATGGTAAAATAAGATACTAAACACTTATTGGTTGTGTTGTAGTATAATGAT
TAATAGGGAAGGGCGGGGCCGTTCATATTGATGGGCGAGAGGTGAAATTCGTTGACCCTATCAAGATGCACTACAGC
GGAAGCATTCGGCAAGTACTTCTCCATTAATCAAGAACGAAAGTTTGGGGATCGAAGACGATCAGATACCGTCGTAG
TCCAAACCATAAACTATGTCGACCAGGGATTGGATGGGTAATTTTTAATAAACCTATTCAGAACCTTGTGGGAAACCA
TGAGTGTTTGGACTCTGGGGGGAGTATGGTCGCAAGTCTGAAACTTAAAGGAATTGACGGAAGGGCACACAATGG
AGTGGAACCTGCGGCTTAATTTGACTCAACTCGGGAAAACTTACCAAGCTAAGATATAGCAAGGATTGACAGACTA
AAAGATTTTTCATGATTCTATAAGTGGTGGTGCATGGTCGTTCTTAGTTGGTGGAGTGATTTGTCTGGTCAATTCCGAT
AACGGACGAGACCTCGACCTACTAAATAGTGGTGTGTATTTGGTCAATATGGGTGATAGAGTTATGGCAATTTTGCAT
TGATTTCGGTTGGTGTAACGTTGACGTGGCTTGTGTAATCTGATCATTTAGACACTAAAAAATTAAAATACTTCTTAG
AGGGACTACCTACCTCAAGTGGGGGGAAGTTCGAGGCAATAACAGGTCTGTGATGCCCTTAGATACCTTGGGCTGC
ACGCGCGTTACAATGTAAAGTGGAAAAAGGTTCCTGGACCGAAAGGTTTGGGTAATCATTTGAATACTCTACGTAAT
GGGGATTGTTCTTTGTAATTATCGAACATCAACGAGGAATTCCTTGTAAGCGTAAATCATTACTTTACGCTGAATATGT
CCCTGCCCTTTGTACACACCGCCCGTCGCTCCTACCGATCGAACGATACGGTAAAATCGACGGATCGTTCTTAATTCC
GAAAGGAAACCTAGAATGAAAAGTTGTTTAAATCTCATTGTTTAGAGGAAGGAGAAGTCGTAACAAGGTATCCGTA
GGTGAACCTGCAGAAGGATCAA

>AM168058.1 *Synstelium polycarpum* OhioWILDS
AACCTGGTTGATCCTGCCAGTAGTCATATGCTTGTCTCAAAGATTAAGCCATGCATGTCTAAGTATAAATCTTTGTACG
GTGAAACTGCAGACGGCTCATTACAACAGTGATAAACTACAGAACTTTCGCGATTTATCGTTATGGGTAACCGCAGT
AAATCGGGGCTAATACATACAAACGGAAGGCGAGCGGGCAGCTGCGAGTCCTTGCGATTATTAACCATTCTAACCAA
TCTCTTCGGAGTTTGTGGCGAAACCGAATAATATTGCTGATCGAATTTTAATTCGACAAGTTCTATGTGTCACTGCCC
TATCAACTTTCGATGGTACGGTATTGGCCTACCATGGTTGTAACGGGTAACGGGGAATTAGGGTTCGATTCCGGAGA
GGGCGCCTGAGAAATGGCGACCACTTCTACGGAAGGCAGCAGGCGCGCAAATTACTCAATCCCAACACGGGGAAG
TAGTGACAATAAATATTAATGCCTATCAGTTTTACTGAGGGCAATTGAAATAAGTACAATTTAAATCGCTTAACCAATT
TAATTGGAGGGCAAGTCTGGTGCCAGCAGCCGCGGTAATTCCAGCTCCAATAGCGTATACTAAATTTGTTGCAGTTA
AAAAGCTCGTAGTTAAAATTGAAATTATATGGATAAAACTTGATTAGCATCATTCGTGGTGTTTTATTCTTGCCATTAA
TTTCTTATGATTCAACTCCTCGCCTAGCGTTGGATGTTTGAATTCTCCACTGTGAGAAAAATGTAGTGTTTAAAGCAG
GCGATTAGTCTGATCTTTTGCAGCATGGTATGGTAAAATAAGATACTAAACACTCGTTGGTTGTGTTCTGGTATAATGA
TTAATAGGGAAGGGCGGGGCCGTTCATATTGATGGGCGAGAGGTGAAATTCGTTGACCCTATCAAGATGCACTACAG
CGAAAGCATTCGGCAAGTACTTCTCCATTAATCAAGAACGAAAGTTTGGGGATCGAAGACGATCAGATACCGTCGTA
GTCCAAACCATAAACTATGTCGACCAGGGATTGGGTGGGTAATTTTTAATAAACCTATTCAGAACCTTGTGGGAAAC
CATGAGTGTTTGGACTCTGGGGGGAGTATGGTCGCAAGTCTGAAACTTAAAGGAATTGACGGAAGGGCACACAGTG
GAGTGGAACCTGCGGCTTAATTTGACTCAACTCGGGAAAACTTACCAAGCTAAGATATAGCAAGGATTGACAGACT
AAAAGATTTTTCATGATTCTATAAGTGGTGGTGCATGGTCGTTCTTAGTTGGTGGAGTGATTTGTCTGGTCAATTCCG
ATAACGGACGAGACCTCGACCTACTAAATAGTGGTGCGTATTTGGTCAATATGGGTGATAGTATTATGGCAGTGCTGC
AGCGTTTTCGGATGTTGTAGTATTGACATAGTATGTGTAATTCGATCATTTAGGCACTAAAAAATTAAAATACTTCTTA
GAGGGACTACCTACCTCAAGTGGGGGGAAGTTCGAGGCAATAACAGGTCTGTGATGCCCTTAGATACCTTGGGCTG
CACGCGCGTTACAATGTAAGGTGGAAAAAGGTTCCTGGACCGAAAGGTTTGGGTAATCATTTGAATACCCTACGTAA
TGGGGATTGTTCTTTGTAATTATCGAACATCAACGAGGAATTCCTTGTAAGCGTAAATCATTACTTTACGCTGAATATG
TCCCTGCCCTTTGTACACACCGCCCGTCGCTCCTACCGATCGAACGATACGGTAAAATTGACAGATTGTTTTTAATTC
CGAAAGGAAAGTCTAAGACAAAAAGTTATTTAAATCTCATTGTTTAGAGGAAGGAGAAGTCGTAACAAGGTATCCG
TAGGTGAACCTGCAGAAGGATCAA

>OR294048 *Polysphondylium fuscans* A241
GGGCATCTTGTCTCAAGATTAGCCATGCATGTCTAAGTATAAATTCTTGTACGATGAAACTGCAGACGGCTCATTACA
ACAGTGATAAACTAATAGACTTTCGGGTTTTACCTTTTGGATAACCGCAGTAAATCGGGGCTAATACATAGAAGCGAG
GGGTGATTGATTTATCAAGAGCTCCGCGATTATTAGCATTCAACCAATACCCGCAAGGGTTCTGTTGGTGAAACCGA

ATAATATTGCAGATCAAAGATTTATCTTTGACAAGTCTACTGTGTCACTGCCCTATCAACTTTCGATGGTACGGTATTG
GCCTACCATGGTTGTAACGGGTAACGGGGAATTAGGGTTCGATTCCGGAGAGGGAGCCTGAGAAATGGCTACCACT
TCTACGGAAGGCAGCAGGCGCGCAAATTACTCAATCCCAATACGGGGAAGTAGTGACAATAAATATCGATGCCTAAC
CATTTTTGGAAGGGTAATTGAAATGAACACAAATTAAAACTCTTAATTAATACAATTGGAGGGCAAGTCTGGTGCCA
GCAGCCGCGGTAATTCCAGCTCCAATAGCATATACTAAAGTTGTTGCAGTTAAAAAGCTCGTAGTTGAAGTTAAAGC
TTTATTTGGGTTAAAAATTCATTTACCTTTTATTGGTTAAATTGGATTCCAGTATTGCTTTTTTAAAGTTCAGTTTGTATT
GCCTTTGGTAGTATTTATTTGGACATTTCACTGTGAGAAAATTGTGGTGTTTAAAGCATGCGTCTCTGCCTGATCTTTT
GCAGCATGGTATGATGGAACATGACATTTTGCGCAATTGGTTGCGATTAAAGTGTAATGATTAATAGGGATGGATGGG
GGGTGTTCATATTGGTGGGCGAGAGGTGAAATTCGTTGACCCTATCAAGATGAACTTCTGCGAAAGCATTCACCAAA
TACTTCCCCATTAATCAAGAACGAAAGTTTGGGGATCGAAGACGATCAGATACCGTCGTAGTCCAAACTATAAACTA
TGTCGACCAGGGATCGGTTAAAACTTTTTAAAAGTTTAATCGGCACCTTGTGAGAAATCACGAGTGTTTAGATTCCG
GGGGGAGTATGGTCGCAAGTCTGAAACTTAAAGGAATTGACGGAAGGGCACACAATGGAGTGGAGCCTGCGGCTT
AATTTGACTCAACTCGGGAAAACTTACCAAGCTAAGATATAGTAAGGATTGACAGACTAAGAGATCTTTCATGATTCT
ATAAGTGGTGGTGCATGGTCGTTCTTAGTTGGTGGAGCGATTTGTCTGGTCAATTCCGATAACGGACGAGACCTCGA
CCTGCTAACTAGTAGTATTTATTGGCCGTTATGGATGATAGCTATTTCGGGGTTTGGAAGGTCTTCGGATCTCTCCGCT
TCGTGTGGTGTGTAATCTGGTCGGATAGGTACGAATTAAAACTTCTTAGAGGGACTACCTGCCTCAAGCAGGCGGAA
GTCCGAGGCAATAACAGGTCTGTGATGCCCTTAGATACCTTGGGCCGCACGCGCGCTACAATGTAGAAAGCAAAAA
GGTTCCTGGTCCGGAAGGATTGGGTAATCAATTGAATTTTCTACGTAACTGGGATTGATCTTTGTAATTATTGATCATC
AACGAGGAATTCCTTGTAAGCGTAAGTCATTACCTTATGCTGAATATGTCCCTGCCCTTTGTACACACCGCCCGTCGC
TCCTACCGATCGAATGATACGGTAAAGCCAACGGATAAGGTCCTATTGGGCAACCAGTACGGATTTTAAAAGTTGTT
TAAATCTCATTGTTAGAGGAAGGAGAAGTCTACAATTTCCCC

>**OR294050** ***Dictyostelium robusticaule*** **B341**
GTGGGCTGTCTAAGATTAAGCCATGCATGTCTAAGTATAAATTCTTGTACGATGAAACTGCAGACGGCTCATTACAAC
AGTGATAAACTAATAGACTTTCGGGTTTTTAACCTTTTGGATAACCGCAGTAAATCGGGGCTAATACATAGAAGCGAT
GGGTGACTGGCAACGGAAGCTCAGCGATTATTAGCATTACTACCAATACCTTCGGGTCTTGTGGTGAAACCGAATAA
TATTGCAGATCGAGGATTTATCTTCGACAAGTCTACTGTGTCACTGCCCTATCAACTTTCGATGGTACGGTATTGGCCT
ACCATGGTTGTAACGGGTAACGGGGAATTAGGGTTCGATTCCGGAGAGGGAGCCTGAGAAATGGCTACCACTTCTA
CGGAAGGCAGCAGGCGCGCAAATTACTCAATCCCAATACGGGGAAGTAGTGACAATAAATATCAATACCTATCCTTA
ACGGAGGGCAATTGAAATGAACACAAATTAAAACTCTTAATTAACACAATTGGAGGGCAAGTCTGGTGCCAGCAGC
CGCGGTAATTCCAGCTCCAATAGCATATACTAAAGTTGTTGCAGTTAAAAAGCTCGTAGTTGAAGTTAAAGGTTTACT
GGGCTAAAGTTATTTGCCGCTCTGGTGGTTAAATCAACTCCAGTATCTCTTTTTTTAATAGTTCAGCTTCTATTATCTTT
GATAGTAGTTGTTTGGACATTTCACTGTGAGAAAATTGTGGTGTTTAAAGCAGGCGTTTCGCCTGATCTTTTGCAGC
ATGGTATGATGAAACATGACATTTTACGCTATTGGTTTGCGTCTAAAGTGTAATGATTAATAGGGATGGATGGGGGTG
TTCATATTGGTGGGCGAGAGGTGAAATTCGTTGACCCTATCAAGATGAACTTCTGCGAAAGCATTCACCAAATACTT
CCCCCATTAATCAAGAACGAAAGTTTGGGGATCGAAGACGATCAGATACCGTCGTAGTCCAAACTATAAACTATGTC
GACCAGGGATCGGTTAAAATTTTTTTAAAATTTAATCGGCACCTTGTGAGAAATCACGAGTGTTTAGATTCCGGGGG
GGAGTATGGTCGCAAGTCTGAAACTTAAAGGAATTGACGGAAGGGCACACAATGGAGTGGAGCCTGCGGCTTAATT
TGACTCAACTCGGGAAAACTTACCAAGCTAAGATATAGTAAGGATTGACAGACTAAAAGATCTTTCATGATTCTATAA
GTGGTGGTGCATGGTCGTTCTTAGTTGGTGGAGCGATTTGTCTGGTCAATTCCGATAACGGACGAGACCTCGACCTG
CTAACTAGTAGTATTTATTAGTCAATATGGGCGATAGCTTCTCTGGGGTTTGGAATGGTTTCGGCCATCTCCTGCTTCA
AGGAGTGTGTAGTCTGACTTGATAGGTACGAATTAAAAAACTTCTTAGAGGGACTACCTGCCTCAAGCAGGCGGAA
GTCCGAGGCAATAACAGGTCTGTGATGCCCTTAGATACCTTGGGCCGCACGCGCGCTACAATGTAGAAAACAAAAA
GGTTCCTGGTCCGGAAGGATTGGGTAATCATTTGAATTTTCTACGTAACTGGGATTGATCTTTGTAATTATTGATCATC
AACGAGGAATTCCTTGTAAGCGTAGGTCATTACCCTATGCTGAATATGTCCCTGCCCTTTGTACACACCGCCCGTCGC
TCCTACCGATCGAATGATACGGTAAAGTTAACGGATCGTTTTATCTTTGGCAACATTGATATAAACTAAAAGTTATTTA
AATCTCATTGTTAGAGGAAGGAGAAGTCGAATCAAGGCCC

>**OR294052** ***Heterostelium pallidum*** **C345**
TACGGCCATGCATGTCTAAGTATAACCTTTATACGGTGAAACTGCAGACGGCTCATTACAACAGTGATAAACTAAAG
AACTTCCGCGCTTCGGCGTCTTGGATAACCGCAGTAAATCGGGGCTAATACATATAAACGAGAGGGTGAGCGGGCA
ACTGCGAACCTTTGCGATTGTTAGCTATCTTTTTCACCAACCTCTTCGGAGTTTGTGGTGAATCCGAACAATATTGCT
GATCGAAAATTTATTTTCGACGAGTTCTTTGTGTCACTGCCCTATCAACTTTCGATGGTAAGGTATTGGCTTACCATGG
TTGTAACGGGTGACGGGGAATCAGGGTTCGATTCCGGAGAGGGAGCCTGAGAAATGGCTACCACTTCTACGGAAGG
CAGCAGGCGCGCAAATTACTCAATCCCAATACGGGGAAGTAGTGACAAAAAATACTAATGCCTTTCCATATTATGGG
GGGCAATTGGAATAAGTACAACTTAAATCGCTTAGCAAAAGTGATTGGAGGGCAAGTCTGGTGCCAGCAGCCGCGG
TAATTCCAGCTCCAATAGCGTATACTAAATTTGTTGCAGTTAAAAAGCTCGTAGTTGAGATTGAGATTTCTTGGGTTTA
AGCCAGTCATAGTAGCTTTCGGGTTATTATGATTTCGGTTAAAGCTTTTGAGTGGATTTTATTTTTCACTTTTAAAGTT
ACCAAGGGATTTCCAACTGCCCATGTAAGCTGGCAACAGTTTACAATCGGGTGATCTACTGTGAGAAAATTGTAGTG
TTCAAAGCACGCGTCTTACGTTTGTTCAATGCAGCATGGTATAGTAAAATATGACACTAAATATATGTTGGTTGTATAT
TCTTAGTGTAATGACTAATAGGGAAGGGCGGGGCCCGTTCATATTGATGGGCGAGAGGTGAAATTCGTTGACCCTAT
CAAGATGCACTACAGCGAAAGCATTCGGCAAGTACCTTCTCCATTAATCAAGAACCGAAAGTTTGGGGATCGAAGA
CGATCAGATACCGTCGTAGTCCAAACCATAAACTATGTCGACCAGGGATTGGACGGATAATTTTTTAAAAACTCGCTC
AGAACCTTGTGAGAAATCATGAGTGTTTGGACTCTGGGGGGAGTATGGTCGCAAGTCTGAAACTTAAAGGAATTGA
CGGAAGGGCACACAATGGAGTGGAACCTGCGGCTTAATTTGACTCAACTCGGGAAAACTTACCAAGCTCAGATATA
ATAAGGATTGACAGACTAAAAGATCTTTCATGATTGTATAAGTGGTGGTGCATGGTCGTTCTTAGTTGGTGGAGTGAT
TTGTCTGGTCAATTCCGATAACGGACGAGACCTCTACCTGCTAACTAGTGGTATTTATTTGGTCAATATGGAAGATAG
TCATTTGGTGTTGGTGTCAGGGTCAAACTTGGCATTCTTCATTGAGTGGTGTGTATTCTGGTCAGATAGGTACTAACT
AAAAAAATAAACTTCTTAGAGGGACTACCTACCTCAAGTGGGGGGAAGTCGGAGGCAATAACAGGTCTGTGATGCC
CTTAGATACCTTGGGCTGCACGCGCGTTACAATGTAGATGAGAAAAAGGTTTCCGACATCGAAAGGTGCCGGTAATC
AATTGAATTGTCTACGTAATGGGGATTAATTTTTGTAATTATCGATTATCAACGAGGAATTCCTTGTAAGCGTAAATCA
TTACTTTACGCTGAATATGTCCCTGCCCTTTGTACACACCGCCCGTCGCTCCTACCGATCGAACGATTAGGTAAAACT
GACGGATTAGATGATTTCCTCGCAAGGGGTTATTGTTTGAGAAGTTAGTTAAATCTCATTGTTAGAGGAAGAGAAGT
CTAACCCCG

>**OR294051** ***Dictyostelium purpureum*** **C211**
CAAGCCCATGCATGTCTAAGTATAAATTCTTGTACGATGAAACTGCAGACGGCTCATTACAACAGTGATAAACTGCTA
GACTTTCGGGTTTTTAACCTTTTGGATAACCGCAGTAAATCGGGGCTAATACATAGAAGCGATGGGTGACTGGCAAC
GGAAGCTCAGCGATTATTAGCATTACTACCAATACCTTCGGGTCTTGTGGTGAAACCGAATAATATTGCAGATCGAGG
ATTTATCTTCGACAAGTCTACTGTGTCACTGCCCTATCAACTTTCGATGGTACGGTATTGGCCTACCATGGTTGTAACG
GGTAACGGGGAATTAGGGTTCGATTCCGGAGAGGGAGCCTGAGAAATGGCTACCACTTCTACGGAAGGCAGCAGG
CGCGCAAATTACTCAATCCCAATACGGGGAAGTAGTGACAATAAATATCAATACCTATCCTTAACGGAGGGCAATTGA
AATGAACACAAATTAAAACTCTTAATTAACACAATTGGAGGGCAAGTCTGGTGCCAGCAGCCGCGGTAATTCCAGCT
CCAATAGCATATACTAAAGTTGTTGCAGTTAAAAAGCTCGTAGTTGAAGTTAAAGTTTTATTGGGCTAACGTTTTTTA
CCACCCTGTGGCTAAAATAACTCCAATATTTCTTTTTTTAATAGCTCAGTTTCTAGGTCTTTGACTCTAGTTATTTGGG
CATTTCACTGTGAGAAAATTGTGGTGTTTAAAGCAGGCGTCTCGTCTGATCTTTTGCAGCATGGTATGATGGAACATG
ACATTTTACGCTATTGGTTTGCGTCTAAAGTGTAATGATTAATAGGGATGGATGGGGATGTTCATATTGGTGGGCGAG
AGGTGAAATTCGTTGACCCTATCAAGATGAACTTCTGCGAAAGCATTCATCAAATACTTCCCCATTAATCAAGAACG
AAAGTTTGGGGATCGAAGACGATCAGATACCGTCGTAGTCCAAACTATAAACTATGTCGACCAGGGATCGGCTAATA
TTTTTTTAAAATTAGTCGGCACCTTGTGAGAAATCATGAGTGTTTAGATTCCGGGGGGAGTATGGTCGCAAGTCTGA
AACTTAAAGGAATTGACGGAAGGGCACACAATGGAGTGGAGCCTGCGGCTTAATTTGACTCAACTCGGGAAAACTT
ACCAAGCTAAGATATAGTAAGGATTGACAGACTAAAAGATCTTTCATGATTCTATAAGTGGTGGTGCATGGTCGTTCT
TAGTTGGTGGAGCGATTTGTCTGGTCAATTCCGATAACGGACGAGACCTCGACCTGCTAACTAGTAGTATTTATTAGT
CGATATAGGCGATAGCTTCTCTGGGGTTGGAGTGCGGCAACGTATCTCTGCTTCAAGGAGTGTGTAGTCTGACTTGA
TAGGTACGTTAACAAAAAACTTCTTAGAGGGACTACCTGCCTCAAGCAGGCGGAAGTCCGAGGCAATAACAGGTCT
GTGATGCCCTTAGATACCTTGGGCCGCACGCGCGCTACAATGCAGATAGCAAAAAGGTTCCTGGTCTGGAAAGATTG
GGTAATCAATTGAATTTTCTGCGTAACTGGGATTGATCTTTGTAATTATTGATCATCAACGAGGAATTCCTTGTAAGCG
TAAGTCATTACCTTATGCTGAATATGTCCCTGCCCTTTGTACACACCGCCCGTCGCTCCTACCGATCGAATGATACGGT
AAAGTTAACGGATAGTTTTTTTGTGGCAACACAATTAAAATTAAAAGTTATTTAAATCTCATTGTTAGAGGAAGGAGA
AGTCGAAGCAAGACCG

>**OR295629** ***Polysphondylium fuscans*** **A241**
TCTTTTCAATTTATTTTGCGATGAGGCGAAATATATACAACTGTTGAACCAGTAAAGATAAACTAAACCGTTAATGTT
CATAAGGTTGCTTCTTTATCAGTGTTTGGAATATATAATAGAGTCGAACCAGTTCAGATATCAAATTAACATTAAAGAG
CCATTATAAGAGAGTTGTAAACTAAACCCTTGATGAAATTAAATCTATTATTCGCTTTTTCAATTGAATGTTAGATAAA
GTTTCAGGTAAAGGAAACGCCATCAATTAGAATGGTTTGACAGAATTTGGAACAAAGTCAATTATACCACTGCAAAG
ACTACTATAGATCAACTATAAAAATAATTTTACTATAATGAAATTCATTATAATAAAACTATTATACCATACGATTGTTTG
TAAAAATACTCCCAAAGGTGAATTTAAACTAATAAAAAGTTTGATATTTAATGAGAGATCGCTCTCACCCAAGTATGA
CACTTAACCCAAAGGGCCAATGCCATCATGTGCGTTCAACATTTCAACTATCCCAGTAGGCTGCAATTCAAGTGAAT
TATCGCAGTTTGCTACGGTCTTCATCGATTTAGGAGTCGAGGTATTCACCGTTTATGCTTTAGTGATTTAAAGCTTAAA
CATCTTTAAATGGTTGTTTGACATGCTAGAAAAATAATCTACAAAGAGATTAGAGTTTTTTGAGATTTTTATCAACATT
GAAAAGAATCAGTTATTATAAAATAAAGAAAGAAATCTTATGTAAACAATGTGTTTGGAATTGCAGATGGAACAAAA
AAGAACCCGTTCTTTGTAAAAAGATCAAAGGTTCAAATTTTTGTTTTCTAATTTTTTTG

>**OR295630** ***Dictyostelium robusticaule*** **B341**
TTTTTAAAAGAAACTTACCAGGTGAGGCACATATAAAGTCATGGGTTTAATAAAAAGACTAATAGGAGTAGCACTCC
ATGCTTATCAAACCGACTAAACAGATTGTTATTCTACACTGGCACACAATATAGGTTTAAATCCGAATTAAAGTCAGG
TTGCAATAGGTCTGTTAAGTCATAGCGCTCTAACTAGTGATAAATCAATTGTTAGATTACTAAAGTTAAACTCGCTAA
AGCGACCCTTAATGACTAAAATCCATATGGAAGTAGTTTAATAAAGTGGTCAGTGAGAAAACTGTGAGCAATTACTA
CGGAGTACCGTATAAATGTATAGTCCGTTAGTGCGCGAGTACCGGGAAGGTACCTTTGCAGTTAACTCGCTATACAAA
TCATTCTCAAAGAGATTCGCGCACCTAAGTGTTTGAAAATCATTTGATTATGACTGGTTTAAAAGCCACTCTCACCCA
GGTATAACATTTAATCCGTAAGGACCAATGTCATCATGTGCGTTCAACATTTCAACTATCCCAGTAGGCTGCAATTCA
AGTGAATTATCGCAGTTTGCTACGGTCTTCATCGATTTAGGAGTCGAGGTATCCACCGTTTATGCTTAAAGATTTAAA
ATTCTGTAAACATATTTTAAATGTTGTGTTTGTCGTACTAGAAAAATGGTACAAGTACCATGTGTGTAATTGAGTCGG
GATCAGTCGATAACATGTGTAACATTGCTAGATGGATTCAACACTAAAGTTGACATTTTATTACCTTTAAATAATTATA
AACCACAATAAATTGTAATAGAAACATCAAGACAACAGGACTTGAGCTAGTTTACTAGGTCACTAATTGCTTAGCAA
CTTGTTCAACTTACTCCAGACGCTATCATCTAAGATTATAAATTAAA

>**OR295628** ***Dictyostelium purpureum*** **C211**
TTCCTTCCGCTTTTTGATATGCTTAAGTTCAGCGGGTAGTCTTACTTAGATGAGGCAACATAAAAAAGTTCACGGATC
AATATTAGTCTTAATAGAATGACCGAAGTCTTTCCTGCTAATATGAACAACTATACAGTTTTAACTCATATTATTAATAT
TATACAATAACACGTCAAGCATTAATTACTAATAGAAAAACAAGTACCAGGTTATCTTACTAAATGATGAATCAAGTT
AGTTTGAAGGTTAGGAACCTATGCGTTAAACTATAAAATAATTAACTATGAGTTAATGATTATATTAATAGTATGAACT
GTAAATGTGCCGATGGAGTACCAAAAAAATGTATAGAAATTCAATAGGTTCTAAATTGAATAAATCCAATTCAGCCTA
GAGTAATAACTATACAAATCATTCCTATGTTTACCCGGAAGTATTCAGTTAGAATGATTGGTTTAAAGCCACTCTCAC
CCAAGTATAGCACTTAATCCGAAAGGACCAATGCTATCATGTGCGTTCAACATTTCAACTATCCCAGTAGGCTGCAAT
TCAAGTGAATTATCGCAGTTTGCTACGGTCTTCATCGATTTAGGAGTCGAGGTATTCACCGTTTATGCTTAATTTTTTT
AAAATAAAGCATATTTTAAATGTTTTGTTTGTCATACTAGAAAAATTAGAGATTAAATTATAATAGAGTTAGATCAGTT
GTAACAAGTGAACAGTTTTATATTGTTAATGCAGATGGATTCAAGTTTTTACACTCGACATTTTATATCCTAATCGATTA
TCACTCATCAATTTATTGAAAAAGTACACCATGATAAGGGAAGTACTAAACTATAATTATCAATCAATCATATGATCAC
TTTTAATTATAATATGTTTAAACAACCACCTATCAATGATTAATAATCTTAATAAGATAAAAAATGATCCATCCGCAGTC
CACCCTACGGATGG

>**OR295631** ***Heterostelium pallidum*** **C345**
TCGGAAACTCGCTTGGCTGATGTCGCTTTTGGAAGTGTTTATTTTAAAATGTTAAAAACTTAACAATACAGTGTTTAA
AACATTTATTAATAAGGAACCAAAGAATTTTTGTAGGTACTCAAAAAAATTTAACAACCATATTTGAGAGTTGAGTAT
CTTTTATATATGCGCTAAATTTTTTGTGTTTACCGGAAACTTTTCTCTTAAAATGAAAAATTCTCTCATTTTCCCAATTT
TTTCCCTGTGTGATAGCACTGCGAGTCTCCACTAATCTCAAACATACAATAATTACAATTTTTTAACATTTTAAAAAAT
CTCGCGAGGGGATCTTTTTCATTGTTATTTTTAATTTTTCTTTTTCCCTTTTTAATATTGTAAATCCCCTCCCTGAATTAA

CGATAACAACTTCTCACAATCCCAAATTTTCTCTCAAAAATTGAGTCTCAATCTCTAATTGTGAAAAATTTATAACAA
AAAAATTATACTCTTGCGGTGTCCTTGCGGAAACACATTGTAAAACAATATTGTGTCTCATTGTAAATGTGCATGAGA
TTACATTTTTTTTTATATTTAAGTGAAGATTCCGAATGTAATATCTAAGAAATCTTTACCAATACTTCCTTAATAGAATT
AAGAAATGATAATGAGATGCGACTCTCAACCAAGTGTGACATTTAACTCGGGGAGTCAATGTCATCATGTGCGTTCA
CATTTCGACTTTCACAGTAGTCTGCAATTCGAGTGACTTATCGCAGTTTGCTACGGTCTTCATCGATTTAGAGCCGAT
ATCCACCATTTATGCTTACTAAAATCATTGACTTTAATATTCCACAGATGAAACATCTAGACATTACATTATTTGACATA
CGAAATAATTTGAAGATGTCGCCCATTATATTTATTTTTATGAGATCCTATATAAAATATGCGCAAGTTATAAAAACTTT
TTTGAGATCTCTTTTATAGAATAGTACCCTCATCGCTCTCCACTCAAGAAA

>LC159243.1 *Polysphondylium violaceum* WS-17
CATTTTTCACTCGAAATTGATACAAAATTGAACTCTTTTGATCTTACTTGCAAAAGGAAAGAACGGTTCTTTTTTGTT
CCCCATCTGCAATCAAAAACATTGTTTACTGATTTCTTTTTATATATTTTCATATATCTGAATCTATTCAATATAATCTCA
AATTTTTTCTAGAATGACAAACAAACATTTAAAGATGTTTATAAGCTTTAAATAACTAAAGCATAAACGGTGAATACC
TCGACTCCTAAATCGATGAAGACCGTAGCAAACTGCGATAATTCACTTGAATTGCAGCCTACTGGGATAGTTGAAAT
GTTGAACGCACATGATGGCATTGGTCCTTTTGGATTAAGTGTCATACTTGGGTGAGAGCGATCTCTATATTTAATATTA
AAATTTTATTAATTAAATTTAGGTTTAATGAACTAATTTTAATATGAAGTTTATTTATTTAAACTTTTTTATAGTTGATCT
ACAGTAGTCTTTGCAGTGGTATAAAGAATAGTTTTAAACTAAACTTTAATTAAACCATTACTATATTATATGTTTATATA
ATTTAAACTATAATAATTATTCAATTGAAAATGCGAATCTTTATTAAAGTTTATTCAAGTTATATTTACTAAATATTATAAT
GGCTCATTTATAATGTTAATTTAATTAAAATTAAAGCTAATCGATATACATTTAAACACTGATTAAAGAAGTAACTTATG
AACATTAAAACGATATGTTTATCATATTGGTTCAACAGTTATATATTGTCGCCTCATCCAAGTAAGATTACCCGCTGAA
CTTAA

>MG490372.1 *Dictyostelium minimum* 2794
CGGCGTTTTTACTTAGTATATCATAGATGATAGCGTCTGGAGTAAGTTGAACAAGTTATTAAGCAATTAGTAACCTAGT
AAACTAGCTCAAGTCCTGTTGTCTTGATGTTTCTATTACAATTTATTGTGGTTTATAATTATTTAAAGGTAATAAAATGT
CAACTTTAGTGTTGAATCCATCTAGCAATGTTACACATGTTATAGACTAATCCTGACTCAATTACACACATGGTACTTG
TACCATTTTTCTAGTACGACAAACACAACATTTAAAATATGTTTACAGAATTTTAAATTTTTAAGCATAAACGGTGGAT
ACCTCGACTCCTAAATCGATGAAGACCGTAGCAAACTGCGATAATTCACTTGAATTGCAGCCTACTGGGATAGTTGA
AATGTTGAACGCACATGATGACATTGGTCCTTACGGATTAAATGTTATACCTGGGTGAGAGTGGCTTTTAAACCAGTC
ATAATCAAATGATTTTCAAACACTTAGGTGAGCGAATCTCTTTGAGAATGATTTGTATAGCGAGTTAACTGTAAAGGT
ACCTTCCCGGTACTCGCGCACTAACGGACTATACATTTATACGGTACTCCGTAGTAATTGCTCACAGTTTTCTCACTG
ACCACTTTATTAAACTACTTCCATATGGATTTTAGTCATTAAGGGTCGCTTTAGCGAGTTTAACTTTAGTAATCTAACA
ATTGATTTATCACTAGTTAGAGAGCTATAACTTAACAGACCTATTGCAACCTGACTTTAATTCGGATTTAAACCTATATT
GTGTGTCGGTGTAGAATAACAATCTGTTTAGTCGGTTTGATAAGCATGGGAGTGCTACTCCTATTAGTCTTTTTATTAA
CCCATGACTTTATATGTTGCCTCACCTTGGTAAGACTACCCGCTGAACTTAAGCATATCAAAAAGCGGAGGAAA

>MG490373.1 *Dictyostelium multiforme* 4007
CTATTACAATTTATTGTGGTTTATAATTATTTAAAGGTAATAAAATGTCAACTTTAGTGTTGAATCCATCTAGCAATGTT
ACACATGTTATCGACTGATCCCGACTCAATTACACACATGGTACTTGTACCATTTTTCTAGTACGACAAACACAACAT
TTAAAATATGTTTACAGAATTTTAAATTTTTAAGCATAAACGGTGGATACCTCGACTCCTAAATCGATGAAGACCGTA
GCAAACTGCGATAATTCACTTGAATTGCAGCCTACTGGGATAGTTGAAATGTTGAACGCACATGATGACATTGGTCC
TTACGGATTAAATGTTATACCTGGGTGAGAGTGGCTTTTAAACCAGTCATAATCAAATGATTTTCAAACGCTTATGCG
GGAGAATTTCTTTGAGAATGATTTGTATAGCGAGTTAACTGTAAAGGTACCTTCCCGGTACTCGCGCACTGACGGAC
TATACATTTATACGGTACTCCGTAGTAATTGCTCACAGTTTTCTCACTGACCACTTTATTAAACTACTTCCATATGGATT
TTAGTCATTAAGGGTCGCTTTAGCGAGTTTAACTTTAGTAATCTAACAATTGATTTATCACTAGTTAGAGCGCTATGAC
TTAACAGACCTATCGCAACCTGACTTTAATTCGGATTTAAACCTATATTGTGTGTTAGTGTAGAATAACAATCTGTTTA
GTCGGTTTGATAAGCATGGAGTGCTACTCCTATTAGTCTTTTTATTAAACCCATGACTTTATA

>AM282600.1 *Dictyostelium sphaerocephalum* 14A
CGTAGGTGAACCTGCGGATGGATCATTTTTTATCTTAATTATAATCATAGATGATAGCGTCTGGAGTAAGTTGAACAAG
TTGTTAAGCAATTAGCGACCTAGTAAACTAGCTCAAGTCCTGTTGTCTTGATGTTTCTATTACAATTTATTGTGGTTTA
TAATTATTTAAAGGTAATAAAATGTCAACTTTAGTGTTGAATCCATCTAGCAATGTTACACATGTTATCGACTGATCCC
GACTCAATTACACACATGGTACTTGTACCATTTTTCTAGTACGACAAACACAACATTTAAAATATGTTTACAGAATTTA
AATTTTTAAGCATAAACGGTGGATACCTCGACTCCTAAATCGATGAAGACCGTAGCAAACTGCGATAATTCACTTGA
ATTGCAGCCTACTGGGATAGTTGAAATGTTGAACGCACATGATGACATTGGTCCTTACGGATTAAATGTTATACCTGG
GTGAGAGTGGCTTTTAAACCAGTCATAATCAAATGATTTTCAAACACTTAGGTGCGCGAATCTCTTTGAGAATGATTT
GTATAGCGAGTTAACTGTAAAGGTACCTTCCCGGTACTCGCGCACTGACGGACTATACATTTATACGGTACTCCGTAG
TAATTGCTCACAGTTTTCTCACTGACCACTTTATTAAACTACTTCCATATGGATTTTAGTCATTAAGGGTCGCTTTAGC
GAGTTTAACTTTAGTAATCTAACAATTGATTTATCACTAGTTAGAGCGCTATGACTTAACAGACCTATTGCAACCTGAC
TTTAATTCGGATTTAAACCTATATTGTGTGCCAGTGTAGAATAACAATCTGTTTAGTCGGTTTGATAAGCATGGAGTGC
TACTCCTATTAGTCTTTTTATTAAACCCATGACTTTATATGTTGCCTCACCTTGGTAAGACTA

>DQ463371.1 *Dictyostelium macrocephalum*
TAAGTTGAACAAGGTCAATTCATTGGCTCTAGTAAACTAGCTCAAGTCCTGTTGTCTTGATGTTCTATTACAATTTATT
GTGATTTATAATTAACCAAGGTATTAAATGTCAACTTCGGTTGAATCCATCTAGCAATGTTACACATGTTATGACTGAT
CCCAACTCAATAATCTTTTGATGCTTGCATCAATTTTTCTAGTACGACAAACACAACATTTAAAATATGTTTACAGATT
TTAAATCTTTAAGCATAAACGGTGGATACCTCGACTCCTAAATCGATGAAGACCGTAGCAAACTGCGATAATTCACTT
GAATTGCAGCCTACTGGGATAGTTGAAATGTTGAACGCACATGATGACATTGGTCCTTACGGATTAAATGTTATACCT
GGGTGAGAGTGGCTTTTAAACCAGTCATAATCAAATGATTTTCAAAACGCTTGCGTGATGAATCTCTTTGAGAATGAT
TTGTATAGCGAGTTGAATGTAAAGGTACCTTCCCGGTACTTGCACACCAACGGACTATACATTTGTACGGTACTCCGT
AGTAATTGCTCACAGTTTTCTCACTGACCACTTGATTAACCTATCTCCATATGGATTTTGGTCATTAAGGGTCACTTTA
GCGAGTTTAACTTTAAGTACTCTACAATTGATTTATCACGAGTAGAAGACTTATGACCTAACAGACCTATTGTAACCT
GATCTTAATTCGGATTTAAACTTTTCAAGTGAATCAGTGTTGAATAAACAGCTGTTTAGTCGGTTTGATAAGCATGGG
TGCTACTCCTATTAGTCTTTTTATTAAACCCATGACTTTATATGTTGCCTCACCTTGGTAAGACTACCCGC

>LC159248.1 *Dictyostelium purpureum* WS-22
CTGCGGATGGATCATTTTTTATCTATTAAGATTATTAATCATTGATAGGTGGTTGTTTAAACATATTATAATTAAAAGTT
GATCGTATGATTGATTGATAATTATAGTTTAGTACTTCCCTTATCATGATGTACTTTTTCAATAAATTGATGAGTGATAA
TCGATTAGGATATAAAATGTCGAGTGTAAAAACTTGAATCCATCTGCATTAACAATATAAAAACTGTTCACTTGTTAC
AACTGATCTAACTCTATATTAATTTAATCTCTAATTTTTCTAGTATGACAAACAAAACATTTAAAATATGCTTTATTTTA
AAAAAATTAAGCATAAACGGTGAATACCTCGACTCCTAAATCGATGAAGACCGTAGCAAACTGCGATAATTCACTTG
AATTGCAGCCTACTGGGATAGTTGAAATGTTGAACGCACATGATAGCATTGGTCCTTTCGGATTAAGTGCTATACTTG
GGTGAGAGTGGCTTTAAAACCAATCATTCTAACTGAATACTTCGGTAAACATAGGAATGATTTGTATAGTTATTACTCT
AGGCTAAATTGGATTTATTCAATTTAGAACCTATTGAATTTCTATACATTTTTTTGGTACTCCATCGGCACATTTACAGT
TCATACTATTAATATAATCATTAACTCATAGTTAATTATTTTATAGTTTAACGCATAGGTTCCTGACCTTCAAACTAACTT
GATTCATCATTTAGTAAGATAACCTGGAAACTGTTTTTCTATTAGTAATTAATGCTTGACGTGTTATTTGTATGATATTA
ATAATATGAGTTAAAACTGTATAGTTGTTCATATTAGCAGGAAAGACCTCGGTCATTCTATTAAGACTAATATTGATCC
GTGACTTTTTTA

>HQ141472.1 *Dictyostelium purpureum* WS321
AAGTTGGTCAATTGACTGATAATTAATTATTGTTTAGTACTTCCCTTATCATGGTGTACTTTTTCAATTAAACGATGAGT
GATAATCTATATTAAAATGATAATAAATGTCGAGTGTAAAAAGCTTGAATCCATCTGCATTAACAATTAAAAACTGTTC
ACTTGTTACAACTGATCTAACTCTATAATTATTATTAATCTCTAATTTTTCTAGTATGACAAACAAAACATTTAAAATAT
GCTTTATTTTAAAAAAATTAAGCATAAACGGCGAATACCTCGACTCCTAAATCGATGAAGACCGCGGCAAACTGCGA
TAATTCACTTGAATTGCAGCCTACTGGGATAGTTGAAATGTTGAACGCACATGATAGCATTGGTCCTTTCGGATTAAG
TGCTATACTTGGGTGAGAGTGGCTTCAAAACCAATCATTCTGACTGAATATTTTAAAATAGTAATATTTTAGAGTAGGC
ATAGGGATGATTTGTATAGTTATGACTTGGGGCTAGATTGGCTTGACGATACCCGGAACCCCCTGGATTTCTATACAGT
TTTTTGGTGATCCATCGGCACATTTACAGTTCATAACTATTATTATTATATTTAACTCTTAGTTAATTATTTTATAGTTAAA
CGCATAGGTTCCTAACCTTCAAACTAACTTGATTTATCAACTAGCTAGATAACCTGGTTCCTGTTTTTCTATTTATTAGT
TAATGCTTGACGTGTTATATTATGATAATAATATTATGAGTTTAACCTGTATAGTTGTTCATATTAGCAGGAAGACGTGA
TCGGGCCTGTCGGGGGCCGTAAAACCCCCCCGAGTGAAAAATATAAGCCGCTGGTCGTTCTAATAAGACCTAATTTT
GAT

>LC159251.2 *Heterostelium pallidum* WS-28
GCGGATGGATCATTTCATAAAAAAAAAGAACTCAAAAAAAGTTATTTATTTAACTTTTGCCTAATTTTTTTAATTTAGTA
TTCGTATTAATTTAAATTAATTTGGGCATCTCTCTCAAAATTTATTTCGTATGTCAAATTAATTGTAATTGTCTAAGATGT
TTCCACTCTGTGGAAATATTAAAGTCAATGATTTTTAGTAAGCATAAATGGTGGATACCTCGGCTCCTAAATCGATGA
AGACCGTAGCAAACTGCGATAAGTCACTCGAATTGCAGACTACTGTGAAAGTCGAAATGTTGAACGCACATGATGA
CATTGACTCCCCCGAGTTAAATGTCACACTTGGTTGAGAGTCGCATCTCATTATCATTTCTTAATTCTATTAAGAAAGT
ATTGGTAAAGATTTCTTAGATATTACATTCGGAATCTTCACTTAAATATAAAAAAAAAAAGTAATATCAGGTACAATTCC
AATAAGAAACAAAATCGTTTTCCAATTTGTTTCCGCAAGGAAACAGCAATTGAATAATTTTTTTGTTATAAATTTTTA
ACAATTATTGATTTCGATTCAATTTTTGTGAGAAAATTTCGGATAGTACGAAGTTTTGATCGTTAATTCAAGGATGGGA
TTTACAAAATCTTAAACTGGTAATGAAAAATTAAAACTATCAATTTGAAAGATTGCCTCGCGTGATTTTTTAAAATGT
TTTAAATTAGTAATTATCGTATGTTTTTGATTATTGTAGACTTGCACTGCTTTAACACATGGTTAAAATTTTGGAAATTT
GAGAATTTTTCATATTAATTGAAATGATTCCGGTAATGACATAAAATTTTTAGCATATATAAAAGTTATTCAACTGTCAA
TTATGGTTGTTAAATTTTTTTGAGTACCTACAAAAATTCTTTGGTTCCTTATTAACAAATGTTTTAAACACTGTATTGTT
AAGTTTTTAACATTTTAAAATAAACACTTCCAAAAGCGAC

>X13160.1 *Physarum polycephalum*
TACCTGGTTGATCCTGCCAGTAGTGTATGCTTCTCCTAAAGACTAAGCCATGCATGTCTCCGAATAGAAGAGCAAGTC
TCTCTGAATCTGCGAACGGCTCCGCATACCAGTTGTAAACCATAGCAAGCAAGCCGCGTTGTTGCCGCAAGGCGAC
GGCGCGGTTCACAGGGATAACCCTGGTAATTCTGAGGCTAATACAAGAACGTACCACCCGCTTCGACCCGTAAGGG
GAGGGCGGGGGTTGTGTGACCCAGGTCGCAAATATTAACTGGGAGTGGCCACACGATCTGACCACCATACCAAACG
GTTATCCGCTTCGAAAGCTTCGGTGAGTAACGGCGGATTTCTGGGTGGCTCTCGCTGTGTGCTTCTGACCTATCAAC
TAGATGGCAGCGTAACGGACATGCCATGGTAACAACGGGTACAGAGGATAAGGGTTCGATCCTGGAGAGTGGGCCT
GAGAGATTGCTCACACTTCTAAGGAAGGCAGCAGGCGCGCAACGTTCCCATTGGGCAAAGCTCGAGGGCGTTAGG
GGACATATGAATGCCTGCCTTATGGTGGGCAATTCAAATGGGACTGTTTTAAACATCCTATCGAGTAACAATTAGAGG
ACAAGTCTGGTGCCAGCACCCGCGGTAATTCCAGCTCTAATAGCATACGTTAAAGTTGTTGCGGTTAAAACGCTCGT
AGTCGGCTCCAGACCTTCAGAGCCTTGATTCGGGACACTGGGTCAGCTGCTCTTCTCCAAGAGCGCGCCAACTACT
GATGATAGGGGACGTCGGTCAATCGTGCCCTGGGCTGTCTCCCCCCCGGGGGAGTCACAGGGTCGTTCAGCGGTCT
TCGCGATCGCGGTTCGGCTCGGCTCGGGGTACCAATCACCATGATTAAACCGTAGTGACCAAAGCACGTCTTTAGAC
GGGCACGGCACAGCATGGGACGAAACGCACCGGGCTCGCCTTTTTTTTGCGGGGGCGTGACTCGGTAAAAGCGAA
AGGGATGTTCGAGGGTGACCGAATTGCTGGGCGAGTGGTGAAATACGTTGACCCTAGCAAGTCGACCAAAGGCGTA
AGCAGTCATCAAGGGCATTCCCGTTGATCAAGAGCGAAAGTTAAGGGTTCGAAGACGATCAGATACCGTCGTAGTC
TTAACTATAAATGATGCAGACCAGGGATAGGACAGTGTCCATCTCGACTCTTCCGGACCTTGGAGAAATCACGAGTC
TATGGGTTCTGGGGGGAGTATGGTCGCAAGGCTGAAACTTAAAGGAATTGACGGAAGGGCACACAAAGAGTGGAA
CCTGCGGCTTAATTTGACTCAACACGGGAAAACTCACCAGGTCCGGATACACGTATGAAAGTCAAGCTGAAAGACT
TTACTCAATGATGTAAGTGGTGGTGCATGGTCGTTCTTAGTTCGTGGATTGATTTGTCTGGTTTATTCCGATAACGAGC
GAGACCCCGGCGTTCCTAATAGGGGTGGCAGCCAGACCGGTCGCAAGACAGGTTAGCTCGCCACCTGAAGTTATGC
TTCTTAGACGTATCAGAGCCGATAAGGTTCTTGAAATGGGTTAATAACAGGTCAGTCATGCCCTTAGATGTTCTGGGC
CGCACGCGCGTTACAATGGCATGTAAAACGAGTGCTTGAACAAGGCGTCCCACGGCCGAAAGGTCGTGGTAACCCT
TAGTCCCTGCTTTGACTGGGACAGATCTTTGCAATTATTGGTCTCAAACGAGGAATTTTTAGTAATCGCAGGTCATTA
ACCTGCGTTGAATGCGTCCCTGCCCTTTGTACACACCGCCCGTCGCTGCTACCGATTGGGTTTTACAGTTACGCGTTC
GGAGGACGATGTTTTCGGGTCTTAACGGGCTCCGGGGCGTCGGCCAAAGTTCTAACTACTGTACGGCCTAGAGGAA
GCAGAAGTCGTAACAAGGTAATCGTAGGTGAACCTGCGTTTGGATCATTA
